# Metal‐Free and Open‐Air Arylation Reactions of Diaryliodonium Salts for DNA‐Encoded Library Synthesis

**DOI:** 10.1002/advs.202202790

**Published:** 2022-07-19

**Authors:** Hongtao Xu, Tingting Tan, Yiyuan Zhang, Yan Wang, Kangyin Pan, Ying Yao, Shuning Zhang, Yuang Gu, Wanting Chen, Jie Li, Hewei Dong, Yu Meng, Peixiang Ma, Wei Hou, Guang Yang

**Affiliations:** ^1^ Shanghai Institute for Advanced Immunochemical Studies ShanghaiTech University Shanghai 201210 P. R. China; ^2^ College of Pharmaceutical Science and Institute of Drug Development & Chemical Biology Zhejiang University of Technology Hangzhou 310014 P. R. China; ^3^ Shanghai Key Laboratory of Orthopedic Implants Department of Orthopedic Surgery Shanghai Ninth People's Hospital Shanghai Jiao Tong University School of Medicine Shanghai 200011 P. R. China

**Keywords:** acylation, DNA, DNA‐encoded library, phenol, oxime

## Abstract

A successful DNA‐encoded library (DEL) will consist of diverse skeletons and cover chemical space as comprehensive as possible to fully realize its potential in drug discovery and chemical biology. However, the lack of versatile on‐DNA arylation methods for phenols that are less nucleophilic and reactive poses a great hurdle for DEL to include diaryl ether, a privileged chemotype in pharmaceuticals and natural products. This work describes the use of “substrate activation” approach to address the arylation of DNA‐conjugated phenols. Diaryliodonium salt, a highly electrophilic and reactive arylation reagent, is employed as Ar^+^ sources to ensure highly selective on‐DNA arylation of phenols and oximes with both high yields and DNA fidelity. Notably, the new on‐DNA arylation reaction can be applied to the late‐stage modification of peptides containing tyrosine side‐chain and to synthesize DNA‐tagged analogues of existing drug molecules such as sorafenib, a known pan‐kinase inhibitor. The new on‐DNA diaryliodonium salts chemistry affords a greater flexibility in DEL design and synthesis.

## Graphical abstract

The first on‐DNA diaryliodonium slats (DAIs) chemistry is developed by substrate activation strategy. The selective on‐DNA arylation methods of phenols and oximes with both high yield and DNA fidelity are developed. The applicable potentiality of these reactions is demonstrated by pilot on‐DNA synthesis of analogs of clinically used drug sorafenib and late‐stage modification of peptides.

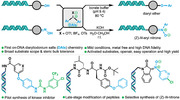

## 1 Introduction

Since its conception by Lerner and Brenner in 1992,^[^ ^1^ ^]^ DNA‐encoded library (DEL), which provides a general way to bridge chemistry and biology,^[^ ^2^, ^3^ ^]^ has become one of the most powerful techniques for the discovery of hit compounds and/or bioactive probes in medicinal chemistry and chemical biology. Generally, DEL has significant advantages such as a huge library size, comprehensive chemical space, and lower cost. It is a bench‐top technique that obviates the need of costly and huge high through‐put screening (HTS) infrastructures, and can easily be accessed by both biotech startups and academic institutions. A large number of hits have been identified from DEL for various disease targets as well as for chemical probes.^[^ ^4^, ^5^, ^6^ ^]^ Notably, some drug candidates originated from DEL hits have already entered into clinical testing for various diseases.^[^ ^7^ ^]^ Nowadays, DEL is becoming an indispensable technology for hit discovery campaigns either in big pharmaceutical companies or academic laboratories.^[^ ^8^, ^9^, ^10^, ^11^, ^12^, ^13^, ^14^, ^15^, ^16^, ^17^ ^]^

**Figure 1:**
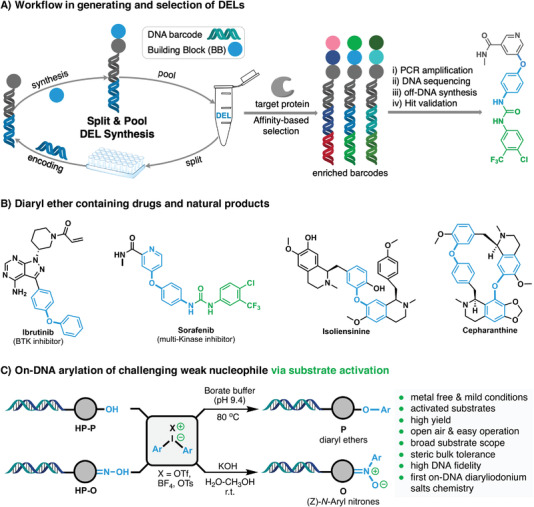
A) Workflow in generating and selection of DELs. B) Diary ether containing drugs and natural products. C) On‐DNA arylation of weak nucleophiles via substrate activation.

DEL is a collection of small molecules in which each of the compounds is annotated by a unique DNA barcode,^[^ ^18^ ^]^ enabling a single‐pooled selection of millions or billions of compounds against a specific target protein. Typically, DEL can be designed and synthesized using the strategy of “Split and Pool” (**Figure** **1**A). Generally, chemical building blocks (BBs) and DNA barcodes can be assembled efficiently by 3 to 4 cycles of interactive enzymatic‐encoding and DNA‐compatible reactions, and pooled together to generate the corresponding DEL. After panning against a target protein, DNA barcodes of enriched DELs are PCR amplified and DNA sequenced. Chemical structures of hit compounds with high‐affinity, thus, can be de‐convoluted and validated through subsequent off‐DNA synthesis.

Despite many important progresses in DEL, there are still some obstacles that hinder the realization of DEL's full potential.^[^ ^19^, ^20^ ^]^ One of the most fundamental bottlenecks is the availability of DNA‐compatible chemical reactions that can provide more flexibility in DEL's design and synthesis. In fact, the idiosyncratic nature of oligonucleotide (DNA barcode) imposes a stringent restriction on the reaction conditions in DEL synthesis, such as highly diluted aqueous media, high chemo‐selectivity, etc., so as to keep the integrity and maintain the genetic information of DNA barcodes. As a consequence, most of the conventional organic reactions are excluded from the toolbox of DEL chemistry. Researchers from academia and industry have since developed various DNA‐compatible reactions such as on‐DNA amide formation,^[^ ^21^, ^22^ ^]^ diazo‐transfer,^[^ ^23^ ^]^ cross‐coupling reaction,^[^ ^24^, ^25^, ^26^, ^27^, ^28^, ^29^ ^]^ C‐H activation and functionalization,^[^ ^30^, ^31^, ^32^, ^33^, ^34^, ^35^ ^]^ ring‐closing metathesis,^[^ ^36^, ^37^ ^]^ Sulfur‐Fluoride Exchange (SuFEx) chemistry,^[^ ^38^ ^]^ photo‐catalyzed reaction,^[^ ^39^, ^40^, ^41^, ^42^, ^43^, ^44^, ^45^ ^]^ and cycloaddition reactions.^[^ ^46^, ^47^, ^48^, ^49^, ^50^ ^]^ Recently, the strategies of micellar‐mediated on‐DNA synthesis,^[^ ^51^, ^52^ ^]^ using chemically stabilized DNA barcodes,^[^ ^53^ ^]^ solid support mediated on‐DNA synthesis,^[^ ^54^ ^]^ and especially the reversible adsorption of DNA on solid supports have greatly expanded the on‐DNA synthetic toolbox.^[^ ^55^, ^56^, ^57^ ^]^ More importantly, some of them have already been applied in DEL synthesis.^[^ ^58^ ^]^

Among various DNA‐compatible reactions, methods for C(sp^2^)‐*O* bonds formation are of significant importance due to the fact that diaryl ethers are privileged motifs in pharmaceuticals and natural products (Figure 1B). However, the existing synthesis toolbox of DEL lacks an efficient method to prepare compounds containing this skeleton, as evident in that only one example involving the reaction of special DNA‐tagged electron‐deficient pyrimidyl ammonium salts with phenol or alcohol has been reported.^[^ ^59^ ^]^ The C(sp^2^)‐hetero (N, O, and S) bonds are traditionally constructed mainly through transition‐metal‐catalyzed cross‐coupling reactions. Up to now, although the metal‐catalyzed version of on‐DNA C(sp^2^)‐hetero bonds formation have elegantly developed by Lu,^[^ ^60^ ^]^ Berst,^[^ ^61^ ^]^ Torrado,^[^ ^62^ ^]^ Simmons,^[^ ^63^ ^]^ Dawson,^[^ ^55^ ^]^ Chen,^[^ ^64^ ^]^ Liu,^[^ ^65^ ^]^ Pentelute^[^ ^66^ ^]^ and their coworkers as well as our group,^[^ ^26^, ^32^, ^67^ ^]^ the coupling partners are still limited to amines or thiophenols that are highly nucleophilic. For phenolic substrates, the inherently weak nucleophilic activity endows them with poor reactivity in the *O*‐arylation reaction. Interference of DNA bases and amide linkage makes it even more difficult to achieve *O*‐arylation with high chemo‐selectivity. An efficient, selective and operationally simple *O*‐arylation reaction is, thus, highly desirable to unlock the chemical space of diaryl ethers in DEL.

Apart from the classical arylation reaction via cross‐coupling, diaryliodonium salts (DAIs) have received increasing attention as versatile arylation reagents for their excellent merits including being air‐ and water‐stable, water‐soluble, and highly electrophilic and reactive.^[^ ^68^ ^]^ These merits make DAIs able to proceed arylation reactions with weak nucleophiles under metal‐free conditions.^[^ ^69^ ^]^ We envisaged that the weakly acidic properties of phenols (p*K*a ≈ 9.94) could make them readily dissociate into anionic forms of ArO^−^ under weakly basic conditions, thus offering an opportunity for them to outcompete the DNA bases and amide competitors when combined with DAIs. Taking these observations into account and to continue our ongoing effort in DEL synthesis and selection,^[^ ^5^, ^34^ ^]^ we reported herein the address of the sluggish issue of the arylation of DNA‐conjugated phenolic compounds via a substrate activation strategy (umpolung). We employed the highly electrophilic and reactive DAIs as Ar^+^ sources to replace Ar‐X (X = halogen, OTf, OFs, activated by metal to form Ar‐M‐X, Ar^−^) in cross‐coupling, thus converting the conventional cross‐coupling pathway into concerted nucleophilic aromatic substitution (*CS_N_Ar*) pathway (Figure 1C),^[^ ^69^ ^]^ and, at the same time, obviates the need to activate the aromatic ring by using highly electron‐withdrawing substituents such as those in traditional *S_N_Ar* reactions. We demonstrated that this new strategy could achieve highly selective and efficient on‐DNA *O*‐arylation of phenols with high yields and DNA fidelity. More importantly, the new DEL chemistry of DAIs adopts a metal‐free and open‐air reaction condition, which effectively eliminates the potential of metal‐induced DNA degradation and by‐products of dehalogenation. Moreover, the same strategy could be extended to the on‐DNA metal‐free *N*‐arylation of isatin oximes. We demonstrated that the highly versatile and biological useful (*Z*)‐*N*‐aryl nitrones could be readily incorporated into DEL by the on‐DNA DAIs reaction.

## 2 Results and Discussion

Initially, to test the above hypothesis, DNA‐conjugated phenol (HP‐P1) and diaryliodonium salt (DAI‐a) were chosen as the model substrates to optimize the on‐DNA reaction conditions. A preliminary screening of the reaction temperature indicated that the desired product P1a could be obtained in 65% yield at 80 °C when NaOH (800 equiv.) was employed, along with 21% of a by‐product which has the same molecular weight as P1a. This by‐product is most likely an amide arylated product (Table S1, Supporting Information, entries 2–4), which might be reduced by using a slightly weaker base. The screening of a series of borate buffers showed that the desired P1a could be obtained in an increased yield of 73% when a pH 9.4 borate buffer (250 mm) was used (Table S1, Supporting Information, entry 7). Further investigation of the solvent ratio of DMA and borate buffer suggested that increasing the ratio of DMA/borate buffer to 4:1 could further increase the yield of P1a to 80% (Table S1, Supporting Information, entry 8). In addition, when reducing the amount of DAI‐a to 200 equiv., and performing the reaction in a 2:3 DMA‐borate buffer (pH 9.4), the desired P1a was obtained in a satisfactory yield (87%, Table S1, Supporting Information, entry 1). Reducing reaction temperature and shortening reaction time both resulted in negative effects on the reaction (Table S1, Supporting Information, entries 11 and 12). The final on‐DNA product P1a was further confirmed by co‐injection experiment (Figure S3, Supporting Information).

**Figure 2:**
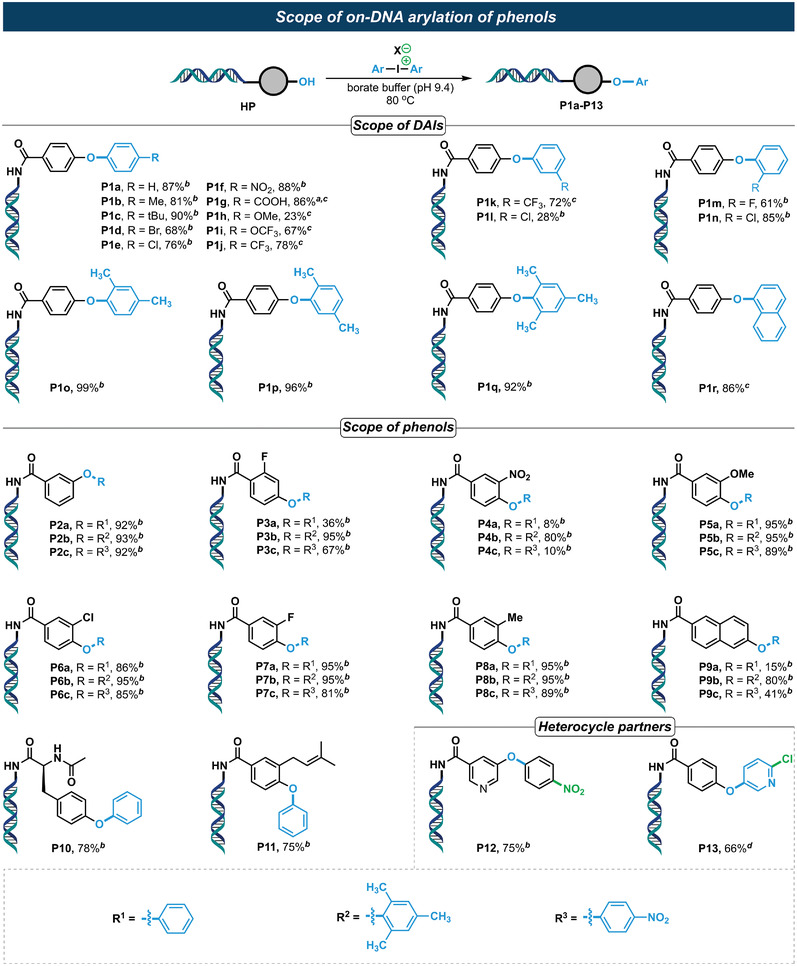
On‐DNA arylation of phenols by using diaryliodonium salts. Reaction conditions: 1 equiv. of HP (1 mm in *dd*H_2_O, *dd*H_2_O = double distilled H_2_O), 200 equiv. of DAI (500 mm in DMA) in total volume of 50 µL aqueous solutions (DMA:borate buffer [pH 9.4] = 2:3), 80 °C, 2.5 h. X = OTf, OTs, or BF_4_. The conversion yield of HP was determined by LC‐MS. *^a^* Bis(4‐(methoxycarbonyl)phenyl)iodonium salt was used for the reaction, and p1g is the ester hydrolyzed product. *^b^* The counterion of DAI is OTf. *^c^* The counterion of DAI is BF_4_. *^d^* The counterion of DAI is OTs.

With the optimized reaction condition in hand, we subsequently examined the substrate scope of DAIs in the on‐DNA *O*‐arylation reaction. As expected, most of the DAIs delivered the corresponding *O*‐arylation products in good to excellent yields (**Figure** **2**). Both electron‐donating (P1b‐P1c), electron‐withdrawing (P1f, P1g and P1i‐P1k) and halogen (P1d, P1e, P1l‐P1n) substituents were well tolerated, delivering the corresponding arylation products in good to excellent yields. However, the *para*‐methoxy substituted DAI afforded the corresponding arylation product P1h in a poor yield of 22%. It was noted that sterically hindered *ortho*‐monomethyl and *ortho*‐dimethyl substituted DAIs, in accordance with the DAIs’ scope in conventional off‐DNA synthesis, delivered the corresponding diaryl ethers (P1o–P1q) in good to excellent yields, which is a great challenge to conventional metal‐catalyzed *O*‐arylation reactions as well. Importantly, the tolerance of ‐NO_2_ (P1g) provides a valuable anchor that can be reduced by the known on‐DNA nitro reduction,^[^ ^70^ ^]^ thus allowing further diversification of the diaryl ether skeleton by other known on‐DNA syntheses such as amide formation,^[^ ^21^ ^]^ and Buchwald–Hartwig amination reactions.^[^ ^26^ ^]^

The investigation of substrate‐scope regarding DNA‐tethered (hetero)aryl phenols (Figure 2) indicated that the majority of tested phenols reacted readily with the three representative DAIs, delivering the corresponding diaryl ethers with good to excellent yields. Substituents of electron‐donating, electron‐withdrawing, halogen and vinyl group (P11) were all well tolerated, except for *ortho*‐nitro substituted DNA substrate, which gave poor yields when reacted with phenyl diaryliodonium salts (P4a) and *para*‐nitrophenyl diaryliodonium salts (P4c), but high yield for *ortho*‐dimethyl substituted DAI (P4b) despite the large steric hindrance in both of the DNA‐conjugated phenol and the DAI reagent. DNA‐conjugated naphthol could also deliver the corresponding arylation product in moderate to good yields (P9b and P9c), except for the reaction with diaryliodonium salt DAI‐a (P9a). Significantly, the reaction of either DNA conjugated heteroaryl phenol or asymmetric heteroaryl DAI proceeded well under the optimized reaction conditions, delivering the corresponding heterocycle‐containing products in good yields (P12 and P13). Particularly, the reactive chloride, an important anchor widely used in DEL synthesis, at C2′ of DAI‐s, was spared in the *O*‐arylation reaction, highlighting its great potential in DEL chemistry. The present results also indicated that the counterions (OTf, OTs and BF_4_) of DAIs showed no apparent influence on the on‐DNA *O*‐arylation reactions that have been tested.

*N*‐aryl nitrones are a family of highly useful substrates in various organic transformations such as 1,3‐dipoles cycloaddition,^[^ ^71^ ^]^ nucleophilic addition,^[^ ^72^, ^73^ ^]^ C−H activation,^[^ ^74^ ^]^ rearrangement reactions,^[^ ^75^ ^]^ etc. They have also been demonstrated to be invaluable in the synthesis of heterocycle and natural products.^[^ ^76^, ^77^ ^]^ More importantly, nitrone is a known pharmacophore, and many nitrone‐containing compounds exhibiting a wide range of biological activities such as antioxidation,^[^ ^78^, ^79^, ^80^ ^]^ free radical scavenging^[^ ^81^ ^]^ and antiproliferative activity.^[^ ^82^ ^]^ Conventional strategies to prepare *N*‐aryl nitrones mainly include: i) oxidation of aryl imines and aryl amines;^[^ ^83^ ^]^ ii) condensation of ketones with *N*‐arylhydroxylamines;^[^ ^84^ ^]^ and iii) copper‐promoted *N*‐arylation of oximes with aryl boronic acids.^[^ ^85^ ^]^ However, the inherent harsh reaction conditions for these strategies such as the need for an excess amount of copper salts, poor functional group tolerance, etc., pose a great challenge to their application in DEL, in which chemical reactions are carried out in the presence of oligo DNA. Encouraged by the excellent on‐DNA *O*‐arylation reaction profile of DAIs, we tested if the same substrate activation strategy of DAIs could be extended to direct *N*‐arylation of oximes.

DNA‐conjugated 2‐(hydroxyimino)indolin‐2‐one (HO1) and diphenyliodonium salt (DAI‐a) were chosen as the model substrates to optimize the on‐DNA reaction conditions. As illustrated in Table S2, Supporting Information, we systematically investigated various reaction conditions such as base, solvent, and the amount of DAI‐a. In the presence of 2000 equiv. of KOH and 500 equiv. of DAI‐a in a mixture of *dd*H_2_O/MeOH (1:4), the reaction proceeded smoothly and delivered the corresponding DNA‐tethered *N*‐arylation product O1a in 82% yield. In addition, the final product was identified as a stereo‐specific *Z* isomer by the co‐injection experiment (Figure S4, Supporting Information).

**Figure 3:**
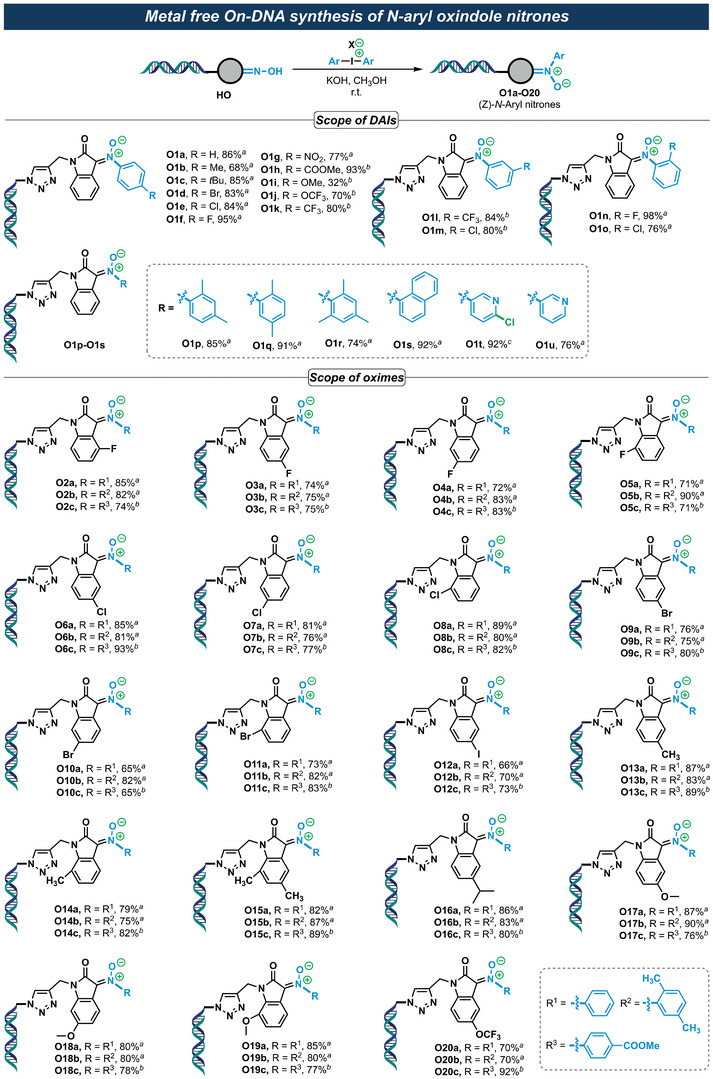
Metal‐free on‐DNA synthesis of (Z)‐*N*‐aryl oxindole nitrones. Reaction conditions: 1 equiv. of **HO** (1 mm in *dd*H_2_O), 500 equiv. of DAI (500 mm in MeOH), 2000 equiv. of KOH (500 mm in *dd*H_2_O, *dd*H_2_O = double distilled H_2_O) in a total volume of 50 µL aqueous solution (*dd*H_2_O/MeOH = 1:4), r.t., 2 h. X = OTf, OTs, or BF_4_. The conversion yield of HO was determined by LC‐MS. *^a^* The counterion of DAI is OTf. *^b^* The counterion of DAI is BF_4_. *^c^* The counterion of DAI is OTs.

With the optimized reaction condition in hand, we subsequently examined the substrate scope of different DAIs in the on‐DNA *N*‐arylation reactions. As illustrated in **Figure** **3**, most of the DAIs could deliver the corresponding (Z)‐*N*‐aryl nitrones in good to excellent yields. Alkyl (O1b and O1c), electron‐withdrawing (O1g, O1h, and O1j–O1l), and halogen (O1d–O1f, O1m–O1o) substituents were well tolerated. The *para*‐methoxy substituted DAI, on the other hand, delivered the corresponding products (O1i) in relative low yield, suggesting it is not a suitable reaction partner of *N*‐arylation for DEL chemistry. Significantly, DAIs bearing sterically hindered *ortho*‐ mono‐substituents (O1n–O1q) or *ortho*‐ dimethyl substituents (O1r) also afforded the corresponding (Z)‐*N*‐arylation products in moderate to excellent yields. Notably, the reaction showed a broad functional group tolerance including sensitive substituents like nitro (O1g), ester (O1h), and heterocycles (O1t and O1u). Similar to the aforementioned *O*‐arylation reaction of DAIs (Figure 2, P13), the reactive chloride at C2′ of the asymmetric DAI was also spared in (Z)‐*N*‐arylation (O1t). The substrate scope of DNA‐conjugated 2‐(hydroxyimino)indolin‐2‐one (HO) (Figure 3) was investigated and shown a broad reactivities with the three representative DAIs (‐H, ‐Me, and ‐COOMe substituted) under the optimized on‐DNA reaction condition, delivering the corresponding (Z)‐*N*‐arylation products in good to excellent yields. Substrates with halogen (O2a–O12c), alkyl (O13a–O16c), electron‐donating (O17a–O19c), and electron‐withdrawing (O20a–O20c) substituents were all well tolerated. Notably, just like the on‐DNA *O*‐arylation reaction, the counterions (OTf, OTs, and BF_4_) of DAIs showed no apparent influence on the on‐DNA *N*‐arylation of isatin oximes.

**Figure 4:**
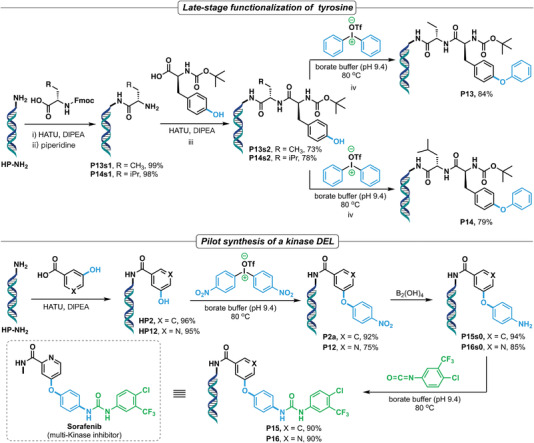
Synthetic utility of the DAI chemistry in DEL rehearsal: late‐stage functionalization of peptides and pilot synthesis of a kinase‐targeting DEL.

To further explore this new on‐DNA arylation chemistry of DAIs, its application in late‐stage arylation of tyrosine on DNA‐conjugated dipeptides was evaluated. The headpiece DNA (HP‐NH_2_) was first coupled with Fmoc‐Abu‐OH and Fmoc‐Leu‐OH under the standard amide coupling and deprotection conditions, delivering P13s1 and P14s1 in excellent yields (**Figure** **4**). Amide coupling of P13s1 and P14s1 with Boc‐Tyr‐OH provided dipeptides P13s2 and P14s2 in 73% and 78% yields, respectively. Under the optimal *O*‐arylation condition, both dipeptides reacted smoothly with DAI‐a, delivering the corresponding arylation products P13 and P14 in 84% and 79% yields, respectively. Moreover, this chemistry was evaluated in the synthesis of a focused DEL library of sorafenib, a known anti‐cancer drug with inhibitory activity to multiple tyrosine kinases, as the key step. In a pilot experiment, two DNA‐conjugated sorafenib analogues (P15 and P16) were synthesized and both showed satisfactory yields in a four‐step reaction including: i) amide coupling of HP‐NH_2_ with phenols (HP2 and HP12); ii) arylation of phenol with *para*‐nitrophenyl diaryliodonium salt (P2a and P12); iii) reduction of nitro group (P15s0 and P16s0); and iv) installation of the urea functionality. The established reaction route could be used as a template in the assembly of a sorafenib‐like DEL of large structural diversity by using various building blocks in each step of the synthesis. Taken together, as a new DEL chemistry, the excellent reaction profile of the on‐DNA arylation reactions of DAIs’ endows the method with a great potential to be widely used in DEL's design and synthesis.

**Figure 5:**
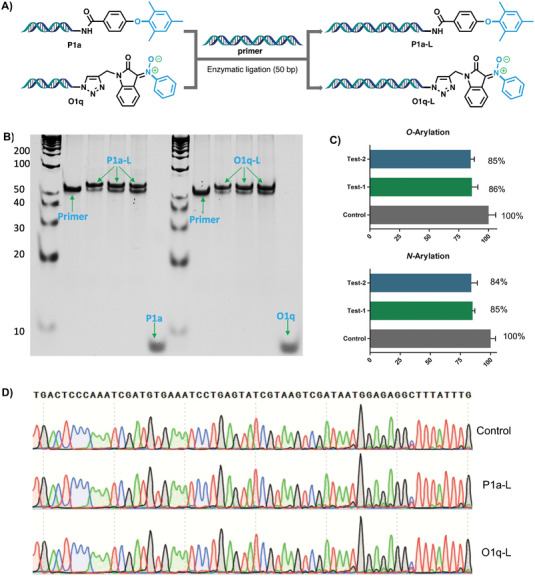
Validation of the integrity of the oligo DNA barcode from the samples of enzymatic ligation. A) DNA ligation. B) DNA ligation analysis of the final products of on‐DNA *O*‐arylation and *N*‐arylation. (2 repeat). C) qPCR analysis of residual amplifiable material after exposing to the reaction conditions of on‐DNA *O*‐arylation and *N*‐arylation. D) Sanger sequencing results of the on‐DNA *O*‐arylation and *N*‐arylation.

In accordance with one of the most critical criteria in DEL synthesis, the integrity of DNA barcodes in the DAI mediated on‐DNA *O*‐ and *N*‐arylation reactions was also evaluated. In a typical experiment, aliquots of DNA‐conjugated diaryl ether P1a and nitrone O1q, final products of on‐DNA *O*‐ and *N*‐arylation reactions, were subjected to T4 DNA ligase‐catalyzed ligation with a 50‐basepair DNA sequence, producing the corresponding products P1a‐L and O1a‐L, respectively (**Figure** **5**A). Gel electrophoresis analysis of both compounds showed complete ligation (Figure 5B). Furthermore, DNA degradation under the chemical reaction conditions was analyzed by real‐time polymerase chain reaction (qPCR). A model DEL library was exposed to the chemical reaction conditions, and followed by PCR amplification. Quantification of amplifiable material in the testing samples indicated no significant DNA degradation. Under the standard reaction conditions of *O*‐arylation and *N*‐arylation, DEL library showed ca. 86% and 85% in remaining amplifiable material (Figure 5C), respectively, greatly exceeding the acceptable threshold of 30% required in DEL synthesis.^[^ ^86^ ^]^ Sanger sequencing of the DNA‐tags of P1a‐L a nd O1q‐L showed no mutaion of the DNA sequences after the reaction, indicating the on‐DNA *O*‐arylation and *N*‐arylation reactions maintain the DNA fidelity (Figure 5D).

## 3 Conclusion

To circumvent issues concerning weak nucleophilicity and sluggish reactivity associated with phenol‐like chemotypes in cross‐coupling reaction and hostile conditions for DNA fragments in the conventional metal‐catalyzed coupling reaction, a “substrate activation” approach was developed for DEL chemistry. Diaryliodonium salt, a highly reactive reagent in arylation, was examined in DEL application. A broad spectrum of phenols and isatin oximes were shown to be active in the reaction with DAIs in the presence of DNA oligos, affording biologically important diaryl ether and nitrone containing molecules efficiently. Inclusion of the pharmaceutically privileged scaffolds into DEL underline the importance of the on‐DNA DAI chemistry. Furthermore, for the first time, we successfully applied this approach in the late‐stage arylation of dipeptides and in the synthesis of sorafenib analogues, demonstrating the versatility of this method in focused DEL library construction.

## Conflict of Interest

The authors declare no conflict of interest.

## Supporting information

Supporting Information

## Data Availability

The data that support the findings of this study are available in the supplementary material of this article.
