# Correction to “Metal‐Free and Open‐Air Arylation Reactions of Diaryliodonium Salts for DNA‐Encoded Library Synthesis”

**DOI:** 10.1002/advs.202413292

**Published:** 2024-11-08

H. Xu, T. Tan, Y. Zhang, Y. Wang, K. Pan, Y. Yao, S. Zhang, Y. Gu, W. Chen, J. Li, H. Dong, Y. Meng, P. Ma, W. Hou, G. Yang, Metal‐Free and Open‐Air Arylation Reactions of Diaryliodonium Salts for DNA‐Encoded Library Synthesis, *Adv. Sci*. (**2022**), *9*, 2202790. https://doi.org/10.1002/advs.202202790.

In page 1, the author affiliation section, Tingting Tan's affiliation “Shanghai Institute for Advanced Immunochemical Studies ShanghaiTech University, Shanghai 201210, P.R. China.” was not corrected.

This should be revised to “Shanghai Institute for Advanced Immunochemical Studies and School of Life Science and Technology, ShanghaiTech University, Shanghai 201210, China.”

We apologize for this error.

