# Supplementary material for: Metal‐Free and Open‐Air Arylation Reactions of Diaryliodonium Salts for DNA‐Encoded Library Synthesis
Source: Adv Sci (Weinh). 2022 Jul 19;9(26):2202790. doi: 10.1002/advs.202202790 (PMC9475524; doi:10.1002/advs.202202790)

ADVANCED SCIENCE
Open Access

Supporting Information

for *Adv. Sci.*, DOI 10.1002/advs.202202790

Metal-Free and Open-Air Arylation Reactions of Diaryliodonium Salts for DNA-Encoded Library Synthesis

Hongtao Xu, Tingting Tan, Yiyuan Zhang, Yan Wang, Kangyin Pan, Ying Yao, Shuning Zhang, Yuang Gu, Wanting Chen, Jie Li, Hewei Dong, Yu Meng, Peixiang Ma*, Wei Hou* and Guang Yang**

Supporting Information

Metal-Free and Open-Air Arylation Reactions of Diaryliodonium Salts for DNA-Encoded Library Synthesis

Contents

I. General Information......S3

II. Abbreviations......S4

III. Experimental Procedures and Characterizations......S5

IV. Co-Injection Experiment......S31

V. Evaluation of the DNA Tags Stability......S35

VI. Reference......S40

VII. Copies of ^{1}H, ^{13}C NMR, HPLC and MS Spectra......S42

I. General Information

All commercially available organic compounds were purchased from Sigma-Aldrich, and oligonucleotides were purchased from Shanghai AngewGen Biotech Co., Ltd. in China. Unless otherwise noted, all commercial reagents and solvents were used without additional purification. The substrates **1** were prepared according to literature methods.[1] Water was purified with a Millipore Milli-Q system. NMR spectra were recorded on Bruker AM-500 instruments. Chemical shifts are reported in δ (ppm) referenced to TMS as an internal standard for ^{1}H NMR and $CDCl_3$ (δ 77.0) or DMSO-d_6 (δ 39.5) for ^{13}C NMR. High-resolution mass spectra were obtained on High-resolution mass spectra (HRMS-ESI) were obtained on an Agilent Technologies 6230 Accurate Mass TOF LC/MS instrument or an AB Sciex 4600 QTOF MS instrument.

All reagents and DNA headpiece HP-NH2 (5’- / 5phos / GAGTCA / iSp9 / iUniAmM / iSp9 / TGACTCCC-3’, Figure 1) were obtained from commercial sources unless otherwise noted and used as received. All on-DNA reactions were performed in 1.5 mL or 5 mL Eppendorf tubes. On-DNA reactions in the studies of reaction condition optimization and substrate scope extension were analyzed by UPLC-MS. Typically, samples were dissolved in an appropriate amount of distilled and deionized water (ddH_2O) and injected into a reverse-phase chromatography column (Xbridge Oligonucleotide BEH C18 column, 1.7 µm, 2.1×50 mm). The elution was carried out as followings: 5–95% solvent B over 4.5 min, 0.4 mL/min, λ = 260 nm; solvent A: 0.75% v/v hexafluoroisopropanol/ 0.038% v/v triethylamine in methanol/water = 5/95; solvent B: 0.75% v/v hexafluoroisopropanol/ 0.038% v/v triethylamine in methanol/water = 90/10. The effluents were analyzed by a Xevo G2-XS Q-TOF with electrospray ionization source was used for detection. On-DNA reaction yield calculation: Ignoring UV coefficient difference for all DNA products and assuming 100% of DNA total recovery, the yield of DNA products were determined from UV absorbance trace (260 nm) peak area.

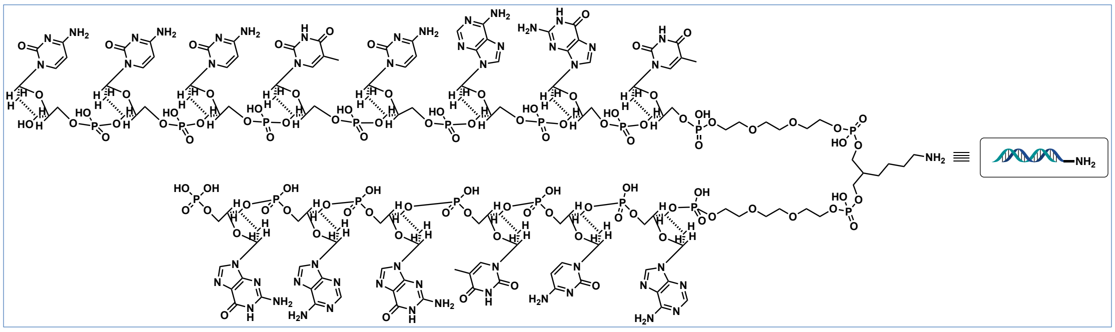

(5′/5Phos/GAGTCA/iSp9/iUniAmM/iSp9/TGACTCCC-3′)
(MW: 4937.23)

Figure S1. The Structure of Headpiece DNA (**HP-NH_2**).

II. Abbreviations

$BF_3\cdot OEt_2$: boron trifluoride ether complex
DAI: diaryliodonium salts
DCM: dichloromethane
DCE: 1,2-dichloroethane
ddH_2O: double distilled waster
DIPEA: *N,N*-diisopropylethylamine
DMA: *N,N*-dimethylacetamide
DMAP: 4-dimethylaminopyridine
DMF: *N,N*-dimethylformamide
DMSO: dimethyl sulfoxide
EDC: 1-Ethyl-3-(3-dimethylaminopropyl)carbodiimi
HATU: 2-(7-Azabenzotriazol-1-yl)-N,N,N',N'-tetramethyluronium hexafluorophosphate
HOAc: acetic acid
m-CPBA: *m*-chloroperbenzoic acid
MTBE: methyl *tert*-butyl ether
THF: tetrahydrofuran
PBS: phosphate buffer saline
PivOH: pivalic acid
*s*NHS: N-Hydroxysulfosuccinimide sodium salt
TfOH: trifluoromethanesulfonic acid
TMP-H: 1,3,5-trimethoxybenzene
$TsOH\cdot H_2O$: *p*-toluenesulfonic acid monohydrate

III. Experimental Procedures and Characterizations

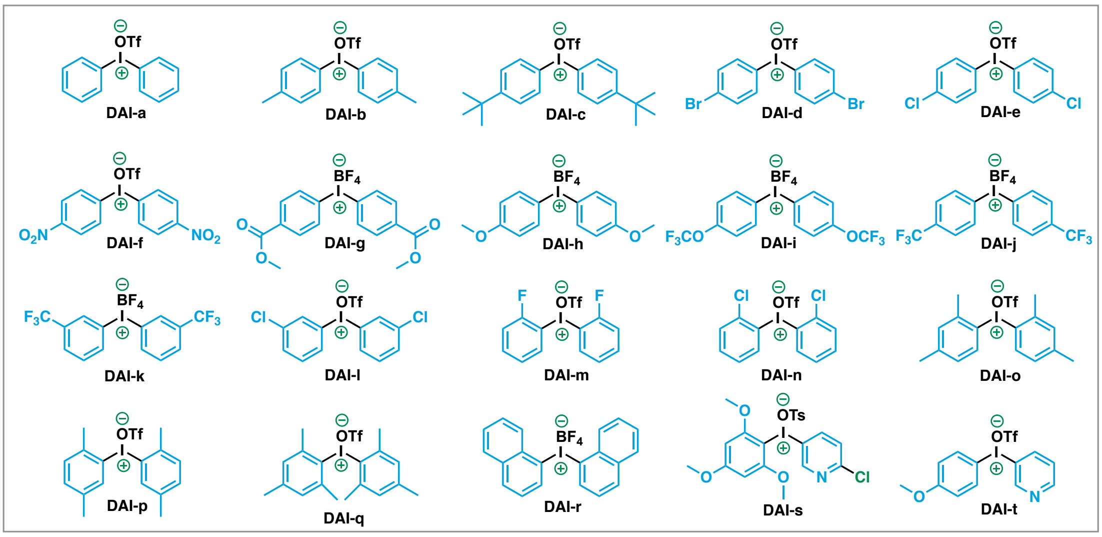

Figure S2. Structures of diaryliodonium salts **DAI-a–DAI-t**

General procedure (i) for the synthesis of diaryliodonium salts from arenes and iodine:[1]

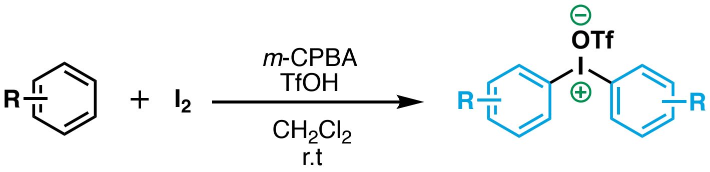

m-CPBA (85% active oxidant, 15 mmol) and iodine (5 mmol) were dissolved in DCM (50mL) in a sealed tube, which resulted in a dark purple solution that was cooled to 0 ˚C. Arene was added followed by dropwise addition of TfOH (10 mmol), resulting in a slight heat increase and change of color to a yellow transparent solution. The solution was stirred at the indicated temperature and time and concentrated in vacuo. MTBE (2–3mL) was added and the mixture was stirred at rt for 5 min to precipitate out an off-white solid. The flask was stored in the freezer for 30 min, then the solid was filtered off, washed with cold MTBE and dried under vacuum to give diaryliodonium salt.

General procedure (ii) for the one-pot synthesis of diaryliodonium salts from iodides and arenen:[1]

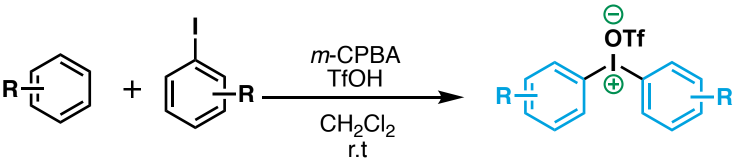

m-CPBA (85% active oxidant, 5 mmol) and aryl iodide 1 (5 mmol) were dissolved in CH_2Cl_2 (20 mL) in a sealed tube. Then arene (5 mmol) was added, and the solution was cooled to 0 ˚C followed by dropwise addition of TfOH (10 mmol), resulting in a slight heat increase. The solution was stirred at the indicated temperature and time and

concentrated in vacuo. MTBE (2–3mL) was added and the mixture was stirred at rt for 5 min to precipitate out an off-white solid. The flask was stored in the freezer for 30 min, then the solid was filtered off, washed with cold MTBE and dried under vacuum to give diaryliodonium salt.

General procedure (iii) for the synthesis of diaryliodonium salts from iodides and arylboronic acid:[2]

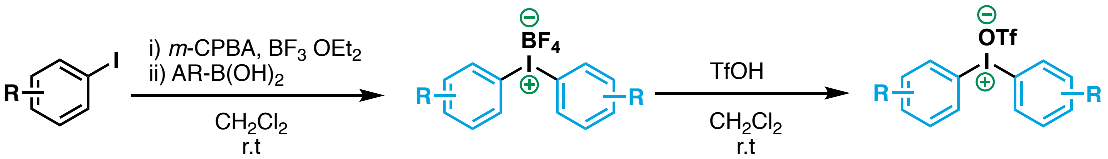

m-CPBA (85% active oxidant, 5 mmol) was dissolved in CH_2Cl_2 (20 mL). To the solution was added aryl iodide (5 mmol) followed by $BF_3{\cdot}OEt_2$ (10 mmol) at room temperature. The resulting yellow solution was stirred at rt for 1h and then cooled to 0 °C, and aryl boronic acid (5 mmol) was added. After 30 min of stirring at rt, the solution was cooled to 0 °C, followed by dropwise addition of TfOH (10 mmol), resulting in a slight heat increase, the solution was stirred at the indicated temperature and time and concentrated in vacuo. Then the crude reaction mixture was applied on a silica plug (10 g) and eluted with CH_2Cl_2 (10 mL) to remove unreacted aryl iodide and m-CPBA, followed by MeOH/ CH_2Cl_2 (5 : 95), to elute the product. The latter solution was concentrated in vacuo. MTBE (2–3mL) was added and the mixture was stirred at rt for 5 min to precipitate out an off-white solid. The flask was stored in the freezer for 30 min, then the solid was filtered off, washed with cold MTBE and dried under vacuum to give diaryliodonium salt.

General procedure (iv) for the synthesis of diaryliodonium salts from iodides and arenes:[3]

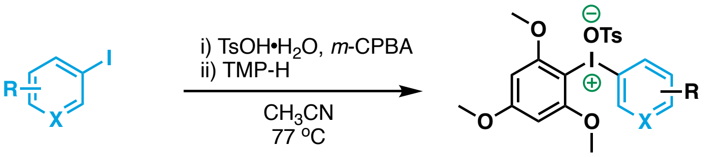

Aryl iodide (1.00 mmol) and acetonitrile (1 ml) were added to a 10 ml round-bottom flask and equipped with a magnetic stir bar. $TsOH{\cdot}H_2O$ (1 mmol) was added in one portion at room temperature, followed by *m*-CPBA (85% active oxidant, 1 mmol). The flask was lowered into a pre-heated heating block set at 77 °C, and stirred for 30 minutes. 1,3,5-trimethoxybenzene (TMP-H) (1 mmol) was then added and the resulting mixture was stirred for 5 minutes at 77 °C. The flask was cooled to ambient temperature before acetonitrile was removed under reduced pressure. Then the resulting crude oil was applied on a silica plug (10 g) and eluted with CH_2Cl_2 (10 mL) to remove unreacted aryl iodide and *m*-CPBA, followed by MeOH/CH_2Cl_2 (1:100–1:10) to elute the product.

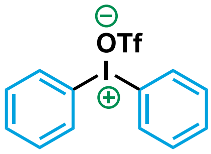

DAI-a

DAI-a was obtained from the commercial source.

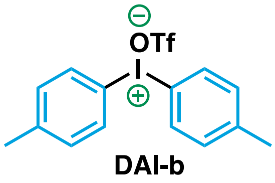

Synthesized according to ***General procedure (ii)***, in 62% yield. ^{1}H NMR (500 MHz, DMSO-d_6) δ 8.18 – 7.88 (m, 4H), 7.35– 7.30 (m, 4H), 2.34 (s, 6H). The ^{1}H NMR matches that previously reported.[1]

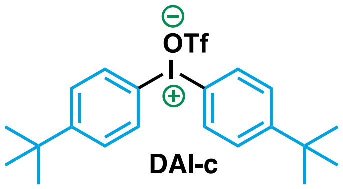

Synthesized according to ***General procedure (i),*** in 86% yield. ^{1}H NMR (500 MHz, Chloroform-*d*) δ 7.95 – 7.86 (m, 4H), 7.53 – 7.45 (m, 4H), 1.33 (d, J = 3.5 Hz, 18H). The ^{1}H NMR matches that previously reported.[4]

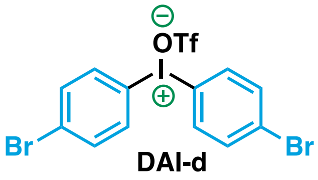

Synthesized according to ***General procedure (ii)***, in 72% yield. ^{1}H NMR (500 MHz, DMSO-d_6) δ 8.22 – 8.10 (m, 4H), 7.82 – 7.68 (m, 4H). The ^{1}H NMR matches that previously reported.[4]

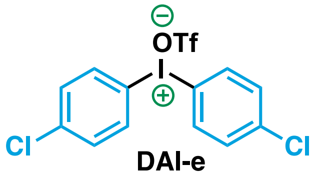

Synthesized according to ***General procedure (ii)***, in 72% yield. ^{1}H NMR (500 MHz, DMSO-d_6) δ 8.26 (d, J = 8.7 Hz, 4H), 7.64 (d, J = 8.8 Hz, 4H). The ^{1}H NMR matches that previously reported.[1]

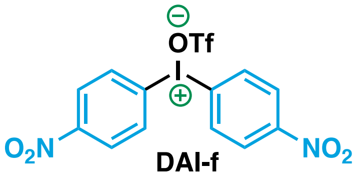

DAI-f

Synthesized according to ***General procedure (iii)***, in 21% yield. ^{1}H NMR (500 MHz, DMSO-d_6) δ 8.51 (d, J = 9.0 Hz, 4H), 8.32 – 8.26 (m, 4H). Exact Mass: 371.9607. Observed mass 371.9636. ^{13}C NMR (126 MHz, DMSO) δ 150.07, 137.27, 126.92, 123.49. HRMS (ESI) calcd for $[M+H]^+$ $[C_{12}H_9IN_2O_4]^+$ 371.9596, observed mass 371.9636.

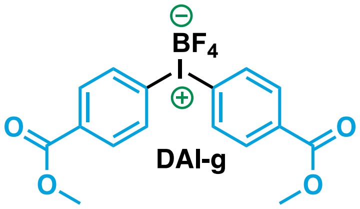

DAI-g

Synthesized according to ***General procedure (iii)***, in 36% yield. ^{1}H NMR (500 MHz, DMSO-d_6) δ 8.42 – 8.37 (m, 4H), 8.07 – 8.01 (m, 4H), 3.87 (s, 6H). The ^{1}H NMR matches that previously reported.[5]

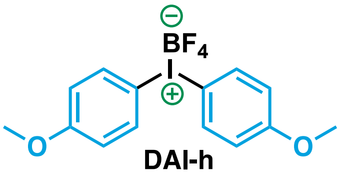

DAI-h

Synthesized according to ***General procedure (iii)***, in 33% yield. ^{1}H NMR (500 MHz, DMSO-d_6) δ 8.16 – 8.08 (m, 4H), 7.10 – 7.02 (m, 4H), 3.79 (s, 6H). The ^{1}H NMR matches that previously reported.[2]

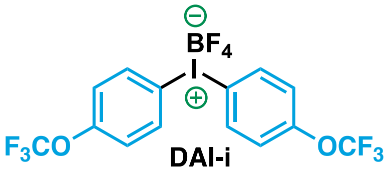

DAI-i

Synthesized according to ***General procedure (iii)***, in 42% yield. ^{1}H NMR (500 MHz, DMSO-d_6) δ 8.44 – 8.37 (m, 4H), 7.62 – 7.55 (m, 4H). The ^{1}H NMR matches that previously reported.[6]

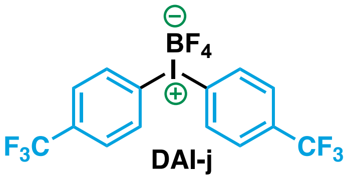

DAI-j

Synthesized according to ***General procedure (iii)***, in 56% yield. ^{1}H NMR (500 MHz, DMSO-d_6) δ 8.58 – 8.40 (m, 4H), 7.95 (d, *J* = 8.4 Hz, 4H). The ^{1}H NMR matches that previously reported.[2]

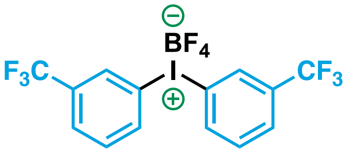

DAI-k

Synthesized according to ***General procedure (iii)***, in 37% yield. ^{1}H NMR (500 MHz, DMSO-d_6) δ 8.82 (d, *J* = 1.9 Hz, 2H), 8.65 – 8.56 (m, 2H), 8.12 – 8.03 (m, 2H), 7.79 (t, *J* = 8.0 Hz, 2H). The ^{1}H NMR matches that previously reported.[2]

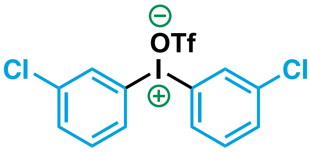

DAI-l

Synthesized according to ***General procedure (iii)***, in 74% yield. ^{1}H NMR (500 MHz, DMSO-d_6) δ 8.50 (t, *J* = 1.9 Hz, 2H), 8.25 (ddd, *J* = 8.1, 1.7, 0.9 Hz, 2H), 7.76 (ddd, *J* = 8.1, 2.1, 0.9 Hz, 2H), 7.58 (t, *J* = 8.1 Hz, 2H). The ^{1}H NMR matches that previously reported.[7]

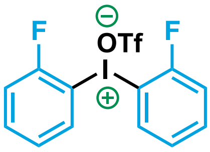

DAI-m

Synthesized according to ***General procedure (iii)***, in 76% yield. ^{1}H NMR (500 MHz, DMSO-d_6) δ 8.40 (td, *J* = 6.2, 3.0 Hz, 2H), 7.78 – 7.69 (m, 2H), 7.59 (ddd, *J* = 8.5, 7.2, 1.4 Hz, 2H), 7.38 (ddd, *J* = 8.7, 7.4, 1.4 Hz, 2H). The ^{1}H NMR matches that previously reported.[2]

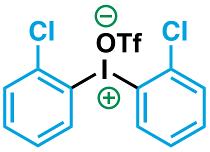

DAI-n

Synthesized according to ***General procedure (iii)***, in 81% yield. ^{1}H NMR (500 MHz, DMSO-d_6) δ 8.52 (dd, *J* = 8.1, 1.5 Hz, 2H), 7.84 (dd, *J* = 8.1, 1.5 Hz, 2H), 7.71 (td, *J* =

7.7, 1.5 Hz, 2H), 7.49 (td, J = 7.7, 1.5 Hz, 2H). ^{13}C NMR (126 MHz, DMSO) δ 139.40, 136.44, 135.25, 130.95, 130.68, 120.00. HRMS (ESI) calcd for $[M+H]^+$ $[C_{12}H_9Cl_2I]^+$ 349.9126. Observed mass 349.9130.

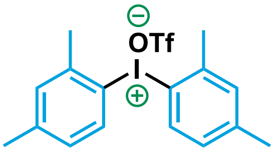

DAI-o

Synthesized according to ***General procedure (i)***, in 82% yield. ^{1}H NMR (500 MHz, DMSO-d_6) δ 8.14 (d, J = 8.2 Hz, 2H), 7.36 (d, J = 2.2 Hz, 2H), 7.10 (dd, J = 8.3, 2.2 Hz, 2H), 2.54 (s, 6H), 2.30 (s, 6H). The ^{1}H NMR matches that previously reported.[5]

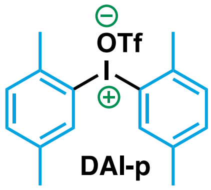

DAI-p

Synthesized according to ***General procedure (i)***, in 90% yield. ^{1}H NMR (500 MHz, Chloroform-d) δ 7.75 (s, 2H), 7.36 (s, 4H), 2.59 (s, 6H), 2.37 (s, 6H). The ^{1}H NMR matches that previously reported.[4]

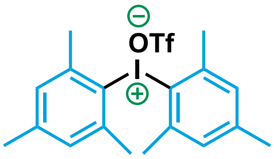

DAI-q

Synthesized according to ***General procedure (ii)***, in 90% yield. ^{1}H NMR (500 MHz, DMSO-d_6) δ 7.19 (s, 4H), 2.46 (s, 12H), 2.29 (s, 6H). The ^{1}H NMR matches that previously reported.[4]

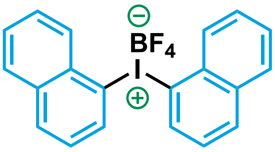

DAI-r

Synthesized according to ***General procedure (iii)***, in 42% yield. ^{1}H NMR (500 MHz, DMSO-d_6) δ 8.88 (dd, J = 7.6, 1.1 Hz, 2H), 8.49 (d, J = 8.5 Hz, 2H), 8.21 (d, J = 8.1 Hz, 2H), 8.07 – 7.98 (m, 2H), 7.81 (ddd, J = 8.4, 6.9, 1.3 Hz, 2H), 7.68 (ddd, J = 8.1, 6.9, 1.0 Hz, 2H), 7.57 (t, J = 7.8 Hz, 2H). The ^{1}H NMR matches that previously

reported.[4]

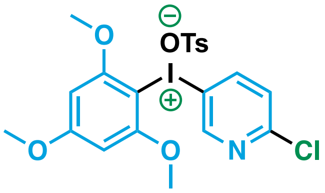

DAI-s

Synthesized according to ***General procedure (iv)***, in 79% yield. ^{1}H NMR (500 MHz, DMSO-d_6) δ 8.86 (d, J = 2.5 Hz, 1H), 8.36 (dd, J = 8.5, 2.4 Hz, 1H), 7.67 – 7.58 (m, 1H), 7.49 – 7.47 (m, 2H), 7.12 (d, J = 7.9 Hz, 2H), 6.46 (s, 2H), 4.10 – 3.87 (m, 6H), 3.87 (s, 3H), 2.29 (s, 3H). The ^{1}H NMR matches that previously reported.[8]

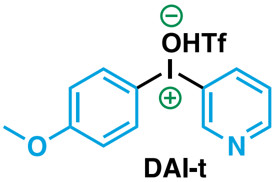

DAI-t

Synthesized according to ***General procedure (ii)***, in 61% yield. ^{1}H NMR (500 MHz, DMSO-d_6) δ 9.31 – 9.24 (m, 1H), 8.82 (dd, J = 4.7, 1.4 Hz, 1H), 8.63 (ddd, J = 8.2, 2.3, 1.5 Hz, 1H), 8.24 – 8.18 (m, 2H), 7.58 (ddd, J = 8.2, 4.7, 0.7 Hz, 1H), 7.13 – 7.06 (m, 2H), 3.80 (s, 3H). The ^{1}H NMR matches that previously reported.[9]

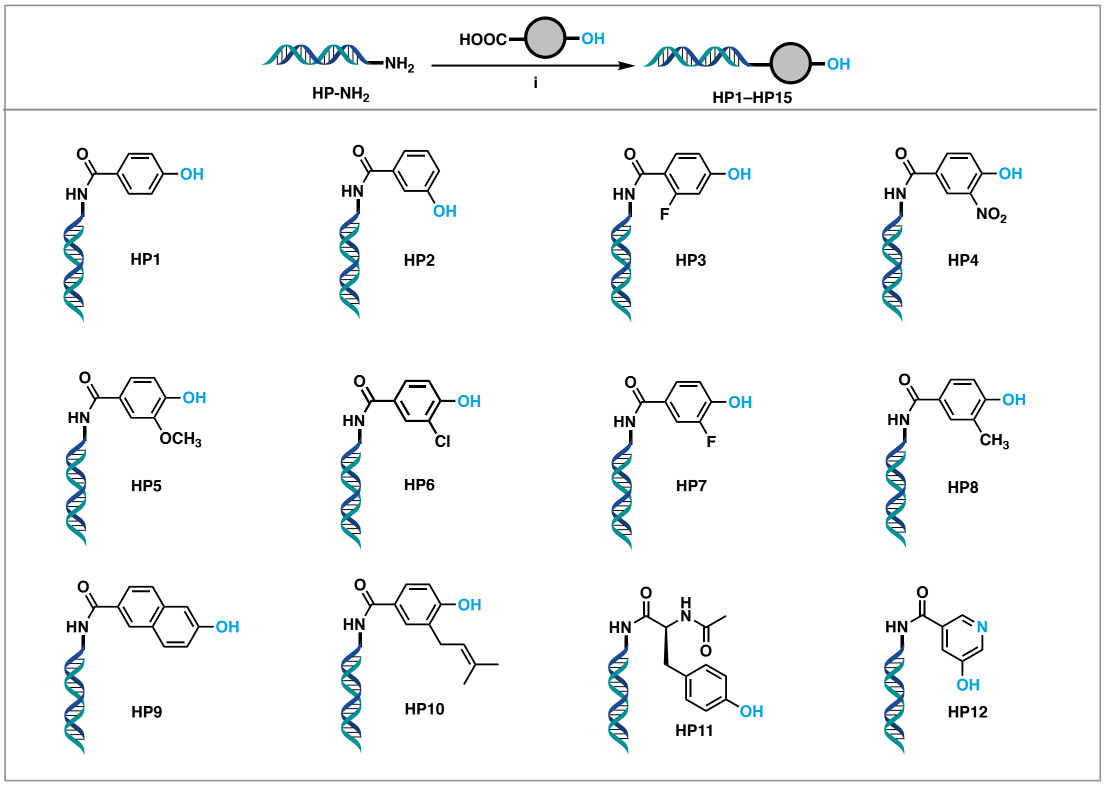

Scheme S1. Synthesis of DNA-conjugated phenols **HP1**–**HP9 and HP11**–**HP12**. i) To a solution of DNA headpiece (**HP-NH₂**) (600 *μ*L, 300 nmol) in borate buffer (250 mM, pH = 9.4) was added a mixture of DMA solution of HATU (60 *μ*L, 200 mM), DIPEA (60 *μ*L, 200 mM) and acids (60 *μ*L, 200 mM). The resultant mixture was vortexed and stood at 25 °C for 8 hours. 5M NaCl (78 *μ*L) and cold ethanol (2.34 mL) were sequentially added, and the resultant mixture was stored at −80 °C for 30 min. The mixture was centrifuged at 4 °C for 30 min at 12000 rpm to remove the supernatant. The resulting pellet was re-dissolved in *dd*H_2O (300 *μ*L), which was used in following reaction without further purification.

Synthesis of DNA-conjugated phenols **HP10**. Amino acid (5 *μ*L, 200 mM in DMSO) was incubated with EDC (2.5 μL, 400 mM DMSO) and *s*NHS sodium salt (6.6 *μ*L, 133 mM DMSO/H_2O (2:1) for 25 min at 30℃. The solution of **HP-NH₂** (20 *μ*L, 20 nmol) in borate buffer (250 mM, pH = 9.4) was then added to the mixture and the reaction was let to proceed for 3 hours at 30 °C. The reaction mixture was next added 5 M NaCl solution (10% by volume) and cold ethanol (2.5 times by volume, ethanol stored at -20°C) were added, vortexed, and incubated at -80°C for at least 30 minutes. The sample was centrifuged for 30 minutes at 4°C in a microcentrifuge at 12,000 rpm to remove the supernatant. The resulting pellet was re-dissolved in *dd*H_2O (20 *μ*L), which was used in following reaction without further purification.

Table S1. Screening of the on-DNA *O*-arylation reaction parameters

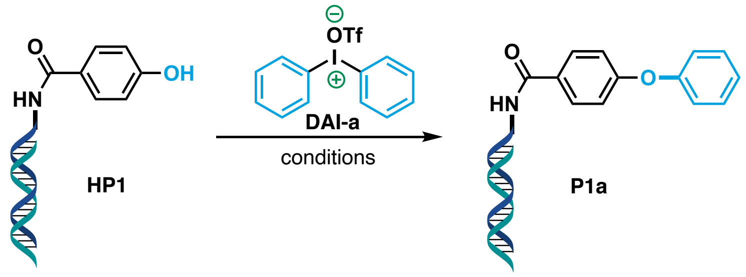

Entry	DAI-a (equiv)	pH of BBS	Solvent	Temp.	Time (h)	Yield (%)[b]
**1**[a]	200	9.4	DMA-BBS (2:3)	80	2.5	87
**2**	400	NaOH (800eq)	DMA-ddH_2O (1:9)	70	2.5	37
**3**	400	NaOH (800eq)	DMA-ddH_2O (1:9)	80	2.5	65
**4**	400	NaOH (800eq)	DMA-ddH_2O (1:9)	90	2.5	46
**5**	800	8	DMA- BBS (1:9)	80	2.5	14
**6**	800	9	DMA-BBS (1:9)	80	2.5	48
**7**	800	9.4	DMA-BBS (1:9)	80	2.5	73
**8**	800	9.4	DMA-BBS (1:4)	80	2.5	80
**9**	600	9.4	DMA-BBS (2:3)	80	2.5	76
**10**	400	9.4	DMA-BBS (2:3)	80	2.5	78
**11**	200	9.4	DMA-BBS (2:3)	70	2.5	59
**12**	200	9.4	DMA-BBS (2:3)	80	2	80

[a] Reactions were performed at 80°C with **HP1** (5 nM, 1 mM in *dd*H_2O) and **DAI-a** (200 eq, 500 mM in DMA) in total volume of 50 μL aqueous solutions ($V_{DMA}/V_{borate\ buffer\ (pH\ 9.4)}$ = 2 : 3), 2.5h. [b] The conversion yield was determined by LC-MS. BBS: borate buffer solution.

General Procedures (v) for the on-DNA O-arylation reactions

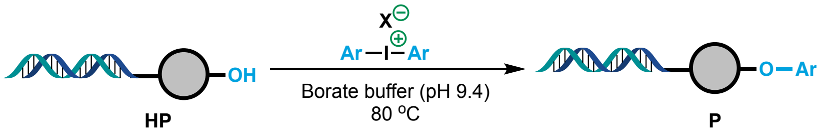

To **HP** (5 μL, 10 nmol, 1 mM in *dd*H_2O) was added 25 μL of borate buffer (pH 9.4, 250 mM) and 200 equiv. of **DAI** (20 μL, 50 mM in DMA). The resulting mixture was vortexed and stood at 80 °C for 2.5 h. The reaction mixture was next added 5 M NaCl solution (10% by volume) and cold ethanol (2.5 times by volume, ethanol stored at -20°C) were added, vortexed, and incubated at -80°C for at least 30 minutes. The sample was centrifuged for 30 minutes at 4°C in a microcentrifuge at 12,000 rpm to remove the supernatant. The resulting pellet (precipitate) was re-dissolved in *dd*H_2O (300 μL) for LC-MS detection.

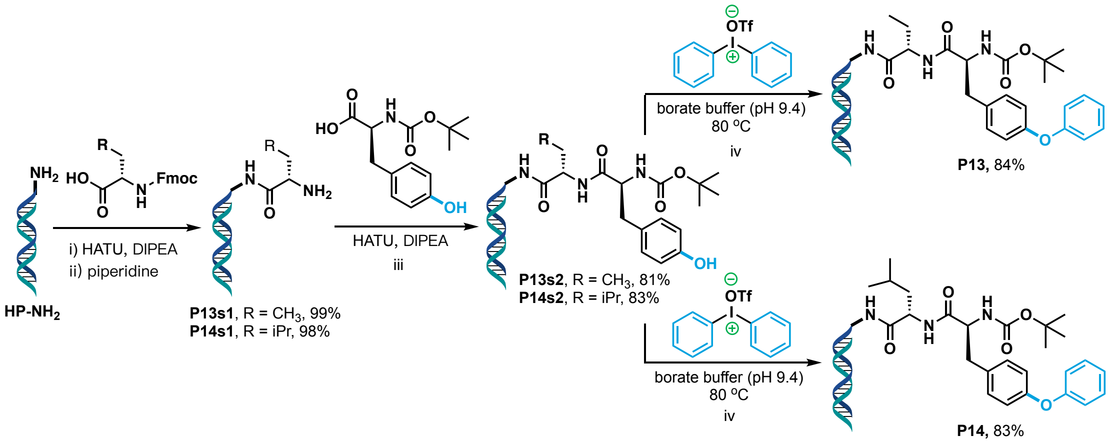

Scheme S2. Syntheis of **P13** and **P14**

Step i): To a solution of **HP-NH₂** (10 μL, 10 nmol) in borate buffer (250 mM, pH = 9.4) was added a mixture of DMA solution of HATU (2 μL, 200 mM), DIPEA (2 μL, 200 mM) and acids (2 μL, 200 mM). The resultant mixture was vortexed and stood at 25 °C for 8 hours. The reaction mixture was next added 5 M NaCl solution (10% by volume) and cold ethanol (2.5 times by volume, ethanol stored at -20°C) were added, vortexed, and incubated at -80°C for at least 30 minutes. The sample was centrifuged for 30 minutes at 4 °C in a microcentrifuge at 12,000 rpm to remove the supernatant. The resulting pellet was re-dissolved in *dd*H_2O (10 μL), which was used in following reaction without further purification.

Step ii): To a solution of Fmoc protected DNA material (10 μL, 10 nmol) in *dd*H_2O, was added 5 μL of piperidine. The resultant mixture was vortexed and stood at 25 °C for 2 hours. The reaction mixture was next added 5 M NaCl solution (10% by volume) and cold ethanol (2.5 times by volume, ethanol stored at -20 °C) were added, vortexed, and incubated at -80 °C for at least 30 minutes. The sample was centrifuged for 30 minutes at 4°C in a microcentrifuge at 12,000 rpm to remove the supernatant. The resulting pellet was re-dissolved in *dd*H_2O (10 μL), which was used in following reaction without further purification.

Step iii): To a solution of **P13s1/P14s1** (5 nmol) in 10 μL borate buffer (250 mM, pH = 9.4) was added HATU (2 μL, 400 mM in DMA), DIPEA (2 μL, 200 mM) and acids (2 μL, 200 mM). The resultant mixture was vortexed and stood at 25 °C for 8 hours. After that 5 M NaCl solution (10% by volume) and cold ethanol (2.5 times by volume, ethanol stored at -20°C) were added, vortexed, and incubated at -80 °C for at least 30 minutes. The sample was centrifuged for 30 minutes at 4 °C in a microcentrifuge at 12,000 rpm to remove the supernatant. The resulting pellet was re-dissolved in *dd*H_2O (5 μL), which was used in following reaction without further purification.

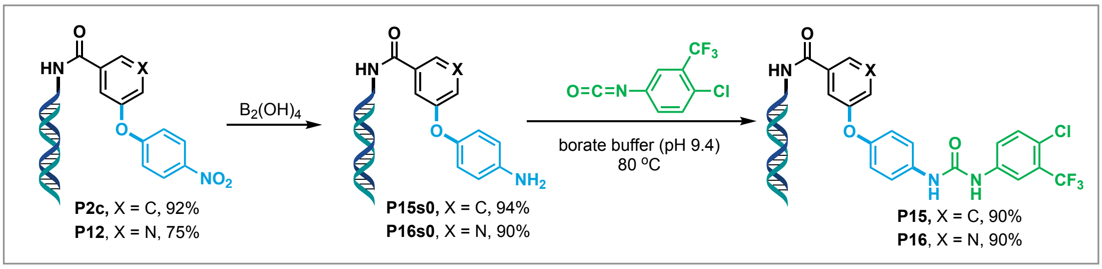

Scheme S3. Synthesis of **P15** and **P16**

Step i): To **P2c** or **P12** (5 μL, 5 nmol, 1 mM in *dd*H_2O) was added $B_2(OH)_4$ (5 μL, 750 nmol, 150 mM in *dd*H_2O), NaOH (5 μL, 2500 nmol, 500 mM in ddH_2O), 2.5 μL *dd*H_2O and 7.5 μL EtOH (30% by volume). The resulting mixture was vortexed and stood at rt for 2 hours.[10] The reaction mixture was next added 5 M NaCl solution (10% by volume) and cold ethanol (2.5 times by volume, ethanol stored at -20°C), vortexed, and incubated at -80°C for at least 30 minutes. The sample was centrifuged for 30 minutes at 4°C in a microcentrifuge at 12,000 rpm to remove the supernatant. The resulting pellet (precipitate) was re-dissolved in *dd*H_2O (300 μL) for LC-MS detection.

Step ii): To **P15s0**or **P16s0** (10 μL, 10 nmol, 1 mM in pH 9.4 borate buffer) was added 1-chloro-4-isocyanato-2-(trifluoromethyl)benzene (1 μL, 500 nmol, 500 mM in MeCN).[11] The resulting mixture was vortexed and stood at 80°C for 16 hours. The reaction mixture was next added 5 M NaCl solution (10% by volume) and cold ethanol (2.5 times by volume, ethanol stored at -20°C), vortexed, and incubated at -80°C for at least 30 minutes. The sample was centrifuged for 30 minutes at 4°C in a microcentrifuge at 12,000 rpm to remove the supernatant. The resulting pellet (precipitate) was re-dissolved in *dd*H_2O (10 μL) for LC-MS detection.

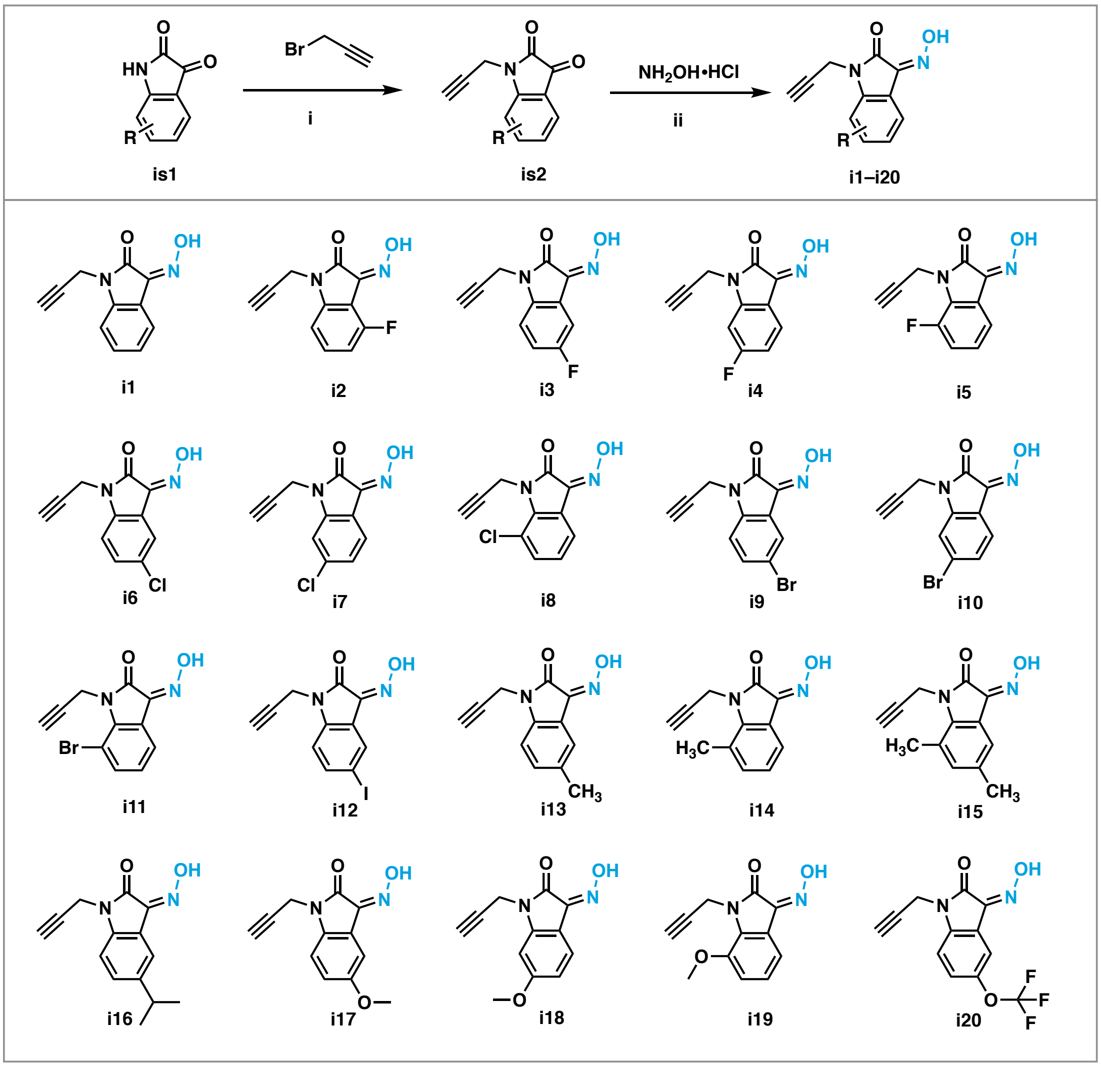

Scheme S4. Synthesis of isatin-oximes **i1–i20**.

Step i): To a solution of **is1** (2 mmol, 1 equiv.) in 10 mL CH_2Cl_2 was added K_2CO_3 (6 mmol, 3 equiv), followed by dropwise addition of propargyl bromide (6 mmol, 3 equiv). The resulting reaction mixture was stirring at rt for 3 hours.[12] Then the solid was filtered off and the organic was concentrated in vacuo. Then the crude reaction mixture was applied on a silica plug (10 g) and eluted with CH_2Cl_2 (10 mL) followed by MeOH/CH_2Cl_2 (1:100–1:10) to elute the product.

Step ii): **is2** (1mmol, 1 equiv.) was dissolved in ethanol (5 ml). To this solution was added hydroxylamine hydrochloride (1.2 mmol, 1.2 equiv.) and KOAc (2 mmol, 2 equiv.) in 3 ml of water.[13] The resulting reaction mixture was stirred at 50° for 2 hours, then cooled to rt. The yellow precipitate was removed by filtration and dried under high vacuum to produce the isatin oxime (**i1–i20**).

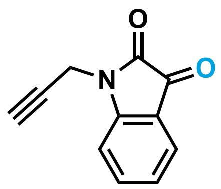

is2-1

91% yield. ^{1}H NMR (500 MHz, DMSO-d_6) δ 7.73 (td, J = 7.8, 1.4 Hz, 1H), 7.60 (dd, J = 7.5, 1.3 Hz, 1H), 7.30 – 7.15 (m, 2H), 4.56 (d, J = 2.5 Hz, 2H), 3.36 (d, J = 2.5 Hz, 1H). The ^{1}H NMR matches that previously reported.[14]

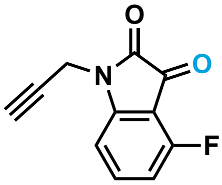

is2-2

68% yield.^{1}H NMR (500 MHz, DMSO-d_6) δ 7.77 (td, J = 8.3, 5.7 Hz, 1H), 7.08 (d, J = 7.9 Hz, 1H), 6.99 (t, J = 8.8 Hz, 1H), 4.57 (d, J = 2.6 Hz, 2H), 3.37 (t, J = 2.5 Hz, 1H). ^{13}C NMR (126 MHz, DMSO) δ 178.64, 159.10, 157.43, 150.42, 140.93, 111.66, 107.95, 106.19, 77.63, 75.62, 29.95. HRMS (ESI) calcd for $[M+H]^+$ $[C_{11}H_7FNO_2]^+$ 204.0455, observed mass 204.0496.

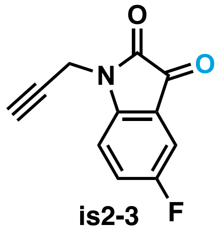

is2-3

40% yield. ^{1}H NMR (500 MHz, Chloroform-d) δ 7.37 (td, J = 8.5, 7.5, 2.4 Hz, 2H), 7.14 – 7.08 (m, 1H), 4.54 (d, J = 2.6 Hz, 2H), 2.33 (t, J = 2.5 Hz, 1H). The ^{1}H NMR matches that previously reported.[15]

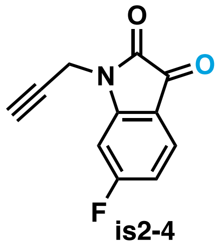

is2-4

36% yield. ^{1}H NMR (500 MHz, DMSO-d_6) δ 7.71 (dd, J = 8.3, 5.7 Hz, 1H), 7.27 – 7.17 (m, 1H), 6.99 (ddd, J = 10.3, 8.3, 2.2 Hz, 1H), 4.57 (d, J = 2.6 Hz, 2H), 3.37 (t, J = 2.5 Hz, 1H). ^{13}C NMR (126 MHz, DMSO-d_6) δ 181.00, 169.41, 167.37, 158.14, 152.66, 128.15, 115.05, 110.62, 100.44, 77.60, 75.61, 29.76. HRMS (ESI) calcd for $[M+H]^+$ $[C_{11}H_7FNO_2]^+$ 204.0455, observed mass 204.0437.

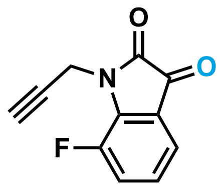

is2-5

70% yield. ^{1}H NMR (500 MHz, DMSO-d_6) δ 7.64 (ddd, J = 11.6, 8.4, 1.1 Hz, 1H), 7.48 (dd, J = 7.3, 1.1 Hz, 1H), 7.21 (ddd, J = 8.3, 7.3, 4.0 Hz, 1H), 4.57 (dd, J = 2.6, 1.1 Hz, 2H), 3.34 (d, J = 2.4 Hz, 1H). ^{13}C NMR (126 MHz, DMSO-d_6) δ 181.79, 157.84, 148.83, 146.87, 135.81, 126.36, 125.28, 121.35, 78.42, 75.12, 31.73. HRMS (ESI) calcd for $[M+H]^+$ $[C_{11}H_7FNO_2]^+$ 204.0455, observed mass 204.0496.

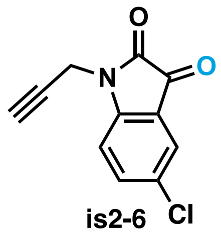

is2-6

83% yield. ^{1}H NMR (500 MHz, DMSO-d_6) δ 7.78 (dd, J = 8.5, 2.3 Hz, 1H), 7.66 (d, J = 2.3 Hz, 1H), 7.27 (d, J = 8.4 Hz, 1H), 4.57 (d, J = 2.5 Hz, 2H), 3.37 (s, 1H). The ^{1}H NMR matches that previously reported.[14]

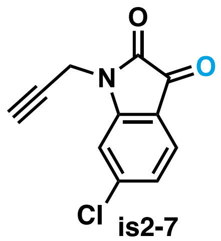

is2-7

85% yield. ^{1}H NMR (500 MHz, DMSO-d_6) δ 7.62 (d, J = 8.0 Hz, 1H), 7.39 (d, J = 1.8 Hz, 1H), 7.24 (dd, J = 8.0, 1.8 Hz, 1H), 4.58 (d, J = 2.6 Hz, 2H), 3.37 (t, J = 2.6 Hz, 1H). ^{13}C NMR (126 MHz, DMSO) δ 181.57, 157.92, 151.00, 142.68, 126.51, 123.92, 117.09, 111.99, 77.61, 75.63, 29.68. HRMS (ESI) calcd for $[M+H]^+$ $[C_{11}H_7ClNO_2]^+$ 220.0160, observed mass 220.0169.

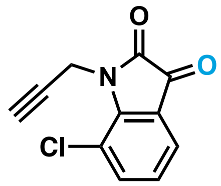

is2-8

81% yield. ^{1}H NMR (500 MHz, Chloroform-d) δ 7.59 (d, J = 7.7 Hz, 2H), 7.13 (t, J = 7.8 Hz, 1H), 4.91 (d, J = 2.5 Hz, 2H), 2.31 (t, J = 2.5 Hz, 1H). The ^{1}H NMR matches that previously reported.[15]

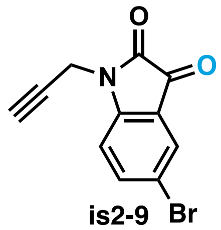

is2-9

85% yield. ^{1}H NMR (500 MHz, Chloroform-*d*) δ 7.76 (d, *J* = 10.4 Hz, 2H), 7.06 (d, *J* = 8.3 Hz, 1H), 4.54 (d, *J* = 2.6 Hz, 2H), 2.34 (d, *J* = 2.6 Hz, 1H). The ^{1}H NMR matches that previously reported.[15]

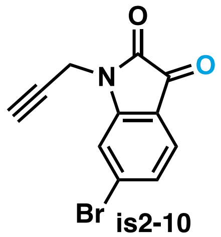

is2-10

89% yield. ^{1}H NMR (500 MHz, DMSO-d_6) δ 7.52 (dd, *J* = 4.8, 3.1 Hz, 2H), 7.38 (dd, *J* = 8.0, 1.6 Hz, 1H), 4.58 (d, *J* = 2.6 Hz, 2H), 3.37 (d, *J* = 2.6 Hz, 1H). ^{13}C NMR (126 MHz, DMSO) δ 181.82, 157.82, 150.77, 132.00, 126.91, 126.46, 117.36, 114.75, 77.60, 75.63, 29.66. HRMS (ESI) calcd for $[M+H]^+$ $[C_{11}H_7BrNO_2]^+$ 263.9655, observed mass 263.9641.

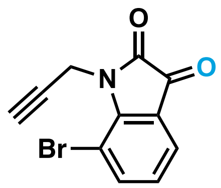

is2-11

86% yield. ^{1}H NMR (500 MHz, DMSO-d_6) δ 7.87 (dd, *J* = 8.1, 1.3 Hz, 1H), 7.62 (dd, *J* = 7.3, 1.3 Hz, 1H), 7.12 (dd, *J* = 8.1, 7.2 Hz, 1H), 4.80 (d, *J* = 2.5 Hz, 2H), 3.37 (t, *J* = 2.4 Hz, 1H). ^{13}C NMR (126 MHz, DMSO) δ 181.79, 158.90, 146.68, 143.33, 125.87, 124.56, 121.84, 104.03, 79.29, 75.72, 31.66. HRMS (ESI) calcd for $[M+H]^+$ $[C_{11}H_7BrNO_2]^+$ 263.9655, observed mass 263.9674.

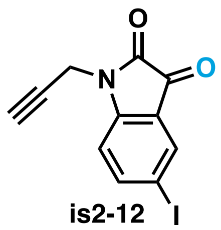

is2-12

87% yield. ^{1}H NMR (500 MHz, DMSO-d_6) δ 8.05 (dd, *J* = 8.2, 1.8 Hz, 1H), 7.85 (d, *J* = 1.8 Hz, 1H), 7.09 (d, *J* = 8.3 Hz, 1H), 4.55 (d, *J* = 2.6 Hz, 2H), 3.36 (t, *J* = 2.5 Hz, 1H). ^{13}C NMR (126 MHz, DMSO) δ 181.63, 157.14, 149.19, 146.02, 132.69, 120.19, 114.05, 87.11, 77.56, 75.65, 29.54. HRMS (ESI) calcd for $[M+H]^+$ $[C_{11}H_7INO_2]^+$ 311.9516. observed mass 311.9500.

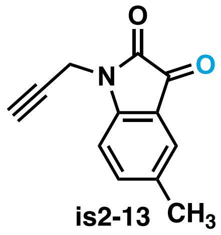

is2-13

90% yield. ^{1}H NMR (500 MHz, DMSO-d_6) δ 7.54 (dd, J = 8.2, 1.9 Hz, 1H), 7.43 (d, J = 1.8 Hz, 1H), 7.14 (d, J = 8.0 Hz, 1H), 4.53 (d, J = 2.4 Hz, 2H), 3.34 (t, J = 2.5 Hz, 1H), 2.31 (s, 3H). The ^{1}H NMR matches that previously reported.[16]

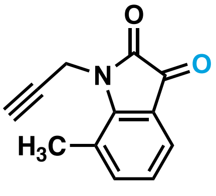

is2-14

89% yield. ^{1}H NMR (500 MHz, DMSO-d_6) δ 7.50 (d, J = 7.7 Hz, 1H), 7.45 (dd, J = 7.3, 1.4 Hz, 1H), 7.09 (t, J = 7.5 Hz, 1H), 4.68 (d, J = 2.5 Hz, 2H), 3.43 (t, J = 2.5 Hz, 1H), 2.64 (s, 3H). ^{13}C NMR (126 MHz, DMSO) δ 183.30, 158.96, 147.75, 142.37, 124.32, 123.14, 122.57, 119.16, 79.82, 75.76, 31.71, 18.04. HRMS (ESI) calcd for $[M+H]^+$ $[C_{12}H_{10}NO_2]^+$ 200.0706, observed mass 200.0707.

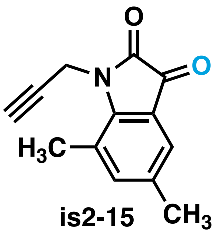

is2-15

88% yield. ^{1}H NMR (500 MHz, DMSO-d_6) δ 7.32 (d, J = 1.9 Hz, 1H), 7.26 (d, J = 1.9 Hz, 1H), 4.65 (d, J = 2.5 Hz, 2H), 3.42 (t, J = 2.4 Hz, 1H), 2.59 (s, 3H), 2.25 (s, 3H). ^{13}C NMR (126 MHz, DMSO) δ 183.51, 159.02, 145.60, 142.80, 133.68, 123.31, 122.38, 119.17, 79.78, 75.69, 31.66, 20.24, 17.90. HRMS (ESI) calcd for $[M+H]^+$ $[C_{13}H_{12}NO_2]^+$ 214.0863, observed mass 214.0814.

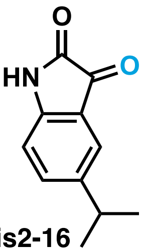

is2-16

31% yield. ^{1}H NMR (500 MHz, Chloroform-d) δ 7.53 (d, J = 1.9 Hz, 1H), 7.51 (dd, J = 8.1, 2.0 Hz, 1H), 7.05 (d, J = 8.0 Hz, 1H), 4.52 (d, J = 2.6 Hz, 2H), 2.92 (hept, J = 6.9 Hz, 1H), 2.30 (t, J = 2.5 Hz, 1H), 1.25 (d, J = 6.9 Hz, 6H). ^{13}C NMR (126 MHz, $CDCl_3$) δ 182.91, 157.43, 147.72, 145.31, 136.74, 123.32, 117.79, 110.95, 75.87, 73.22,

33.58, 29.45, 23.81. HRMS (ESI) calcd for $[M+H]^+$ $[C_{14}H_{14}NO_2]^+$ 228.1019, observed mass 228.1024.

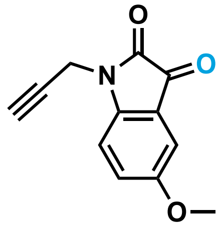

is2-17

66% yield. ^{1}H NMR (500 MHz, DMSO-d_6) δ 7.32 (dt, J = 8.6, 2.0 Hz, 1H), 7.18 (dq, J = 5.5, 1.5 Hz, 2H), 4.53 (t, J = 2.0 Hz, 2H), 3.79 (d, J = 1.5 Hz, 3H), 3.34 (q, J = 2.2 Hz, 1H). The ^{1}H NMR matches that previously reported.[17]

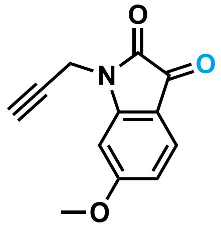

is2-18

72% yield. ^{1}H NMR (500 MHz, DMSO-d_6) δ 7.58 (d, J = 8.4 Hz, 1H), 6.83 (d, J = 2.1 Hz, 1H), 6.70 (dd, J = 8.5, 2.2 Hz, 1H), 4.56 (d, J = 2.5 Hz, 2H), 3.93 (s, 3H), 3.34 (t, J = 2.5 Hz, 1H). ^{13}C NMR (126 MHz, DMSO) δ 180.37, 168.26, 159.01, 152.68, 127.84, 111.42, 109.18, 98.43, 77.98, 75.35, 56.91, 29.50. HRMS (ESI) calcd for $[M+H]^+$ $[C_{12}H_{10}NO_3]^+$ 216.0655, observed mass 216.0619.

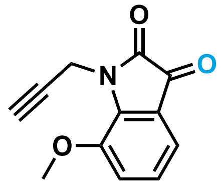

is2-19

80% yield. ^{1}H NMR (500 MHz, DMSO-d_6) δ 7.47 (dd, J = 8.2, 1.2 Hz, 1H), 7.25 – 7.11 (m, 2H), 4.62 (d, J = 2.5 Hz, 2H), 3.91 (s, 3H), 3.24 (t, J = 2.4 Hz, 1H). ^{13}C NMR (126 MHz, DMSO) δ 183.22, 158.45, 146.27, 137.70, 125.21, 123.50, 119.59, 117.33, 79.35, 74.40, 57.33, 32.36. HRMS (ESI) calcd for $[M+H]^+$ $[C_{12}H_{10}NO_3]^+$ 216.0655, observed mass 216.0619.

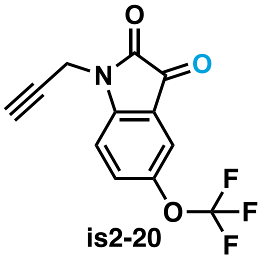

is2-20

75% yield. ^{1}H NMR (500 MHz, DMSO-d_6) δ 7.77 (dd, J = 8.7, 2.6 Hz, 1H), 7.64 (d, J = 2.5 Hz, 1H), 7.34 (d, J = 8.6 Hz, 1H), 4.58 (d, J = 2.5 Hz, 2H), 3.38 (t, J = 2.5 Hz, 1H). ^{13}C NMR (126 MHz, DMSO-d_6) δ 181.81, 173.27, 157.84, 148.58, 144.55, 139.82, 132.54, 131.01, 123.63, 121.59, 119.25, 118.23, 117.26, 113.00, 111.06, 91.42, 77.52, 75.69, 75.24, 29.62, 29.07. HRMS (ESI) calcd for [M+H]+ $[C_{12}H_7F_3NO_3]^+$ 270.0373, observed mass 270.0391.

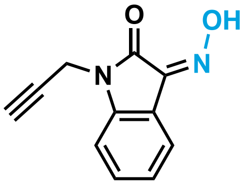

i1

91% yield. ^{1}H NMR (500 MHz, DMSO-d_6) δ 13.57 (s, 1H), 8.03 (dd, J = 7.6, 1.3 Hz, 1H), 7.49 (td, J = 7.8, 1.3 Hz, 1H), 7.24 – 7.08 (m, 2H), 4.58 (d, J = 2.6 Hz, 2H), 3.29 (t, J = 2.5 Hz, 1H). ^{13}C NMR (126 MHz, DMSO-d_6) δ 162.76, 143.67, 142.29, 132.51, 127.40, 123.56, 115.76, 110.14, 78.34, 74.94, 29.10. HRMS (ESI) calcd for $[M+H]^+$ $[C_{11}H_9N_2O_2]^+$ 201.0659, observed mass 201.0617.

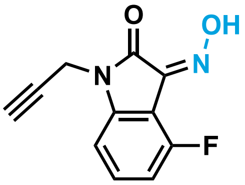

i2

60% yield. ^{1}H NMR (500 MHz, DMSO-d_6) δ 13.83 – 13.23 (m, 1H), 7.51 (dtd, J = 40.9, 8.1, 5.2 Hz, 1H), 7.05 – 6.90 (m, 2H), 4.58 (dd, J = 15.8, 2.5 Hz, 2H), 3.30 (dt, J = 5.3, 2.5 Hz, 1H). ^{13}C NMR (126 MHz, DMSO-d_6) δ 162.38, 157.83, 157.76, 155.81, 155.70, 155.17, 144.55, 144.49, 143.21, 143.15, 140.52, 140.48, 139.91, 139.85, 135.06, 134.99, 132.98, 132.91, 111.78, 111.59, 110.90, 110.75, 106.66, 106.53, 102.65, 102.47, 78.18, 78.12, 75.11, 75.04, 29.52, 29.25. HRMS (ESI) calcd for $[M+H]^+$ $[C_{11}H_8FN_2O_2]^+$ 219.0564, observed mass 219.0528.

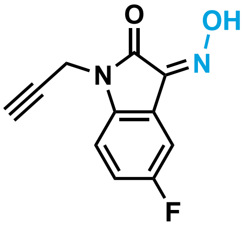

i3

63% yield. ^{1}H NMR (500 MHz, DMSO-d_6) δ 13.85 (s, 1H), 7.79 (dd, J = 8.2, 2.8 Hz, 1H), 7.38 (td, J = 9.1, 2.8 Hz, 1H), 7.20 (dd, J = 8.7, 4.1 Hz, 1H), 4.59 (d, J = 2.5 Hz, 2H), 3.30 (t, J = 2.5 Hz, 1H). ^{13}C NMR (126 MHz, DMSO) δ 162.64, 159.60, 157.70, 143.36, 138.61, 118.86, 116.32, 114.53, 111.27, 78.17, 75.09, 29.26. HRMS (ESI) calcd for $[M+H]^+$ $[C_{11}H_8FN_2O_2]^+$ 219.0564, observed mass 219.0528.

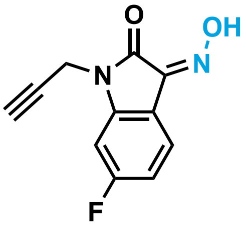

i4

75% yield. ^{1}H NMR (500 MHz, DMSO-d_6) δ 13.62 (s, 1H), 8.05 (dd, J = 8.4, 5.8 Hz, 1H), 7.14 (dd, J = 9.5, 2.4 Hz, 1H), 6.94 (ddd, J = 9.9, 8.4, 2.4 Hz, 1H), 4.59 (d, J = 2.5 Hz, 2H), 3.31 (t, J = 2.4 Hz, 1H). ^{13}C NMR (126 MHz, DMSO-d_6) δ 165.55, 163.57, 163.09, 144.62, 144.52, 142.73, 129.45, 112.44, 109.82, 99.08, 78.09, 75.14, 29.38. HRMS (ESI) calcd for $[M+H]^+$ $[C_{11}H_8FN_2O_2]^+$ 219.0564, observed mass 219.0528.

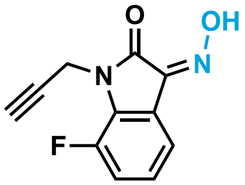

i5

76% yield. ^{1}H NMR (500 MHz, DMSO-d_6) δ 13.88 (s, 1H), 7.90 (dd, J = 7.6, 1.0 Hz, 1H), 7.40 (ddd, J = 11.7, 8.6, 1.0 Hz, 1H), 7.16 (ddd, J = 8.5, 7.5, 4.5 Hz, 1H), 4.61 (dd, J = 2.4, 1.0 Hz, 2H), 3.30 (t, J = 2.4 Hz, 1H). ^{13}C NMR (126 MHz, DMSO-d_6) δ 162.63, 148.19, 146.25, 142.91, 128.68, 124.82, 123.72, 120.36, 118.46, 78.92, 74.79, 31.30. HRMS (ESI) calcd for $[M+H]^+$ $[C_{11}H_8FN_2O_2]^+$ 219.0564, observed mass 219.0528.

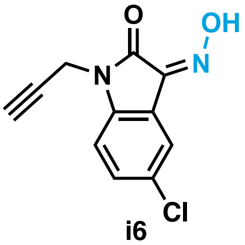

i6

90% yield. ^{1}H NMR (500 MHz, DMSO-d_6) δ 13.90 (s, 1H), 7.99 (d, J = 2.3 Hz, 1H), 7.58 (dd, J = 8.5, 2.2 Hz, 1H), 7.22 (d, J = 8.4 Hz, 1H), 4.59 (d, J = 2.6 Hz, 2H), 3.32 (t, J = 2.5 Hz, 1H). ^{13}C NMR (126 MHz, DMSO-d_6) δ 162.42, 142.91, 141.03, 131.98, 127.39, 126.68, 116.90, 111.75, 78.05, 75.20, 29.28. HRMS (ESI) calcd for $[M+H]^+$ $[C_{11}H_8ClN_2O_2]^+$ 235.0269, observed mass 235.0208.

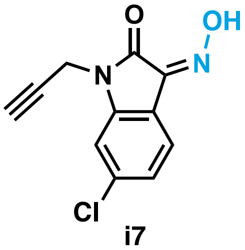

i7

87% yield. ^{1}H NMR (500 MHz, DMSO-d_6) δ 13.75 (s, 1H), 8.00 (d, J = 8.1 Hz, 1H), 7.32 (d, J = 1.8 Hz, 1H), 7.20 (dd, J = 8.1, 1.9 Hz, 1H), 4.61 (d, J = 2.6 Hz, 2H), 3.32 (t, J = 2.5 Hz, 1H). ^{13}C NMR (126 MHz, DMSO-d_6) δ 162.79, 143.64, 142.86, 136.73, 128.58, 123.30, 114.53, 110.58, 78.10, 75.18, 29.31. HRMS (ESI) calcd for $[M+H]^+$ $[C_{11}H_8ClN_2O_2]^+$ 235.0269, observed mass 235.0208.

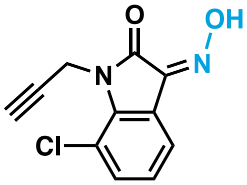

i8

80% yield. ^{1}H NMR (500 MHz, DMSO-d_6) δ 13.95 (s, 1H), 8.07 (dd, J = 7.5, 1.2 Hz, 1H), 7.49 (dd, J = 8.3, 1.2 Hz, 1H), 7.16 (dd, J = 8.3, 7.5 Hz, 1H), 4.81 (d, J = 2.4 Hz, 2H), 3.31 (t, J = 2.4 Hz, 1H). ^{13}C NMR (126 MHz, DMSO-d_6) δ 163.48, 142.37, 138.08, 134.26, 126.36, 125.11, 118.65, 115.48, 79.69, 75.13, 31.56. HRMS (ESI) calcd for $[M+H]^+$ $[C_{11}H_8ClN_2O_2]^+$ 235.0269, observed mass 235.0208.

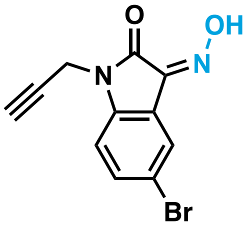

i9

91% yield. ^{1}H NMR (500 MHz, DMSO-d_6) δ 13.97 (s, 1H), 8.12 (d, J = 2.1 Hz, 1H), 7.71 (dd, J = 8.4, 2.2 Hz, 1H), 7.17 (d, J = 8.4 Hz, 1H), 4.59 (d, J = 2.5 Hz, 2H), 3.34 – 3.24 (m, 1H). ^{13}C NMR (126 MHz, DMSO-d_6) δ 162.33, 142.75, 141.35, 134.73, 129.34, 117.34, 115.06, 112.18, 78.02, 75.20, 29.26. HRMS (ESI) calcd for [M+H]+ $[C_{11}H_8BrN_2O_2]^+$ 278.9764, observed mass 278.9718.

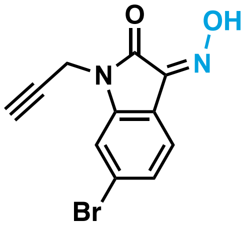

i10

78% yield. ^{1}H NMR (500 MHz, DMSO-d_6) δ 13.81 (s, 1H), 7.93 (d, J = 8.0 Hz, 1H), 7.58 – 7.18 (m, 2H), 4.61 (d, J = 2.5 Hz, 2H), 3.33 (t, J = 2.5 Hz, 1H). ^{13}C NMR (126 MHz, DMSO-d_6) δ 163.53, 162.94, 144.28, 143.22, 129.02, 109.15, 107.79, 97.59, 78.44, 74.90, 56.22, 29.12. HRMS (ESI) calcd for $[M+H]^+$ $[C_{11}H_8BrN_2O_2]^+$ 278.9764, observed mass 278.9718.

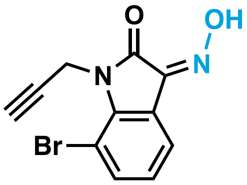

i11

81% yield. ^{1}H NMR (500 MHz, DMSO-d_6) δ 13.82 (s, 1H), 8.12 (d, J = 7.3 Hz, 1H), 7.65 (d, J = 8.1 Hz, 1H), 7.13 – 7.06 (m, 1H), 4.85 (d, J = 2.5 Hz, 2H), 3.33 – 3.29 (m, 1H). ^{13}C NMR (126 MHz, DMSO-d_6) δ 163.75, 142.25, 139.48, 137.51, 126.73, 125.40, 118.98, 102.85, 79.73, 75.36, 31.45. HRMS (ESI) calcd for $[M+H]^+$ $[C_{11}H_8BrN_2O_2]^+$ 278.9764, observed mass 278.9718.

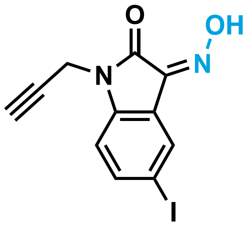

i12

84% yield. ^{1}H NMR (500 MHz, DMSO-d_6) δ 13.87 (s, 1H), 8.29 (d, J = 1.7 Hz, 1H), 7.85 (dt, J = 8.4, 1.5 Hz, 1H), 7.05 (d, J = 8.3 Hz, 1H), 4.57 (d, J = 2.6 Hz, 2H), 3.31 (t, J = 2.5 Hz, 1H). ^{13}C NMR (126 MHz, DMSO-d_6) δ 162.14, 142.64, 141.84, 140.57, 134.95, 117.72, 112.64, 86.51, 78.04, 75.20, 29.20. HRMS (ESI) calcd for $[M+H]^+$ $[C_{11}H_8IN_2O_2]^+$ 326.9625, observed mass 326.9617.

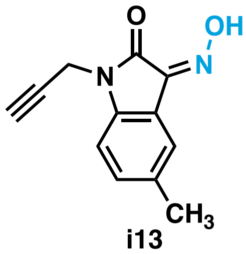

i13

83% yield. ^{1}H NMR (500 MHz, DMSO-d_6) δ 13.53 (s, 1H), 7.87 (d, J = 1.8 Hz, 1H), 7.32 – 7.28 (m, 1H), 7.06 (d, J = 8.0 Hz, 1H), 4.55 (d, J = 2.5 Hz, 2H), 3.27 (t, J = 2.5 Hz, 1H), 2.31 (s, 3H).^{13}C NMR (126 MHz, DMSO-d_6) δ 162.80, 143.80, 140.08, 132.68, 132.60, 127.91, 115.80, 109.89, 78.39, 74.86, 29.09, 21.03. HRMS (ESI) calcd for $[M+H]^+$ $[C_{12}H_{11}N_2O_2]^+$ 215.0815, observed mass 215.0825.

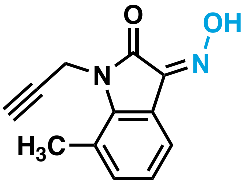

i14

91% yield. ^{1}H NMR (500 MHz, DMSO-d_6) δ 13.58 (s, 1H), 7.95 (dd, J = 7.6, 1.3 Hz, 1H), 7.24 (d, J = 7.8 Hz, 1H), 7.03 (t, J = 7.7 Hz, 1H), 4.71 (d, J = 2.5 Hz, 2H), 3.37 (t, J = 2.5 Hz, 1H), 2.63 (s, 3H). ^{13}C NMR (126 MHz, DMSO-d_6) δ 163.88, 143.33, 140.27, 136.16, 125.52, 123.65, 120.80, 116.56, 80.33, 75.35, 31.30, 18.33. HRMS (ESI) calcd for $[M+H]^+$ $[C_{12}H_{11}N_2O_2]^+$ 215.0815, observed mass 215.0855.

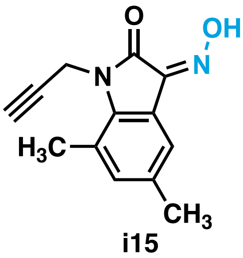

i15

79% yield. ^{1}H NMR (500 MHz, DMSO-d_6) δ 13.52 (s, 1H), 7.78 (d, J = 1.8 Hz, 1H), 7.08 – 7.03 (m, 1H), 4.68 (d, J = 2.5 Hz, 2H), 3.35 (t, J = 2.4 Hz, 1H), 2.58 (s, 3H), 2.25 (s, 3H). ^{13}C NMR (126 MHz, DMSO-d_6) δ 163.92, 143.49, 138.01, 136.49, 132.61, 125.98, 120.47, 116.67, 80.33, 75.26, 31.24, 20.73, 18.19. HRMS (ESI) calcd for $[M+H]^+$ $[C_{13}H_{13}N_2O_2]^+$ 229.0972, observed mass 229.0924.

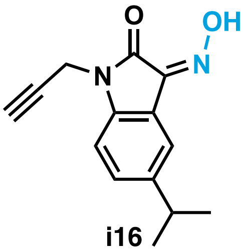

i16

65% yield. ^{1}H NMR (500 MHz, DMSO-d_6) δ 13.54 (s, 1H), 7.91 (d, J = 1.9 Hz, 1H), 7.38 (dd, J = 8.2, 1.9 Hz, 1H), 7.10 (d, J = 8.1 Hz, 1H), 4.56 (d, J = 2.6 Hz, 2H), 3.28 (t, J = 2.5 Hz, 1H), 2.91 (p, J = 6.9 Hz, 1H), 1.20 (d, J = 6.9 Hz, 6H). ^{13}C NMR (126 MHz, DMSO-d_6) δ 162.85, 143.84, 143.82, 140.42, 130.31, 125.37, 115.85, 110.02, 78.41, 74.90, 33.57, 29.10, 24.47. HRMS (ESI) calcd for $[M+H]^+$ $[C_{14}H_{15}N_2O_2]^+$ 243.1128, observed mass 243.1134.

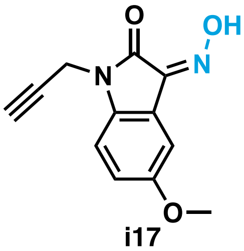

i17

92% yield. ^{1}H NMR (500 MHz, DMSO-d_6) δ 13.61 (s, 1H), 7.61 (dd, J = 2.4, 0.8 Hz, 1H), 7.12 – 7.07 (m, 2H), 4.55 (d, J = 2.6 Hz, 2H), 3.76 (s, 3H), 3.30 – 3.25 (m, 1H). ^{13}C NMR (126 MHz, DMSO-d_6) δ 162.68, 155.91, 143.93, 135.93, 117.47, 116.33, 113.57, 110.80, 78.42, 74.89, 56.15, 29.14. HRMS (ESI) calcd for $[M+H]^+$ $[C_{12}H_{11}N_2O_3]^+$ 231.0764, observed mass 231.0726.

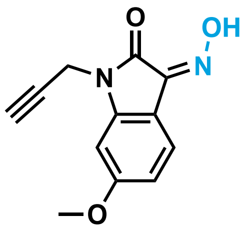

i18

83% yield. ^{1}H NMR (500 MHz, DMSO-d_6) δ 13.18 (s, 1H), 7.94 (d, J = 8.5 Hz, 1H), 6.91 – 6.54 (m, 2H), 4.57 (d, J = 2.6 Hz, 2H), 3.85 (s, 3H), 3.28 (t, J = 2.5 Hz, 1H). ^{13}C NMR (126 MHz, DMSO-d_6) δ 162.68, 143.57, 142.93, 128.72, 126.27, 125.43, 114.84, 113.28, 78.12, 75.19, 29.28. HRMS (ESI) calcd for $[M+H]^+$ $[C_{12}H_{11}N_2O_3]^+$ 231.0764, observed mass 231.0726.

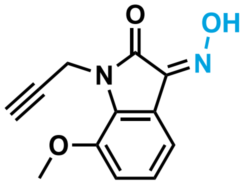

i19

83% yield. ^{1}H NMR (500 MHz, DMSO-d_6) δ 13.60 (s, 1H), 7.69 (dd, J = 7.5, 1.0 Hz, 1H), 7.24 (dd, J = 8.5, 1.1 Hz, 1H), 7.11 (dd, J = 8.5, 7.6 Hz, 1H), 4.67 (d, J = 2.5 Hz, 2H), 3.88 (s, 3H), 3.19 (t, J = 2.4 Hz, 1H).^{13}C NMR (126 MHz, DMSO-d_6) δ 163.37, 145.29, 143.65, 130.06, 124.56, 120.25, 117.59, 117.08, 79.92, 74.02, 57.12, 31.93. HRMS (ESI) calcd for $[M+H]^+$ $[C_{12}H_{11}N_2O_3]^+$ 231.0764, observed mass 231.0726.

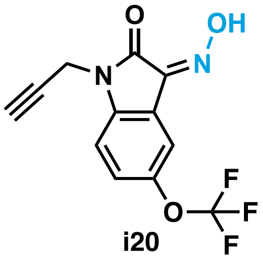

i20

80% yield. ^{1}H NMR (500 MHz, DMSO-d_6) δ 13.97 (s, 1H), 7.93 (d, J = 2.6 Hz, 1H), 7.55 (dt, J = 8.6, 1.8 Hz, 1H), 7.29 (d, J = 8.6 Hz, 1H), 4.61 (d, J = 2.6 Hz, 2H), 3.33 (t, J = 2.5 Hz, 1H). ^{13}C NMR (126 MHz, DMSO-d_6) δ 162.60, 144.07, 144.05, 142.99, 141.25, 125.63, 120.30, 116.35, 111.34, 78.01, 75.25, 29.33. HRMS (ESI) calcd for $[M+H]^+$ $[C_{12}H_8F_3N_2O_3]^+$ 285.0482, observed mass 285.0434.

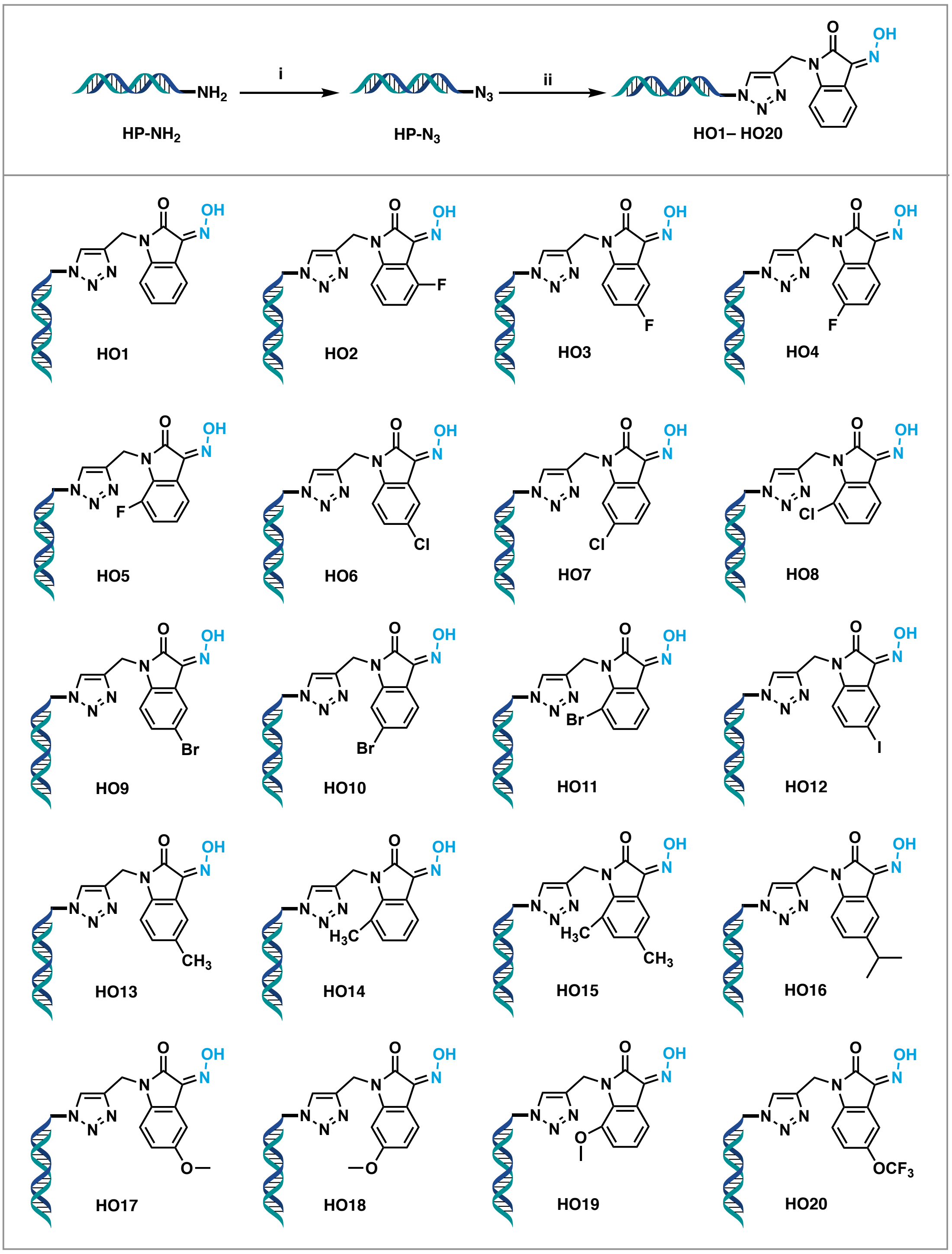

Scheme S5. Synthesis of DNA-conjugated isatin-oxime **HO1–HO20**.

Step i): To **HP-NH₂** (50 μL, 50 nmol, 1 mM in *dd*H_2O) was added 250 μL of borate buffer (pH 9.4, 250 mM in *dd*H_2O) and 100 μL of imidazole-1-sulfonyl azide tetrafluoroborate salt (40 mM in *dd*H_2O). The resulting mixture was vortexed and stood at 37°C for 16 hours. After that 5 M NaCl solution (10% by volume) and cold ethanol (2.5 times by volume, ethanol stored at -20°C) were added, vortexed, and incubated at -80°C for at least 30 minutes. The sample was centrifuged for 30 minutes at 4°C in a microcentrifuge at 12,000 rpm to remove the supernatant. The resulting pellet (precipitate) was re-dissolved in *dd*H_2O (50 μL) for LC-MS detection.

Step ii): To **HP-N_3** (10 μL, 10 nmol, 1 mM in pH 9.4 borate buffer) was added isatin oxime (10 μL, 800 nmol, 80 mM in DMSO), isatin oxime (10 μL, 800 nmol, 80 mM in DMSO), THPTA (10 μL, 800 nmol, 80 mM in DMSO), $CuSO_4$ (10 μL, 800 nmol, 80 mM in ddH_2O), ascorbate (10 μL, 1600 nmol, 160 mM in DMSO). The resulting mixture was vortexed and stood at r.t. for 3 hours. After that 5 M NaCl solution (10% by volume) and cold ethanol (2.5 times by volume, ethanol stored at -20°C) were added, vortexed, and incubated at -80°C for at least 30 minutes. The sample was centrifuged for 30 minutes at 4°C in a microcentrifuge at 12,000 rpm to remove the supernatant. The resulting pellet (precipitate) was re-dissolved in *dd*H_2O (10 μL) for LC-MS detection.

Table S2. Screening of the reaction parameters of on-DNA *N*-arylation

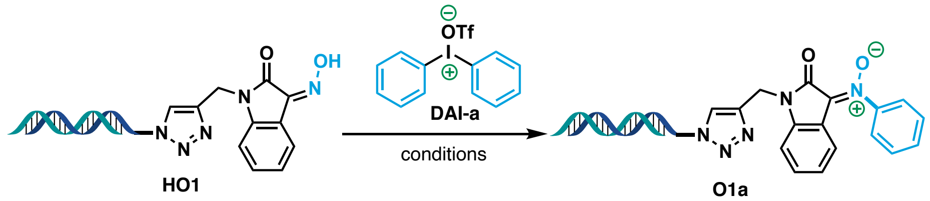

Entry	Base (equiv)	DAI-a (equiv)	Solvent	Temp.	Yield (%)[b]
**1**[a]	KOH (2000)	500	*dd*H_2O/MeOH = 1:4	r.t.	84
**2**	NaOH (2000)	500	*dd*H_2O/MeOH = 1:1	r.t.	60
**3**	KOH (2000)	500	*dd*H_2O/DMA = 1:1	r.t.	30
**4**	KOH (2000)	500	*dd*H_2O/MeOH = 1:1	r.t.	73
**5**	KOH (2000)	500	*dd*H_2O/MeOH = 4:1	r.t.	50
**6**	KOH (2000)	500	*dd*H_2O/MeOH = 1:5	r.t.	62
**7**	KOH (2000)	500	*dd*H_2O/MeOH = 1:9	r.t.	59
**8**	KOH (1500)	500	*dd*H_2O/MeOH = 1:4	r.t.	68
**9**	KOH (500)	500	*dd*H_2O/MeOH = 1:4	r.t.	72
**10**	KOH (2000)	1000	*dd*H_2O/MeOH = 1:4	r.t.	61
**11**	KOH (2000)	750	*dd*H_2O/MeOH = 1:4	r.t.	71
**12**	KOH (2000)	250	*dd*H_2O/MeOH = 1:4	r.t.	63

[a]Reactions were performed at r.t. with **HO1** (2 nmol, 1mM in *dd*H_2O), **DAI-a** (500 eq, 500 mM in MeOH), KOH (2000 eq, 500mM in *dd*H_2O) in total volumes of 50 μL aqueous solutions (V_{ddH2O}/V_{MeOH} = 1:4), 2 h. [b]The conversion yield was determined by LC/MS.

General procedure (vi) for the on-DNA N-arylation of isatin-oxime

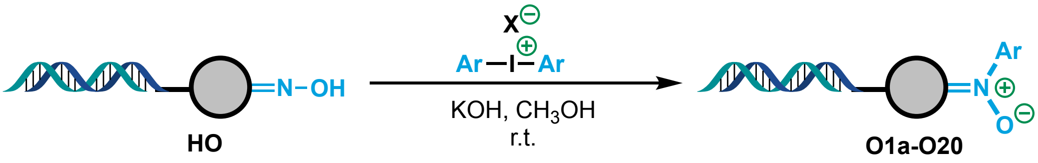

To **HO** (2 μL, 2 nmol, 1 mM in *dd*H_2O) was added **DAI** (2 μL, 1000 nmol, 500 mM in MeOH), KOH (8 μL, 4000 nmol, 500 mM in *dd*H_2O) and 38 μL MeOH. The resulting mixture was vortexed and stood at room temperature for 2 hours. After that 5 M NaCl solution (10% by volume) and cold ethanol (2.5 times by volume, ethanol stored at -20°C) were added, vortexed, and incubated at -80°C for at least 30 minutes. The sample was centrifuged for 30 minutes at 4°C in a microcentrifuge at 12,000 rpm to remove the supernatant. The resulting pellet (precipitate) was re-dissolved in *dd*H_2O (2 μL) for LC-MS detection.

IV Co-injection Experiment

Co-injection experiment (i) of on-DNA O-arylation of phenols

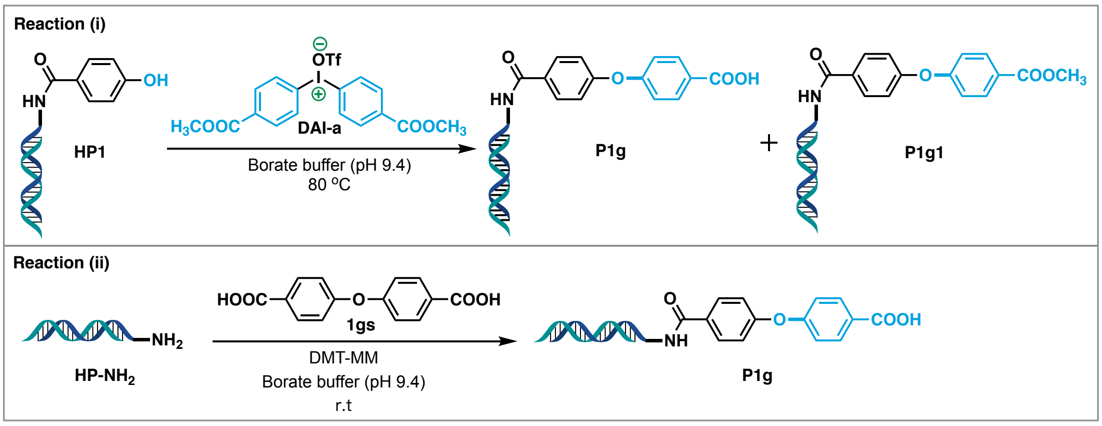

Scheme S6. Synthesis of **P1g** from **HP1** or **HP-NH₂** and acid **1gs**.

Reaction (i) was performed according to the ***general procedure (v)***; Reaction (ii): To a solution of **HP-NH$_2$** (10 μL, 10 nmol) in borate buffer (250 mM, pH = 9.4), was added a mixture of DMA solution of DMTMM (2 μL, 200 mM), **1gs** (2 μL, 200 mM), 2 μL *dd*H_2O of and 12 μL of borate buffer (250 mM, pH = 9.4). The resultant mixture was vortexed and stood at 25 °C for 8 hours. After that 5 M NaCl solution (10% by volume) and cold ethanol (2.5 times by volume, ethanol stored at -20°C) were added, vortexed, and incubated at -80°C for at least 30 minutes. The sample was centrifuged for 30 minutes at 4°C in a microcentrifuge at 12,000 rpm to remove the supernatant. The resulting pellet (precipitate) was re-dissolved in *dd*H_2O (10 μL) for LC-MS detection.

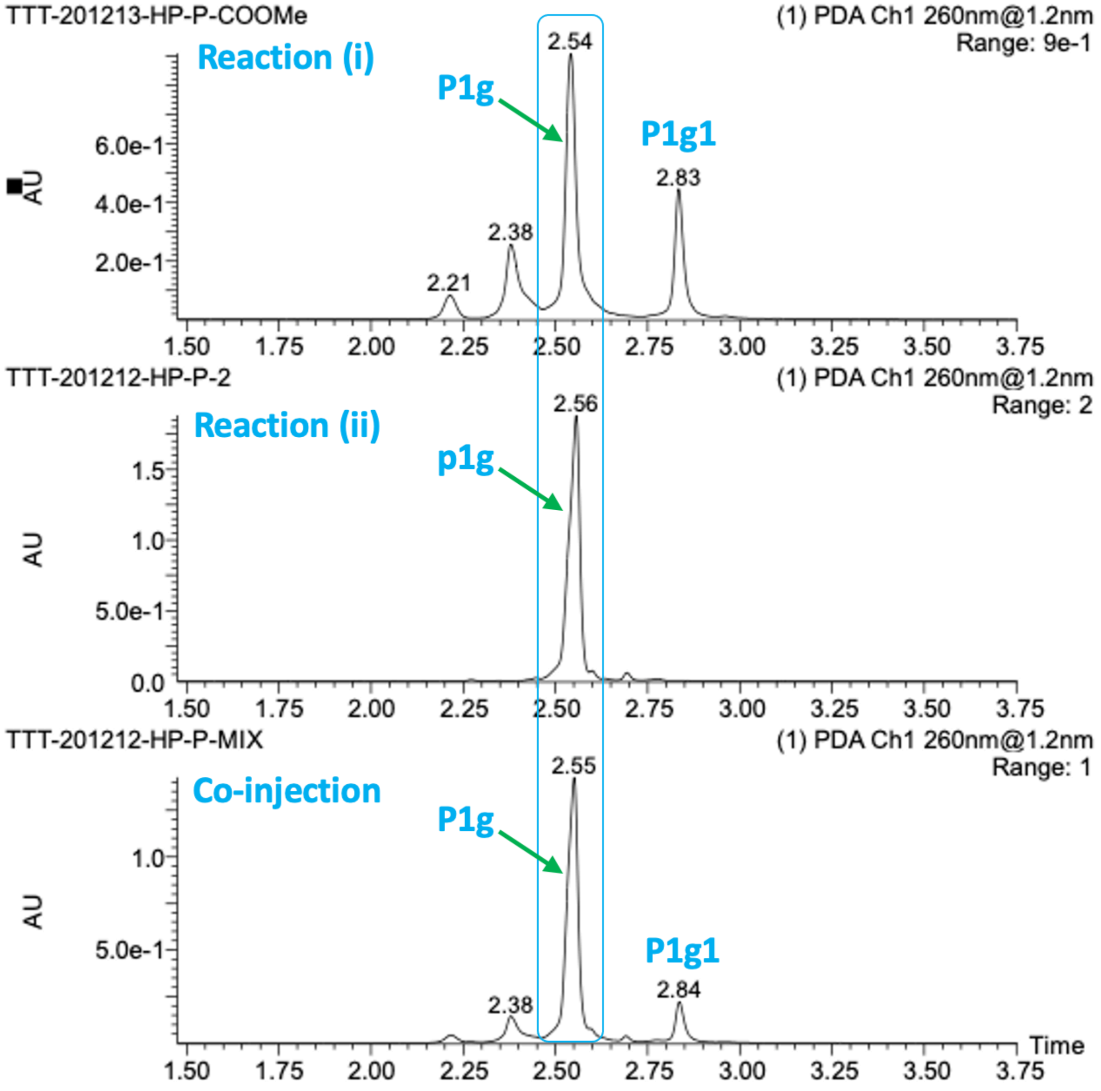

Figure S3. LC trace of reaction (**i**) and reaction (**ii**).

Co-injection experiment (ii) of on-DNA N-arylation of isatin-oxime

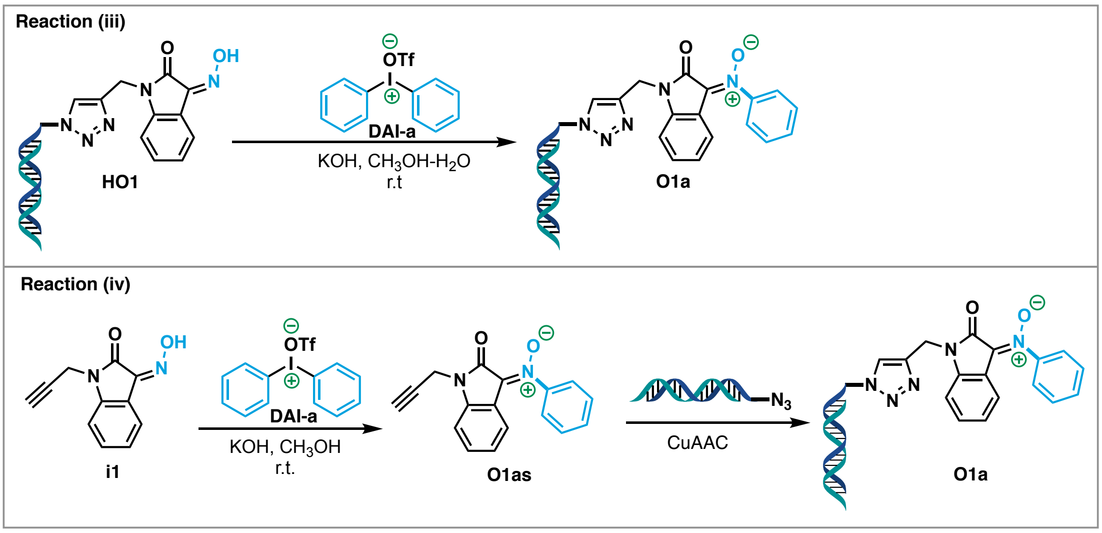

Scheme S7. Synthesis of **O1a** from **HO1** (reaction iii) or **i1** (Reaction iv).

Reaction (iii) was performed as according to ***general procedure (v)***.
Reaction (iv) i1 (1 mmol, 1 equiv) was dissolved in 5 mL MeOH, then KOH (1.5 mmol, 1.5 equiv) and **DAIa** (1.5 mmol, 1.5 equiv) was added to the solution. The reaction mixture was stirred at room temperature for three hours before concentrated in vacuo. Then the crude reaction mixture was extracted with CH_2Cl_2/H_2O, the organic layer was concentrated in vacuo. Then the crude product was applied on a silica plug (10 g) and eluted with CH_2Cl_2 (10 mL) followed by $MeOH/CH_2Cl_2$ (1:100-1:10) to elute the product. To **HP-N₃** (10 μL, 10 nmol, 1 mM in pH 9.4 borate buffer) was added **O1as** (10 μL, 800 nmol, 80 mM in DMSO), isatin oxime (10 μL, 800 nmol, 80 mM in DMSO), THPTA (10 μL, 800 nmol, 80 mM in DMSO), $CuSO_4$ (10 μL, 800 nmol, 80 mM in *dd*H_2O), ascorbate (10 μL, 1600 nmol, 160 mM in DMSO). The resulting mixture was vortexed and stood at r.t. for 3 hours. After that 5 M NaCl solution (10% by volume) and cold ethanol (2.5 times by volume, ethanol stored at -20°C) were added, vortexed, and incubated at -80°C for at least 30 minutes. The sample was centrifuged for 30 minutes at 4°C in a microcentrifuge at 12,000 rpm to remove the supernatant. The resulting pellet (precipitate) was re-dissolved in *dd*H_2O (10 μL) for LC-MS detection.

O1as: 80% yield. ^{1}H NMR (500 MHz, DMSO-d_6) δ 8.24 (d, J = 7.5 Hz, 1H), 7.62 (td, J = 7.8, 1.4 Hz, 1H), 7.50 – 7.43 (m, 4H), 7.28 – 7.18 (m, 3H), 4.63 (d, J = 2.5 Hz, 2H), 3.34 (s, 1H).^{13}C NMR (126 MHz, DMSO) δ 161.90, 159.02, 146.03, 143.72, 134.54, 130.27, 128.98, 124.66, 124.05, 115.34, 115.17, 110.73, 78.03, 75.26, 29.45. HRMS (ESI) calcd for $[M+H]^+$ $[C_{17}H_{13}N_2O_2]^+$ 277.0972, observed mass 277.0919.

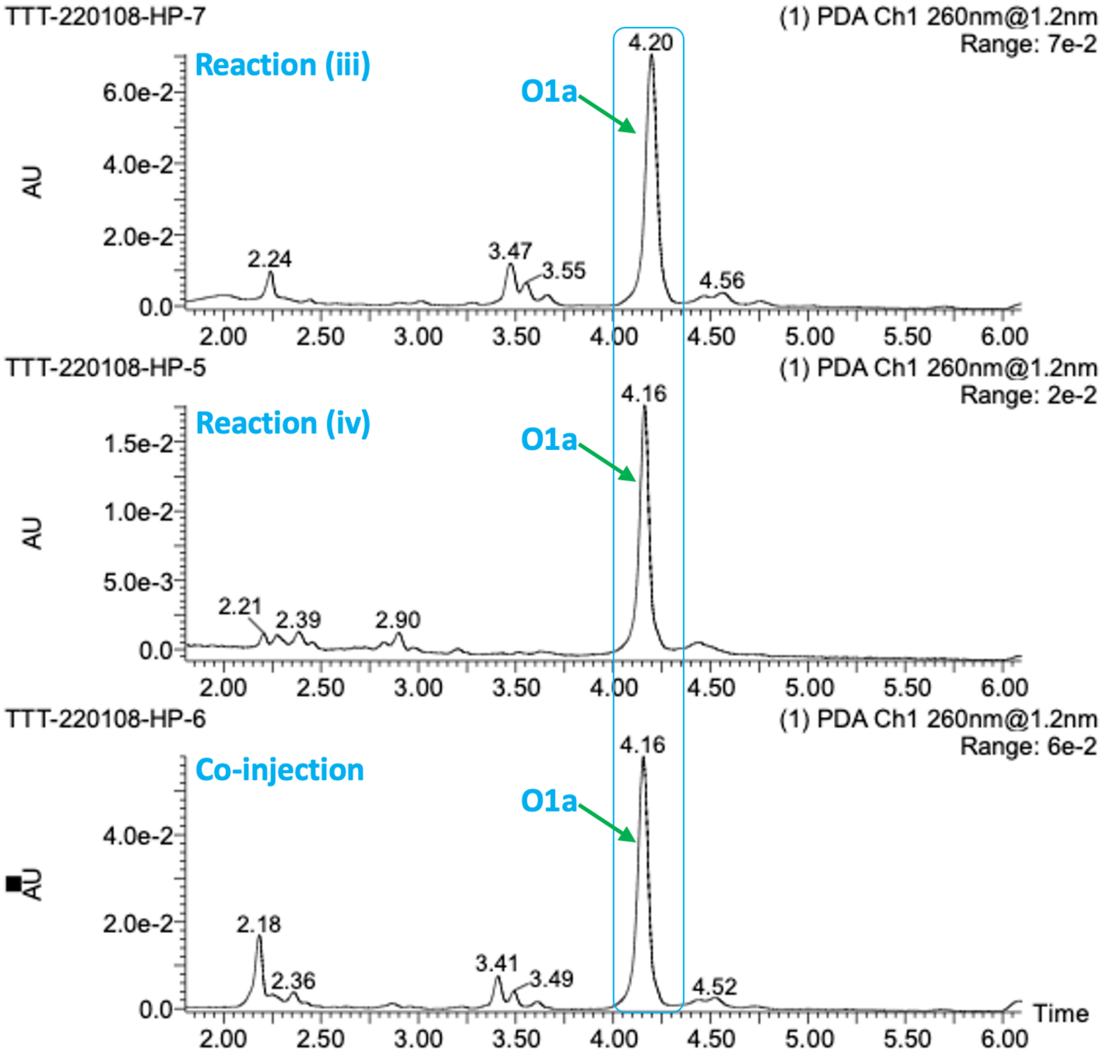

Figure S4. LC trace of reaction (**iii**) and reaction (**iv**).

V. Evaluation of the DNA Tags Degradation

DNA Ligation and sequencing:

The ligation sample was prepared by mixing 5 μL Headpiece (1mM in H_2O) with 5 μL 50 bp oligo DNA (1mM in ddH_2O, prepared by annealing of 2 mM 50 bp-F and 2 mM 50 bp-R). To 0.3 nmol sample in pellet form, 1 μL 10 × T4 DNA reaction buffer (NEB, B0202S), 1 μL T4 DNA ligase (NEB, M0202L), 8 μL ddH_2O were added. The reaction mixtures were kept at room temperature for two hours. Reaction samples were then heated at 65°C for 10 minutes to denature the T4 DNA ligase. Gel electrophoresis is usually executed by using 15% TBE acrylamide gel (5.25 mL 40% acryl (29:1), 7.2 mL MilliQ water,1.4 mL 10 × TBE, 140 μL APS, 6 µL TEMED). The gel box (Bio-Rad, Mini-PROTEAN® Tetra electrophoresis) was filled with 0.5×TAE buffer until the gel was covered. The first lane of the gel was loaded with a DNA ladder, followed by loading 50 ng of DNA sample into each lane. Gel was run at 110 V for 90 min and was stained with 3000 × GelRed from Tanon™ (170-3001) in 15 mL 0.5xTBE buffer for 15 min. DNA fragments were visualized under a UV light device.

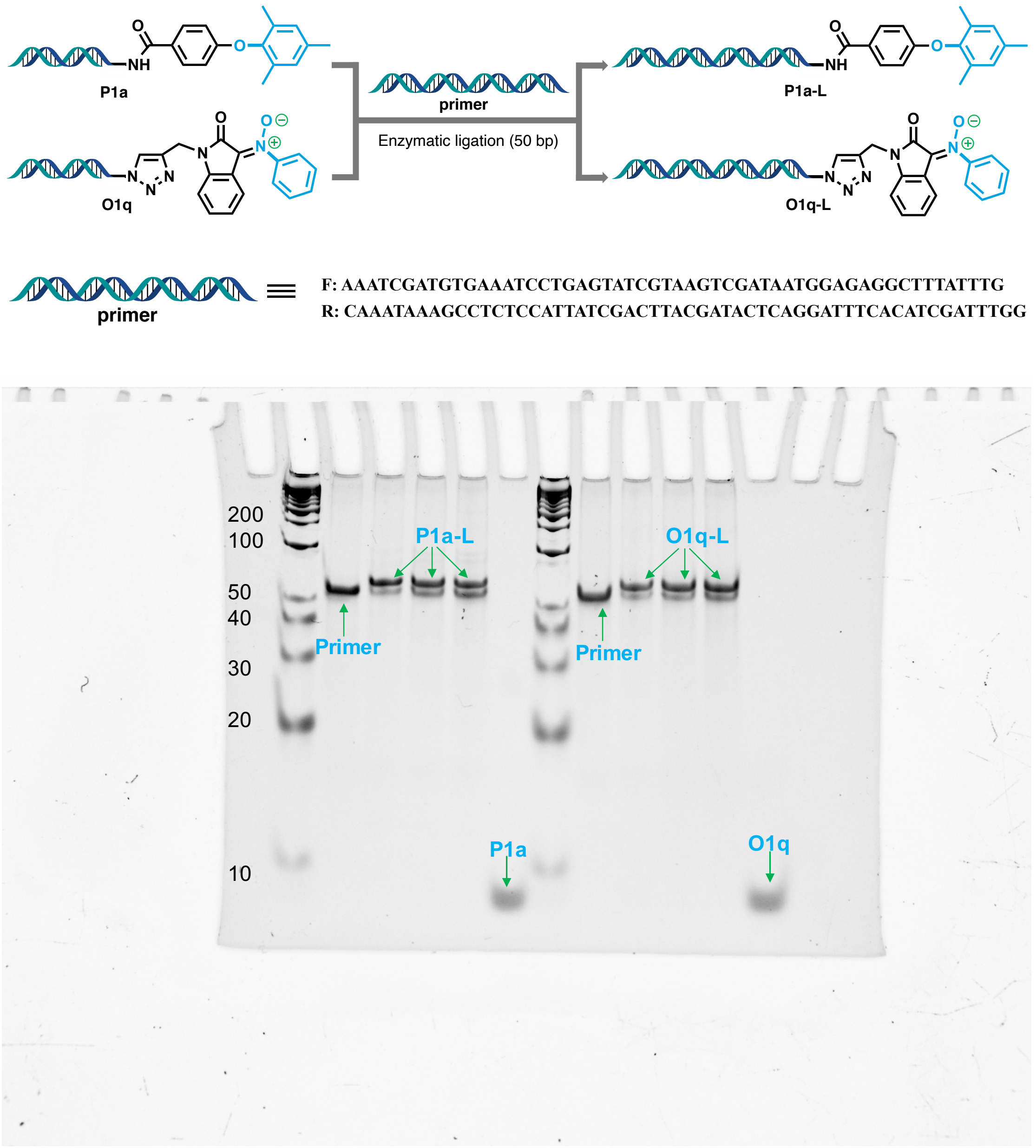

Figure S5. DNA ligation reaction analysis for synthesis of diaryl ethers and aryl oximes.

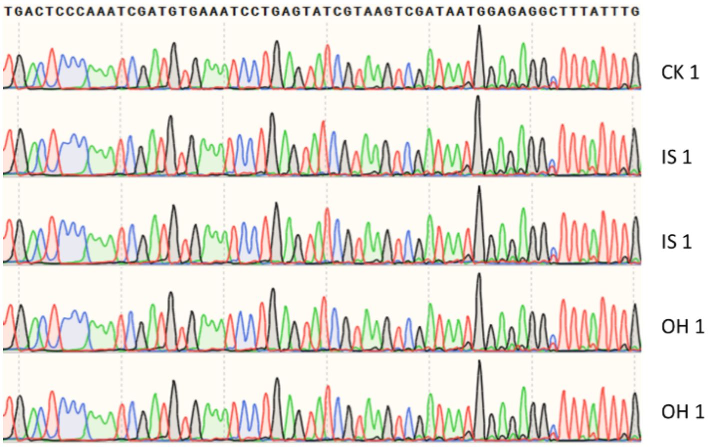

Figure S6. All DNA sample were amplificated by two rounds' PCR steps to reach 184 bp length before sequencing. Following manufacturer's guidelines, 1 ng of former DNA ligation sample was mix with 1 μL of 10 μM primer PCR1-F and PCR1-R, 25 μL 2 × Primer STAR Max DNA Polymerase (Takara, R045A) to reach a 50 μL system finally. PCR cycles as follows: 98 °C heat activation for 1 min followed by 34 cycles of 98 °C denaturation for 10 seconds, 60 °C annealing for 5 seconds and extension at 72 °C for 10 seconds. DNA sample was evaluation by 2% DNA gel and extracted by NucleoSpin Gel Clean-up Mini kit (MACHEREY-NAGEL, 740609.50). After PCR1, PCR2 was executed as the same step by using PCR1 purified sample. DNA sequencing was sent to Sanger sequencing by using primer Sequencing-F.

PCR1-F: CTCTTTCCCTACACGACGCTCTTCCGATCTTGACTCCCAAATC
PCR1-R: GTGACTGGAGTTCAGACGTGTGCTCTTCCGATCAAATAAAGCCTC
PCR2-F:
AATGATACGGCGACCACCGAGATCTACACTCTTTCCCTACACGACGCTC
PCR2-R:
CAAGCAGAAGACGGCATACGAGATGTCGTGATGTGACTGGAGTTCAGACG
TGTGC
Sequencing-F: AATGATACGGCGACC

qPCR Analysis:

qPCR was performed on the Applied Biosystems QuantStudio 7 Real-Time PCR System using 96-well plates. The sample mixtures of 20 μL total volume contained the following: 7 μL *dd*H_2O, 1 μL primer mix (5 μM each, reverse and forward primer), 2 μL diluted DNA sample and 10 μL Bimake™ 2 × SYBR Green qPCR master mix (Low ROX; B21702). The PCR reaction was carried out with the following thermocycling program: 95 °C, 60 s; 40 cycles of (95 °C, 15 s; 55 °C, 30 s; 72 °C, 30 s). Every assay was repeated at least three times.

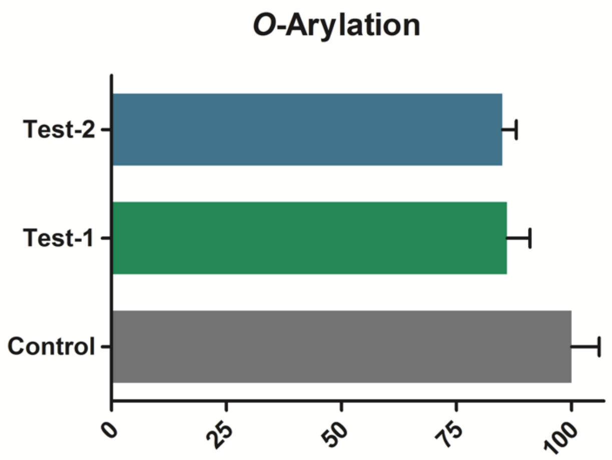

Figure S7. qPCR analysis of residual amplifiable material after the reaction condition of synthesis of diaryl ethers (*O*-Arylation). In a 100 μL polypropylene plate, a solution of a full-length DNA-encoded library (ca. 0.5 mM in water, 10 μL, ca. 5 nmol) was combined with 40 μL *dd*H_2O (control) or combined with **DAI-q** (20 µL, 50 mM in DMA) and 25 μL of borate buffer (pH 9.4, 250 mM) (Test 1 and Test 2). The plate was sealed (adhesive aluminum foil), briefly vortexed, and incubated at 80°C for 150 min. All conditions were tested in triplicate (3×3 wells in total). Each sample was then diluted (three times 1:100, 2 μL sample in 198 μL *dd*H_2O), and desalted by gel filtration (GE healthcare Illustra, G-25 microspin columns) as per the manufacturer's instruction. Amount of amplifiable material was determined by qPCR analysis in technical sextuplets.

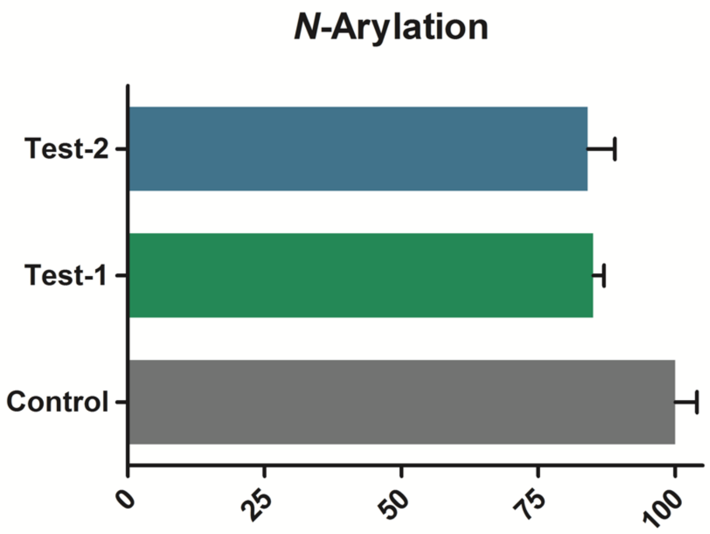

Figure S8. qPCR analysis of residual amplifiable material after the reaction condition of N-arylation aryl oxime. In a 100 μL polypropylene plate, a solution of a full-length DNA-encoded library (ca. 0.5 mM in *dd*H_2O, 4 μL, ca. 5 nmol) was combined with 46 μL water (control) or combined with **DAI-a** (2 μL, 500 mM in MeOH), KOH (8 μL, 500 mM in *dd*H_2O) and 38 μL of MeOH (pH 9.4, 250 mM) (Test 1 and Test 2). The plate was sealed (adhesive aluminum foil), briefly vortexed, and incubated at room temperature for 120 min. All conditions were tested in triplicate (3 × 3 wells in total). Each sample was then diluted (three times 1:100, 2 μL sample in 198 μL *dd*H_2O), and desalted by gel filtration (GE healthcare Illustra, G-25 microspin columns) as per the manufacturer's instruction. Amount of amplifiable material was determined by qPCR analysis in technical sextuplets.

VI. References

1 Bielawski, M. & Olofsson, B. High-yielding one-pot synthesis of diaryliodonium triflates from arenes and iodine or aryl iodides. *Chem Commun (Camb)*, 2521-2523, doi:10.1039/b701864a (2007).

2 Bielawski, M., Aili, D. & Olofsson, B. Regiospecific one-pot synthesis of diaryliodonium tetrafluoroborates from arylboronic acids and aryl iodides. *J Org Chem* **73**, 4602-4607, doi:10.1021/jo8004974 (2008).

3 Zhu, M., Jalalian, N. & Olofsson, B. One-Pot Synthesis of Diaryliodonium Salts Using Toluenesulfonic Acid: A Fast Entry to Electron-Rich Diaryliodonium Tosylates and Triflates. *Synlett* **2008**, 592-596 (2008).

4 Gu, Y., Chang, D., Leng, X., Gu, Y. & Shen, Q. Well-Defined, Shelf-Stable (NHC)Ag(CF2H) Complexes for Difluoromethylation. *Organometallics* **34**, 3065-3071, doi:10.1021/acs.organomet.5b00350 (2015).

5 Monastyrskyi, A., Namelikonda, N. K. & Manetsch, R. Metal-free arylation of ethyl acetoacetate with hypervalent diaryliodonium salts: an immediate access to diverse 3-aryl-4(1H)-quinolones. *J Org Chem* **80**, 2513-2520, doi:10.1021/jo5023958 (2015).

6 Beaud, R., Phipps, R. J. & Gaunt, M. J. Enantioselective Cu-Catalyzed Arylation of Secondary Phosphine Oxides with Diaryliodonium Salts toward the Synthesis of P-Chiral Phosphines. *J Am Chem Soc* **138**, 13183-13186, doi:10.1021/jacs.6b09334 (2016).

7 Hong, X., Tan, Q., Liu, B. & Xu, B. Isocyanide-Induced Activation of Copper Sulfate: Direct Access to Functionalized Heteroarene Sulfonic Esters. *Angew Chem Int Ed Engl* **56**, 3961-3965, doi:10.1002/anie.201612565 (2017).

8 Neerbye Berntsen, L., Nova, A., Wragg, D. S. & Sandtorv, A. H. Cu-catalyzed N-3-Arylation of Hydantoins Using Diaryliodonium Salts. *Organic letters* **22**, 2687-2691, doi:10.1021/acs.orglett.0c00642 (2020).

9 Pauton, M. *et al.* Development, Optimization, and Scope of the Radiosynthesis of 3/5-[18F]Fluoropyridines from Readily Prepared Aryl(pyridinyl) Iodonium Salts: The Importance of TEMPO and K2CO3. *Organic Process Research & Development* **23**, 900-911, doi:10.1021/acs.oprd.9b00021 (2019).

10 Du, H. C. *et al.* A Mild, DNA-Compatible Nitro Reduction Using B2(OH)4. *Organic letters* **21**, 2194-2199, doi:10.1021/acs.orglett.9b00497 (2019).

11 Deng, H. *et al.* Discovery of highly potent and selective small molecule ADAMTS-5 inhibitors that inhibit human cartilage degradation via encoded library technology (ELT). *Journal of medicinal chemistry* **55**, 7061-7079, doi:10.1021/jm300449x (2012).

12 Silva, M., Gonçalves, J. C. O., Oliveira-Campos, A. M. F., Rodrigues, L. M. & Esteves, A. P. Synthesis of Novel Glycoconjugates Derived From Alkynyl Heterocycles through a Click Approach. *Synthetic Communications* **43**, 1432-1438, doi:10.1080/00397911.2011.637655 (2013).

13 Kasprzak, A., Bystrzejewski, M., Koszytkowska-Stawinska, M. & Poplawska, M. Grinding-induced functionalization of carbon-encapsulated iron nanoparticles. *Green Chemistry* **19**, 3510-3514, doi:10.1039/c7gc00282c (2017).

14 Tri, N. M. *et al.* Study on synthesis of some substituted N-propargyl isatins by propargylation reaction of corresponding isatins using potassium carbonate as base under

ultrasound- and microwave-assisted conditions. *Chemical Papers* **75**, 4793-4801, doi:10.1007/s11696-021-01697-6 (2021).

15 Yu, B. *et al.* Efficient synthesis of new antiproliferative steroidal hybrids using the molecular hybridization approach. *European journal of medicinal chemistry* **117**, 241-255, doi:10.1016/j.ejmech.2016.04.024 (2016).

16 Jha, M., Shelke, G. M. & Kumar, A. Catalyst-Free One-Pot Tandem Reduction of Oxo and Ene/Yne Functionalities by Hydrazine: Synthesis of Substituted Oxindoles from Isatins. *European Journal of Organic Chemistry* **2014**, 3334-3336, doi:10.1002/ejoc.201402038 (2014).

17 Day, J. *et al.* Alkaloid inspired spirocyclic oxindoles from 1,3-dipolar cycloaddition of pyridinium ylides. *Org Biomol Chem* **11**, 6502-6509, doi:10.1039/c3ob41415a (2013).

18 Wonganan, O. *et al.* Potent vasorelaxant analogs from chemical modification and biotransformation of isosteviol. *European journal of medicinal chemistry* **62**, 771-776, doi:10.1016/j.ejmech.2013.01.022 (2013).

19 Khaybullin, R. N. *et al.* Design and synthesis of isosteviol triazole conjugates for cancer therapy. *Molecules (Basel, Switzerland)* **19**, 18676-18689, doi:10.3390/molecules191118676 (2014).

20 Skorobogatyi, M. V. *et al.* 5-Alkynyl-2′-deoxyuridines, containing bulky aryl groups: evaluation of structure–anti-HSV-1 activity relationship. *Tetrahedron* **62**, 1279-1287, doi:10.1016/j.tet.2005.10.057 (2006).

21 Gironda-Martinez, A., Neri, D., Samain, F. & Donckele, E. J. DNA-Compatible Diazo-Transfer Reaction in Aqueous Media Suitable for DNA-Encoded Chemical Library Synthesis. *Organic letters* **21**, 9555-9558, doi:10.1021/acs.orglett.9b03726 (2019).

22 Chen, Y., Kamlet, A. S., Steinman, J. B. & Liu, D. R. A biomolecule-compatible visible-light-induced azide reduction from a DNA-encoded reaction-discovery system. *Nat Chem* **3**, 146-153, doi:10.1038/nchem.932 (2011).

23 Wu, S. Y., Ma, X. P., Liang, C. & Mo, D. L. Synthesis of N-Aryl Oxindole Nitrones through a Metal-Free Selective N-Arylation Process. *J Org Chem* **82**, 3232-3238, doi:10.1021/acs.joc.6b02774 (2017).

24 Sheradsky, T. & Nov, E. Studies on the preparation of N-alkyl-O-phenylhydroxylamines. *Journal of the Chemical Society, Perkin Transactions 1*, 2781-2786, doi:10.1039/P19800002781 (1980).

VII. Copies of ^{1}H, ^{13}C NMR, HPLC and MS Spectra

^{1}H NMR of DAI-b

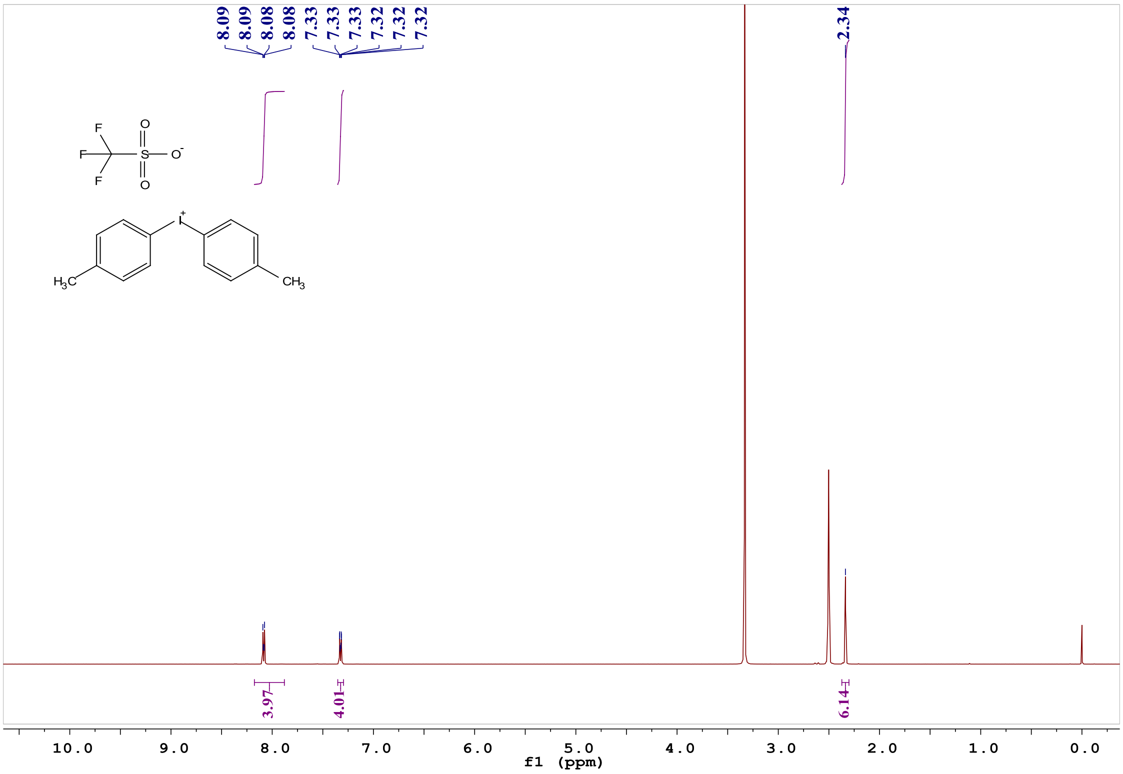

^{1}H NMR of DAI-c

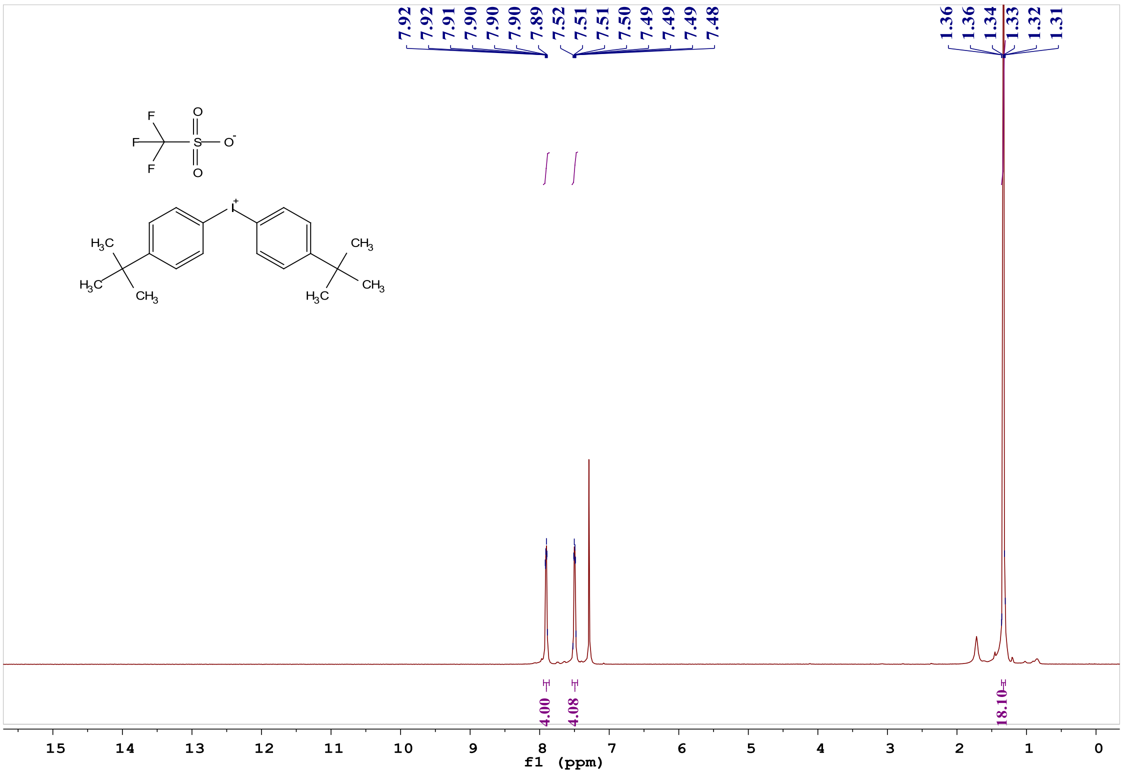

^{1}H NMR of DAI-d

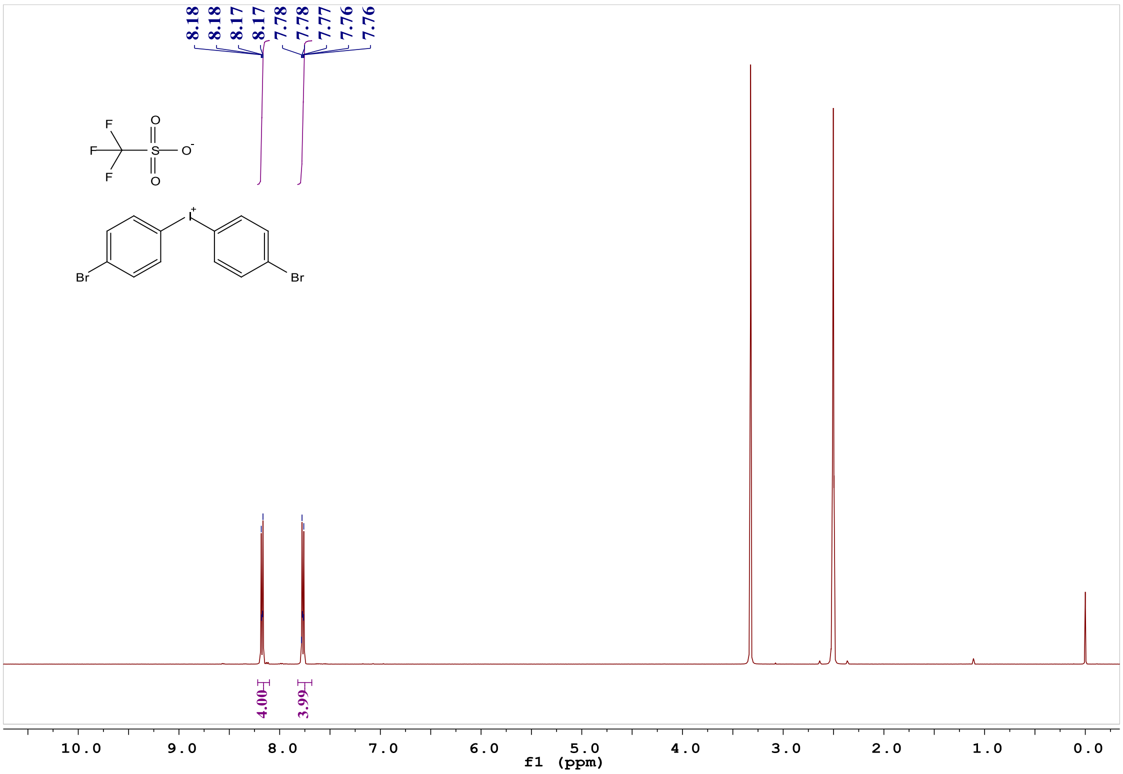

^{1}H NMR of DAI-e

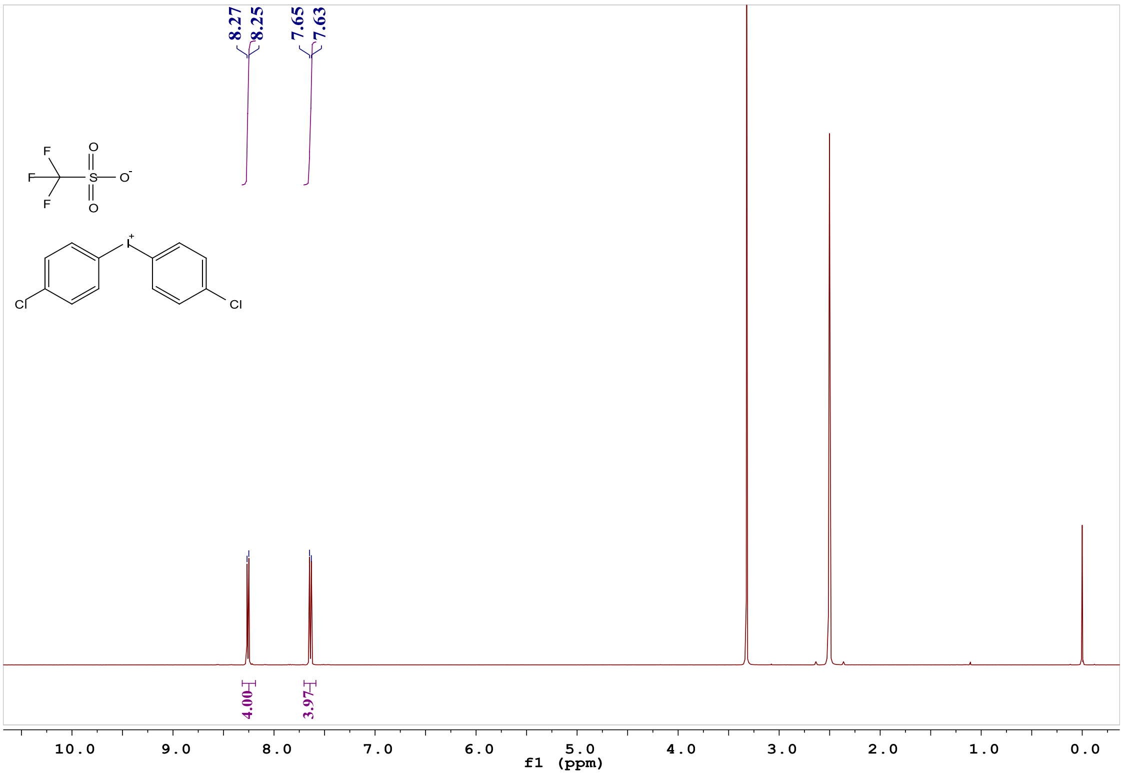

^{1}H NMR of DAI-f

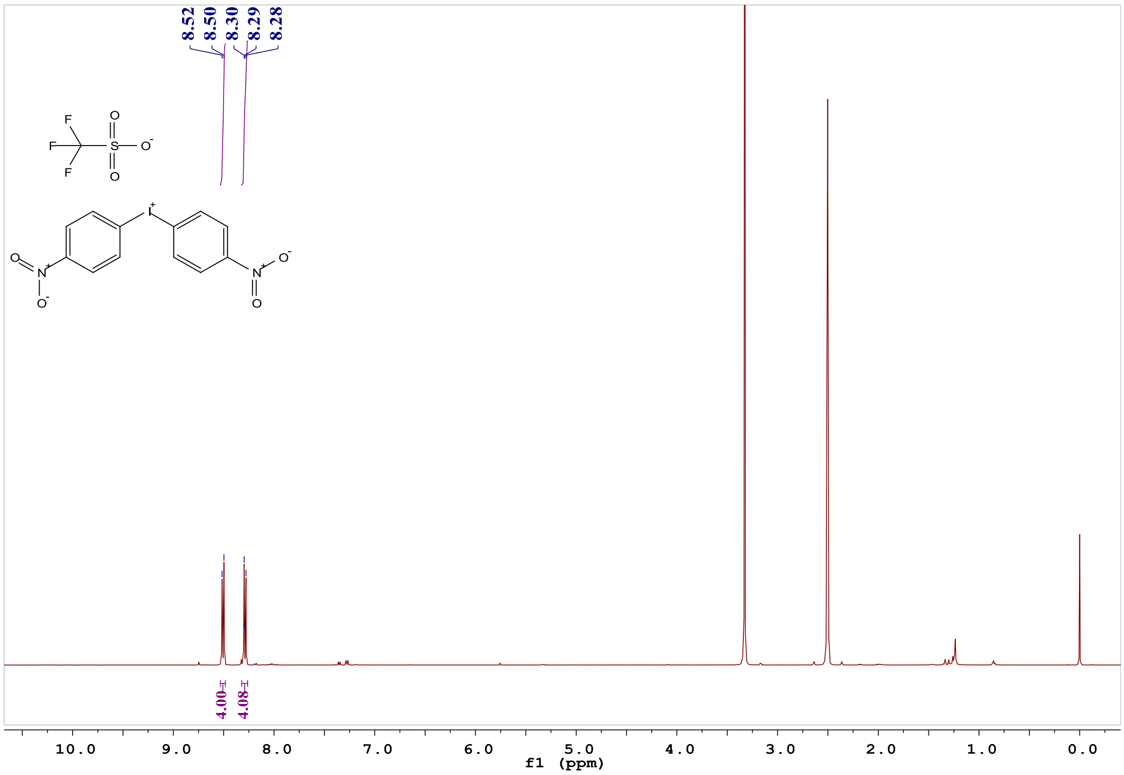

^{13}C NMR of DAI-f

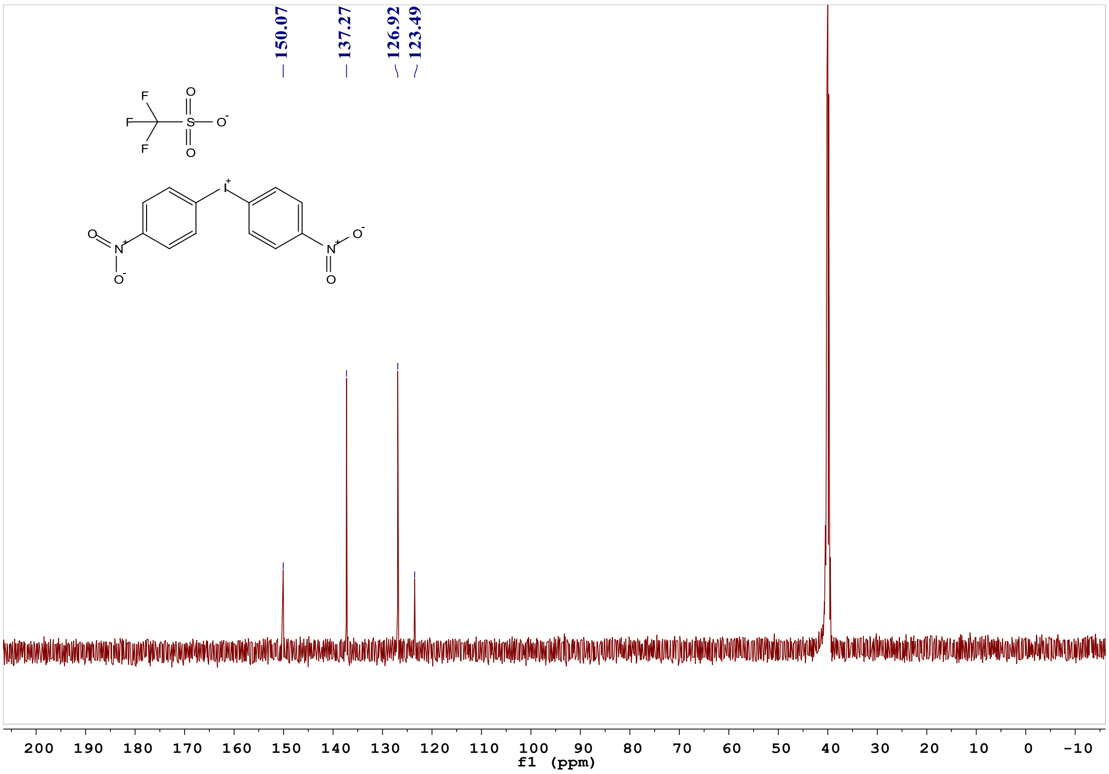

^{1}H NMR of DAI-g

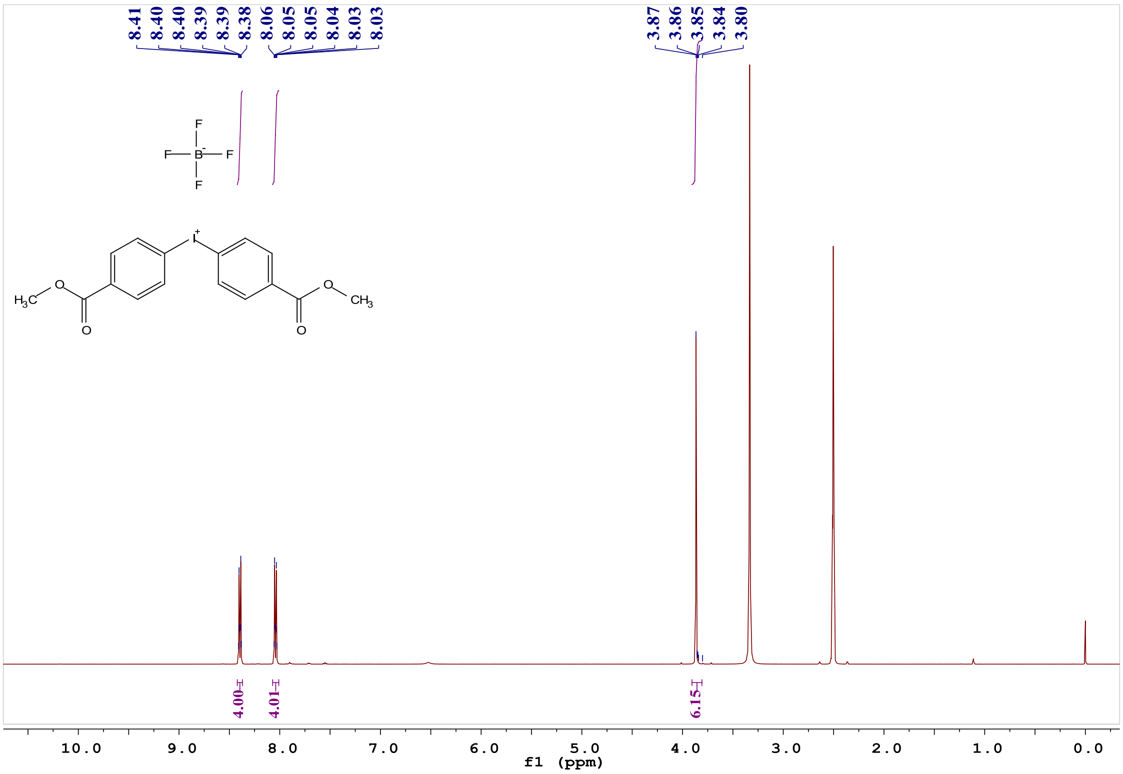

^{1}H NMR of DAI-h

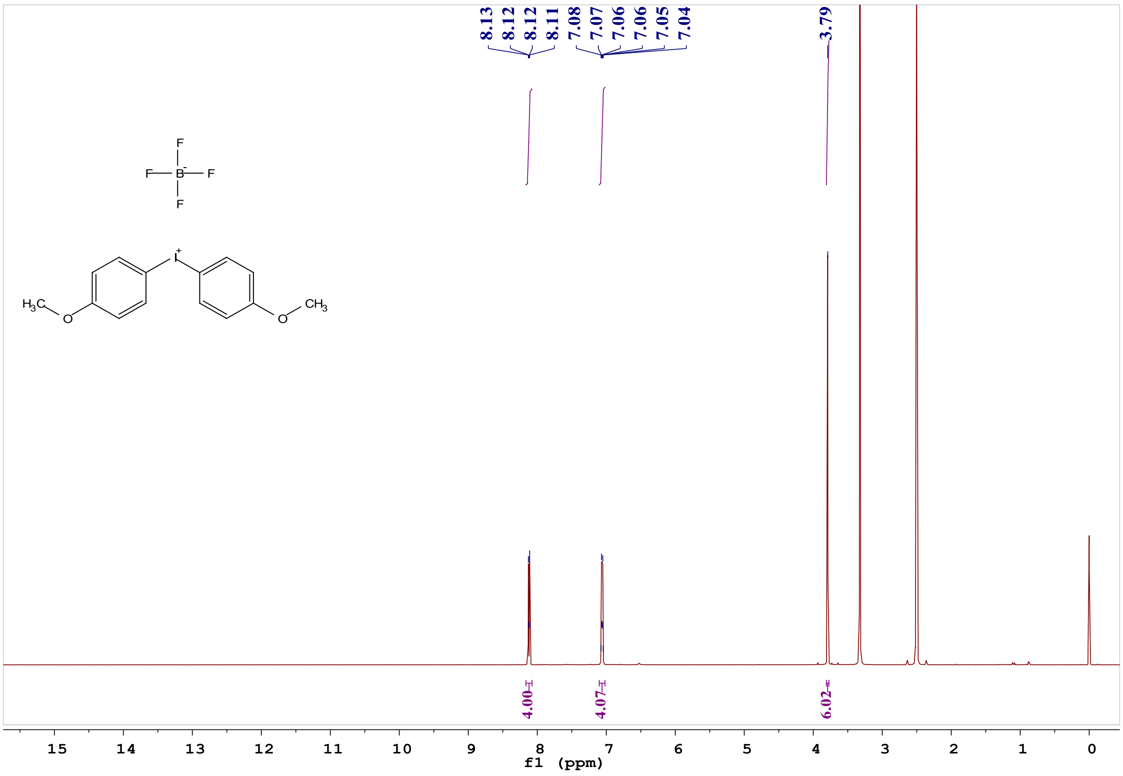

^{1}H NMR of DAI-i

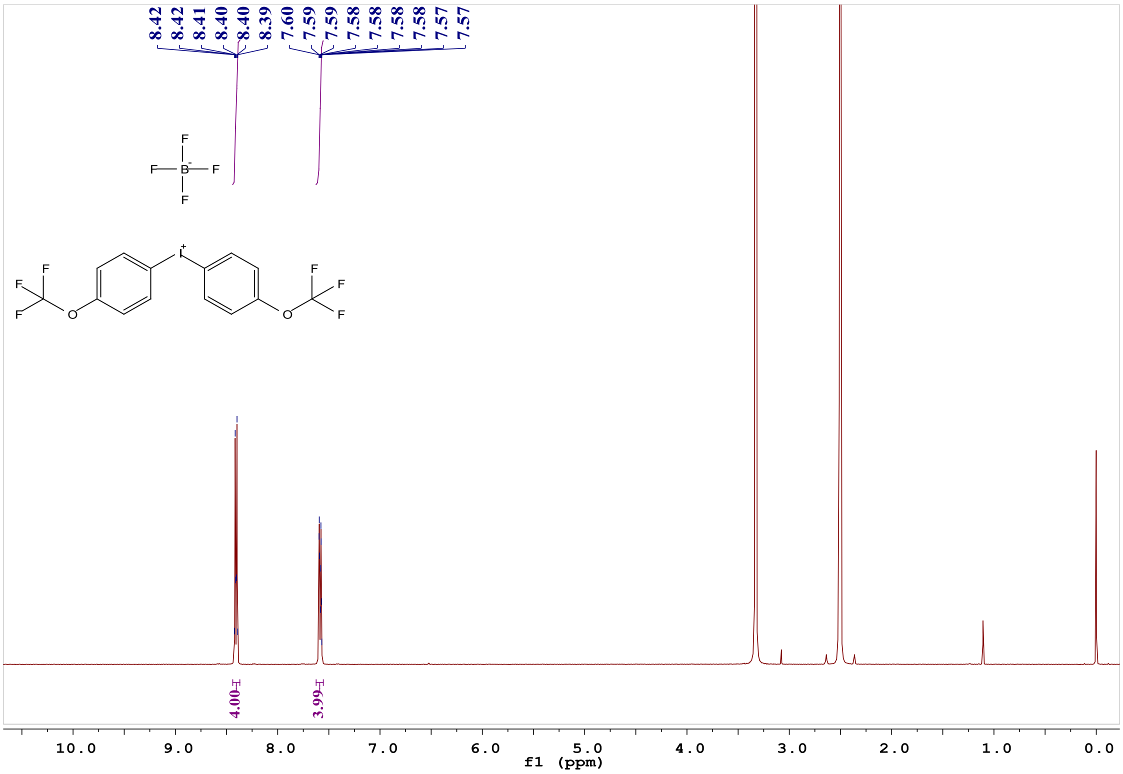

^{1}H NMR of DAI-j

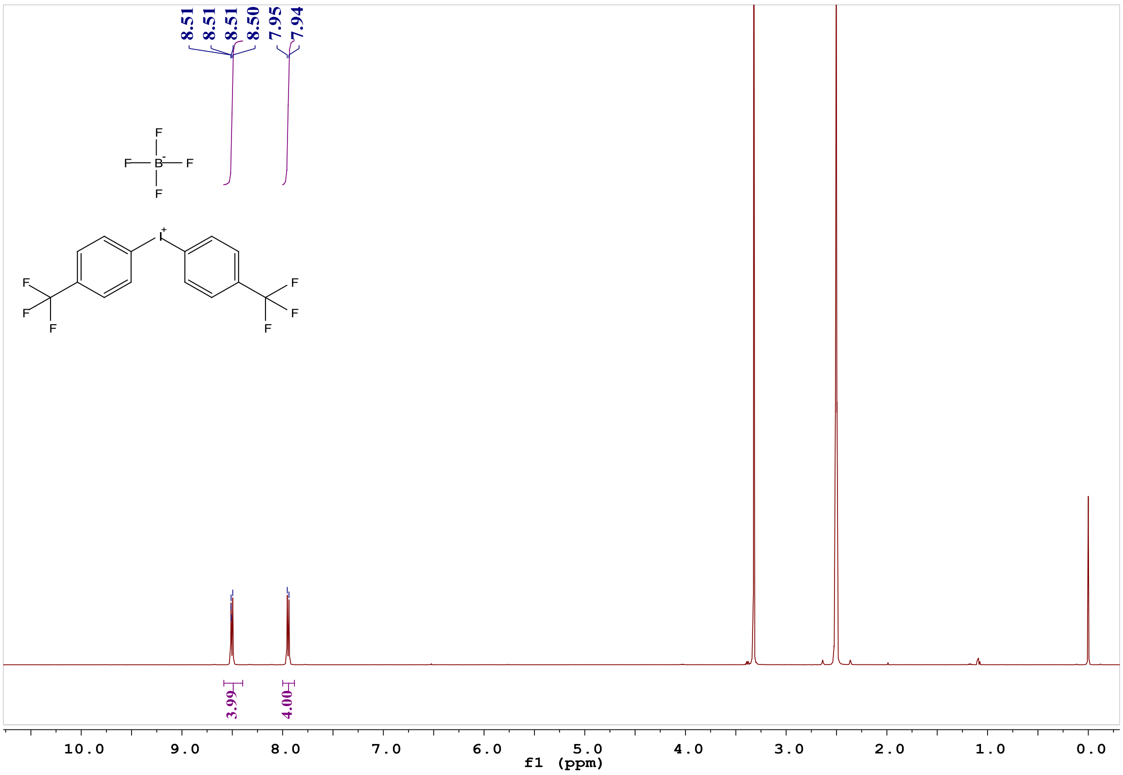

^{1}H NMR of DAI-k

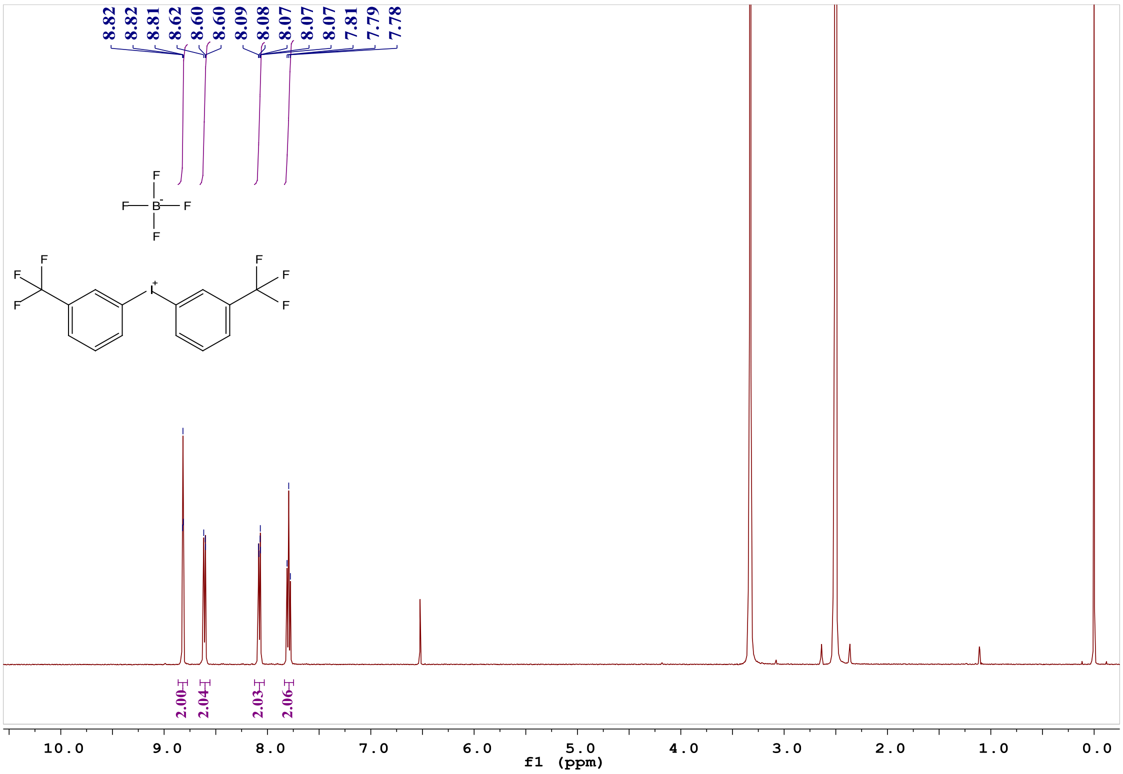

^{1}H NMR of DAI-l

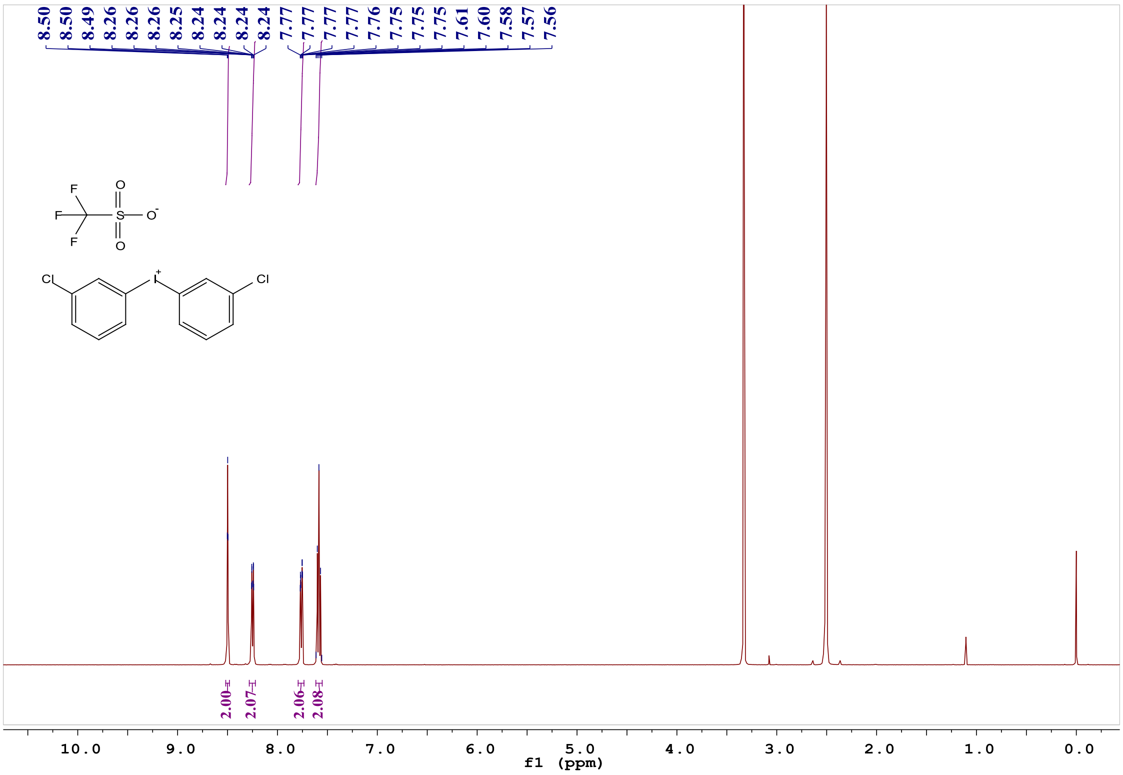

^{1}H NMR of DAI-m

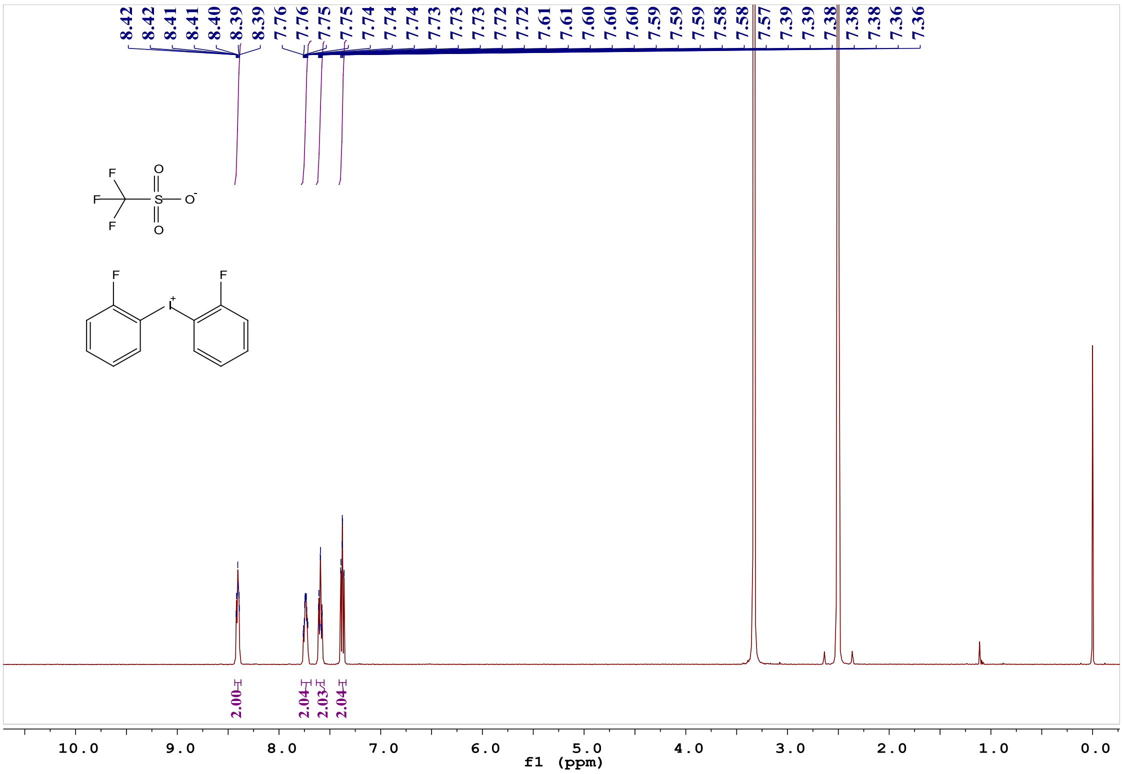

^{1}H NMR of DAI-n

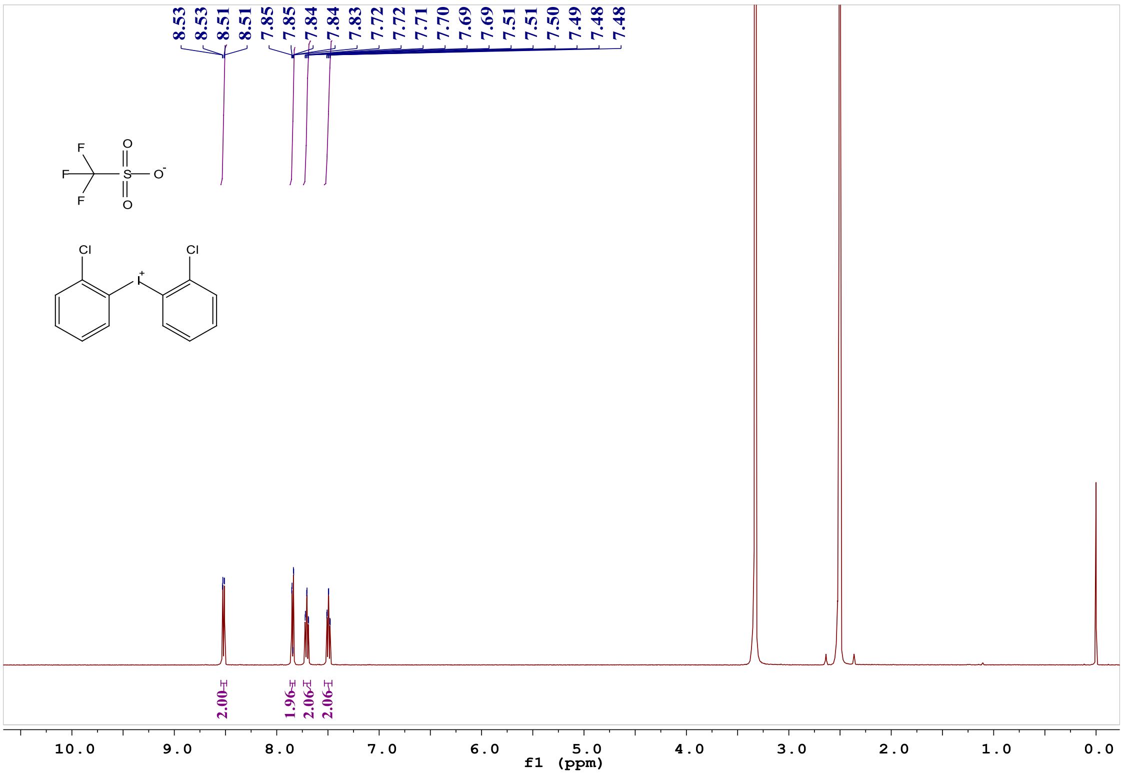

^{13}C NMR of DAI-n

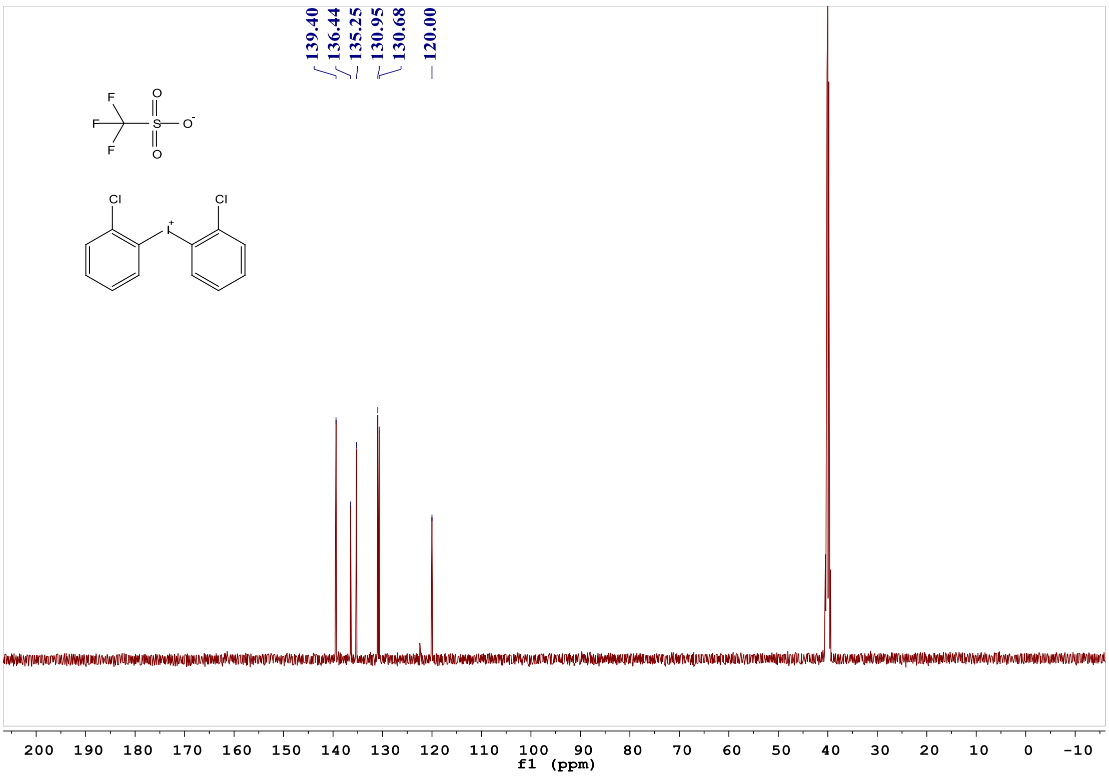

^{1}H NMR of DAI-o

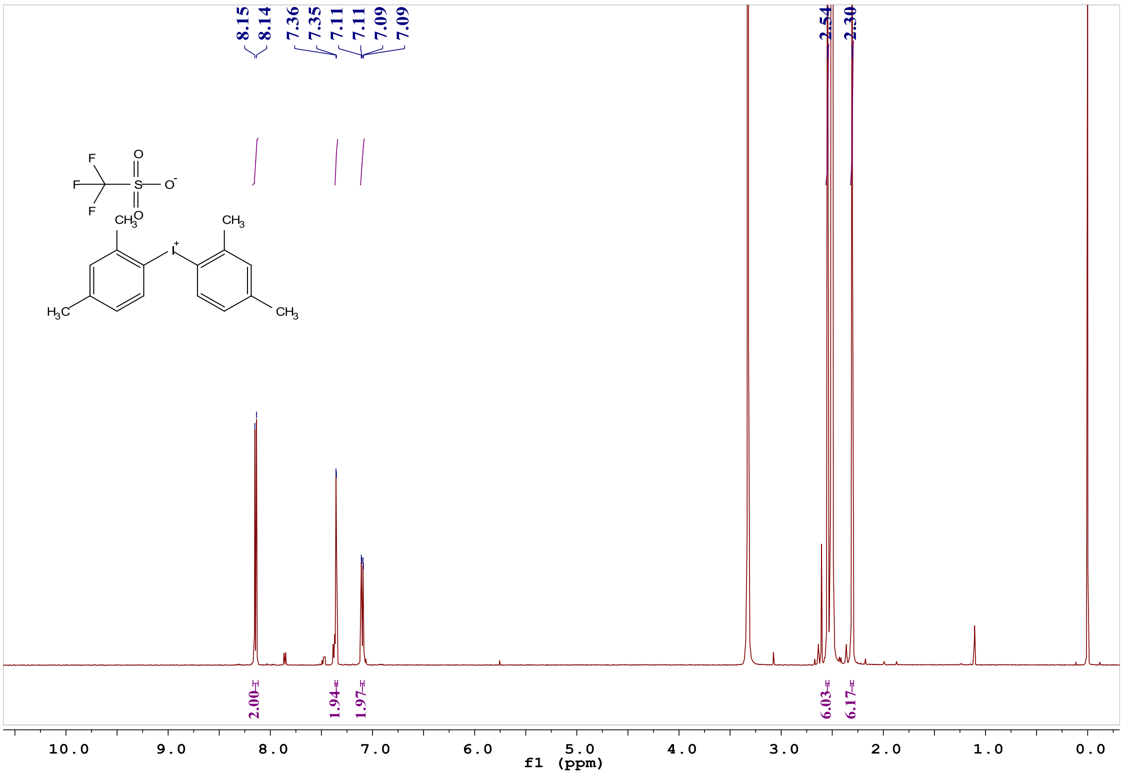

^{1}H NMR of DAI-p

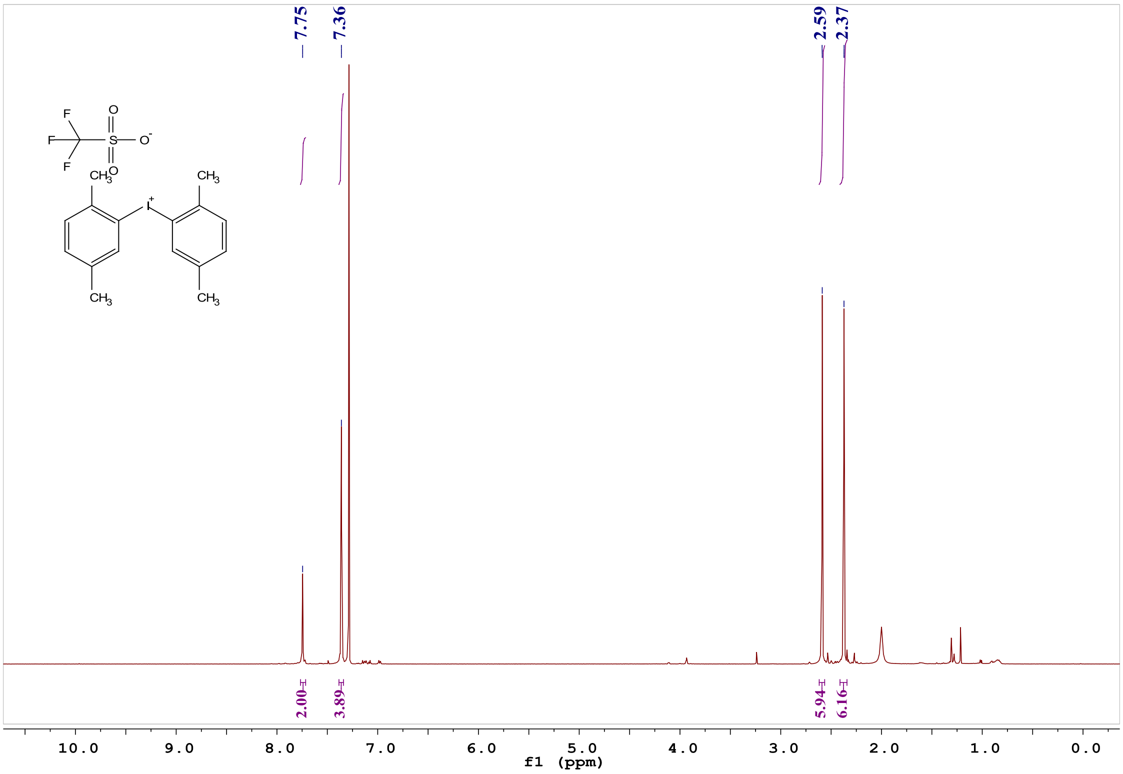

^{1}H NMR of DAI-q

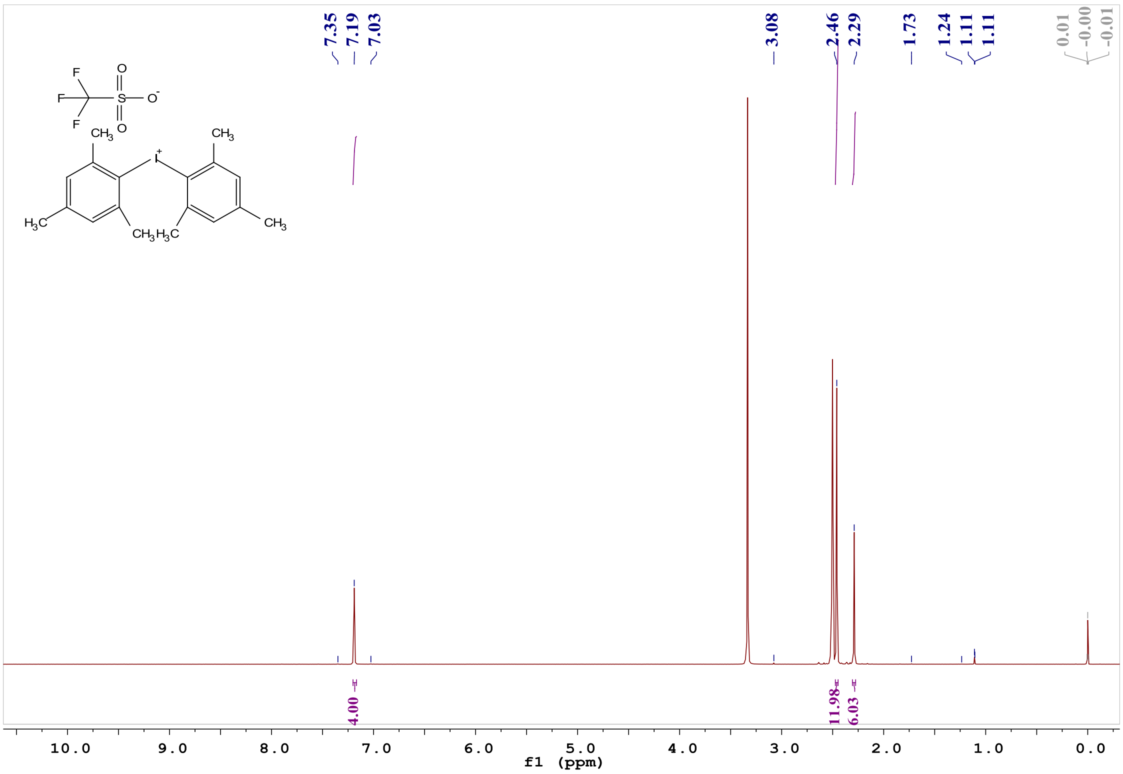

^{1}H NMR of DAI-r

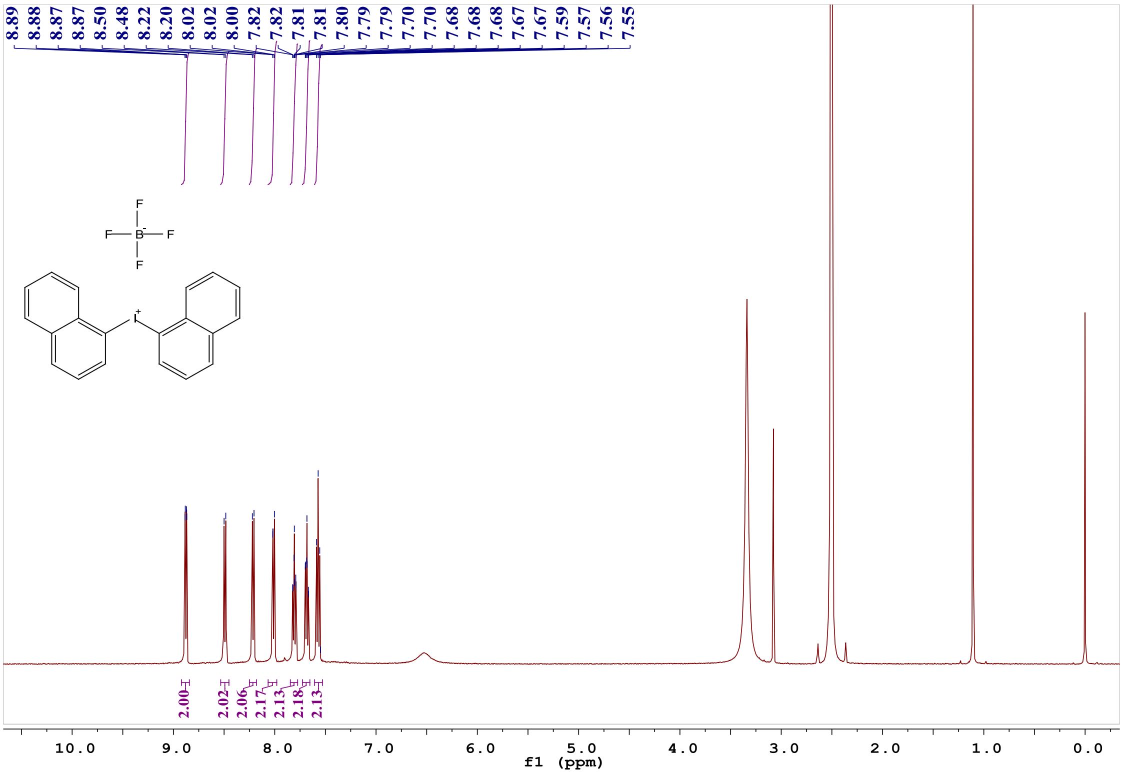

^{1}H NMR of DAI-s

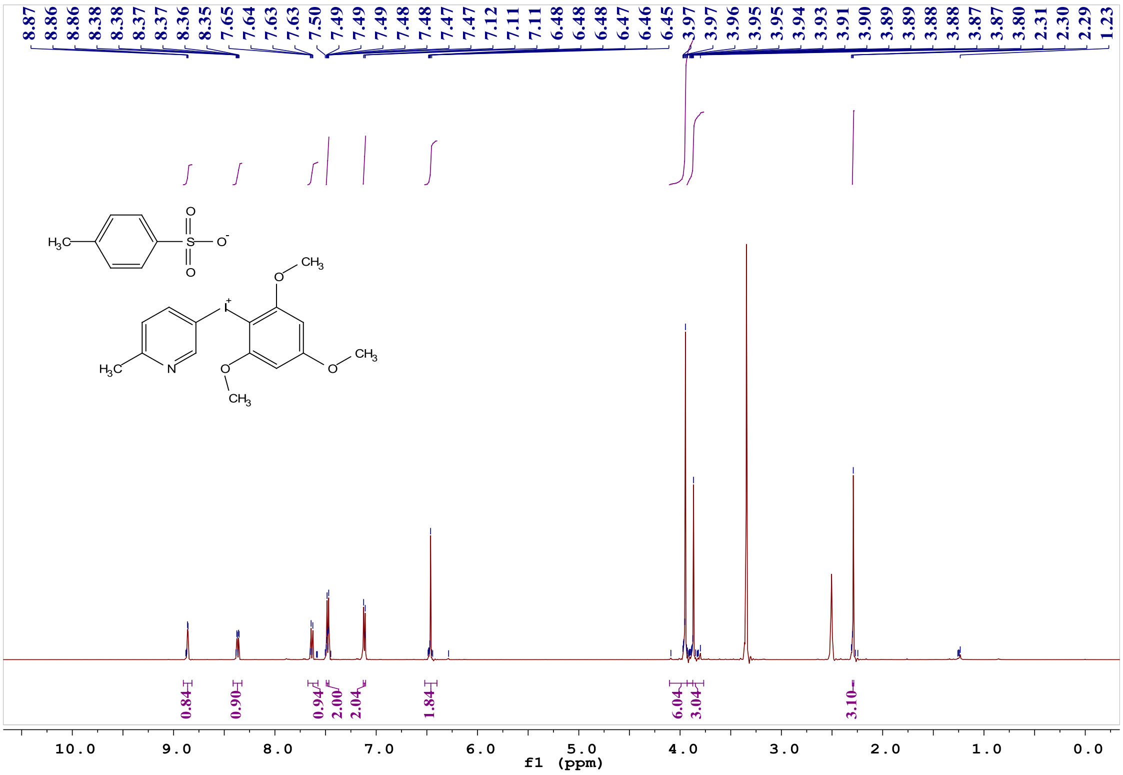

^{1}H NMR of DAI-t

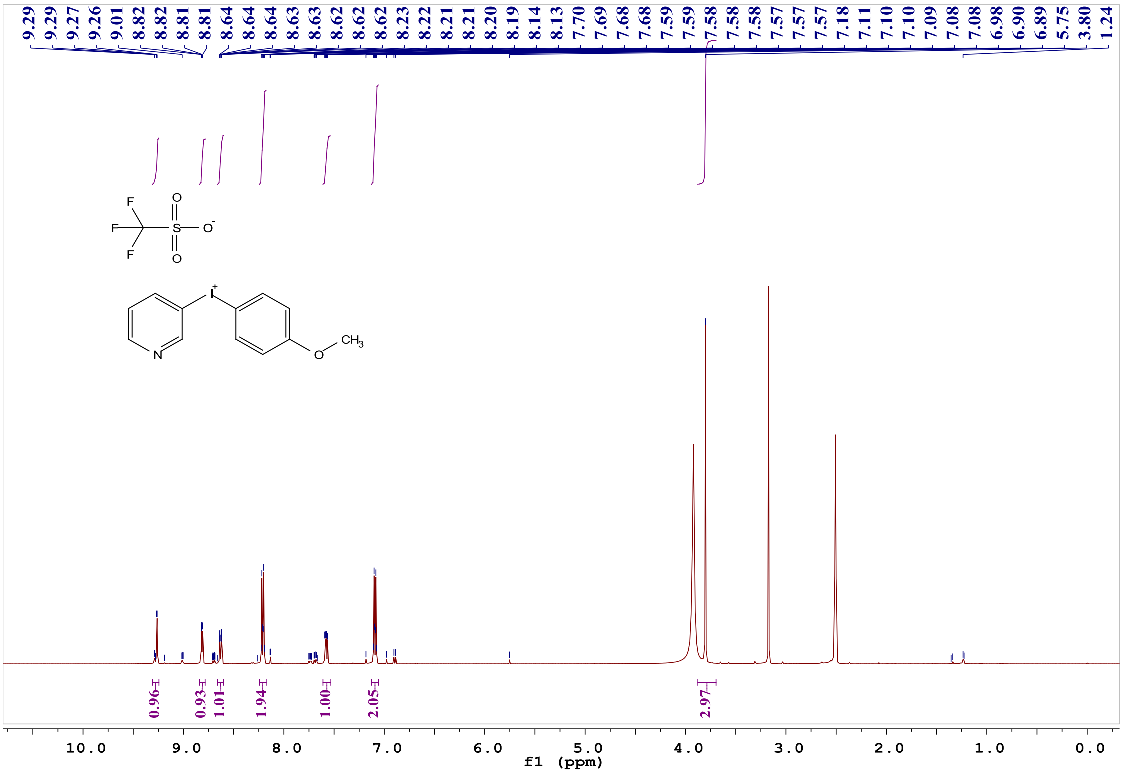

^{1}H NMR of is2-1

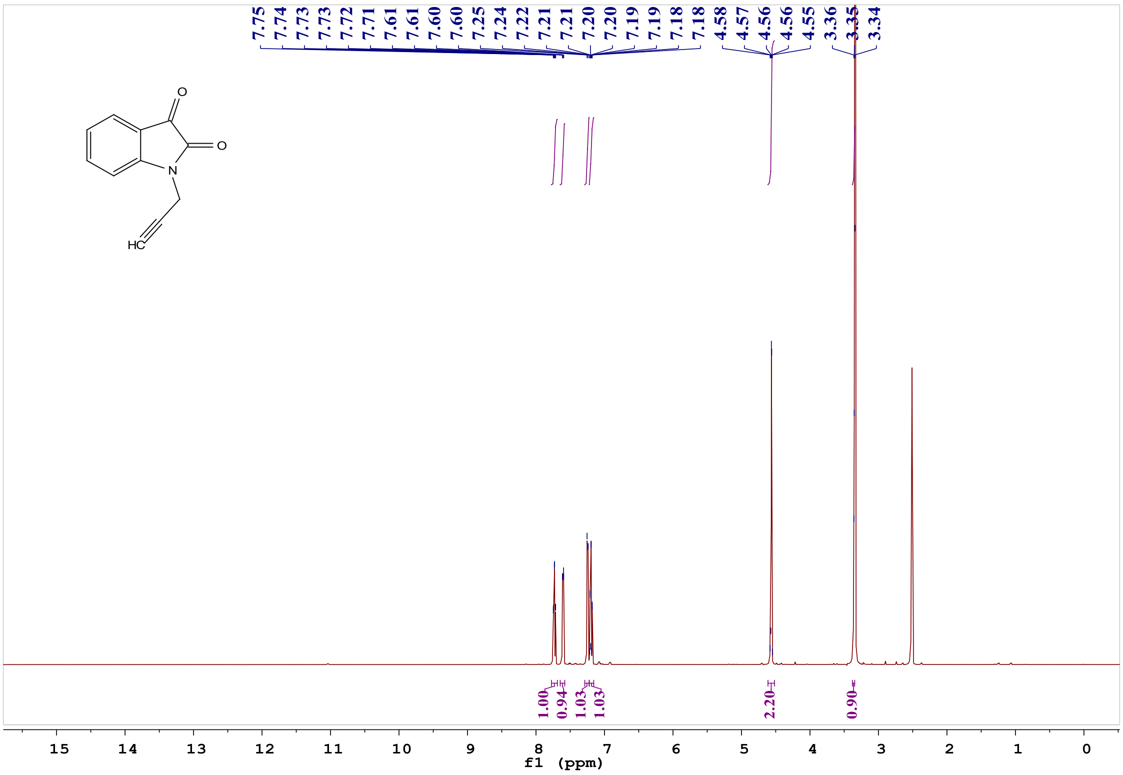

^{1}H NMR of is2-2

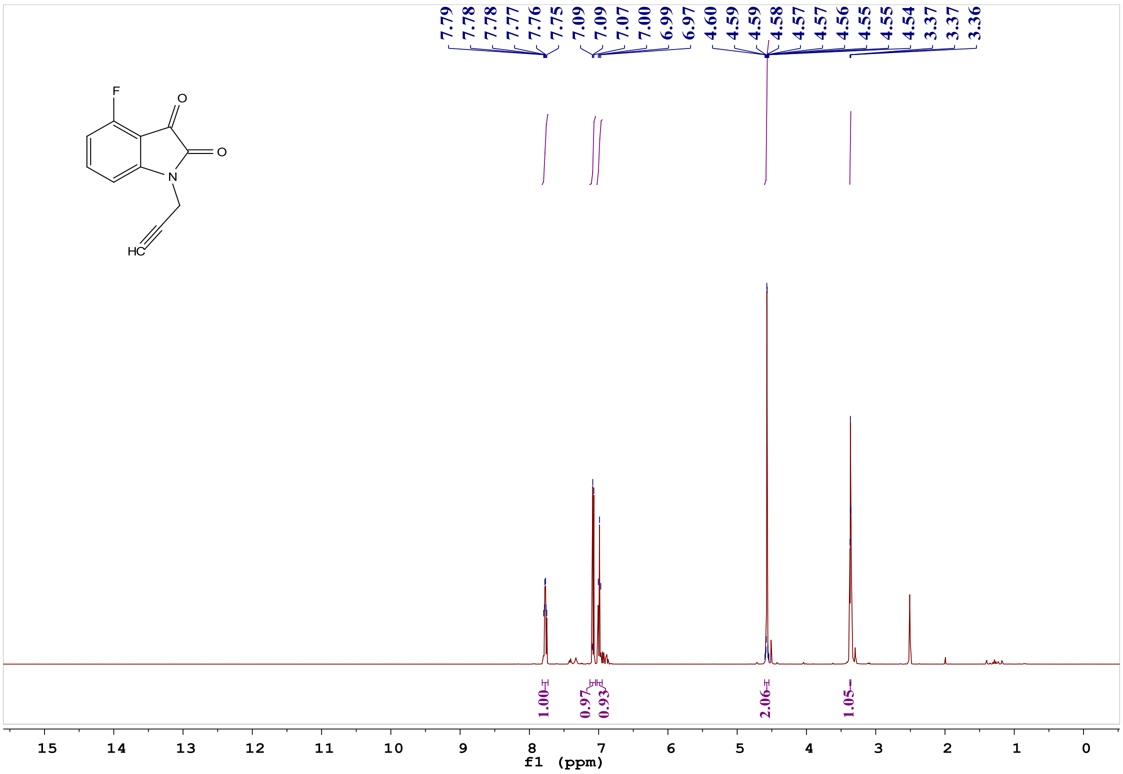

^{13}C NMR of is2-2

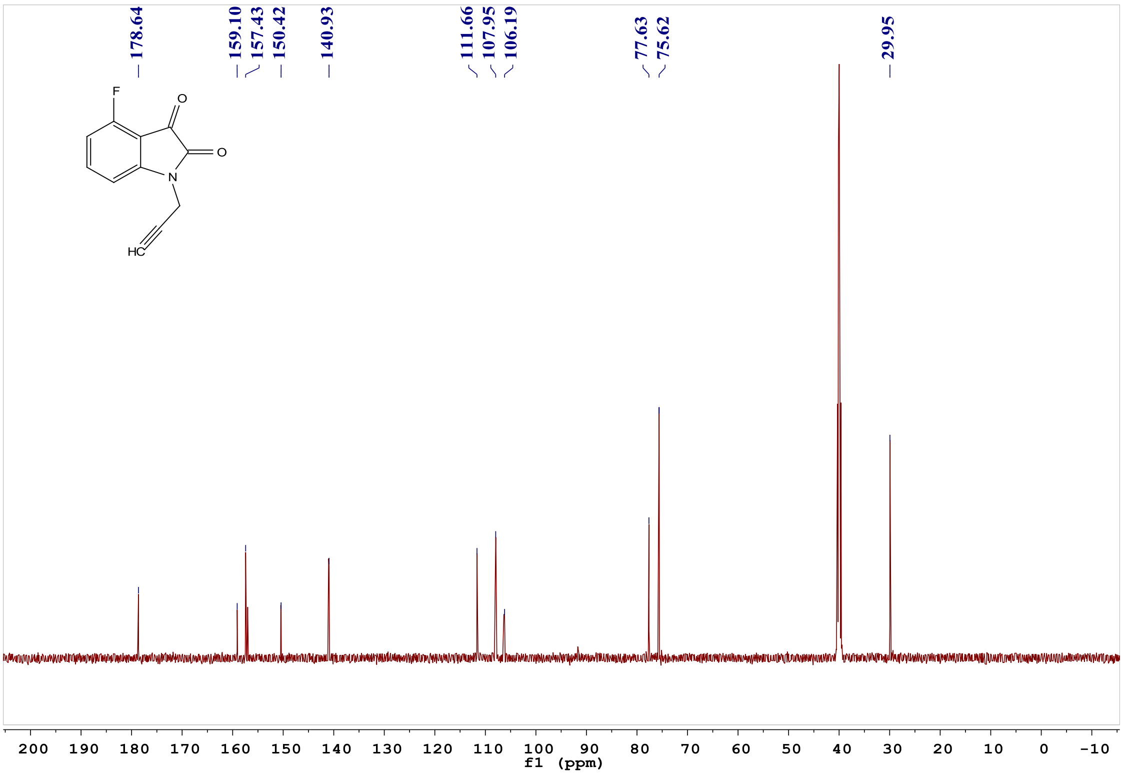

^{1}H NMR of is2-3

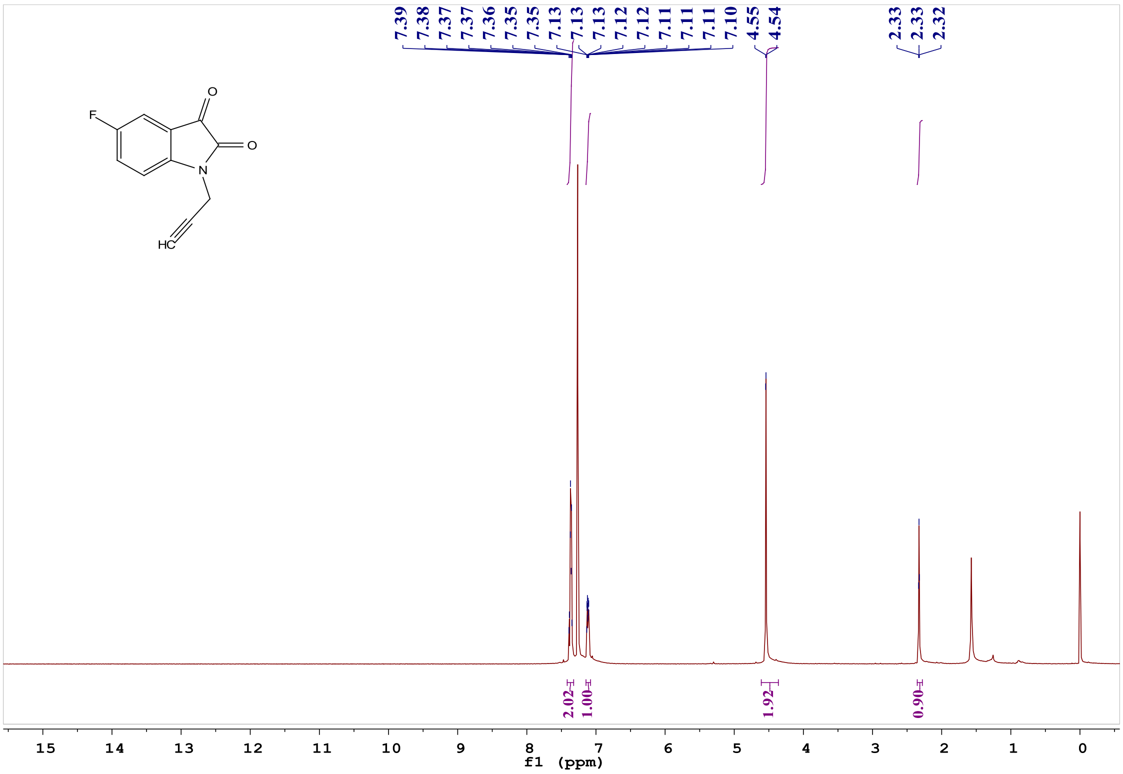

^{1}H NMR of is2-4

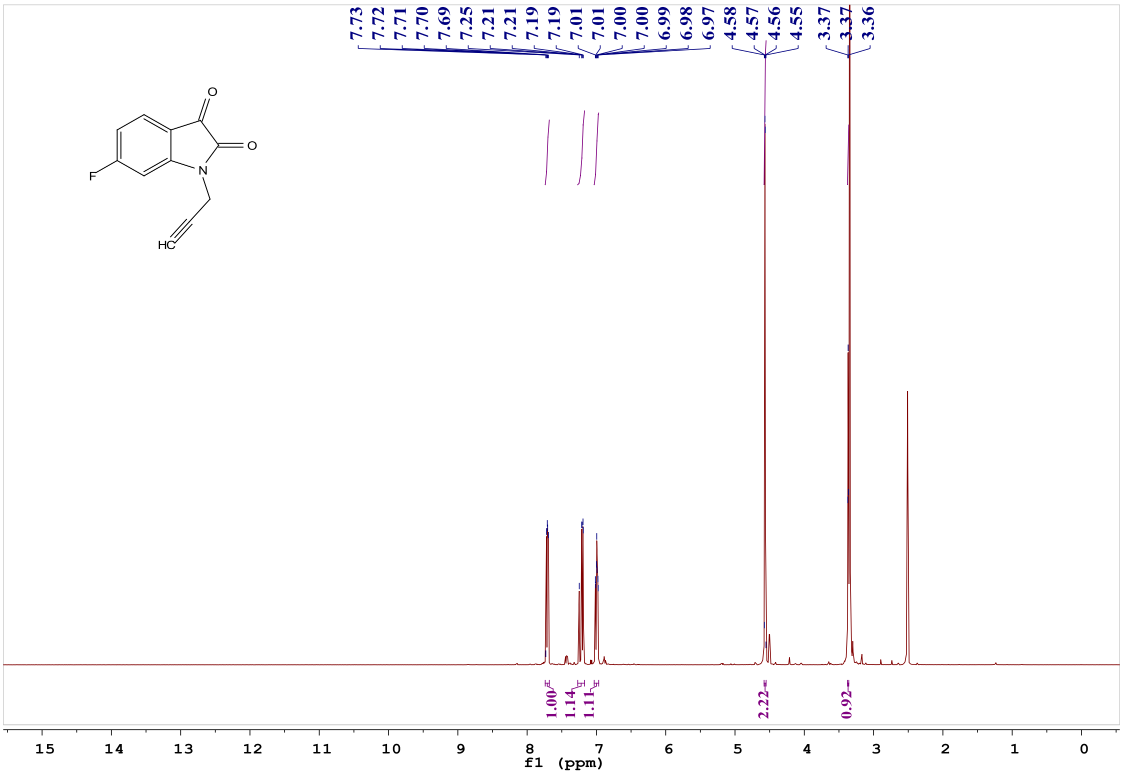

^{13}C NMR of is2-4

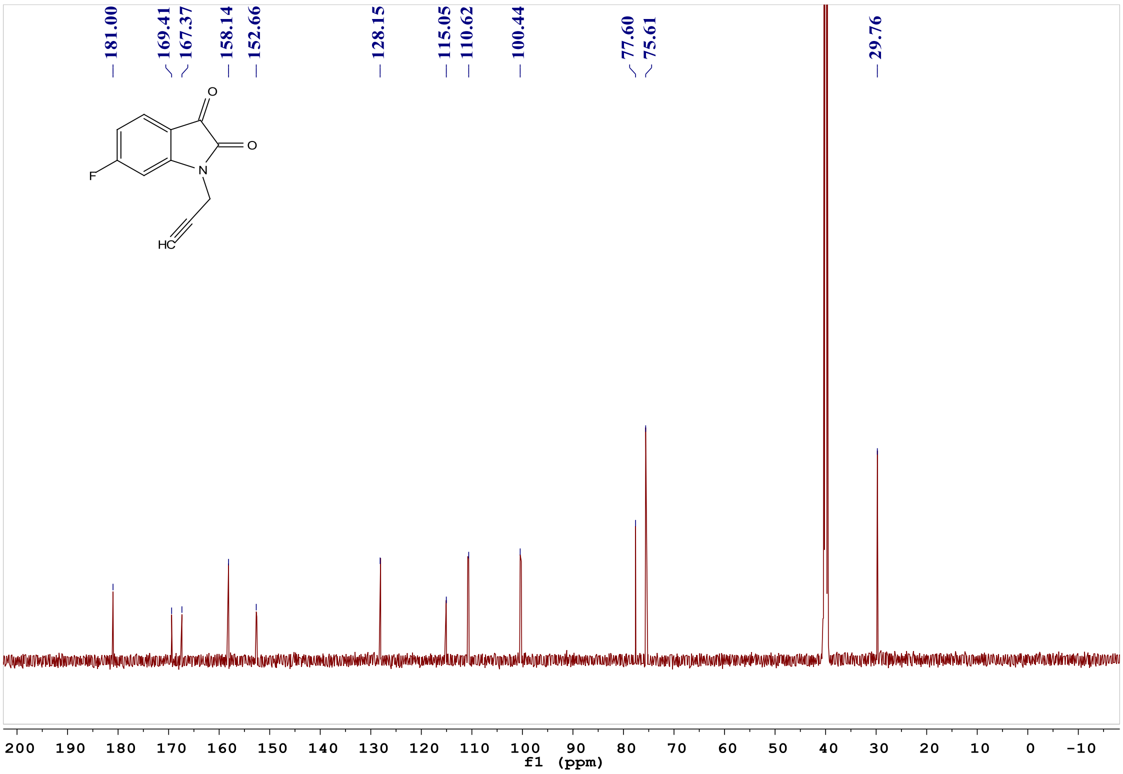

^{1}H NMR of is2-5

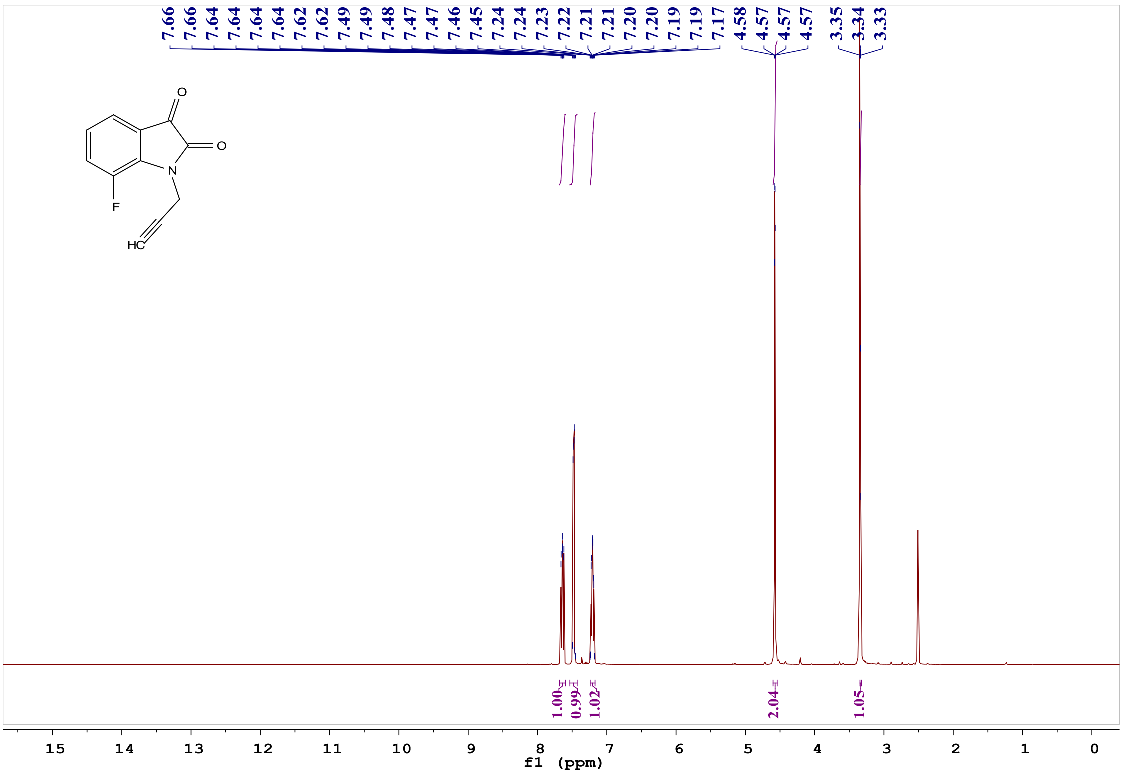

^{13}C NMR of is2-5

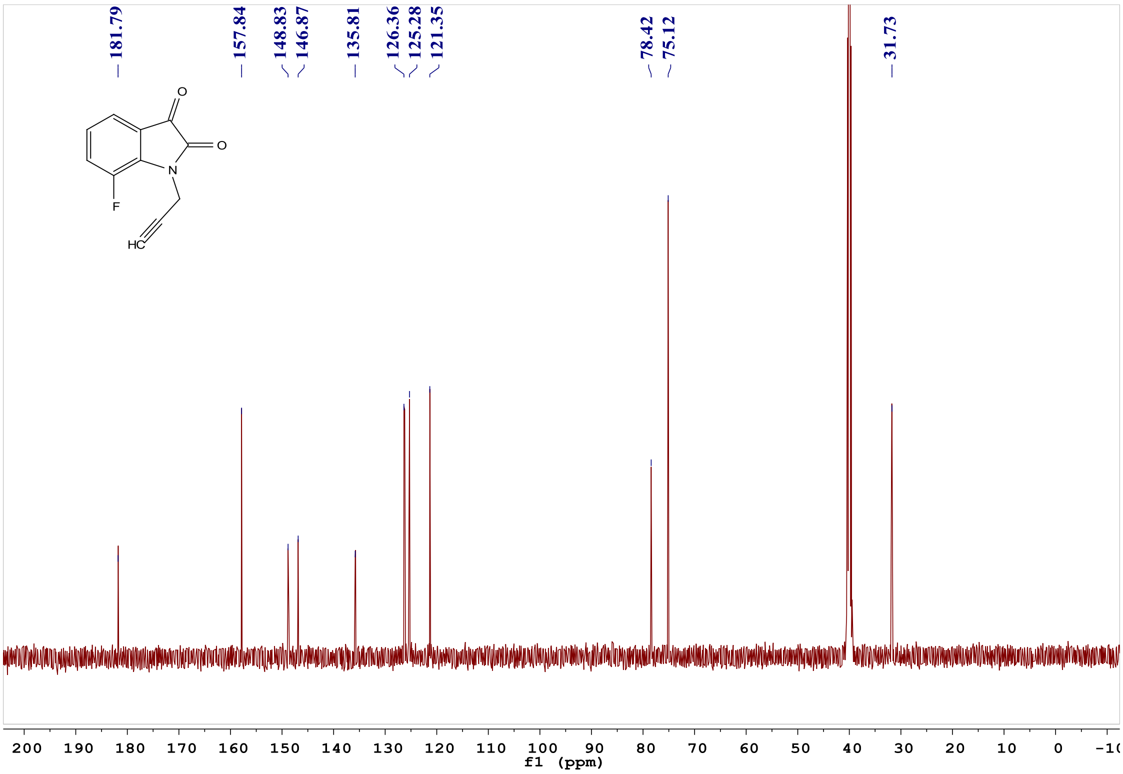

^{1}H NMR of is2-6

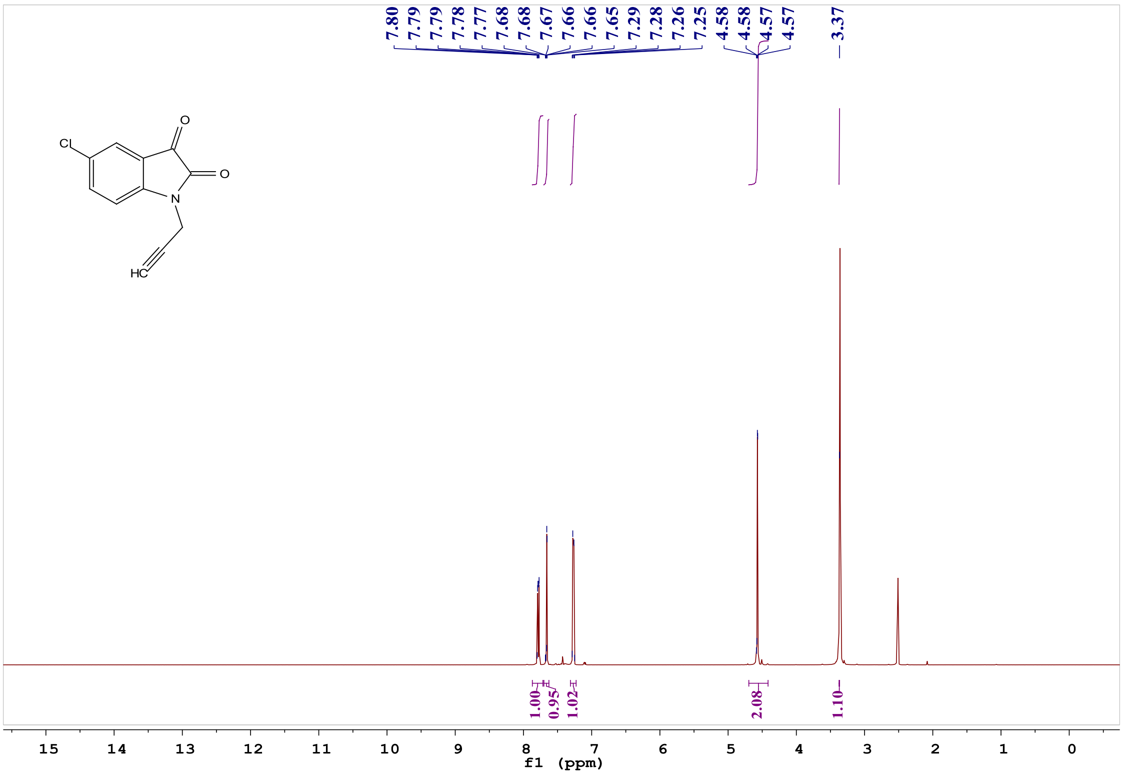

^{1}H NMR of is2-7

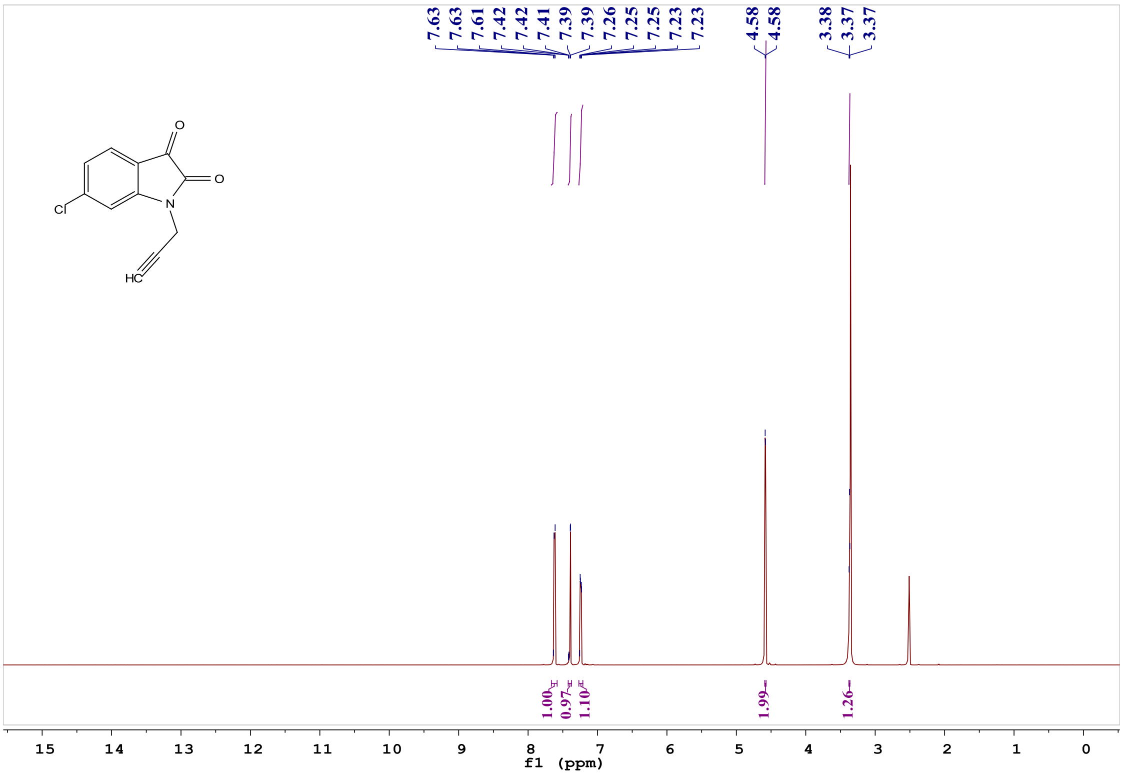

^{13}C NMR of is2-7

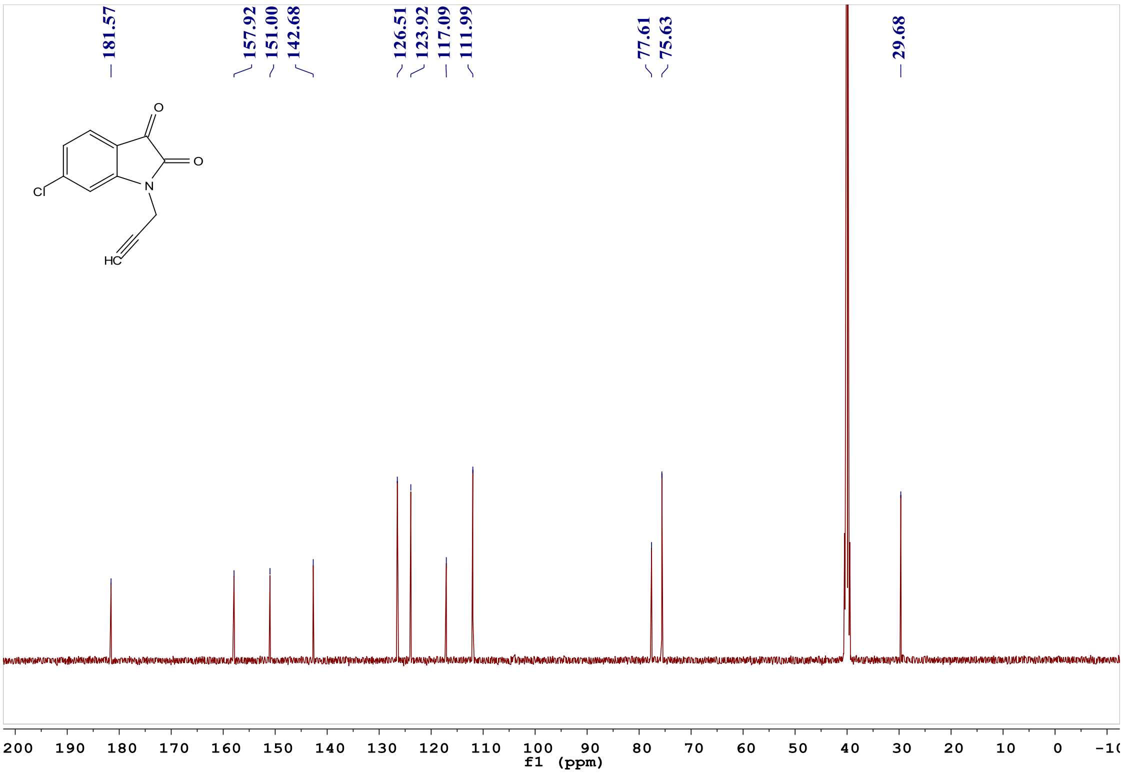

^{1}H NMR of is2-8

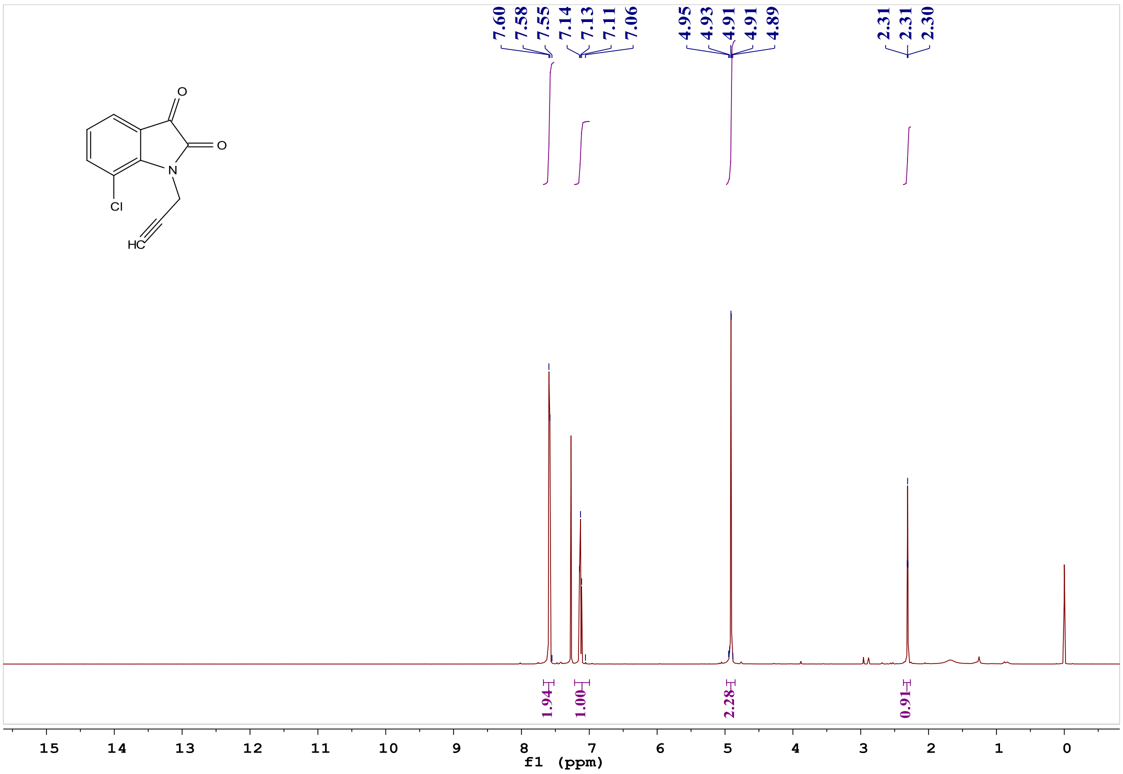

^{1}H NMR of is2-9

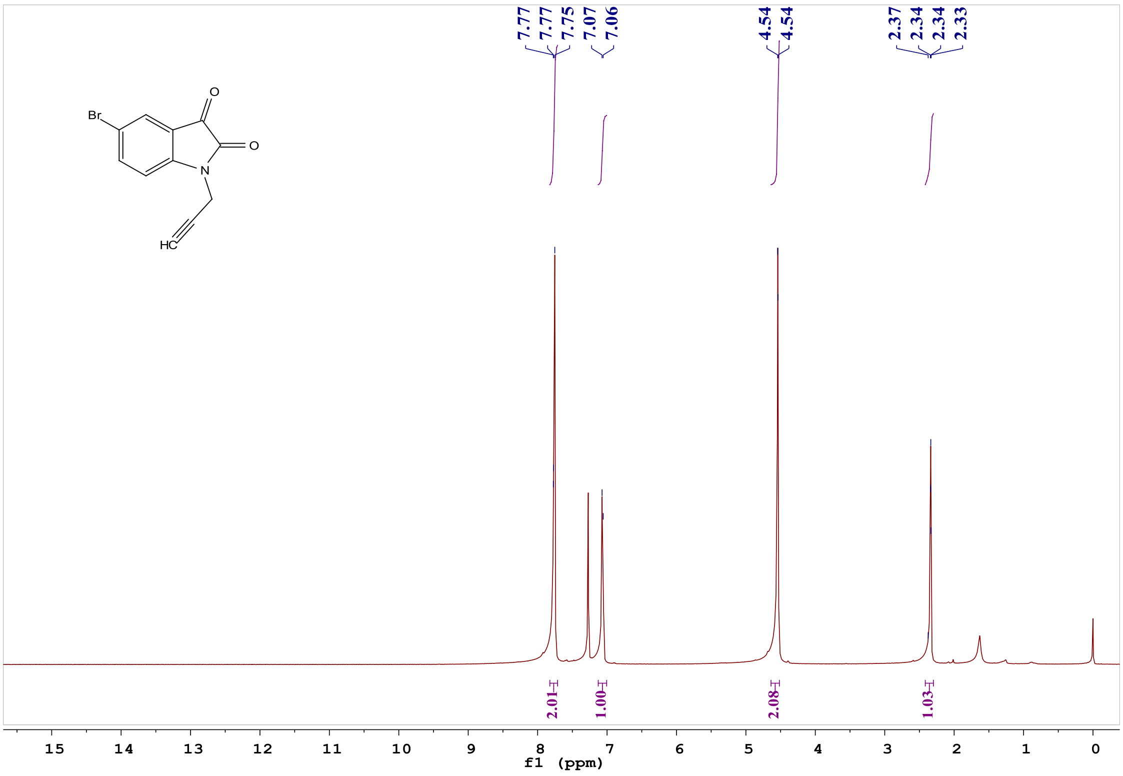

^{1}H NMR of is2-10

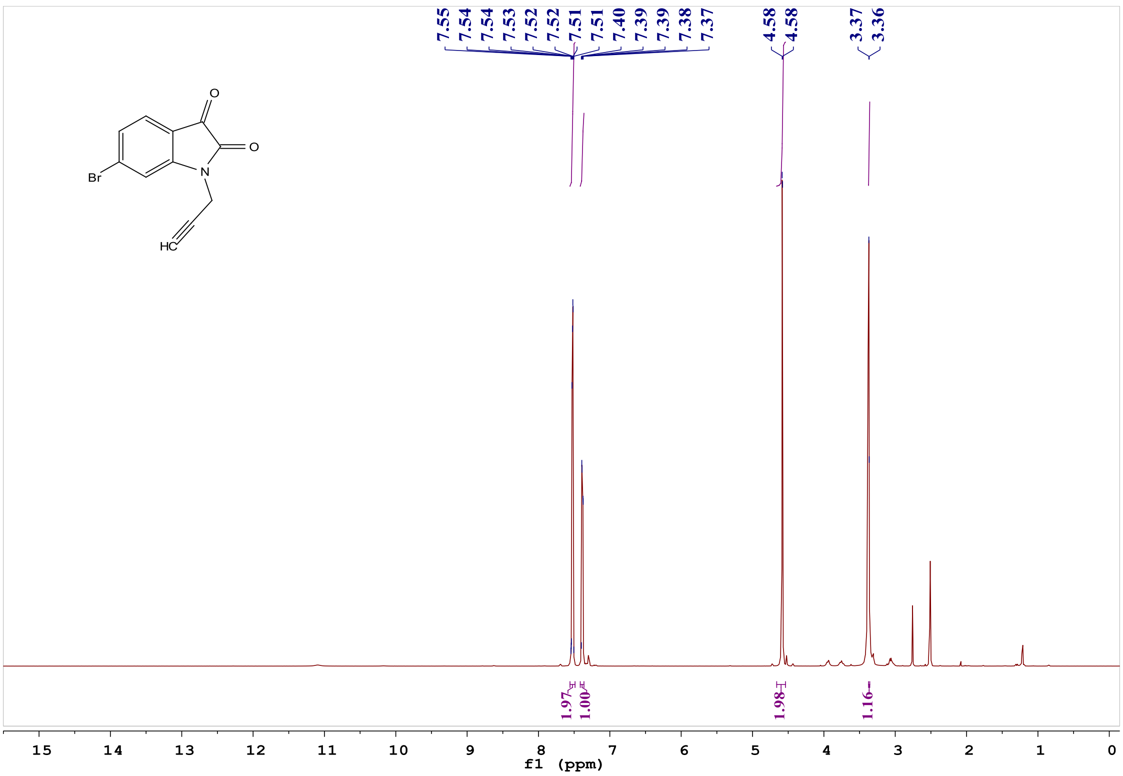

^{13}C NMR of is2-10

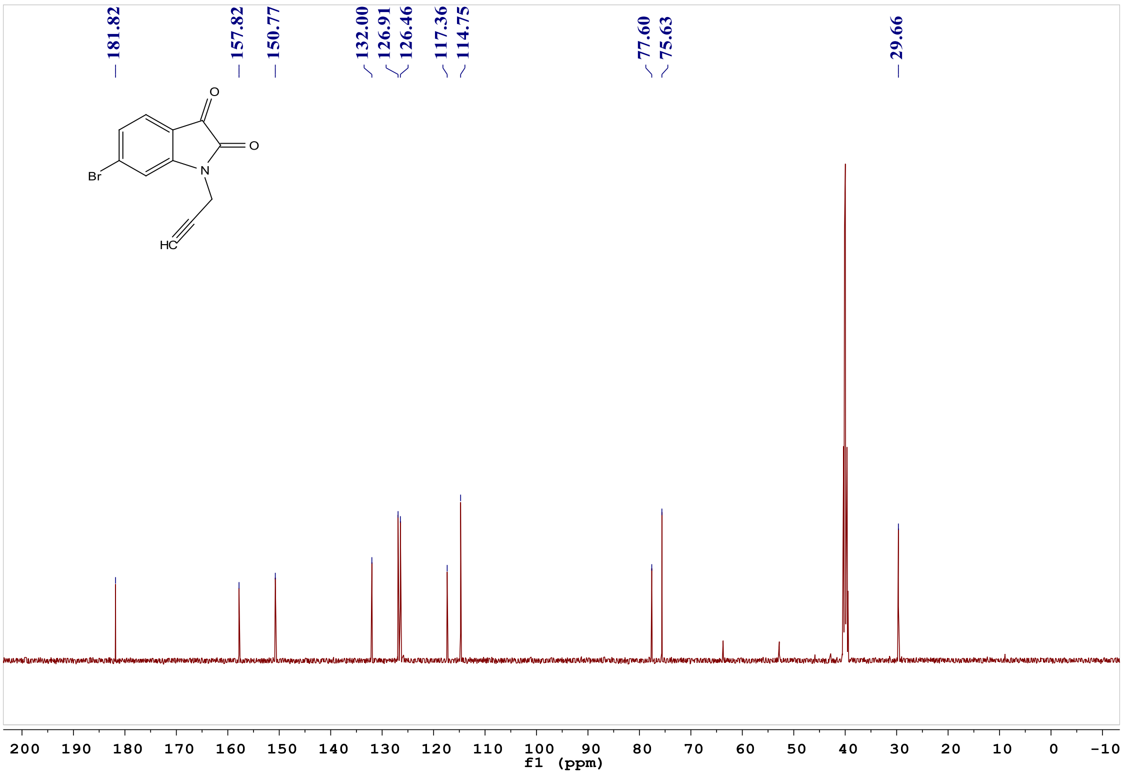

^{1}H NMR of is2-11

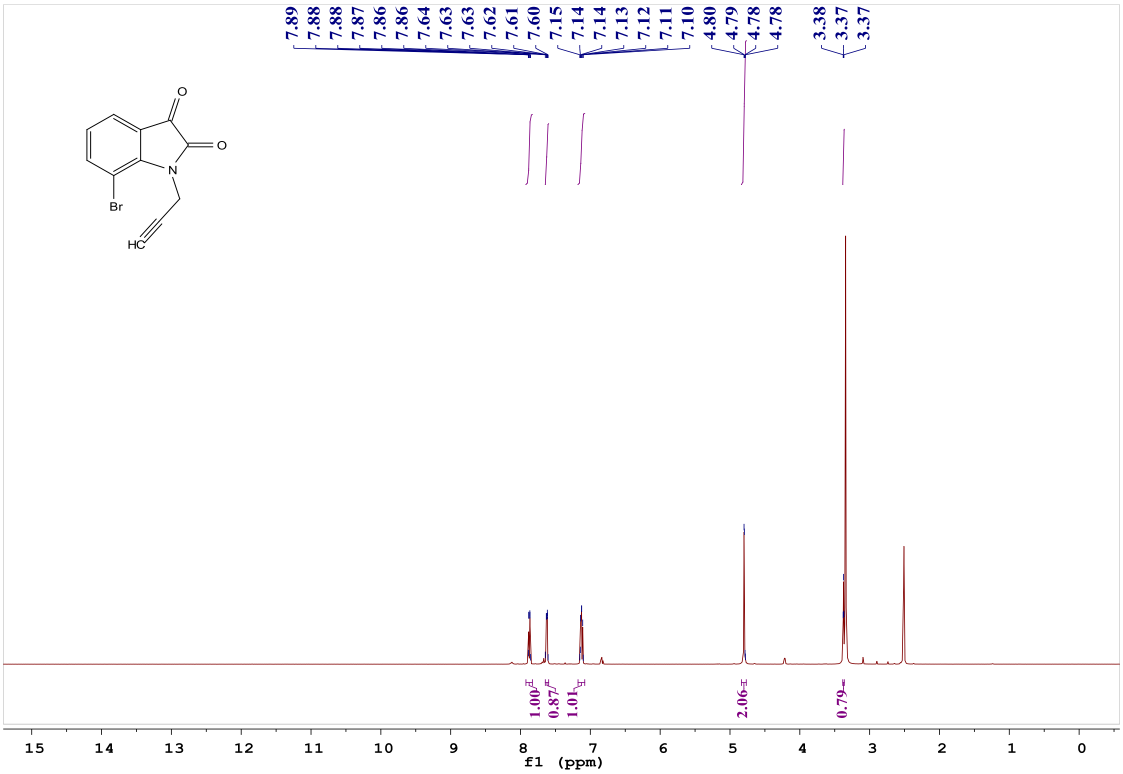

^{13}C NMR of is2-11

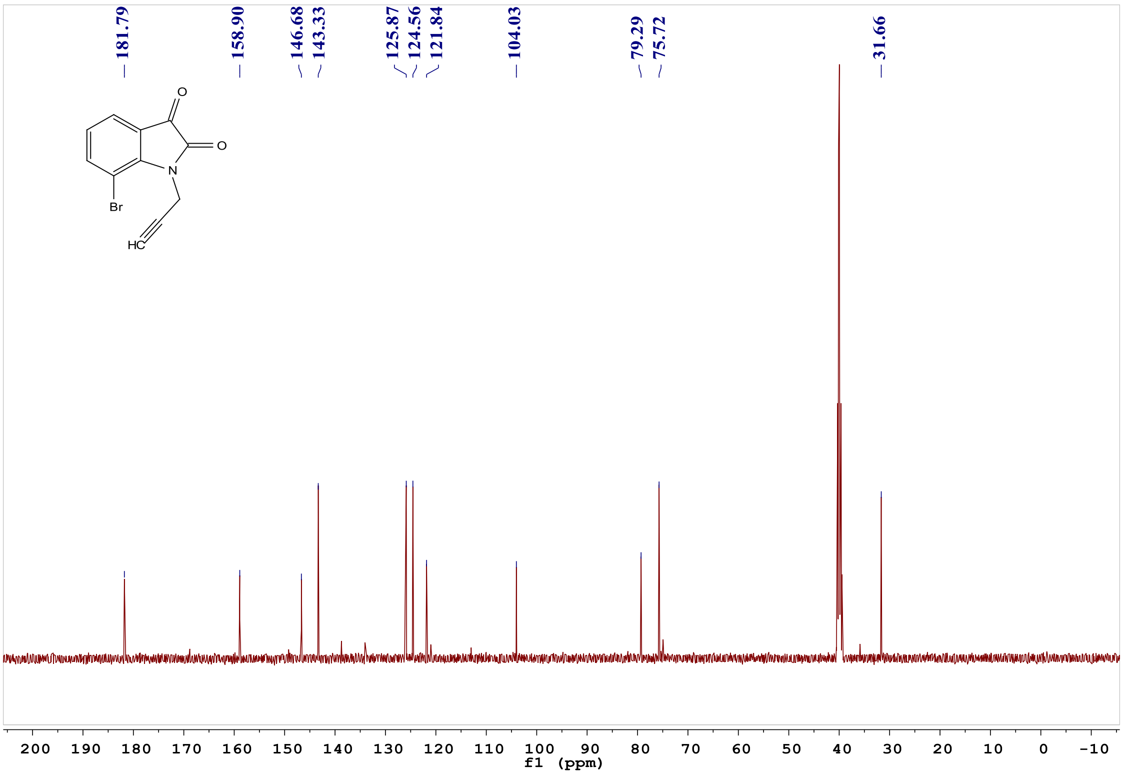

^{1}H NMR of is2-12

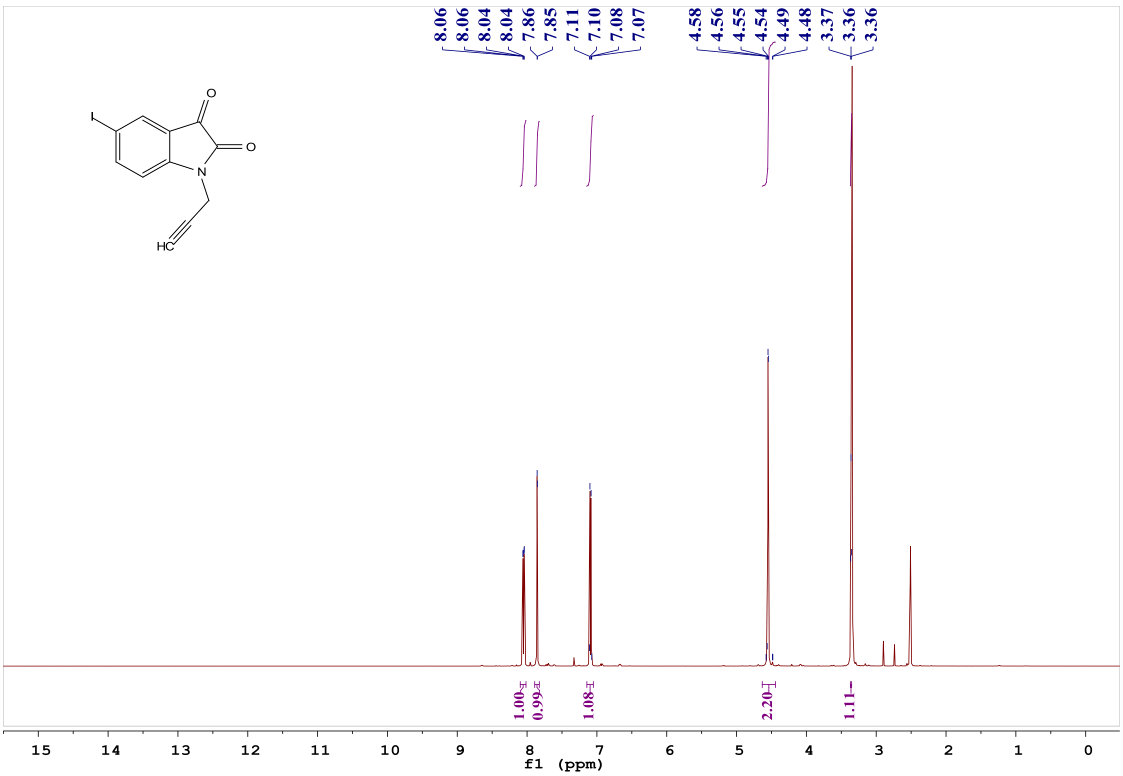

^{13}C NMR of is2-12

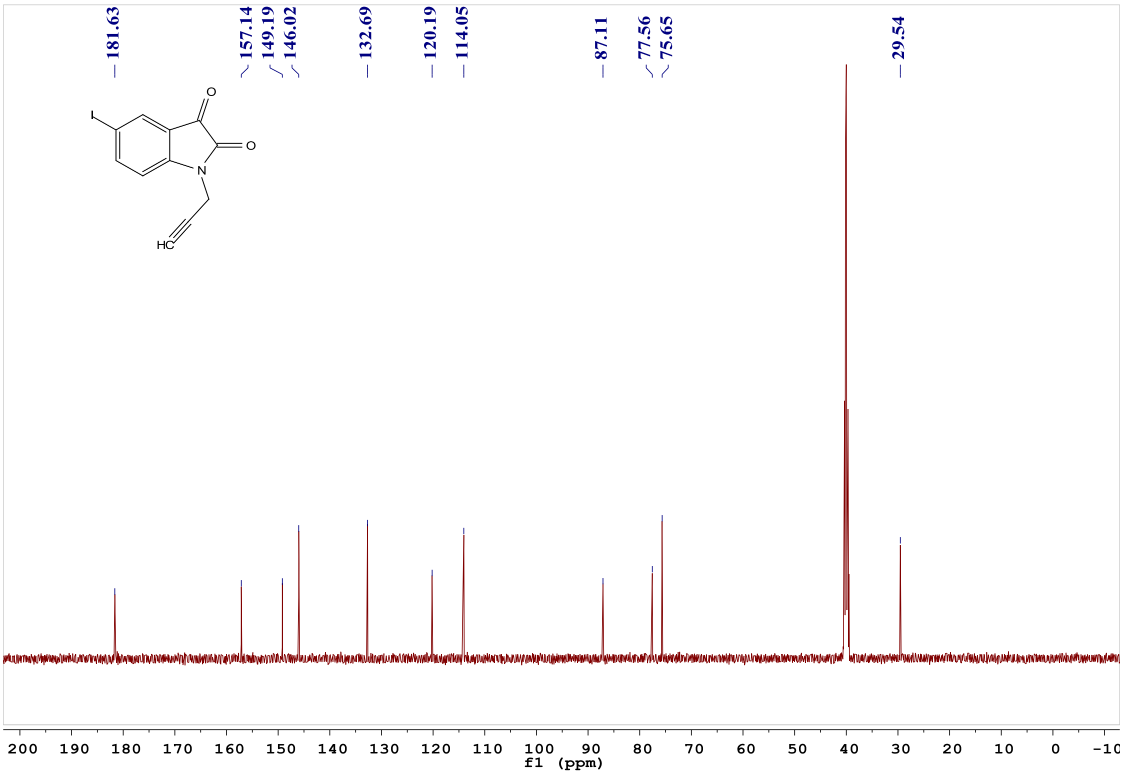

^{1}H NMR of is2-13

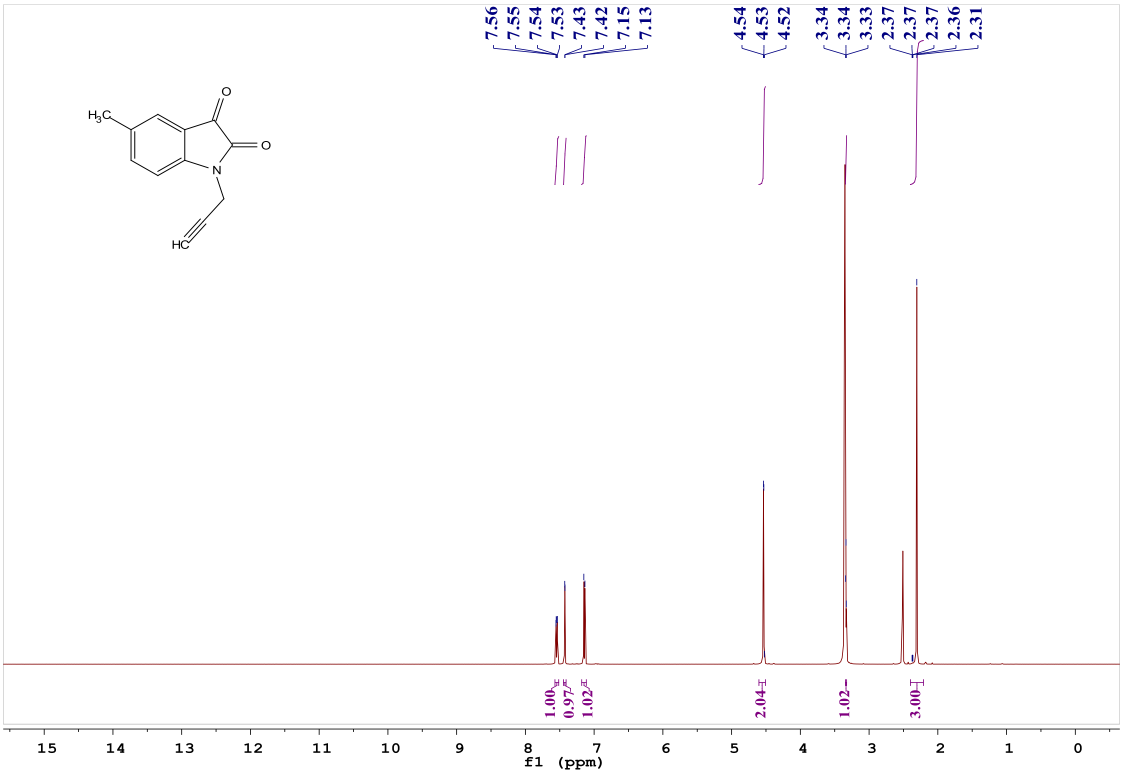

^{1}H NMR of is2-14

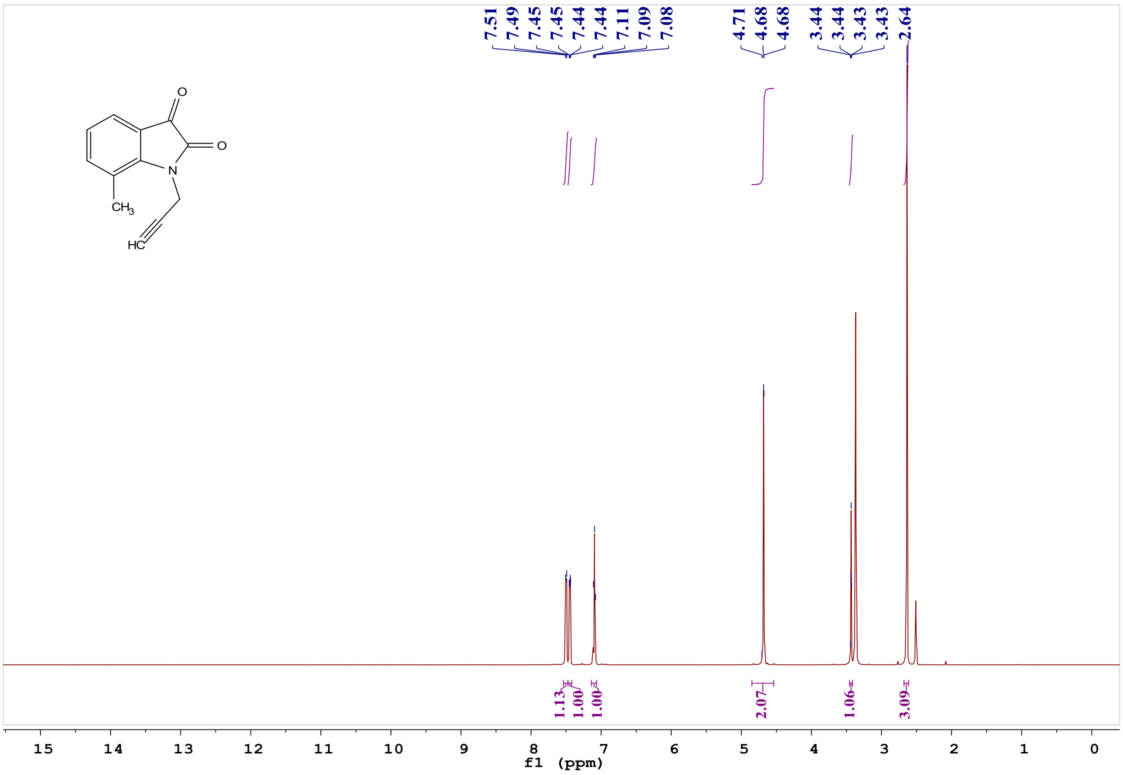

^{13}C NMR of is2-14

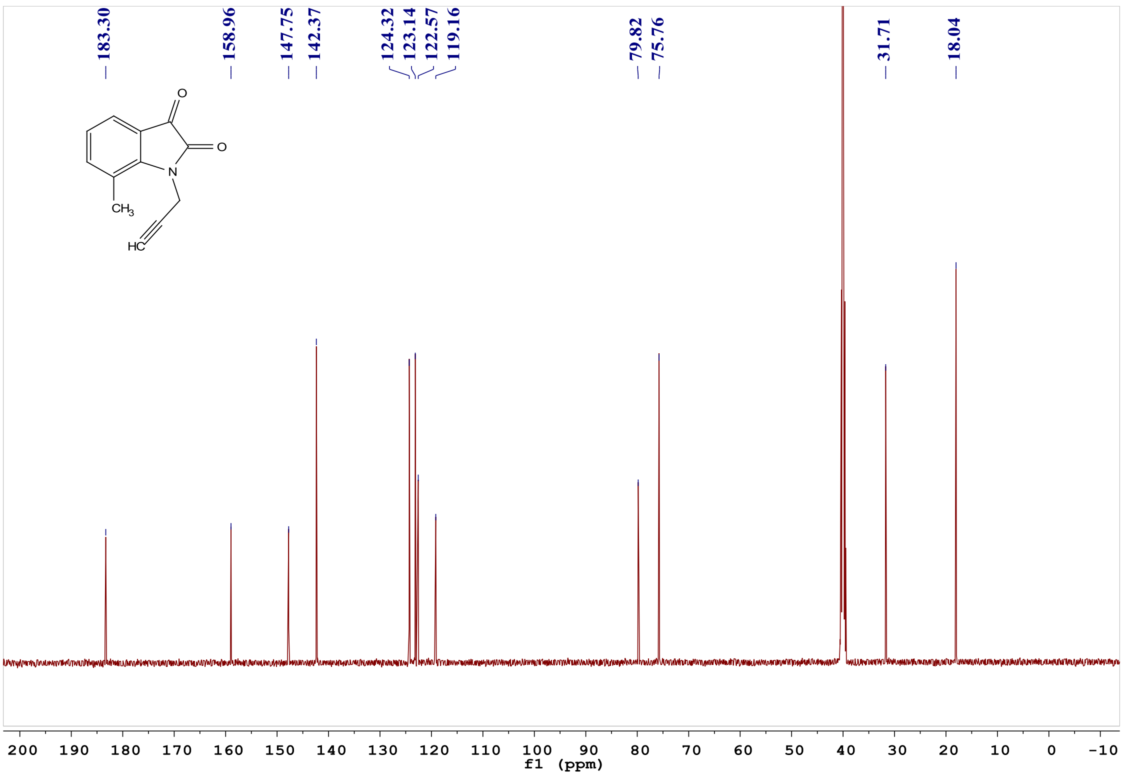

^{1}H NMR of is2-15

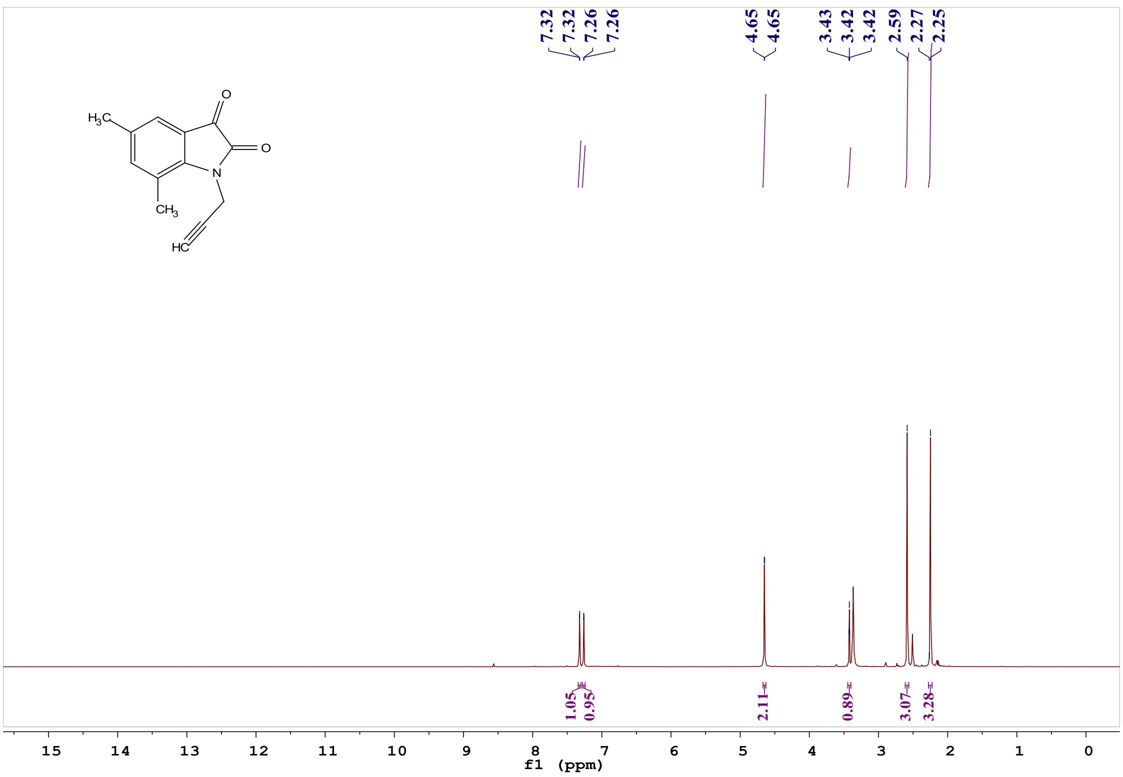

^{13}C NMR of is2-15

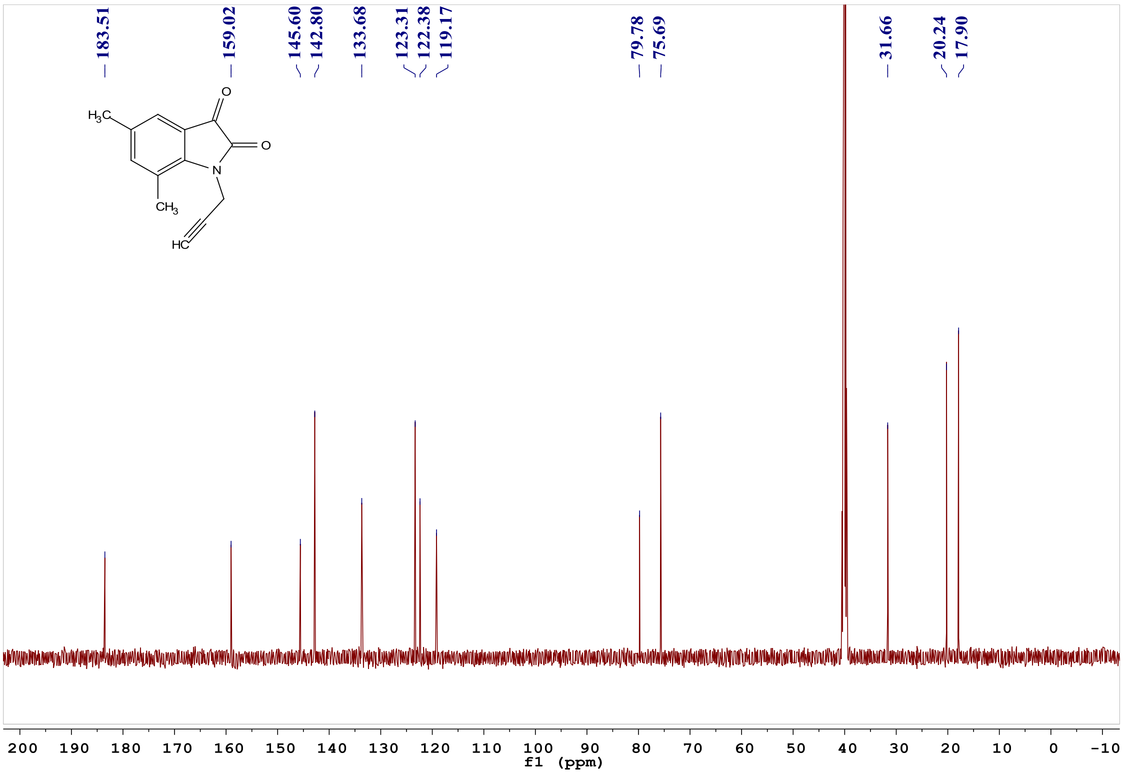

^{1}H NMR of is2-16

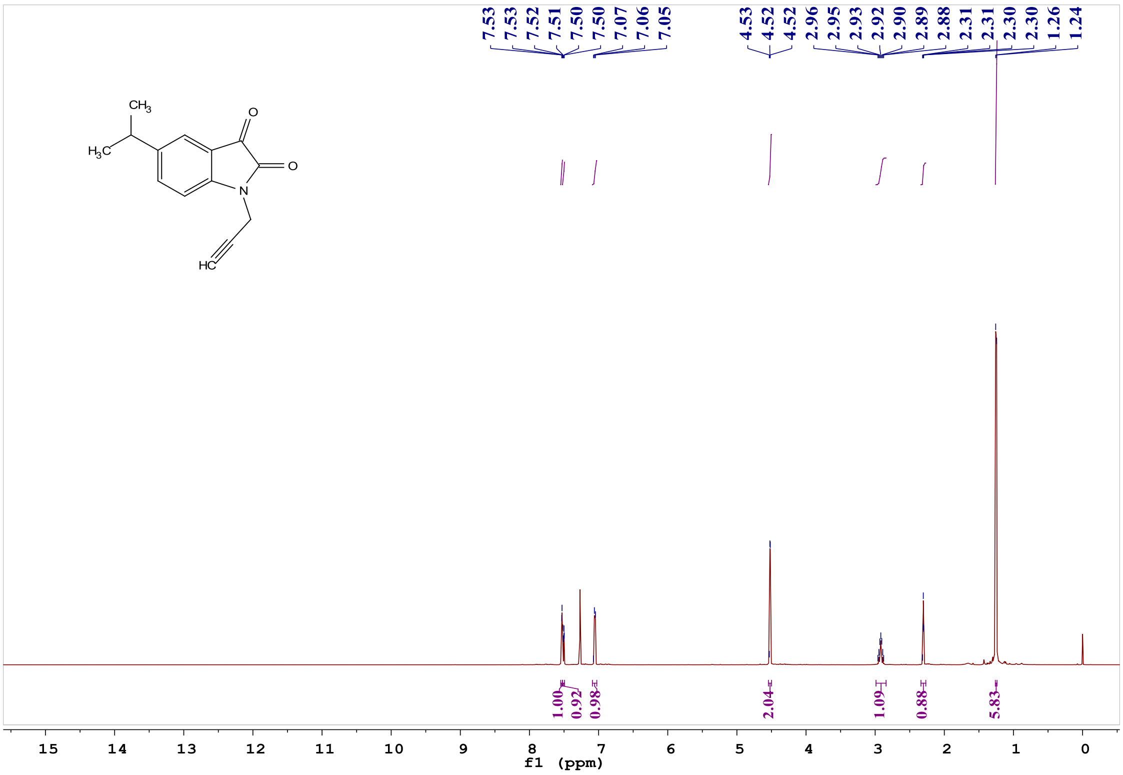

^{13}C NMR of is2-16

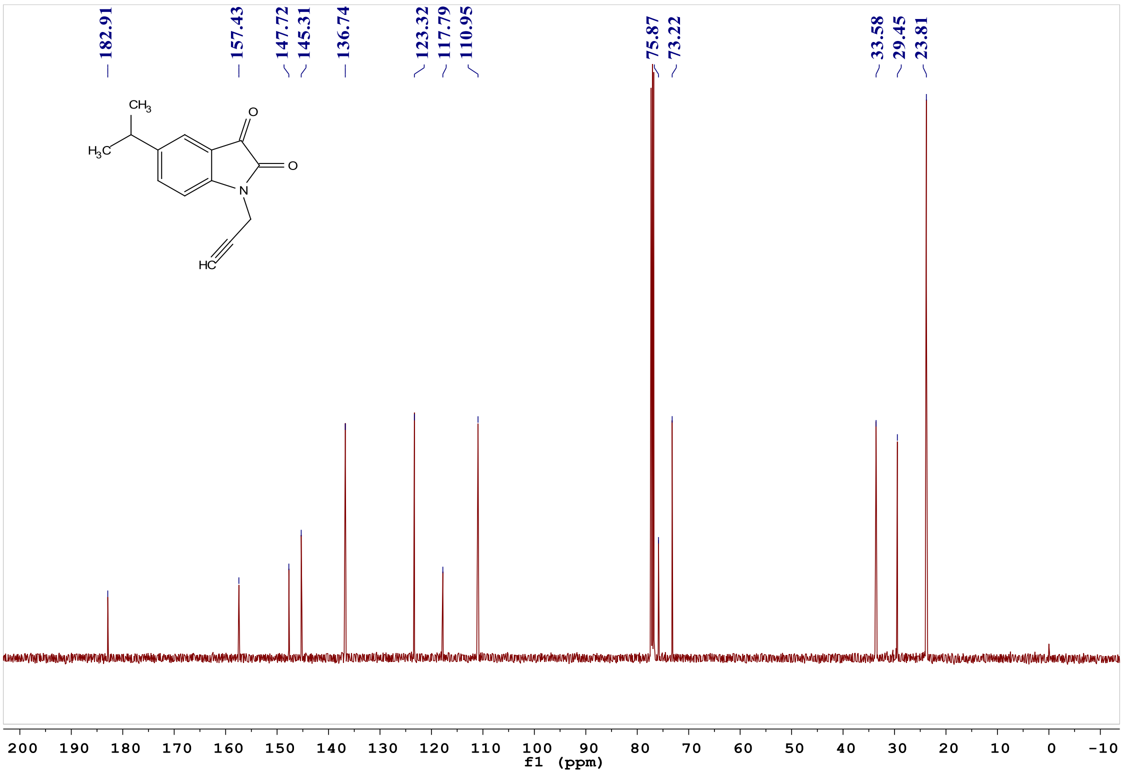

^{1}H NMR of is2-17

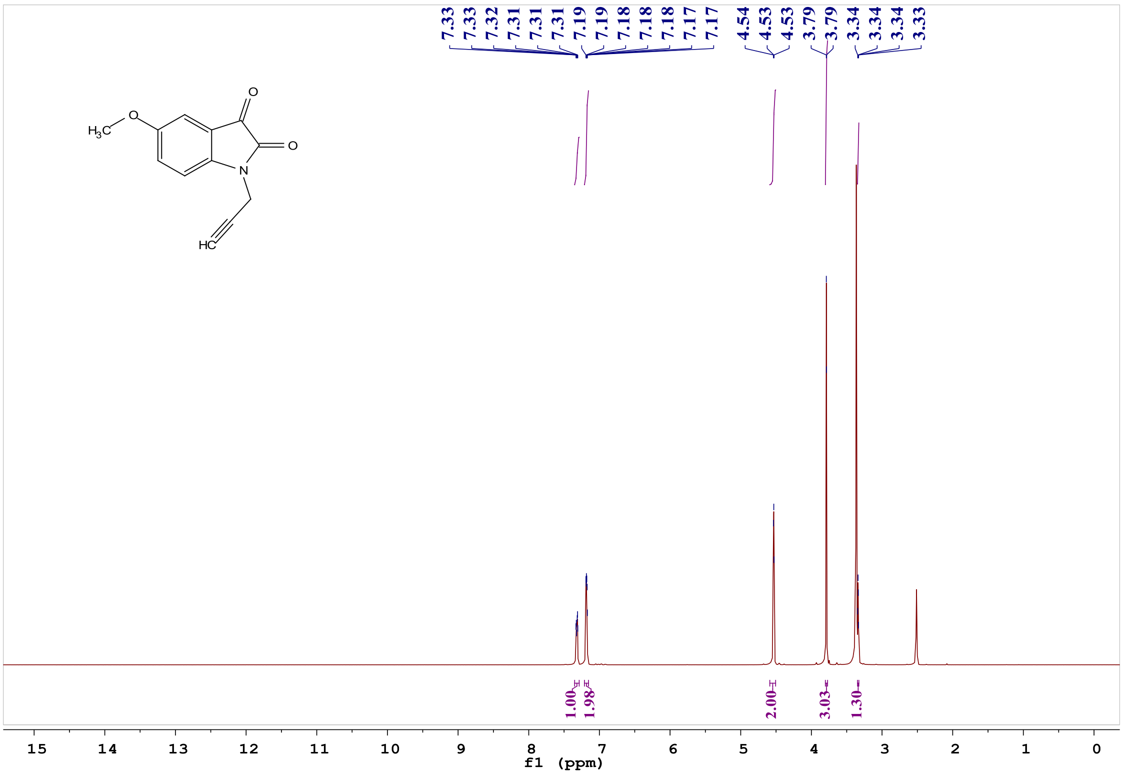

^{1}H NMR of is2-18

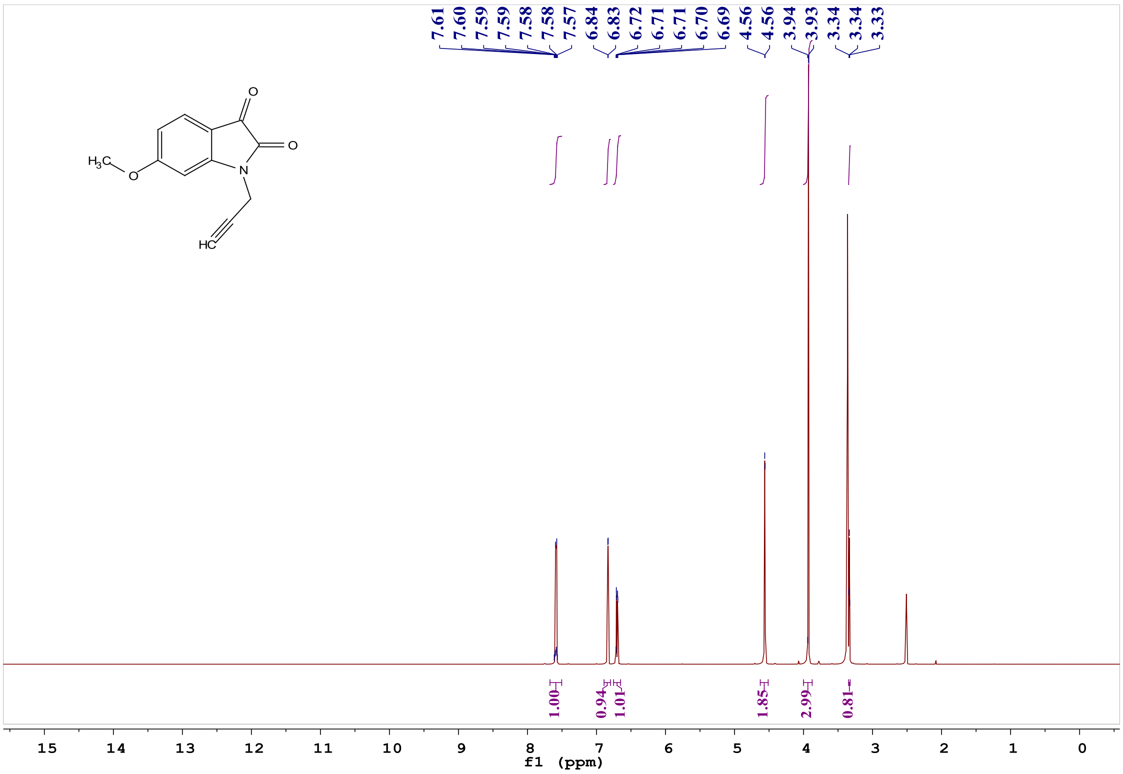

^{13}C NMR of is2-18

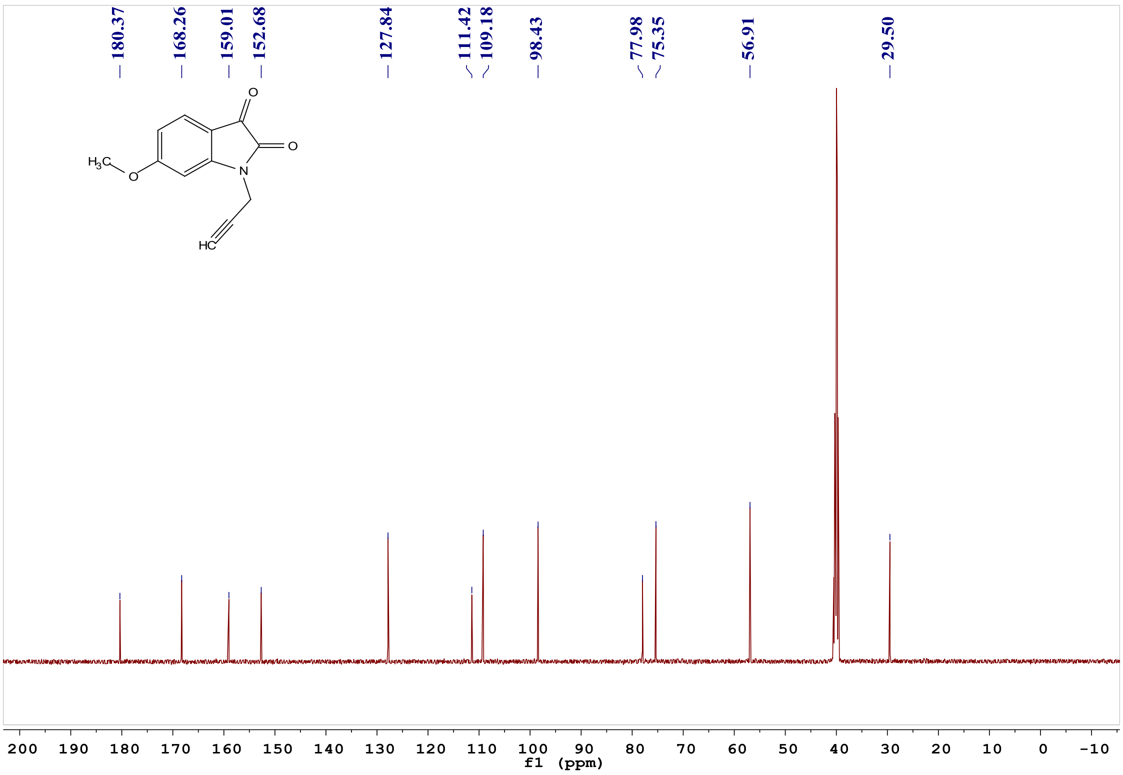

^{1}H NMR of is2-19

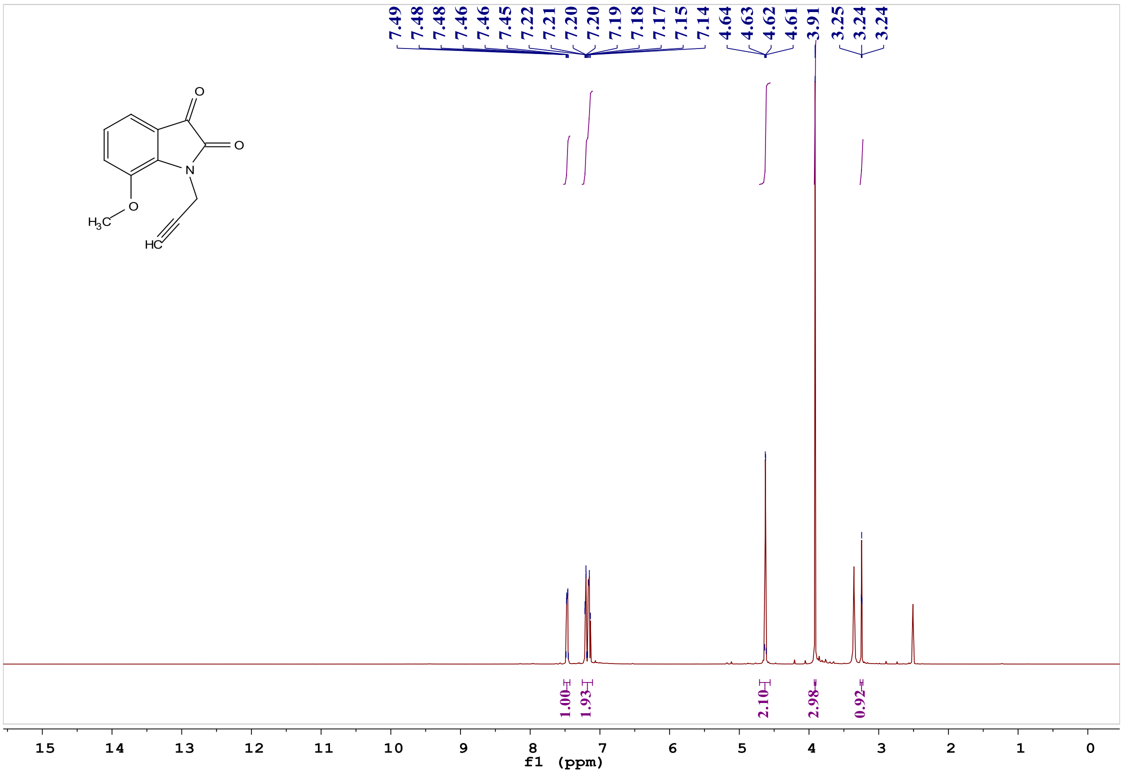

^{13}C NMR of is2-19

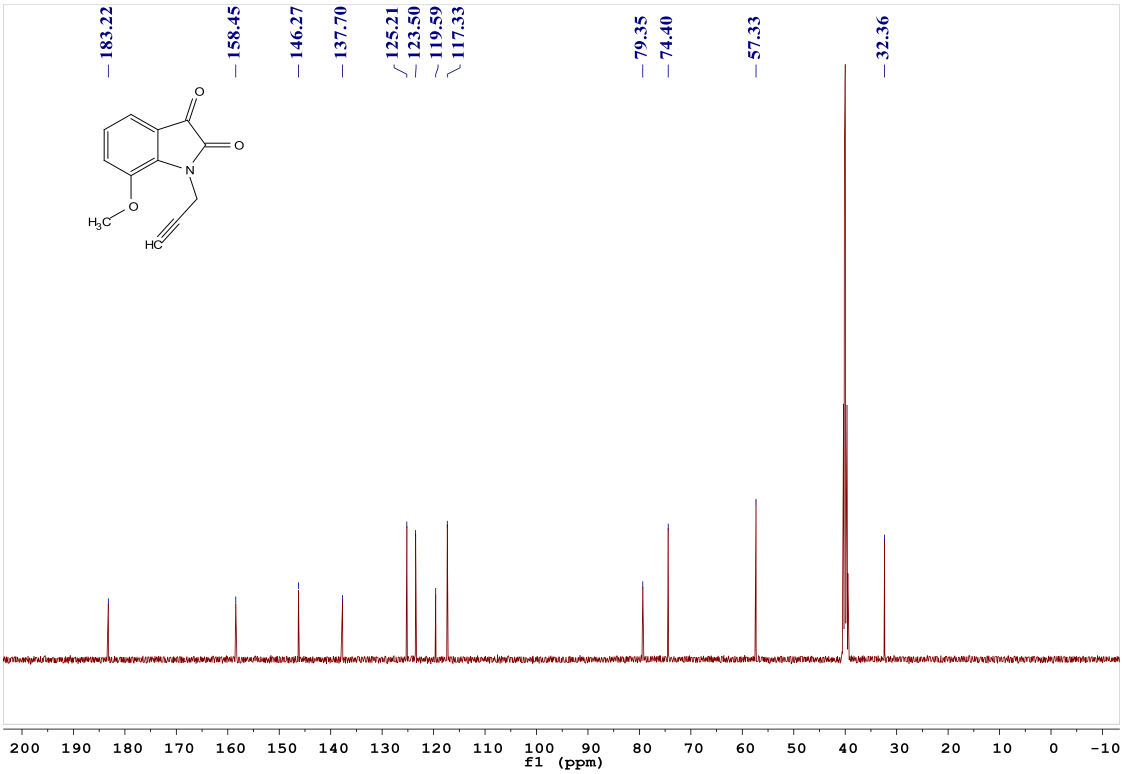

^{1}H NMR of is2-20

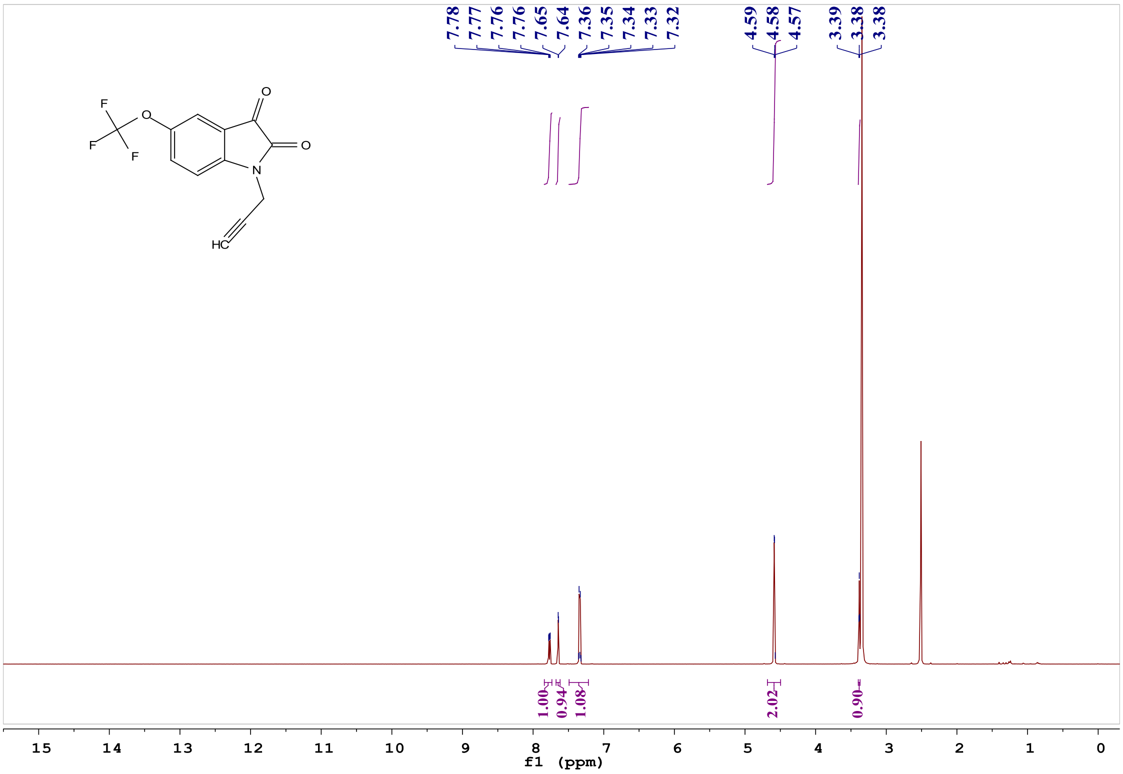

^{13}C NMR of is2-20

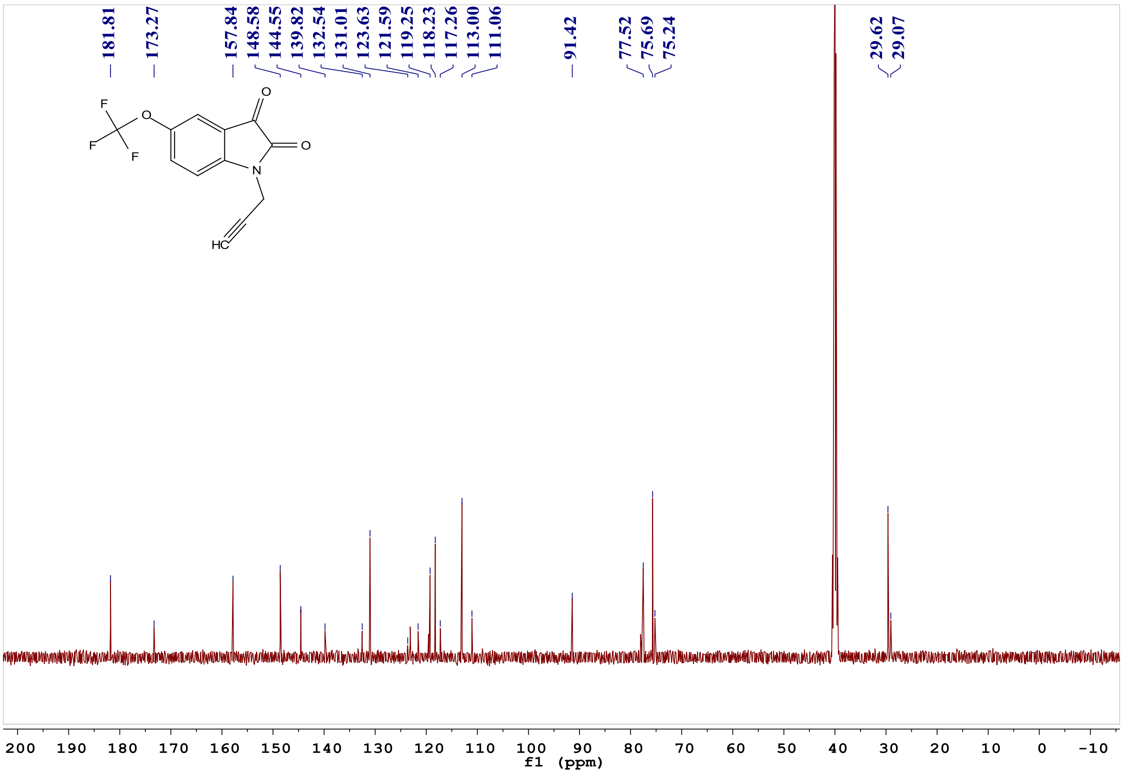

^{1}H NMR of i1

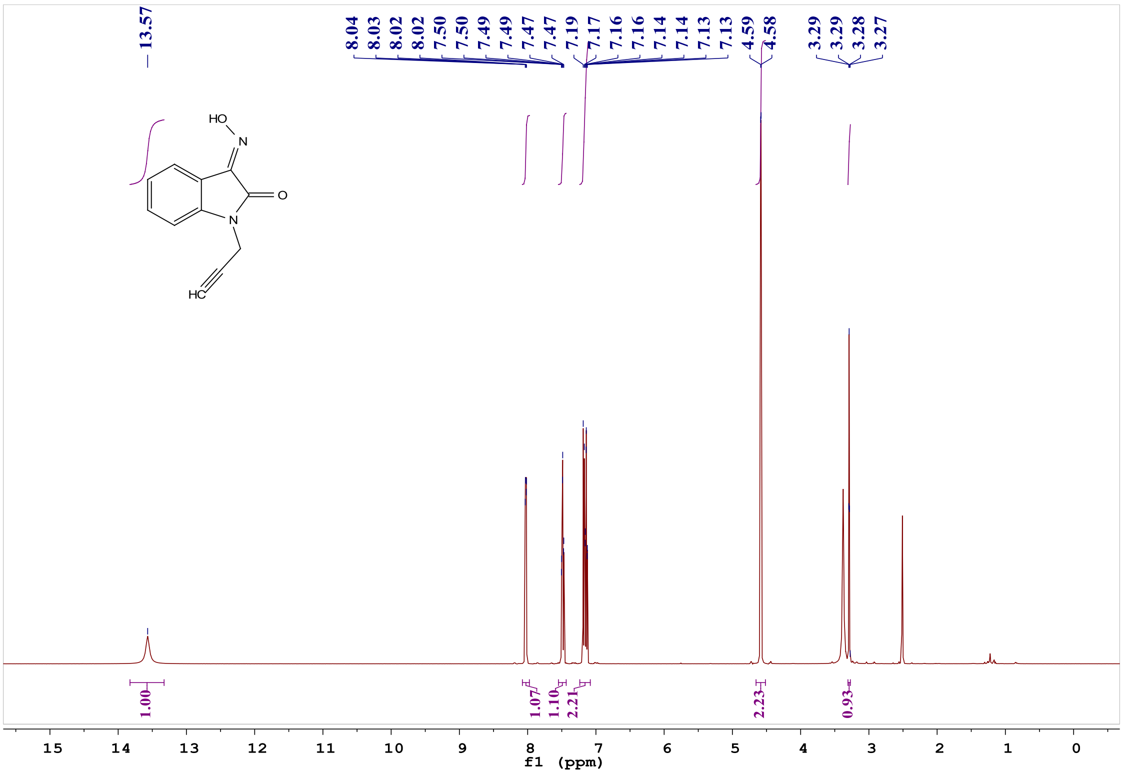

^{13}C NMR of i1

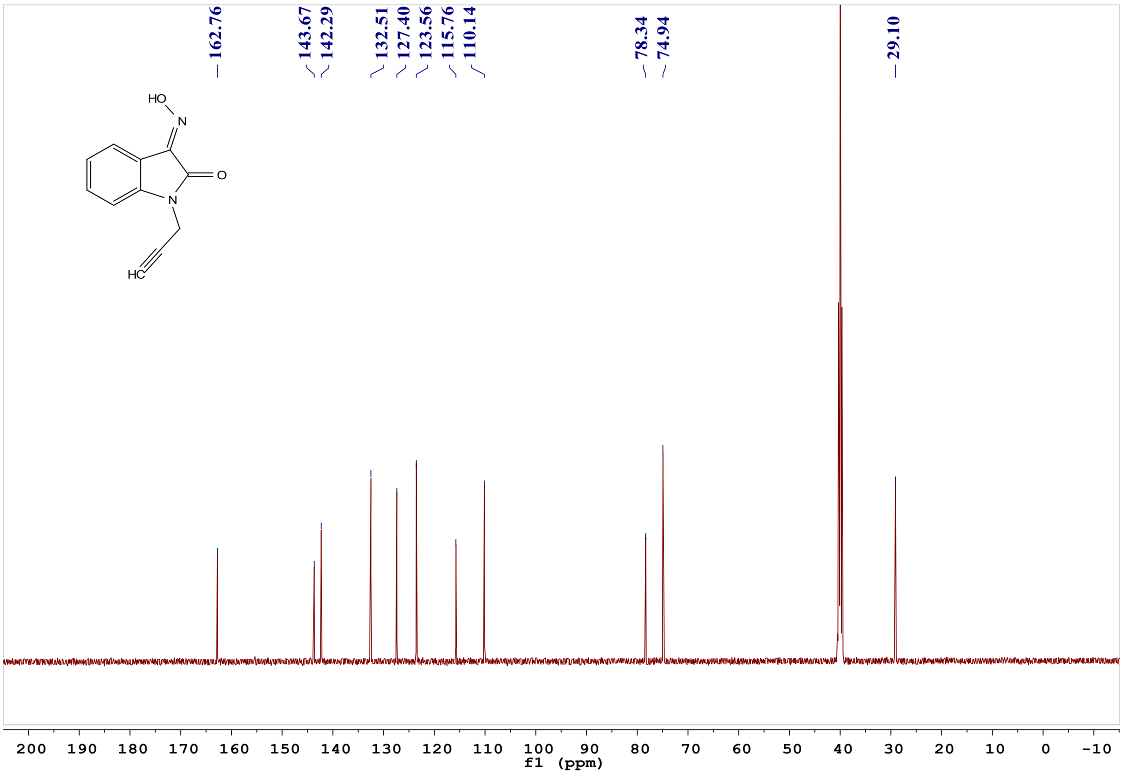

^{1}H NMR of i2

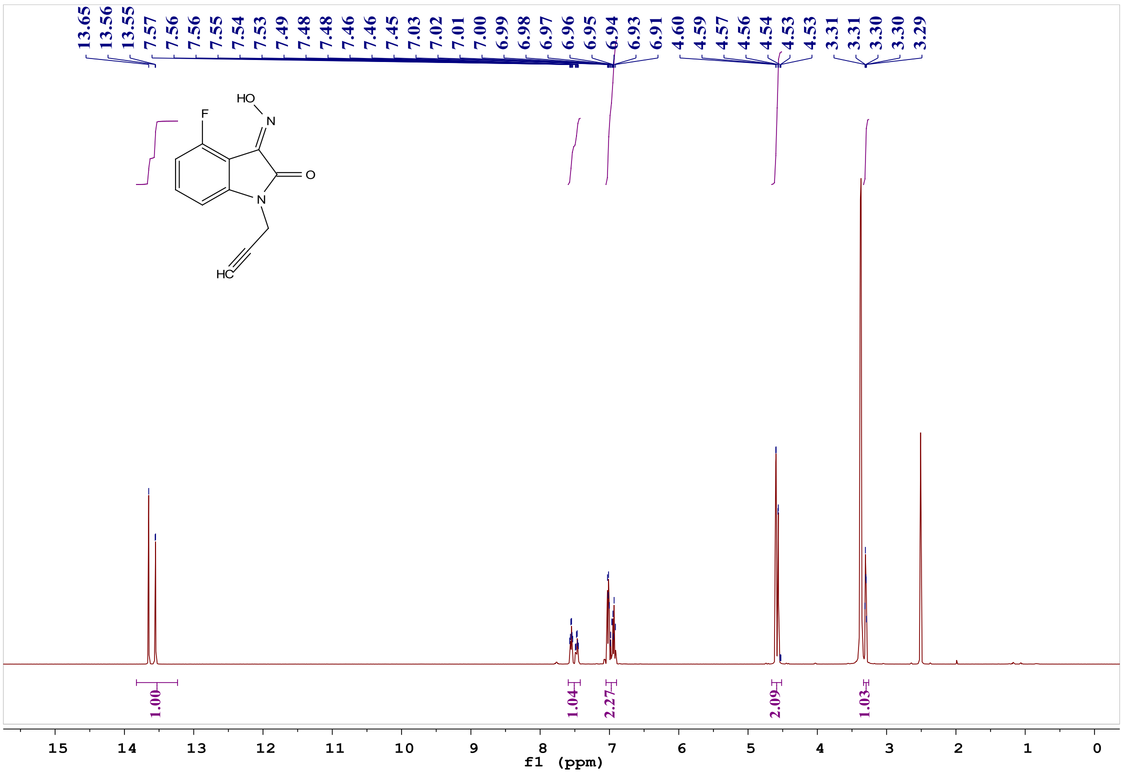

^{13}C NMR of i2

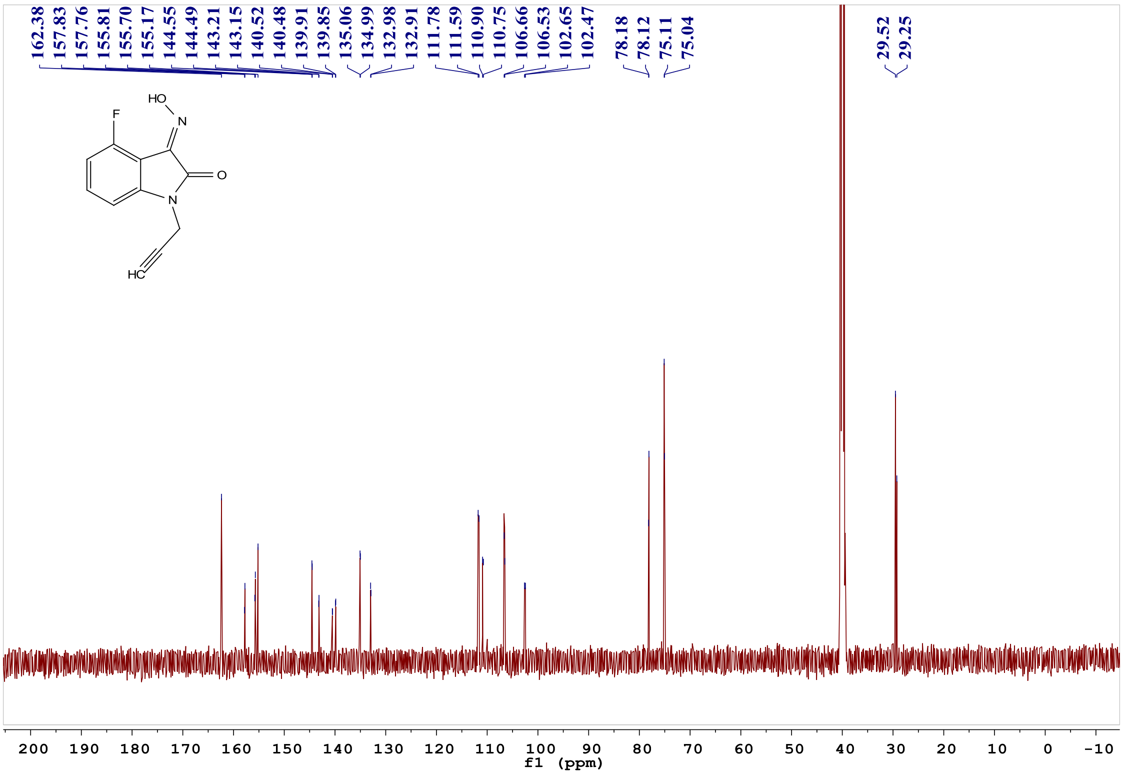

^{1}H NMR of i3

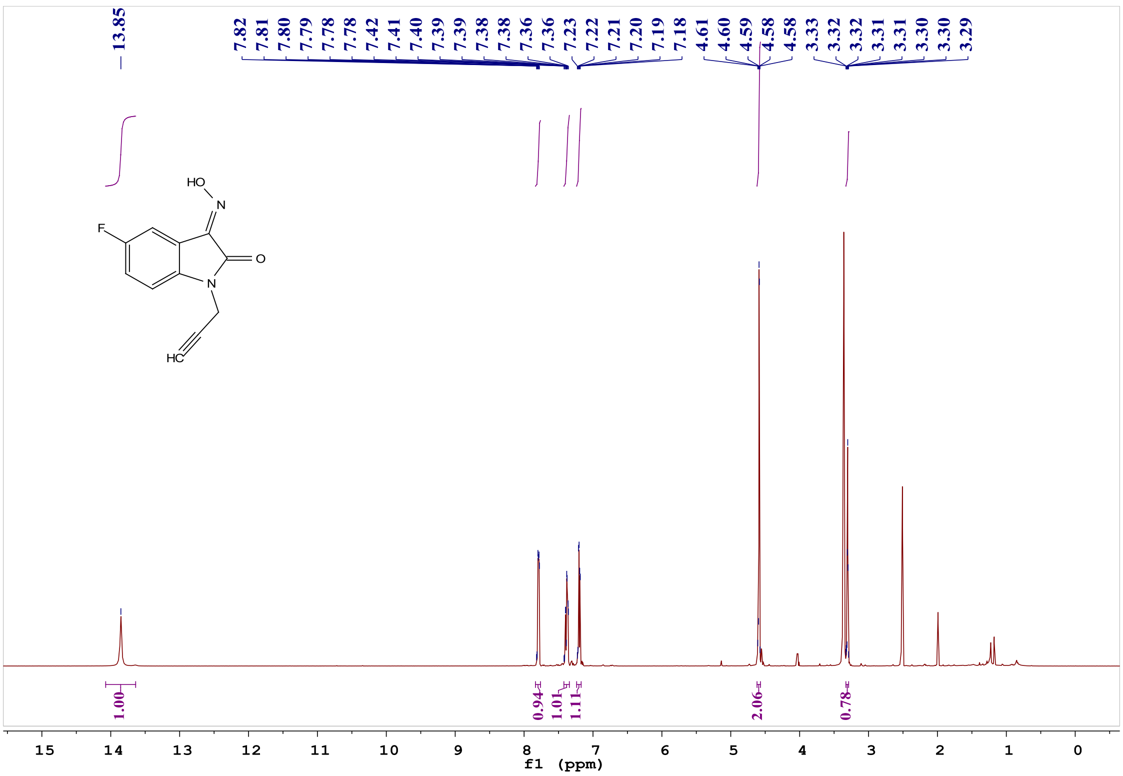

^{13}C NMR of i3

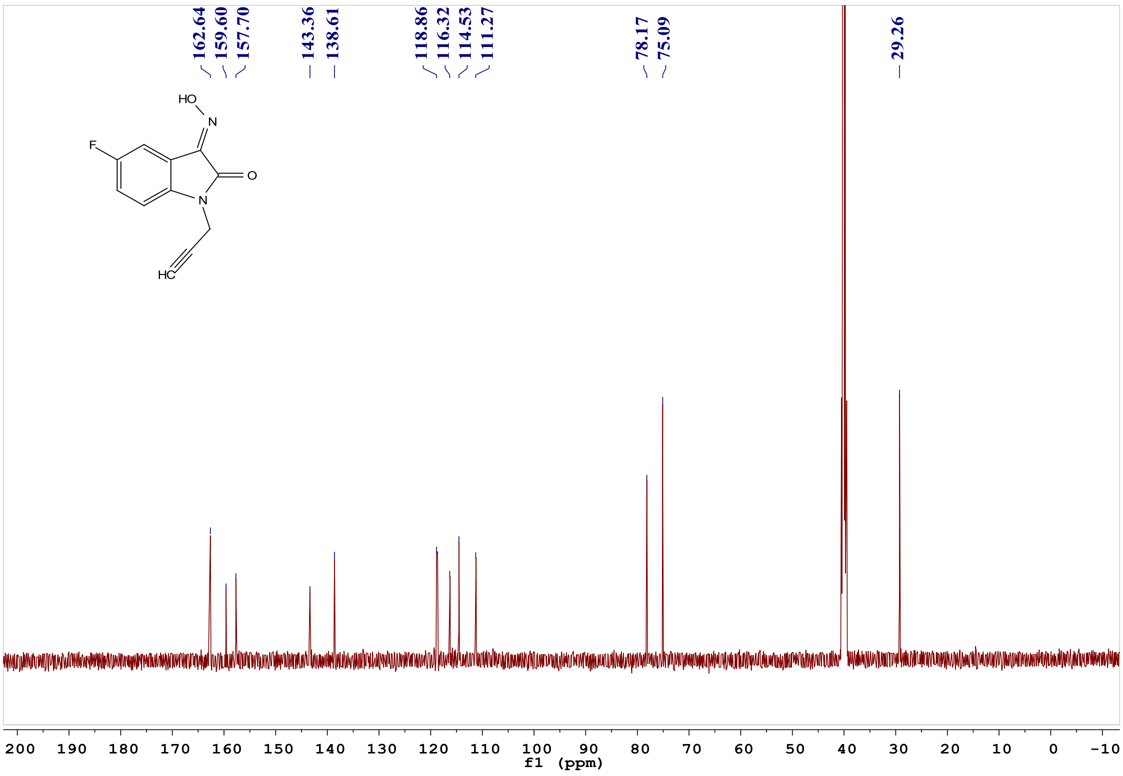

^{1}H NMR of i4

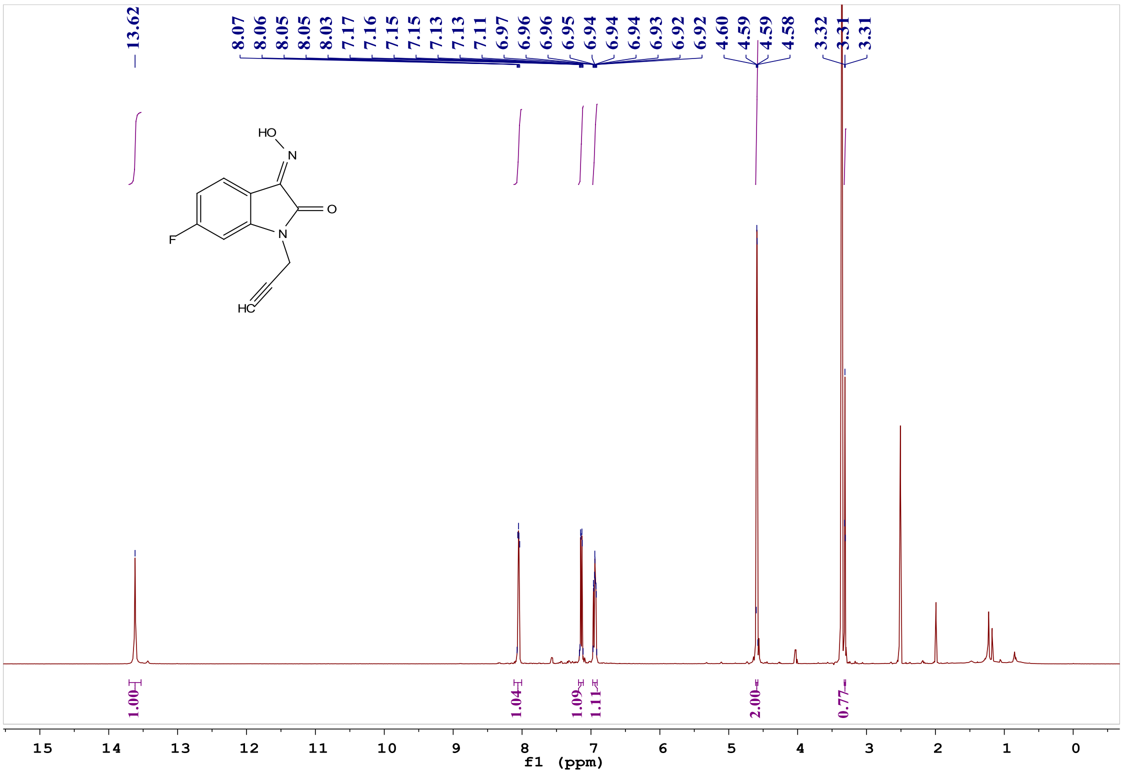

^{13}C NMR of i4

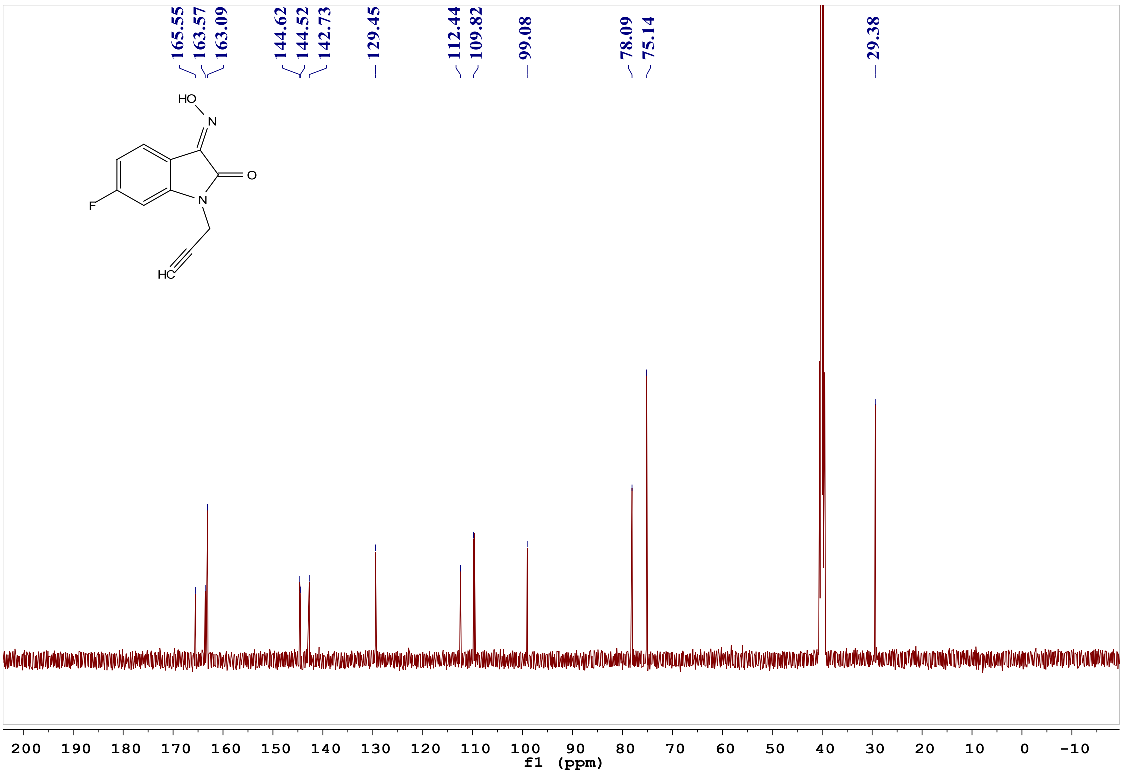

^{1}H NMR of i5

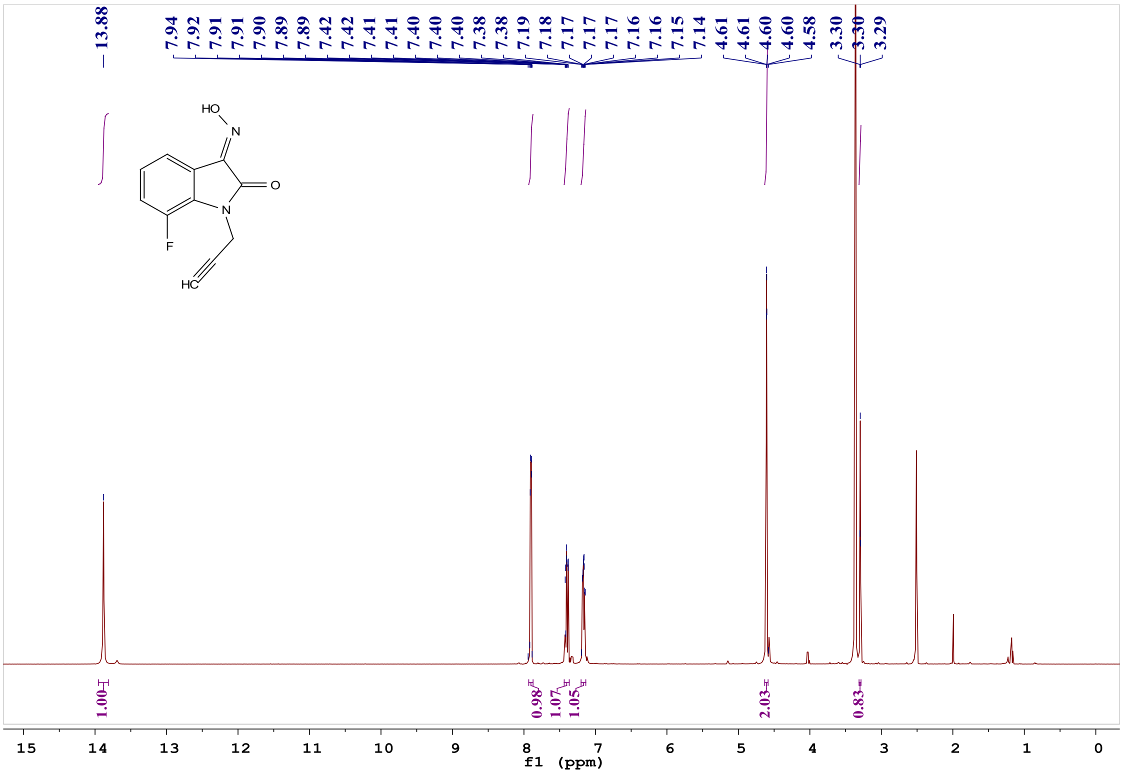

^{13}C NMR of i5

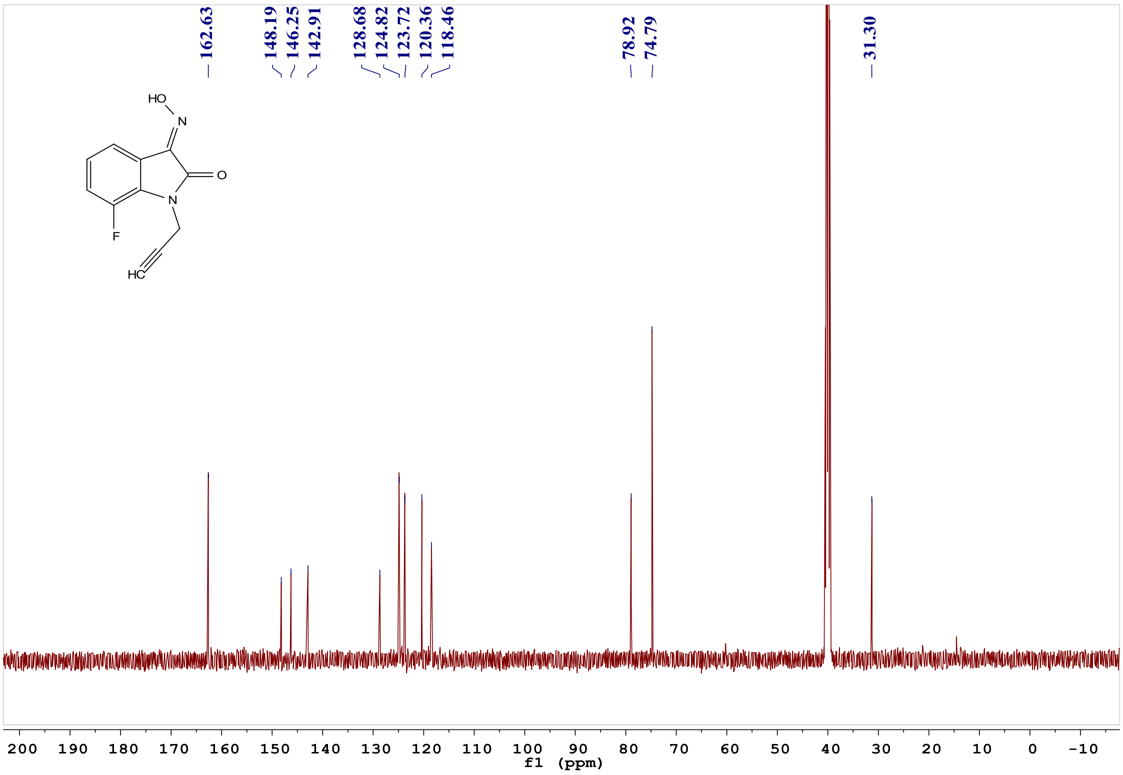

^{1}H NMR of i6

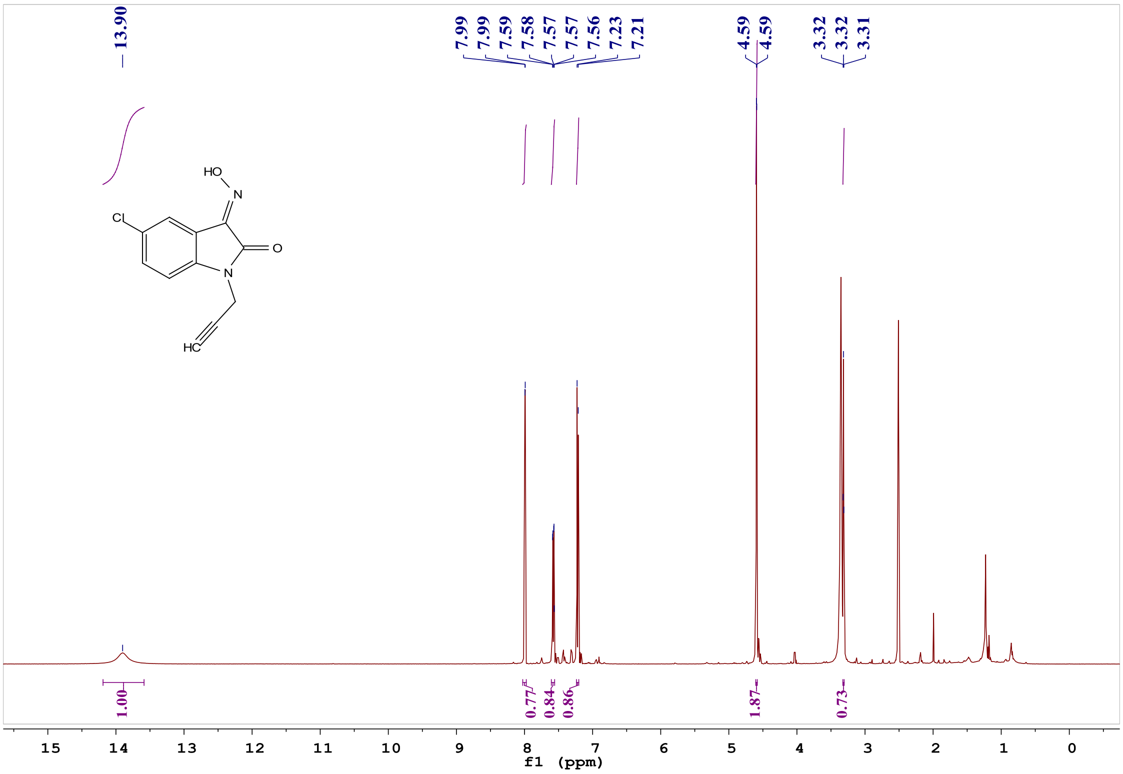

^{13}C NMR of i6

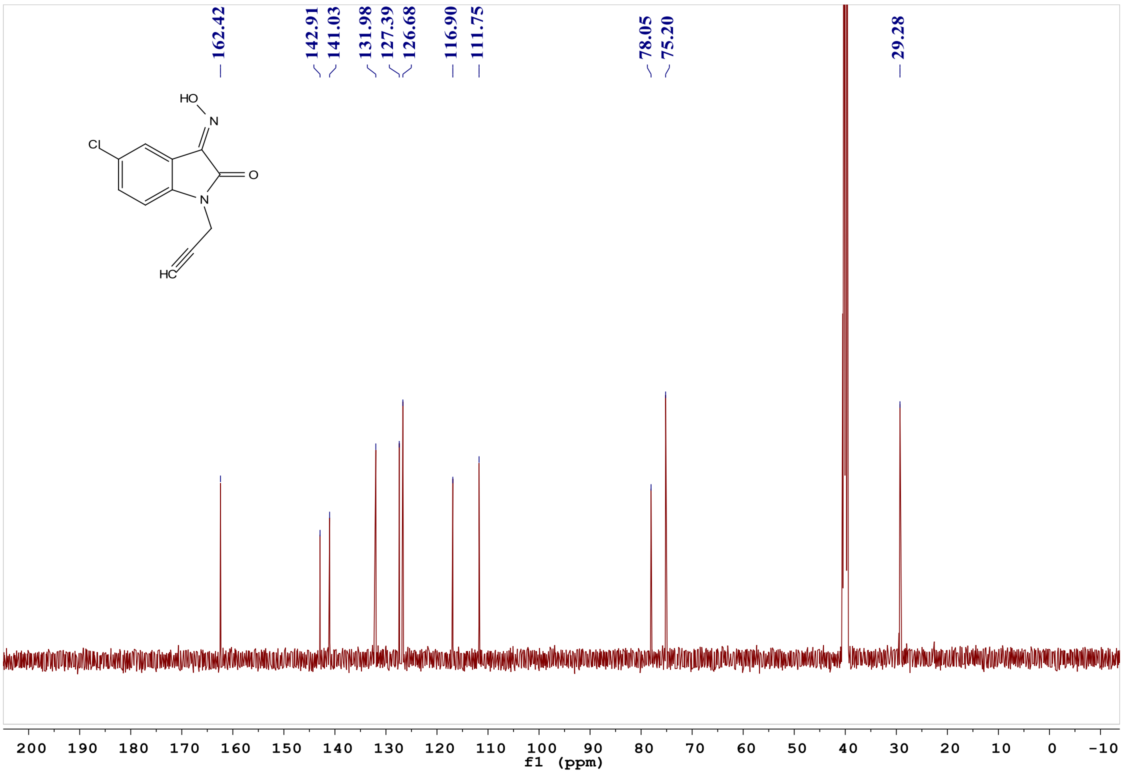

^{1}H NMR of i7

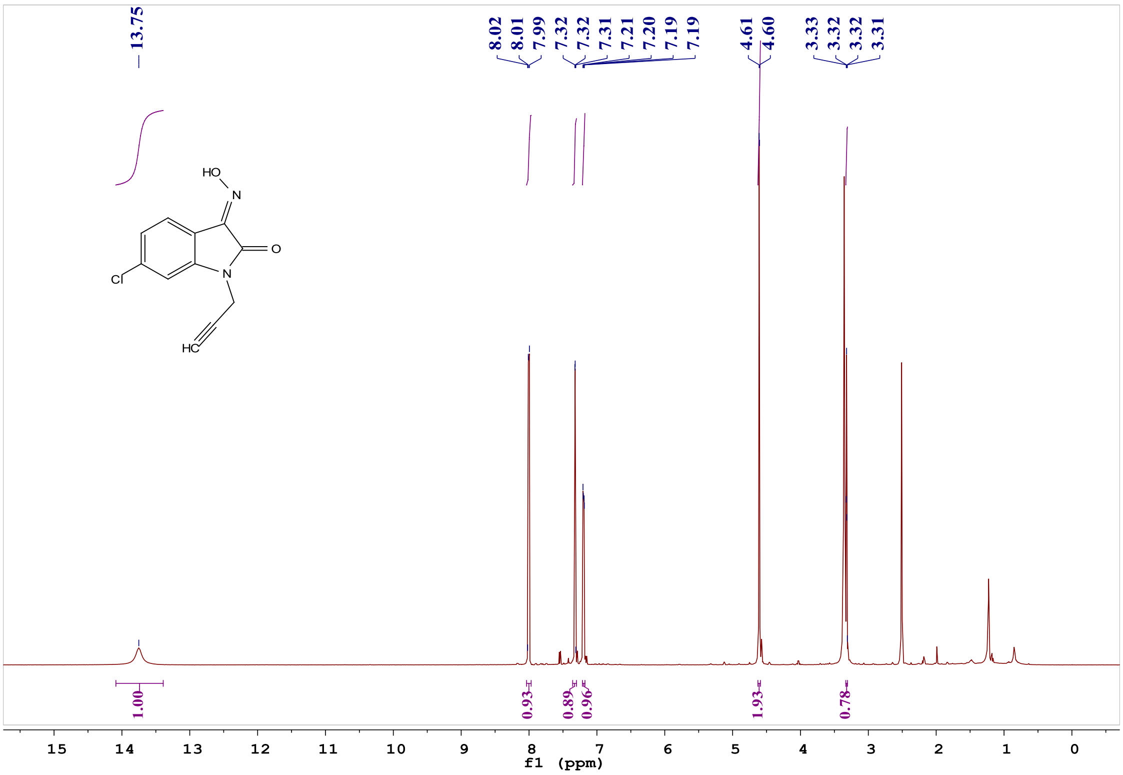

^{13}C NMR of i7

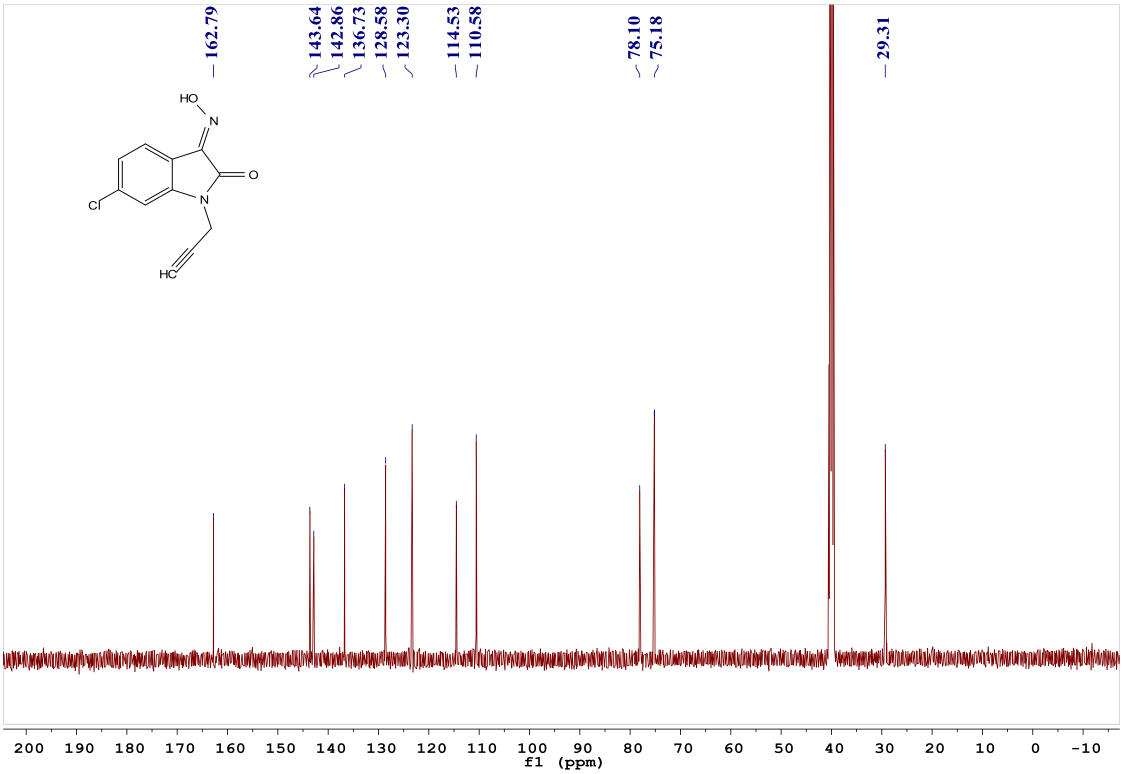

^{1}H NMR of i8

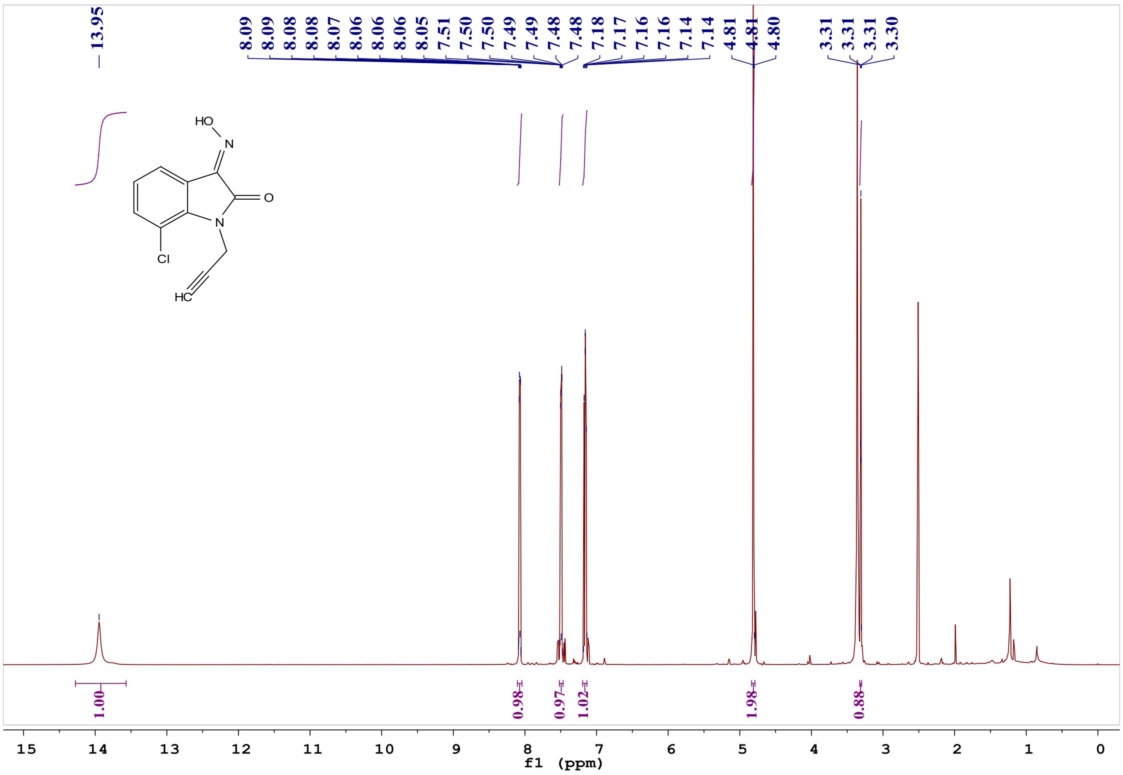

^{13}C NMR of i8

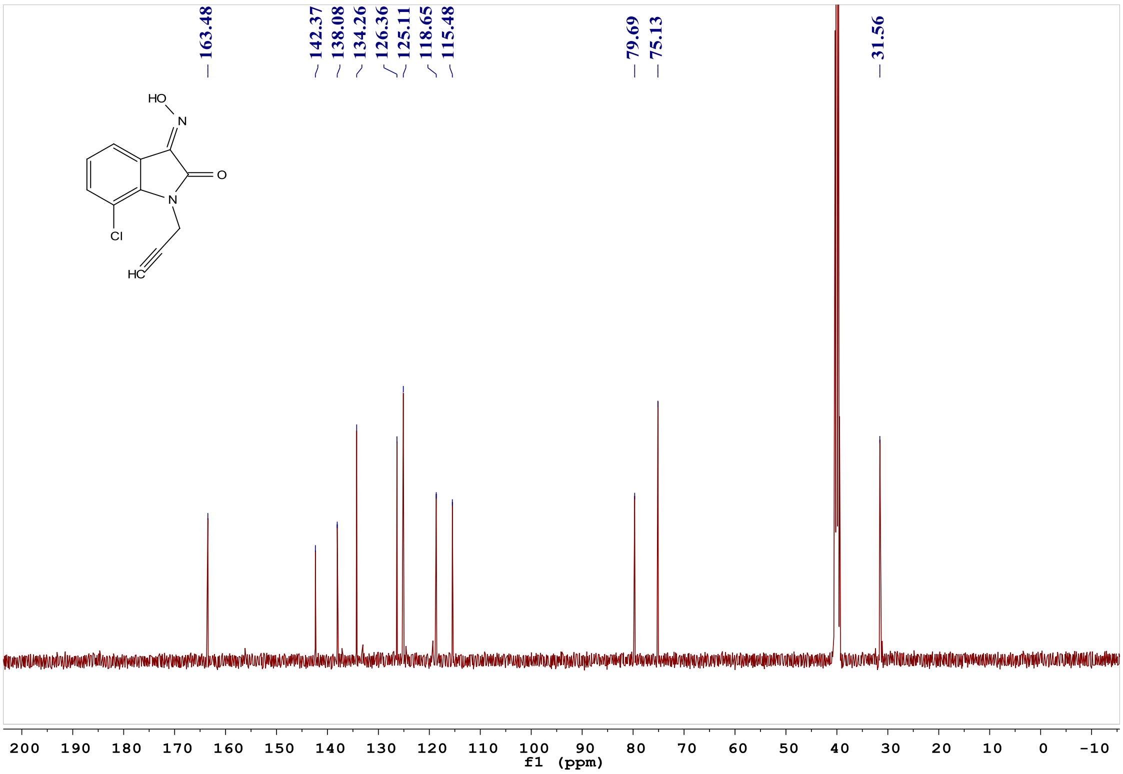

^{1}H NMR of i9

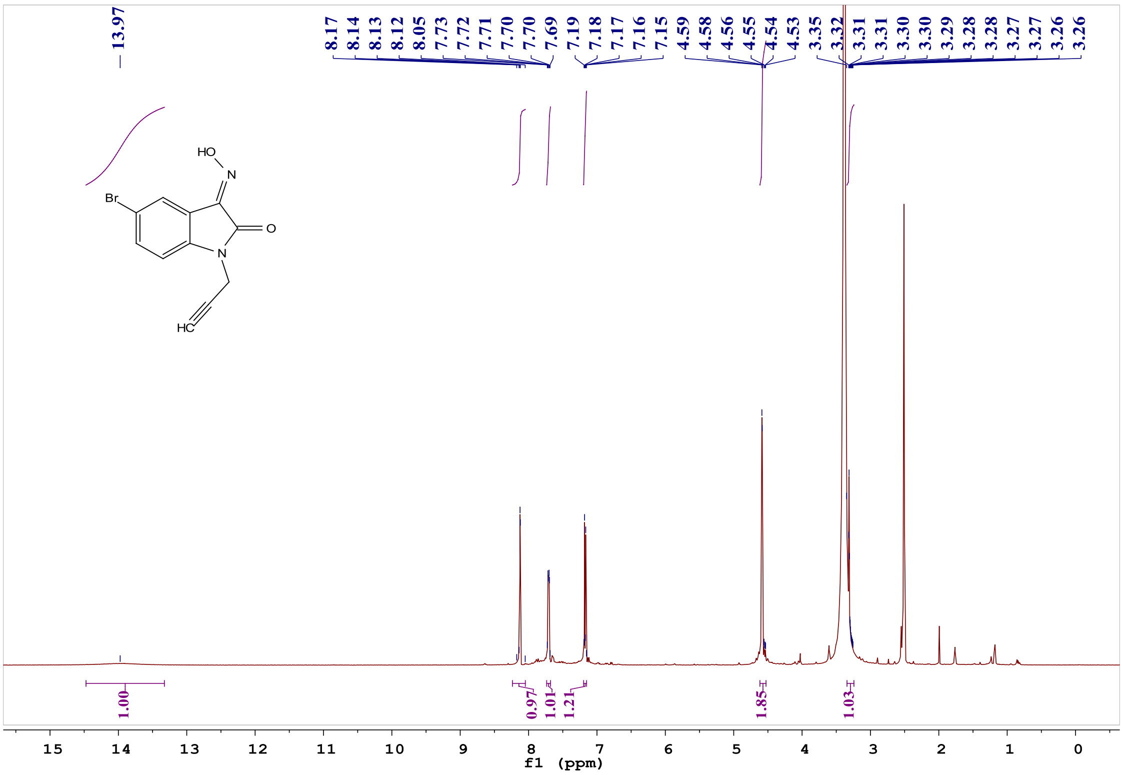

^{13}C NMR of i9

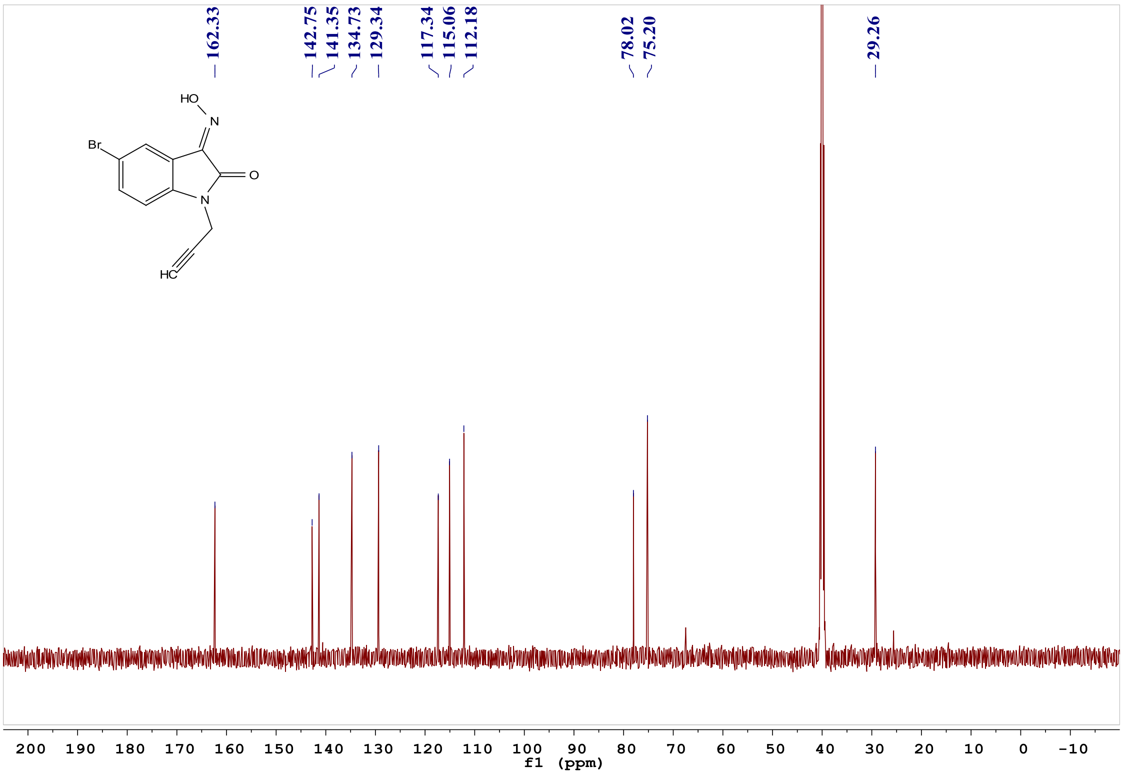

^{1}H NMR of i10

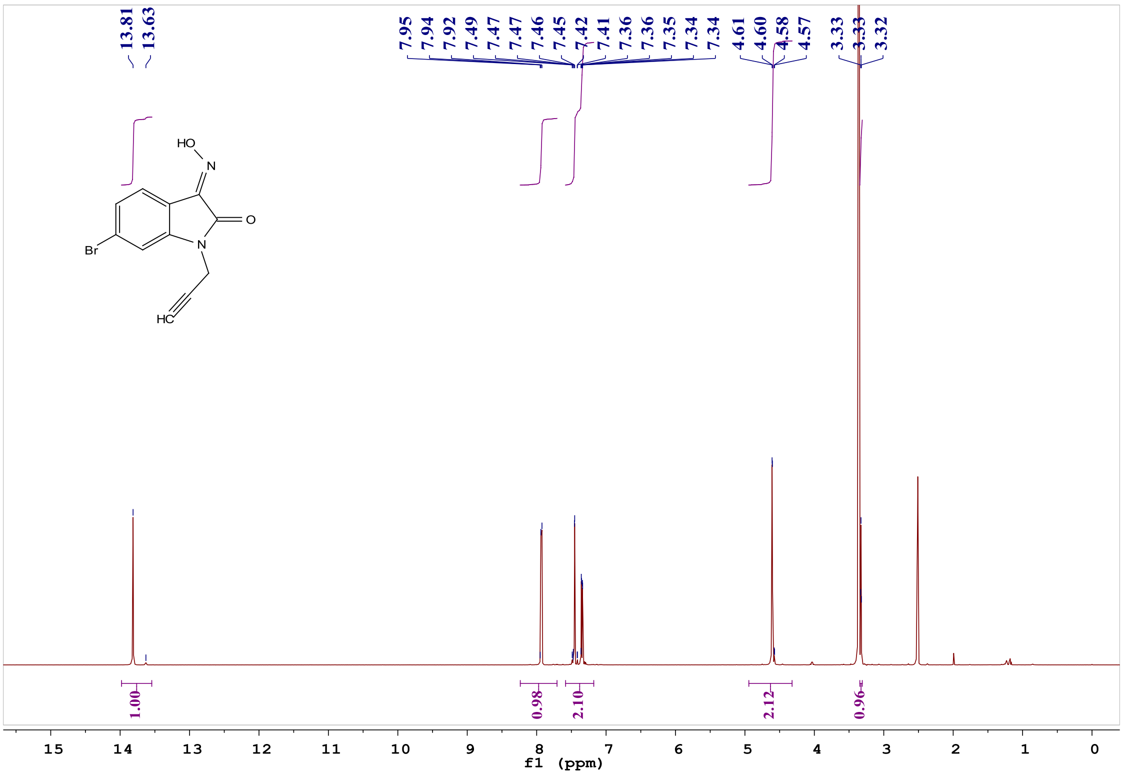

^{13}C NMR of i10

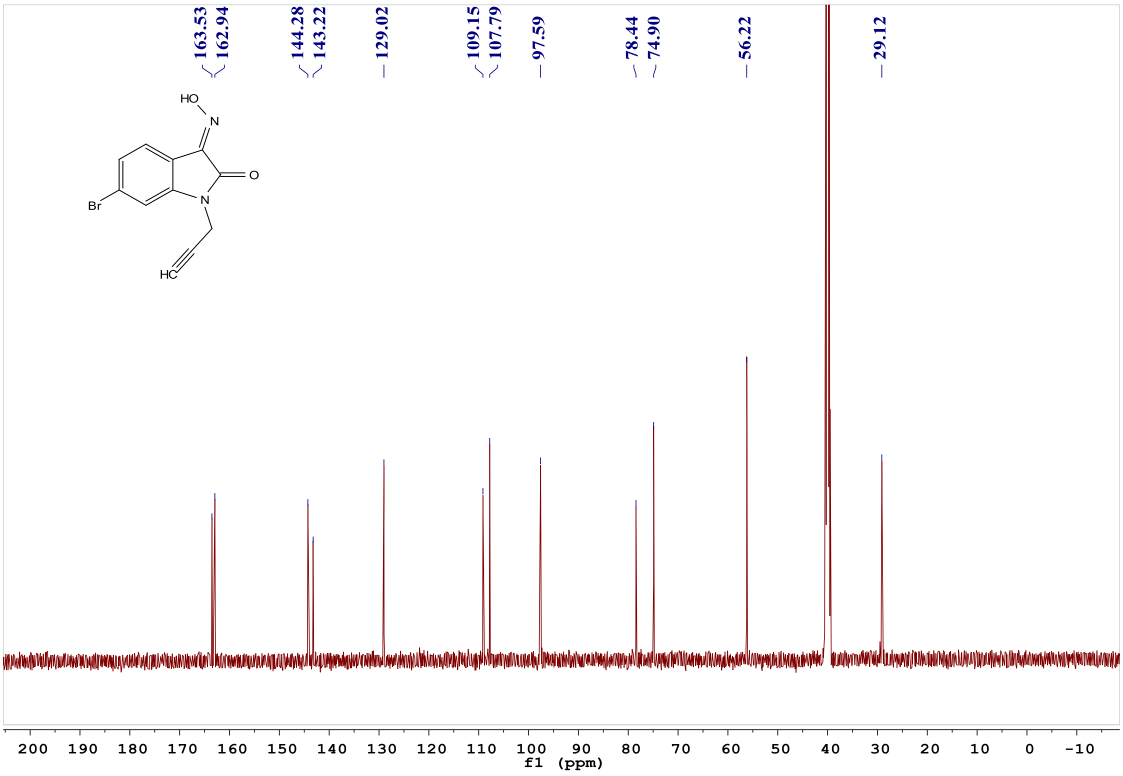

^{1}H NMR of i11

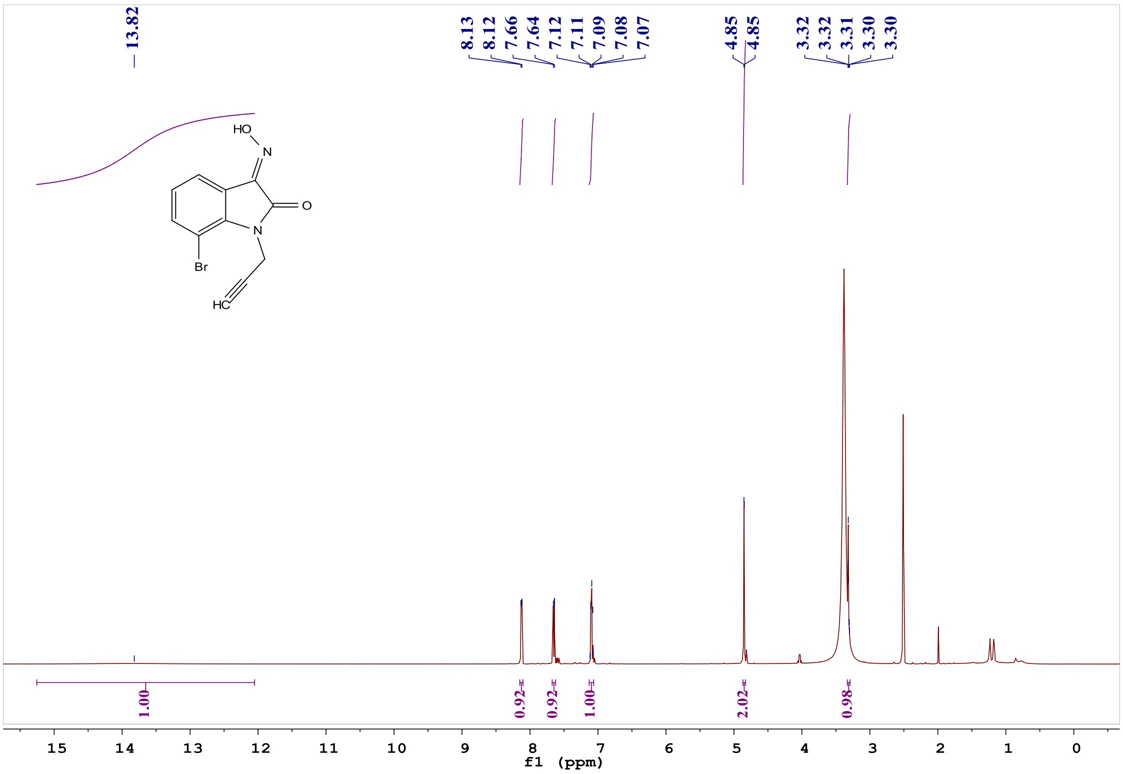

^{13}C NMR of i11

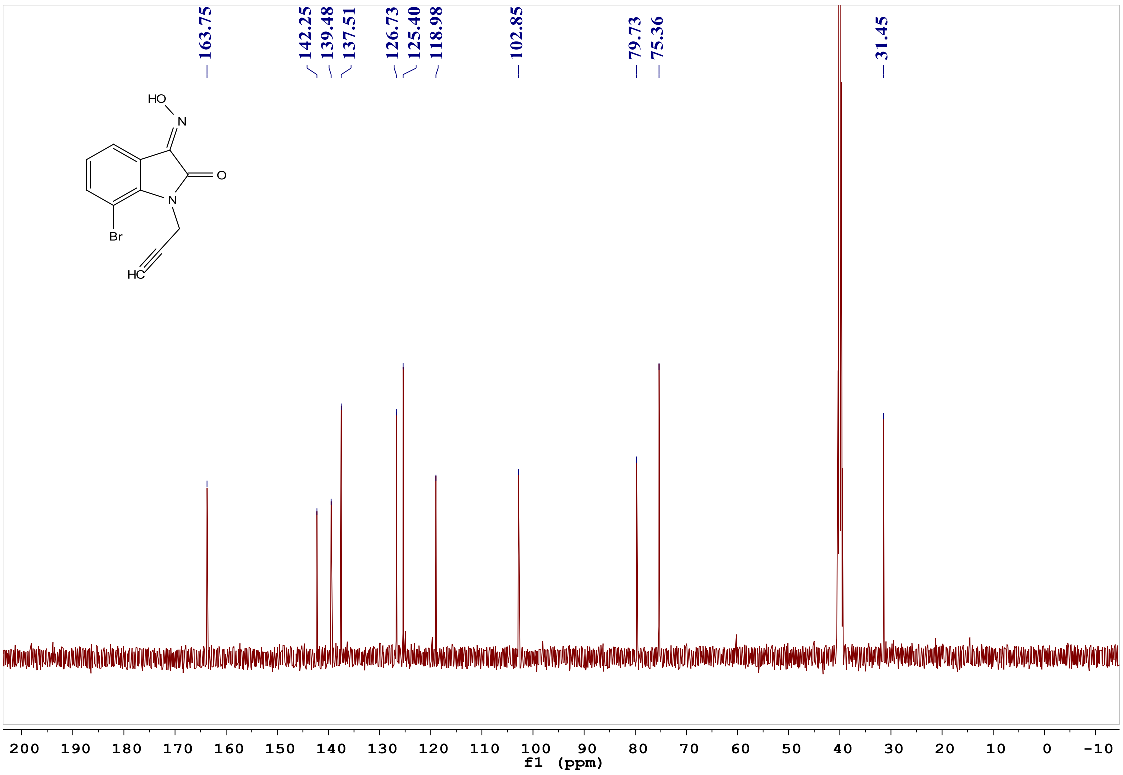

^{1}H NMR of i12

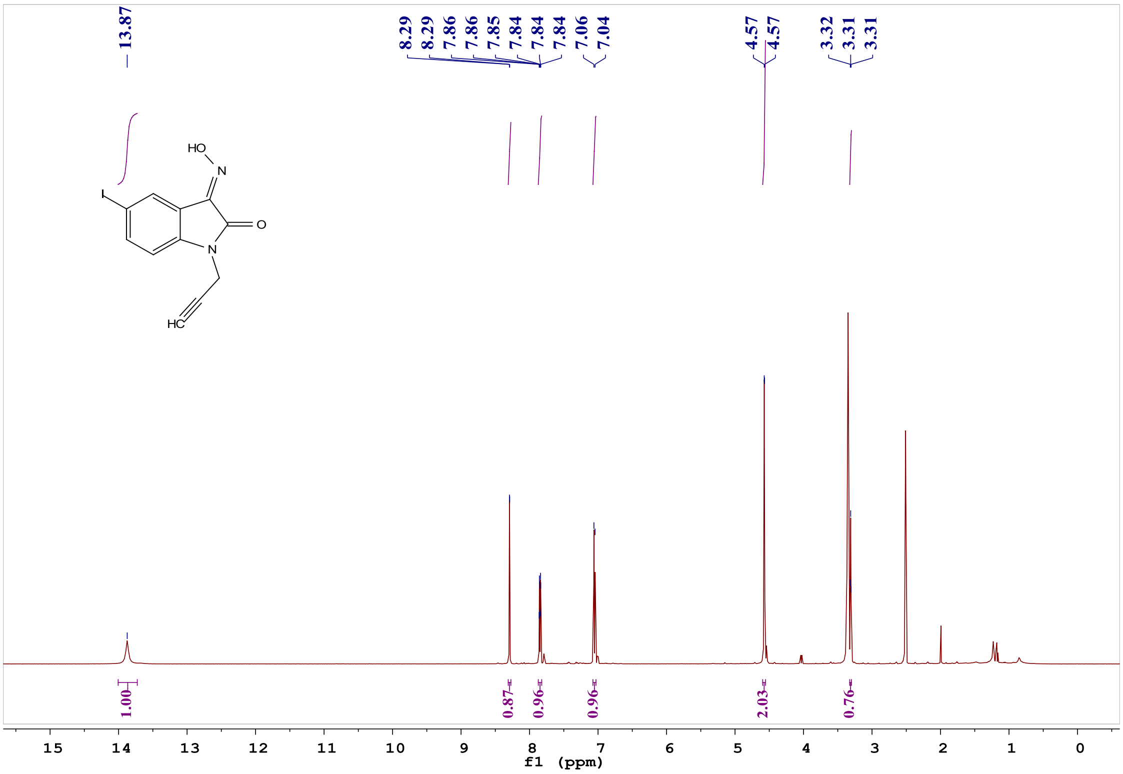

^{13}C NMR of i12

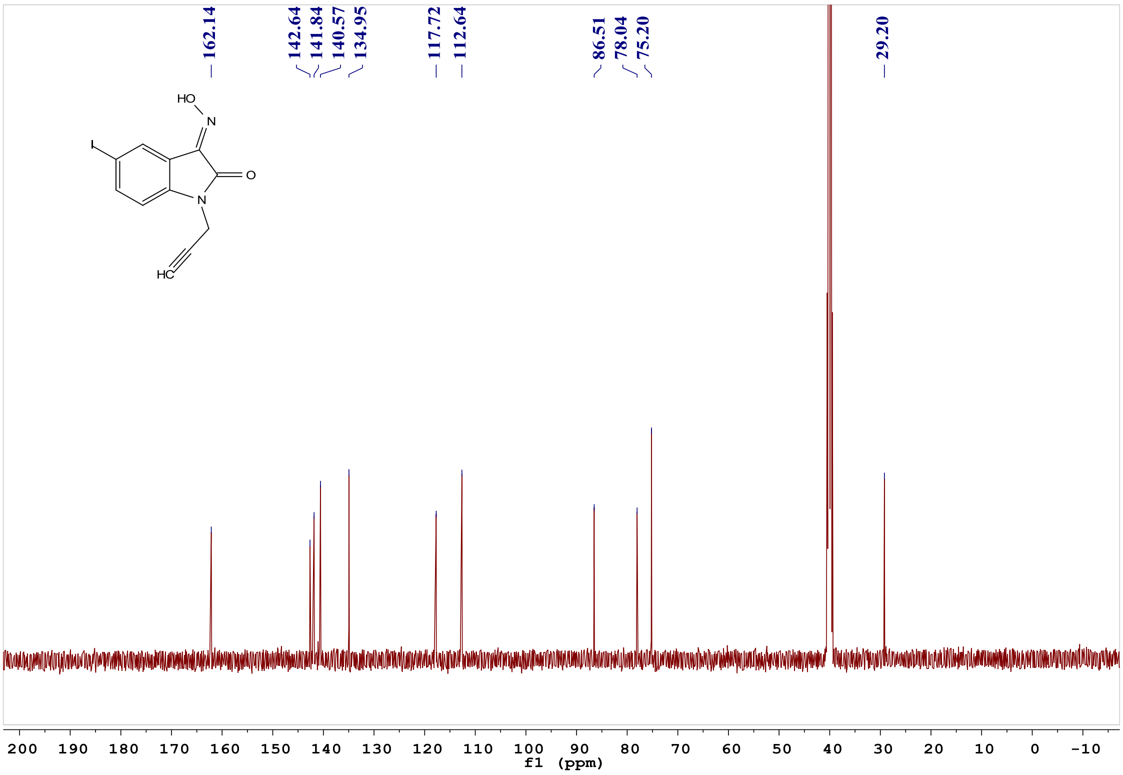

^{1}H NMR of i13

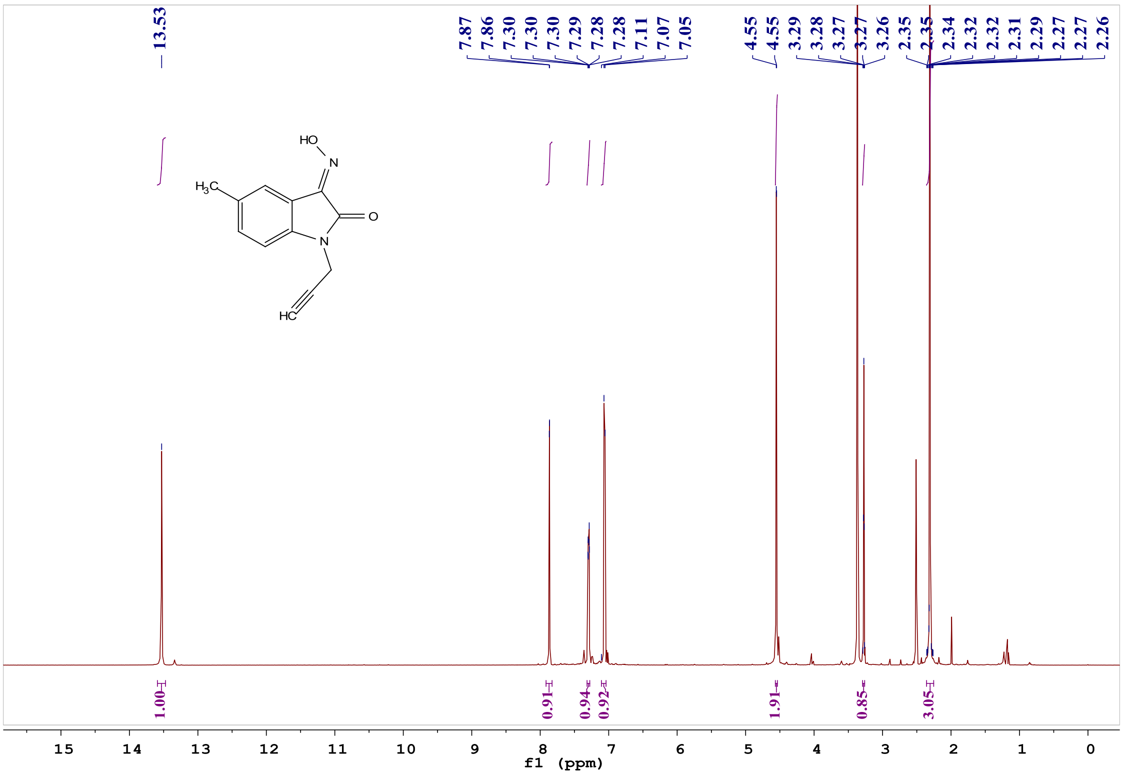

^{13}C NMR of i13

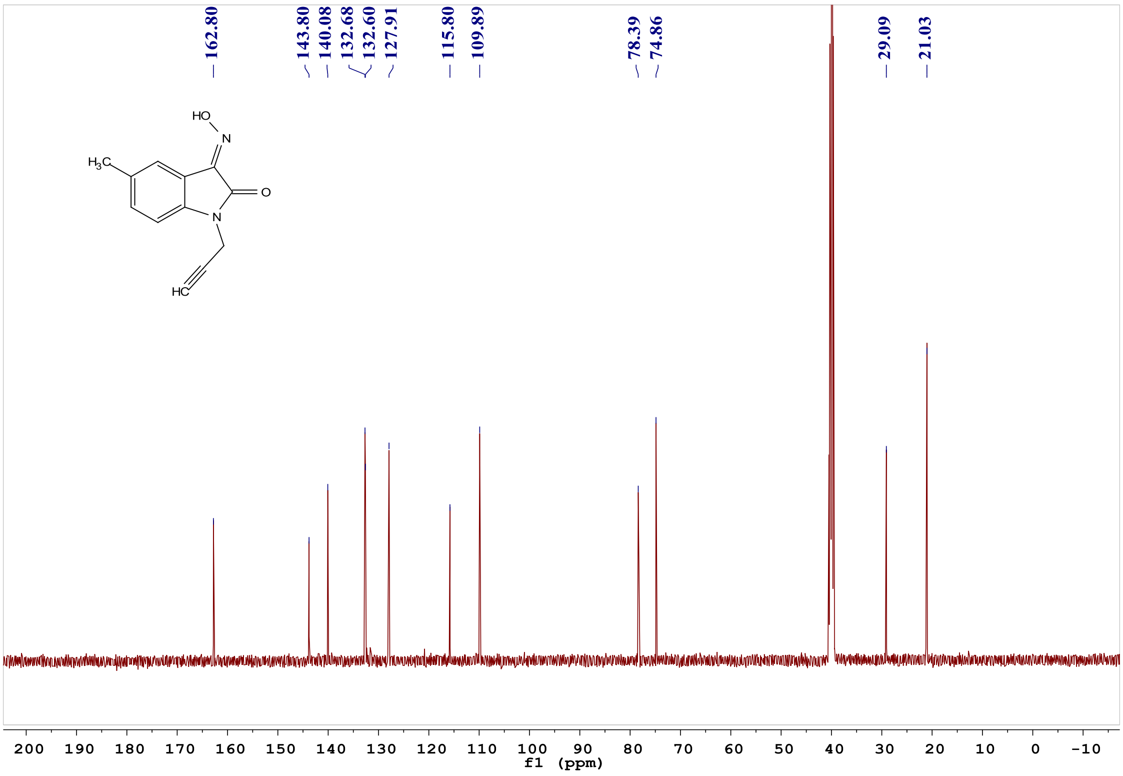

^{1}H NMR of i14

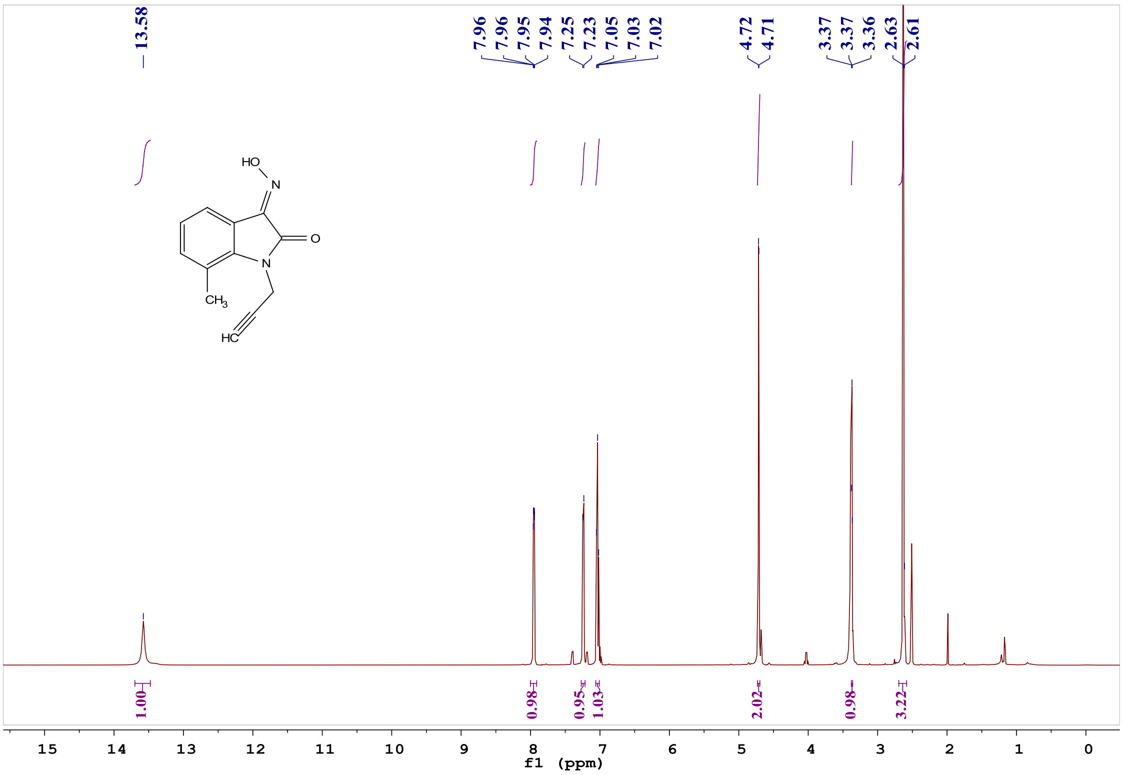

^{13}C NMR of i14

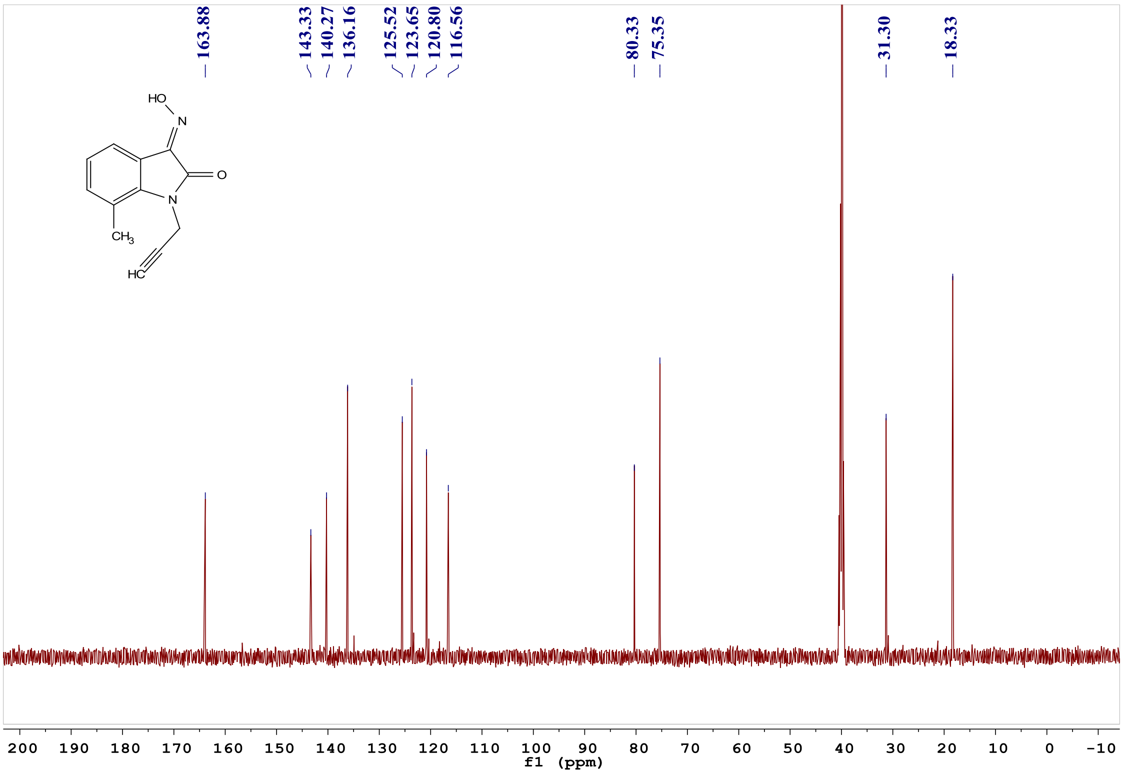

^{1}H NMR of i15

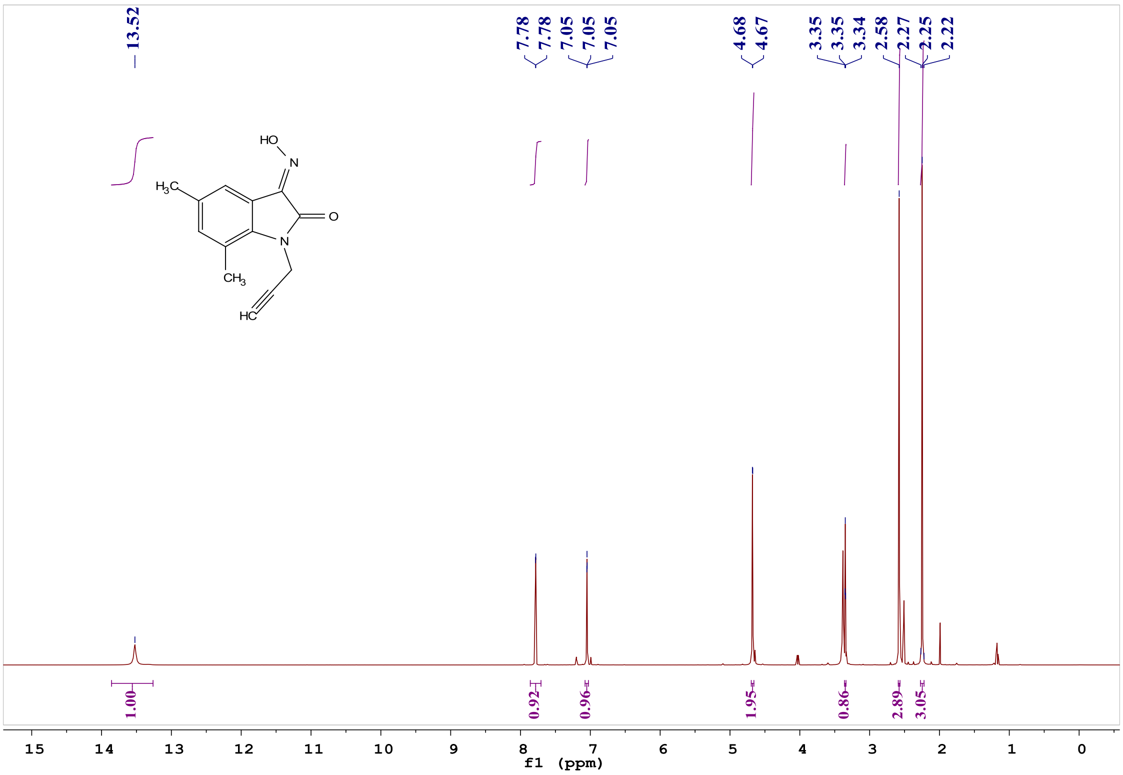

^{13}C NMR of i15

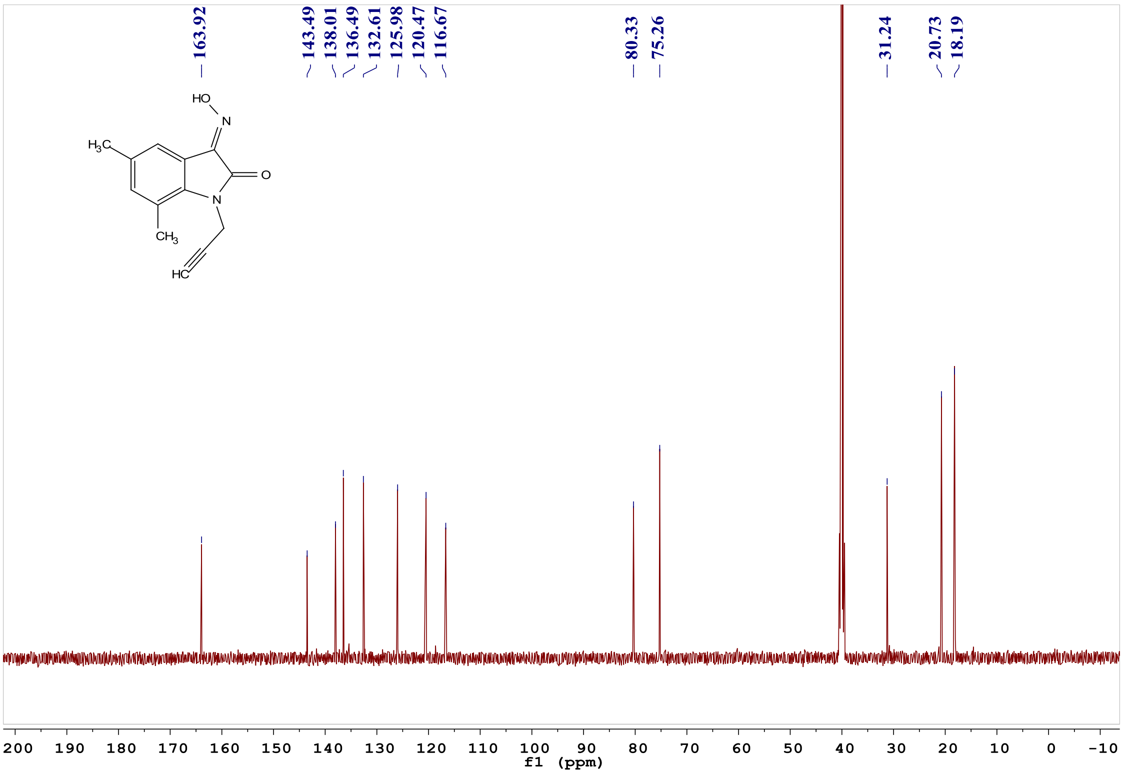

^{1}H NMR of i16

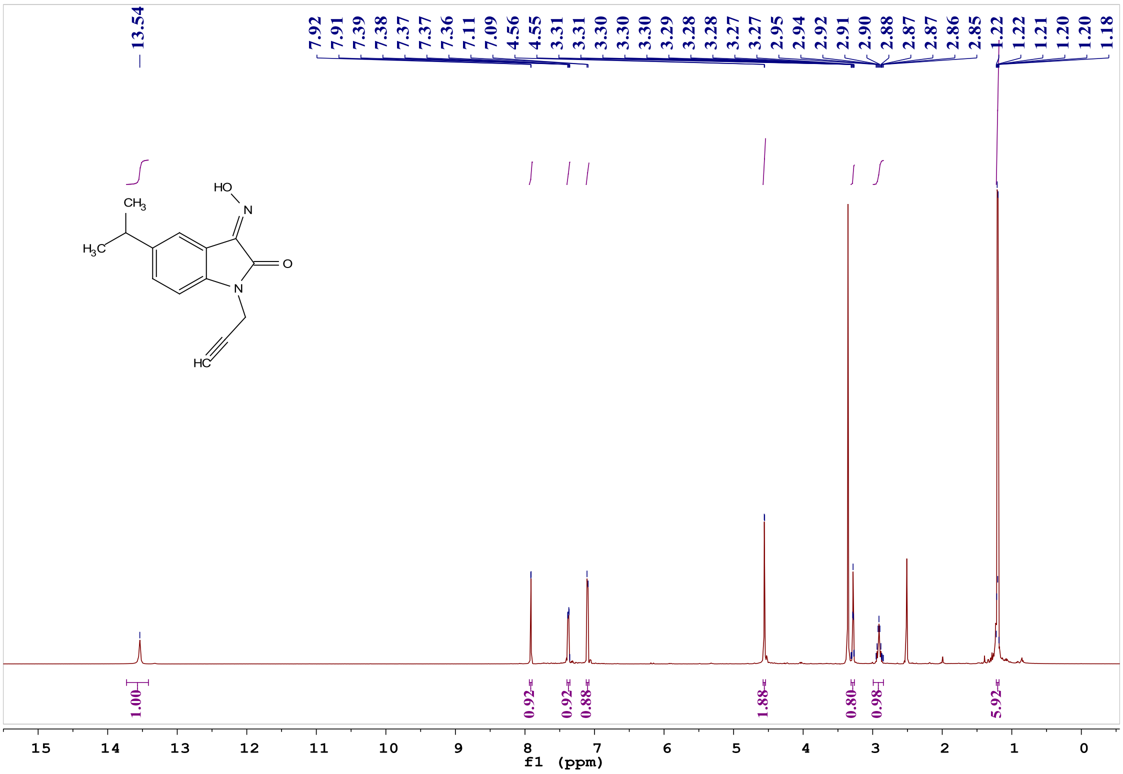

^{13}C NMR of i16

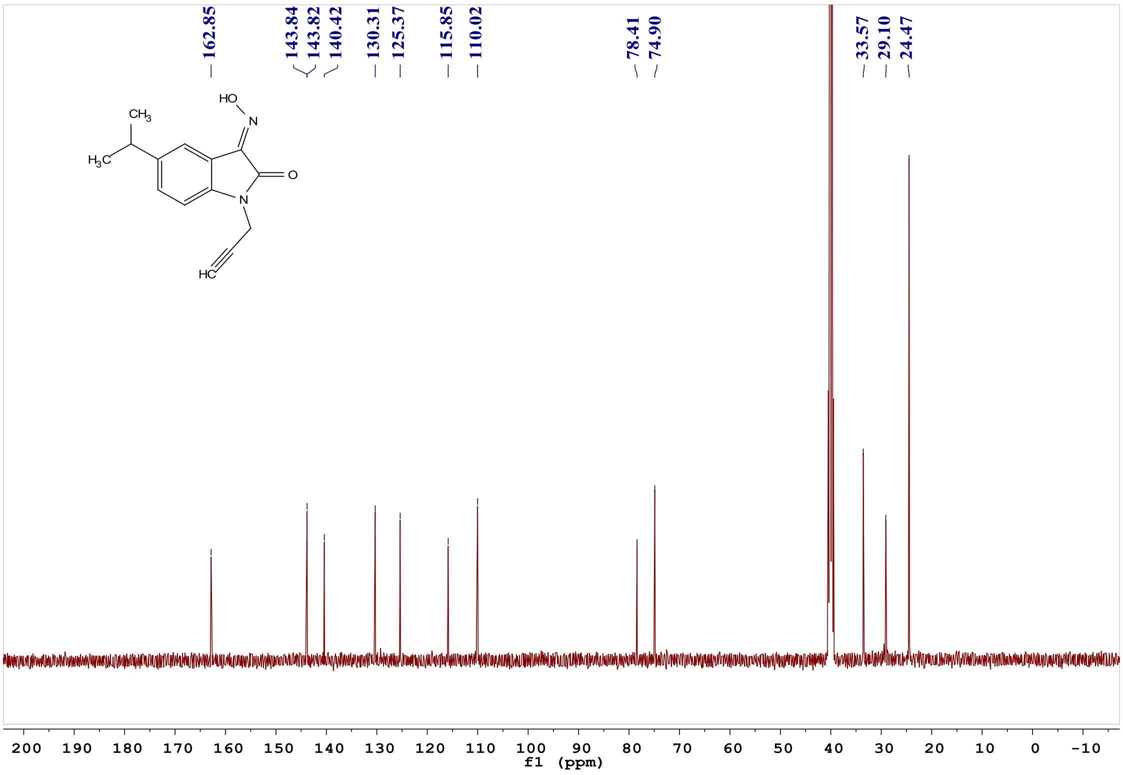

^{1}H NMR of i17

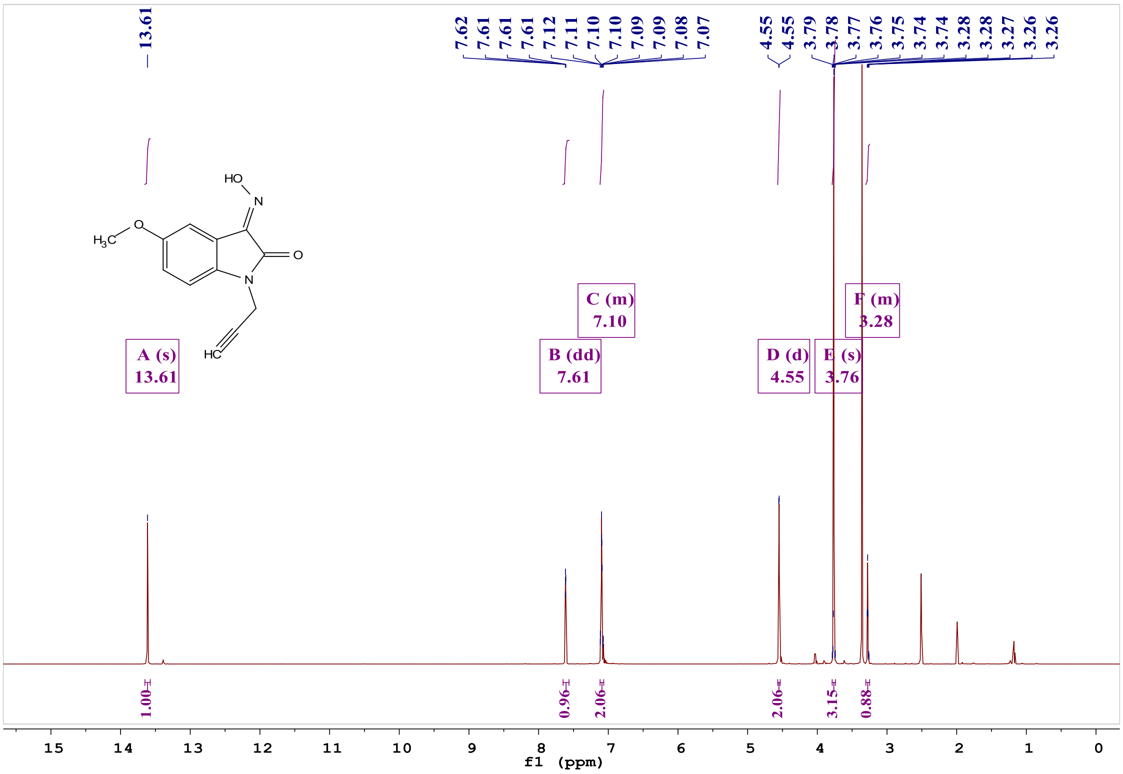

^{13}C NMR of i17

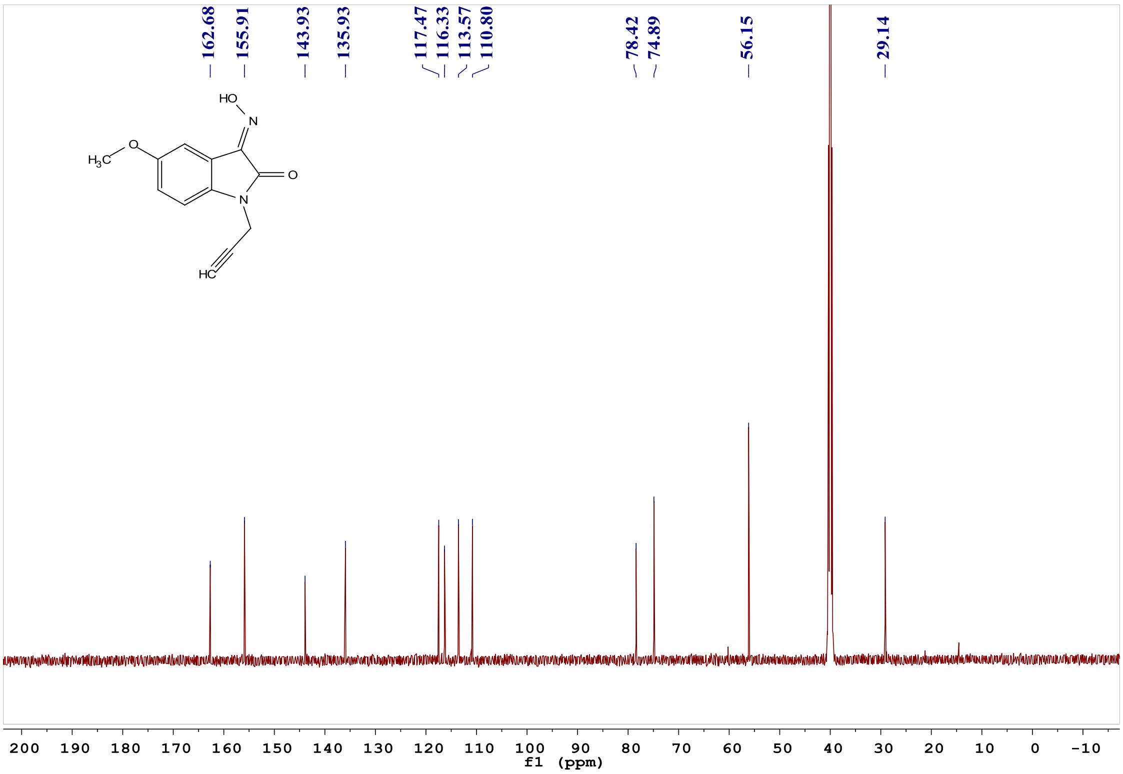

^{1}H NMR of i18

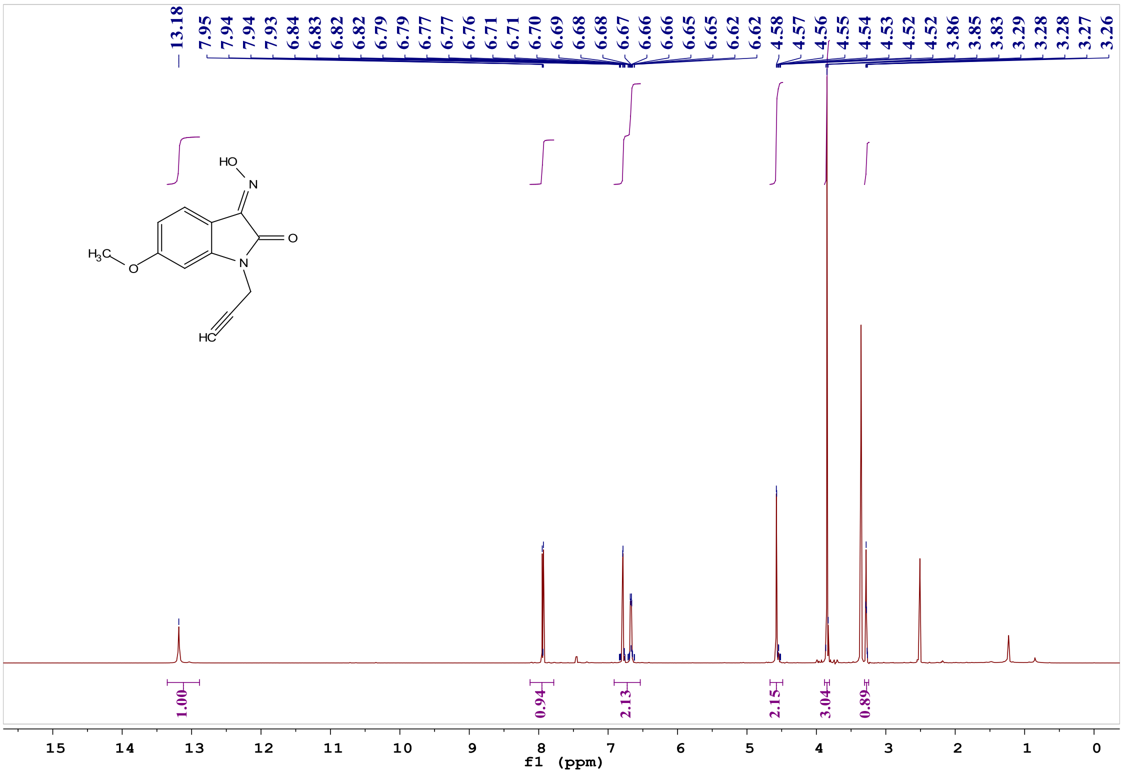

^{13}C NMR of i18

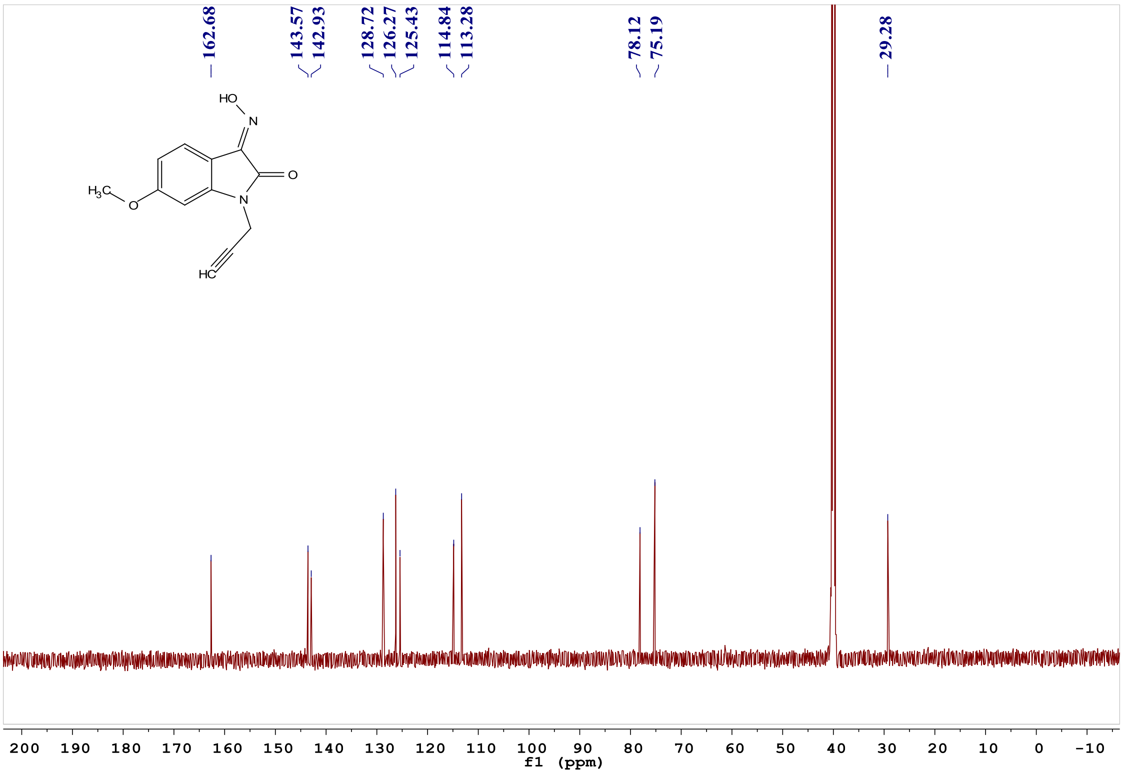

^{1}H NMR of i19

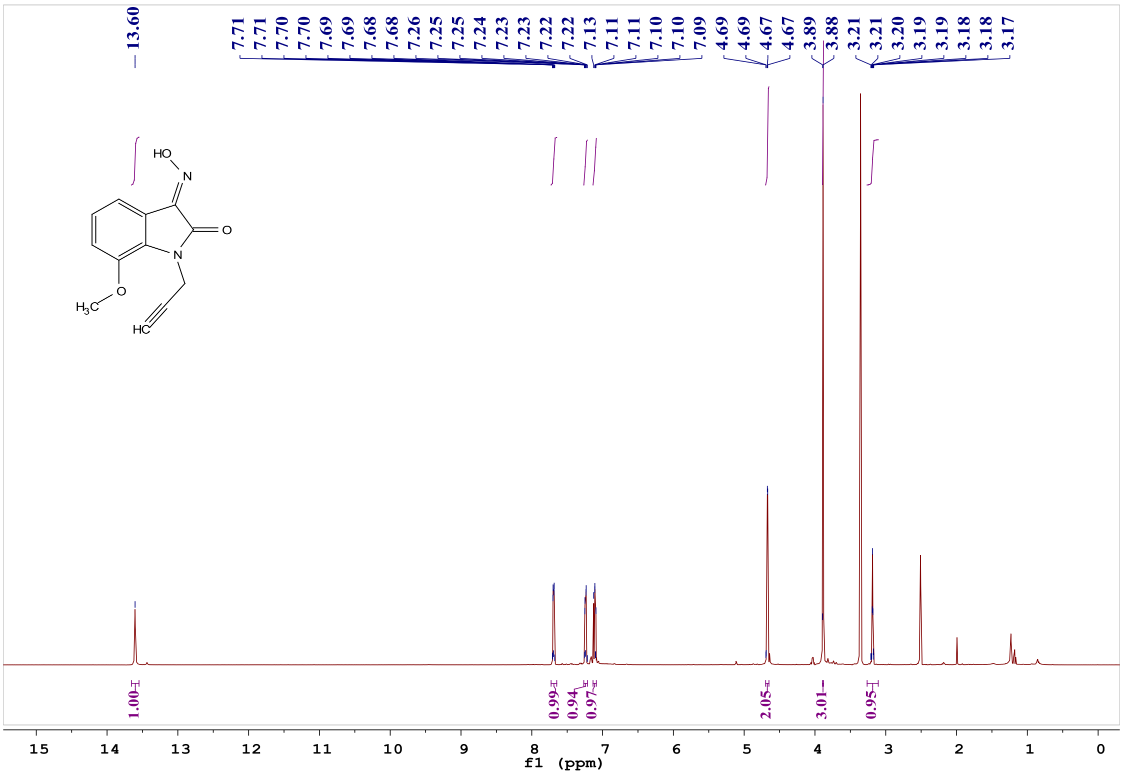

^{13}C NMR of i19

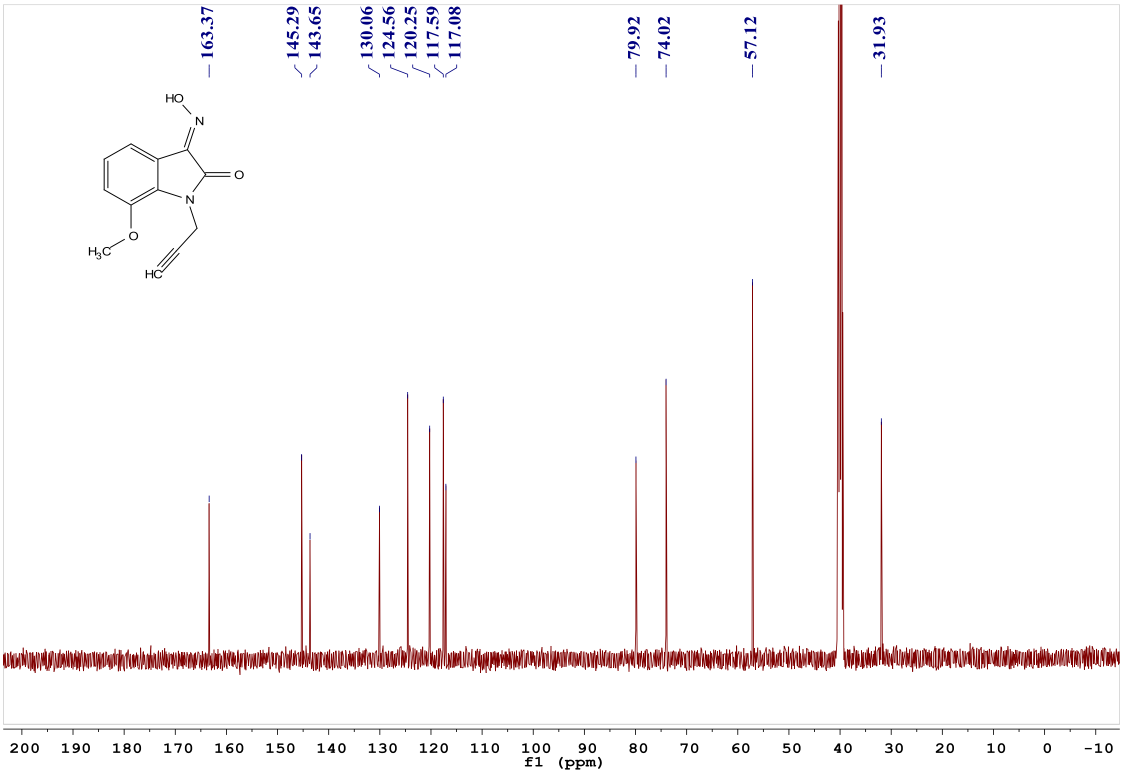

^{1}H NMR of i20

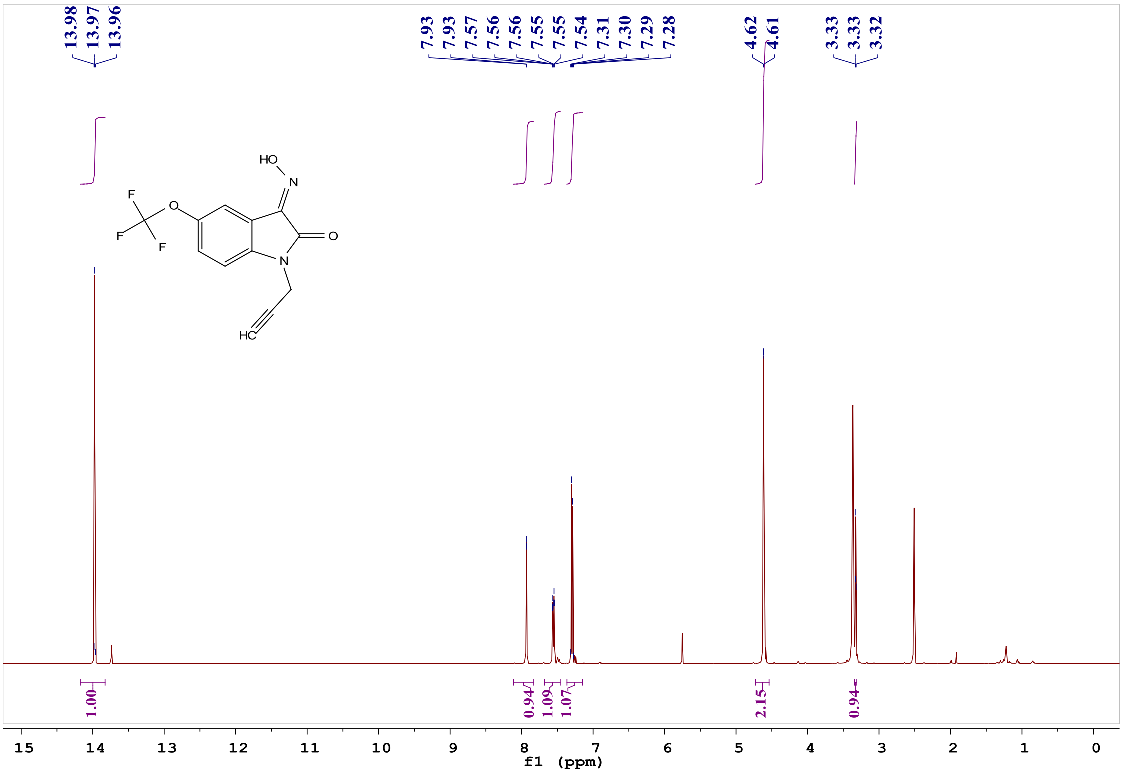

^{13}C NMR of i20

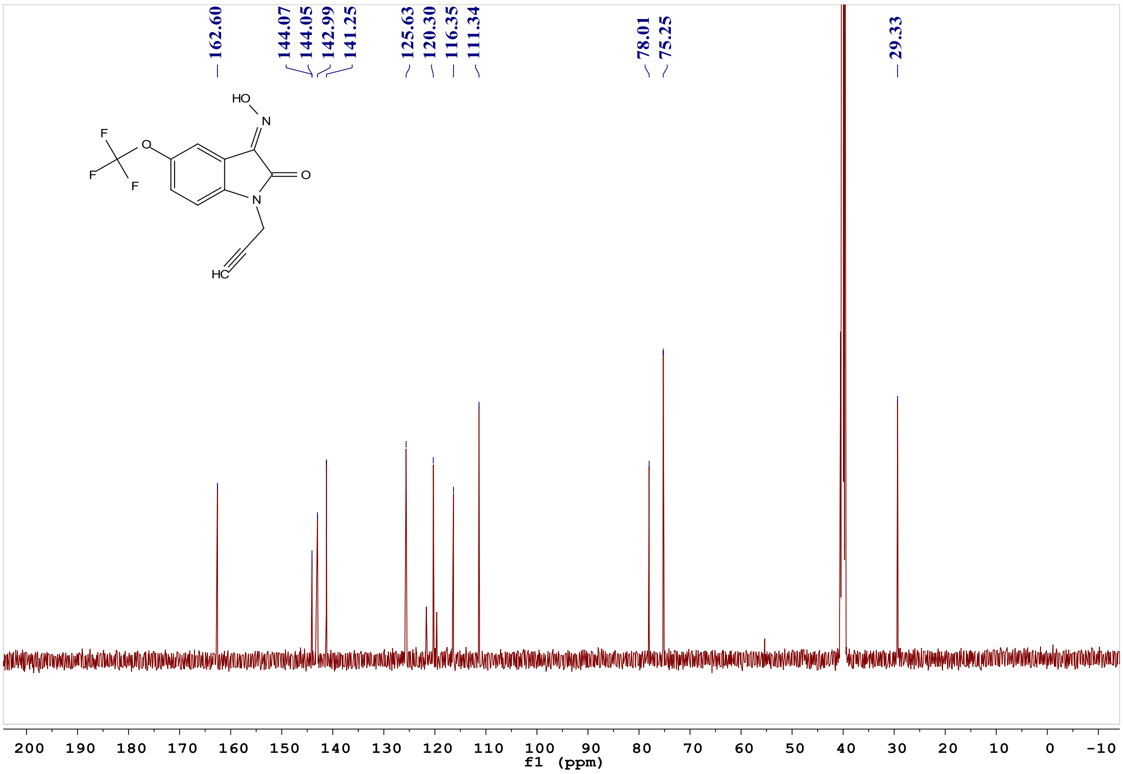

^{1}H NMR of O1as

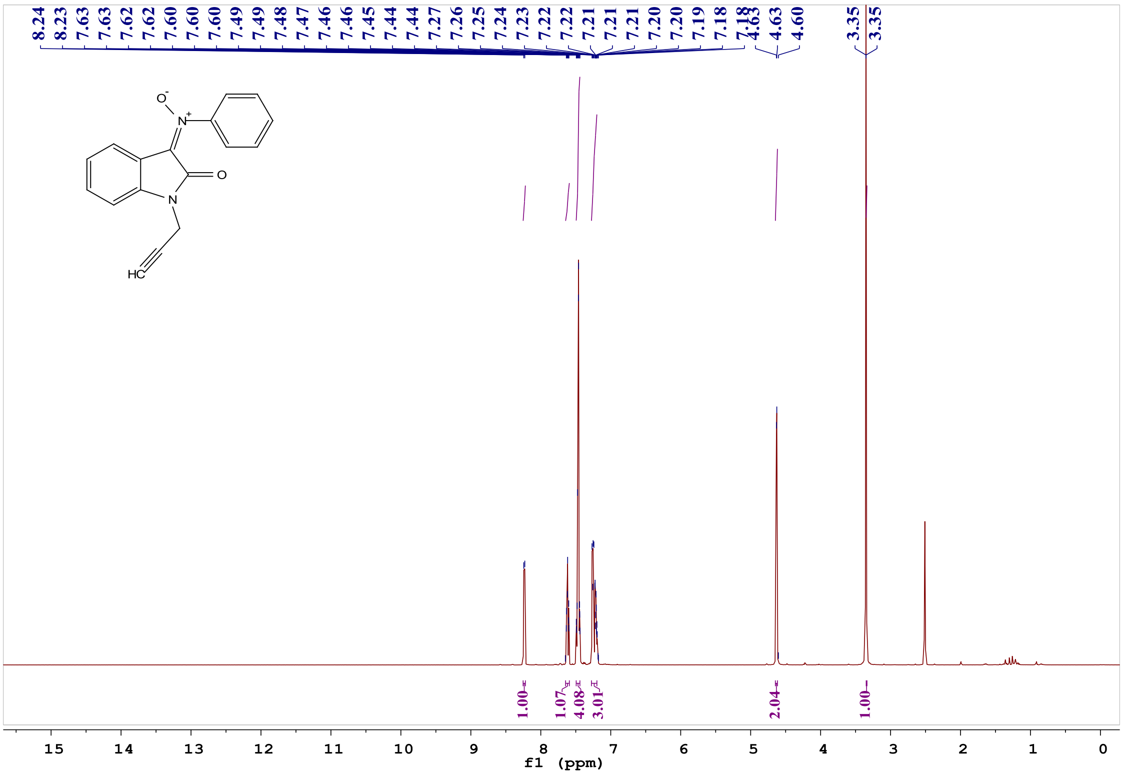

^{13}C NMR of O1as

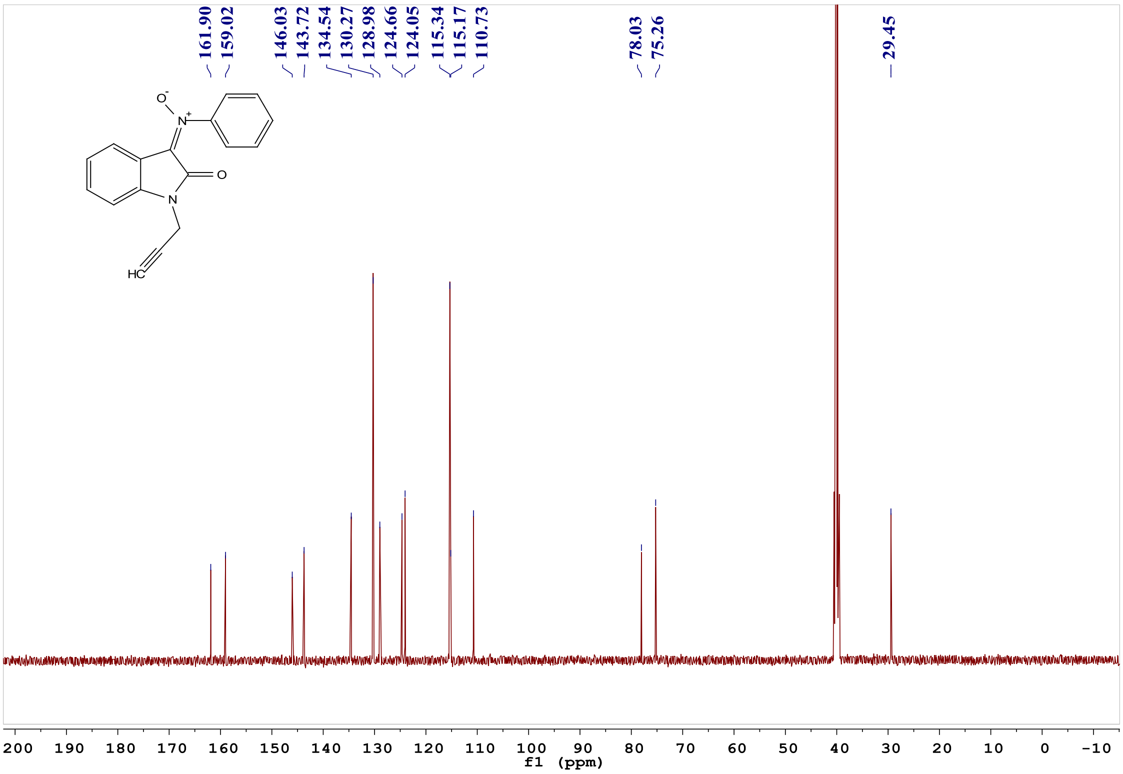

LC Trace and Mass of HP1

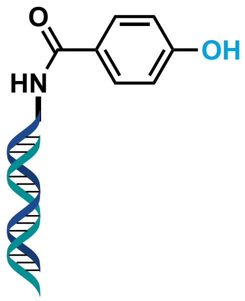

Yield: 100%

Exact mass: 5057.33

Triply charged mass [M]/3 - 1.00794, calculated 1684.7691; observed 1684.3510.

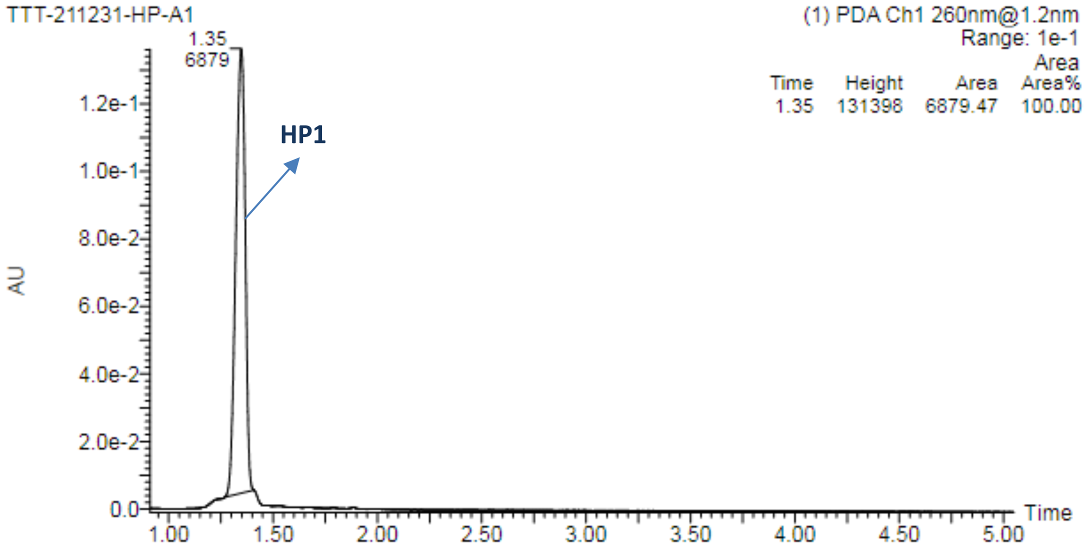

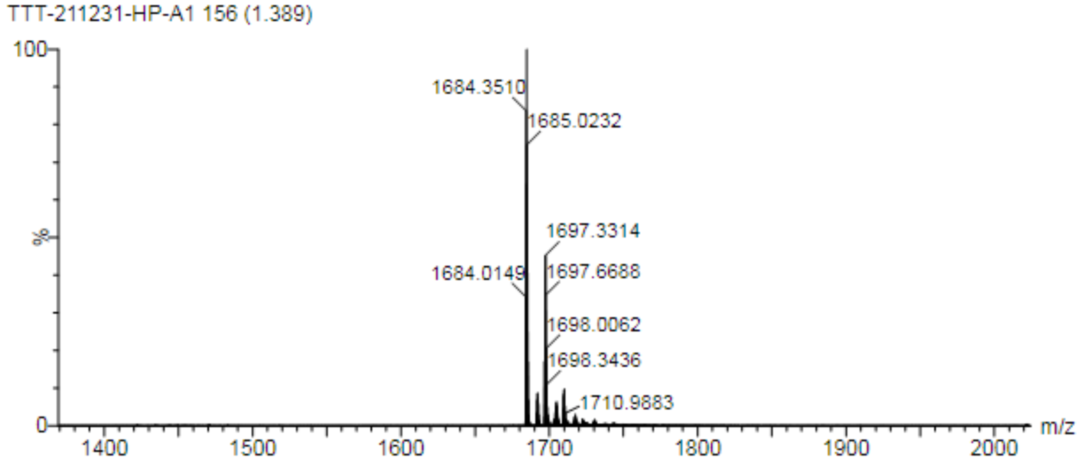

LC Trace and Mass of HP2

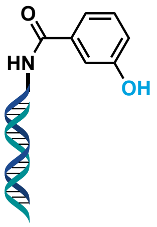

Yield: 100%

Exact mass: 5057.33

Triply charged mass [M]/3 - 1.00794, calculated 1684.7691; observed 1684.8660.

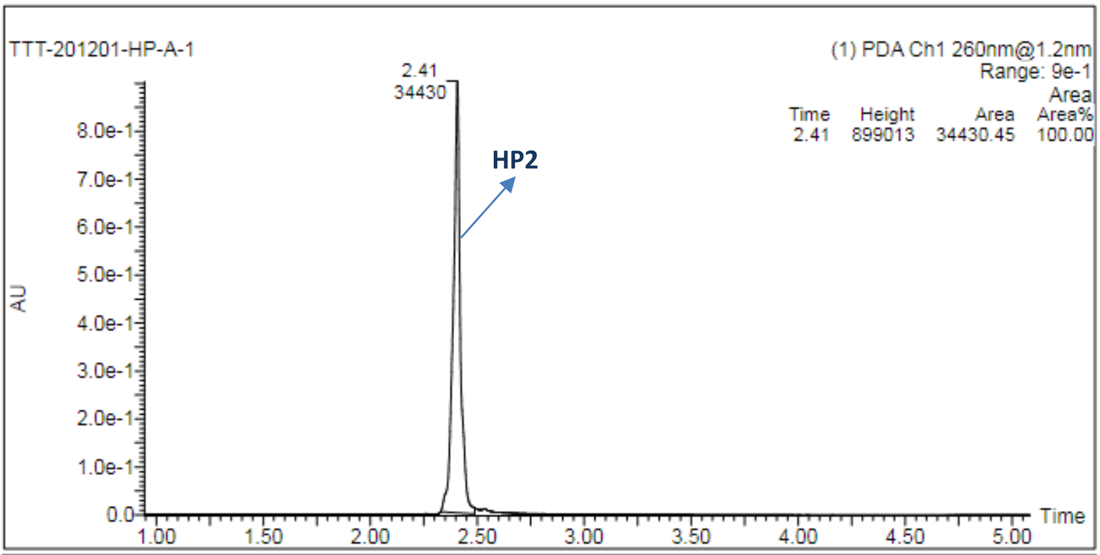

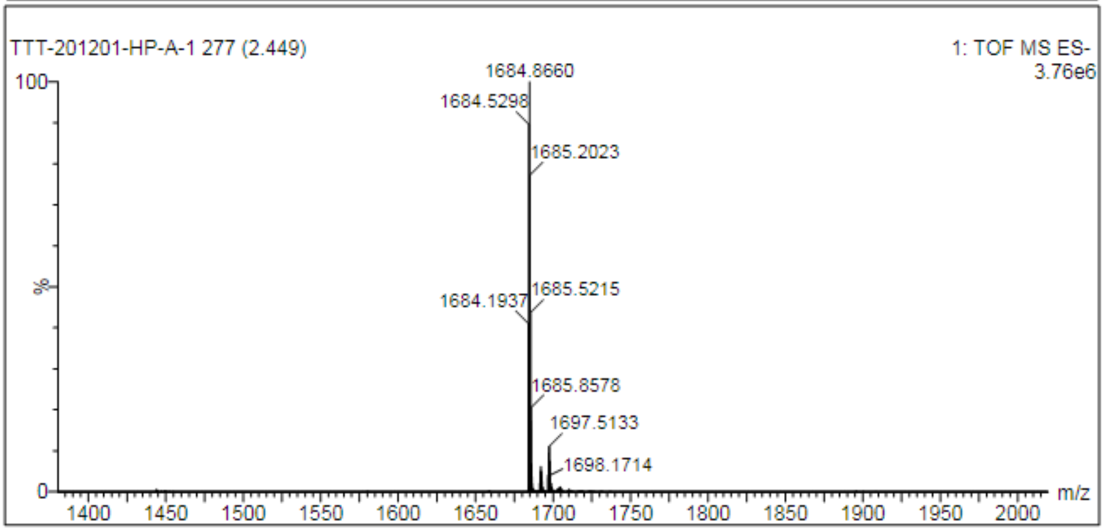

LC Trace and Mass of HP3

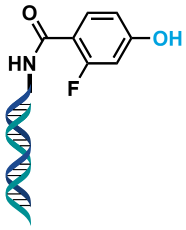

Yield: 96%

Exact mass: 5075.33

Triply charged mass [M]/3 - 1.00794, calculated 1690.76906; observed 1690.8722.

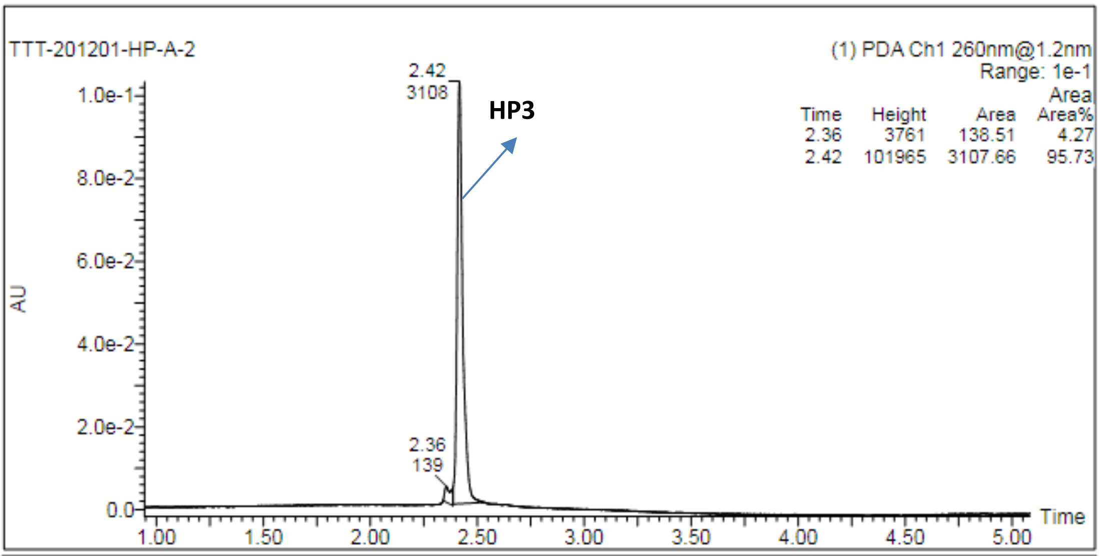

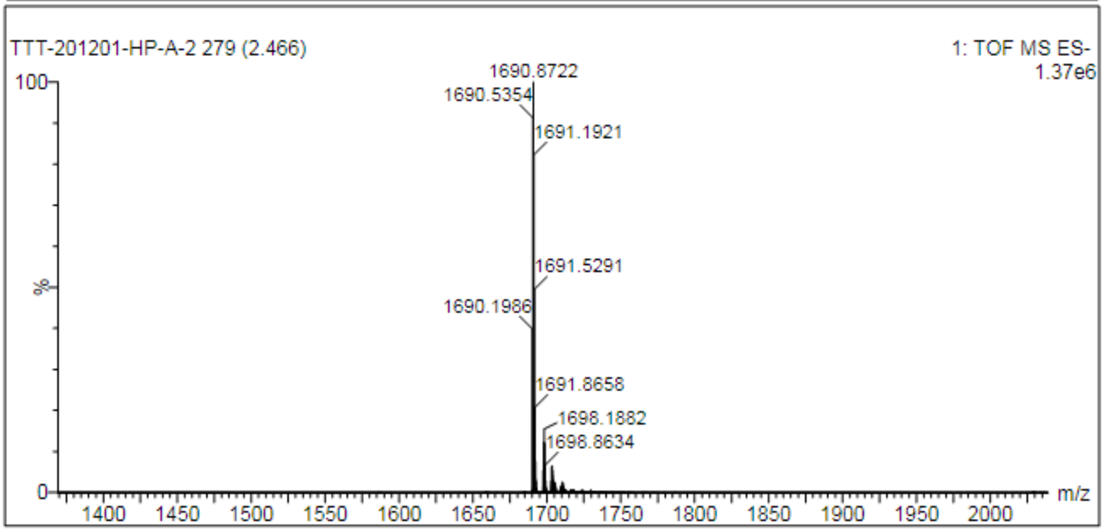

LC Trace and Mass of HP4

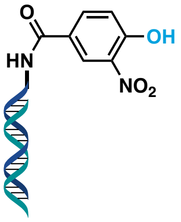

Yield: 92%

Exact mass: 5102.33

Triply charged mass [M]/3 - 1.00794, calculated 1699.76906; observed 1699.8594.

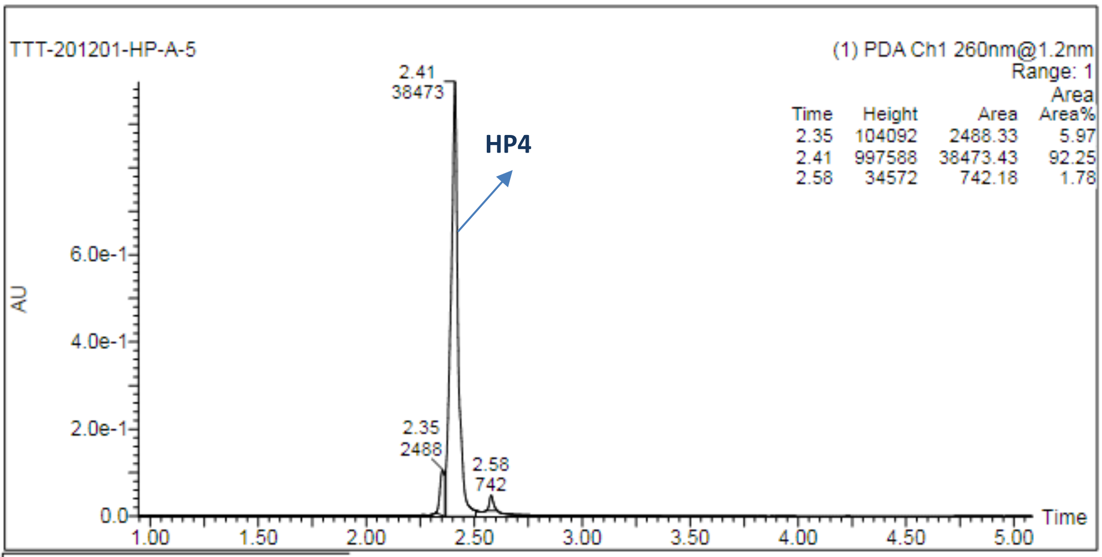

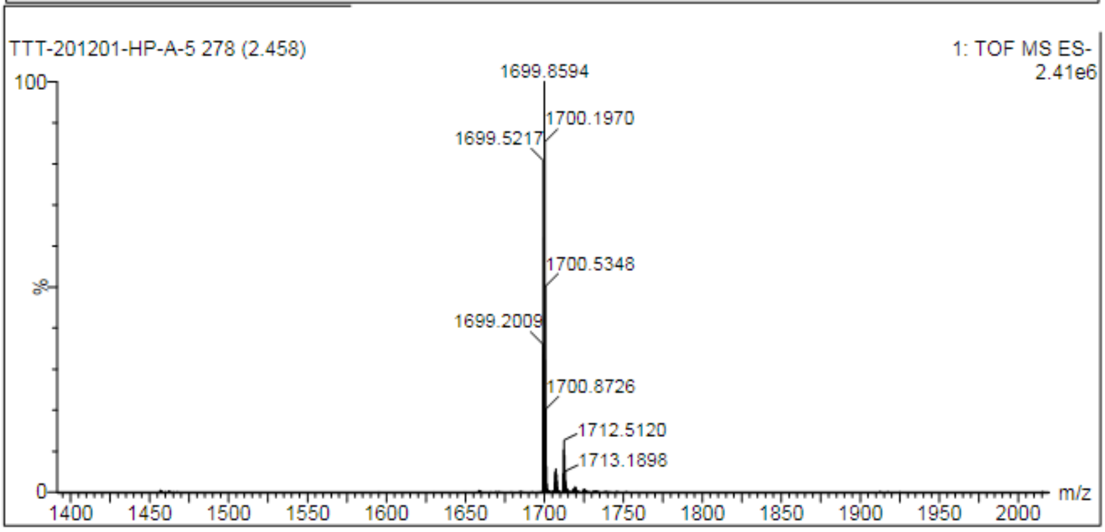

LC Trace and Mass of HP5

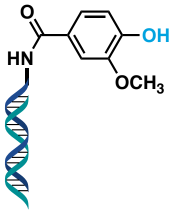

Yield: 100%

Exact mass: 5087.33

Triply charged mass [M]/3 - 1.00794, calculated 1694.76906; observed 1694.8822.

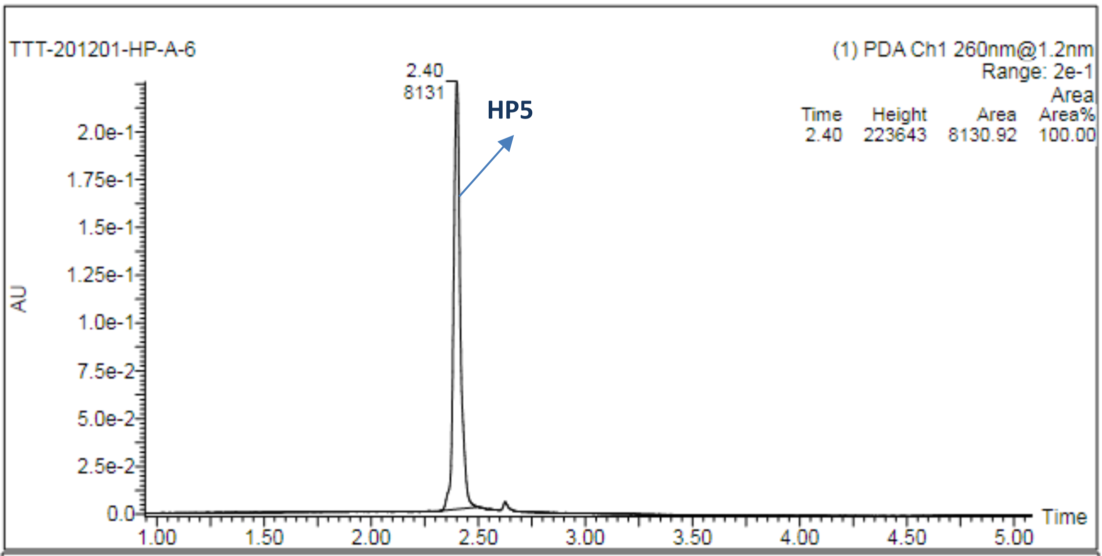

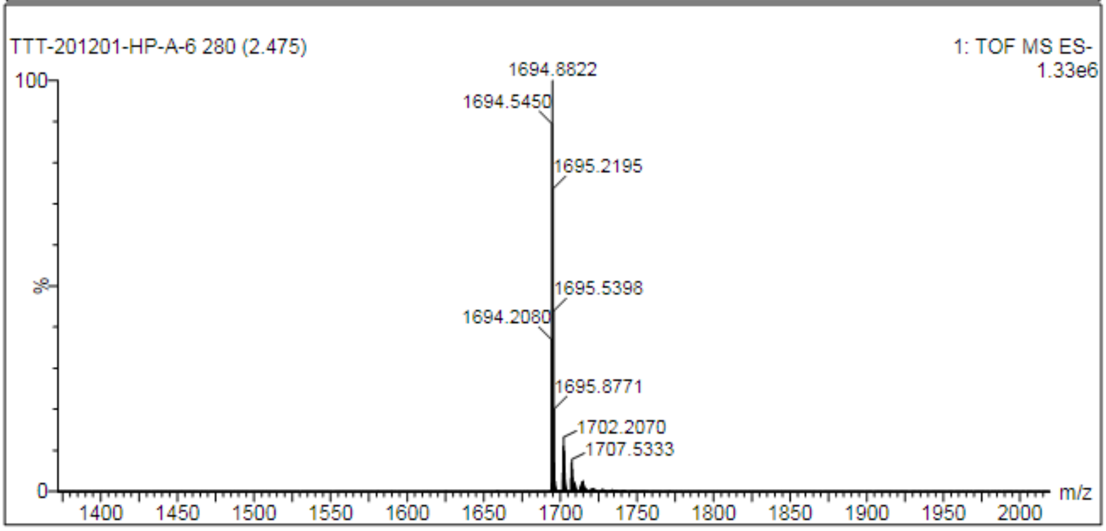

LC Trace and Mass of HP6

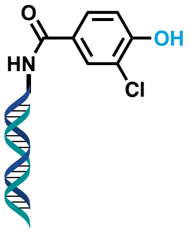

Yield: 96%

Exact mass: 5091.83

Triply charged mass [M]/3 - 1.00794, calculated 1696.26906; observed 1696.1976.

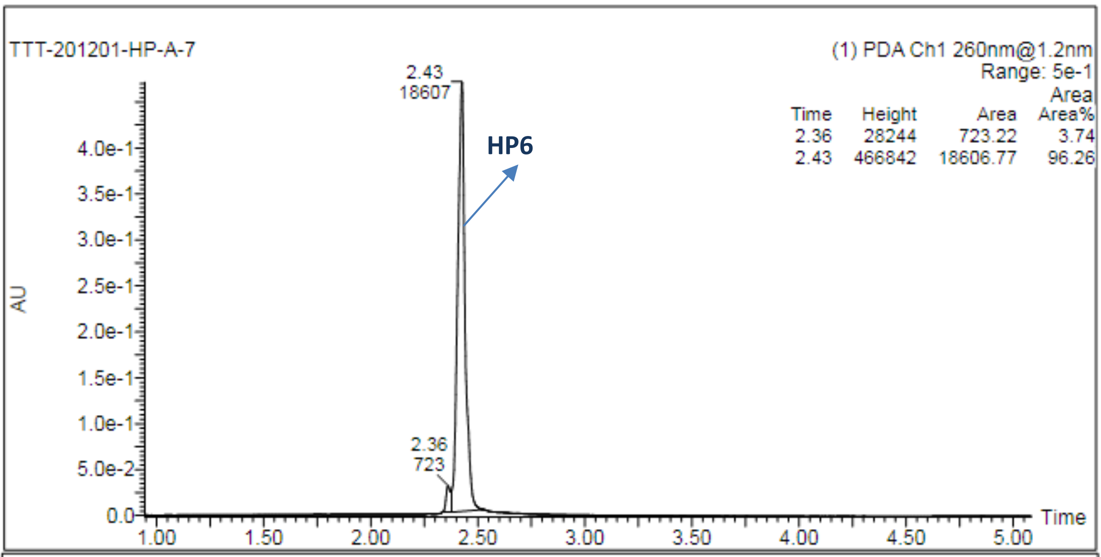

Time	Height	Area	Area%
2.36	28244	723.22	3.74
2.43	466842	18606.77	96.26

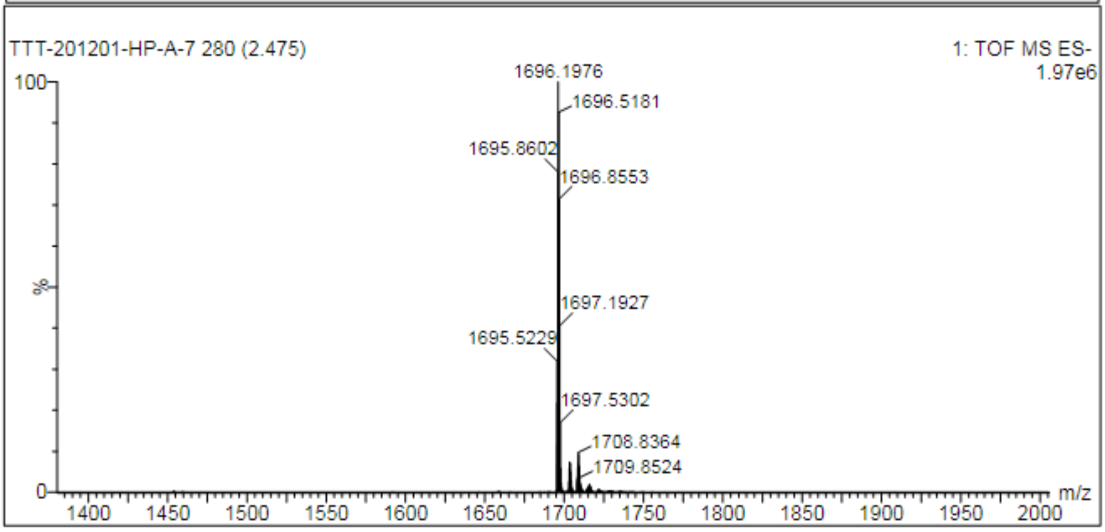

LC Trace and Mass of HP7

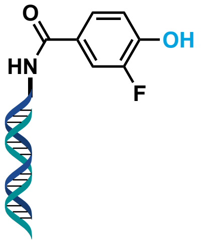

Yield: 100%

Exact mass: 5075.33

Triply charged mass [M]/3 - 1.00794, calculated 1690.76906; observed 1690.8722.

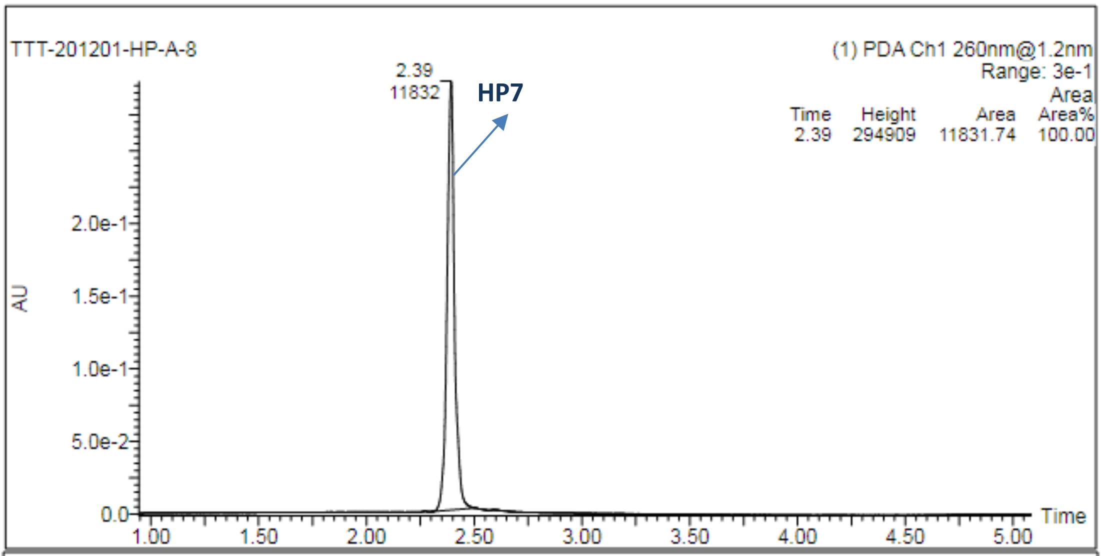

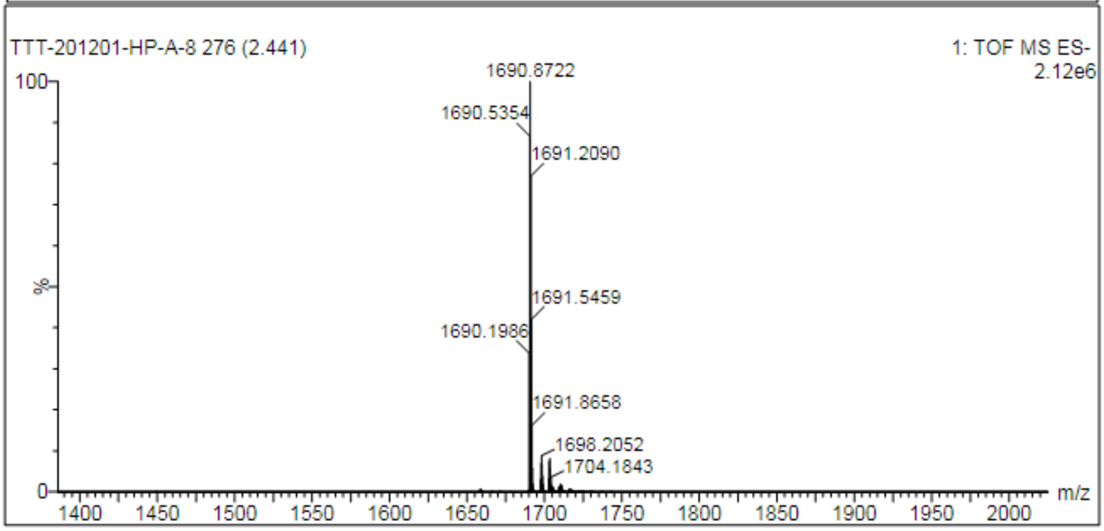

LC Trace and Mass of HP8

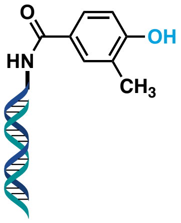

Yield: 96%

Exact mass: 5071.33

Triply charged mass [M]/3 - 1.00794, calculated 1689.43573; observed 1689.7273.

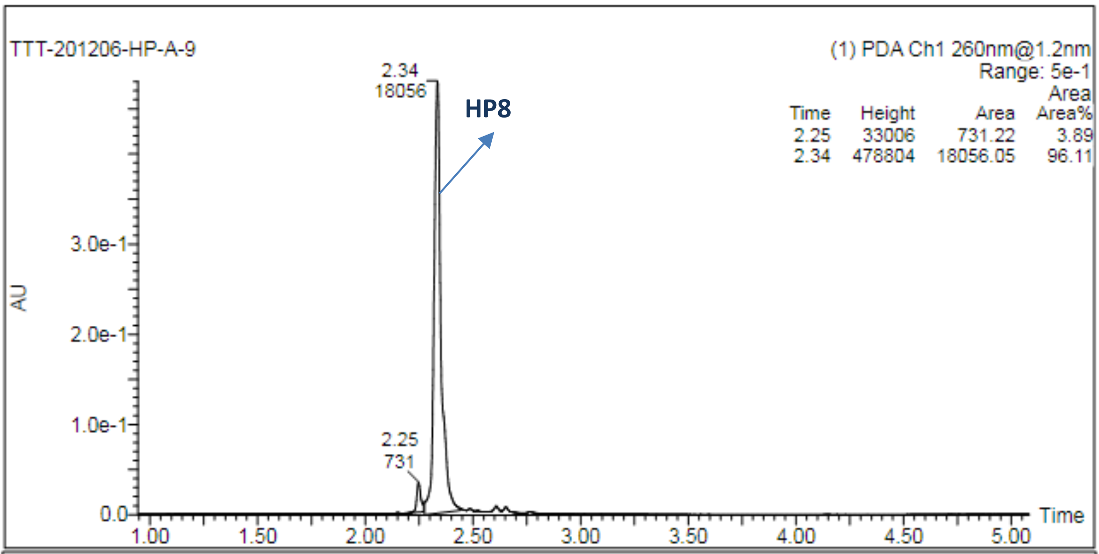

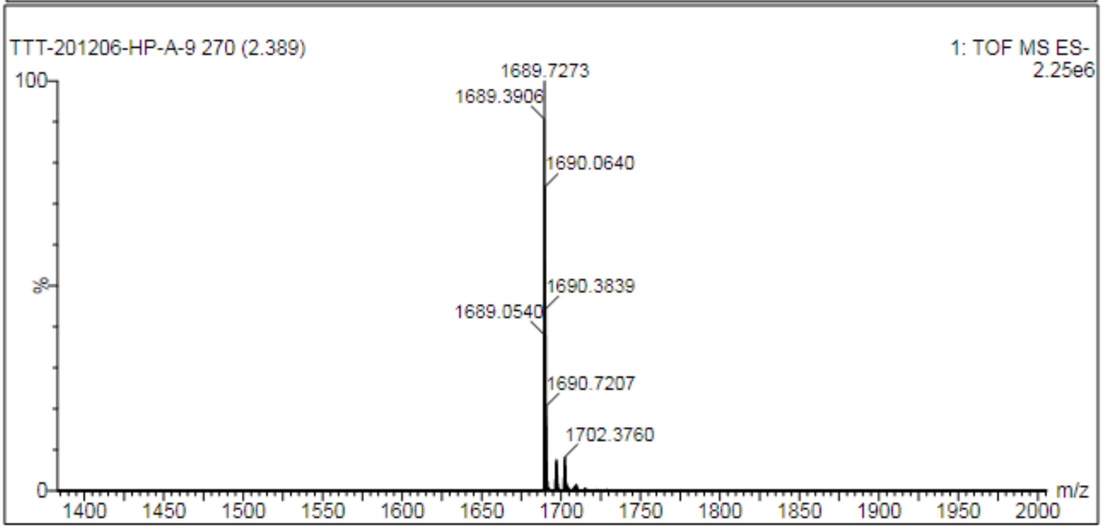

LC Trace and Mass of HP9

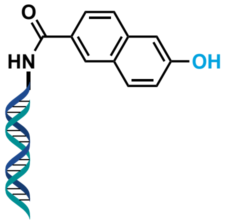

Yield: 98%

Exact mass: 5107.43

Triply charged mass [M]/3 - 1.00794, calculated 1701.46906; observed 1701.5481.

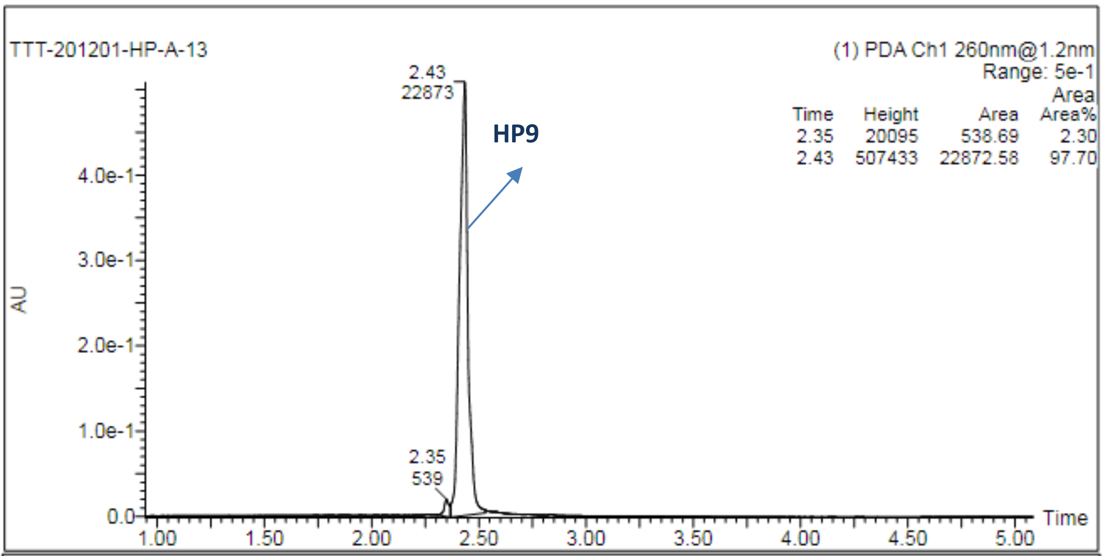

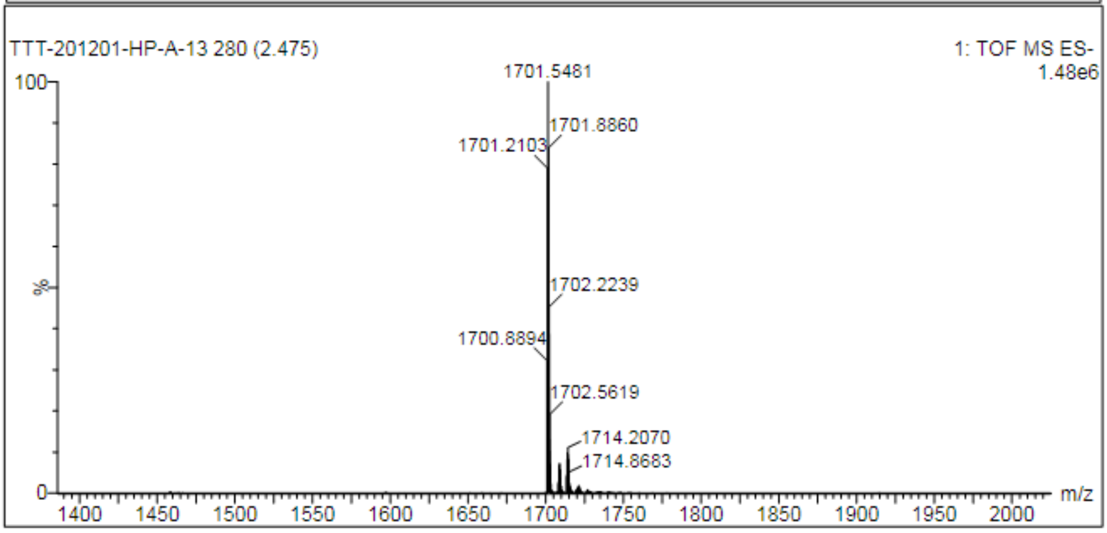

LC Trace and Mass of HP10

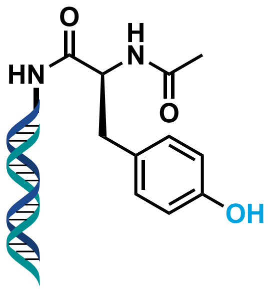

Yield: 98%

Exact mass: 5142.461

Triply charged mass [M]/3 - 1.00794, calculated 1713.1457; observed 1713.0042.

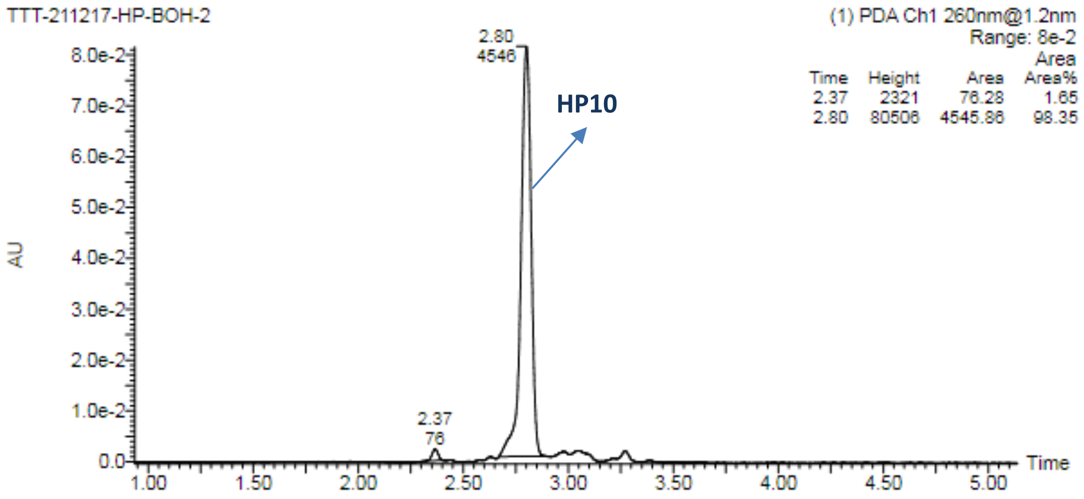

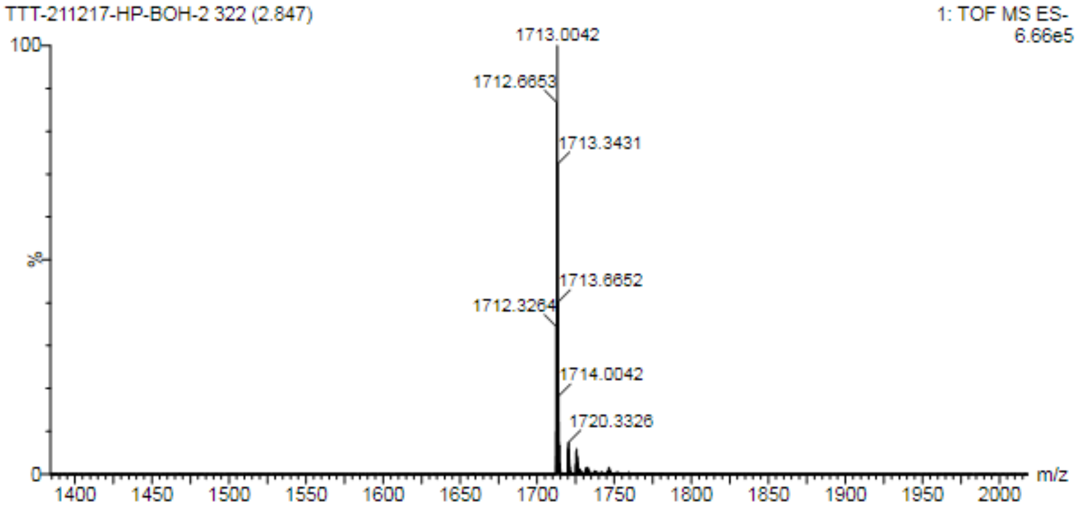

LC Trace and Mass of HP11

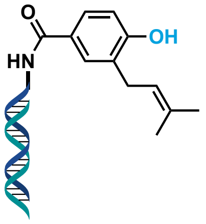

Yield: 76%

Exact mass: 5125.471

Triply charged mass [M]/3 - 1.00794, calculated 1707.4824; observed 1707.2305.

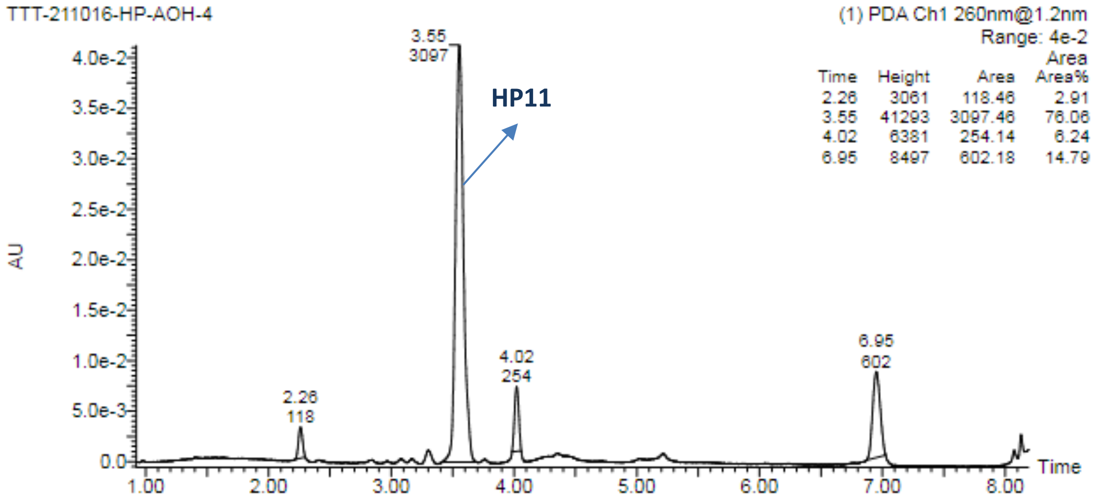

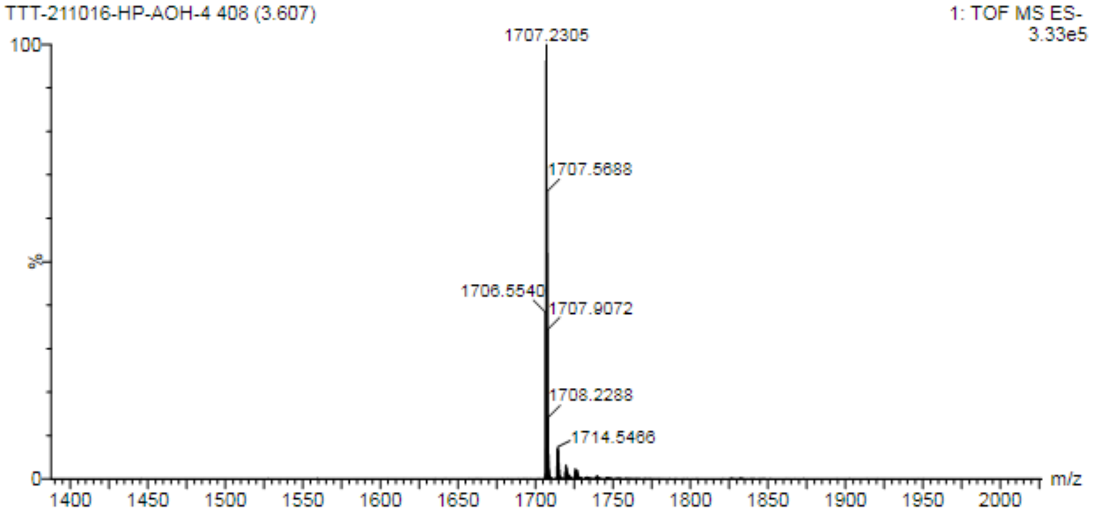

LC Trace and Mass of HP12

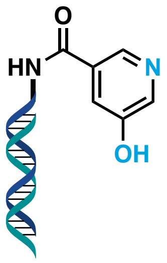

Yield: 96%

Exact mass: 5058.341

Triply charged mass [M]/3 - 1.00794, calculated 1685.1057; observed 1684.9728.

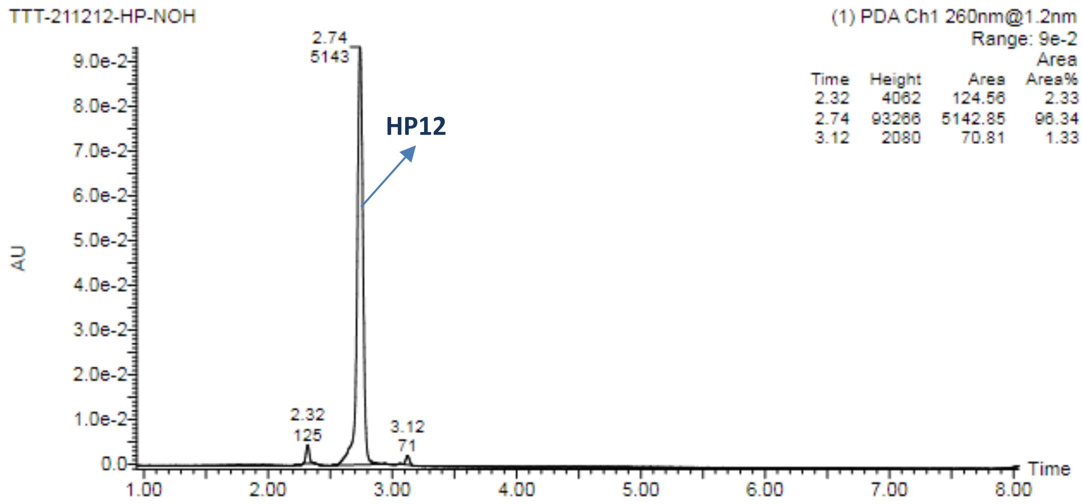

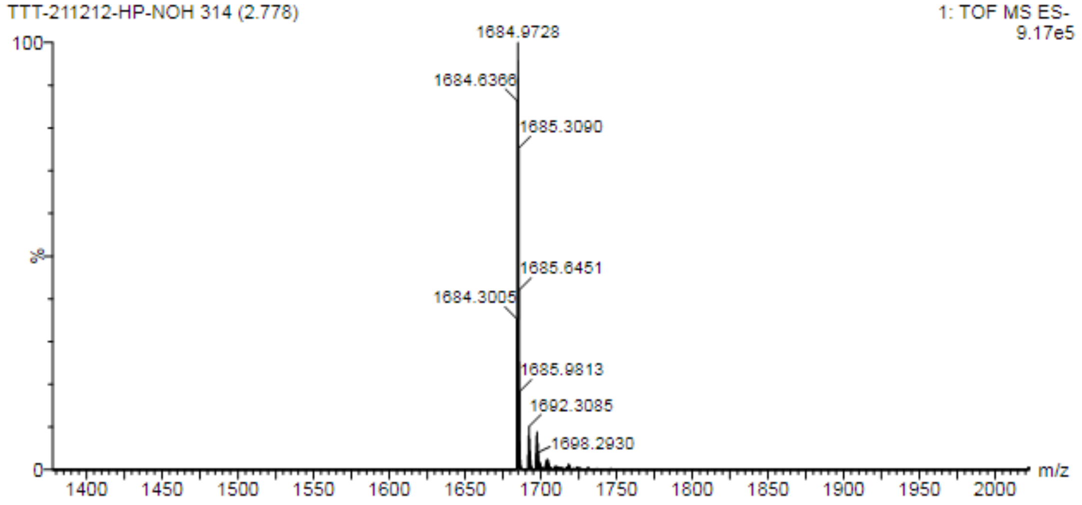

LC Trace and Mass of P1a

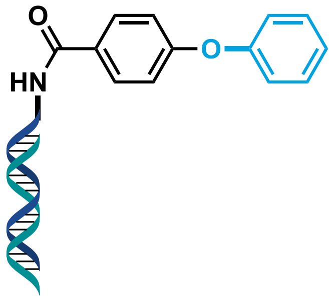

Following General Procedure v

Yield: 87%

Exact mass: 5133.45

Triply charged mass [M]/3 - 1.00794, calculated 1710.14206; observed 1710.1572.

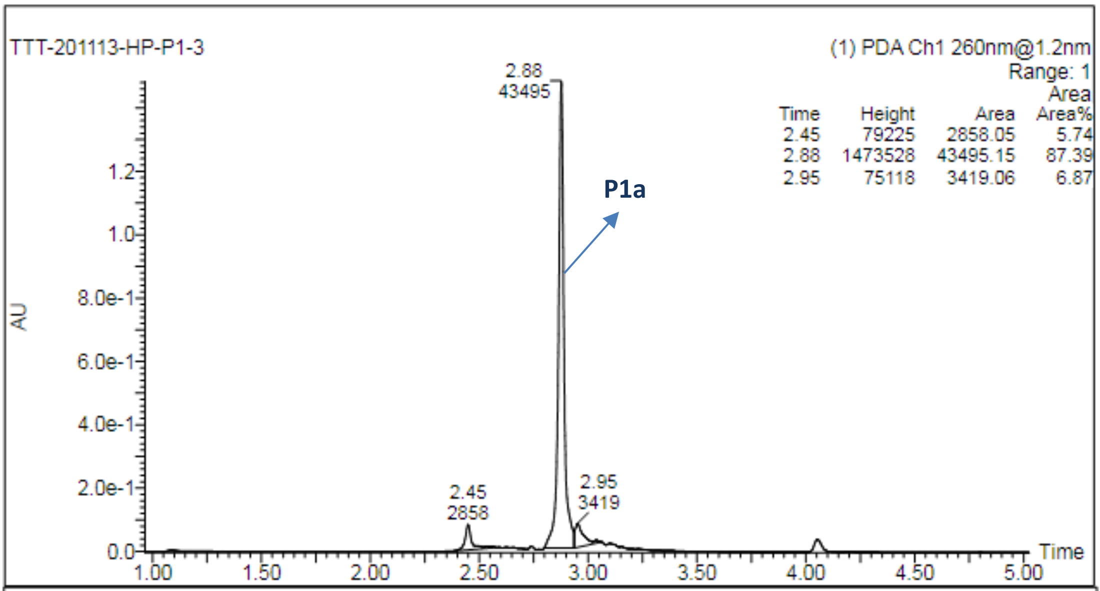

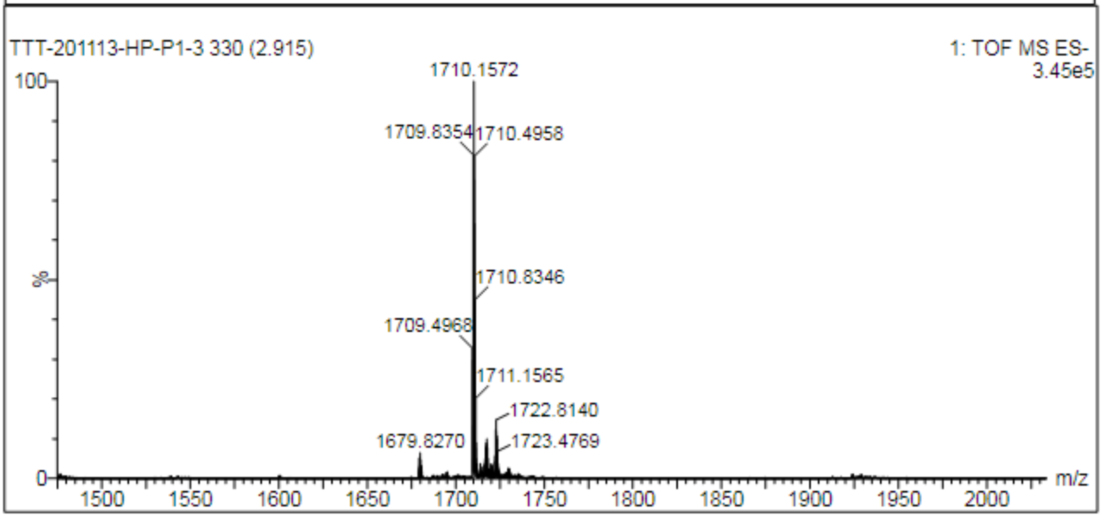

LC Trace and Mass of P1b

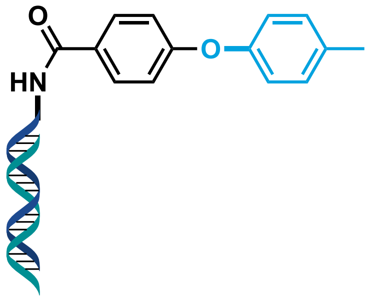

Following General Procedure v

Yield: 81%

Exact mass: 5147.45

Triply charged mass [M]/3 - 1.00794, calculated 1714.80873; observed 1714.8514.

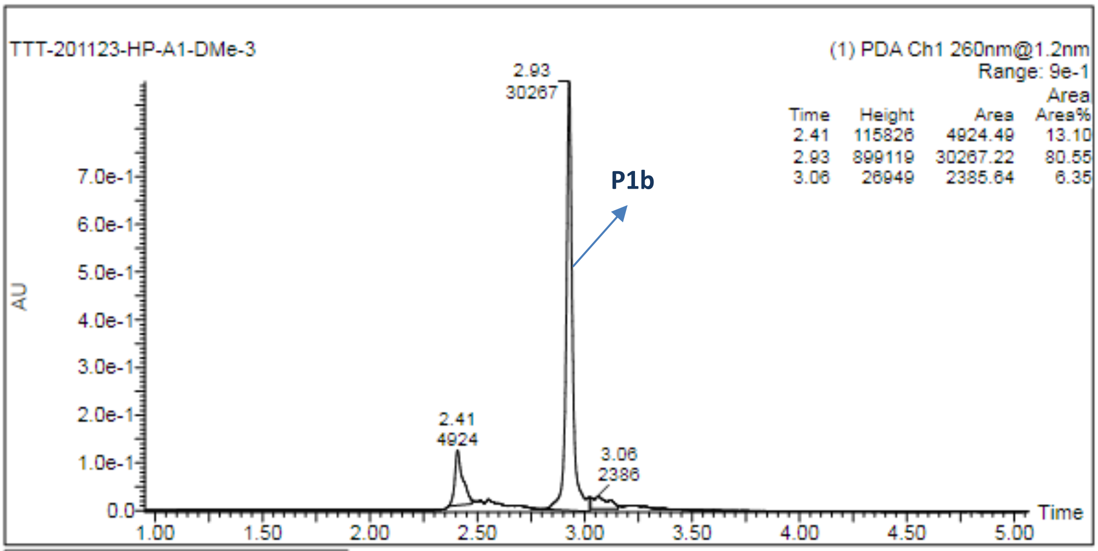

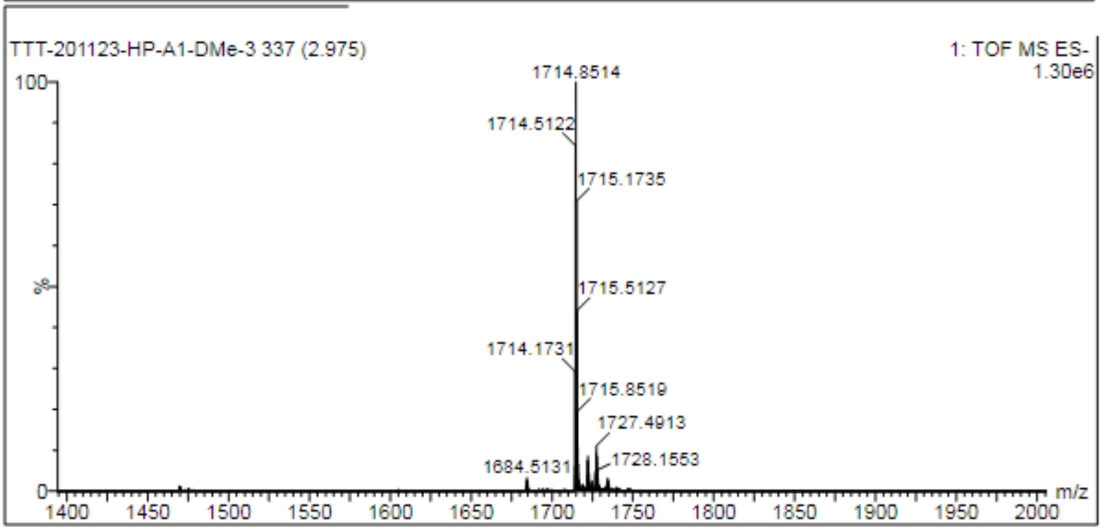

LC Trace and Mass of P1c

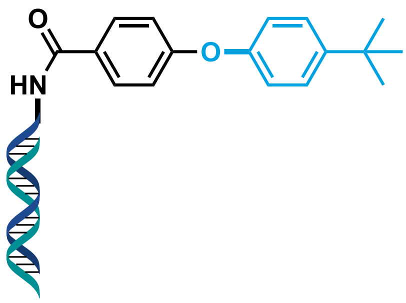

Following General Procedure v

Yield: 90%

Exact mass: 5189.55

Triply charged mass [M]/3 - 1.00794, calculated 1728.84206; observed 1728.5298.

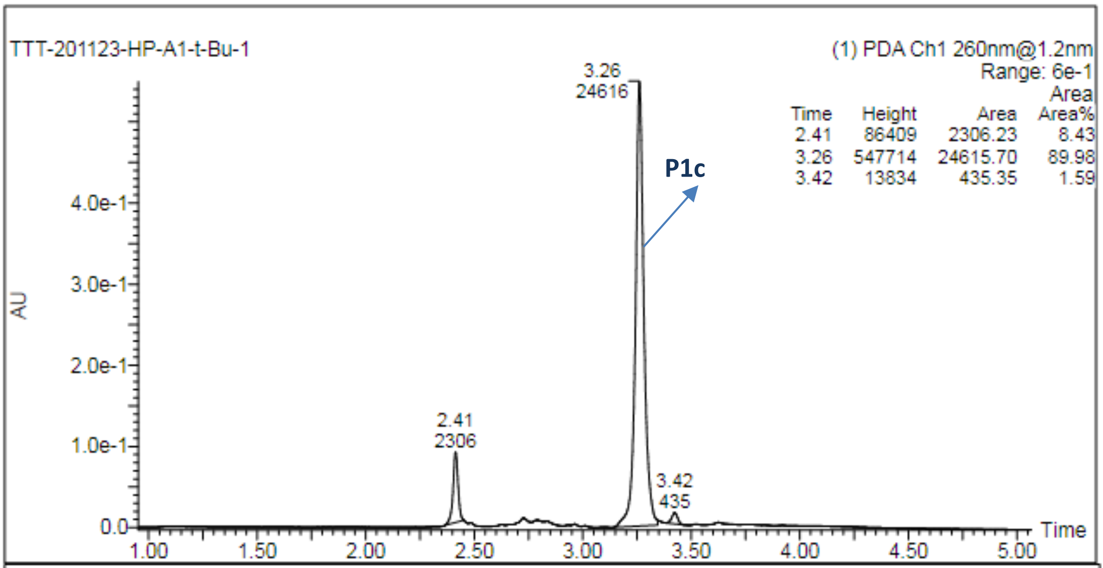

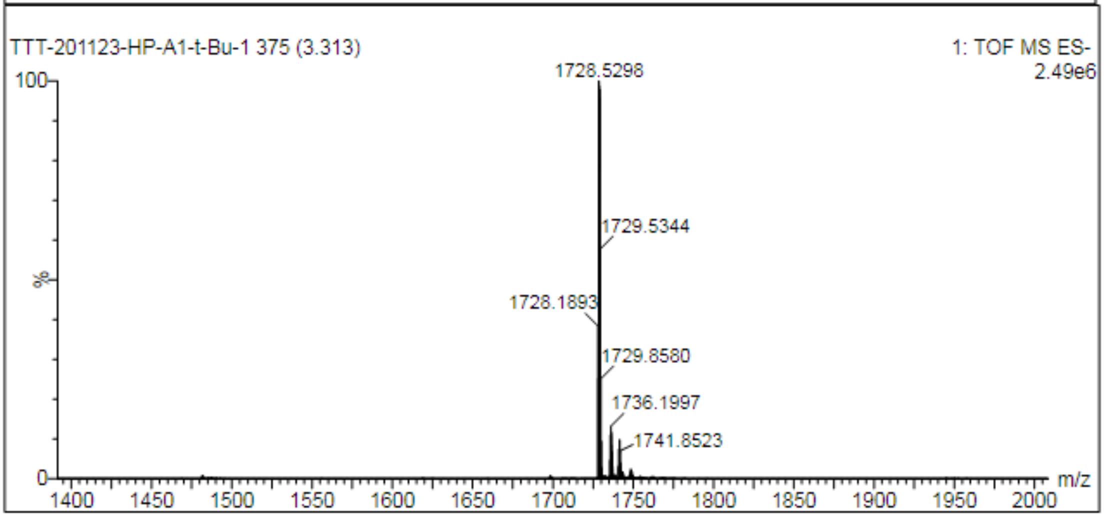

LC Trace and Mass of P1d

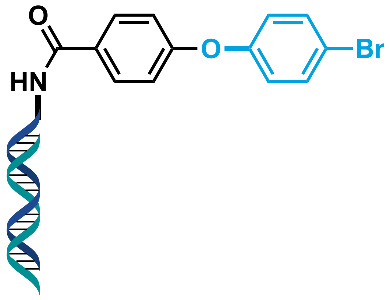

Following General Procedure v

Yield: 68%

Exact mass: 5212.35

Triply charged mass [M]/3 - 1.00794, calculated 1736.44206; observed 1736.4899.

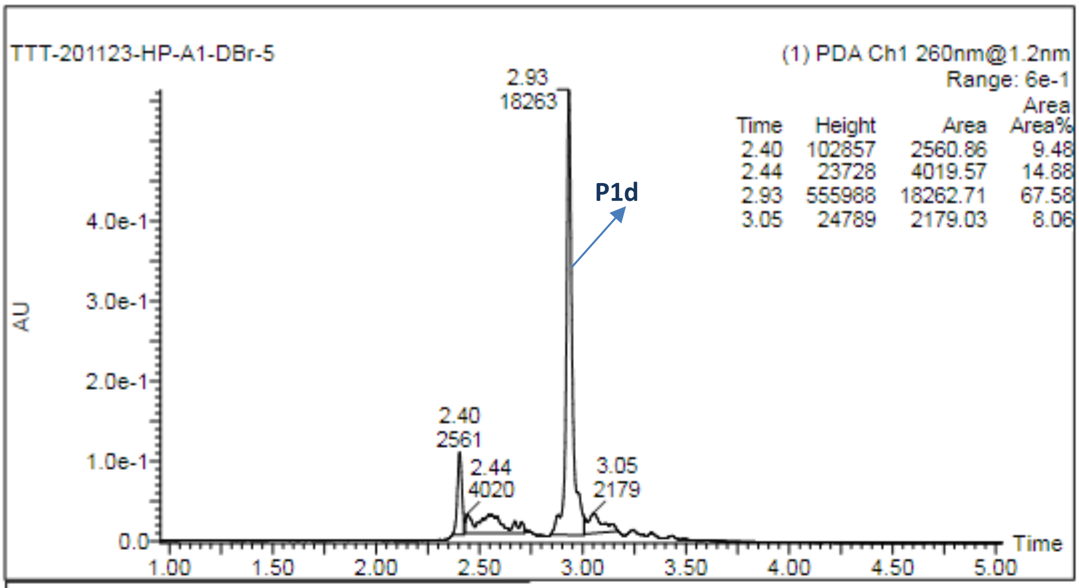

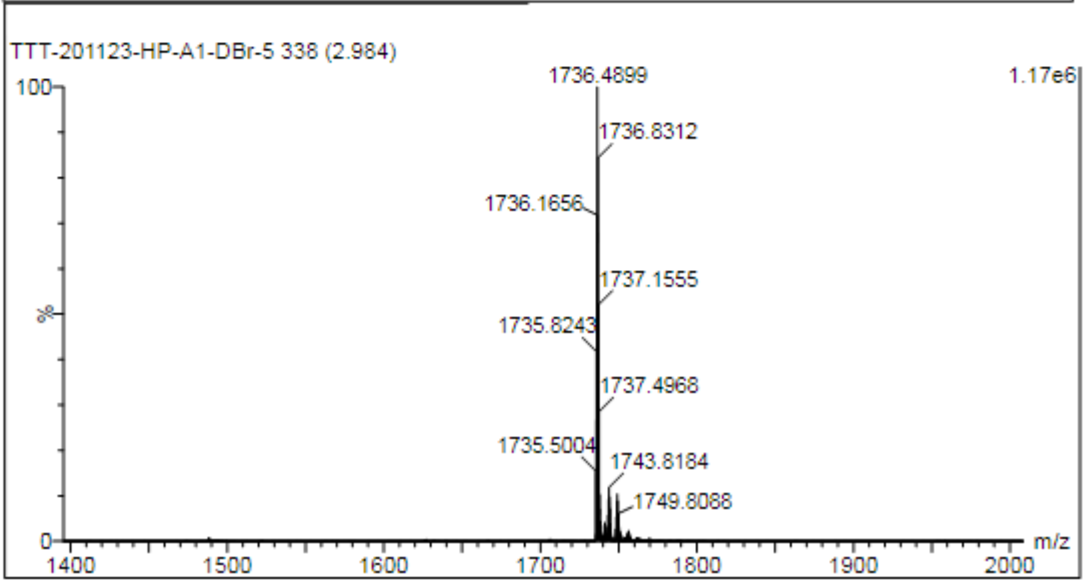

LC Trace and Mass of P1e

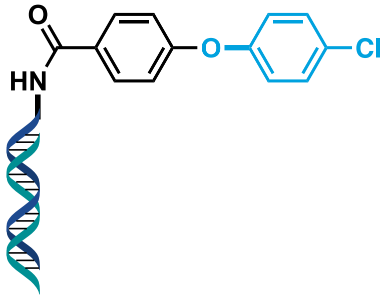

Following General Procedure

Yield: 76%

Exact mass: 5167.90

Triply charged mass [M]/3 - 1.00794, calculated 1721.62539; observed 1721.5054.

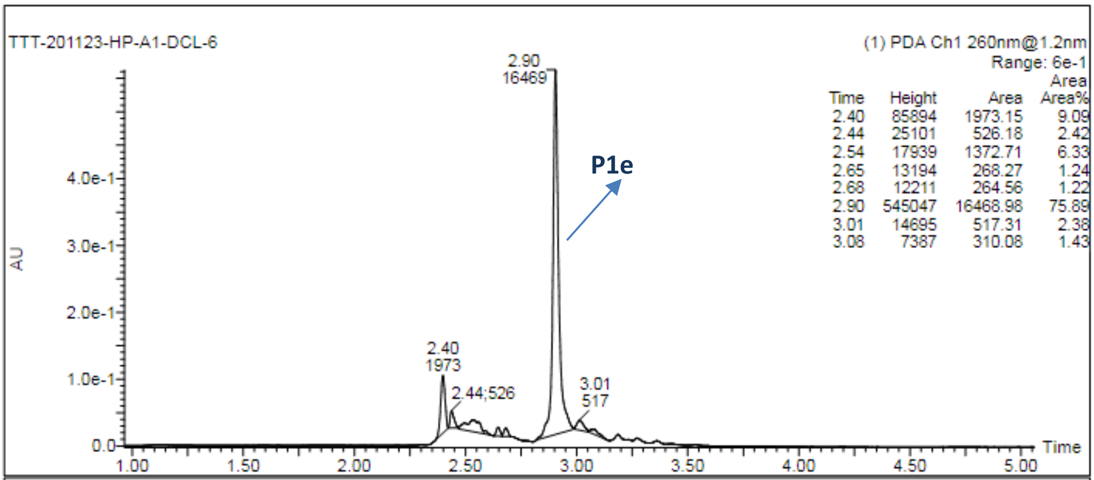

Time	Height	Area	Area%
2.40	85894	1973.15	9.09
2.44	25101	526.18	2.42
2.54	17939	1372.71	6.33
2.65	13194	268.27	1.24
2.68	12211	264.56	1.22
2.90	545047	16468.98	75.89
3.01	14695	517.31	2.38
3.08	7387	310.08	1.43

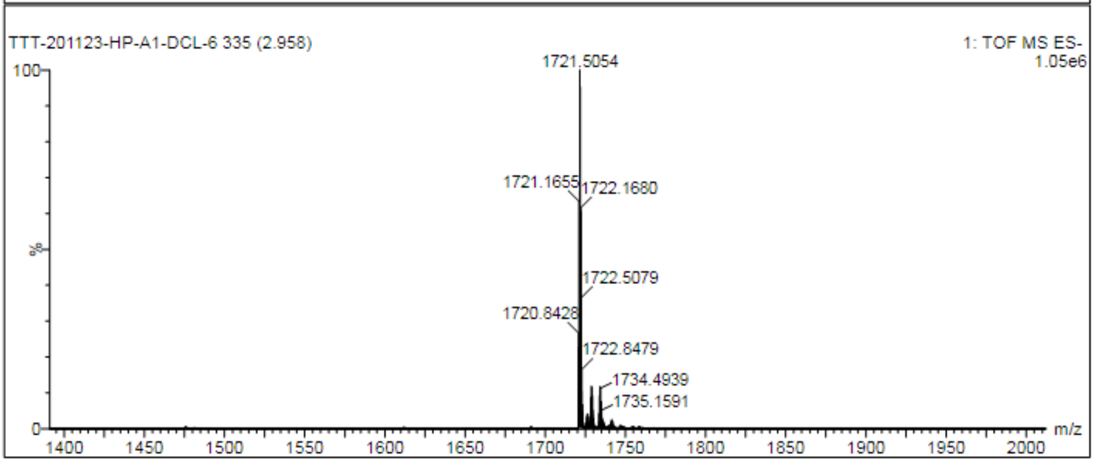

LC Trace and Mass of P1f

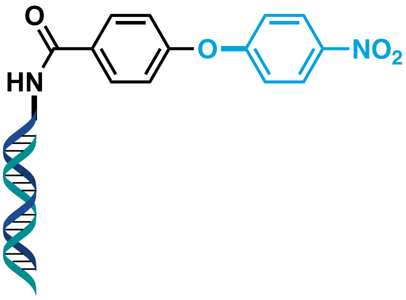

Following General Procedure

Yield: 88%

Exact mass: 5178.45

Triply charged mass [M]/3 - 1.00794, calculated 1725.14206; observed 1725.1943.

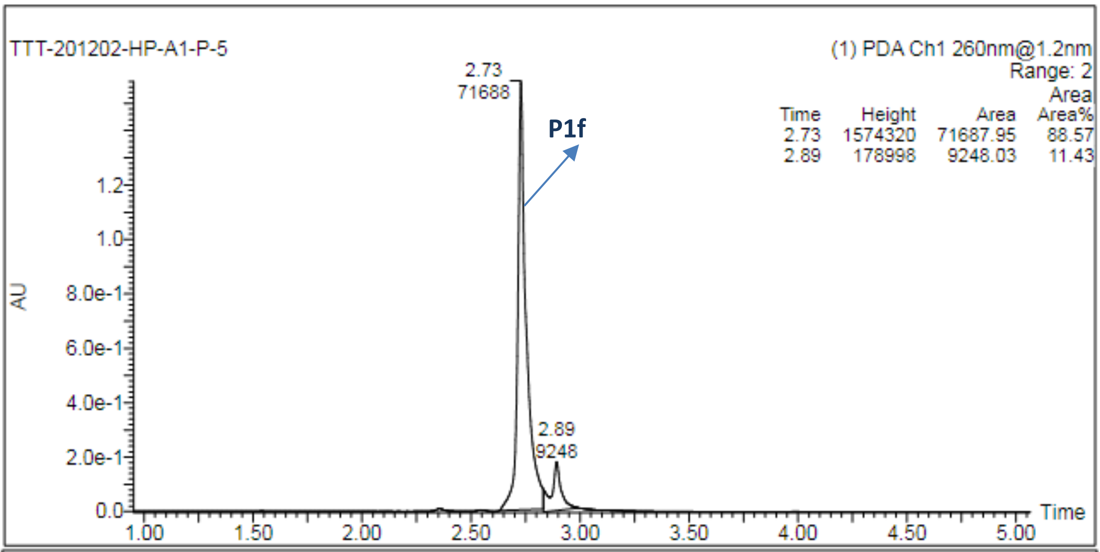

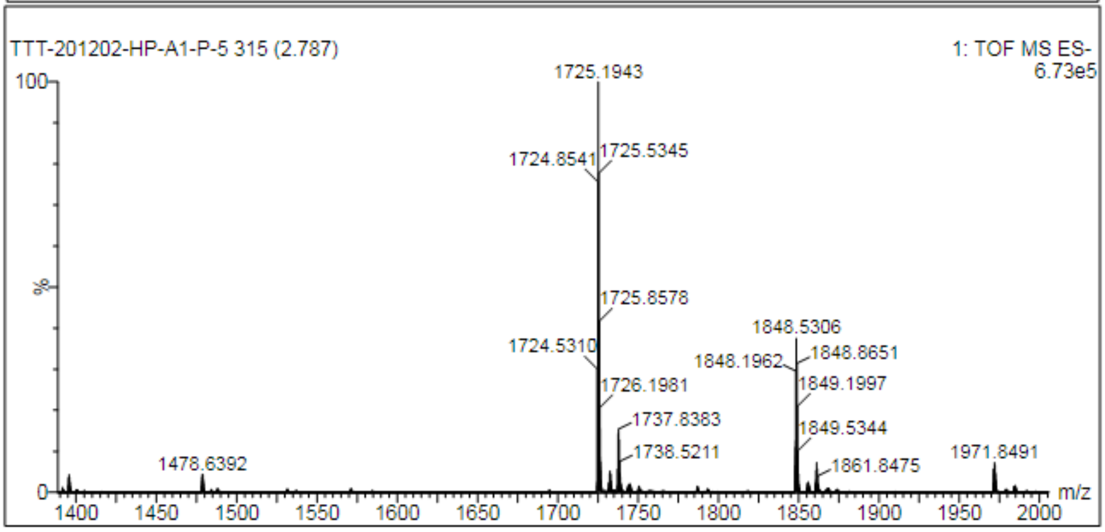

LC Trace and Mass of P1g

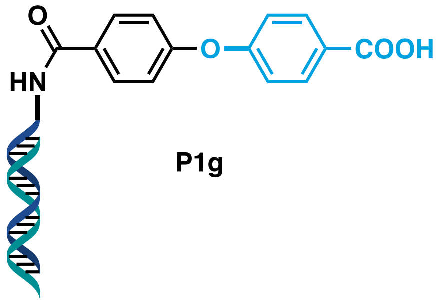

P1g

Following General Procedure

Yield: 86%

Exact mass: 5177.50

Triply charged mass [M]/3 - 1.00794, calculated 1724.82539; observed 1725.0583.

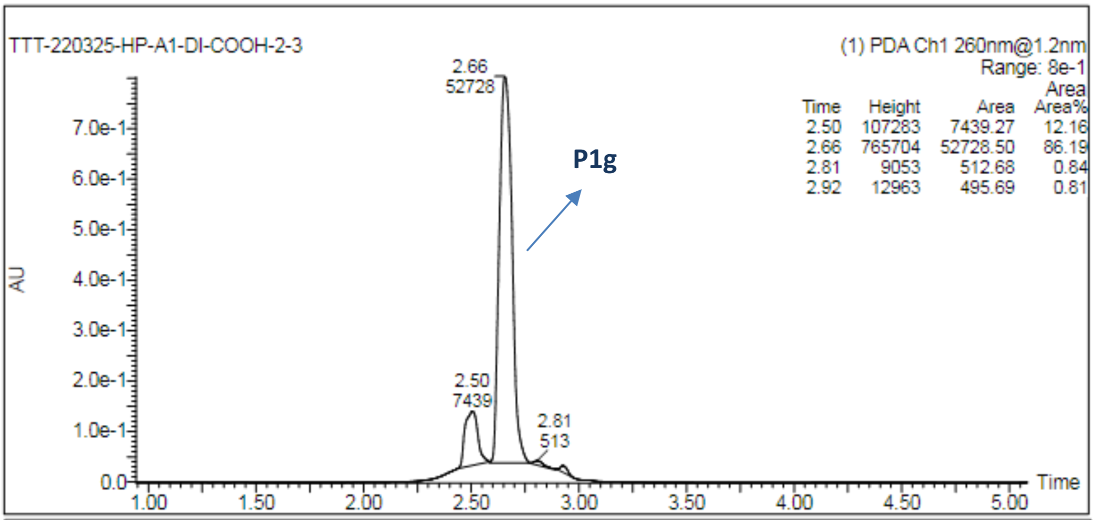

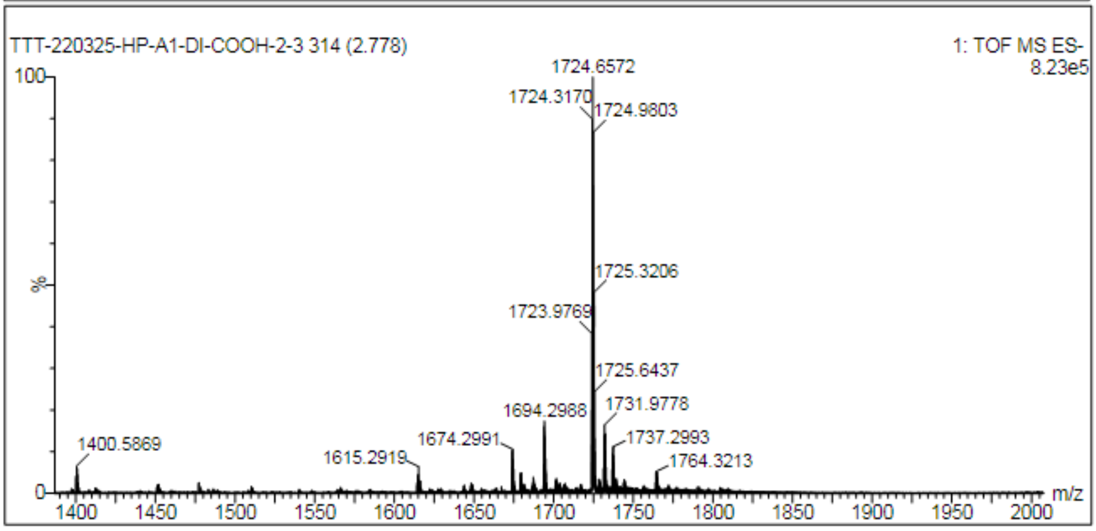

LC Trace and Mass of P1h

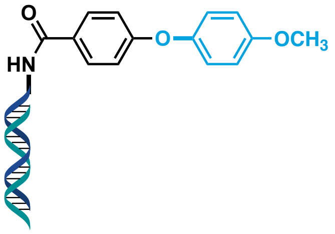

Following General Procedure

Yield: 23%

Exact mass: 5163.50

Triply charged mass [M]/3 - 1.00794, calculated 1720.15873; observed 1720.4011.

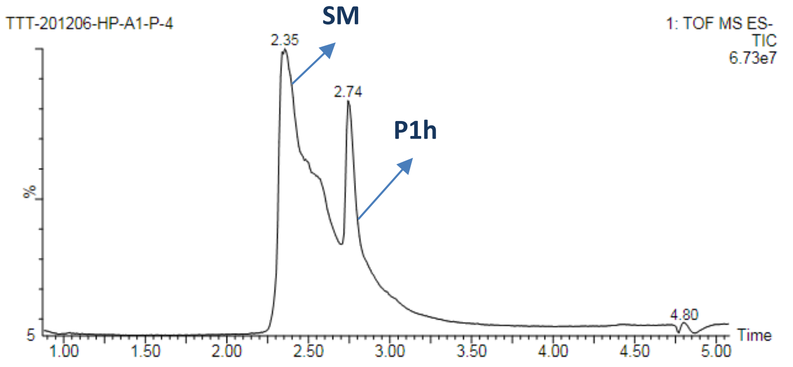

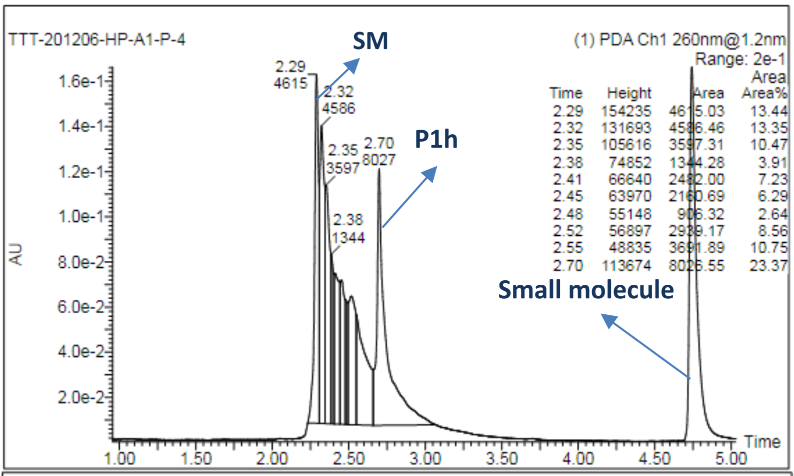

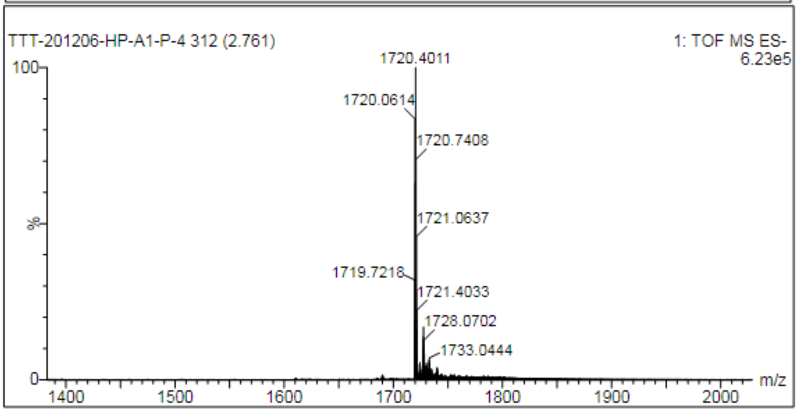

LC Trace and Mass of P1i

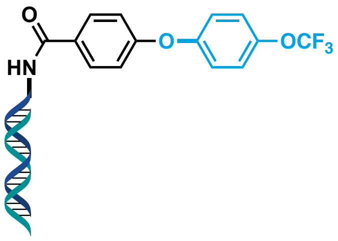

Following General Procedure

Yield: 67%

Exact mass: 5217.45

Triply charged mass [M]/3 - 1.00794, calculated 1738.14206; observed 1738.3845.

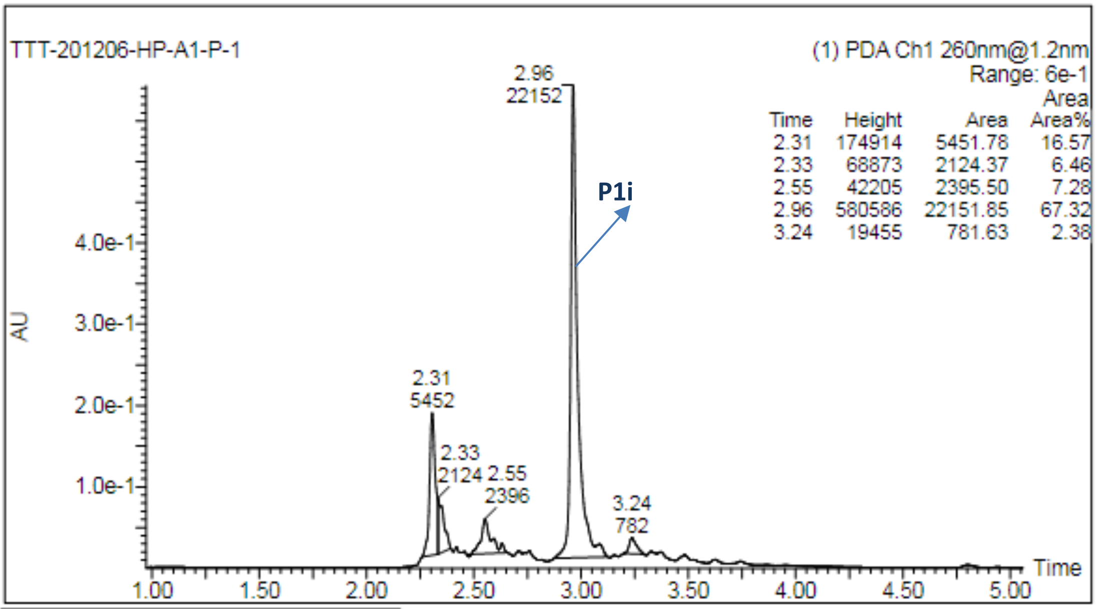

Time	Height	Area	Area%
2.31	174914	5451.78	16.57
2.33	68873	2124.37	6.46
2.55	42205	2395.50	7.28
2.96	580586	22151.85	67.32
3.24	19455	781.63	2.38

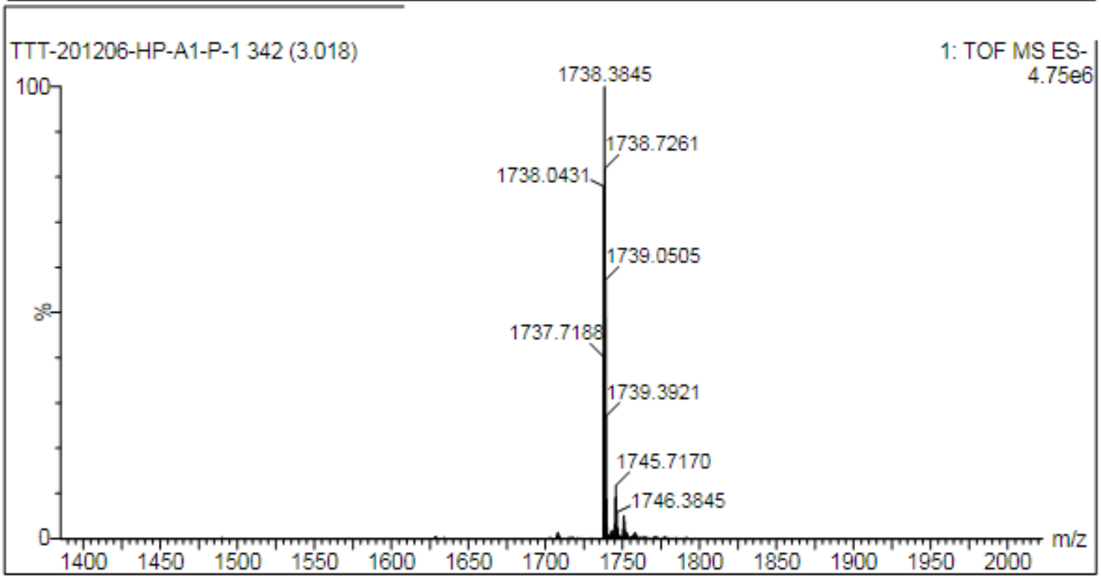

LC Trace and Mass of P1j

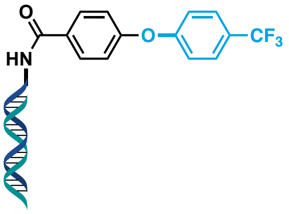

Following General Procedure

Yield: 78%

Exact mass: 5201.45

Triply charged mass [M]/3 - 1.00794, calculated 1732.80873; observed 1732.8571.

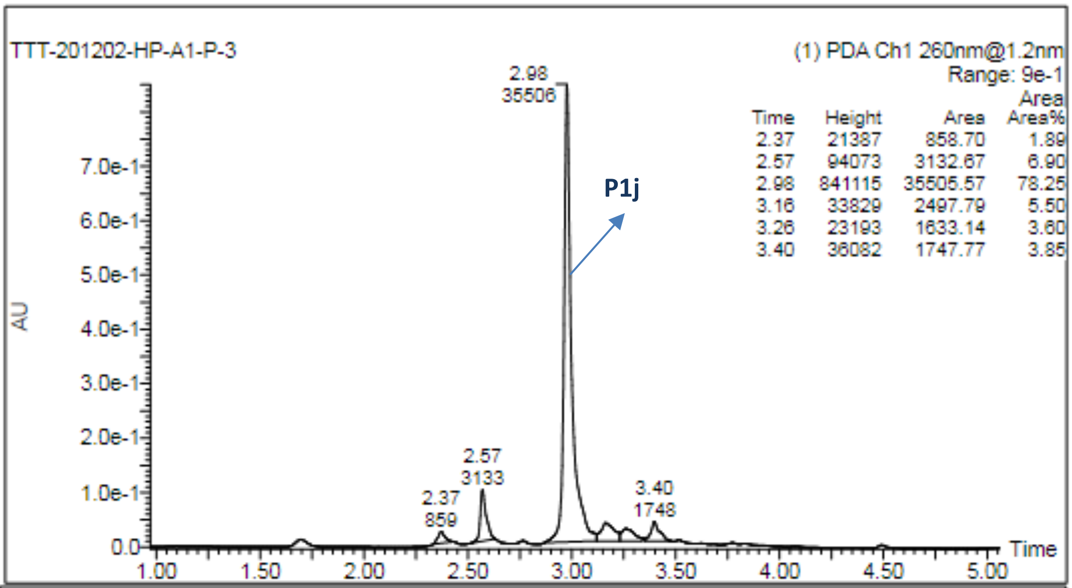

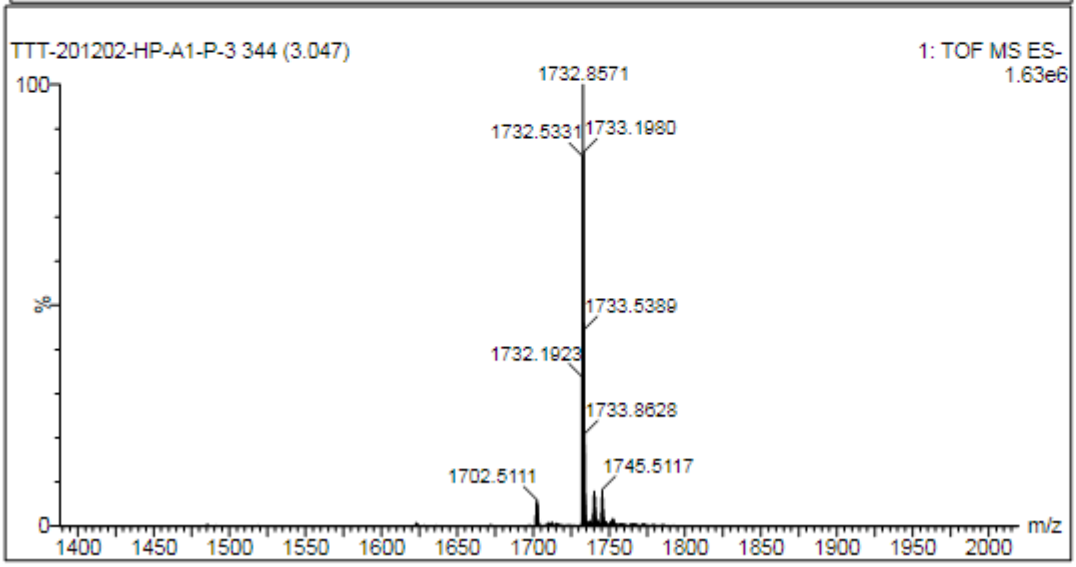

LC Trace and Mass of P1k

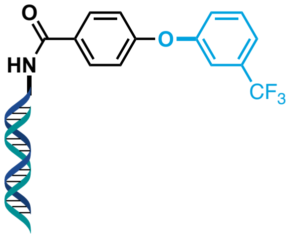

Following General Procedure

Yield: 72%

Exact mass: 5201.45

Triply charged mass [M]/3 - 1.00794, calculated 1732.80873; observed 1733.0615.

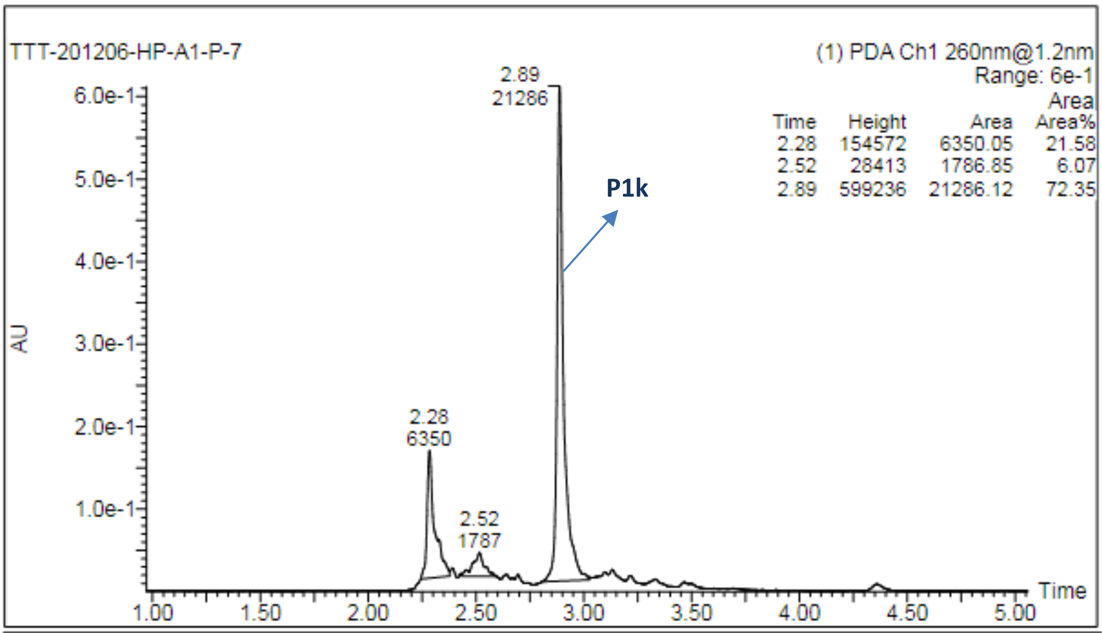

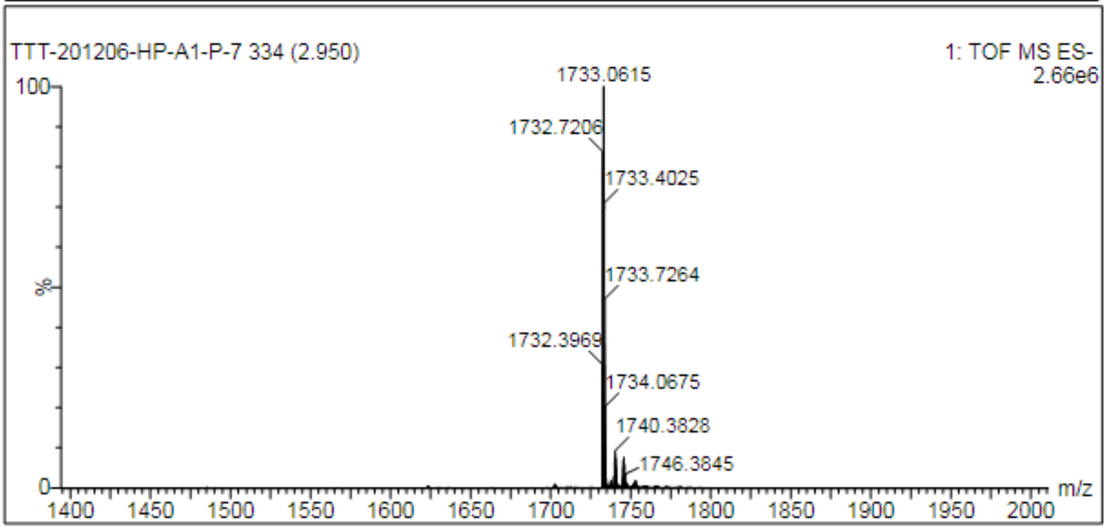

LC Trace and Mass of P1l

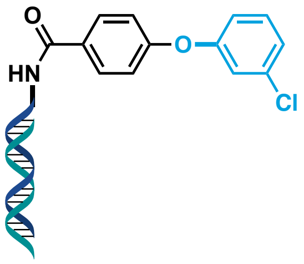

Following General Procedure

Yield: 28%

Exact mass: 5167.90

Triply charged mass [M]/3 - 1.00794, calculated 1721.62539; observed 1722.0491.

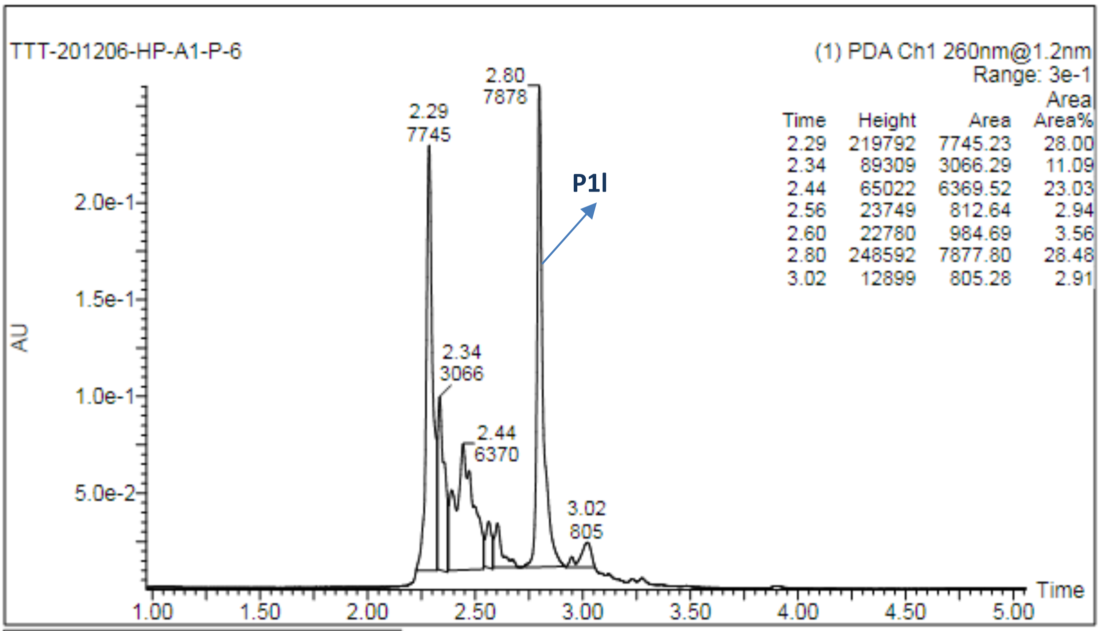

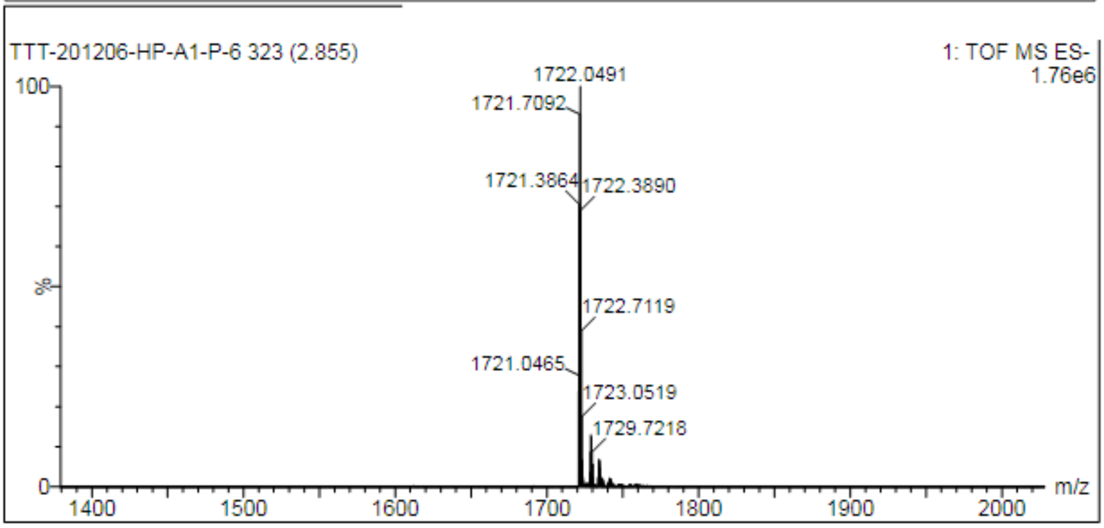

LC Trace and Mass of P1m

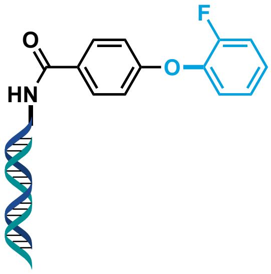

Following General Procedure

Yield: 61%

Exact mass: 5151.45

Triply charged mass [M]/3 - 1.00794, calculated 1716.14206; observed 1716.2083.

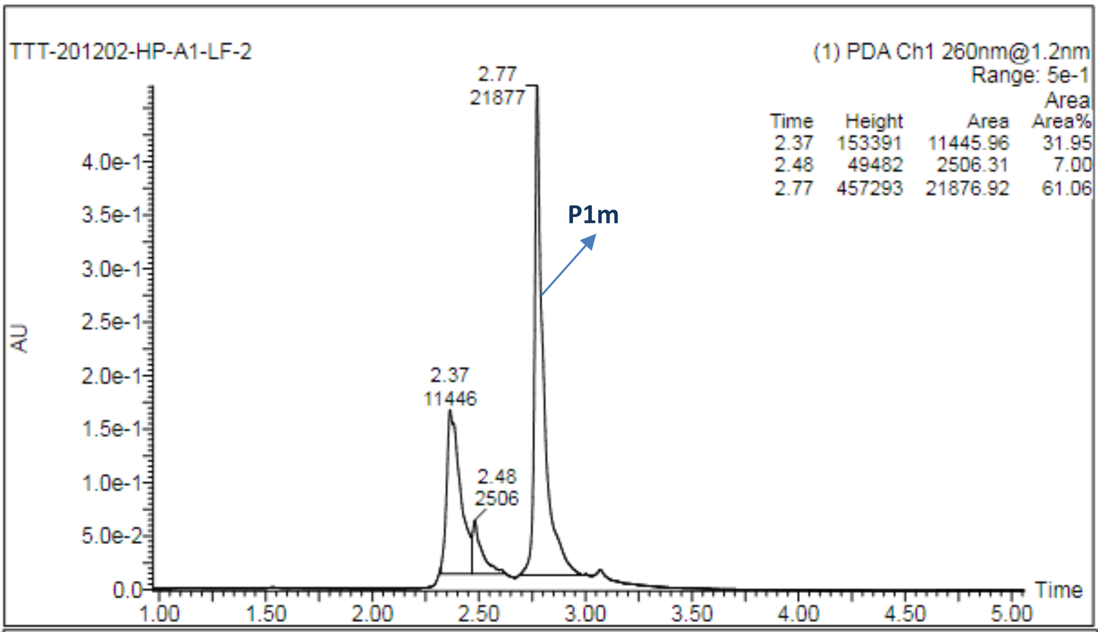

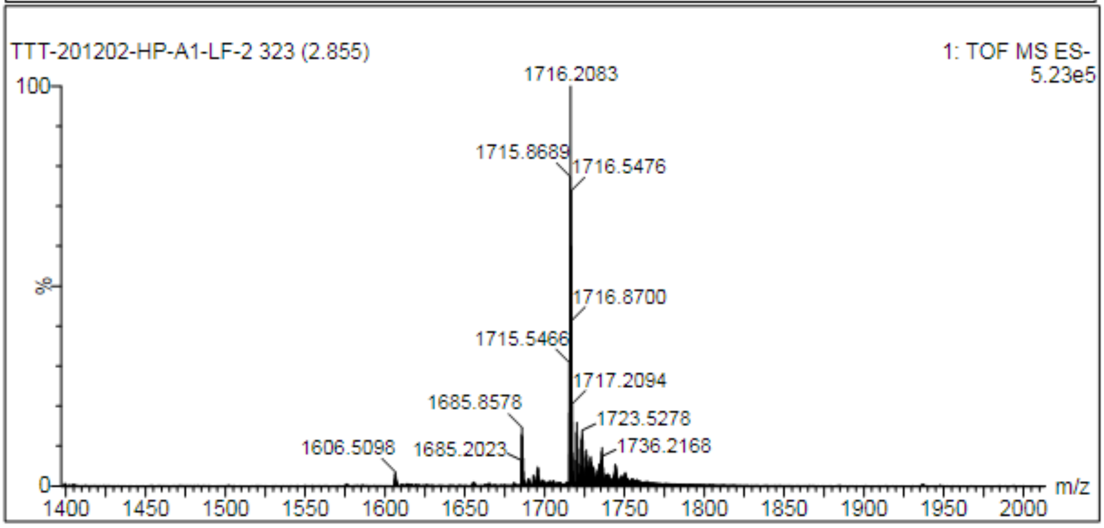

LC Trace and Mass of P1n

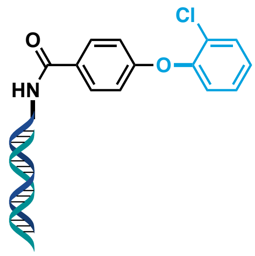

Following General Procedure

Yield: 85%

Exact mass: 5167.90

Triply charged mass [M]/3 - 1.00794, calculated 1721.62539; observed 1721.5223.

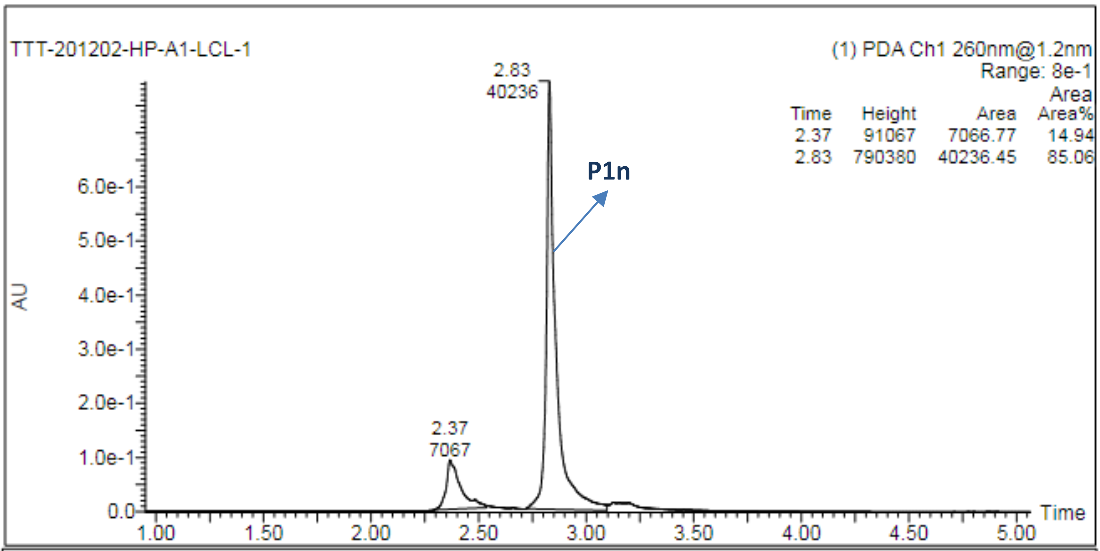

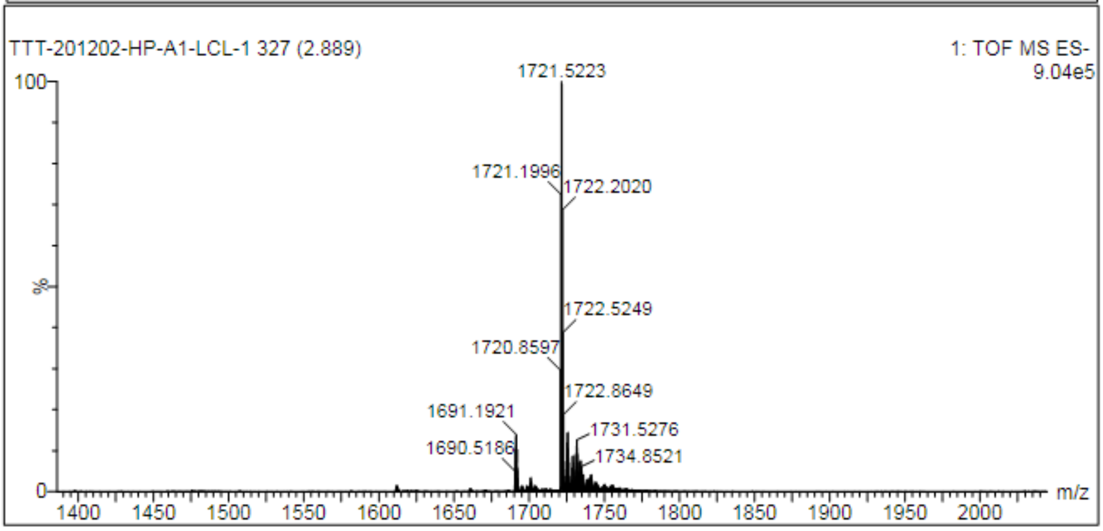

LC Trace and Mass of P1o

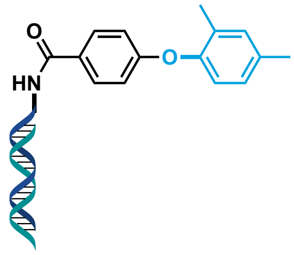

Following General Procedure

Yield: 99%

Exact mass: 5161.50

Triply charged mass [M]/3 - 1.00794, calculated 1719.49206; observed 1719.5350.

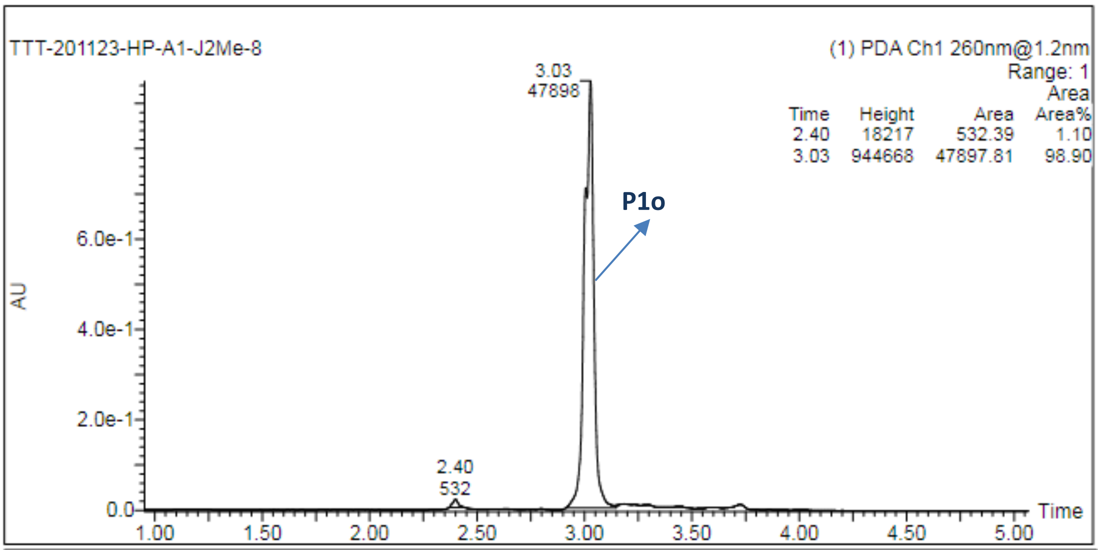

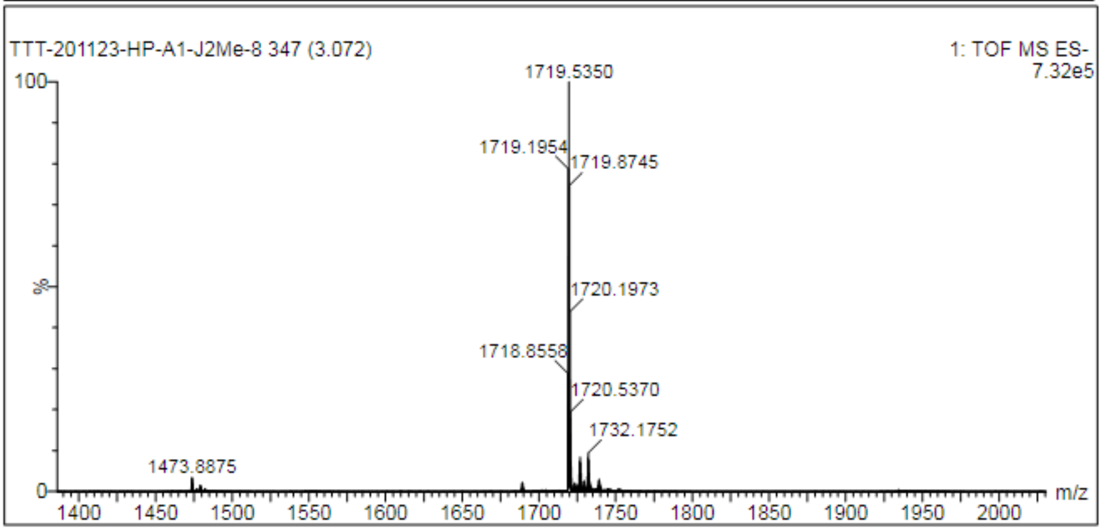

LC Trace and Mass of P1p

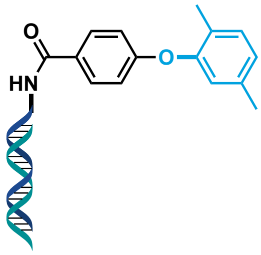

Following General Procedure

Yield: 96%

Exact mass: 5161.50

Triply charged mass [M]/3 - 1.00794, calculated 1719.49206; observed 1719.5181.

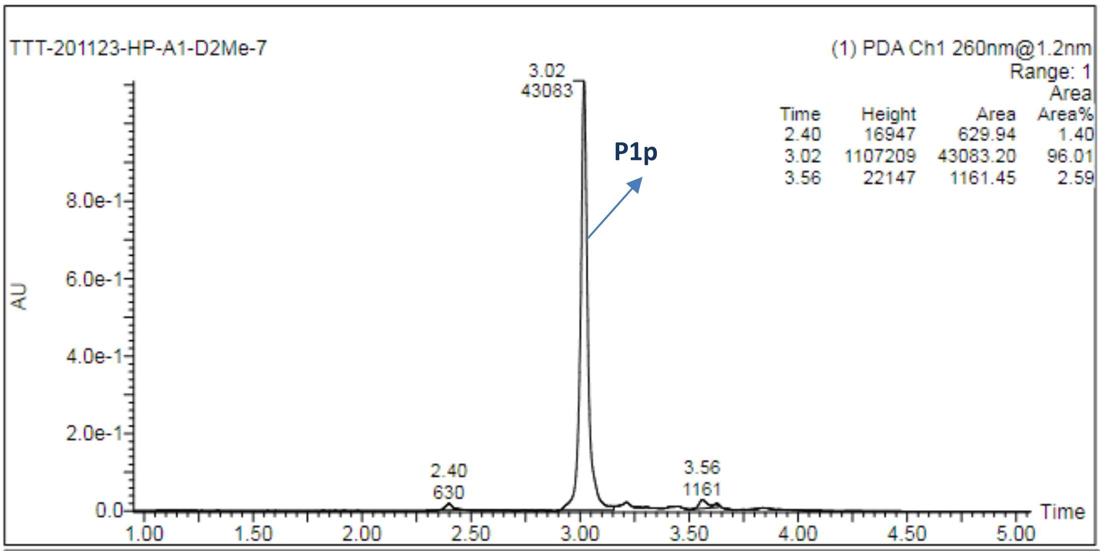

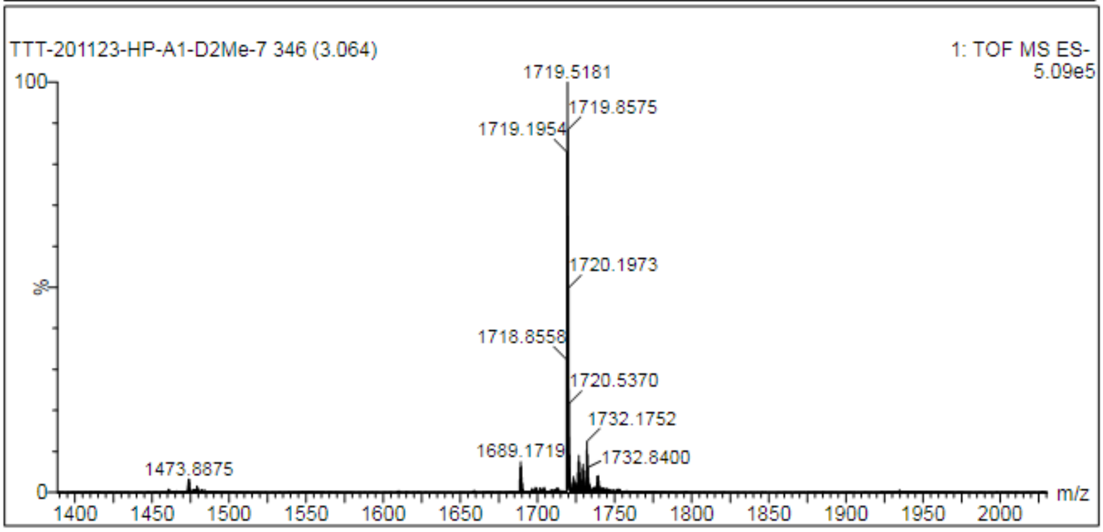

LC Trace and Mass of P1q

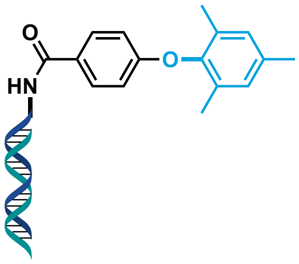

Following General Procedure

Yield: 92%

Exact mass: 5175.50

Triply charged mass [M]/3 - 1.00794, calculated 1724.15873; observed 1724.1910.

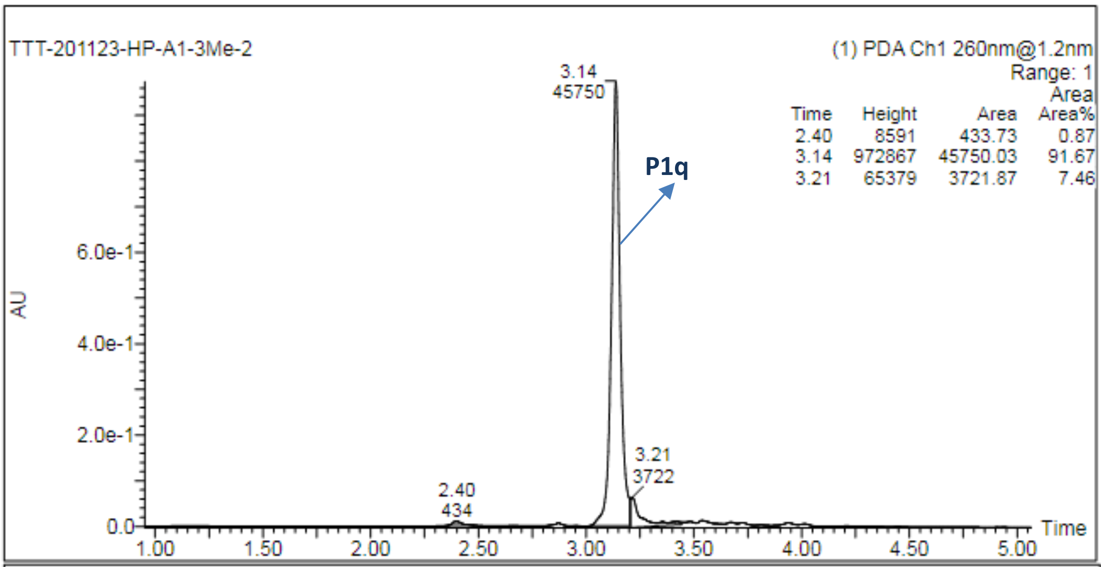

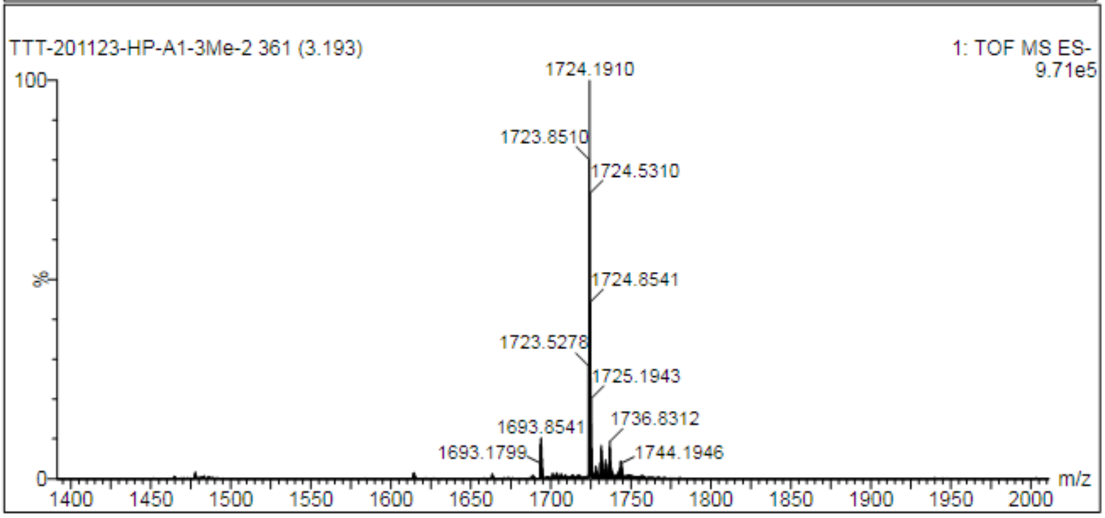

LC Trace and Mass of P1r

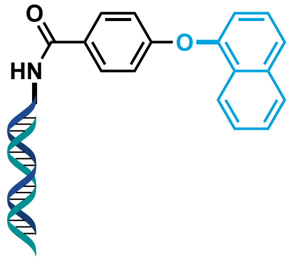

Following General Procedure
Yield: 86%
Exact mass: 5183.50
Triply charged mass [M]/3 - 1.00794, calculated 1726.82539; observed 1727.3893.

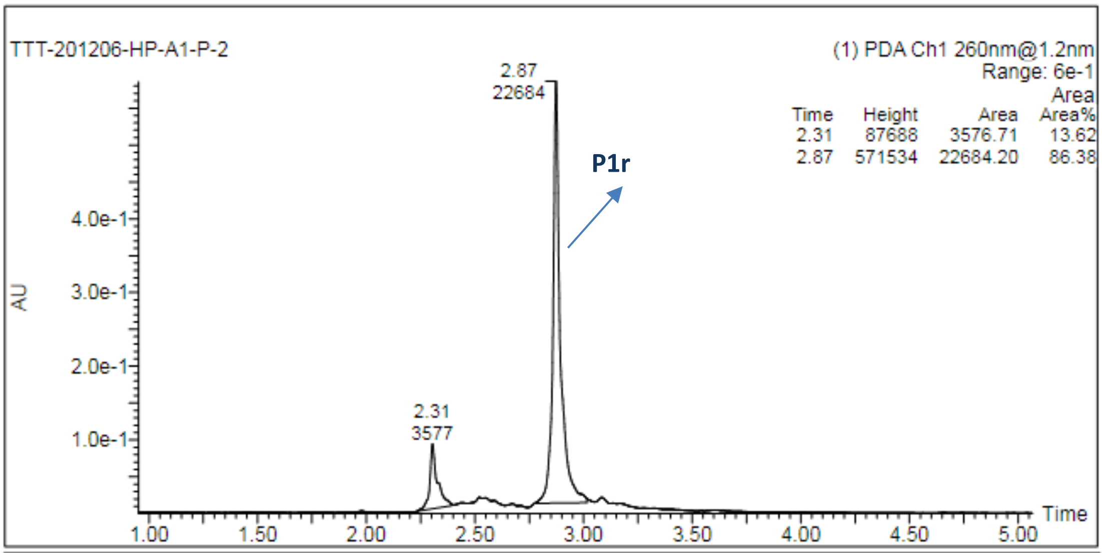

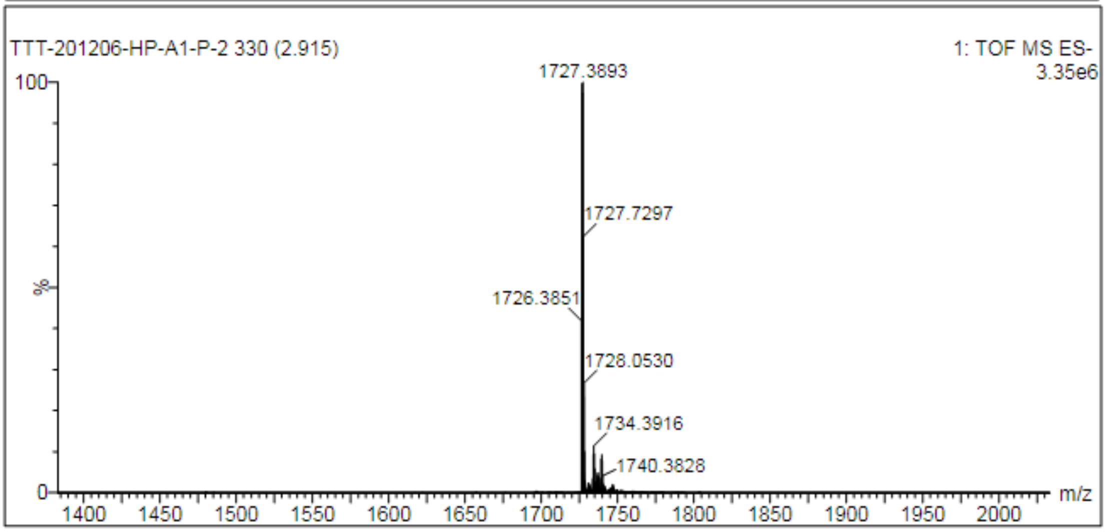

LC Trace and Mass of P2a

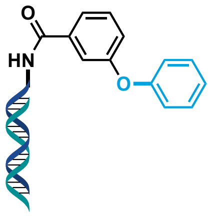

Following General Procedure v

Yield: 92%

Exact mass: 5133.45

Triply charged mass [M]/3 - 1.00794, calculated 1710.14206; observed 1710.3265.

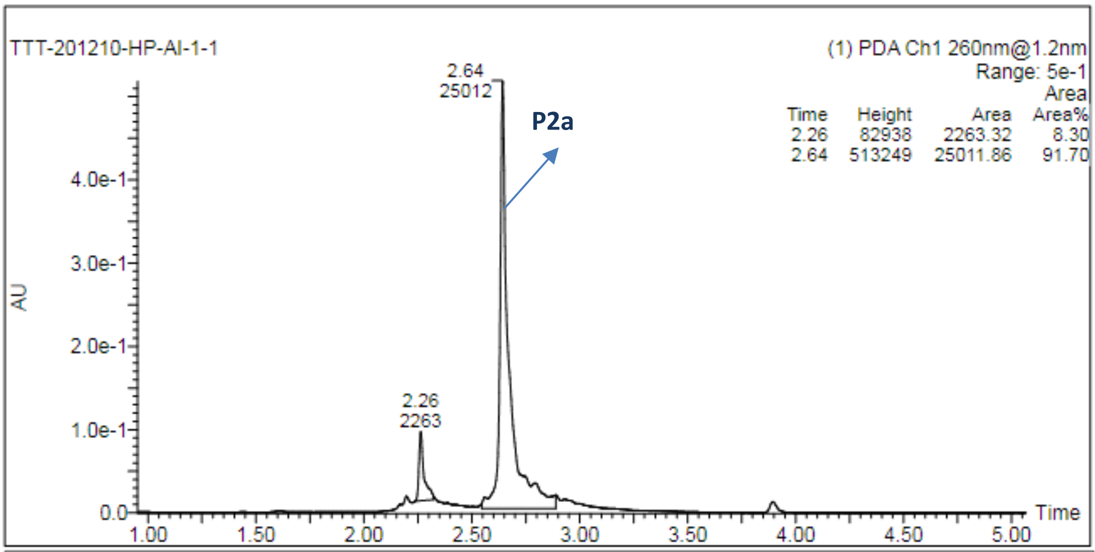

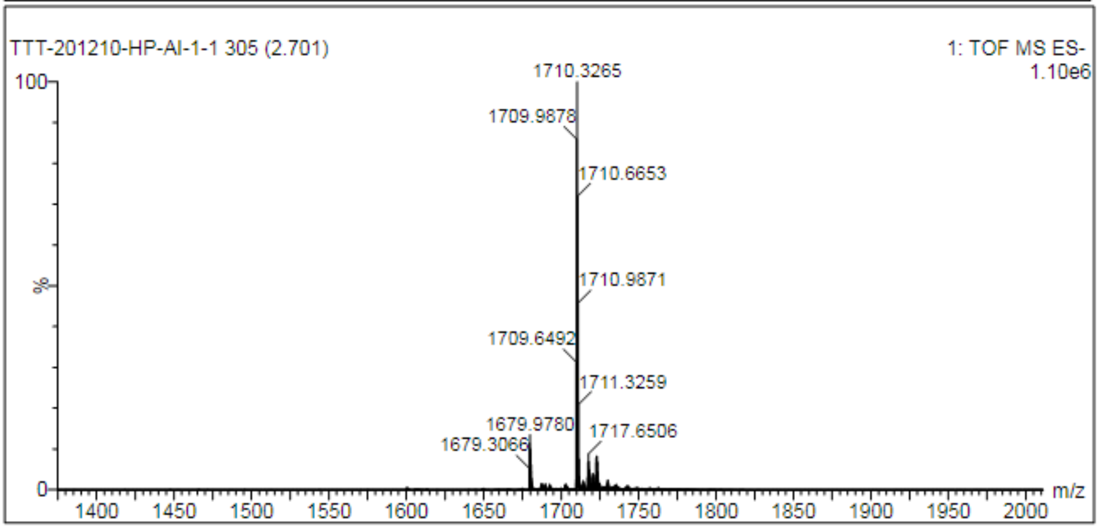

LC Trace and Mass of P2b

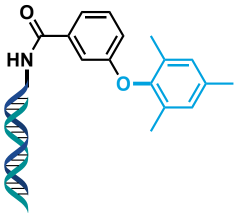

Following General Procedure v

Yield: 93%

Exact mass: 5175.5

Triply charged mass [M]/3 - 1.00794, calculated 1724.15873; observed 1724.3610.

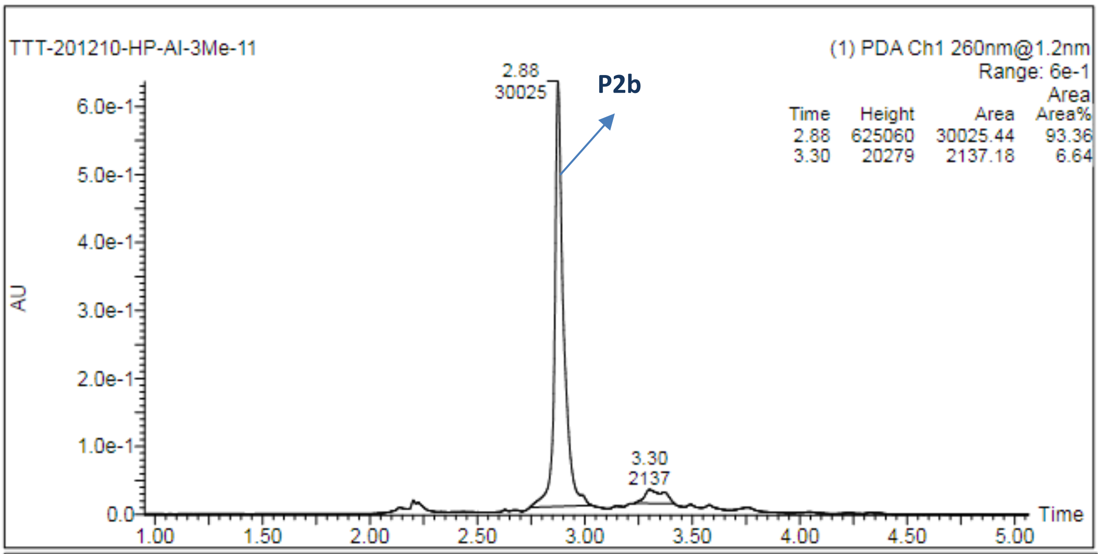

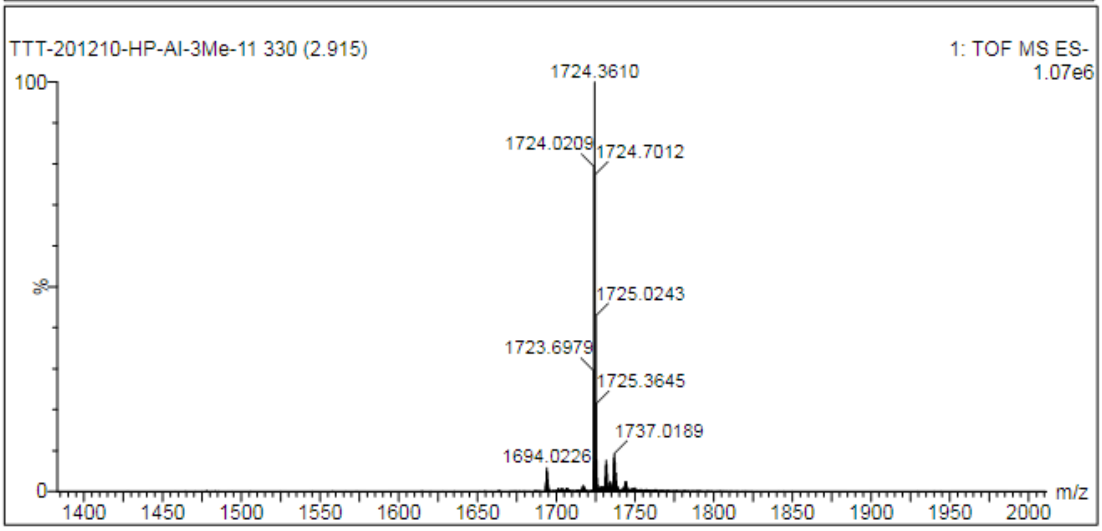

LC Trace and Mass of P2c

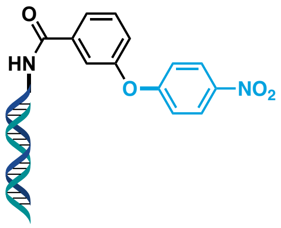

Following General Procedure v

Yield: 92%

Exact mass: 5178.45

Triply charged mass [M]/3 - 1.00794, calculated 1725.14206; observed 1725.0059.

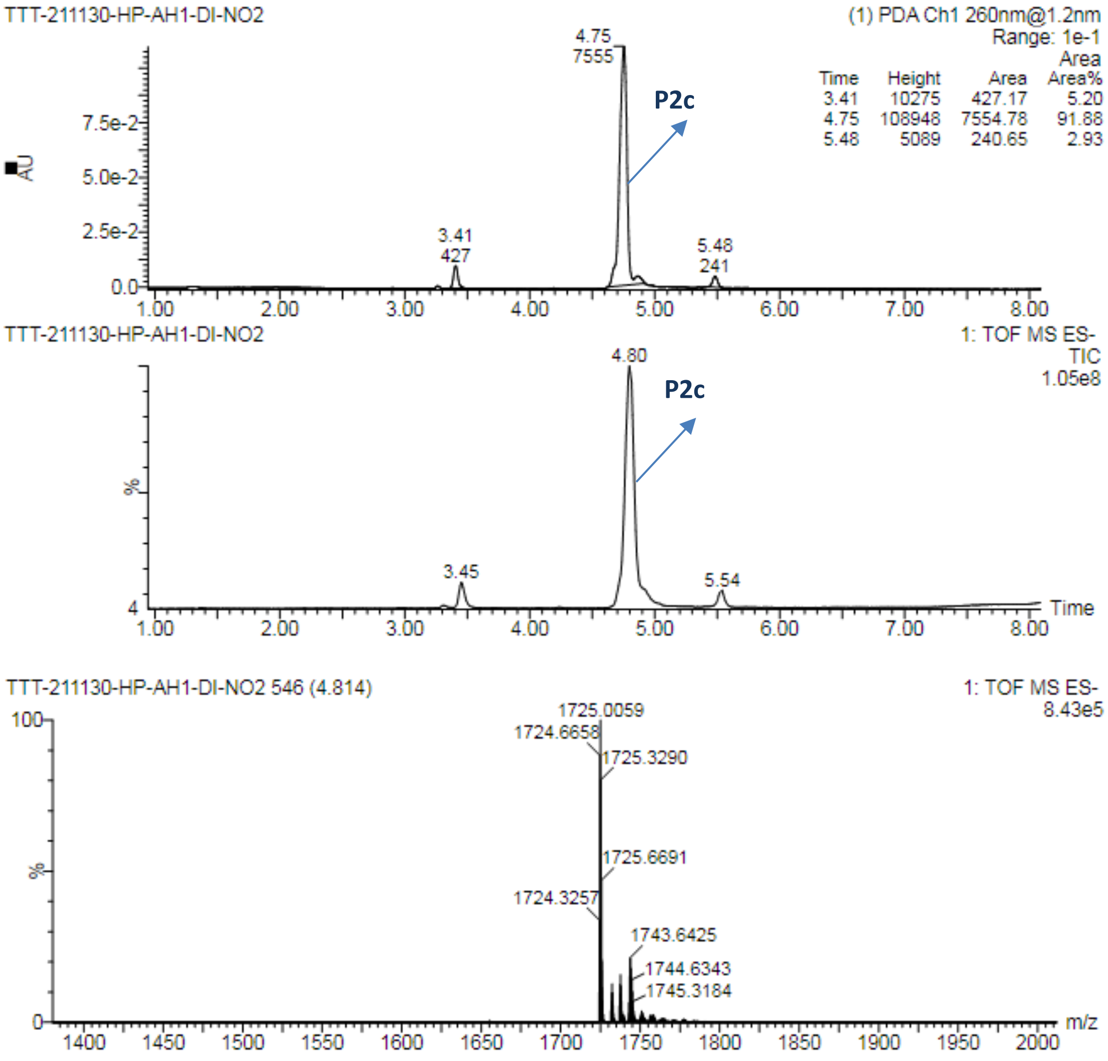

LC Trace and Mass of P3a

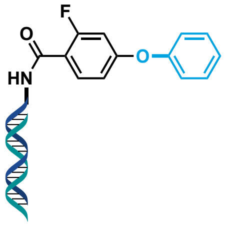

Following General Procedure v

Yield: 36%

Exact mass: 5151.45

Triply charged mass [M]/3 - 1.00794, calculated 1716.14206; observed 1716.3269.

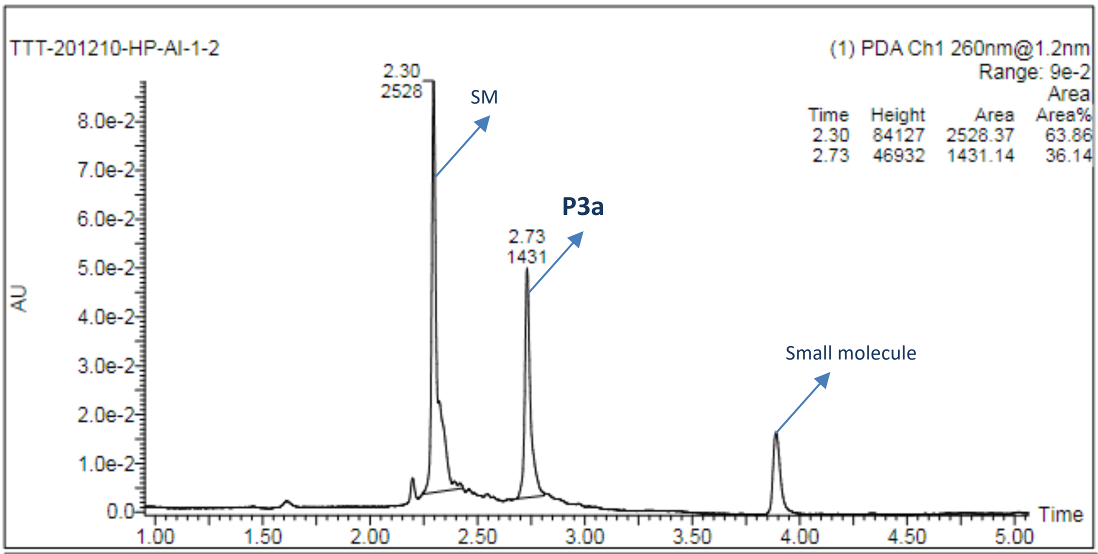

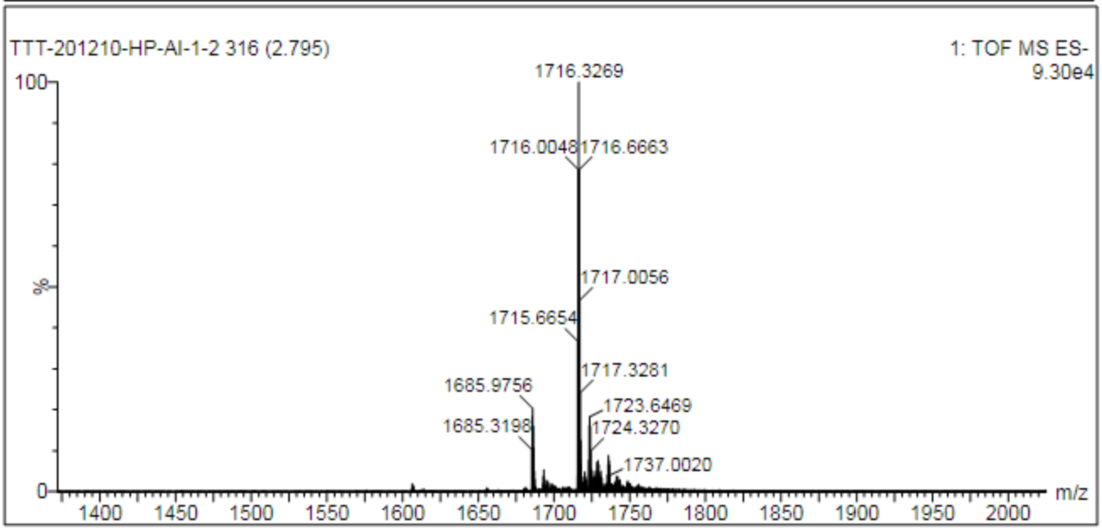

LC Trace and Mass of P3b

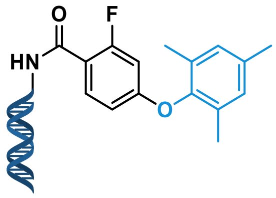

Following General Procedure v

Yield: 95%

Exact mass: 5193.50

Triply charged mass [M]/3 - 1.00794, calculated 1730.15873; observed 1730.3690.

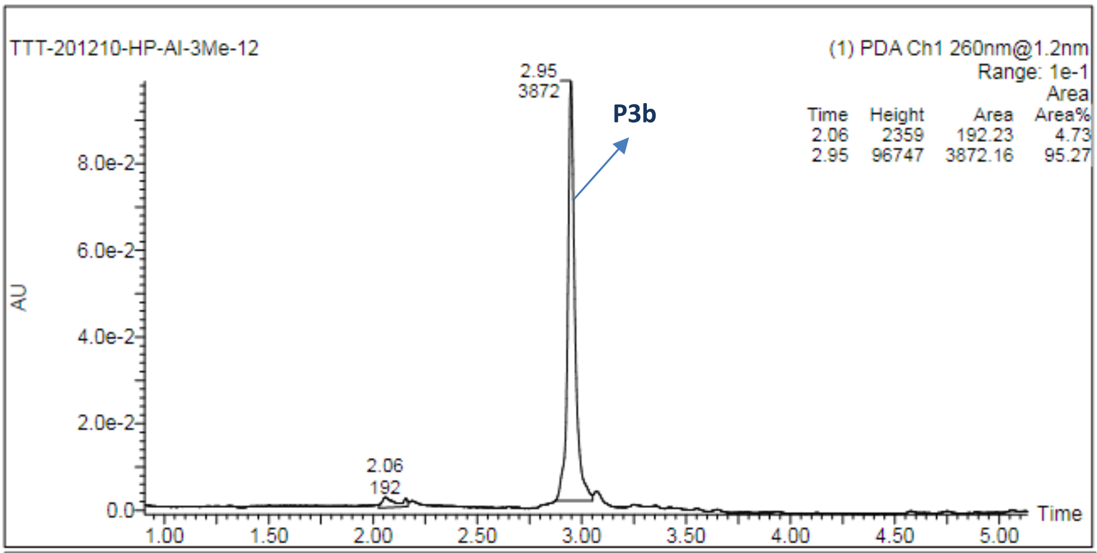

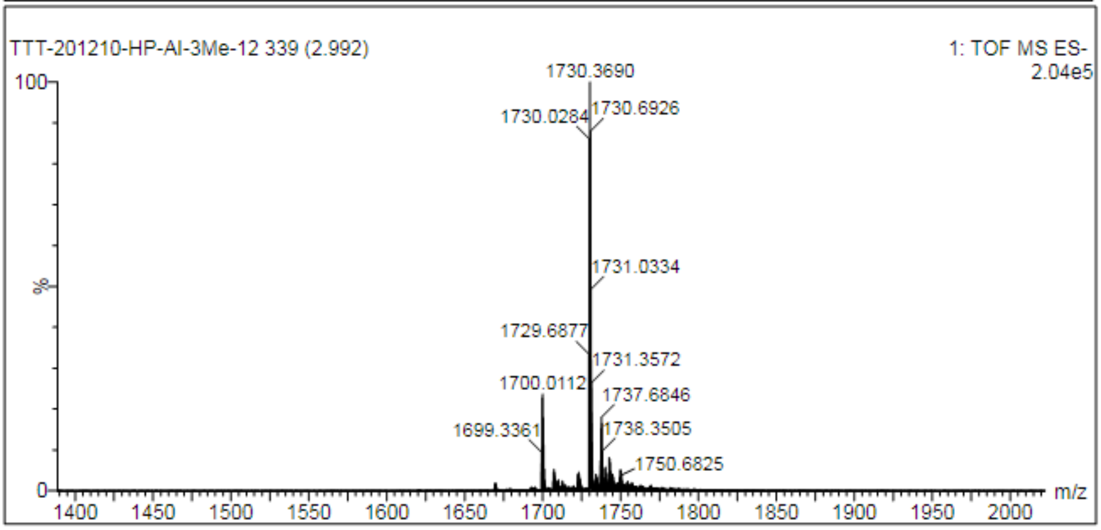

LC Trace and Mass of P3c

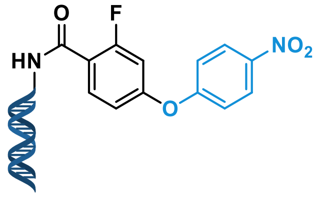

Following General Procedure v

Yield: 67%

Exact mass: 5196.45

Triply charged mass [M]/3 - 1.00794, calculated 1731.14206; observed 1731.3743.

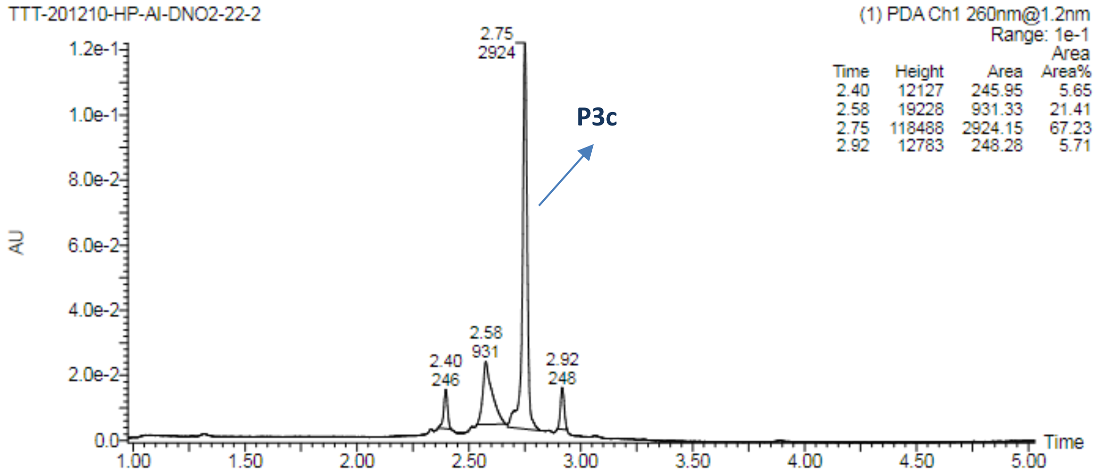

Time	Height	Area	Area%
2.40	12127	245.95	5.65
2.58	19228	931.33	21.41
2.75	118488	2924.15	67.23
2.92	12783	248.28	5.71

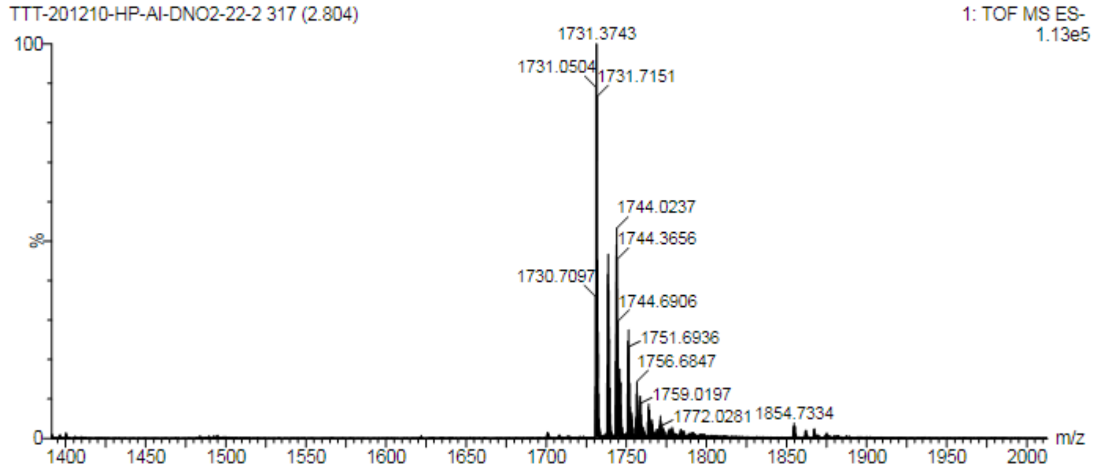

LC Trace and Mass of P4a

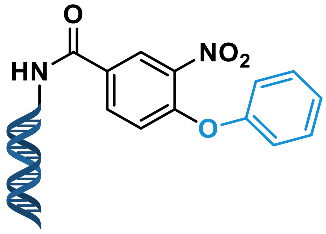

Following General Procedure v

Yield: 8%

Exact mass: 5178.45

Triply charged mass [M]/3 - 1.00794, calculated 1725.14206; observed 1725.3304.

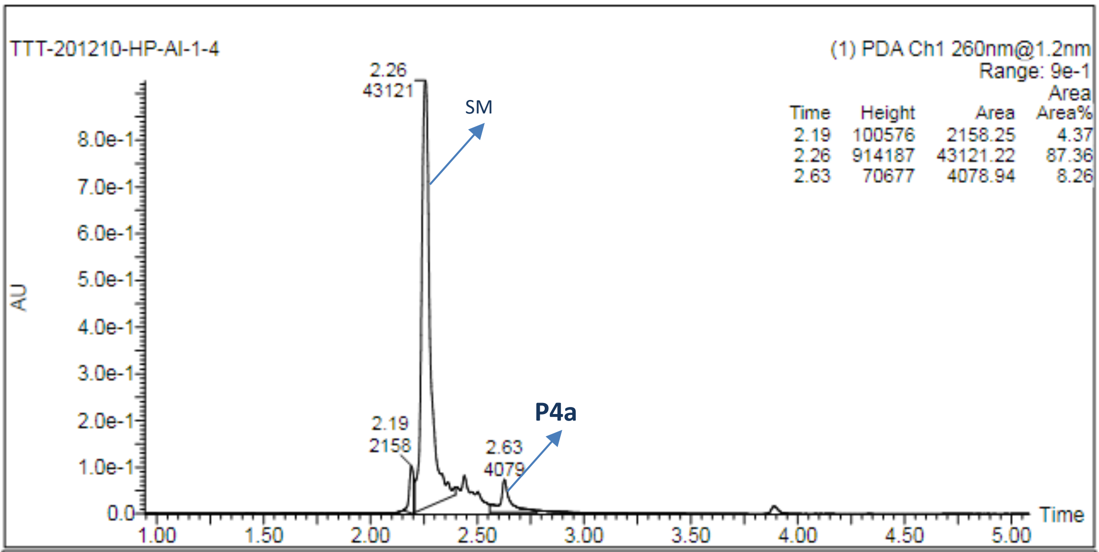

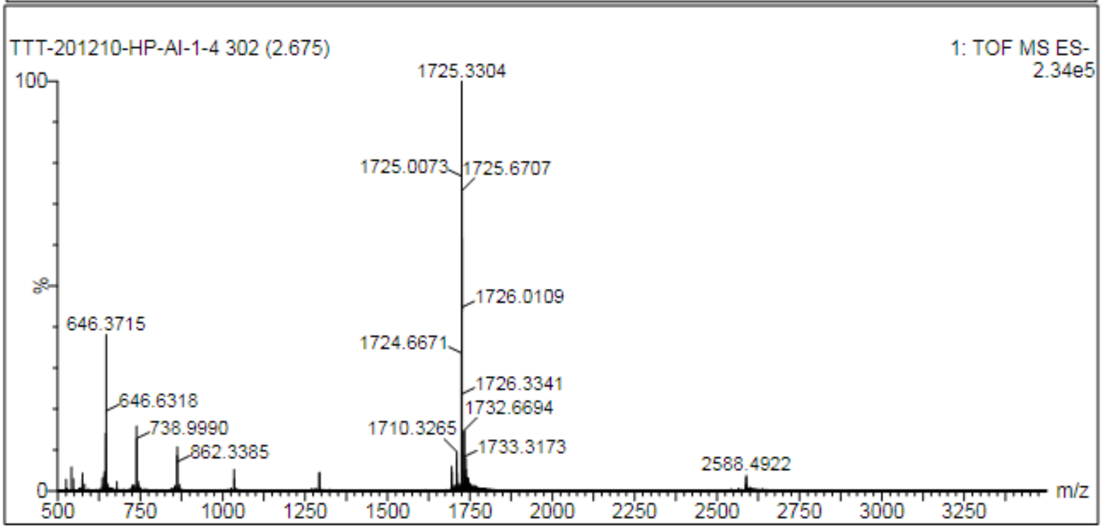

LC Trace and Mass of P4b

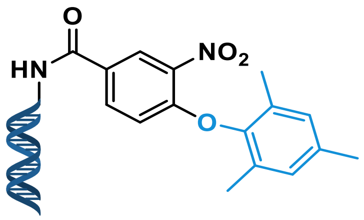

Following General Procedure v

Yield: 80%

Exact mass: 5220.50

Triply charged mass [M]/3 - 1.00794, calculated 1739.15873; observed 1739.3921.

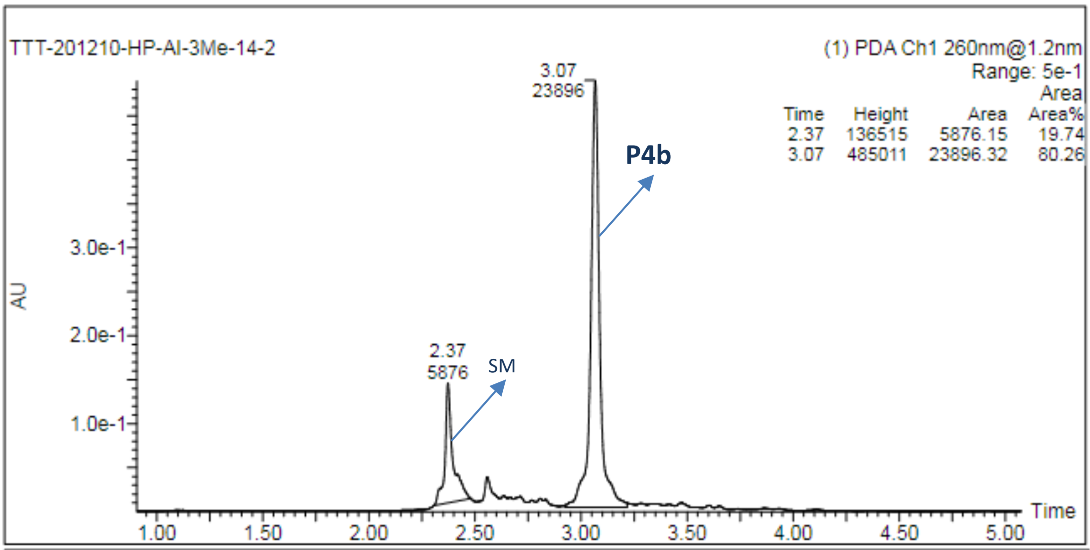

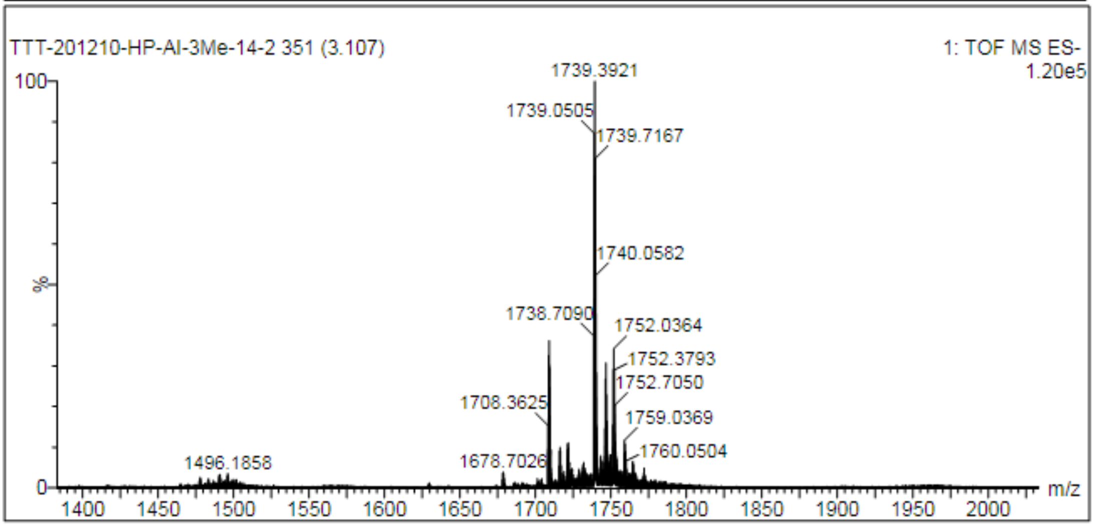

LC Trace and Mass of P4c

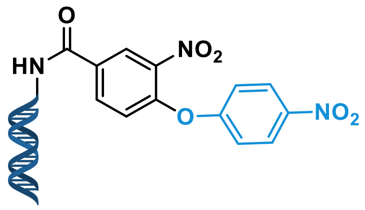

Following General Procedure v

Yield: 10%

Exact mass: 5223.45

Triply charged mass [M]/3 - 1.00794, calculated 1740.14206; observed 1740.3828.

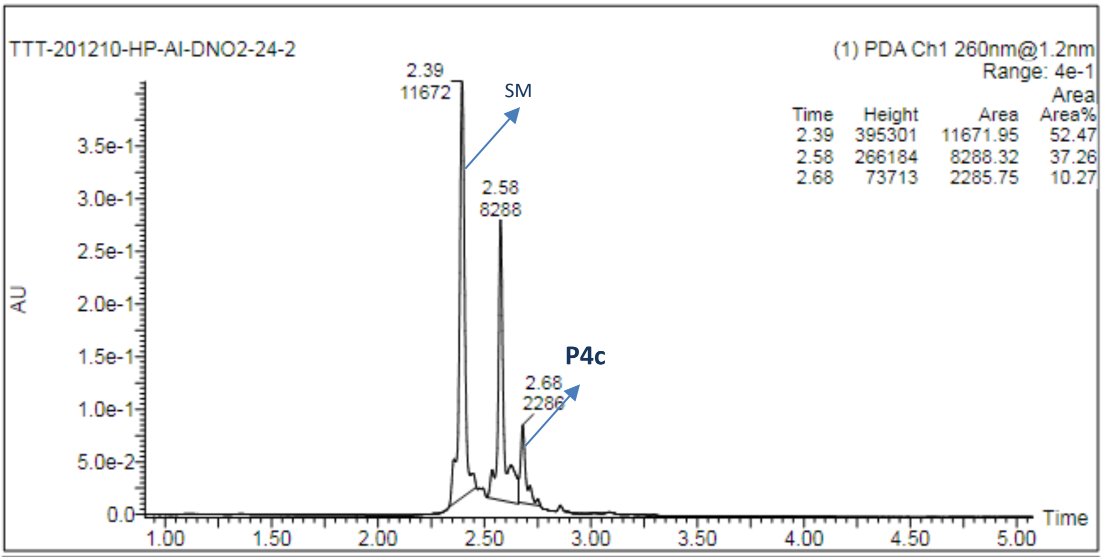

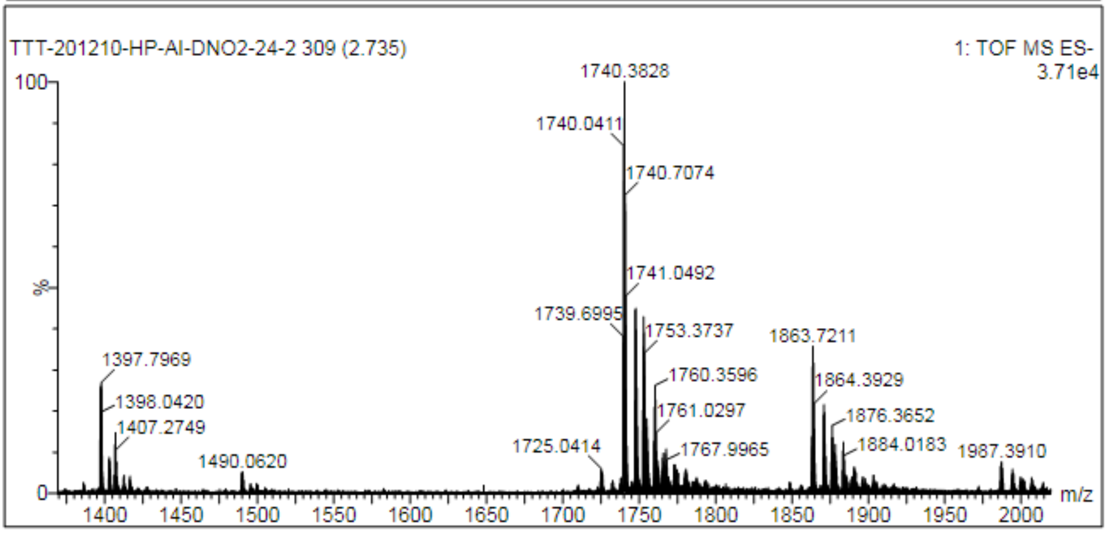

LC Trace and Mass of P5a

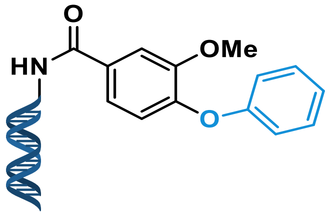

Following General Procedure v

Yield: 95%

Exact mass: 5163.45

Triply charged mass [M]/3 - 1.00794, calculated 1720.14206; observed 1720.3163.

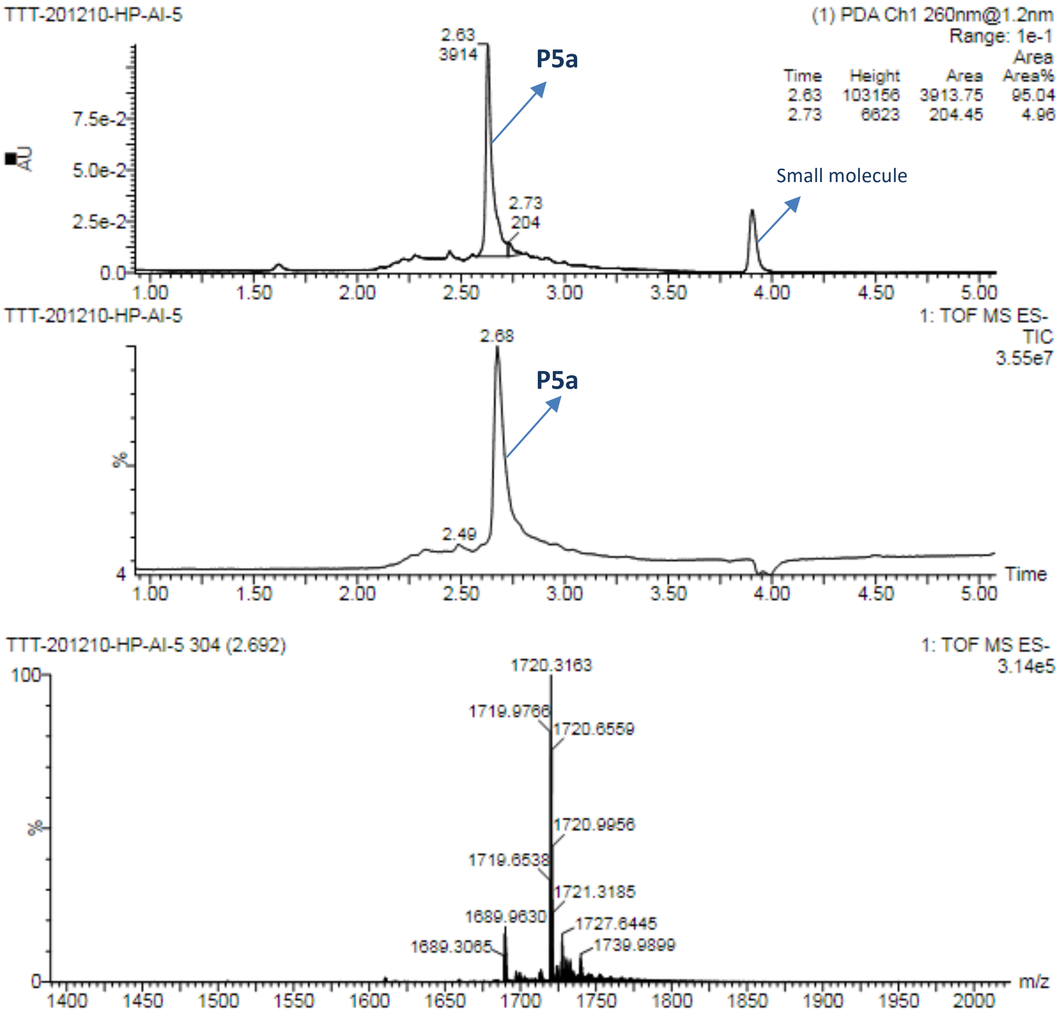

LC Trace and Mass of P5b

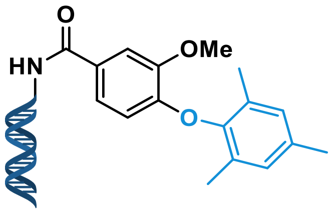

Following General Procedure v

Yield: 95%

Exact mass: 5205.50

Triply charged mass [M]/3 - 1.00794, calculated 1734.15873; observed 1734.3916.

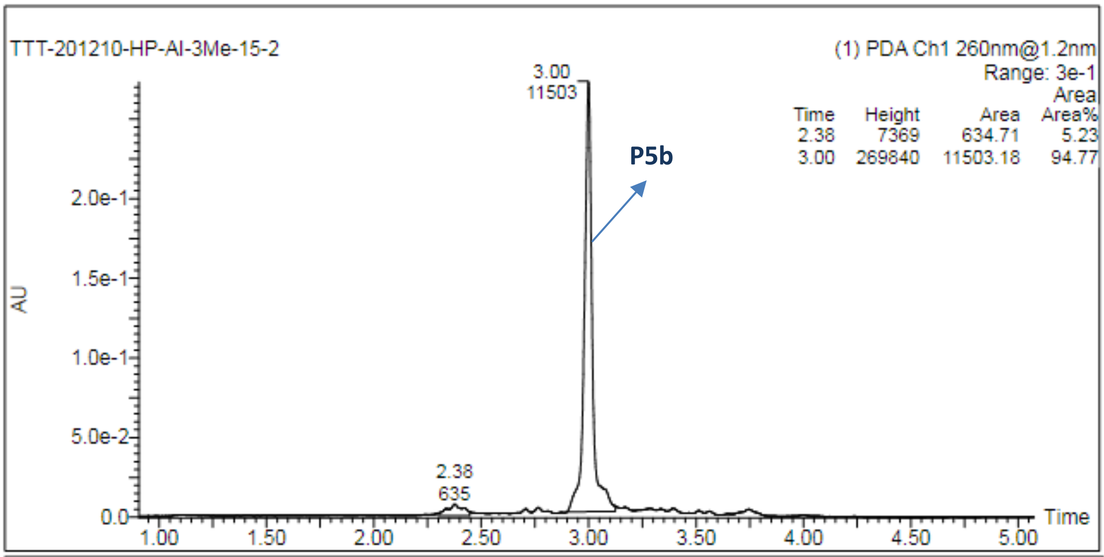

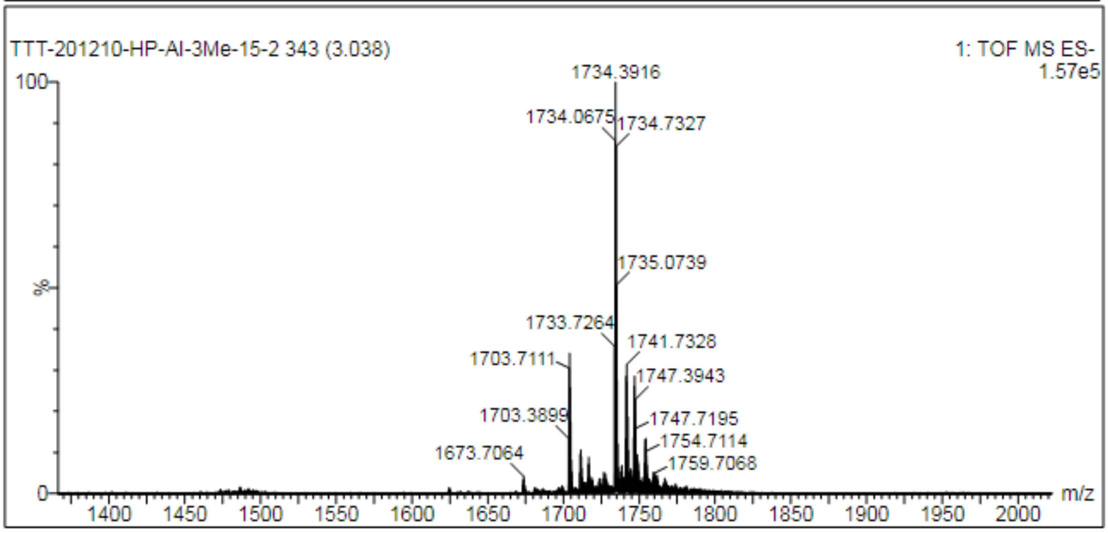

LC Trace and Mass of P5c

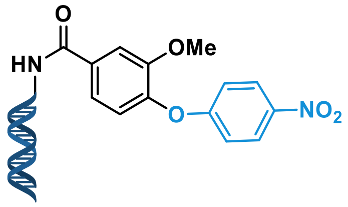

Following General Procedure v

Yield: 89%

Exact mass: 5208.45

Triply charged mass [M]/3 - 1.00794, calculated 1735.14206; observed 1735.3809.

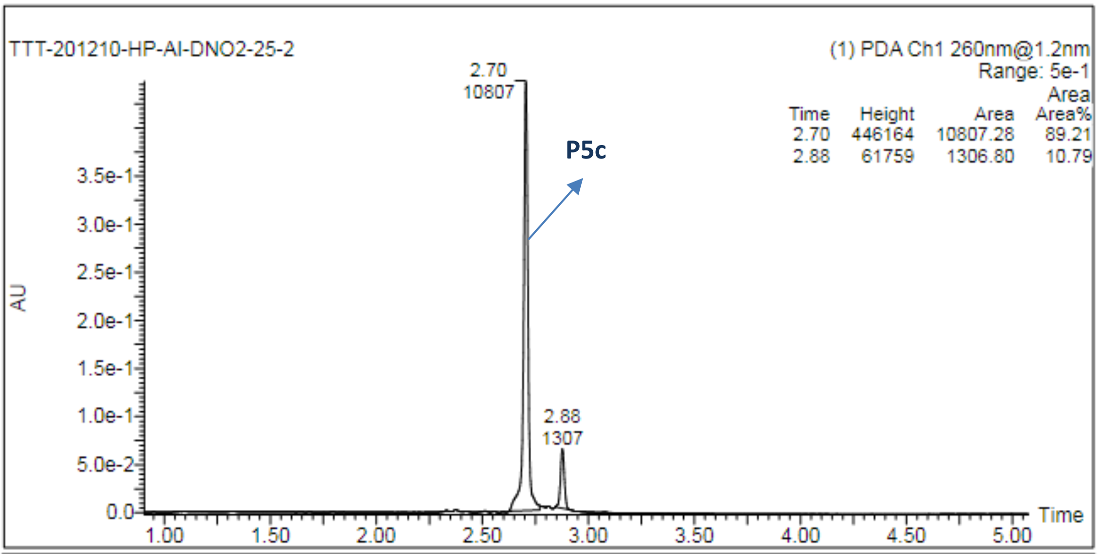

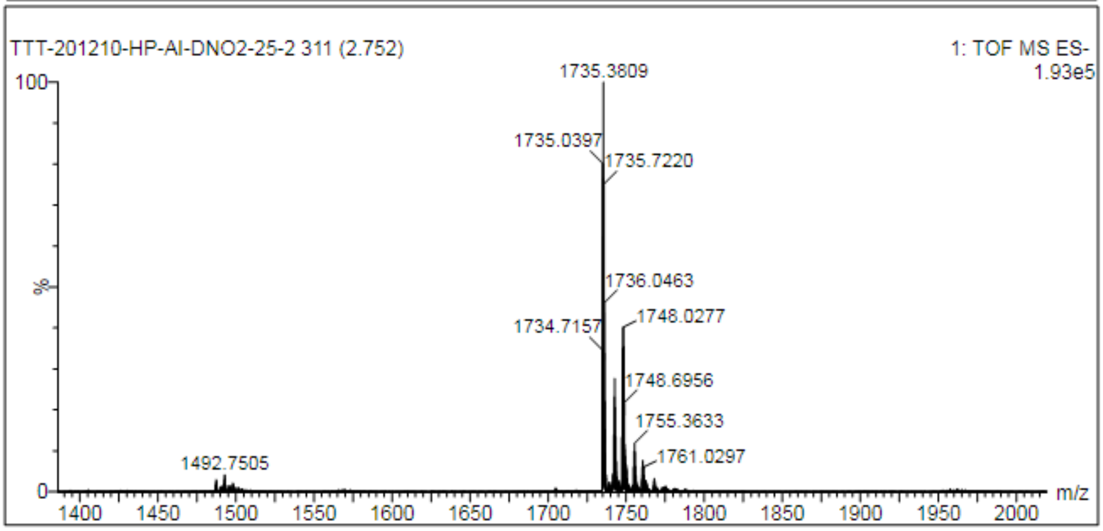

LC Trace and Mass of P6a

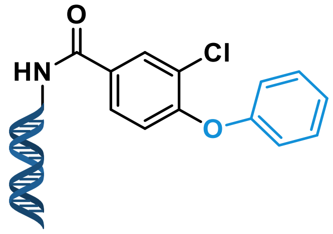

Following General Procedure v

Yield: 86%

Exact mass: 5167.95

Triply charged mass [M]/3 - 1.00794, calculated 1721.64206; observed 1721.6583.

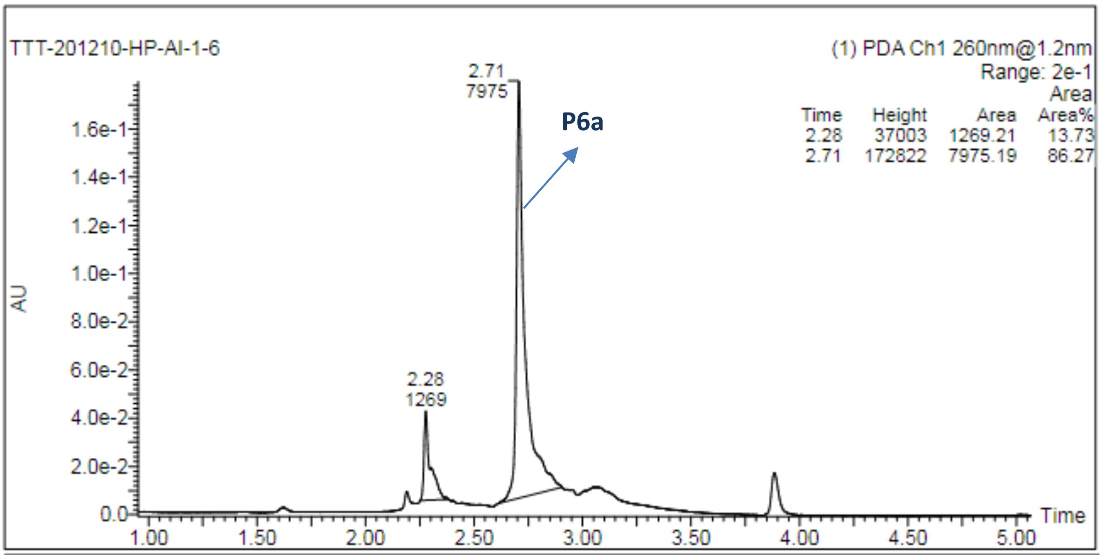

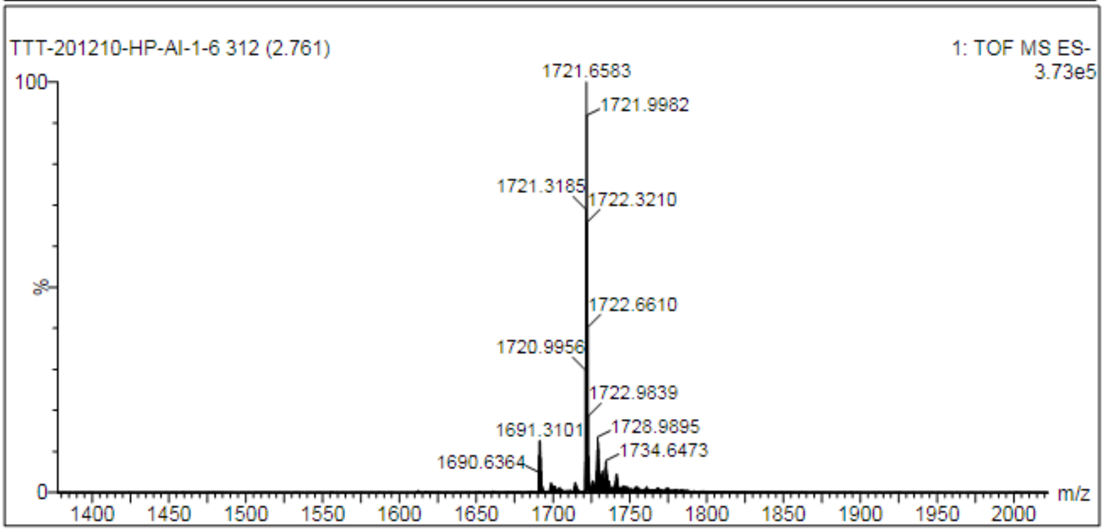

LC Trace and Mass of P6b

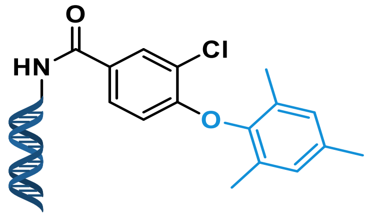

Following General Procedure v

Yield: 95%

Exact mass: 5210.00

Triply charged mass [M]/3 - 1.00794, calculated 1735.65873; observed 1735.7220.

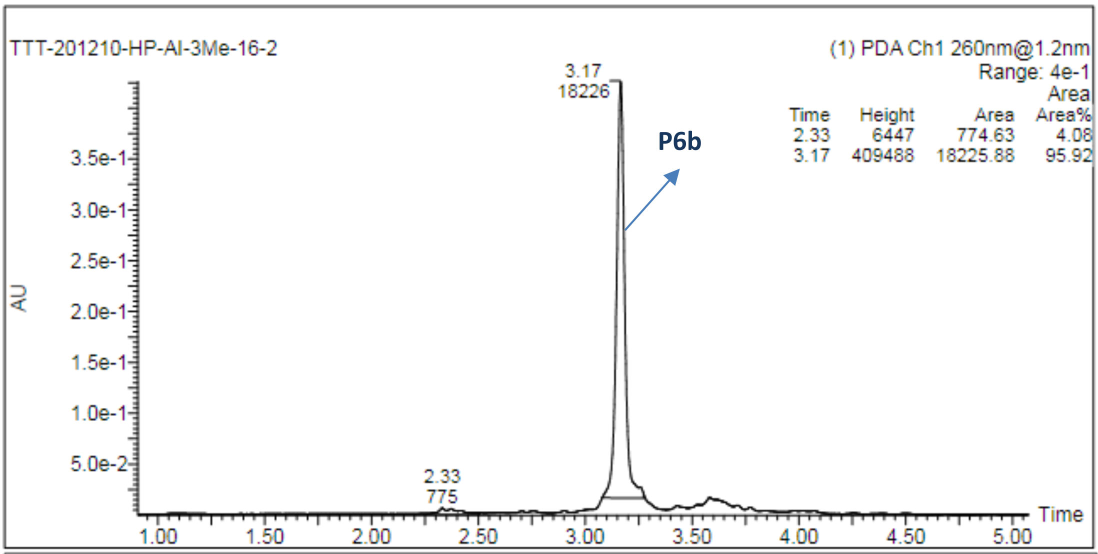

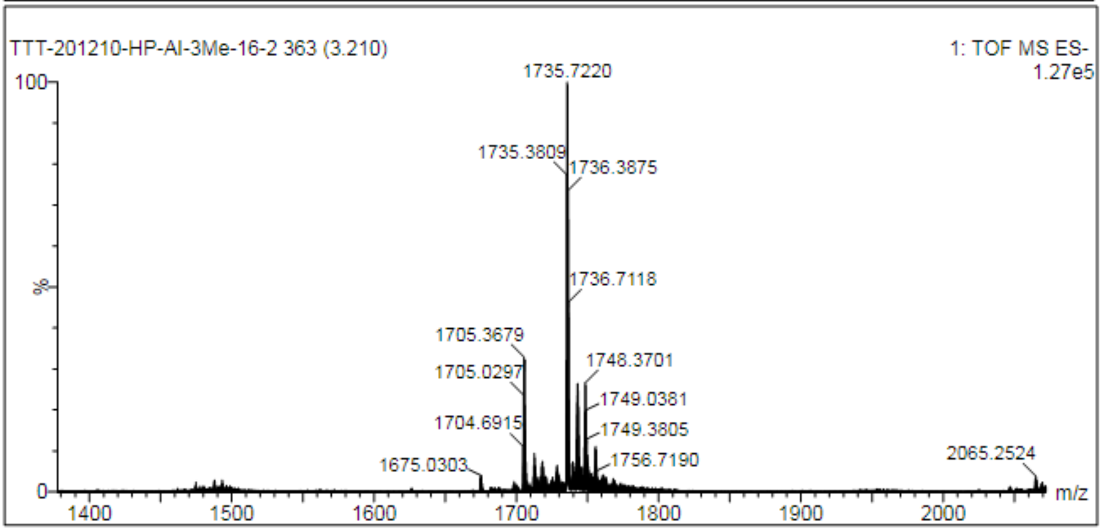

LC Trace and Mass of P6c

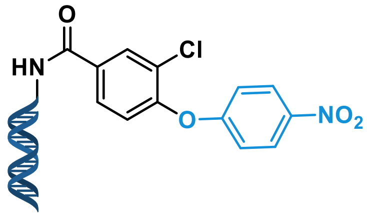

Following General Procedure v

Yield: 85%

Exact mass: 5212.95

Triply charged mass [M]/3 - 1.00794, calculated 1736.64206; observed 1736.6947.

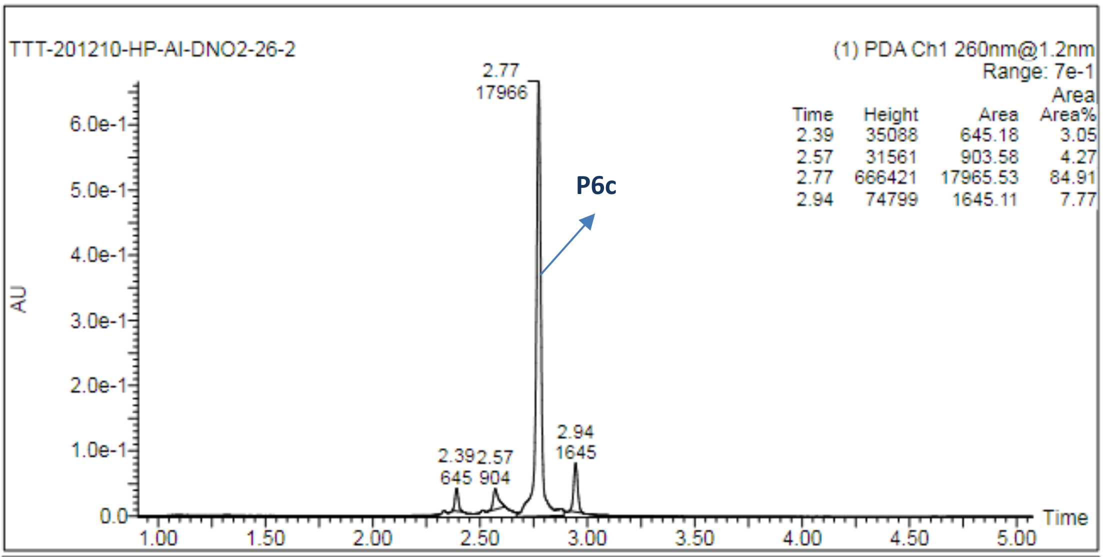

Time	Height	Area	Area%
2.39	35088	645.18	3.05
2.57	31561	903.58	4.27
2.77	666421	17965.53	84.91
2.94	74799	1645.11	7.77

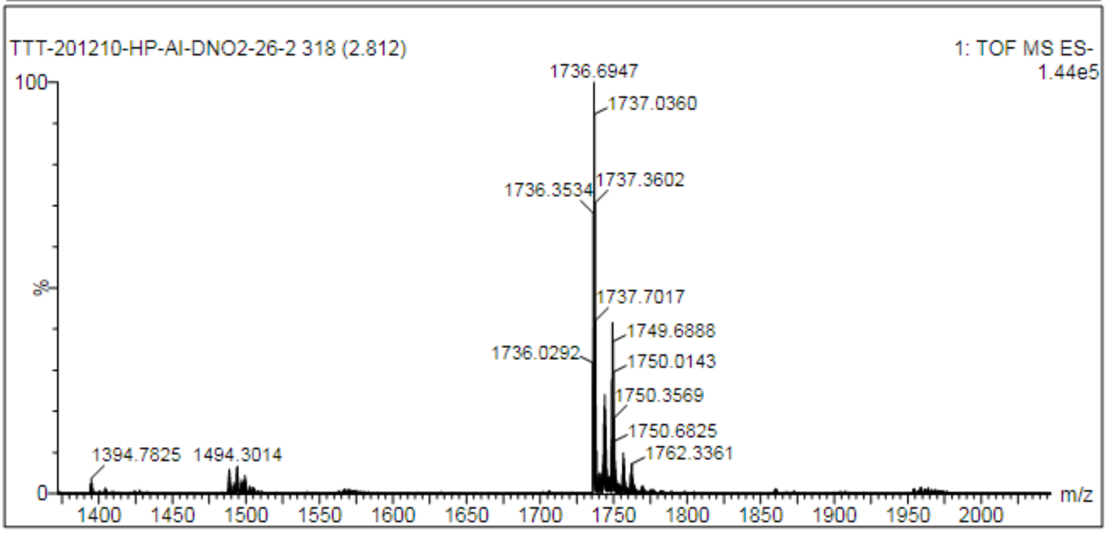

LC Trace and Mass of P7a

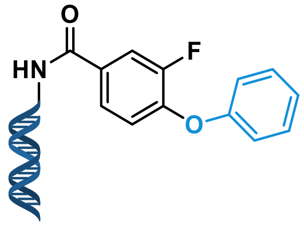

Following General Procedure v
Yield: 95%
Exact mass: 5151.45
Triply charged mass [M]/3 - 1.00794, calculated 1716.14206; observed 1716.3269.

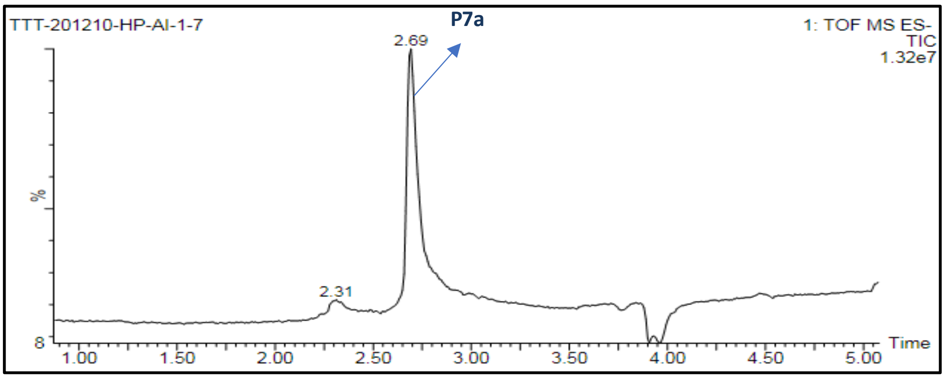

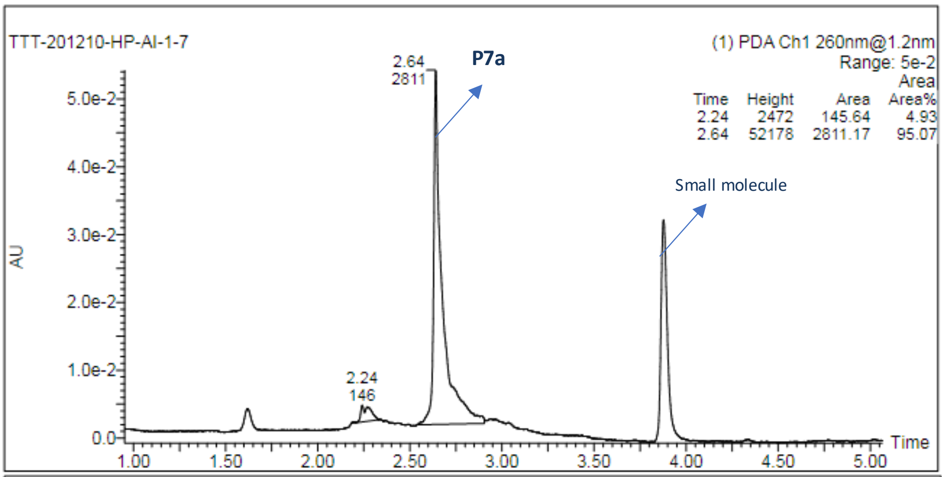

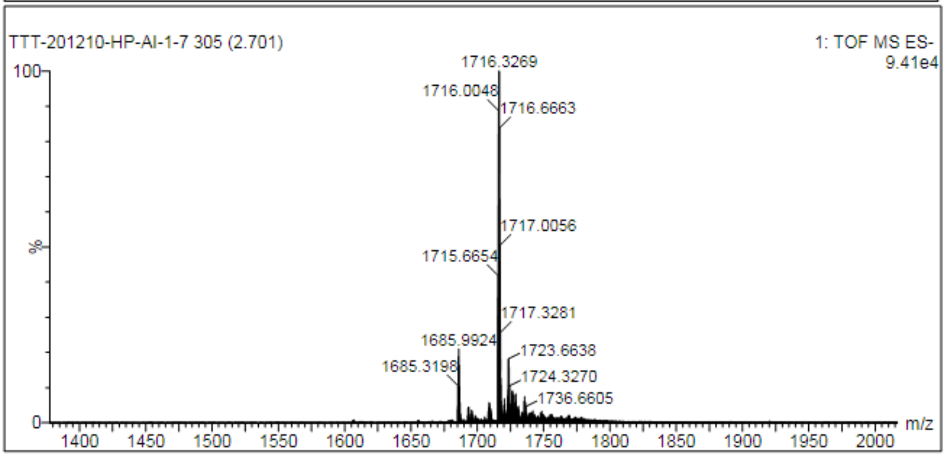

LC Trace and Mass of P7b

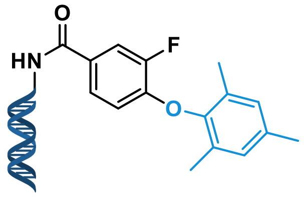

Following General Procedure v

Yield: 95%

Exact mass: 5193.50

Triply charged mass [M]/3 - 1.00794, calculated 1730.15873; observed 1730.3860.

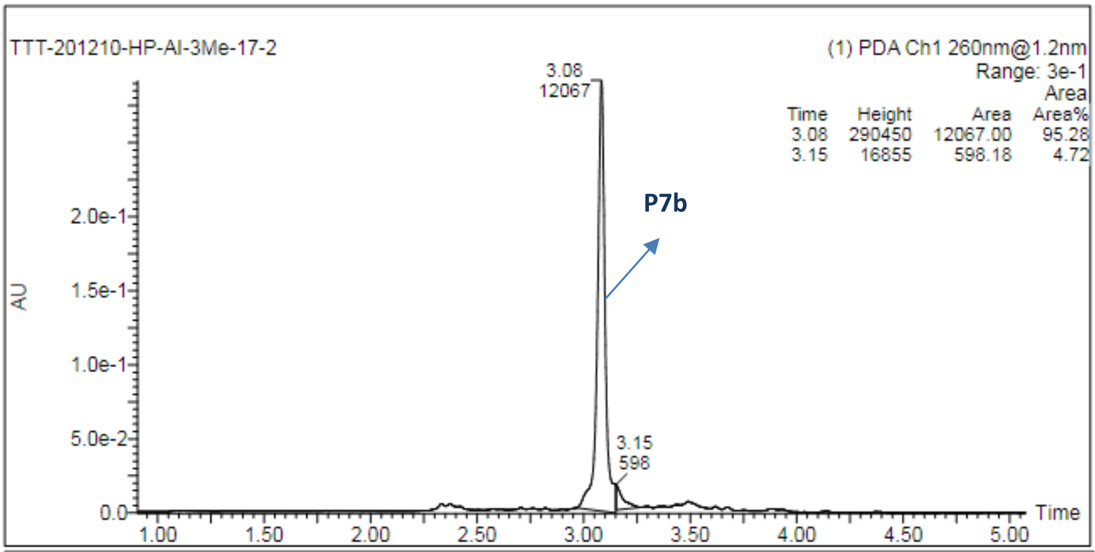

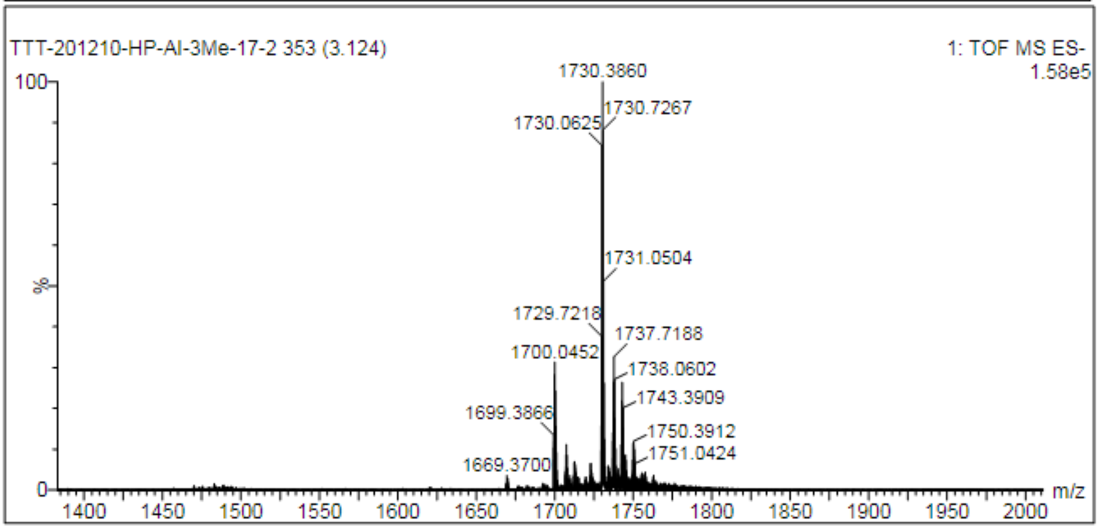

LC Trace and Mass of P7c

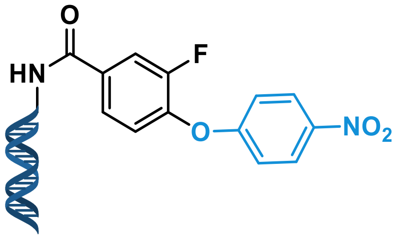

Following General Procedure v

Yield: 81%

Exact mass: 5196.45

Triply charged mass [M]/3 - 1.00794, calculated 1731.14206; observed 1731.3743.

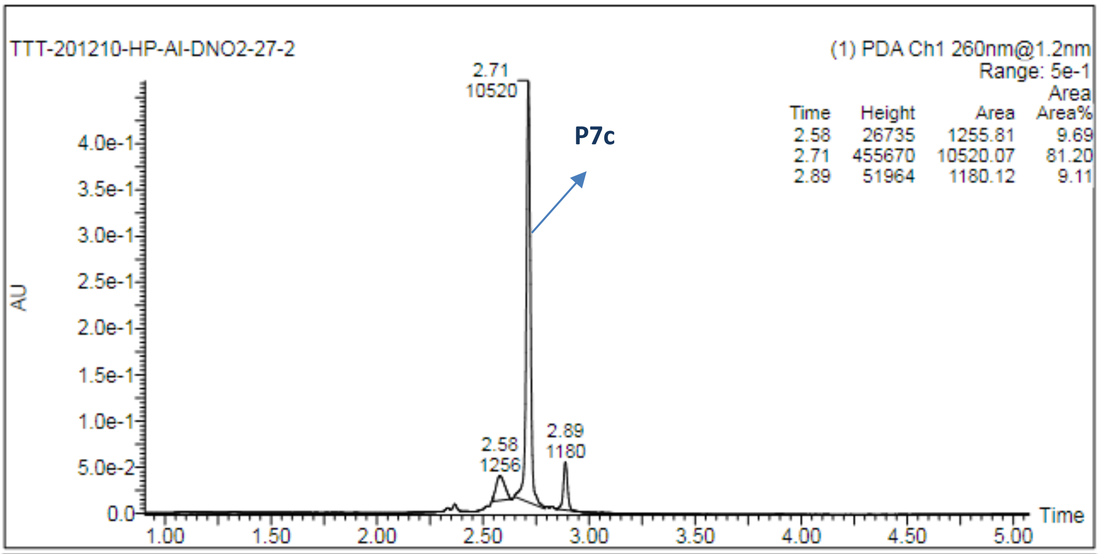

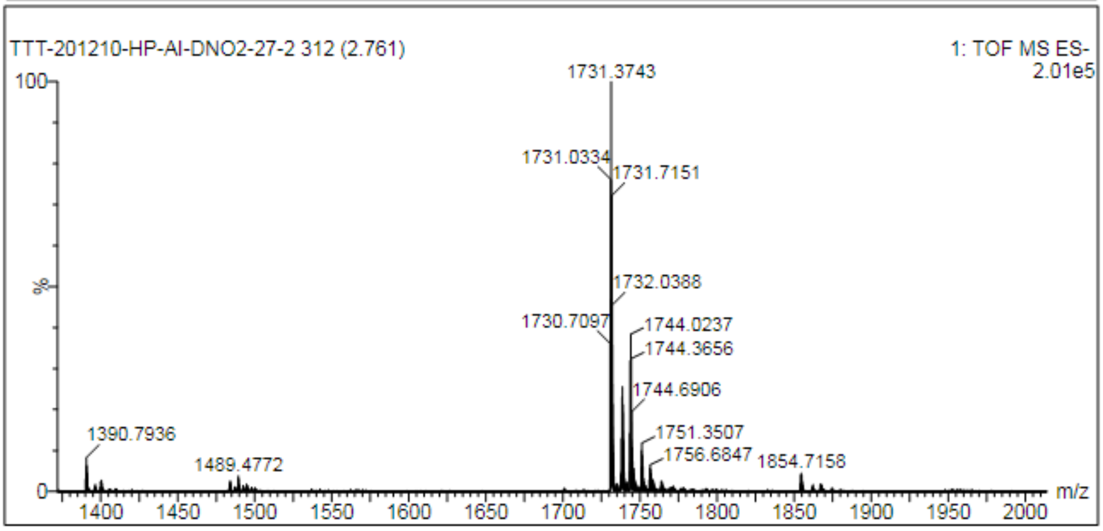

LC Trace and Mass of P8a

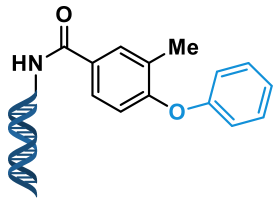

Following General Procedure v
Yield: 95%
Exact mass: 5147.45
Triply charged mass [M]/3 - 1.00794, calculated 1714.80873; observed 1715.0039.

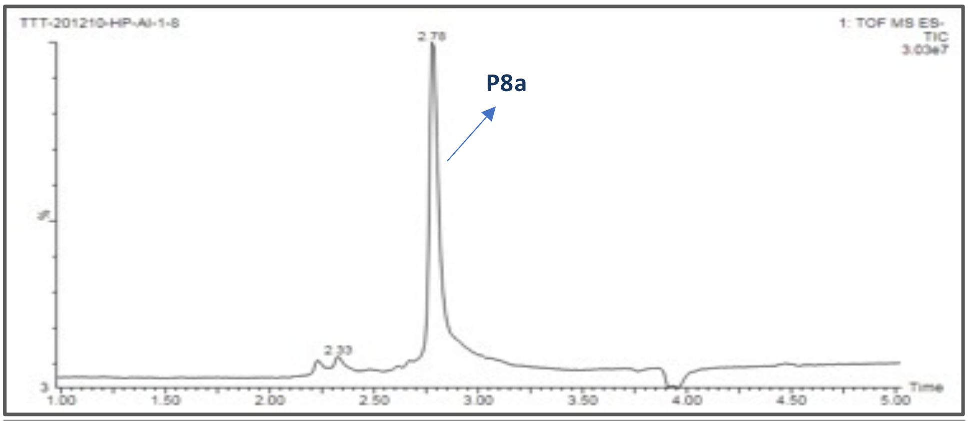

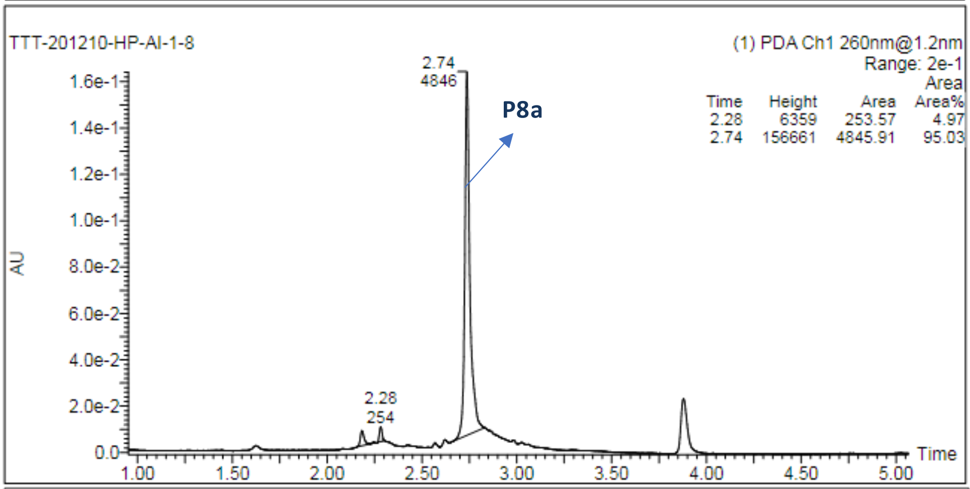

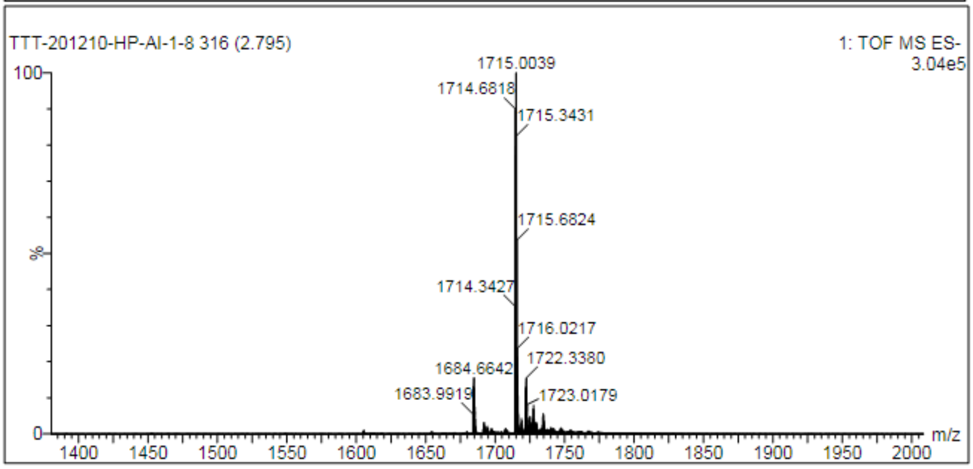

LC Trace and Mass of P8b

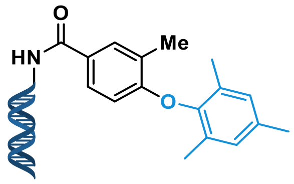

Following General Procedure V
Yield: 95%
Exact mass: 5189.50
Triply charged mass [M]/3 - 1.00794, calculated 1728.82539; observed 1729.0746.

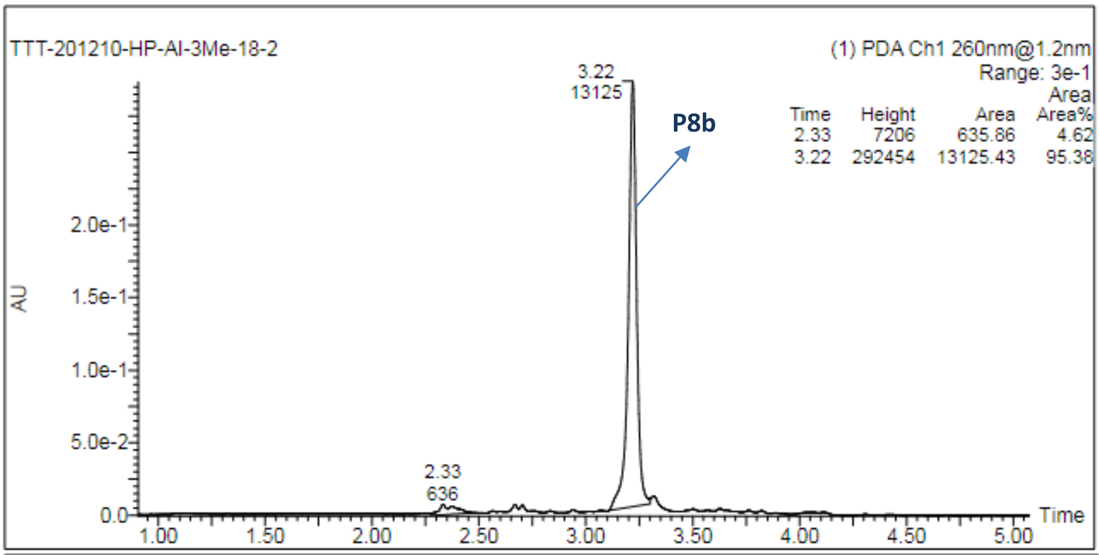

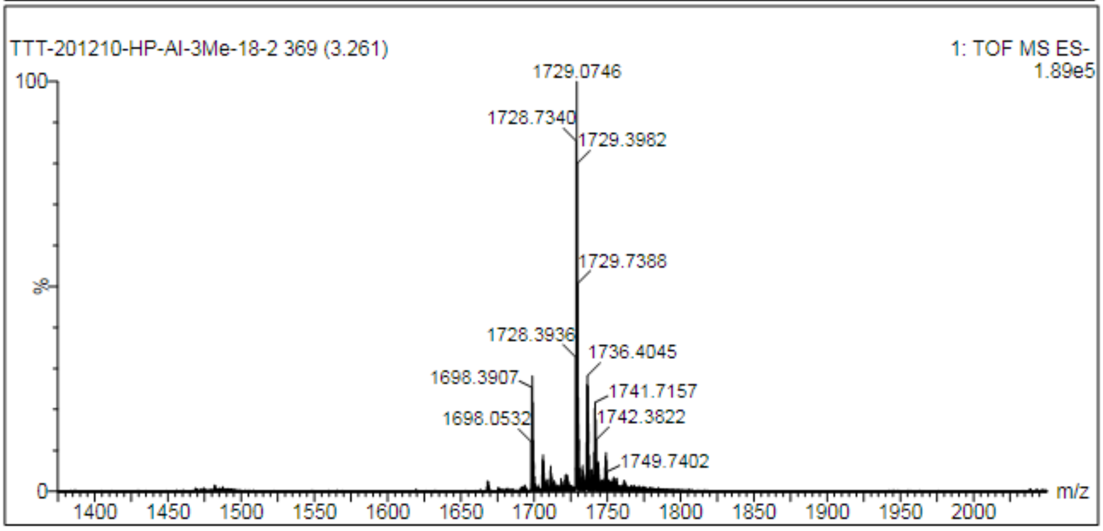

LC Trace and Mass of P8c

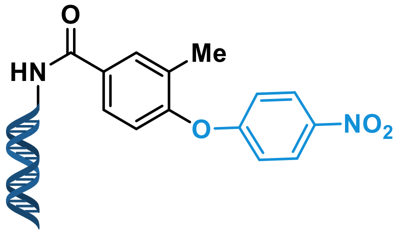

Following General Procedure v

Yield: 89%

Exact mass: 5192.45

Triply charged mass [M]/3 - 1.00794, calculated 1729.80873; observed 1730.0454.

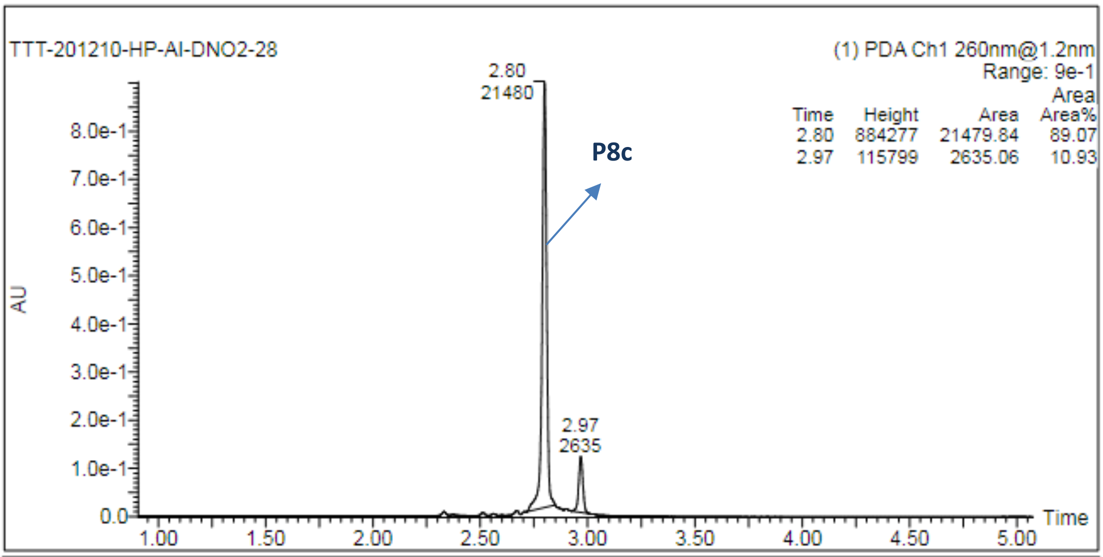

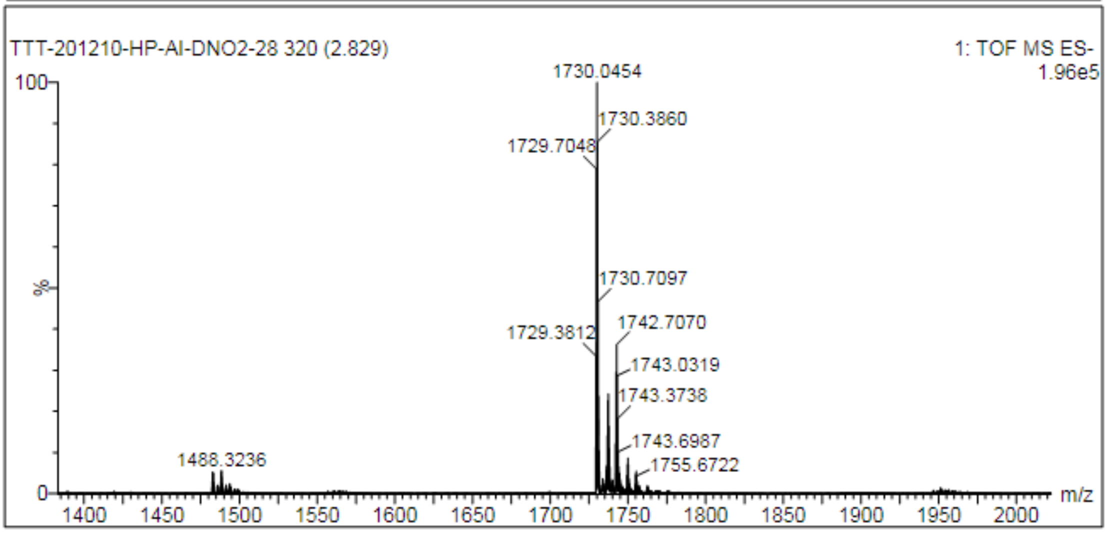

LC Trace and Mass of P9a

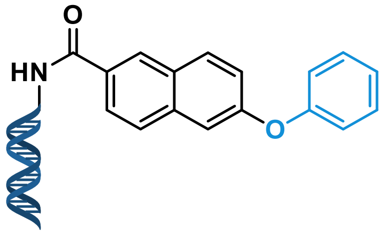

Following General Procedure v

Yield: 15%

Exact mass: 5183.55

Triply charged mass [M]/3 - 1.00794, calculated 1726.84206; observed 1727.0149.

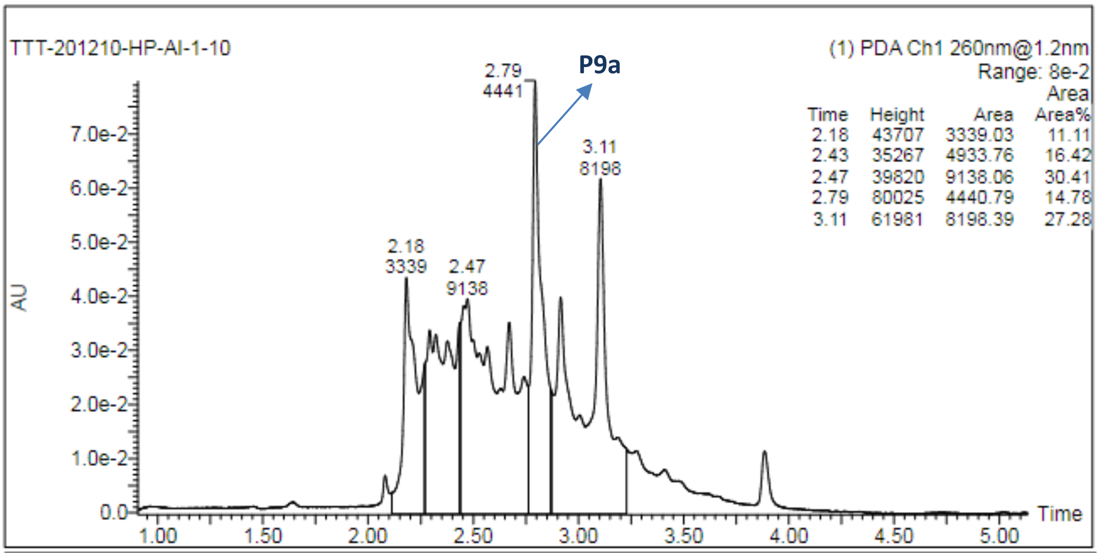

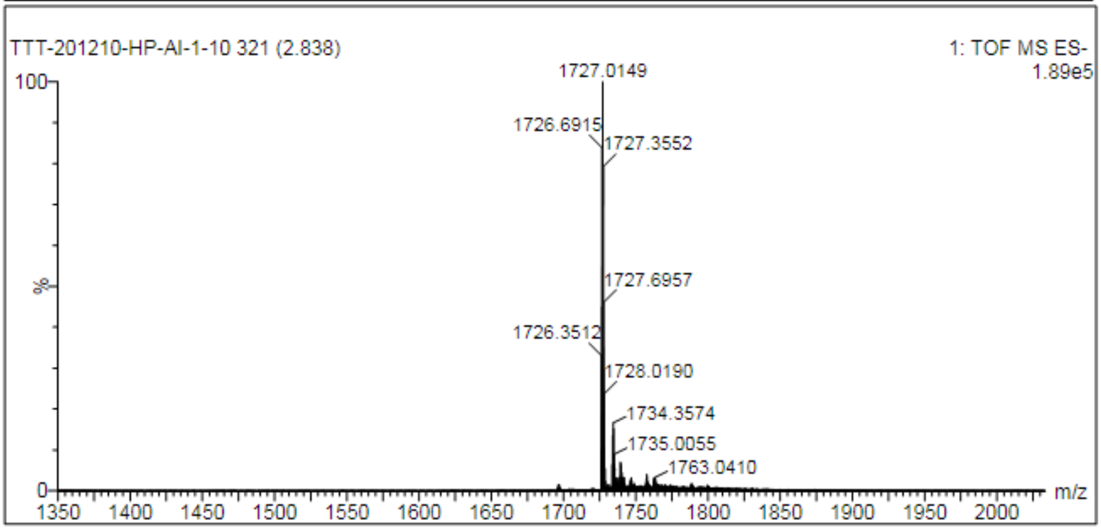

LC Trace and Mass of P9b

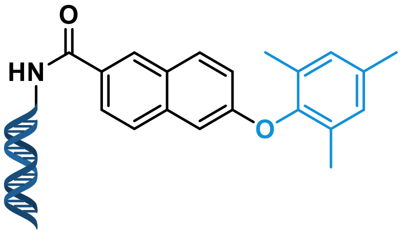

Following General Procedure v

Yield: 80%

Exact mass: 5225.60

Triply charged mass [M]/3 - 1.00794, calculated 1740.85873; observed 1741.0663.

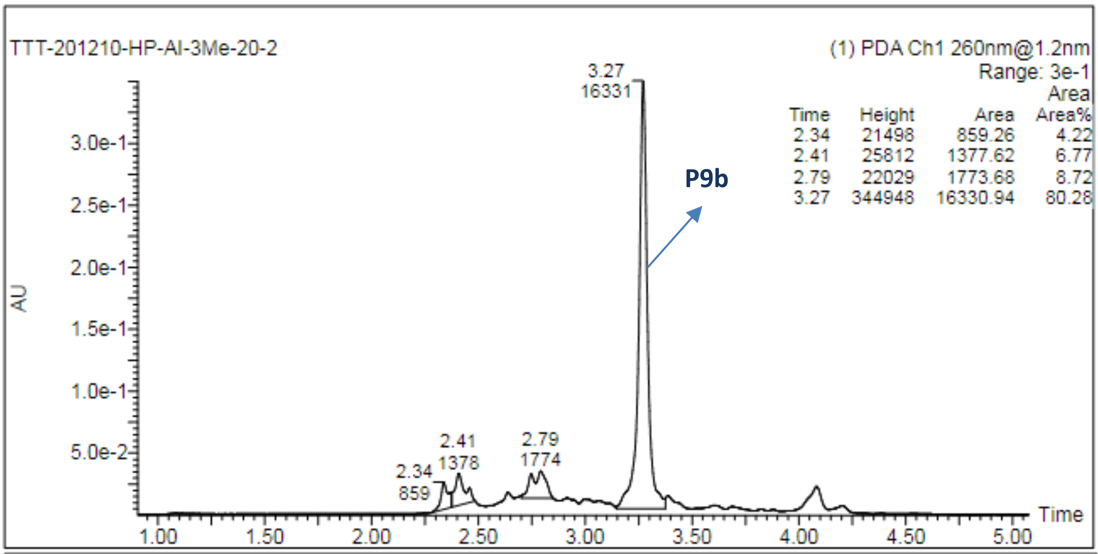

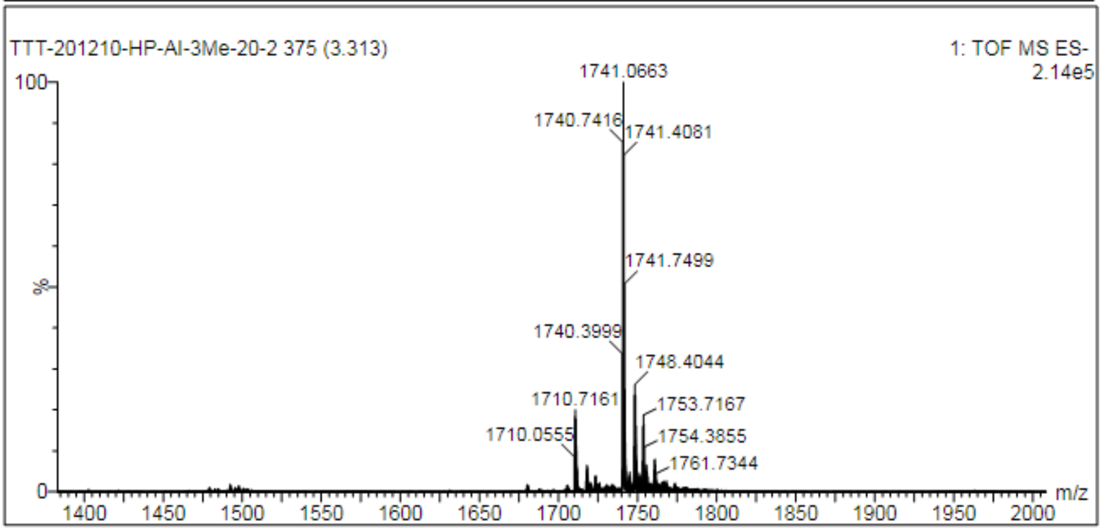

LC Trace and Mass of P9c

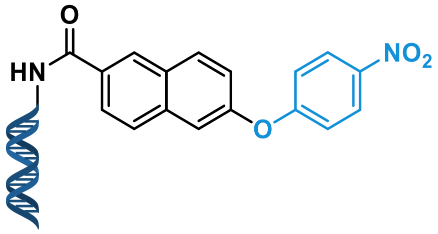

Following General Procedure v

Yield: 41%

Exact mass: 5228.55

Triply charged mass [M]/3 - 1.00794, calculated 1741.84206; observed 1742.0575.

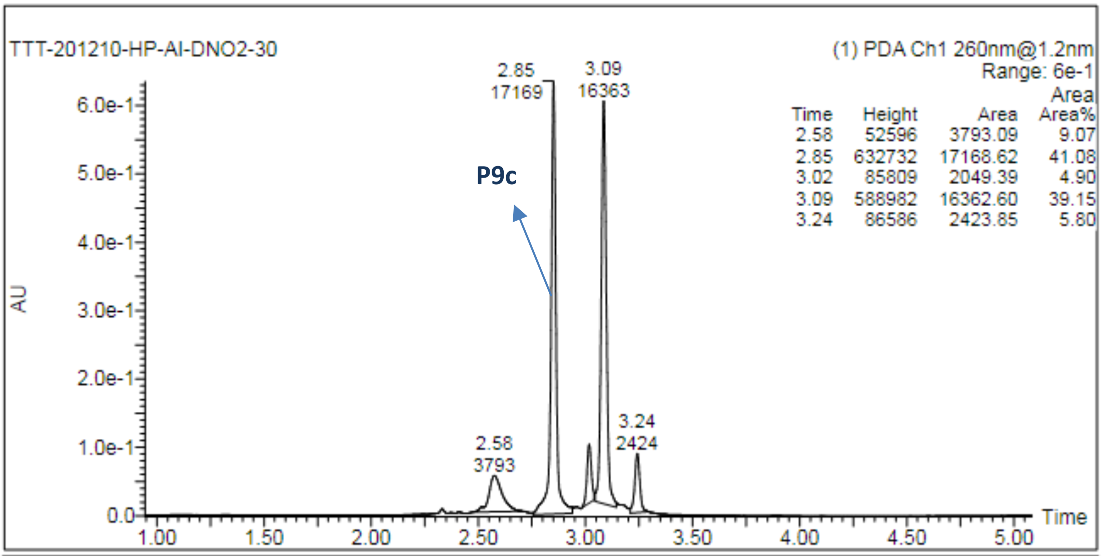

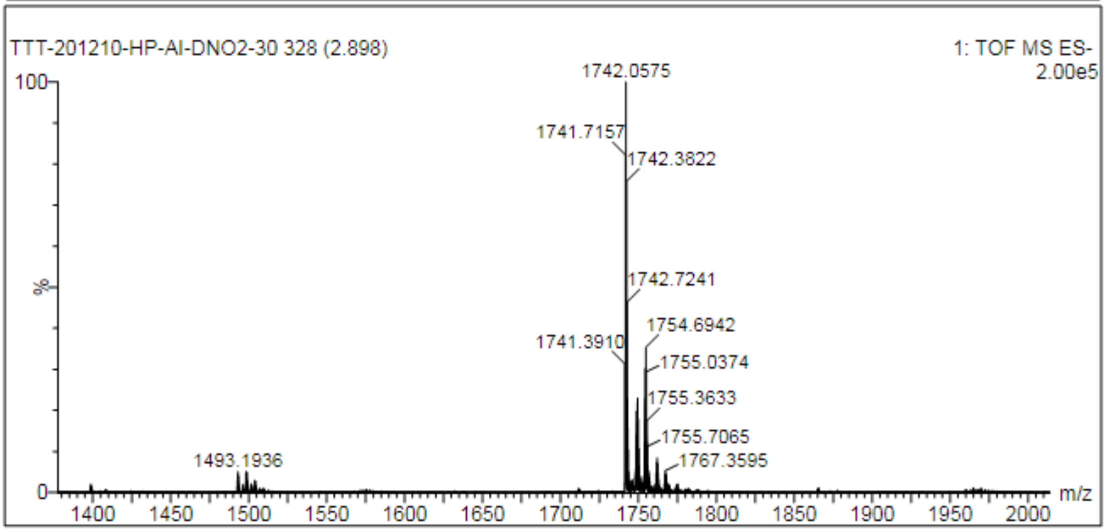

LC Trace and Mass of P10

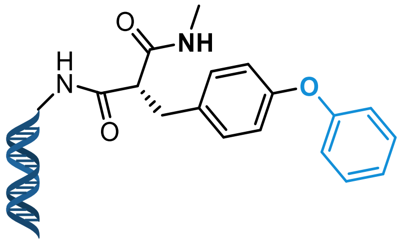

Following General Procedure v

Yield: 78%

Exact mass: 5218.561

Triply charged mass [M]/3 - 1.00794, calculated 1738.5124; observed 1738.3806.

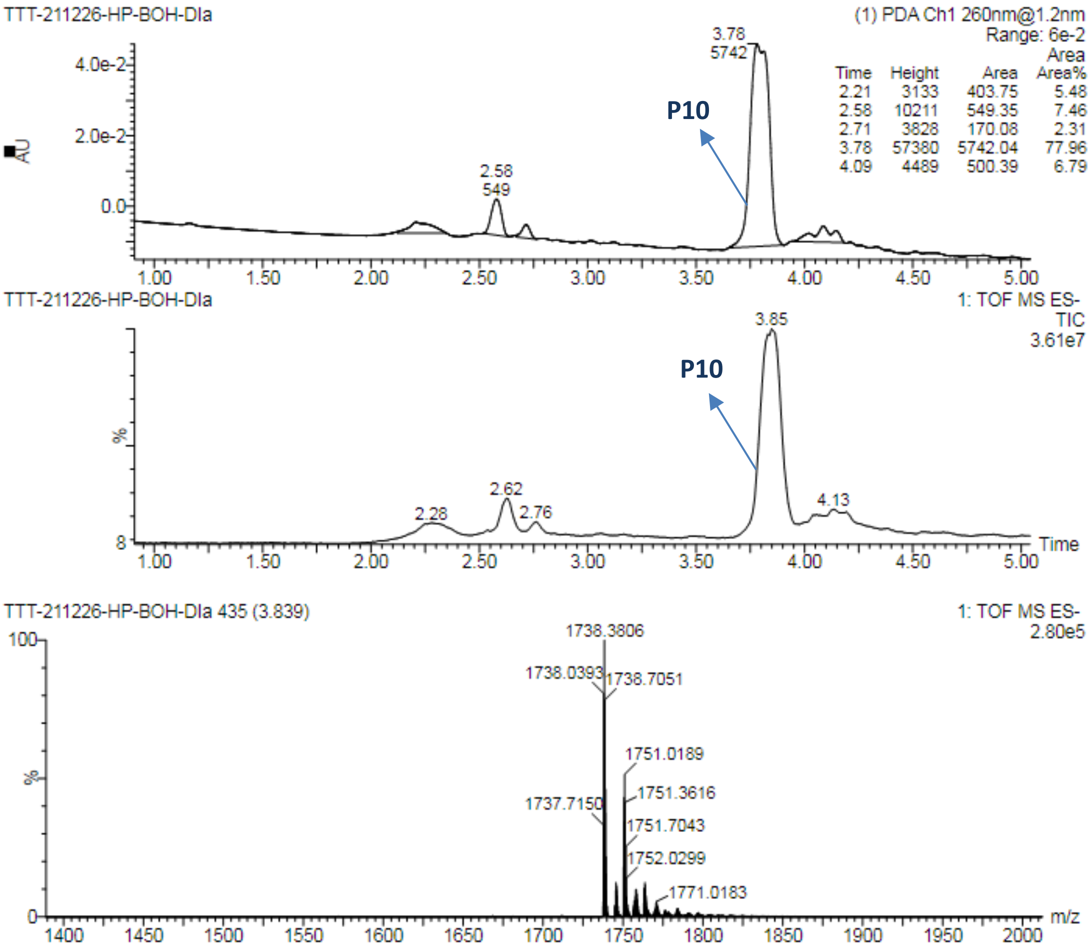

LC Trace and Mass of P11

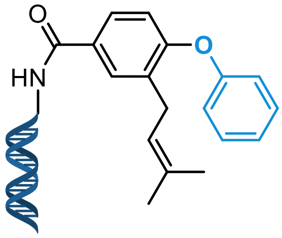

Following General Procedure v

Yield: 75%

Exact mass: 5201.571

Triply charged mass [M]/3 - 1.00794, calculated 1732.8491; observed 1732.5814.

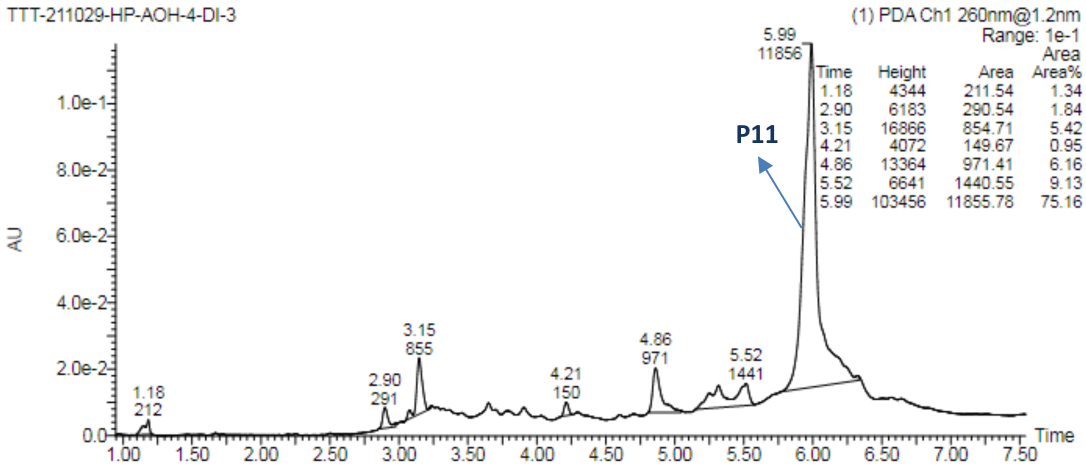

Time	Height	Area	Area Area%
1.18	4344	211.54	1.34
2.90	6183	290.54	1.84
3.15	16866	854.71	5.42
4.21	4072	149.67	0.95
4.86	13364	971.41	6.16
5.52	6641	1440.55	9.13
5.99	103456	11855.78	75.16

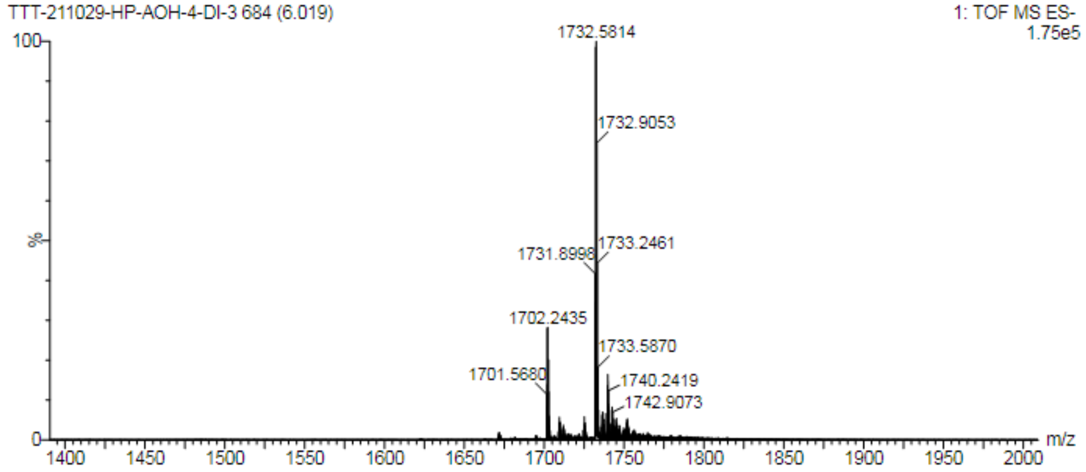

LC Trace and Mass of P12

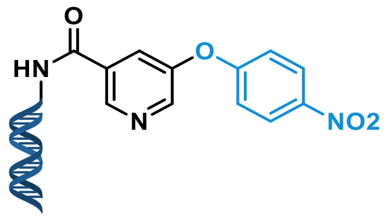

Following General Procedure v

Yield: 75%

Exact mass: 5179.441

Triply charged mass [M]/3 - 1.00794, calculated 1725.4724; observed 1725.3290.

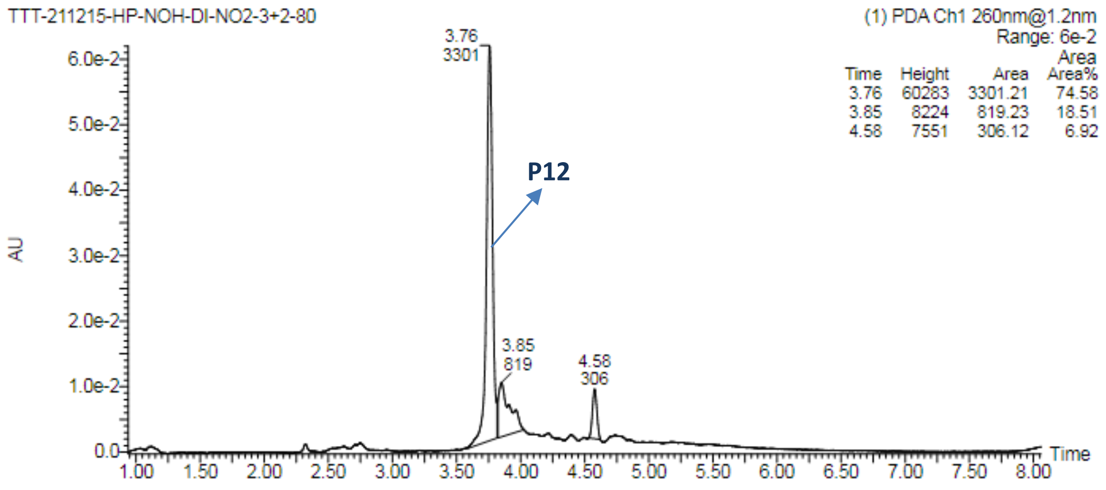

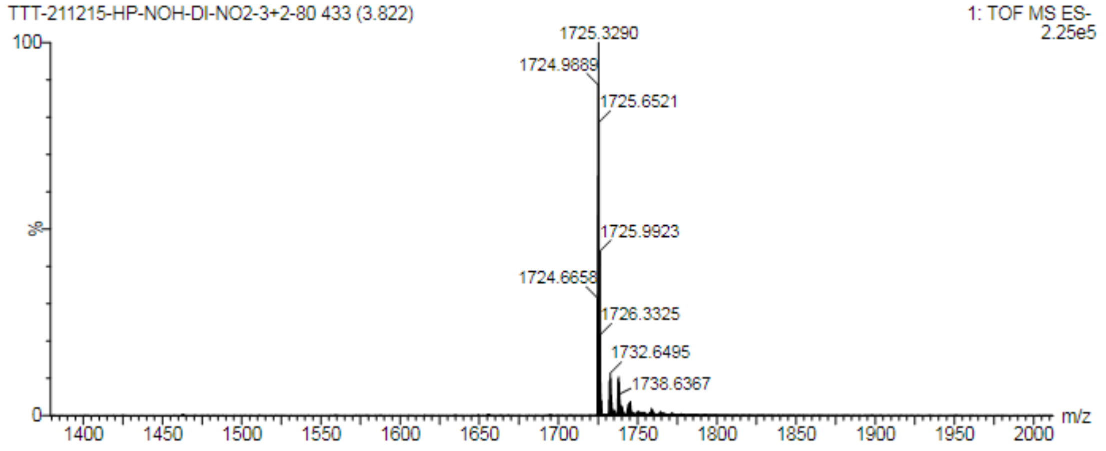

LC Trace and Mass of P13

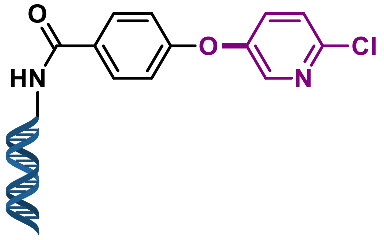

Following General Procedure v

Yield: 66%

Exact mass: 5168.83

Triply charged mass [M]/3 - 1.00794, calculated 1721.9487; observed 1721.6744.

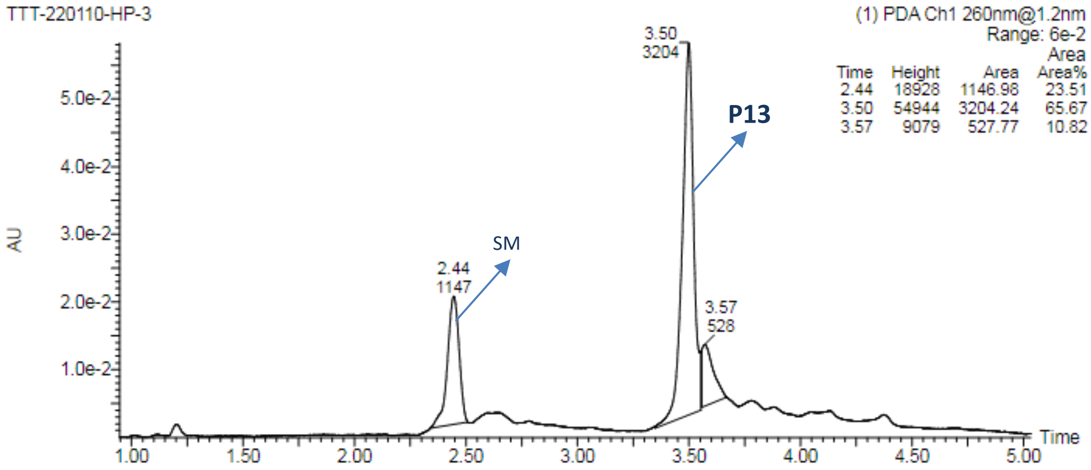

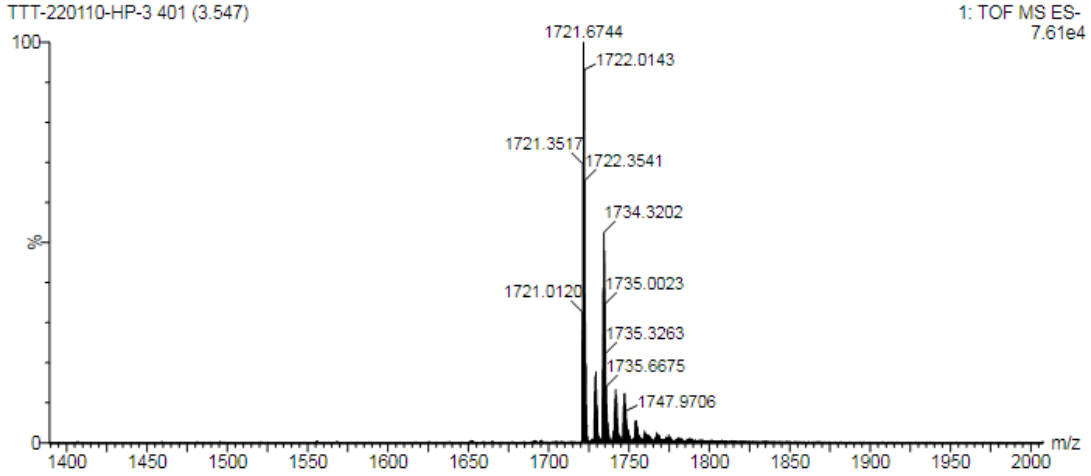

LC Trace and Mass of HP13s0

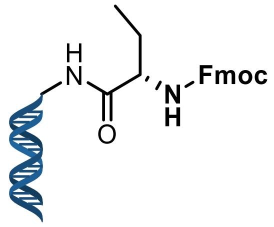

Following General Procedure

Yield: 98%

Exact mass: 5244.591

Triply charged mass [M]/3 - 1.00794, calculated 1747.1891; observed 1747.0464.

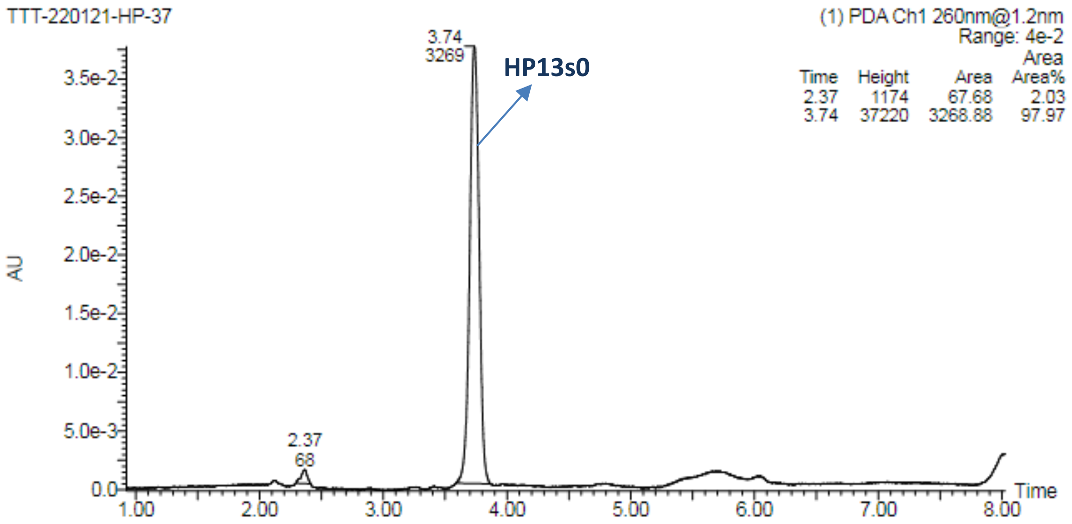

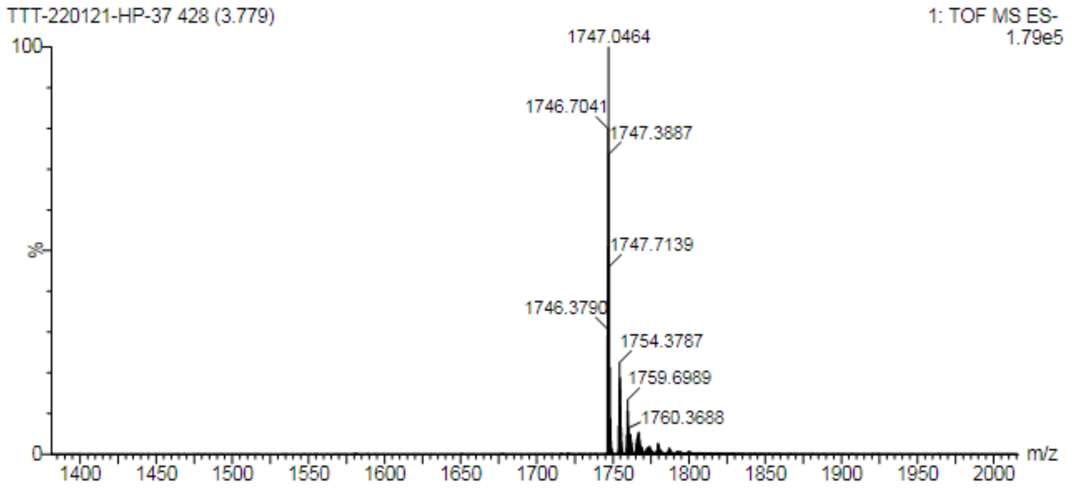

LC Trace and Mass of P13s1

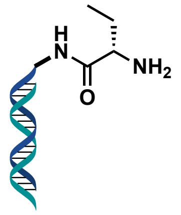

Yield: 99%

Exact mass: 5022.351

Triply charged mass [M]/3 - 1.00794, calculated 1673.1091; observed 1673.0110.

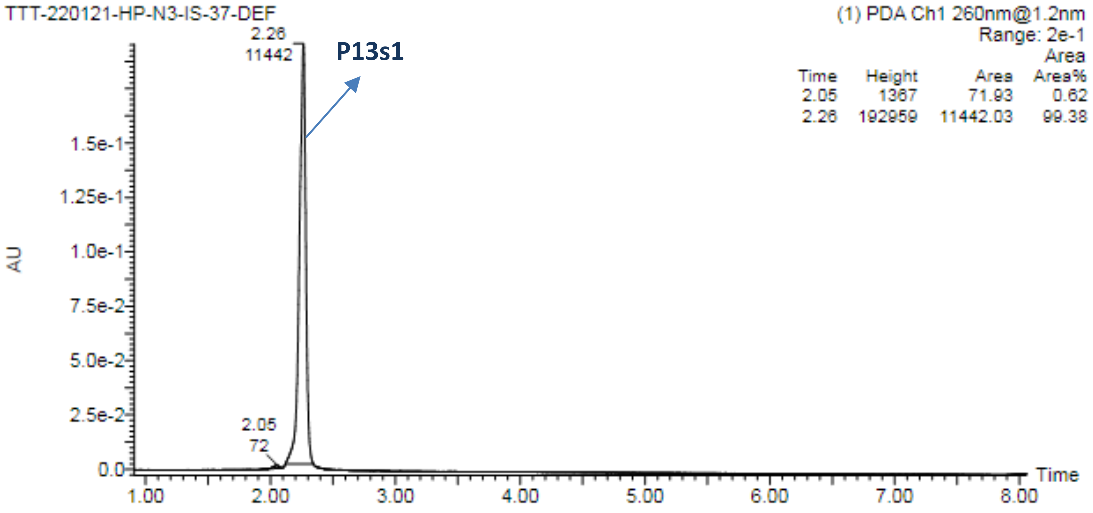

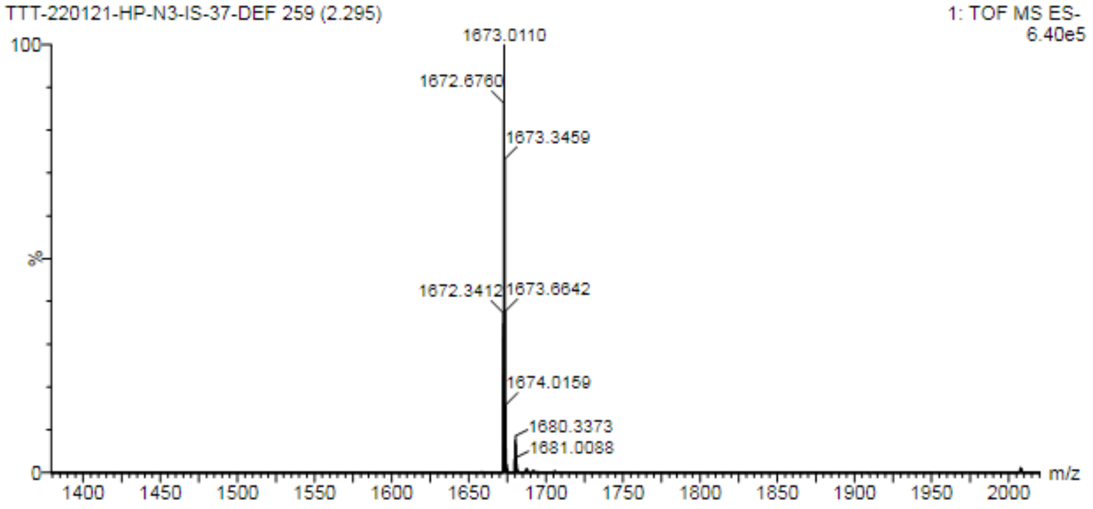

LC Trace and Mass of P13s2

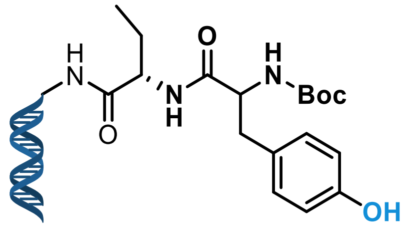

Yield: 73%

Exact mass: 5285.641

Triply charged mass [M]/3 - 1.00794, calculated 1760.8724; observed 1760.7124.

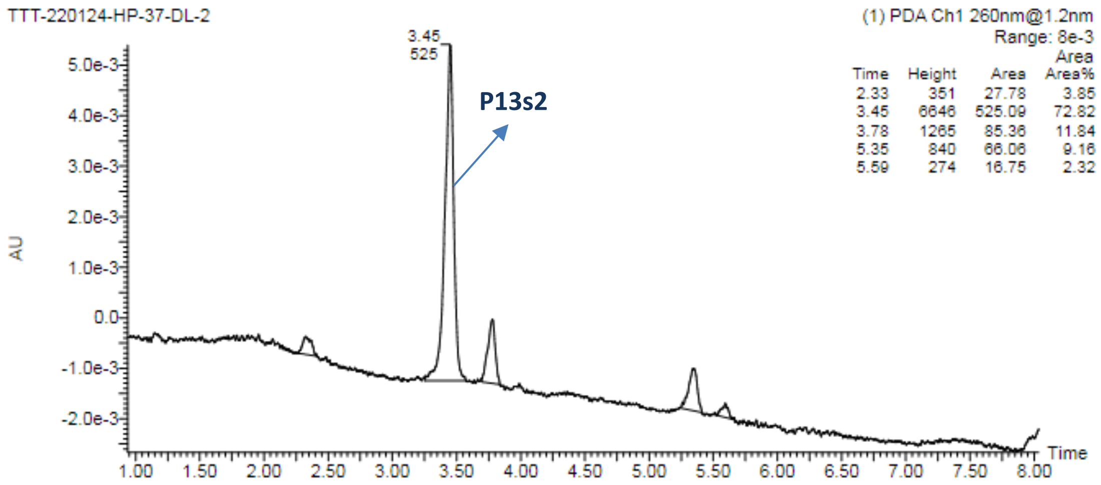

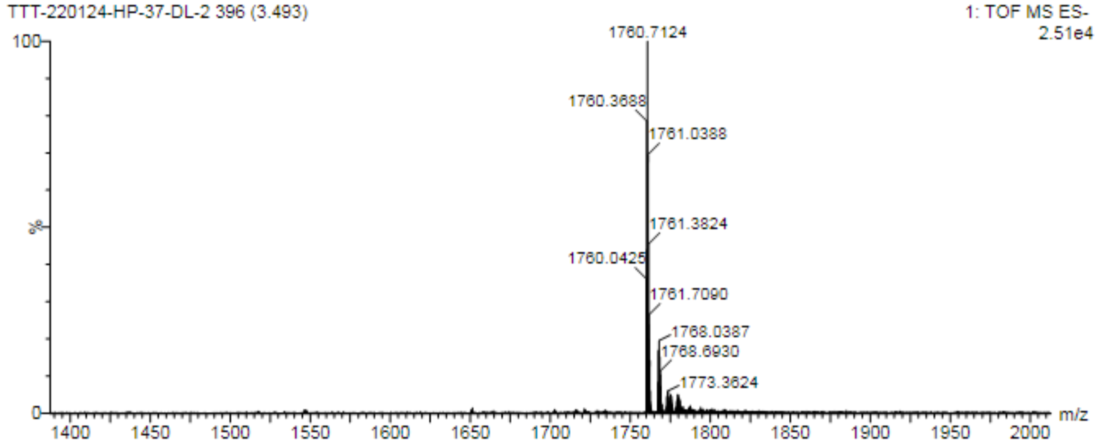

LC Trace and Mass of P13

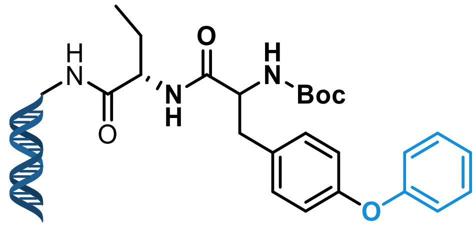

Yield: 84%

Exact mass: 5361.741

Triply charged mass [M]/3 - 1.00794, calculated 1786.2391; observed 1786.0579.

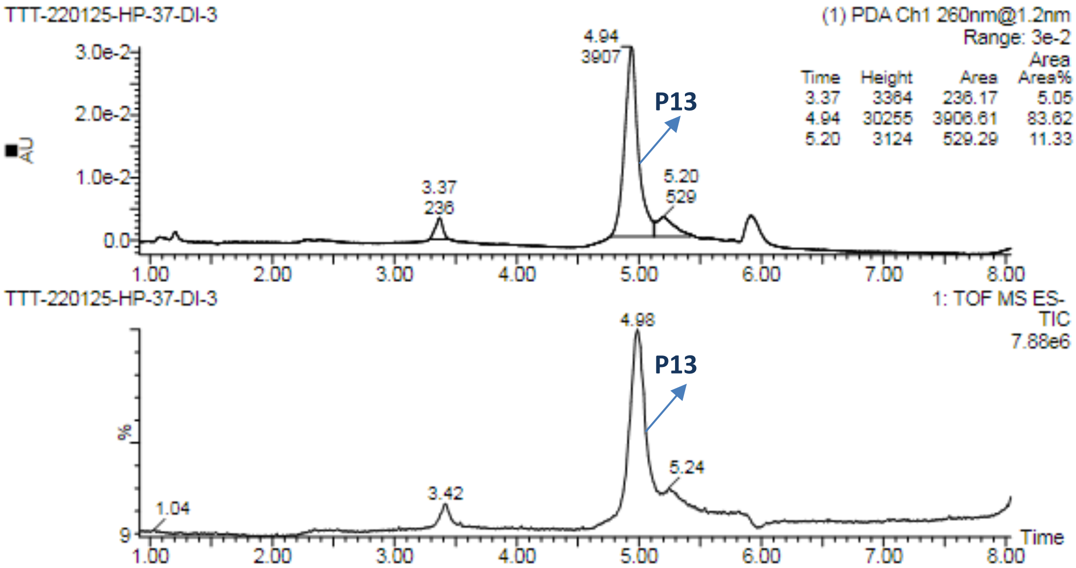

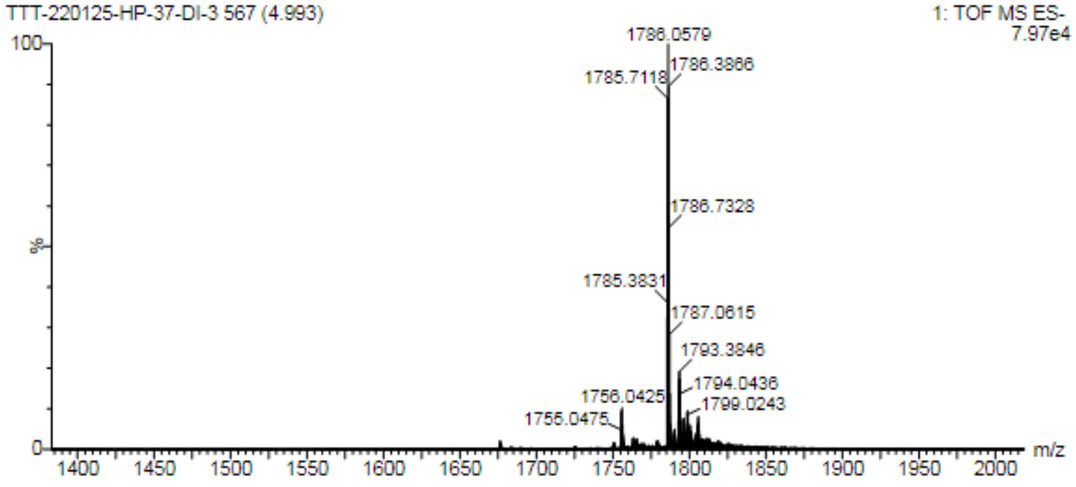

LC Trace and Mass of HP14s0

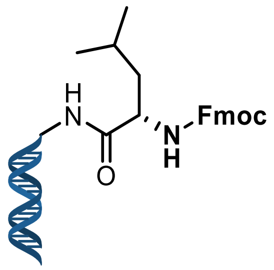

Yield: 100%

Exact mass: 5272.651

Triply charged mass [M]/3 - 1.00794, calculated 1756.5424; observed 1756.3856.

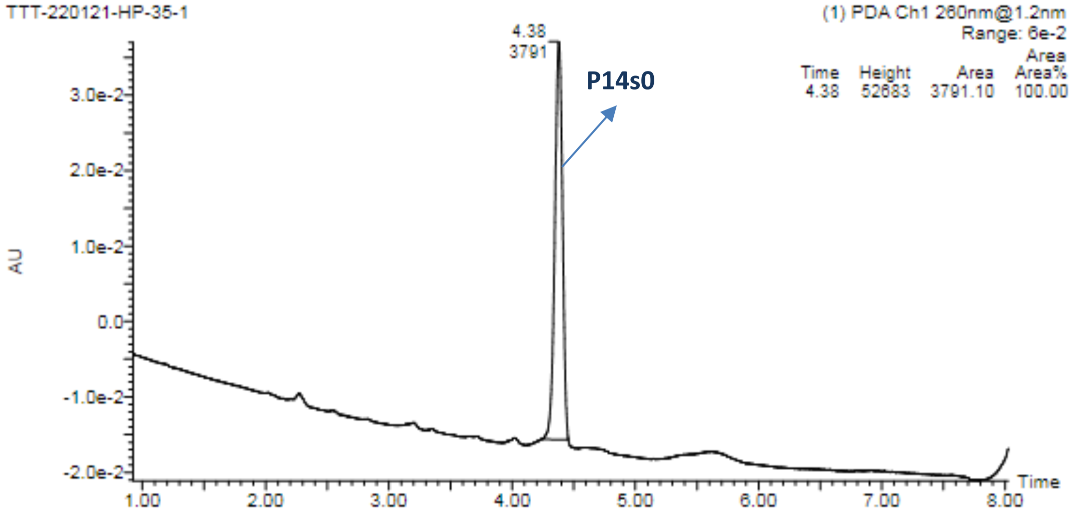

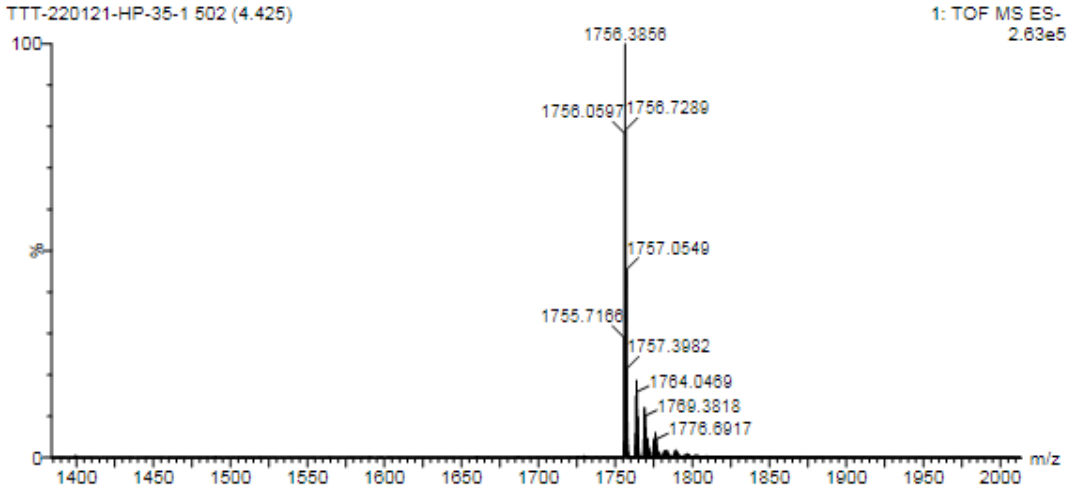

LC Trace and Mass of P14s1

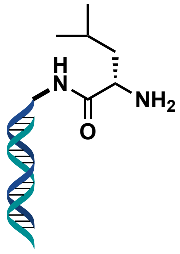

Yield: 98%

Exact mass: 5050.411

Triply charged mass [M]/3 - 1.00794, calculated 1682.4624; observed 1682.3352.

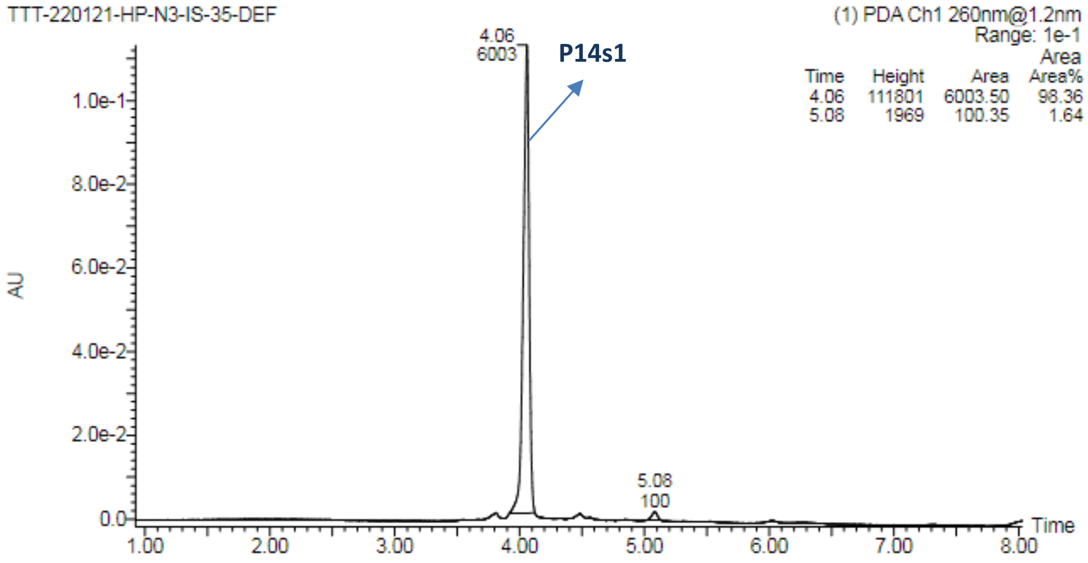

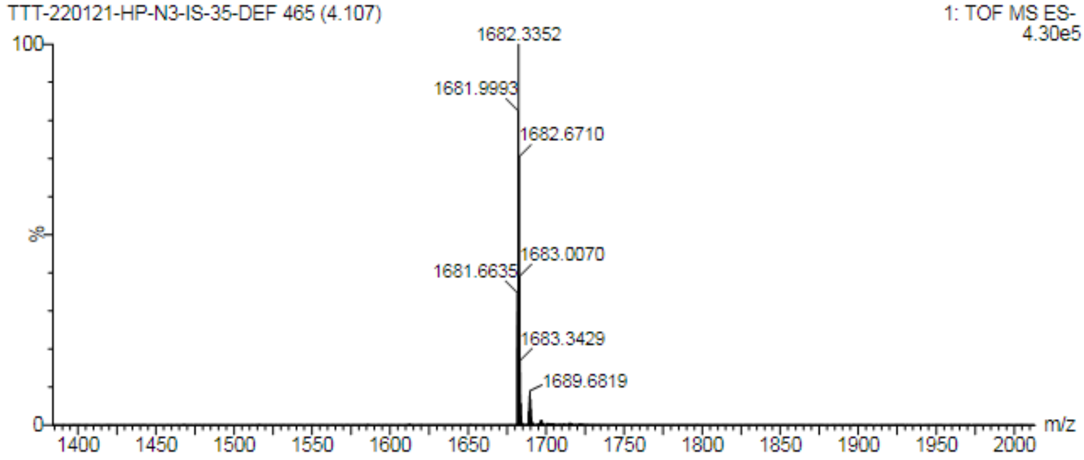

LC Trace and Mass of P14s2

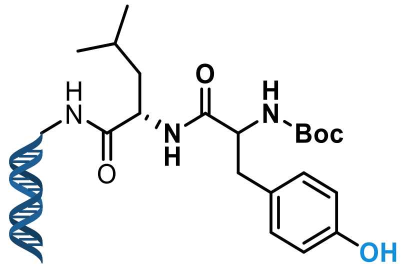

Yield: 78%

Exact mass: 5313.701

Triply charged mass [M]/3 - 1.00794, calculated 1770.2257; observed 1770.0536.

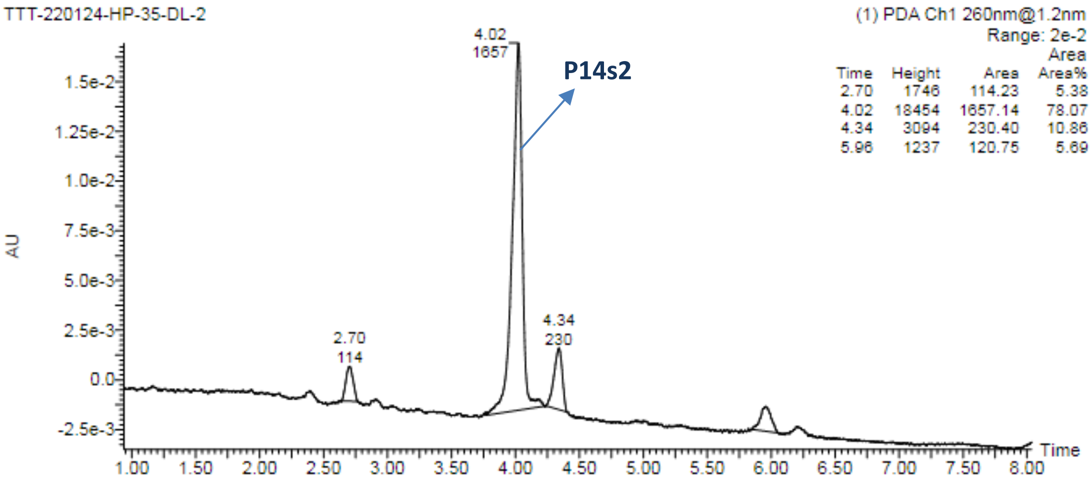

Time	Height	Area	Area%
2.70	1746	114.23	5.38
4.02	18454	1657.14	78.07
4.34	3094	230.40	10.86
5.96	1237	120.75	5.69

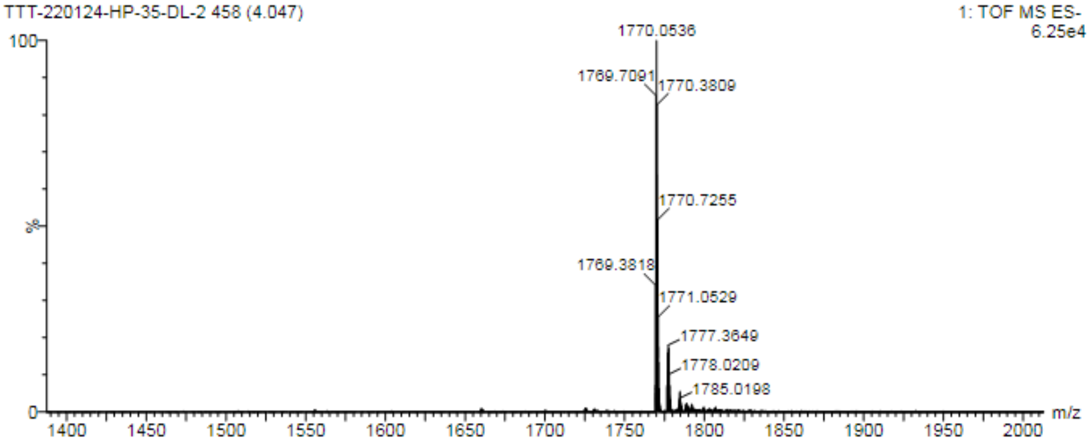

LC Trace and Mass of P14

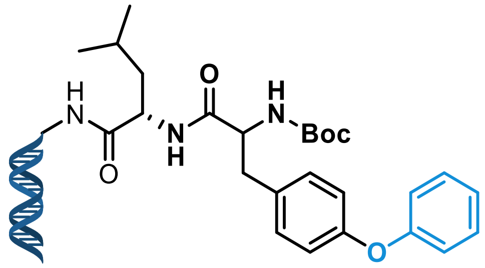

Yield: 79%

Exact mass: 5389.801

Triply charged mass [M]/3 - 1.00794, calculated 1795.5924; observed 1795.3966.

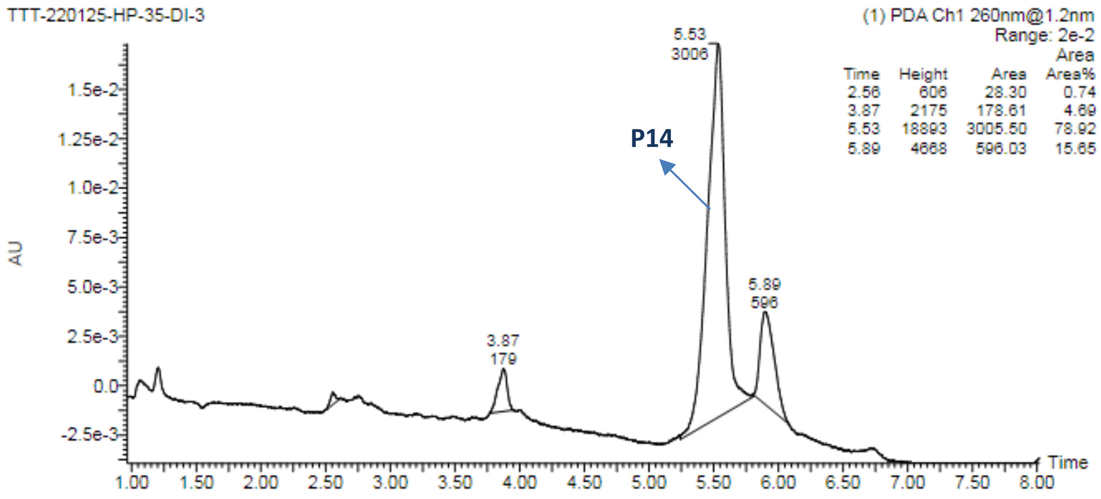

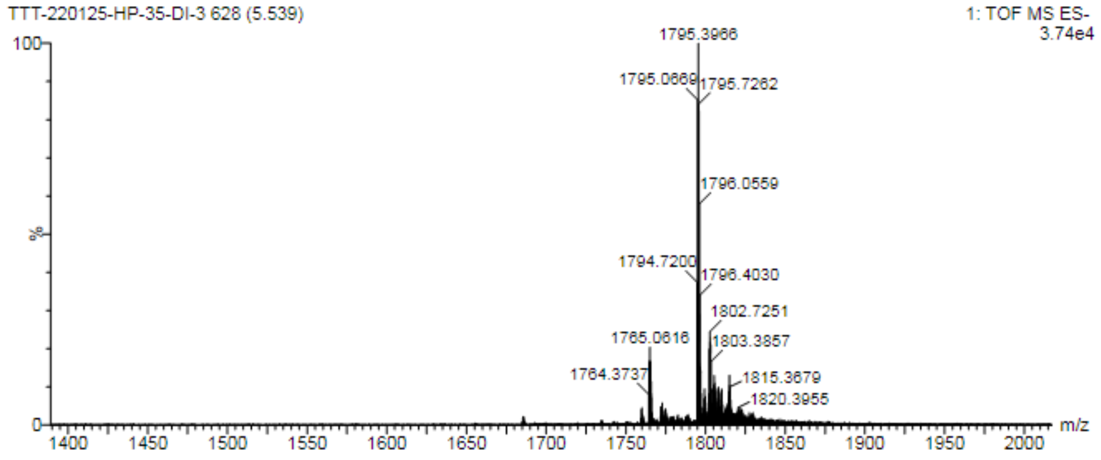

LC Trace and Mass of P15s0

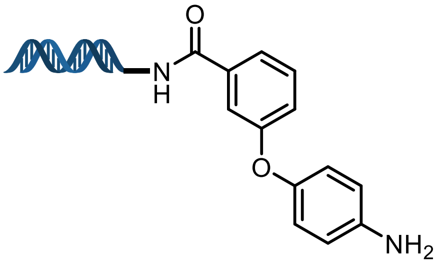

Yield: 94%

Exact mass: 5148.471

Triply charged mass [M]/3 - 1.00794, calculated 1715.1491; observed 1715.0044.

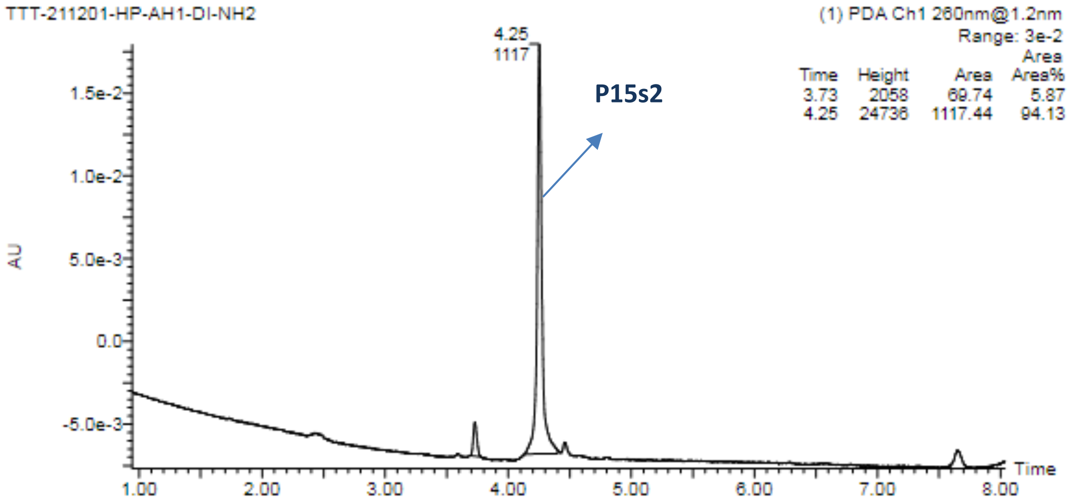

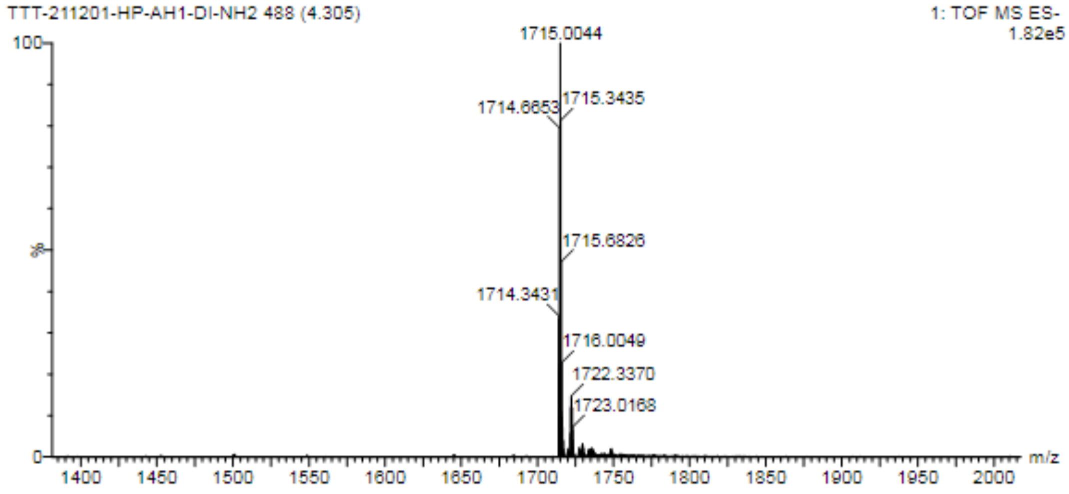

LC Trace and Mass of P15

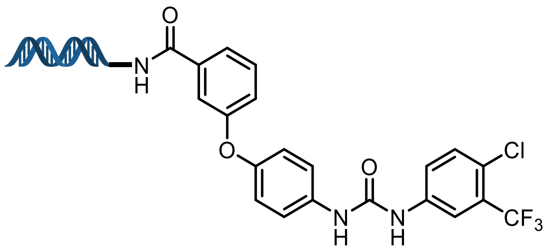

Yield: 90%

Exact mass: 5370.031

Triply charged mass [M]/3 - 1.00794, calculated 1789.0024; observed 1788.9833.

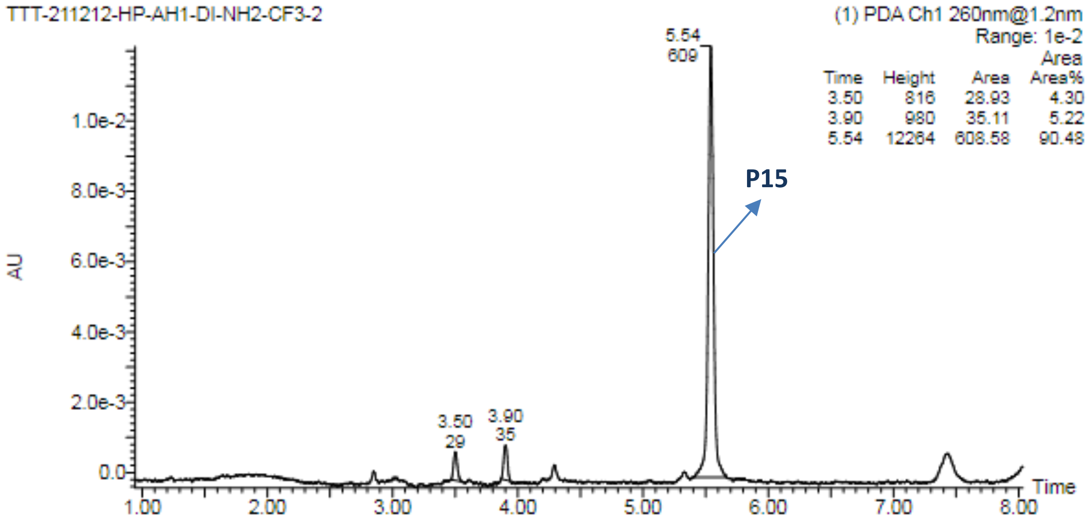

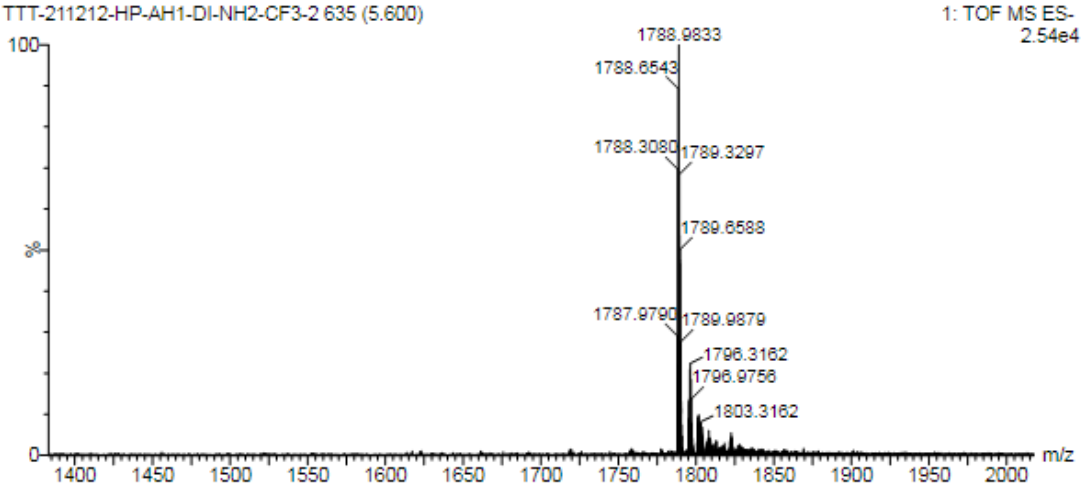

LC Trace and Mass of P16s0

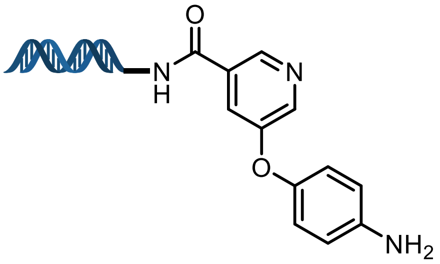

Yield: 85%

Exact mass: 5149.451

Triply charged mass [M]/3 - 1.00794, calculated 1715.4757; observed 1715.3265.

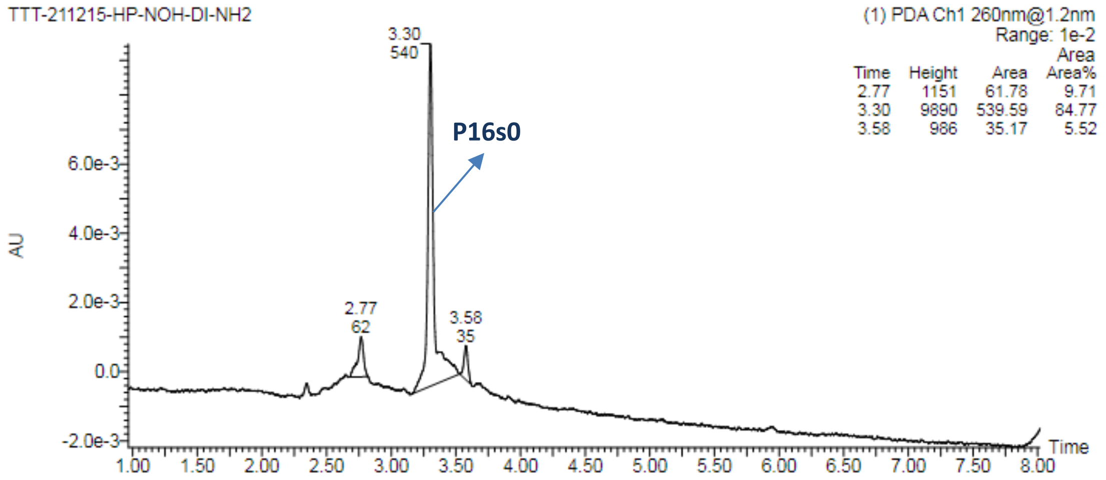

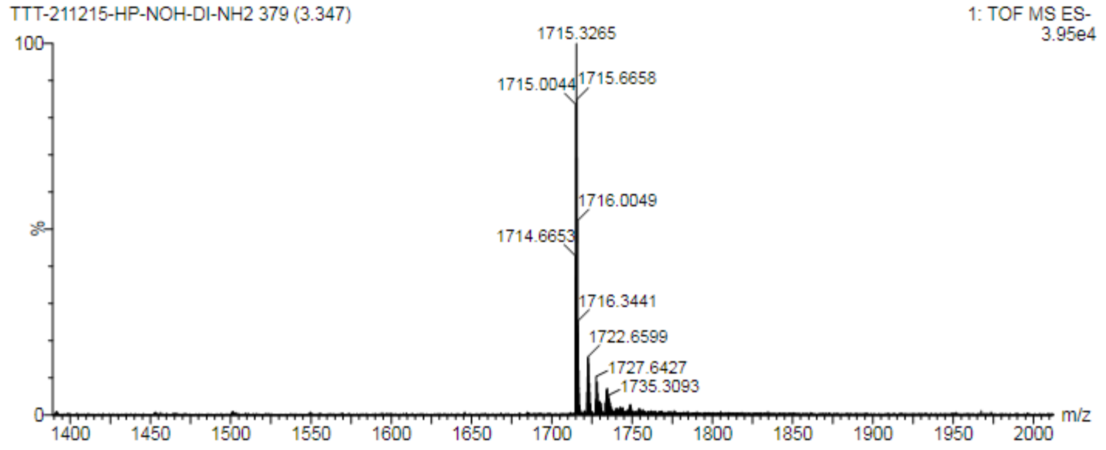

LC Trace and Mass of P16

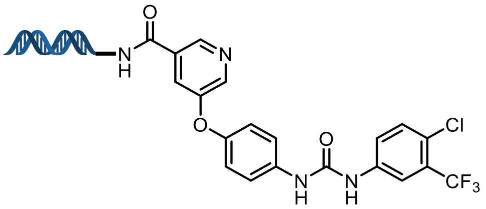

Yield: 90%

Exact mass: 5371.021

Triply charged mass [M]/3 - 1.00794, calculated 1789.3324; observed 1789.3124.

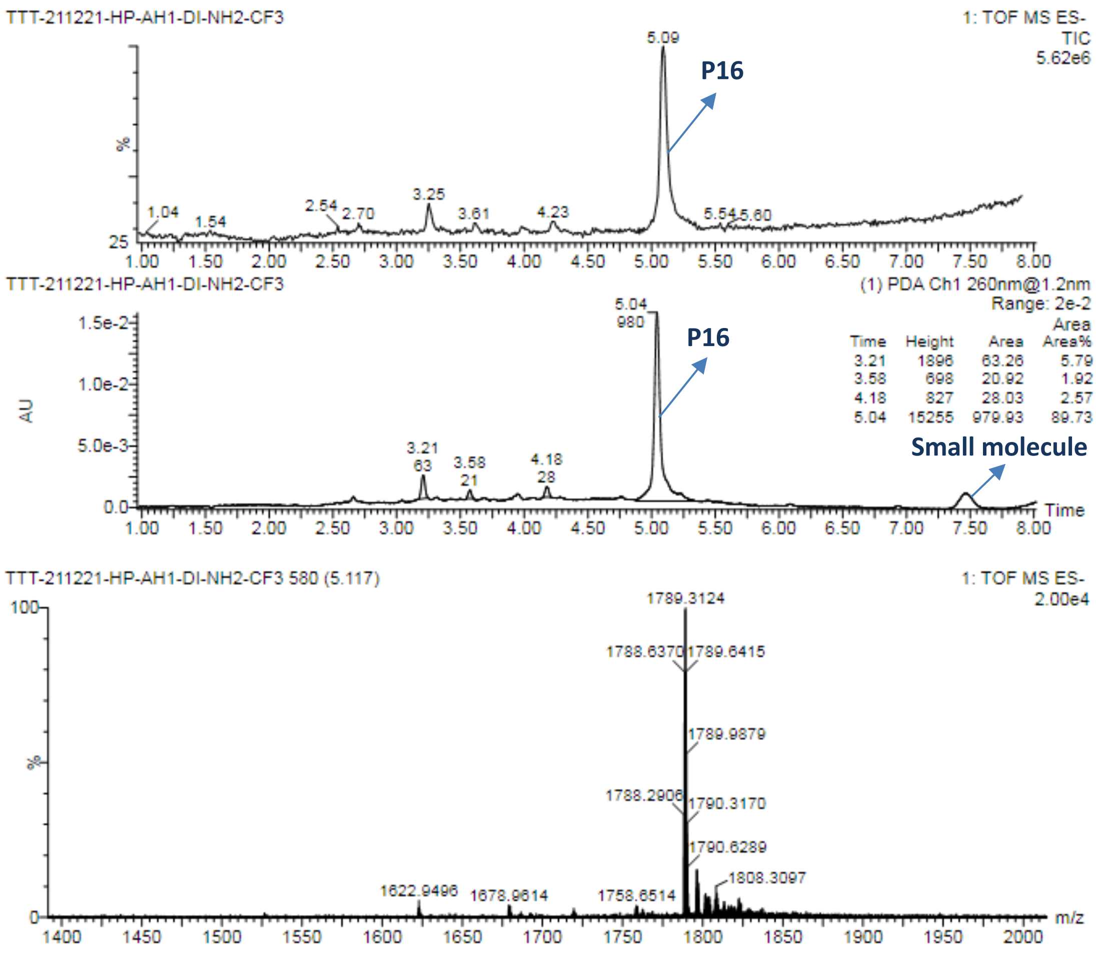

LC Trace and Mass of HP-N_3

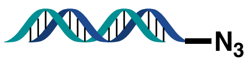

Yield: 97%

Exact mass: 4963.231

Triply charged mass [M]/3 - 1.00794, calculated 1653.4024; observed 1653.3247.

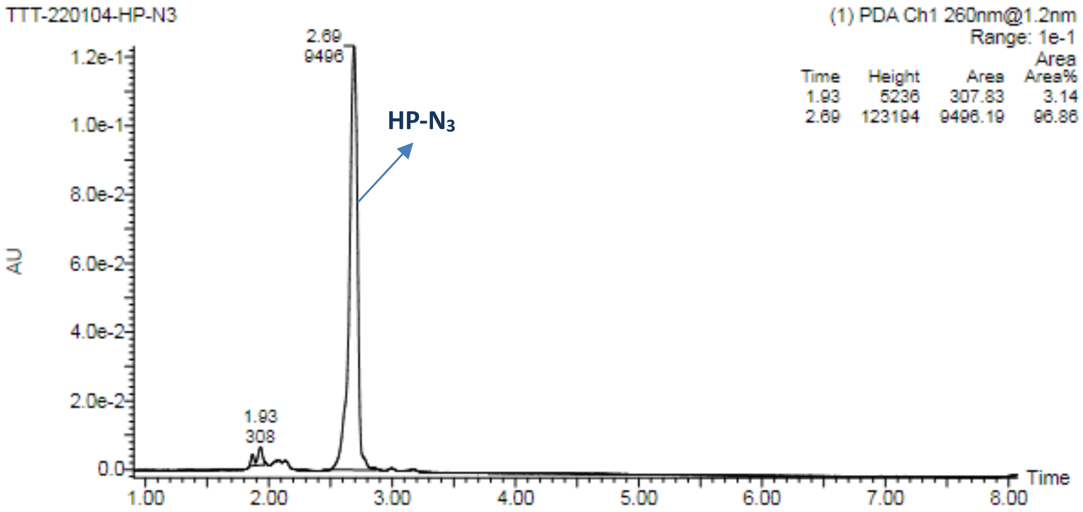

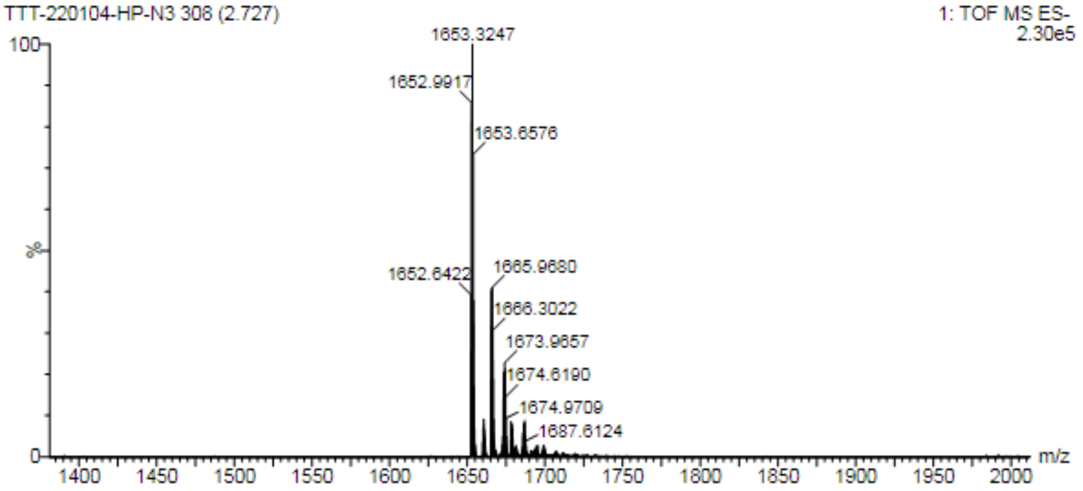

LC Trace and Mass of HO1

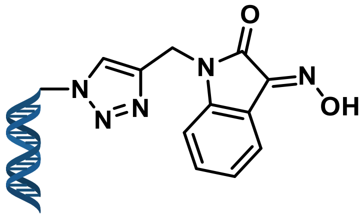

Yield: 90%

Exact mass: 5163.431

Triply charged mass [M]/3 - 1.00794, calculated 1720.1357; observed 1720.0439.

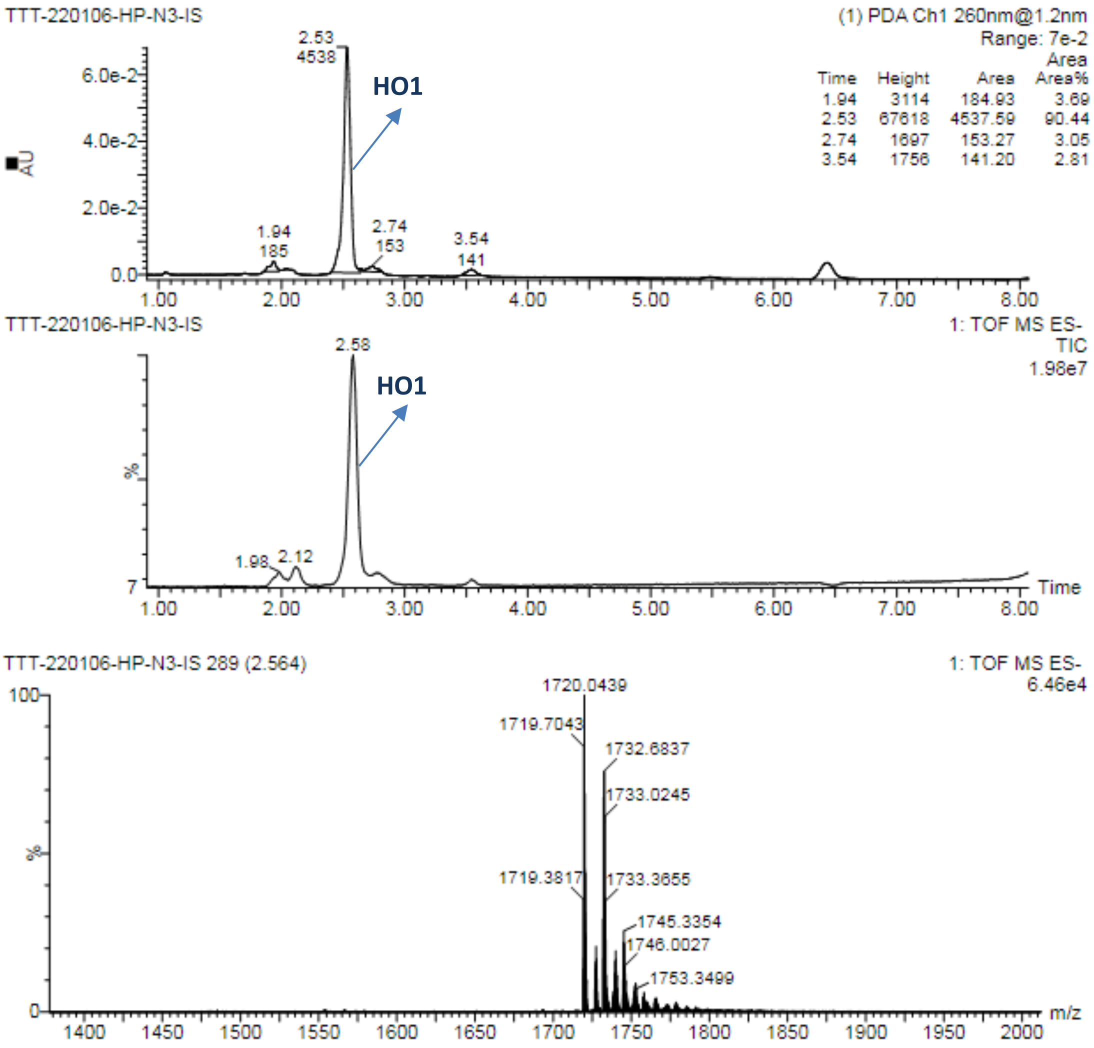

LC Trace and Mass of HO2

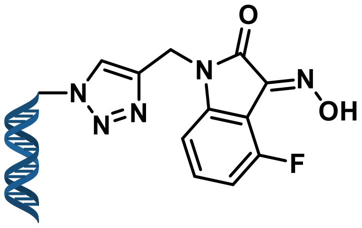

Yield: 81%

Exact mass: 5181.421

Triply charged mass [M]/3 - 1.00794, calculated 1726.1324; observed 1726.0093.

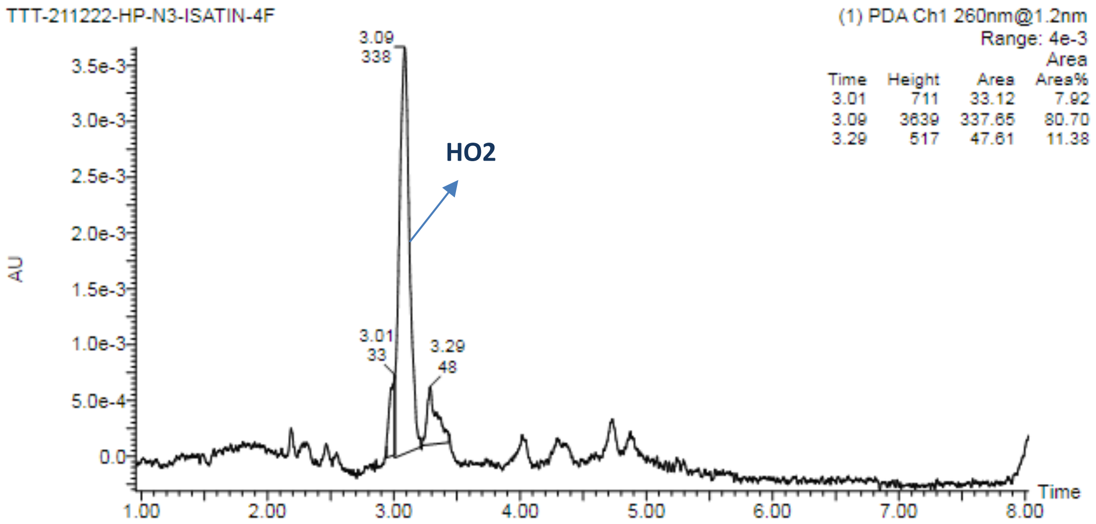

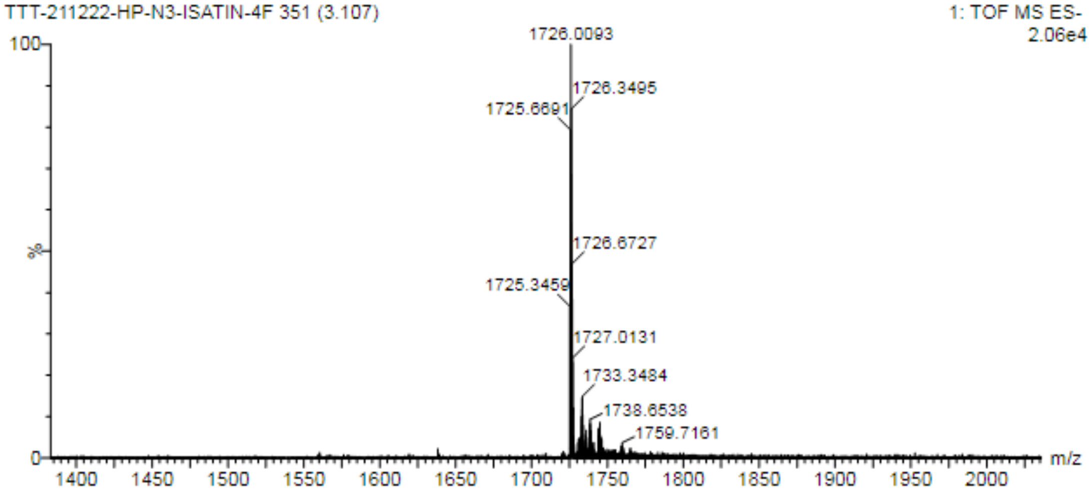

LC Trace and Mass of HO3

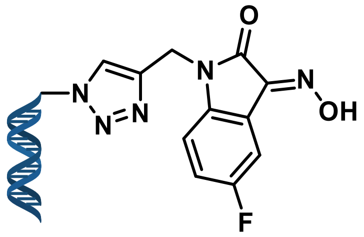

Yield: 89%

Exact mass: 5181.421

Triply charged mass [M]/3 - 1.00794, calculated 1726.1324; observed 1725.7882.

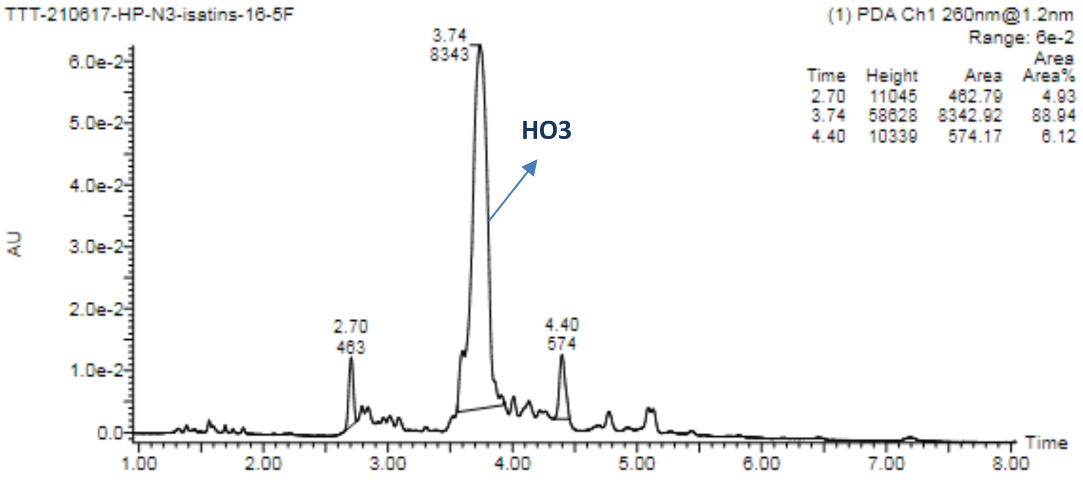

Time	Height	Area	Area%
2.70	11045	462.79	4.93
3.74	58628	8342.92	88.94
4.40	10339	574.17	6.12

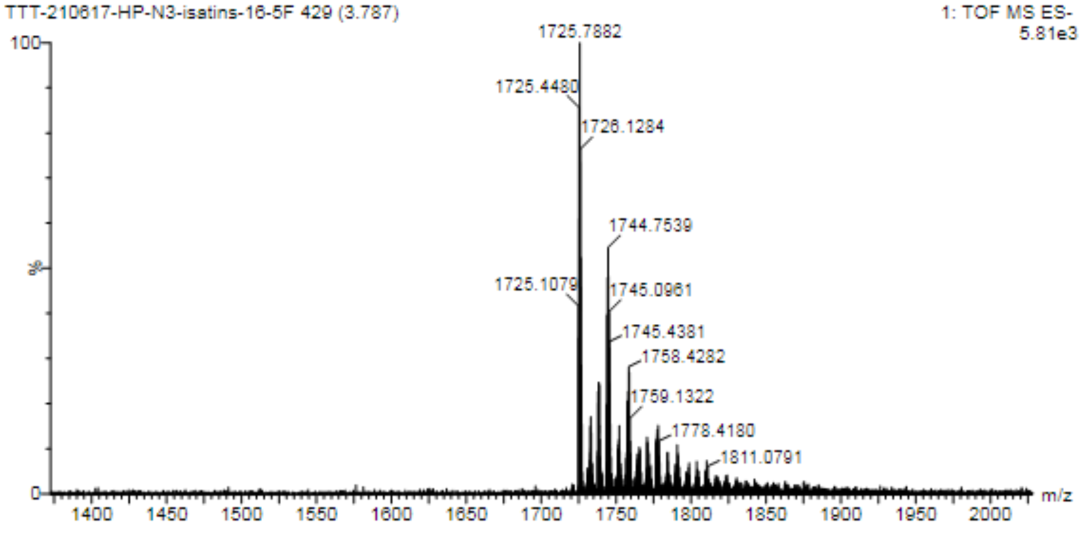

LC Trace and Mass of HO4

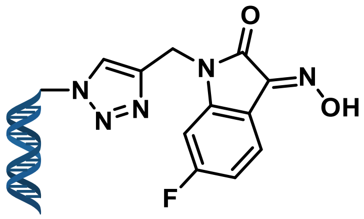

Yield: 84%

Exact mass: 5181.421

Triply charged mass [M]/3 - 1.00794, calculated 1726.1324; observed 1725.7882.

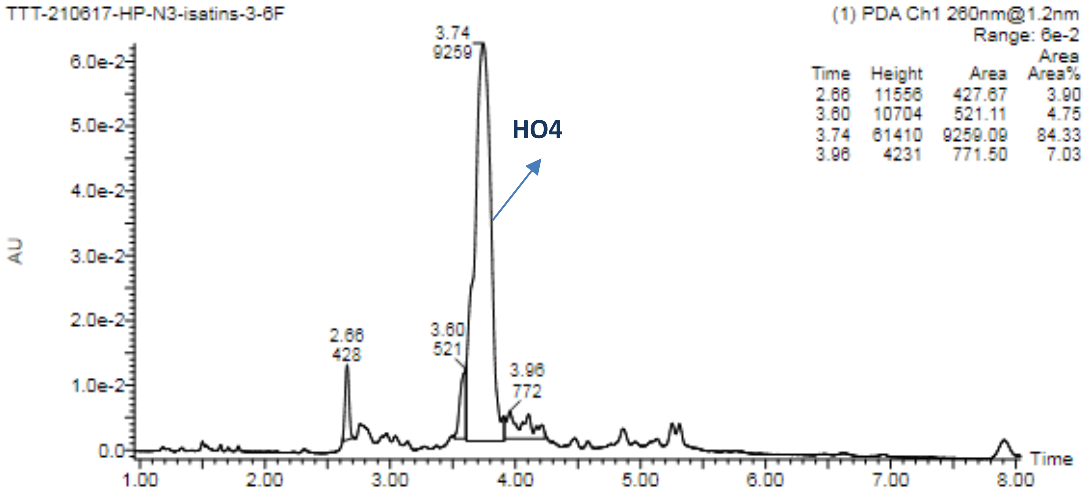

Time	Height	Area	Area%
2.66	11556	427.67	3.90
3.60	10704	521.11	4.75
3.74	61410	9259.09	84.33
3.96	4231	771.50	7.03

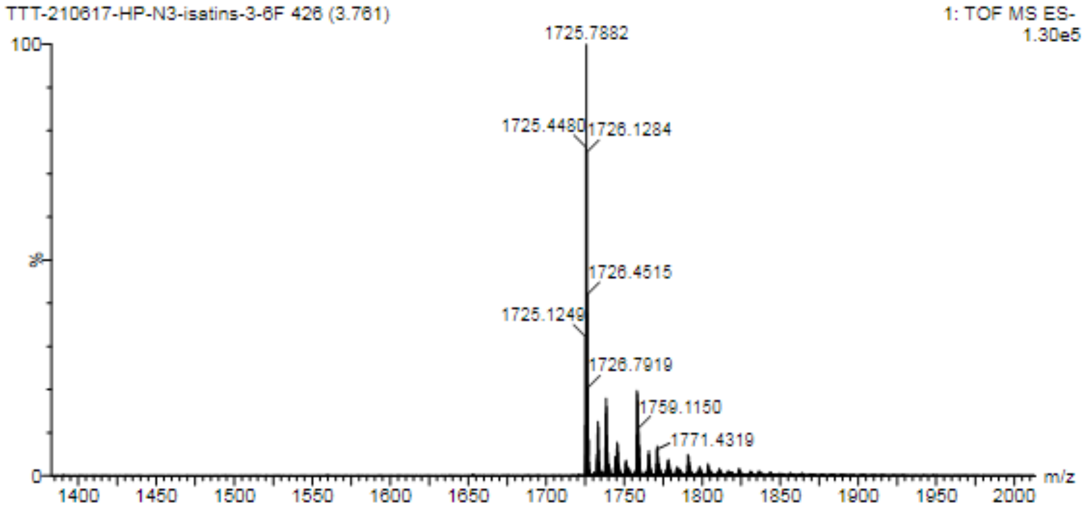

LC Trace and Mass of HO5

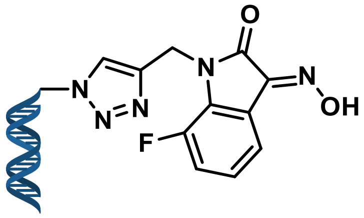

Yield: 81%

Exact mass: 5181.421

Triply charged mass [M]/3 - 1.00794, calculated 1726.1324; observed 1725.7882.

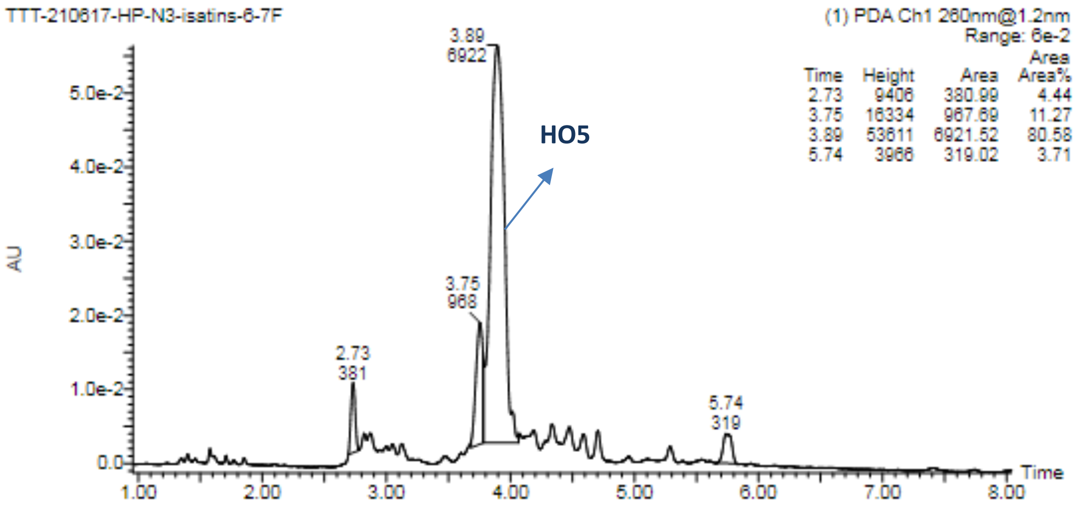

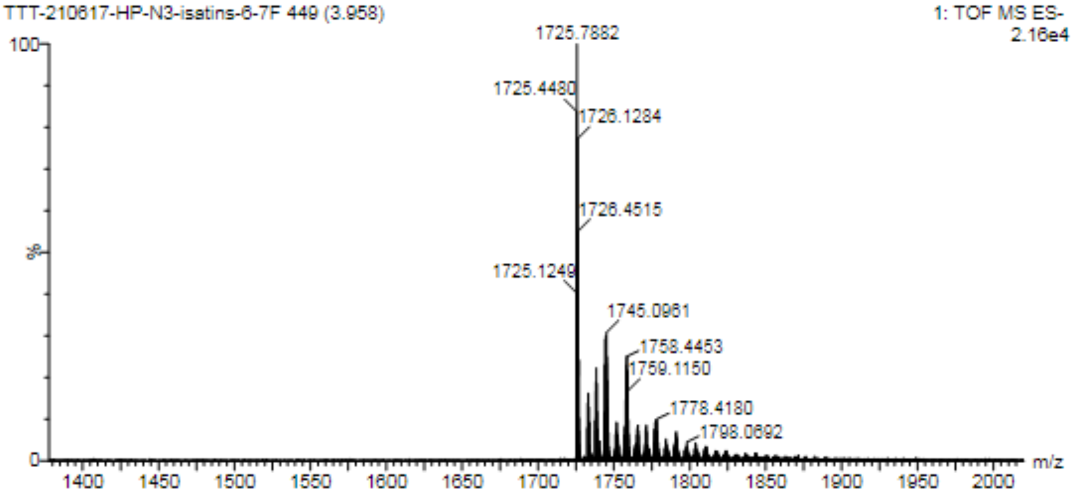

LC Trace and Mass of HO6

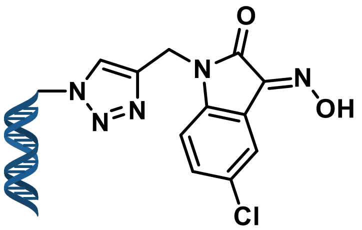

Yield: 94%

Exact mass: 5197.881

Triply charged mass [M]/3 - 1.00794, calculated 1731.6191; observed 1731.4398.

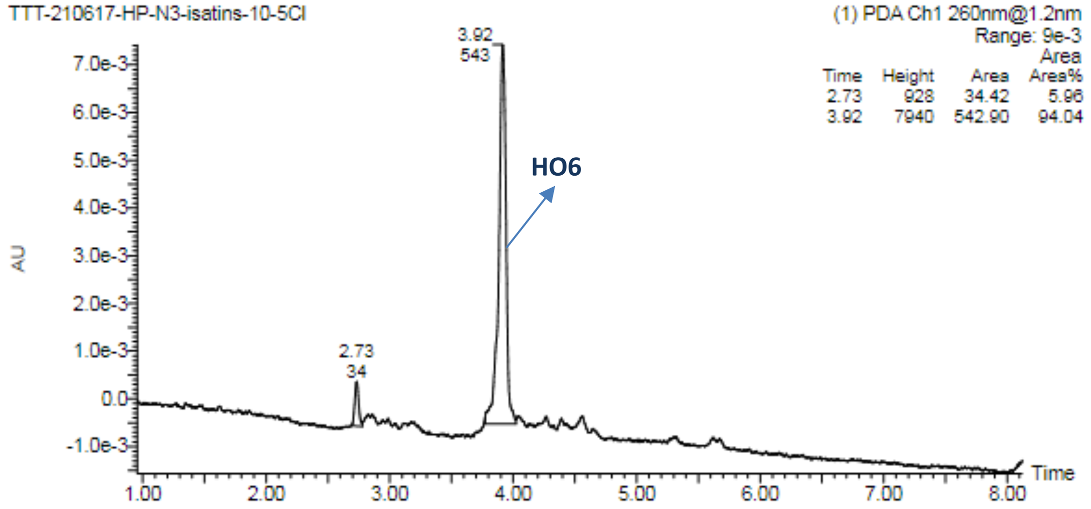

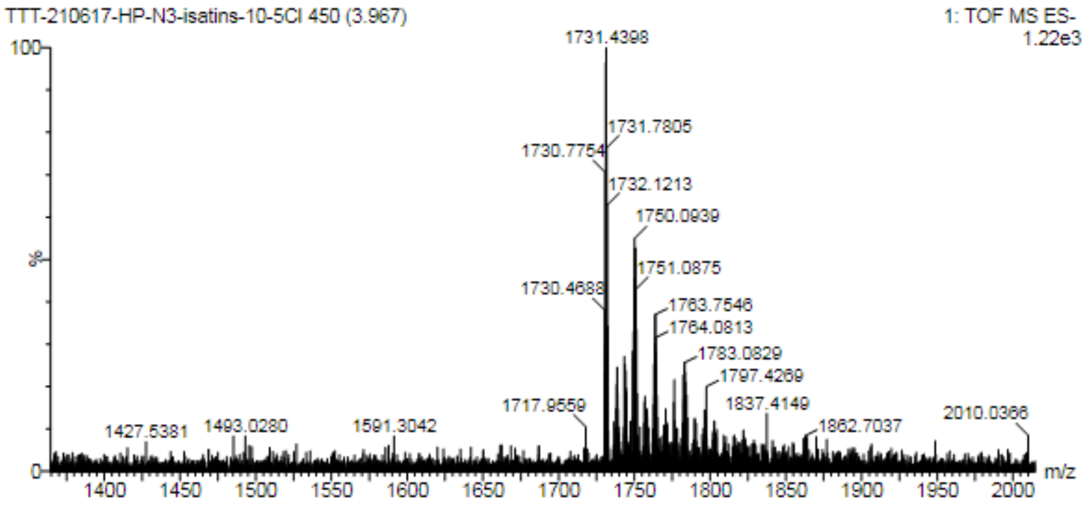

LC Trace and Mass of HO7

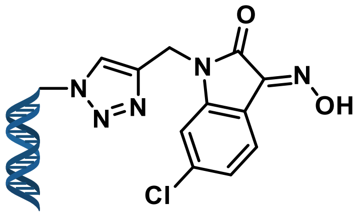

Yield: 80%

Exact mass: 5197.881

Triply charged mass [M]/3 - 1.00794, calculated 1731.6191; observed 1731.4568.

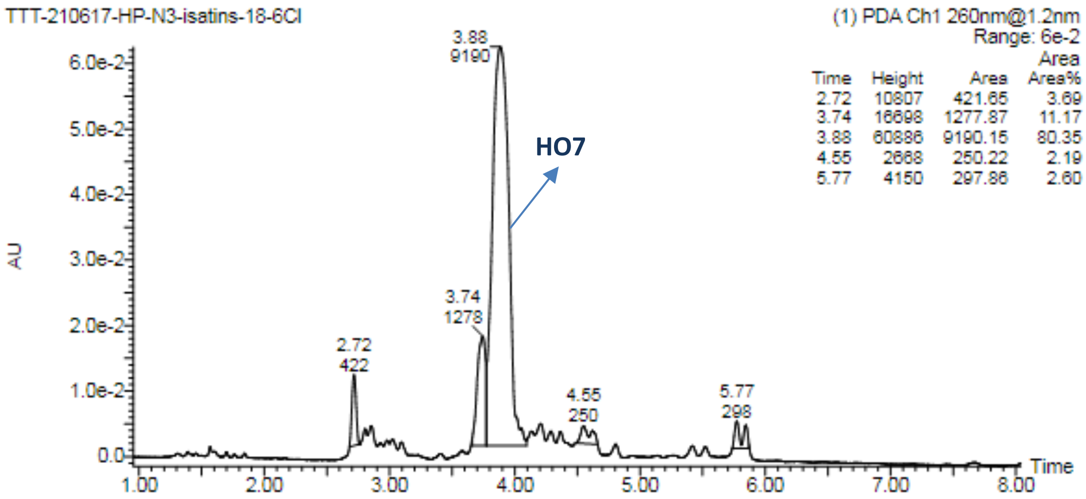

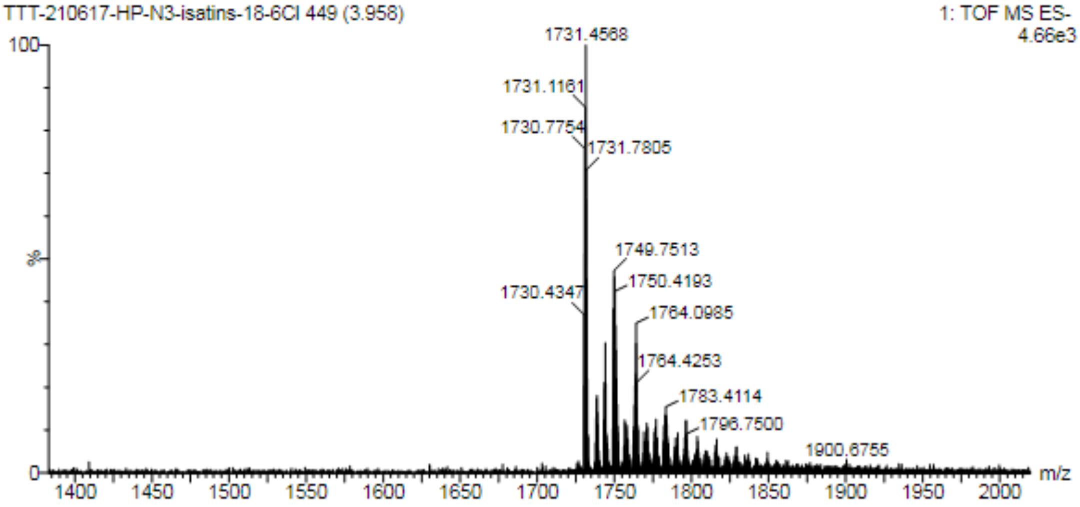

LC Trace and Mass of HO8

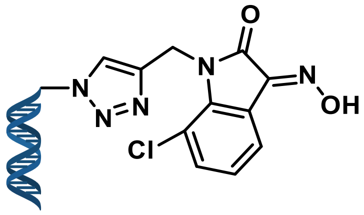

Yield: 80%

Exact mass: 5197.881

Triply charged mass [M]/3 - 1.00794, calculated 1731.6191; observed 1731.0990.

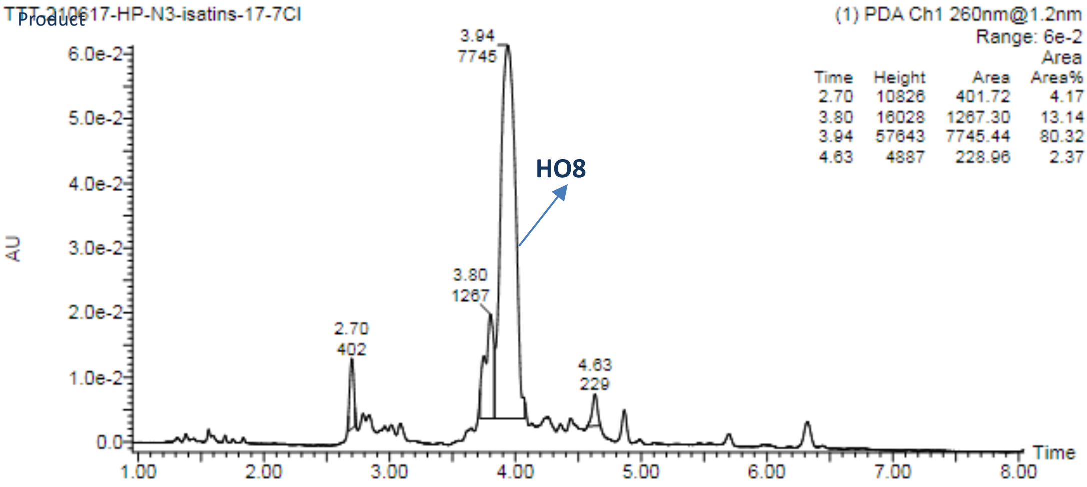

Time	Height	Area	Area%
2.70	10826	401.72	4.17
3.80	16028	1267.30	13.14
3.94	57643	7745.44	80.32
4.63	4887	228.96	2.37

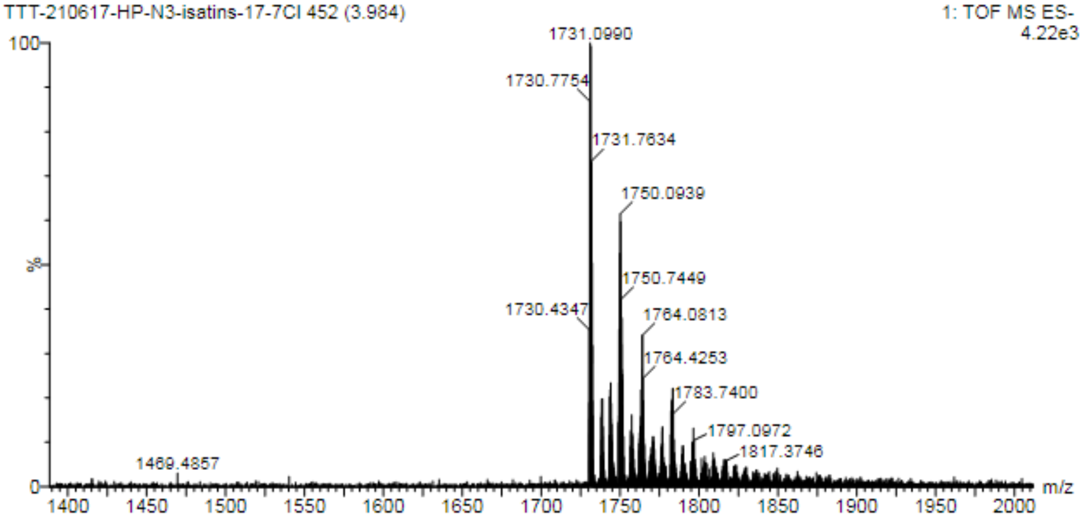

LC Trace and Mass of HO9

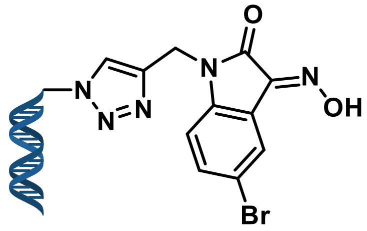

Yield: 85%

Exact mass: 5242.331

Triply charged mass [M]/3 - 1.00794, calculated 1746.4357; observed 1746.0881.

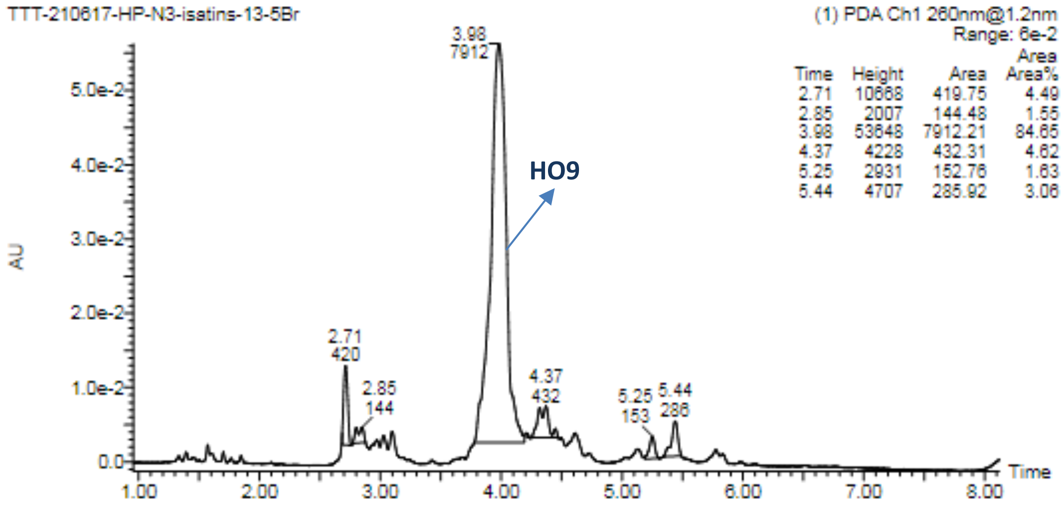

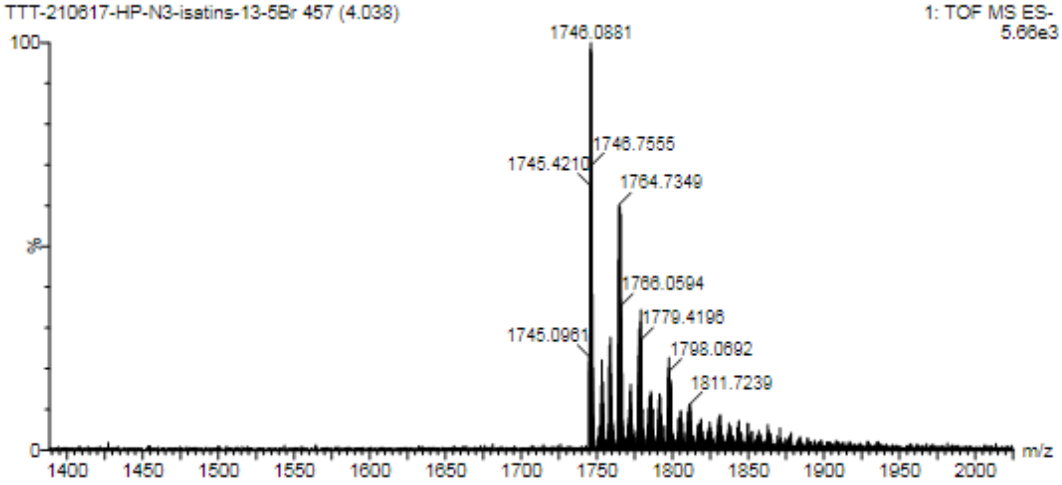

LC Trace and Mass of HO10

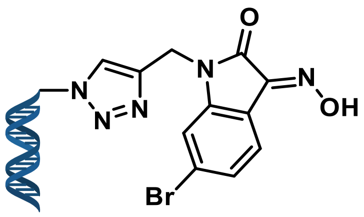

Yield: 82%

Exact mass: 5242.331

Triply charged mass [M]/3 - 1.00794, calculated 1746.4357; observed 1746.0881.

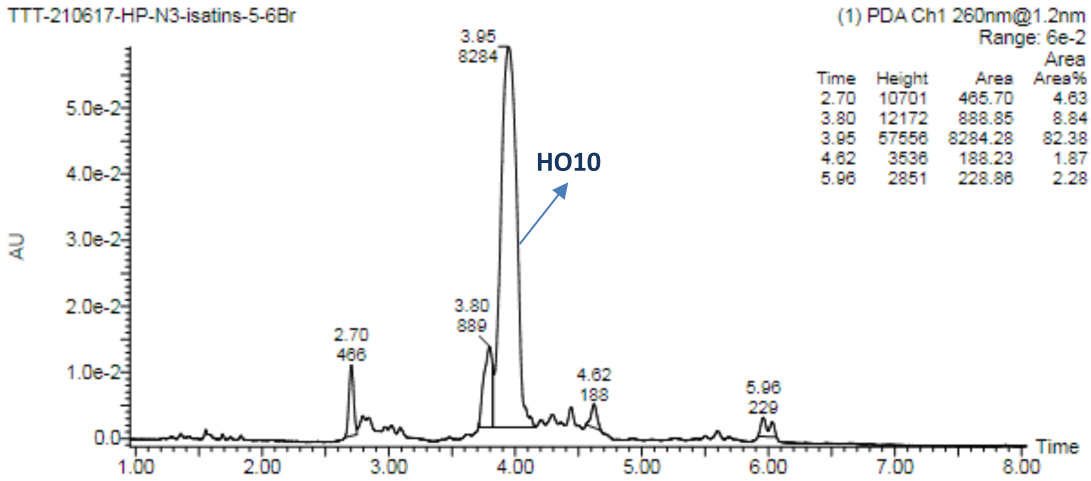

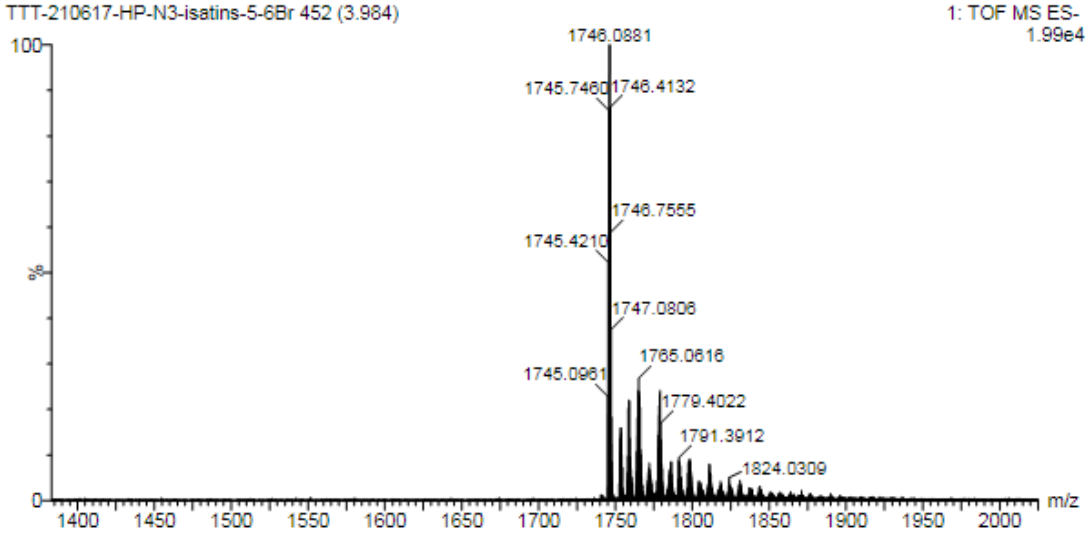

LC Trace and Mass of HO11

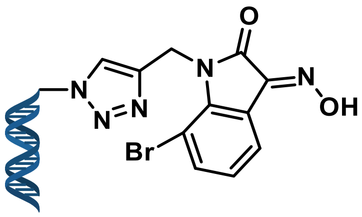

Yield: 81%

Exact mass: 5242.331

Triply charged mass [M]/3 - 1.00794, calculated 1746.4357; observed 1746.0881.

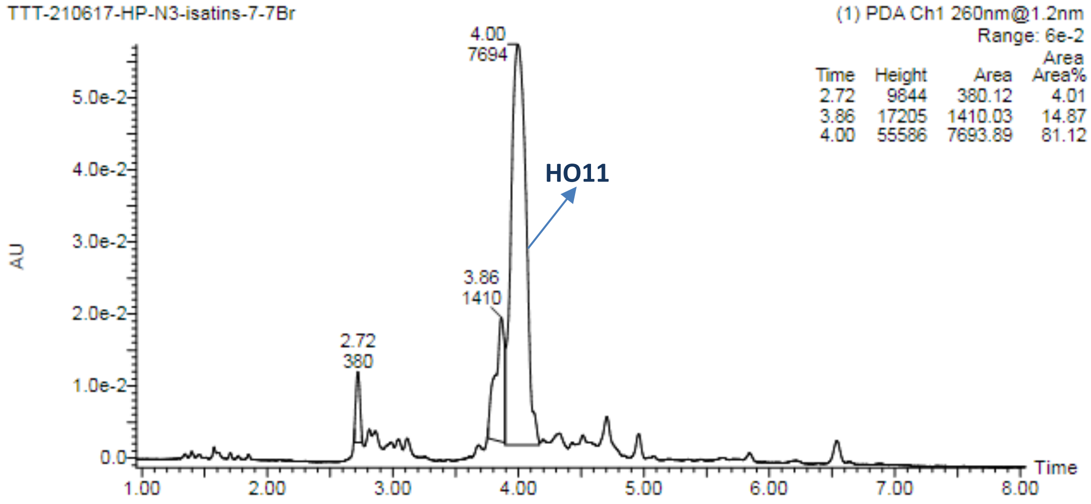

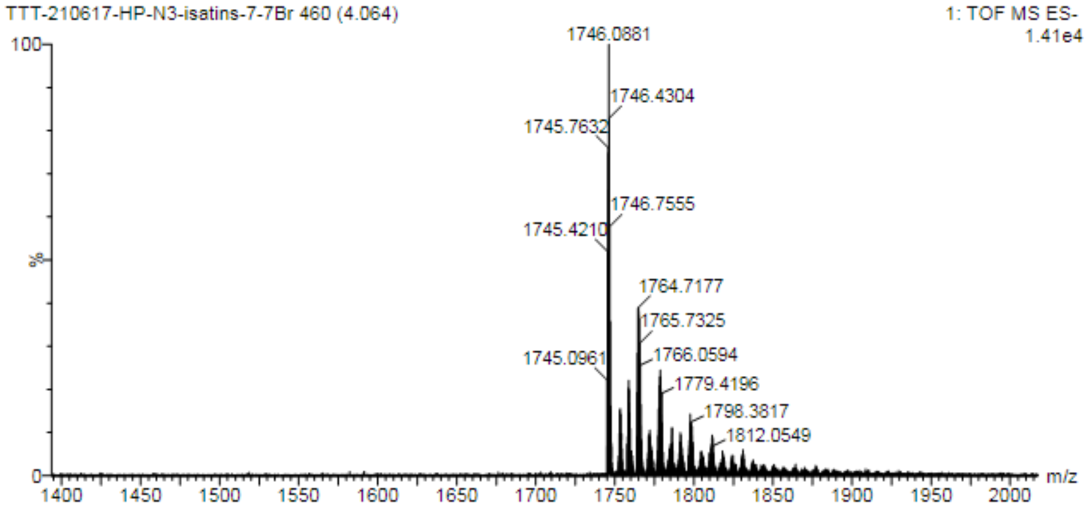

LC Trace and Mass of HO12

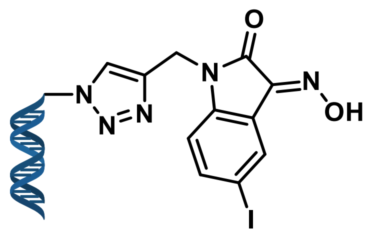

Yield: 81%

Exact mass: 5289.331

Triply charged mass [M]/3 - 1.00794, calculated 1762.1024; observed 1761.7434.

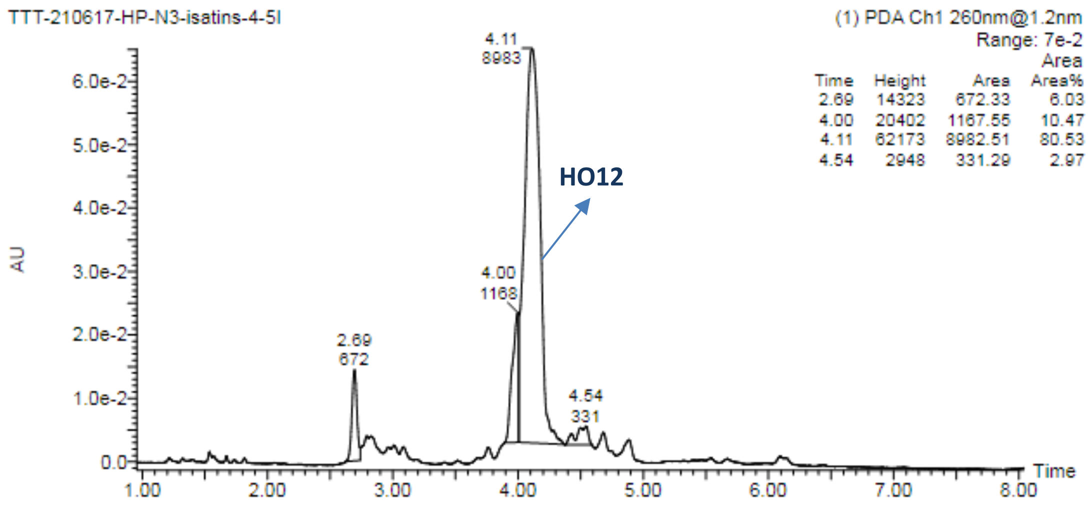

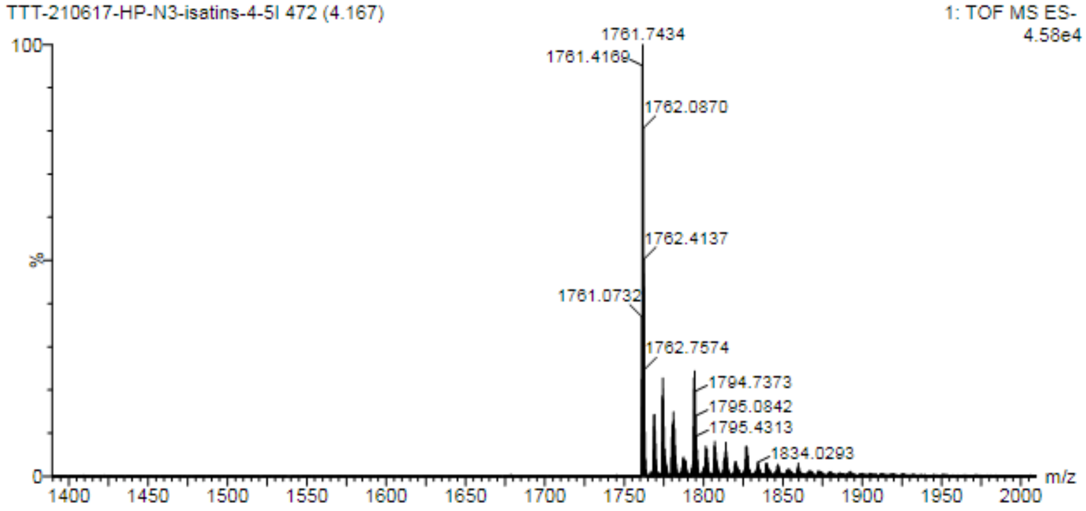

LC Trace and Mass of HO13

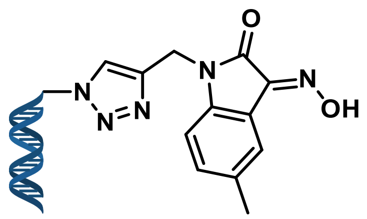

Yield: 89%

Exact mass: 5177.461

Triply charged mass [M]/3 - 1.00794, calculated 1724.8124; observed 1724.4617.

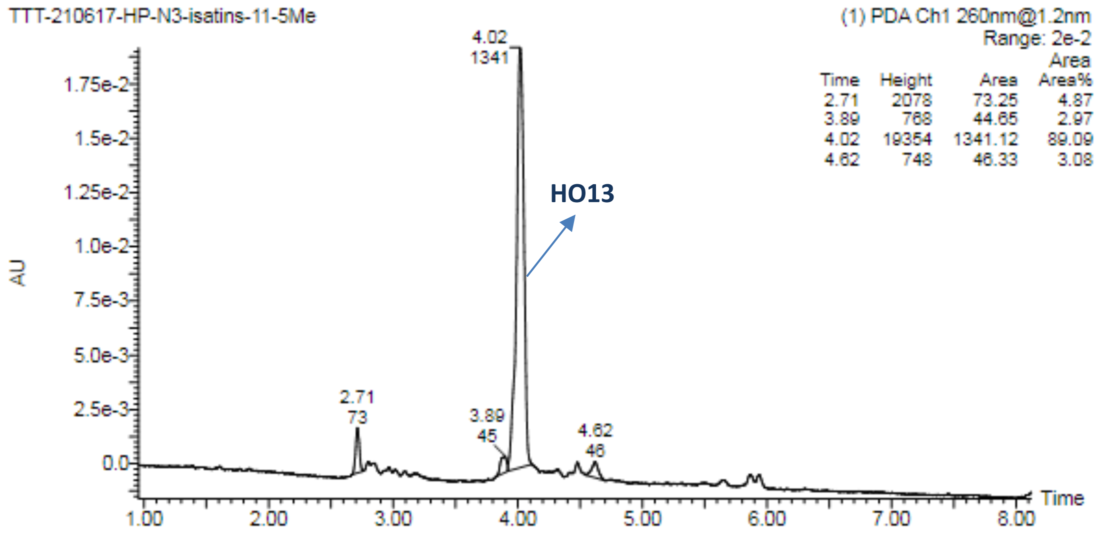

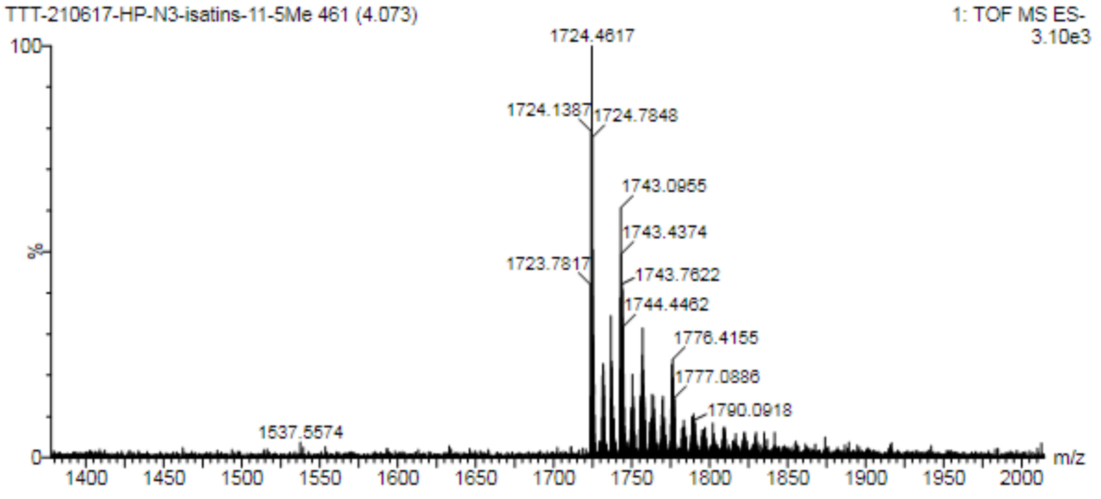

LC Trace and Mass of HO14

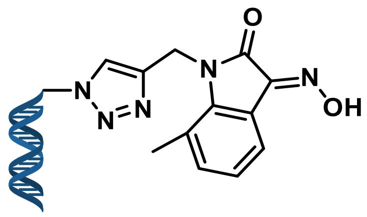

Yield: 85%

Exact mass: 5177.461

Triply charged mass [M]/3 - 1.00794, calculated 1724.8124; observed 1724.4617.

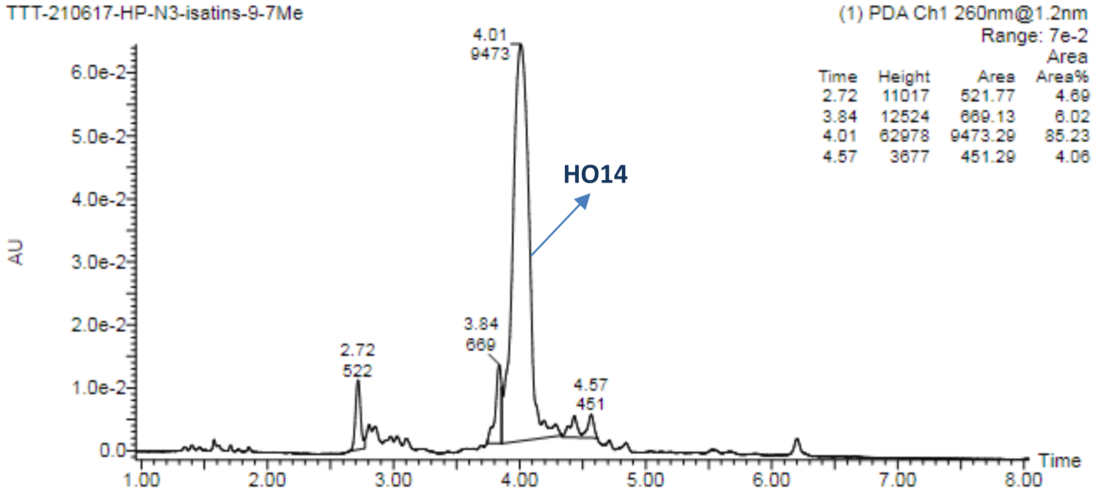

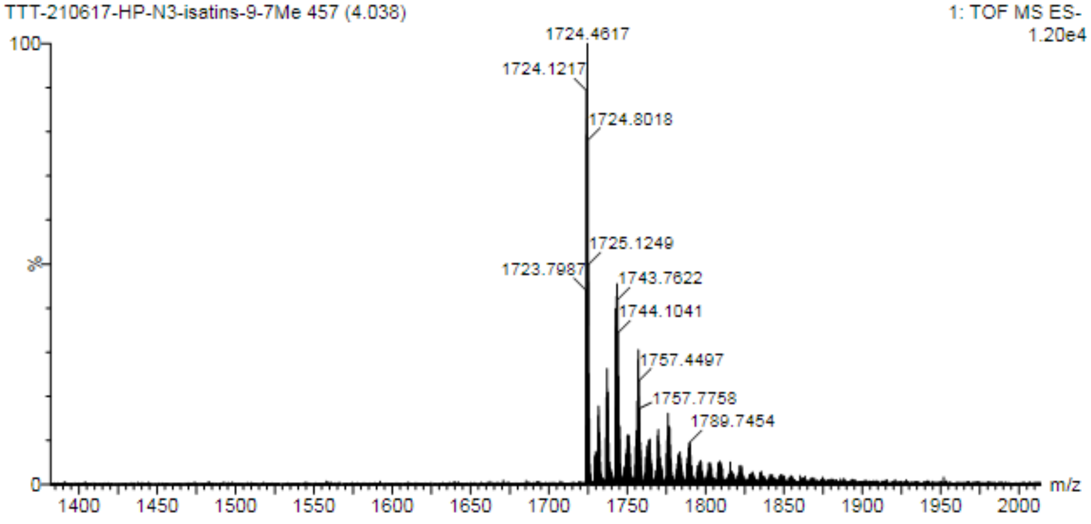

LC Trace and Mass of HO15

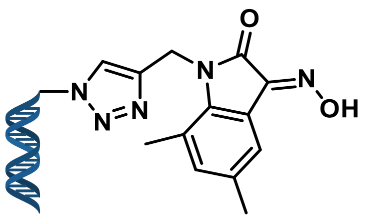

Yield: 86%

Exact mass: 5191.491

Triply charged mass [M]/3 - 1.00794, calculated 1729.4891; observed 1729.1405.

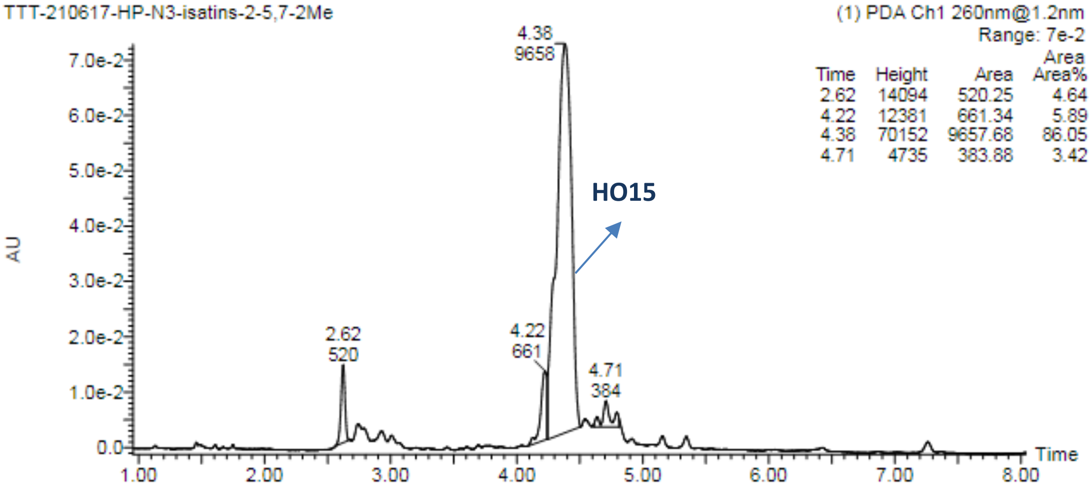

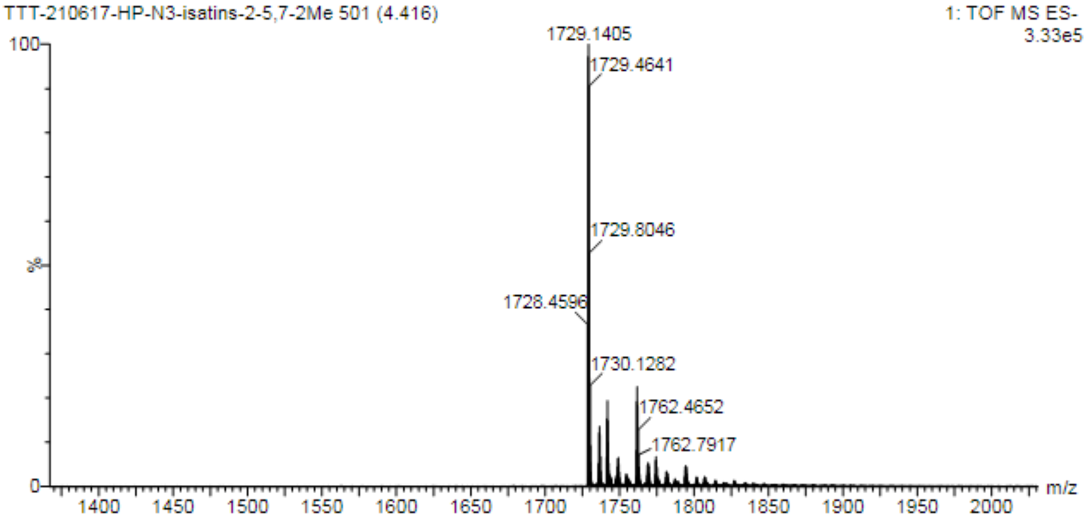

LC Trace and Mass of HO16

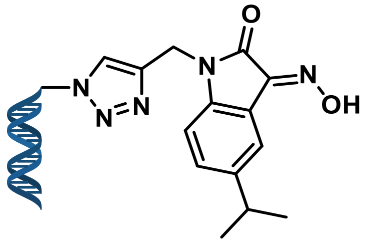

Yield: 87%

Exact mass: 5205.511

Triply charged mass [M]/3 - 1.00794, calculated 1734.1624; observed 1733.8087.

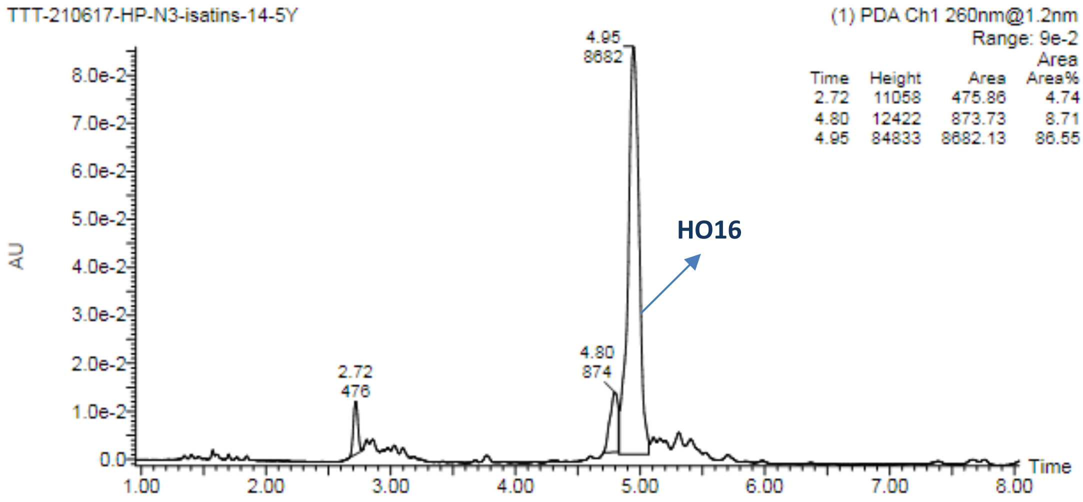

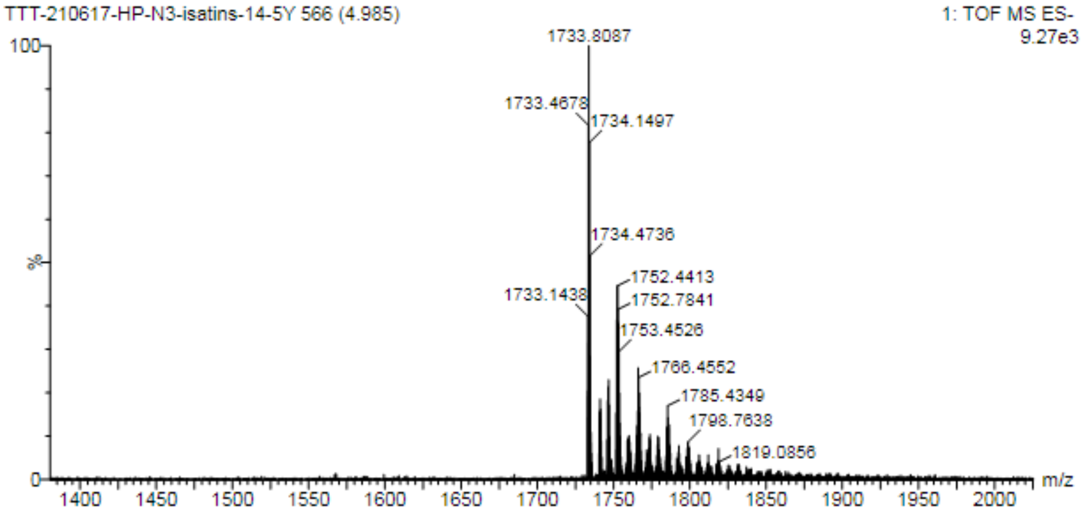

LC Trace and Mass of HO17

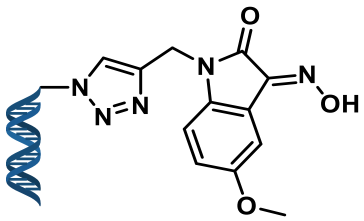

Yield: 88%

Exact mass: 5193.461

Triply charged mass [M]/3 - 1.00794, calculated 1730.1457; observed 1729.8046.

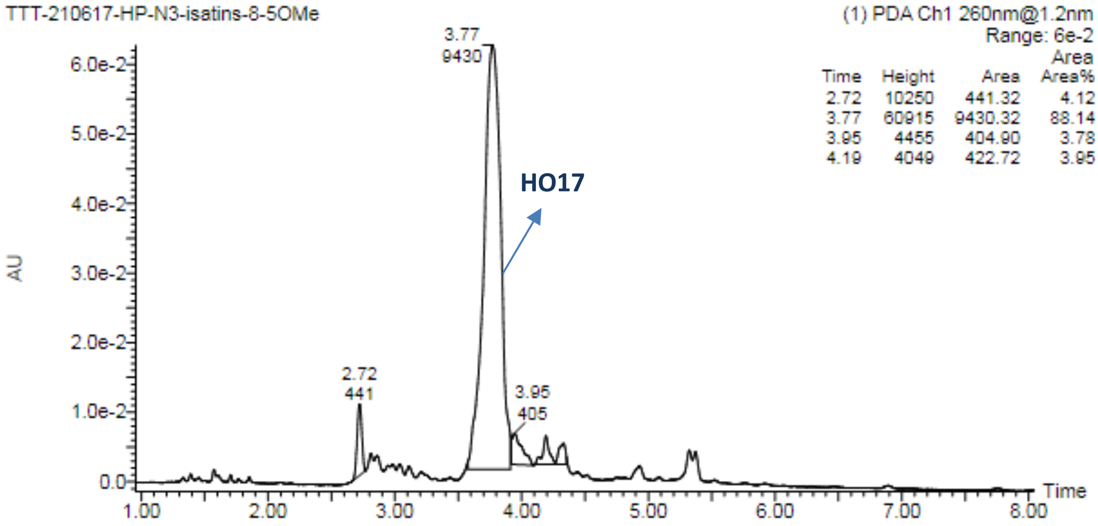

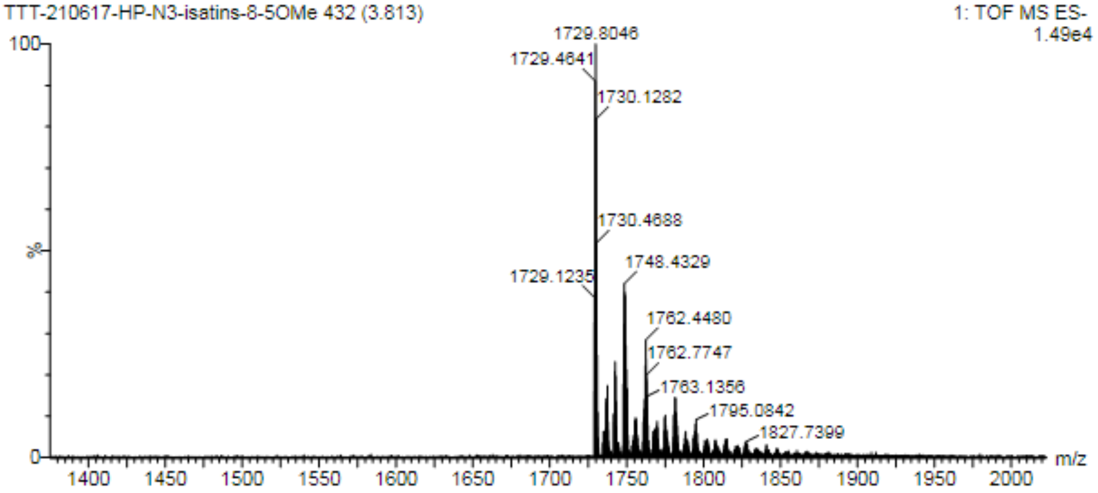

LC Trace and Mass of HO18

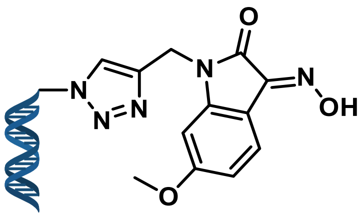

Yield: 82%

Exact mass: 5193.461

Triply charged mass [M]/3 - 1.00794, calculated 1730.1457; observed 1729.4641.

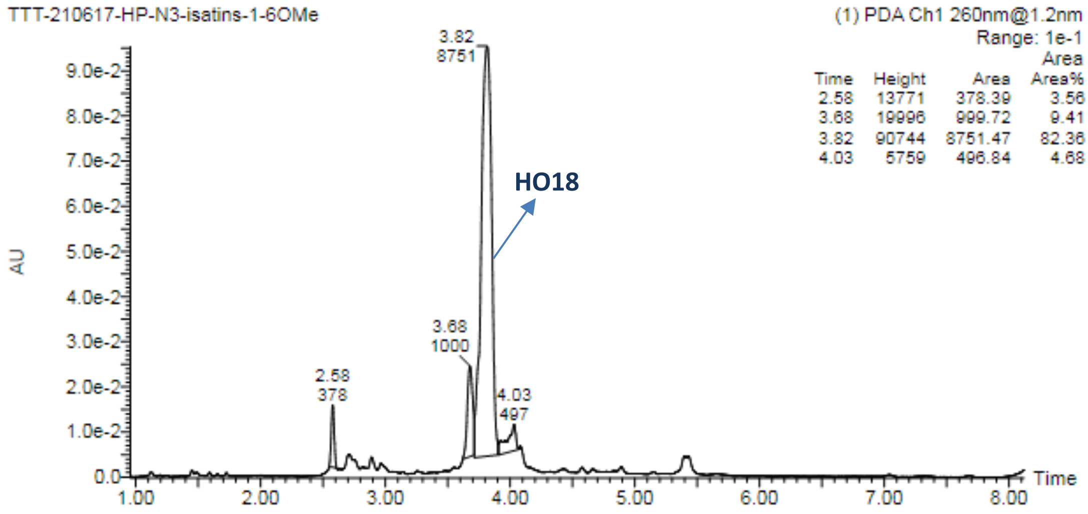

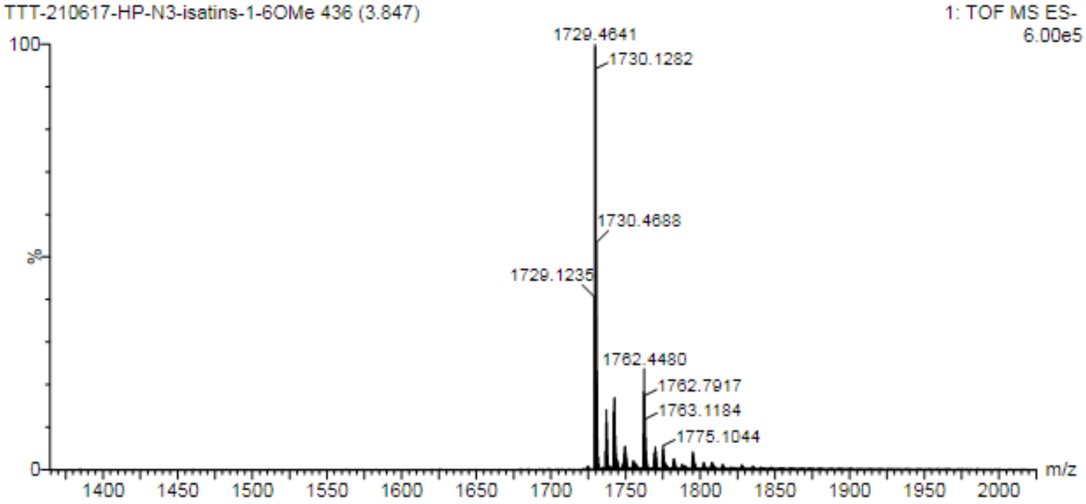

LC Trace and Mass of HO19

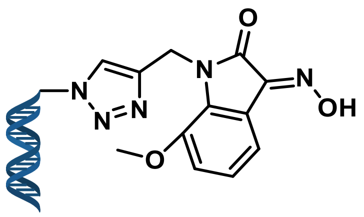

Yield: 87%

Exact mass: 5193.461

Triply charged mass [M]/3 - 1.00794, calculated 1730.1457; observed 1729.8046.

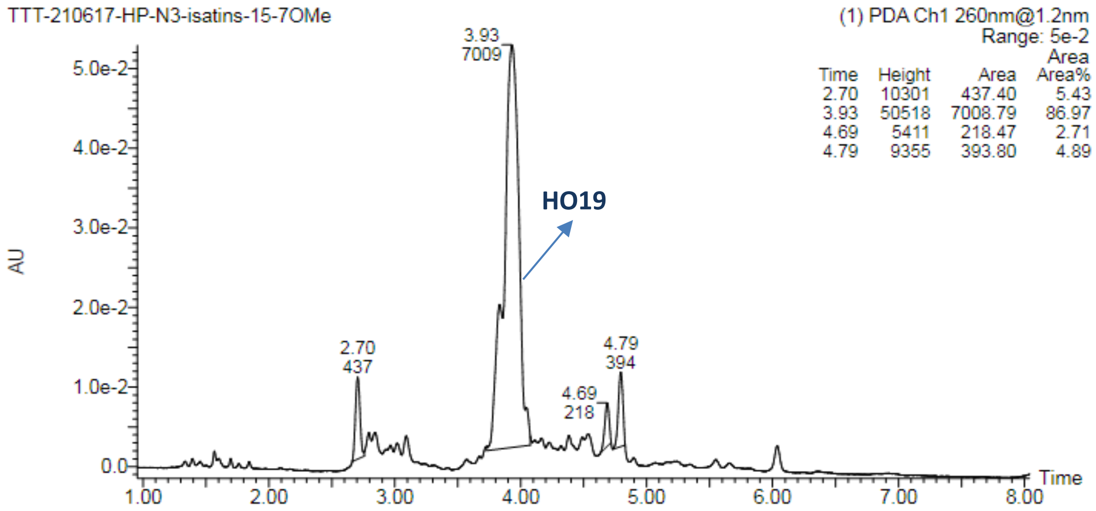

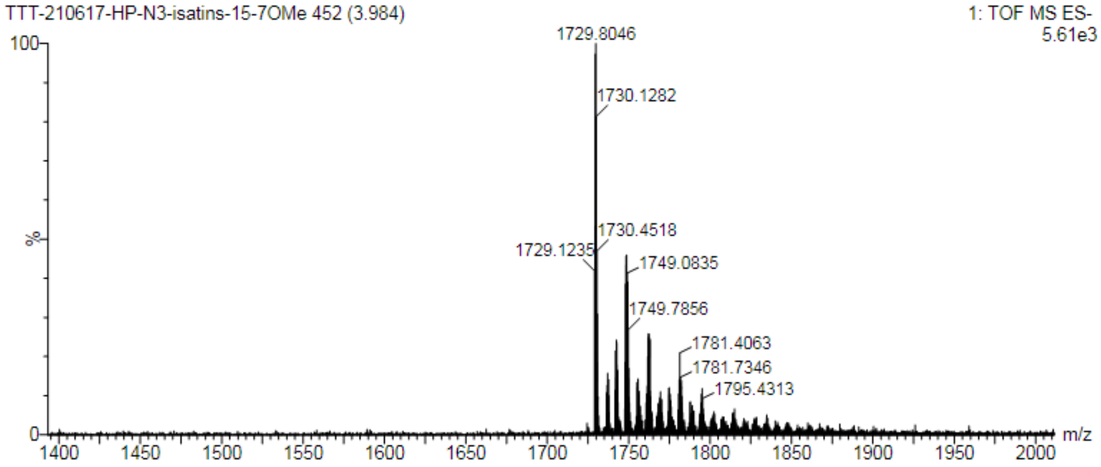

LC Trace and Mass of HO20

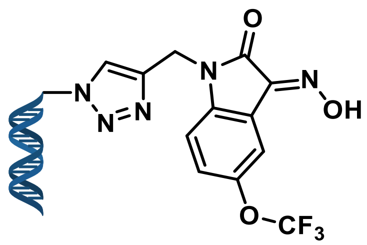

Yield: 88%

Exact mass: 5247.421

Triply charged mass [M]/3 - 1.00794, calculated 1748.1324; observed 1747.9706.

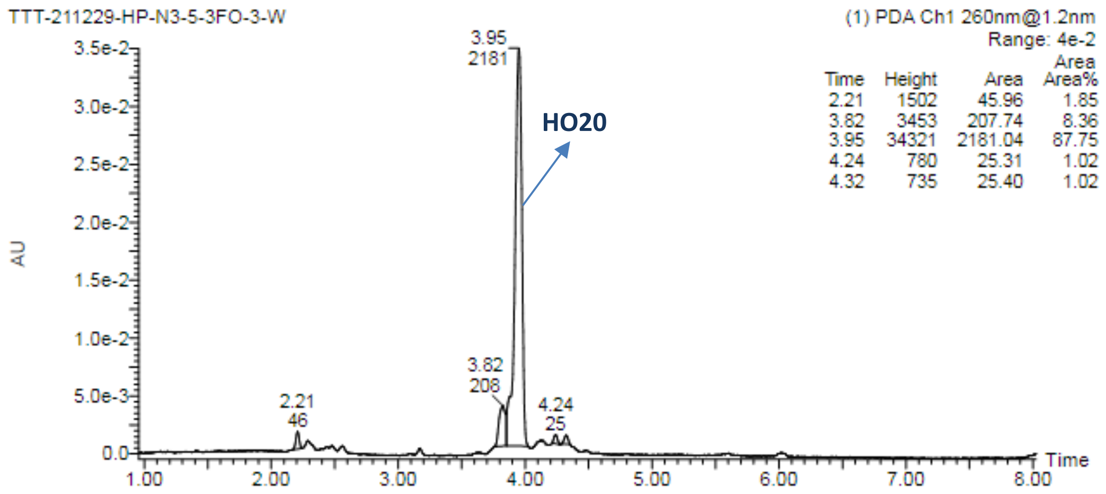

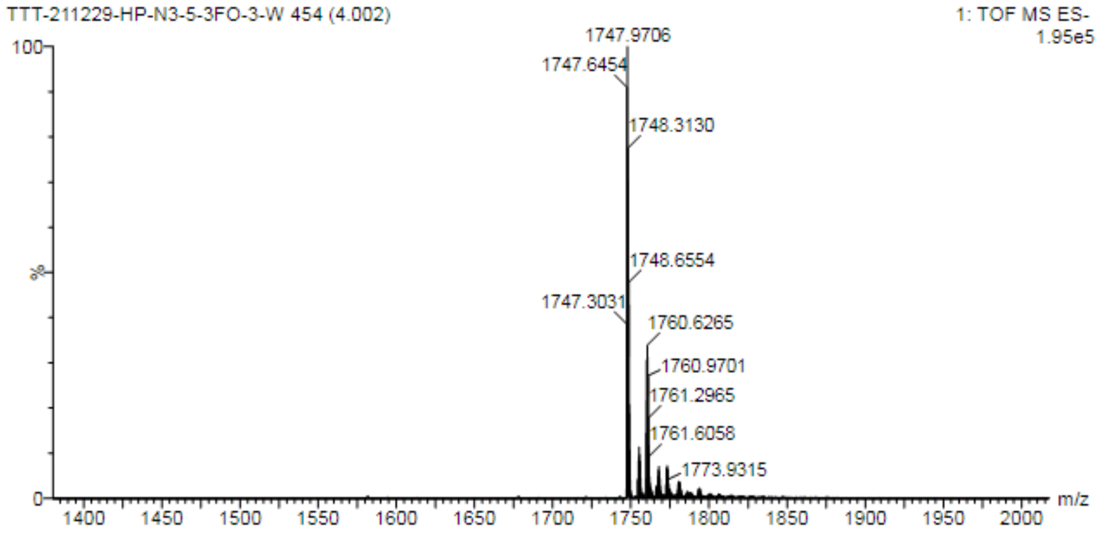

LC Trace and Mass of O1a

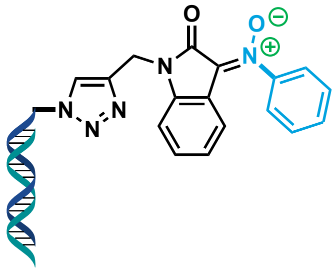

Following General Procedure vi

Yield: 86%

Exact mass: 5239.53

Triply charged mass [M]/3 - 1.00794, calculated 1745.5103; observed 1745.3696.

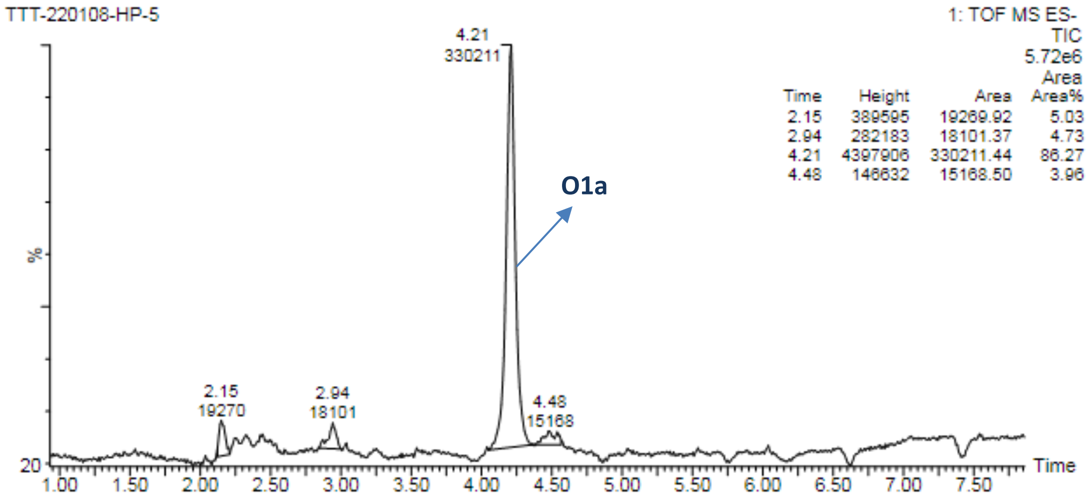

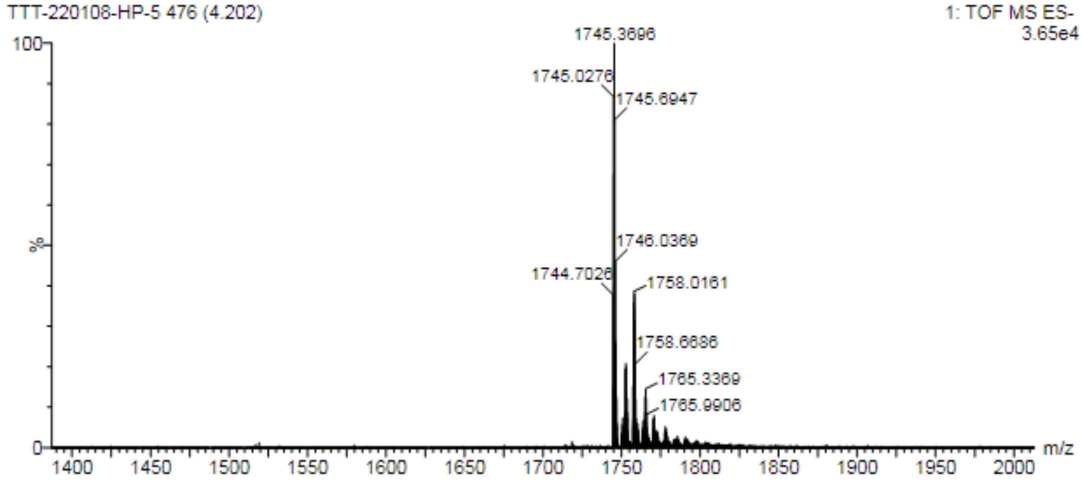

LC Trace and Mass of O1b

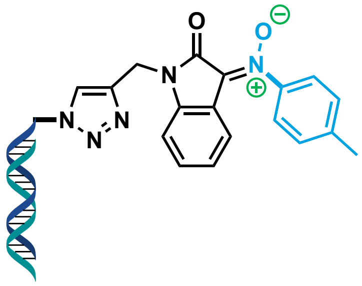

Following General Procedure vi

Yield: 68%

Exact mass: 5253.53

Triply charged mass [M]/3 - 1.00794, calculated 1750.1691; observed 1750.1022.

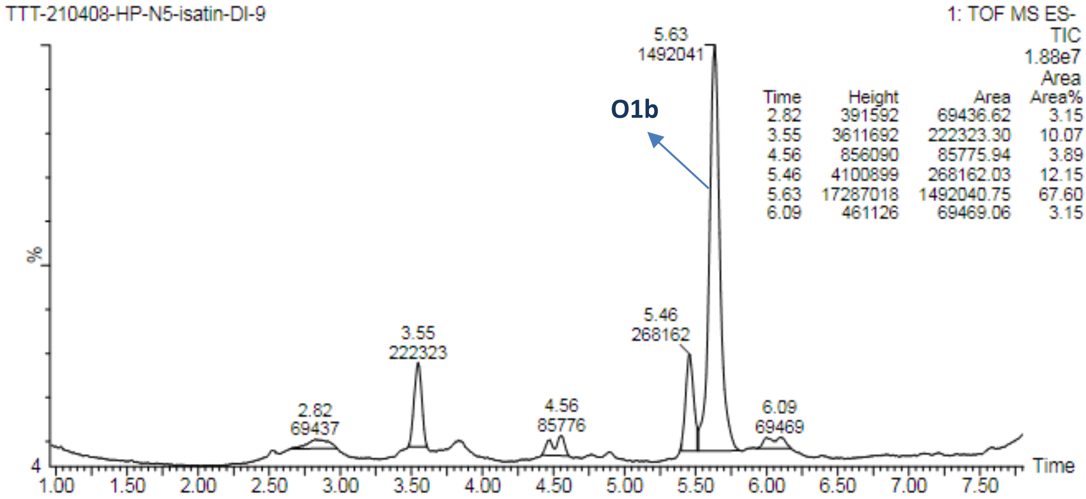

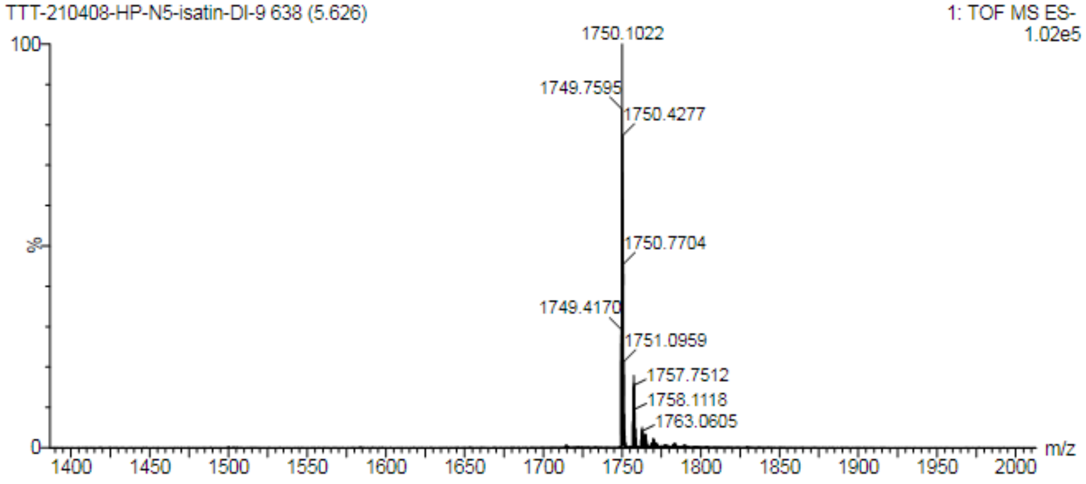

LC Trace and Mass of O1c

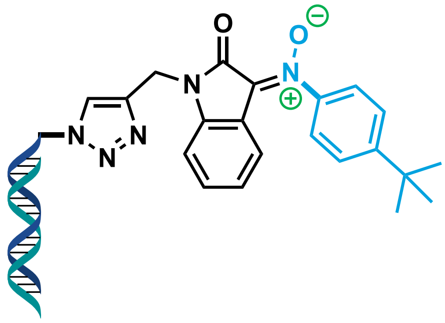

Following General Procedure vi

Yield: 85%

Exact mass: 5195.63

Triply charged mass [M]/3 - 1.00794, calculated 1764.2024; observed 1764.1095.

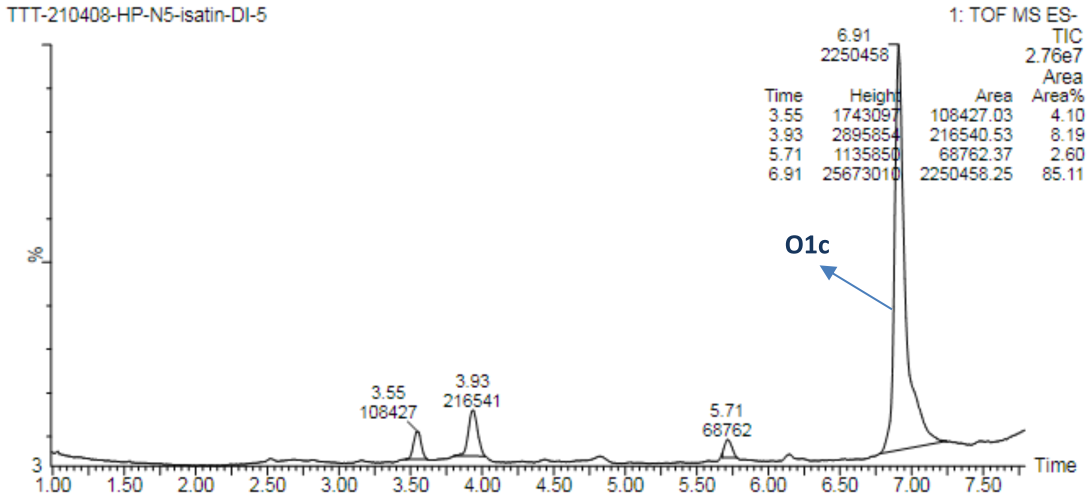

Time	Height	Area	Area%
3.55	1743097	108427.03	4.10
3.93	2895854	216540.53	8.19
5.71	1135850	68762.37	2.60
6.91	25673010	2250458.25	85.11

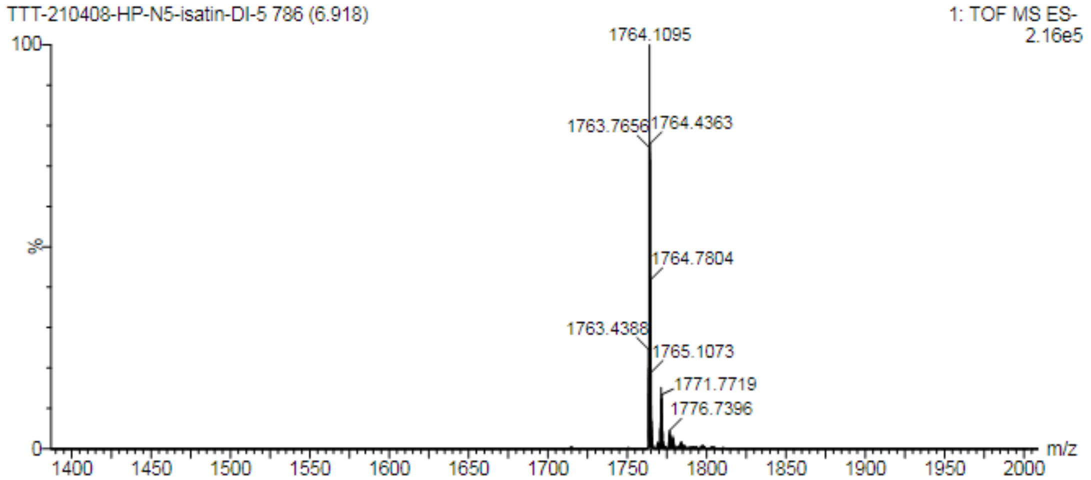

LC Trace and Mass of O1d

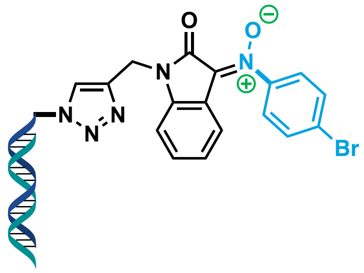

Following General Procedure vi

Yield: 83%

Exact mass: 5318.43

Triply charged mass [M]/3 - 1.00794, calculated 1771.8024; observed 1771.7201.

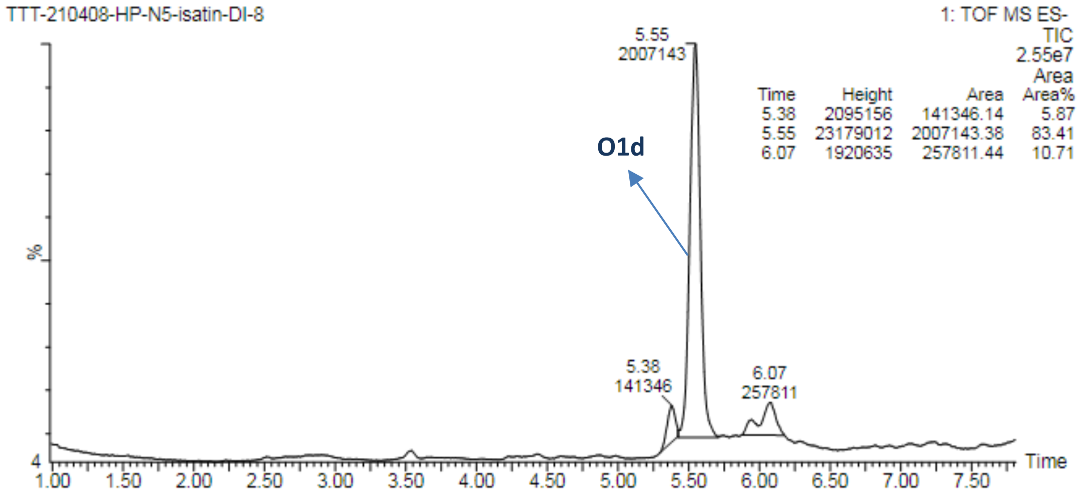

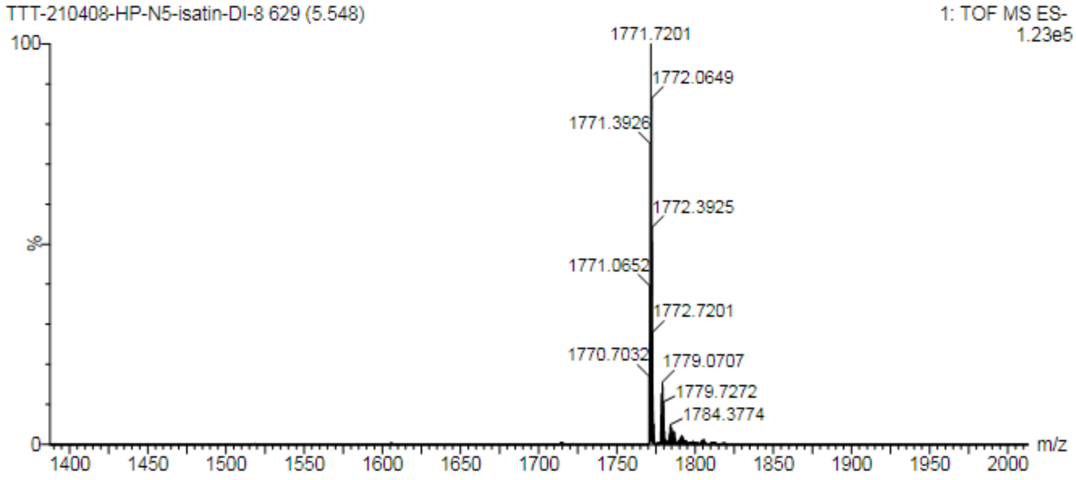

LC Trace and Mass of O1e

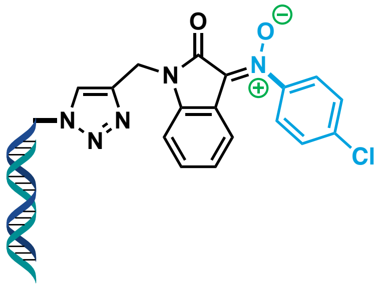

Following General Procedure vi

Yield: 84%

Exact mass: 5273.98

Triply charged mass [M]/3 - 1.00794, calculated 1756.9857; observed 1757.0817.

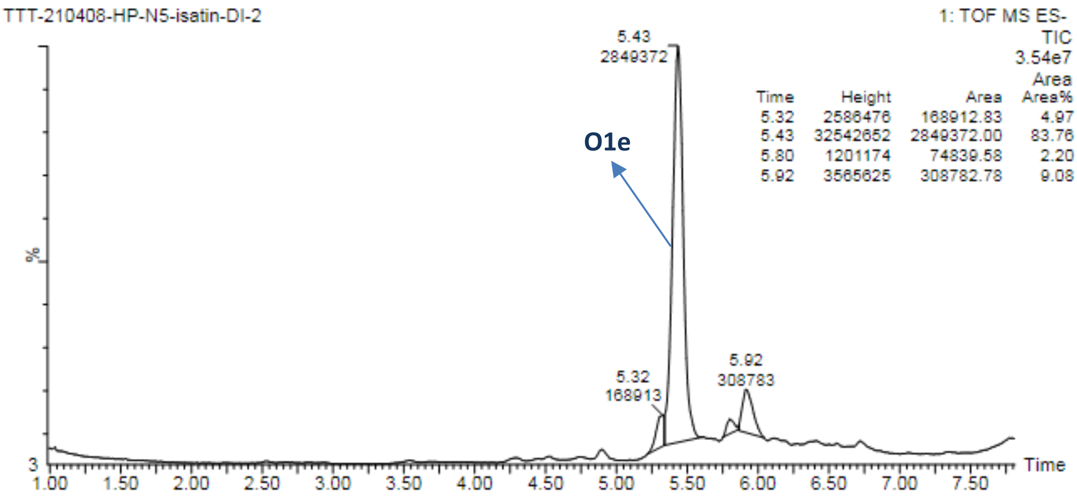

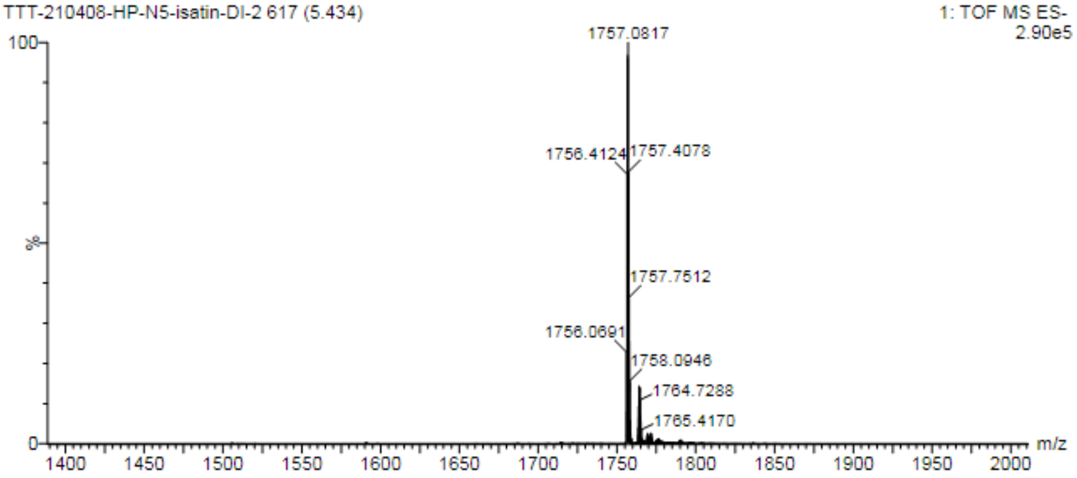

LC Trace and Mass of O1f

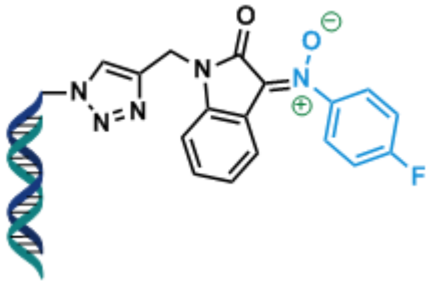

Following General Procedure vi

Yield: 95%

Exact mass: 5257.53

Triply charged mass [M]/3 - 1.00794, calculated 1751.5024; observed 1751.4216.

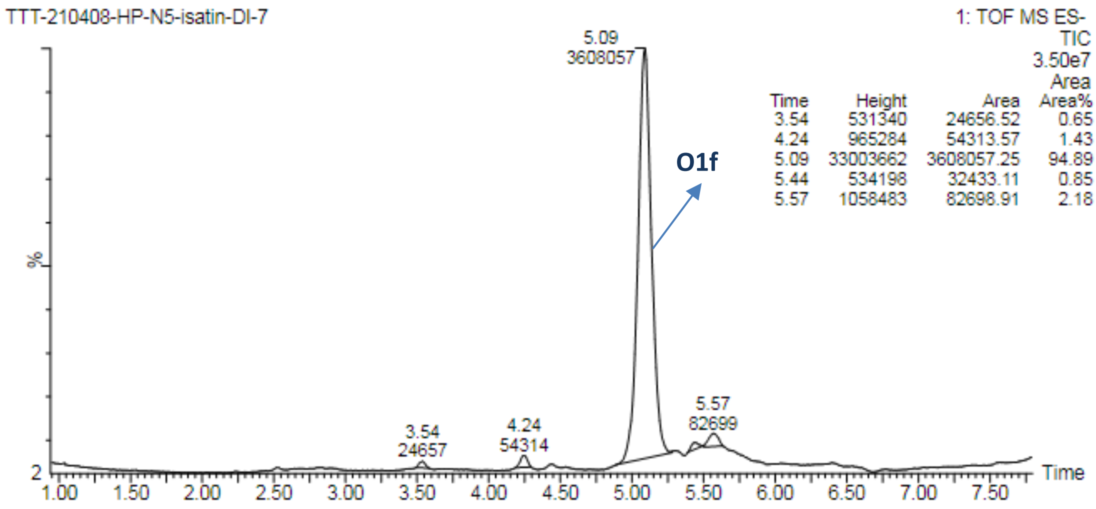

Time	Height	Area	Area%
3.54	531340	24656.52	0.65
4.24	965284	54313.57	1.43
5.09	33003662	3608057.25	94.89
5.44	534198	32433.11	0.85
5.57	1058483	82698.91	2.18

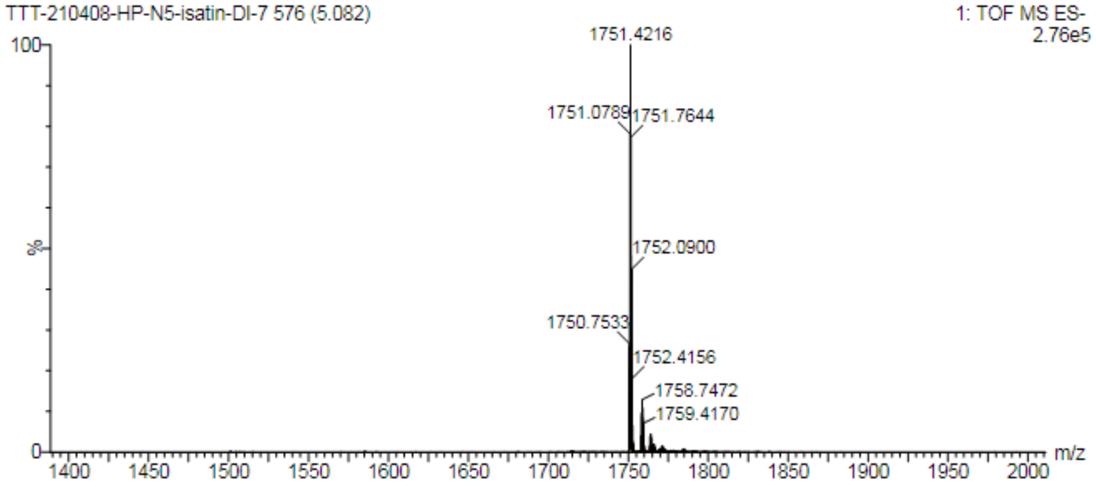

LC Trace and Mass of O1g

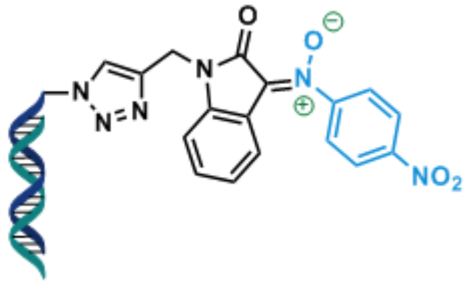

Following General Procedure vi

Yield: 77%

Exact mass: 5284.53

Triply charged mass [M]/3 - 1.00794, calculated 1760.5024; observed 1760.4135.

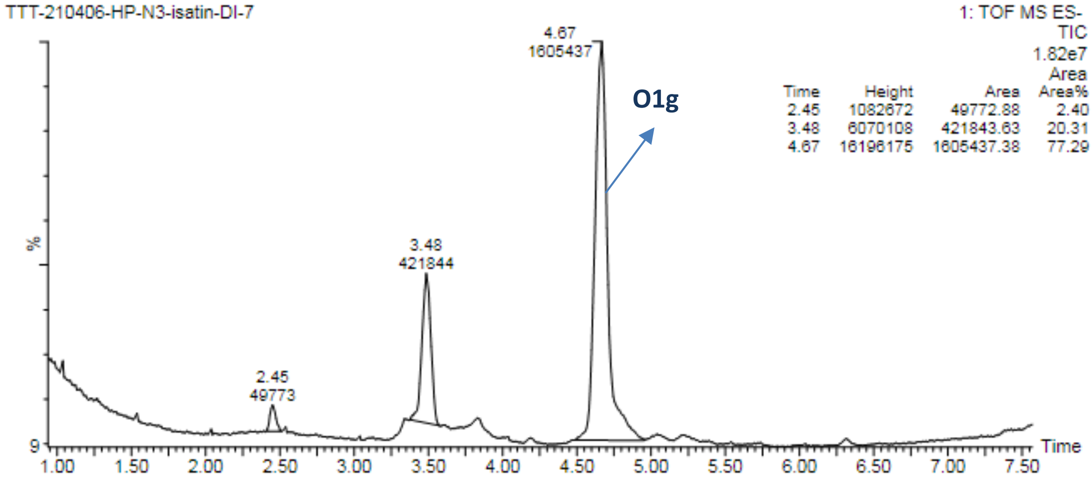

Time	Height	Area	Area%
2.45	1082672	49772.88	2.40
3.48	6070108	421843.63	20.31
4.67	16196175	1605437.38	77.29

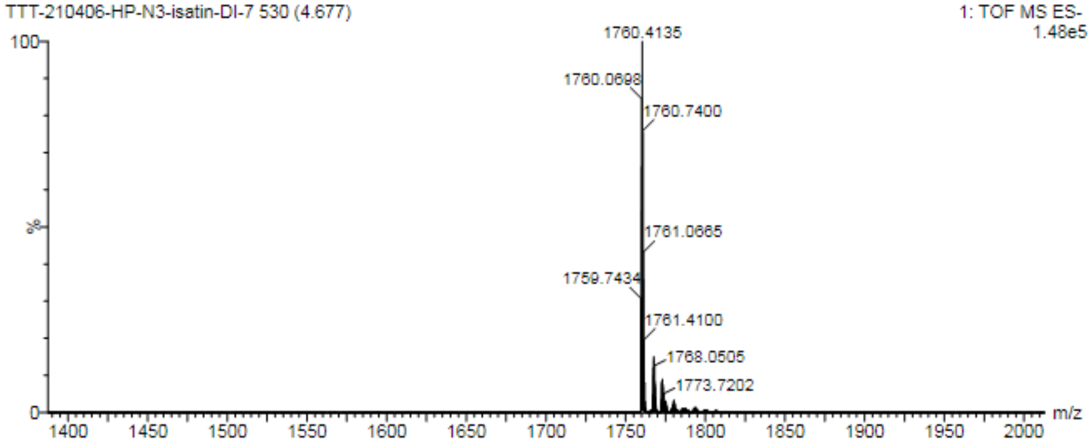

LC Trace and Mass of O1h

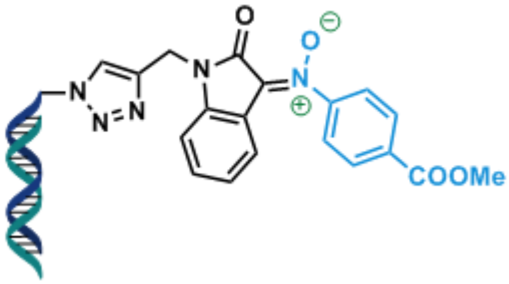

Following General Procedure vi

Yield: 93%

Exact mass: 5297.58

Triply charged mass [M]/3 - 1.00794, calculated 1764.8524; observed 1764.7460.

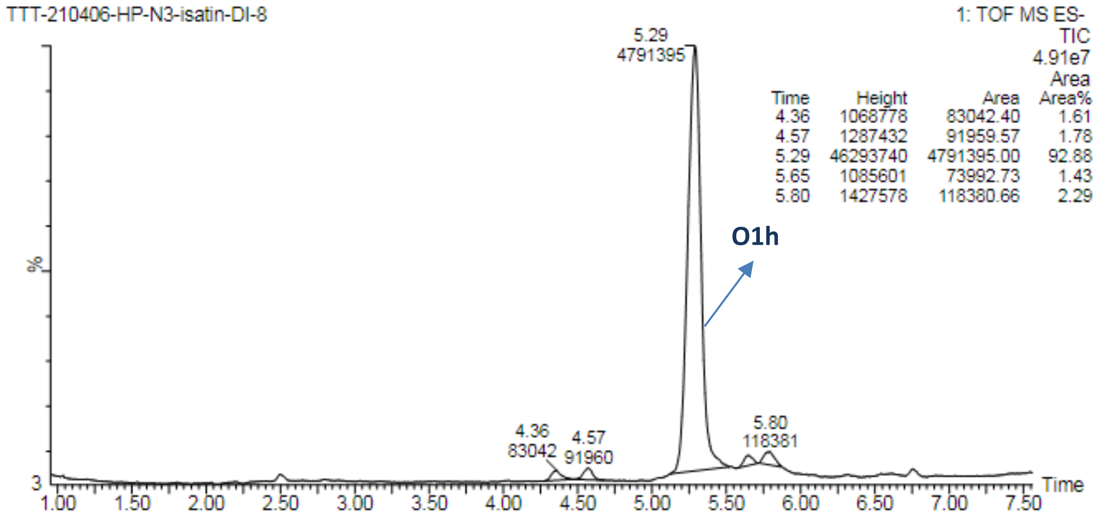

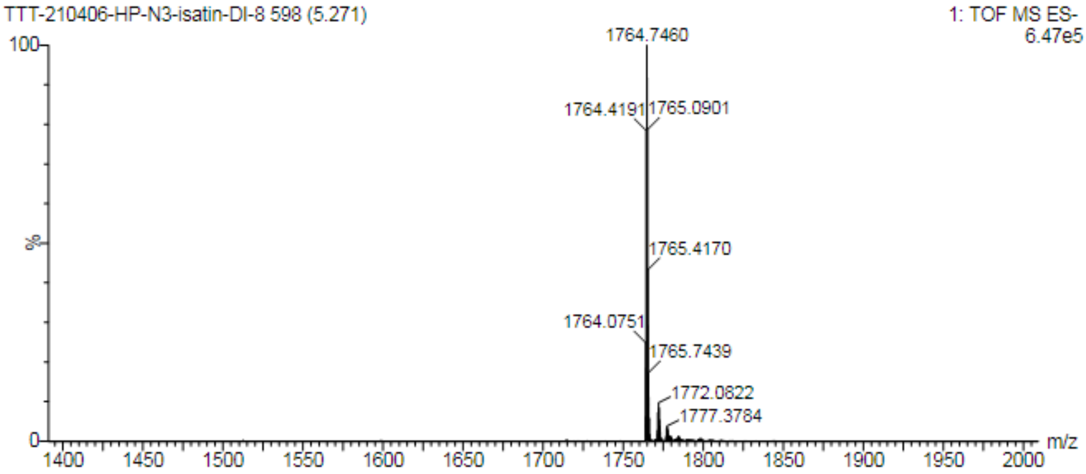

LC Trace and Mass of O1i

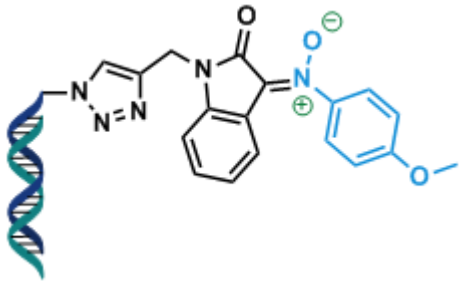

Following General Procedure vi
Yield: 32%
Exact mass: 5269.58
Triply charged mass [M]/3 - 1.00794, calculated 1755.5191; observed 1755.4170.

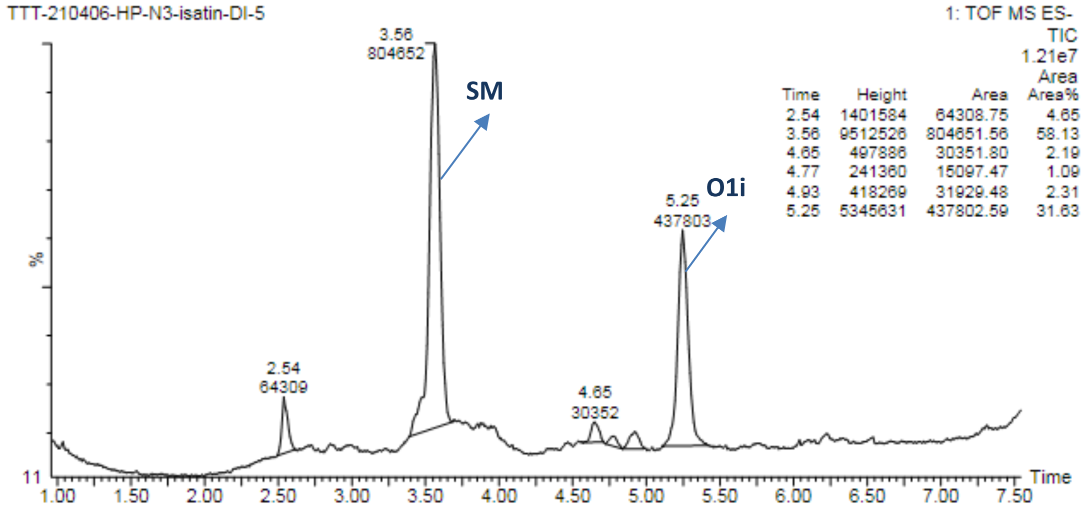

Time	Height	Area	Area%
2.54	1401584	64308.75	4.65
3.56	9512526	804651.56	58.13
4.65	497886	30351.80	2.19
4.77	241360	15097.47	1.09
4.93	418269	31929.48	2.31
5.25	5345631	437802.59	31.63

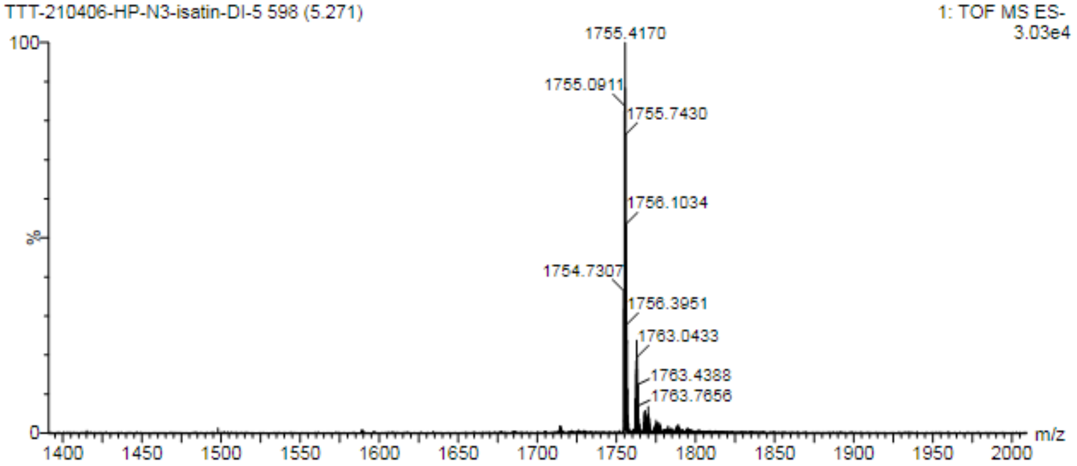

LC Trace and Mass of O1j

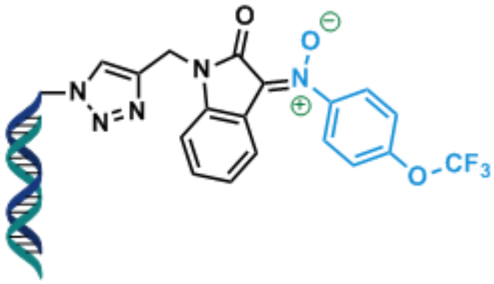

Following General Procedure vi

Yield: 70%

Exact mass: 5323.53

Triply charged mass [M]/3 - 1.00794, calculated 1773.5024; observed 1773.3452.

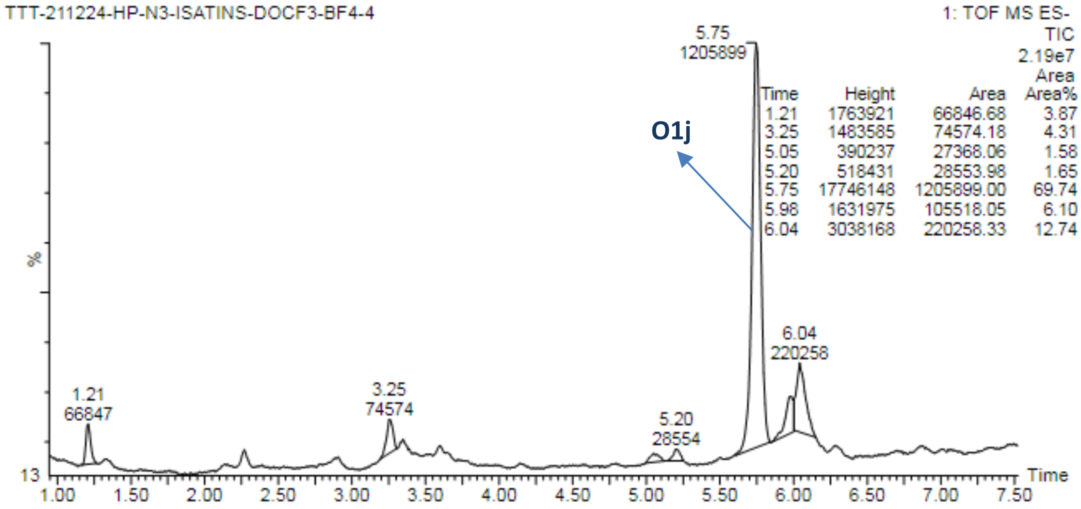

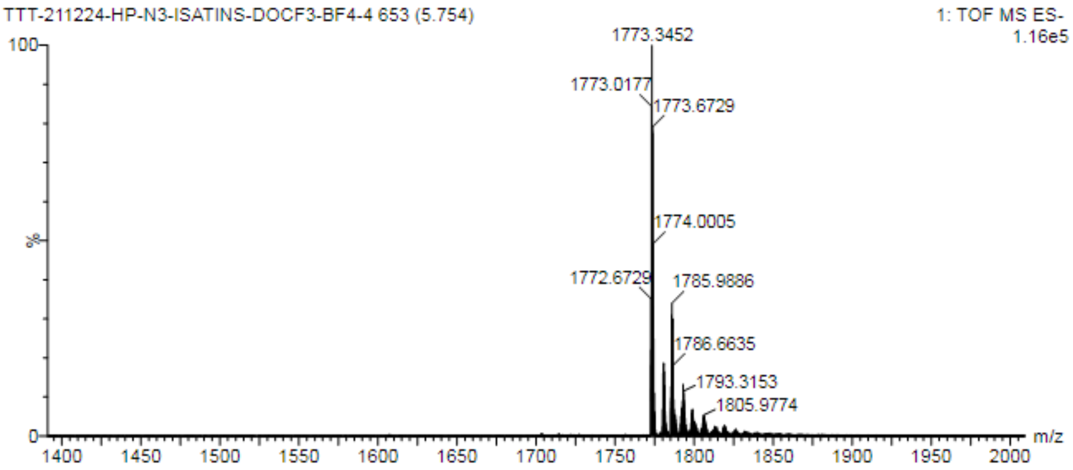

LC Trace and Mass of O1k

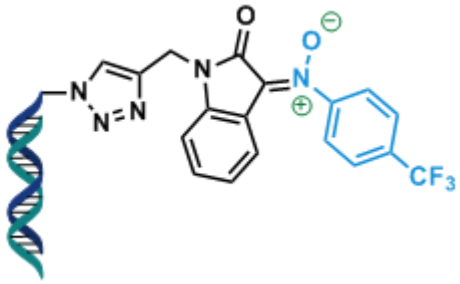

Following General Procedure vi

Yield: 80%

Exact mass: 5307.53

Triply charged mass [M]/3 - 1.00794, calculated 1768.1691; observed 1768.0215.

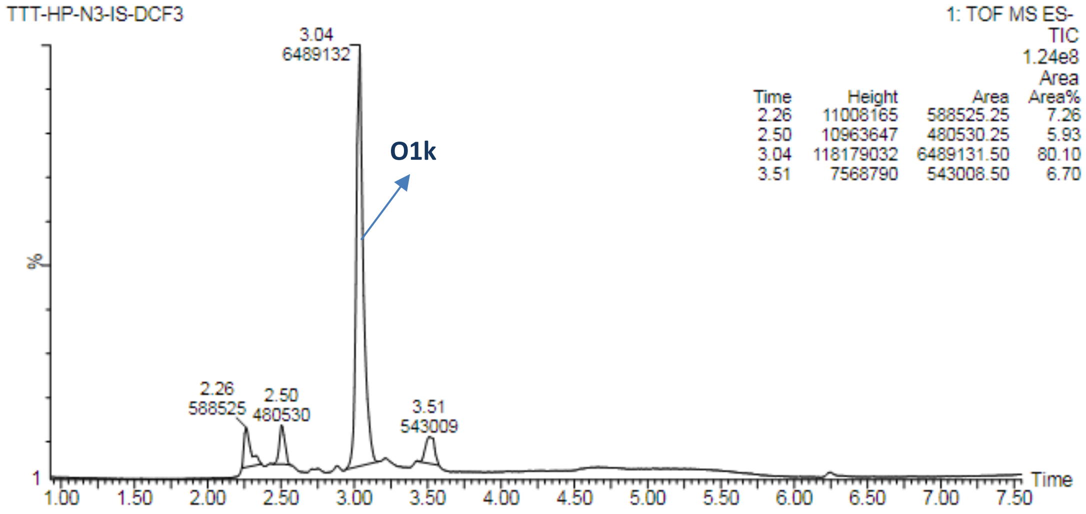

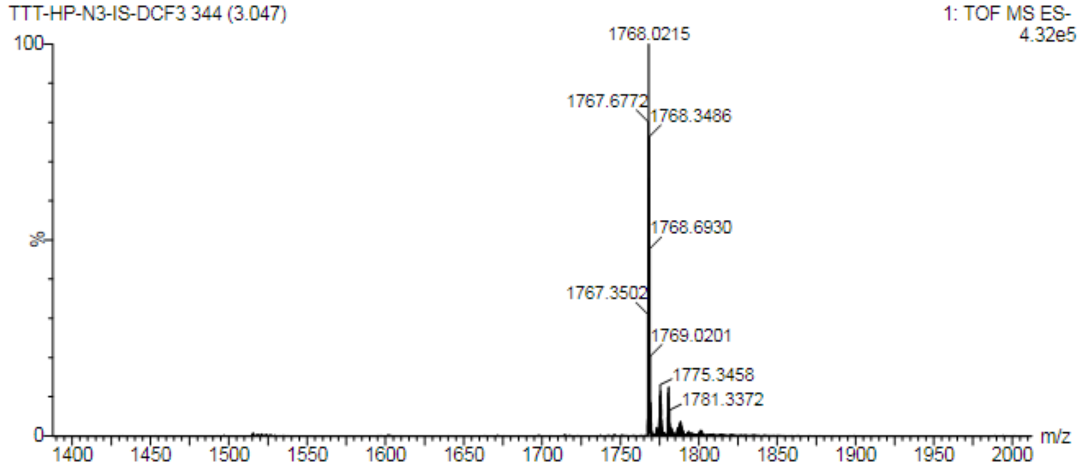

LC Trace and Mass of O1l

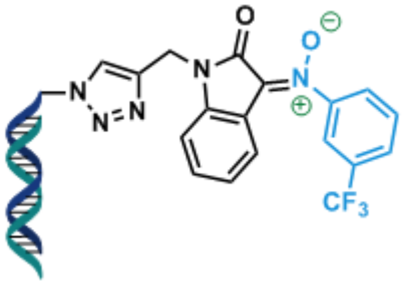

Following General Procedure vi

Yield: 84%

Exact mass: 5307.53

Triply charged mass [M]/3 - 1.00794, calculated 1768.1691; observed 1768.0215.

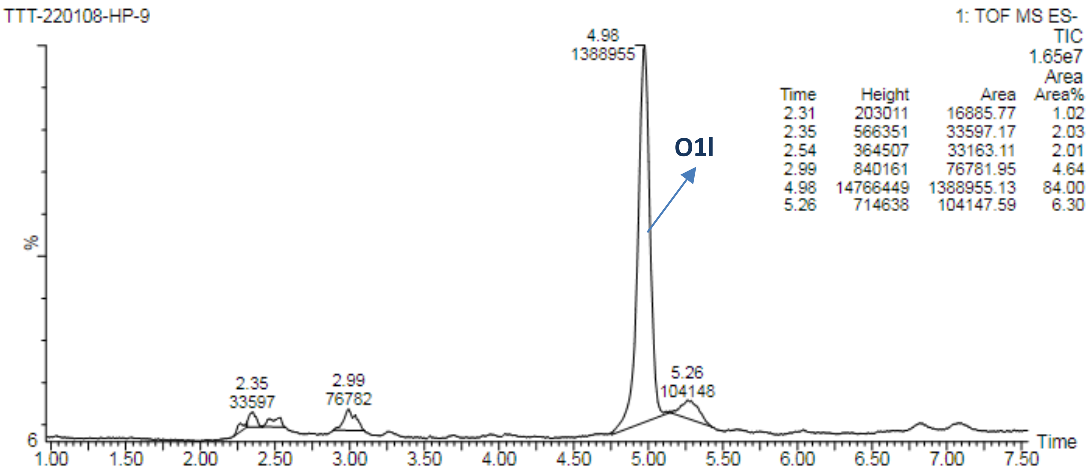

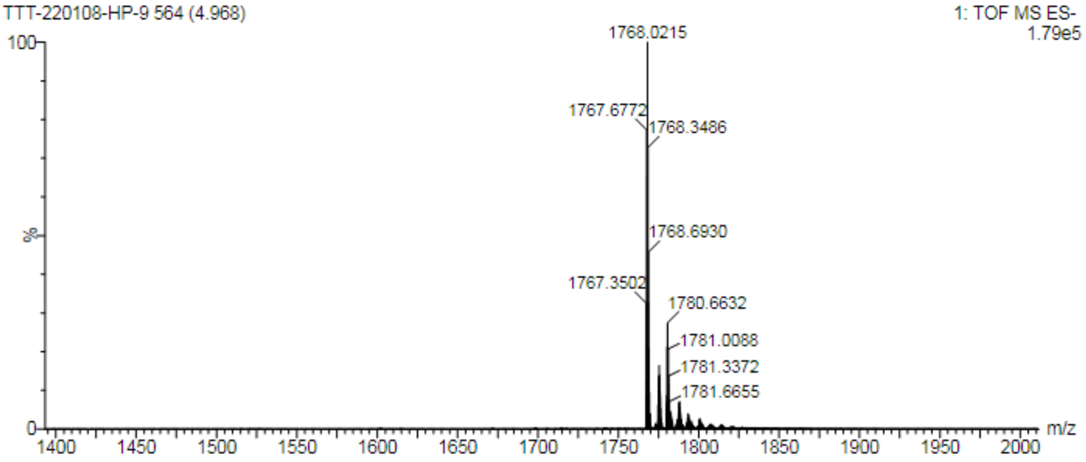

LC Trace and Mass of O1m

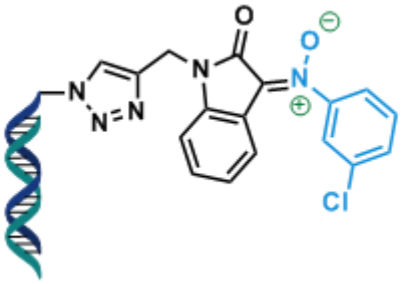

Following General Procedure vi

Yield: 80%

Exact mass: 5273.88

Triply charged mass [M]/3 - 1.00794, calculated 1756.9524; observed 1757.0034.

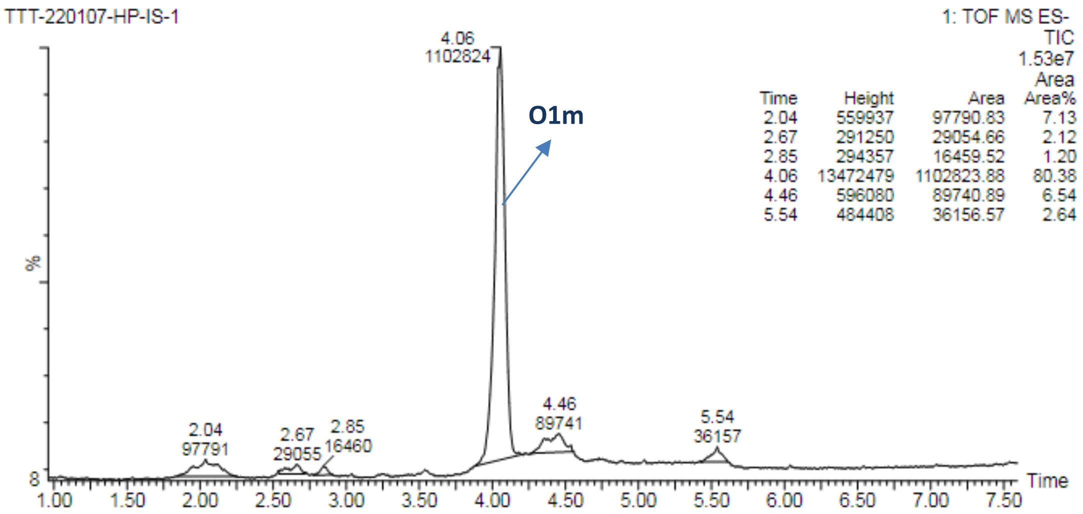

Time	Height	Area	Area%
2.04	559937	97790.83	7.13
2.67	291250	29054.66	2.12
2.85	294357	16459.52	1.20
4.06	13472479	1102823.88	80.38
4.46	596080	89740.89	6.54
5.54	484408	36156.57	2.64

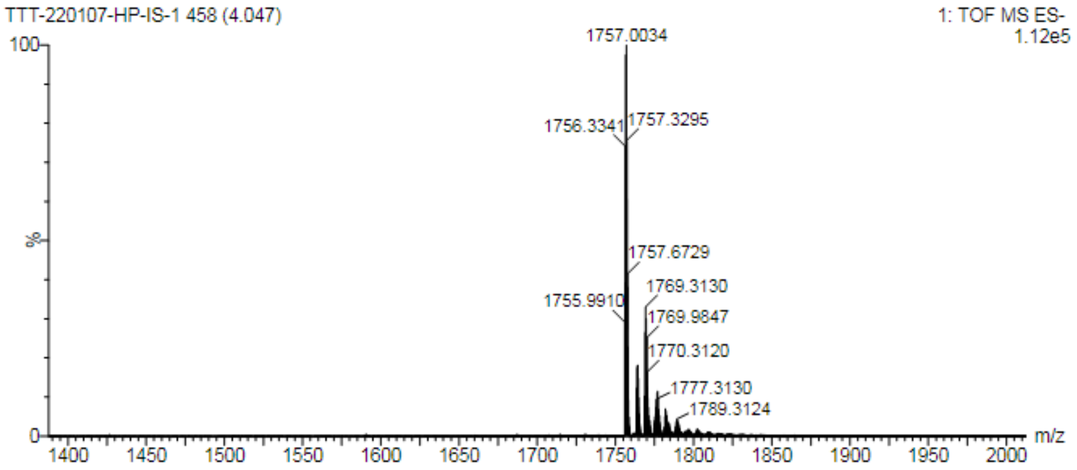

LC Trace and Mass of O1n

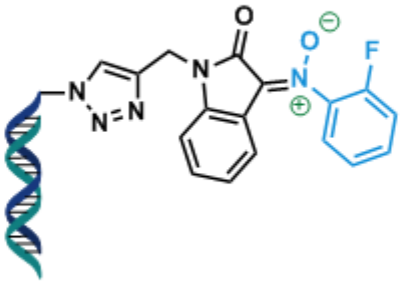

Following General Procedure vi

Yield: 98%

Exact mass: 5257.53

Triply charged mass [M]/3 - 1.00794, calculated 1751.5024; observed 1751.4216.

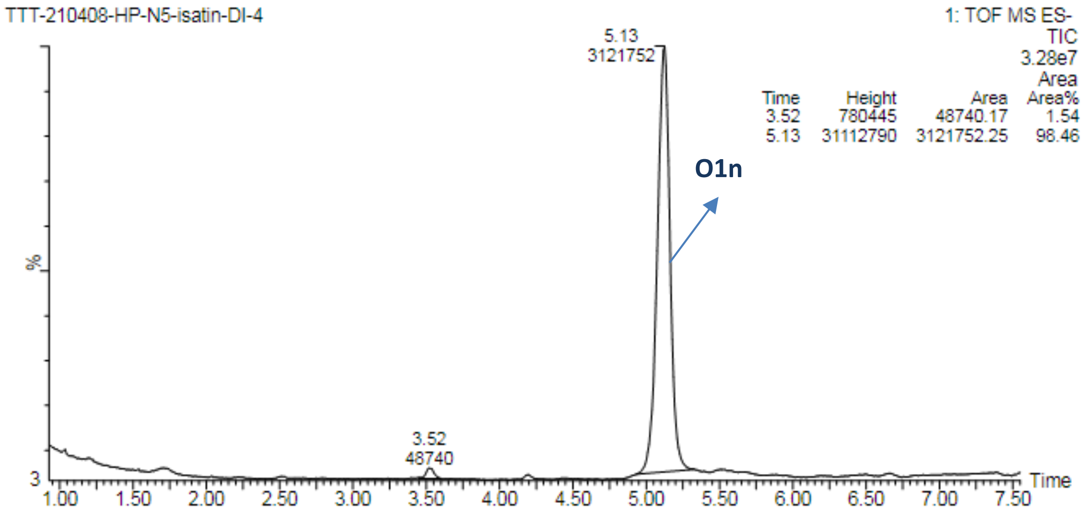

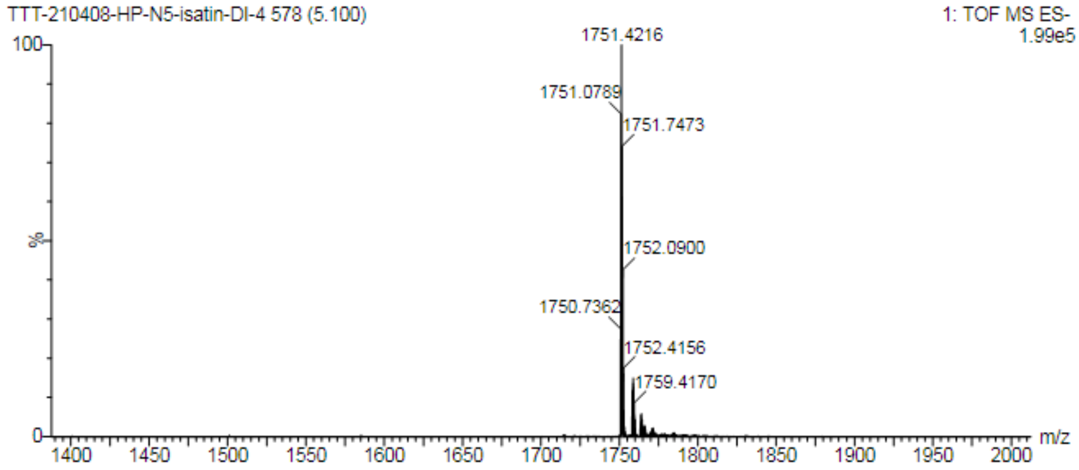

LC Trace and Mass of O1o

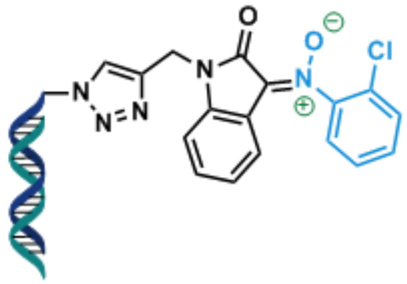

Following General Procedure vi

Yield: 76%

Exact mass: 5273.98

Triply charged mass [M]/3 - 1.00794, calculated 1756.9857; observed 1757.0034.

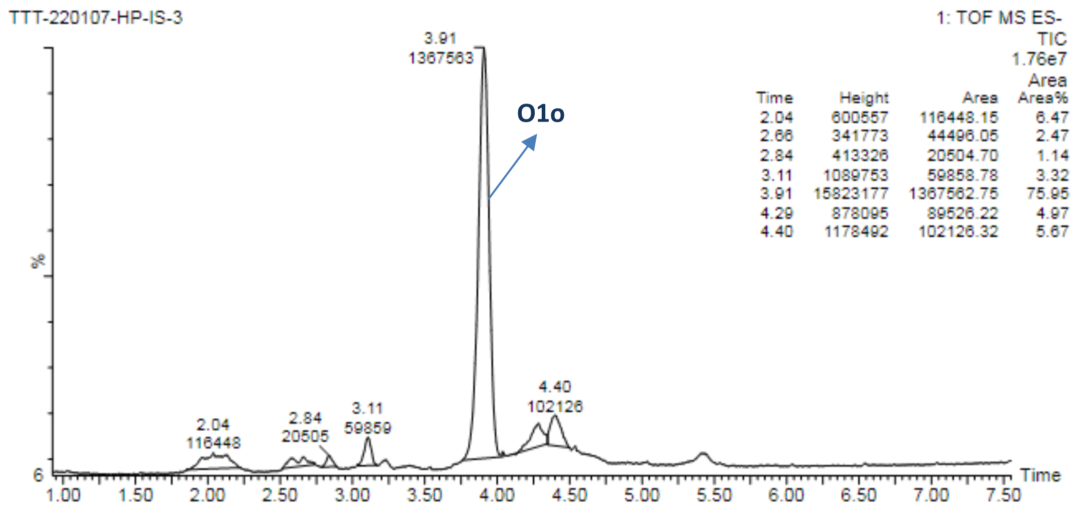

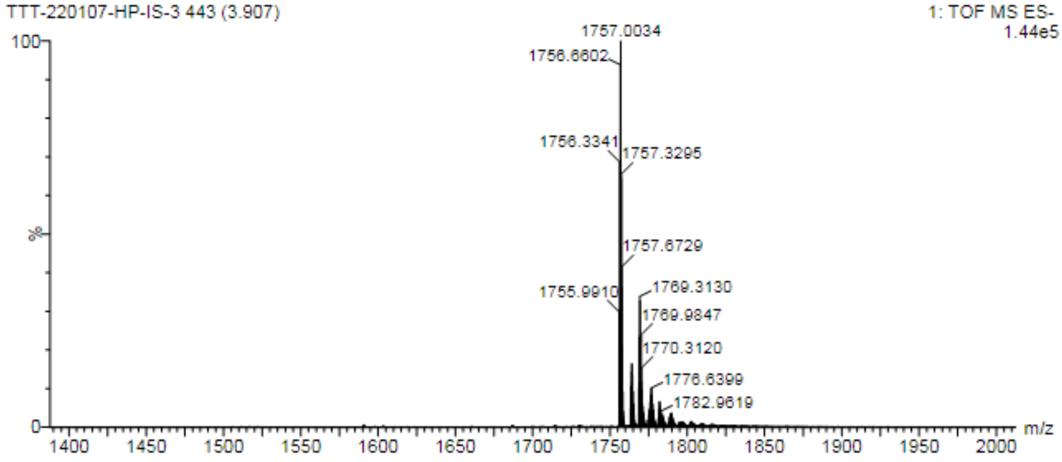

LC Trace and Mass of O1p

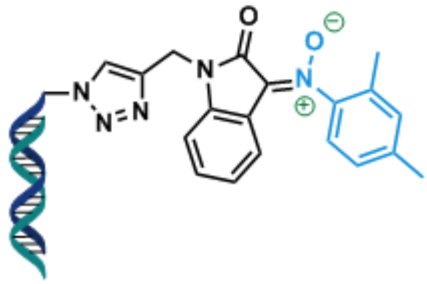

Following General Procedure vi

Yield: 60 + 25% = 85%

Exact mass: 5267.58

Triply charged mass [M]/3 - 1.00794, calculated 1754.8524; observed 1754.7650.

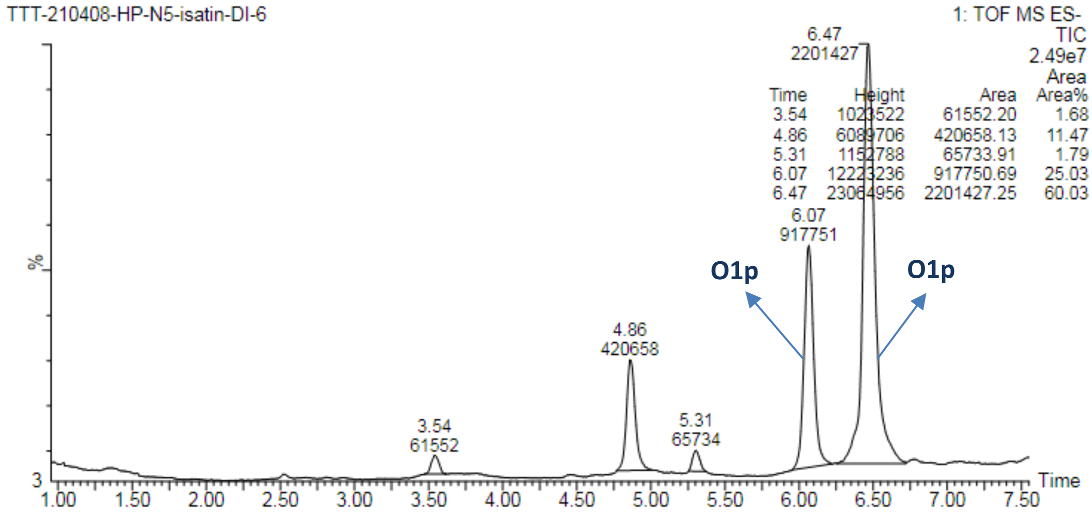

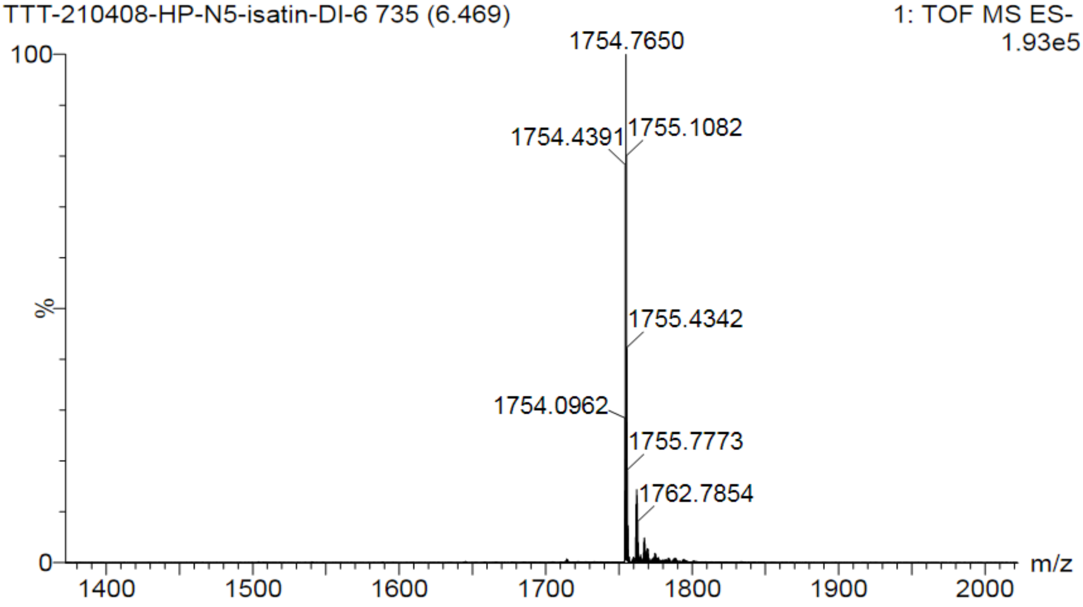

LC Trace and Mass of O1q

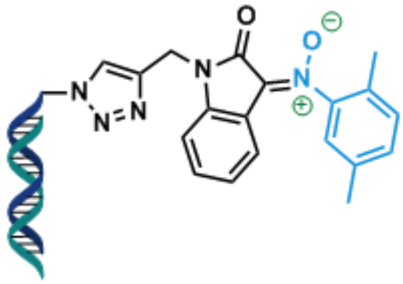

Following General Procedure vi

Yield: 91%

Exact mass: 5267.58

Triply charged mass [M]/3 - 1.00794, calculated 1754.8524; observed 1754.7822.

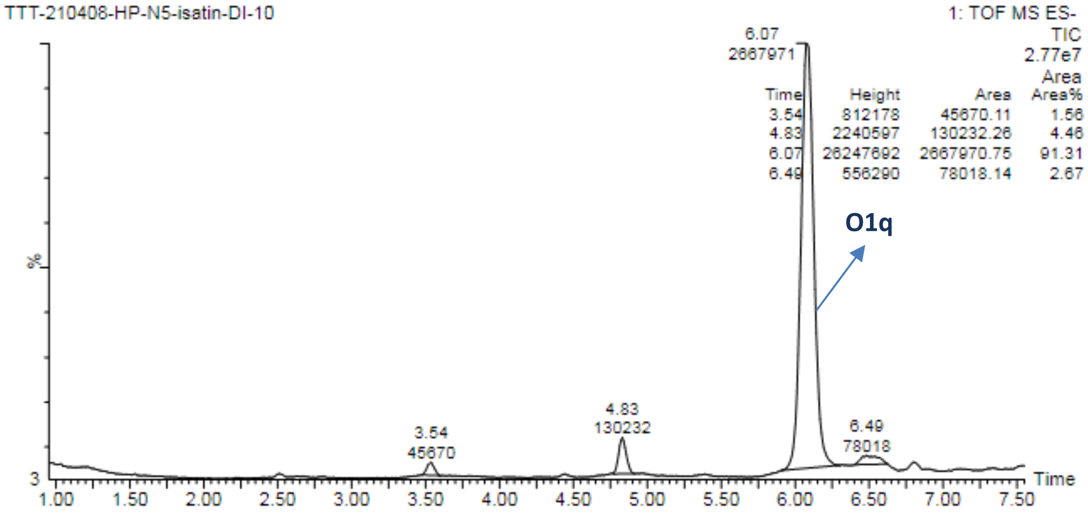

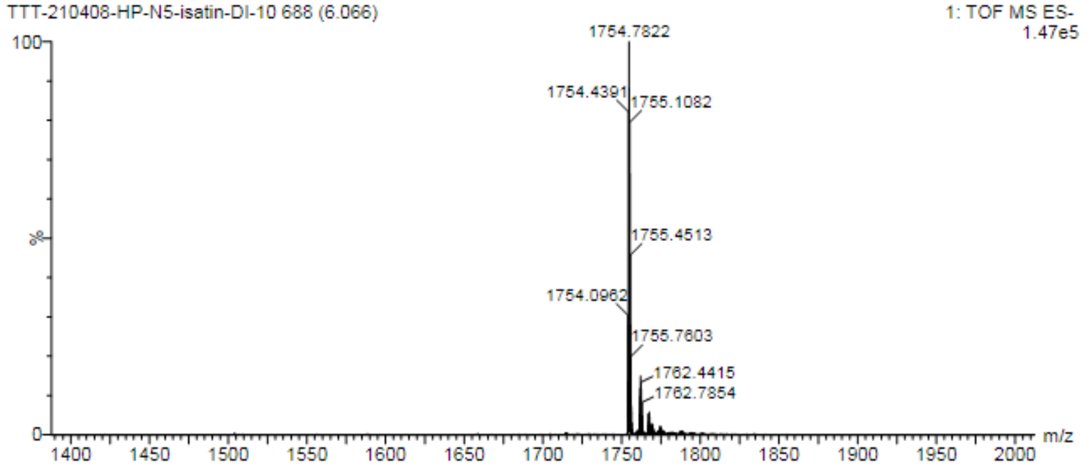

LC Trace and Mass of O1r

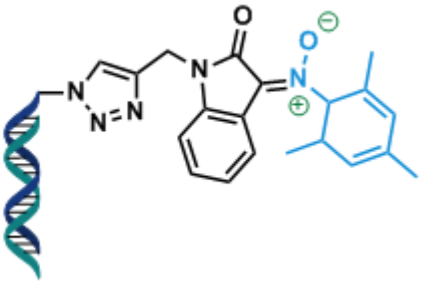

Following General Procedure vi

Yield: 74%

Exact mass: 5281.58

Triply charged mass [M]/3 - 1.00794, calculated 1759.5191; observed 1759.4342.

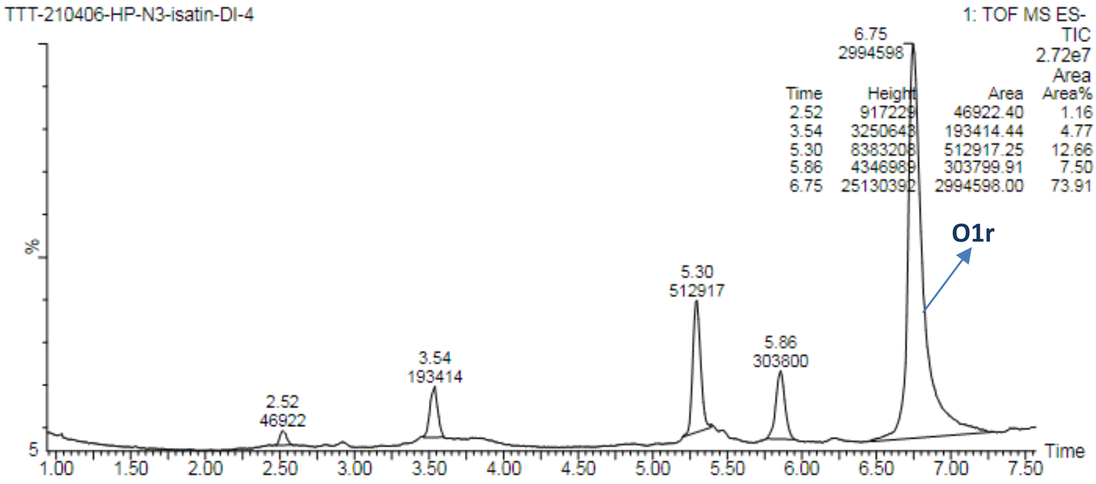

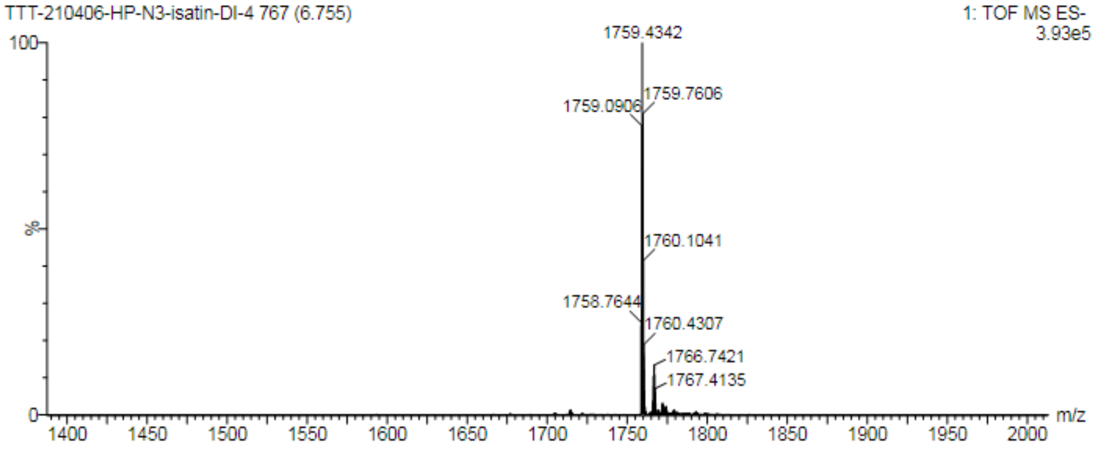

LC Trace and Mass of O1s

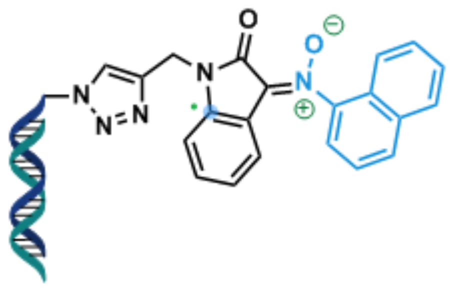

Following General Procedure vi

Yield: 92%

Exact mass: 5289.58

Triply charged mass [M]/3 - 1.00794, calculated 1762.1857; observed 1762.0977.

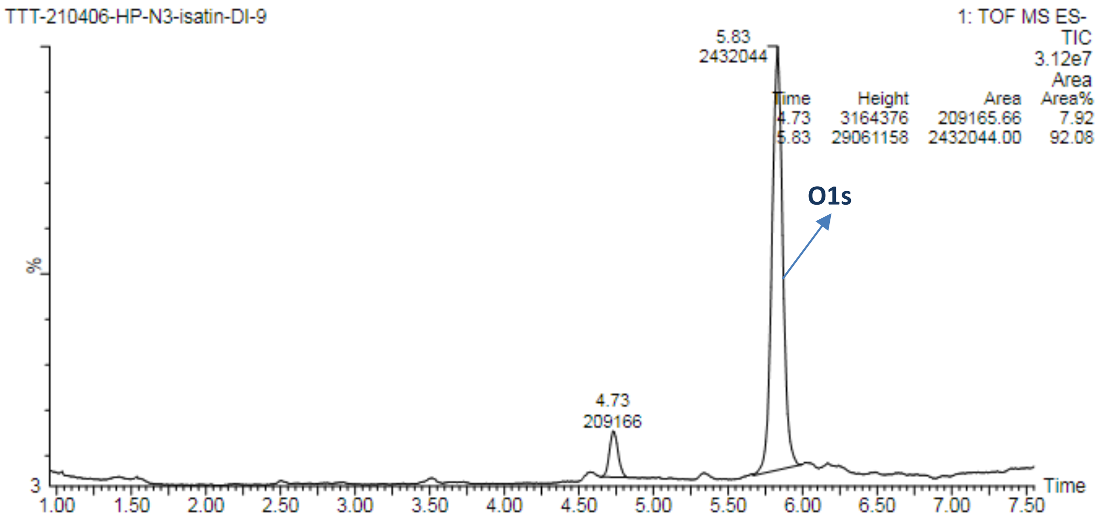

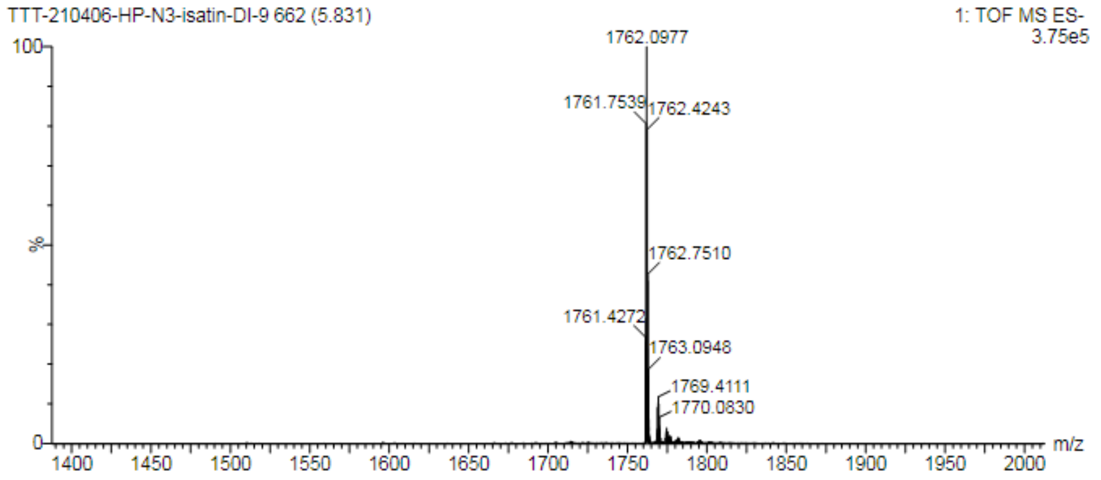

LC Trace and Mass of O1t

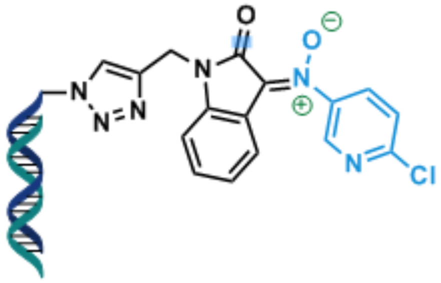

Following General Procedure vi
Yield: 92%
Exact mass: 5274.95
Triply charged mass [M]/3 - 1.00794, calculated 1757.3091; observed 1757.0206.

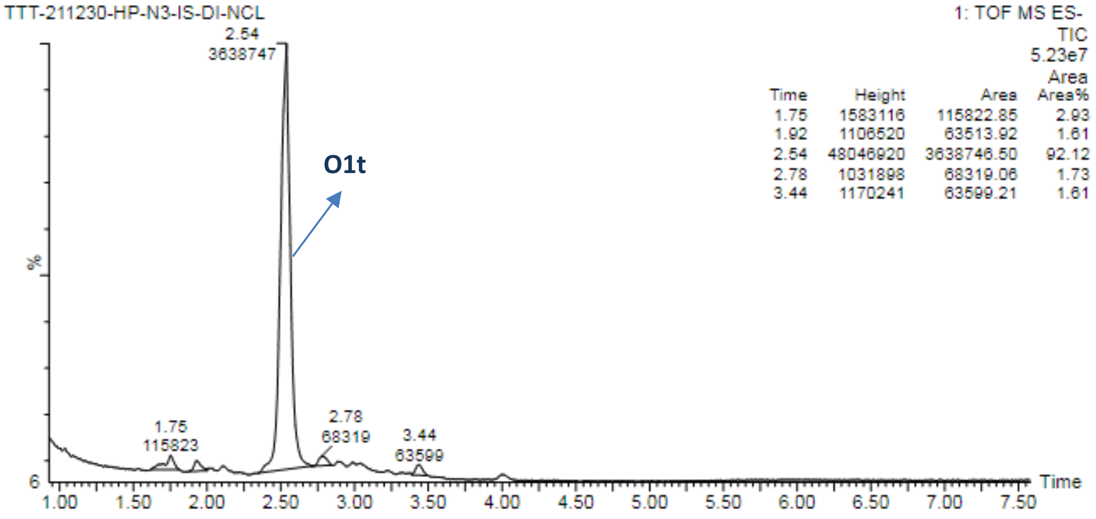

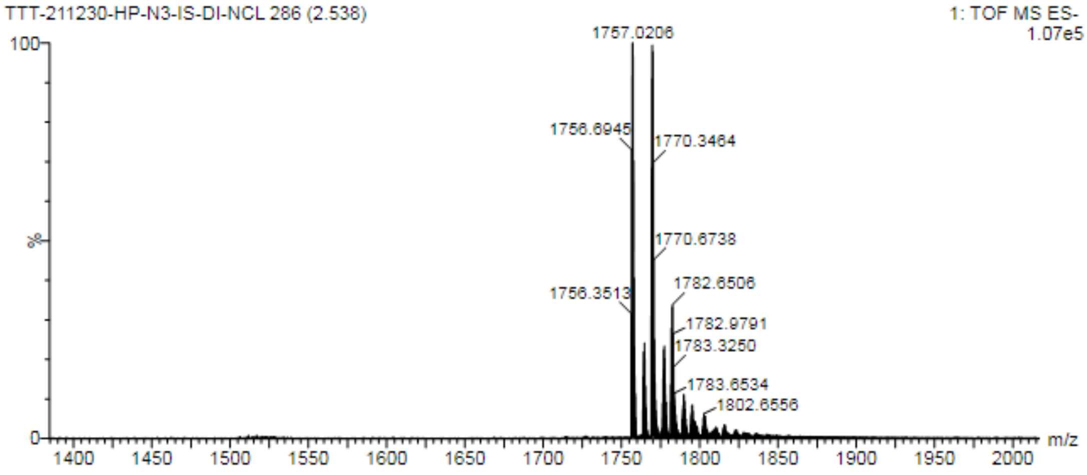

LC Trace and Mass of O1u

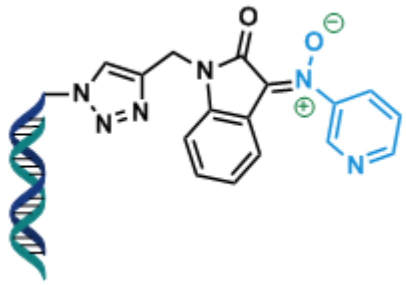

Following General Procedure vi

Yield: 76%

Exact mass: 5240.51

Triply charged mass [M]/3 - 1.00794, calculated 1745.8291; observed 1745.7118.

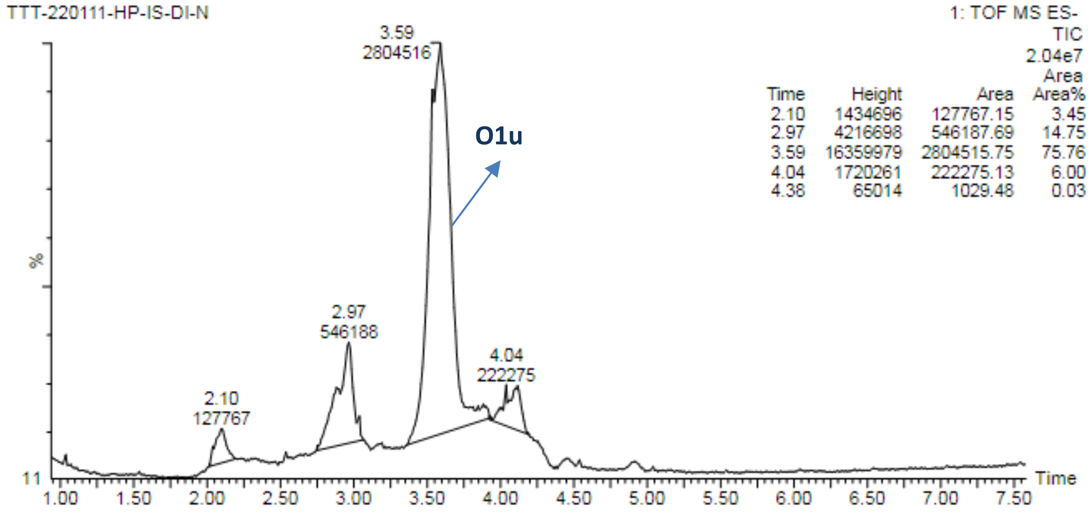

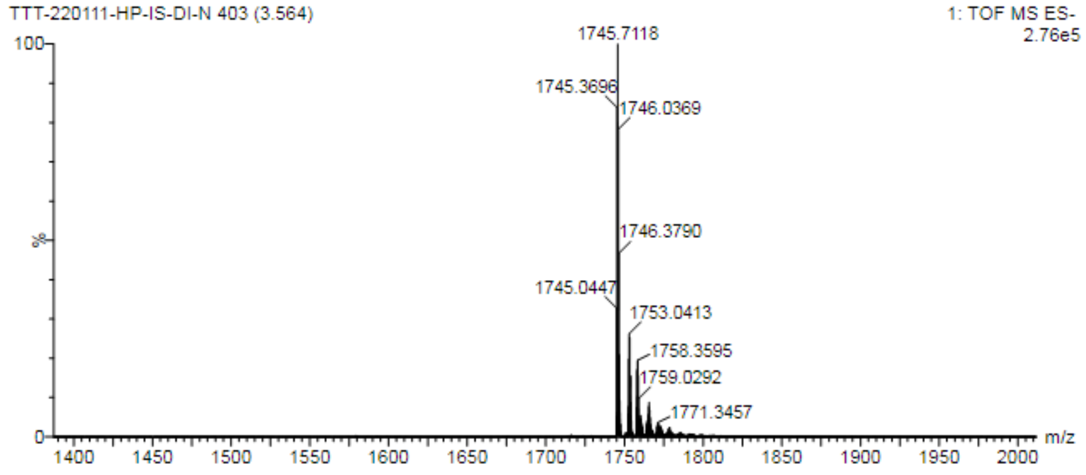

LC Trace and Mass of O2a

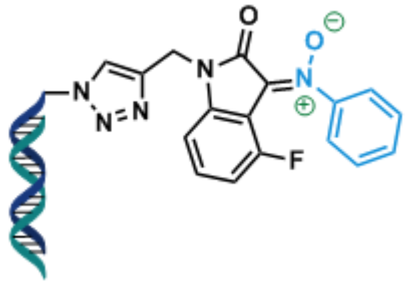

Following General Procedure vi

Yield: 85%

Exact mass: 5257.52

Triply charged mass [M]/3 - 1.00794, calculated 1751.4991; observed 1751.1560.

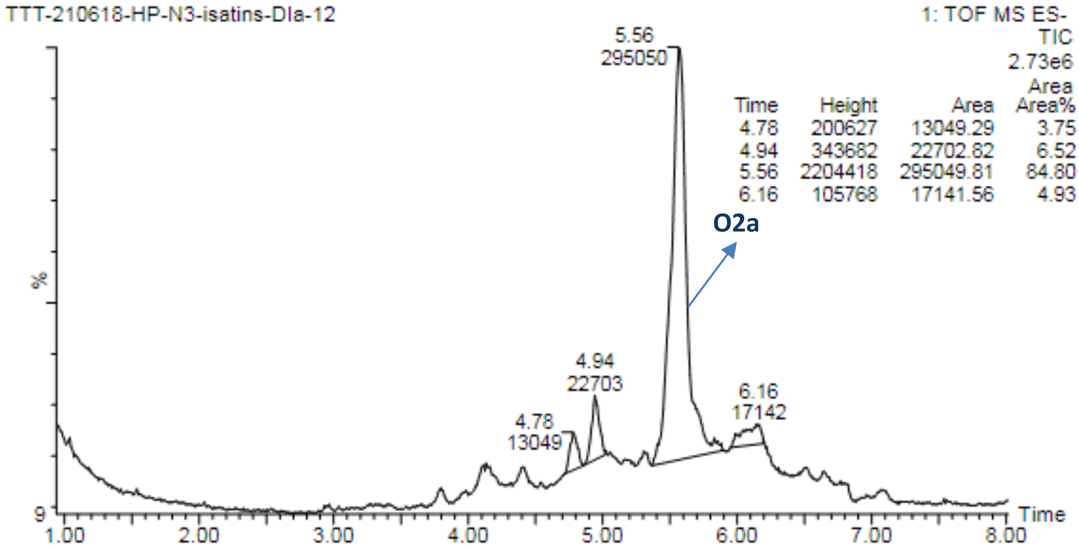

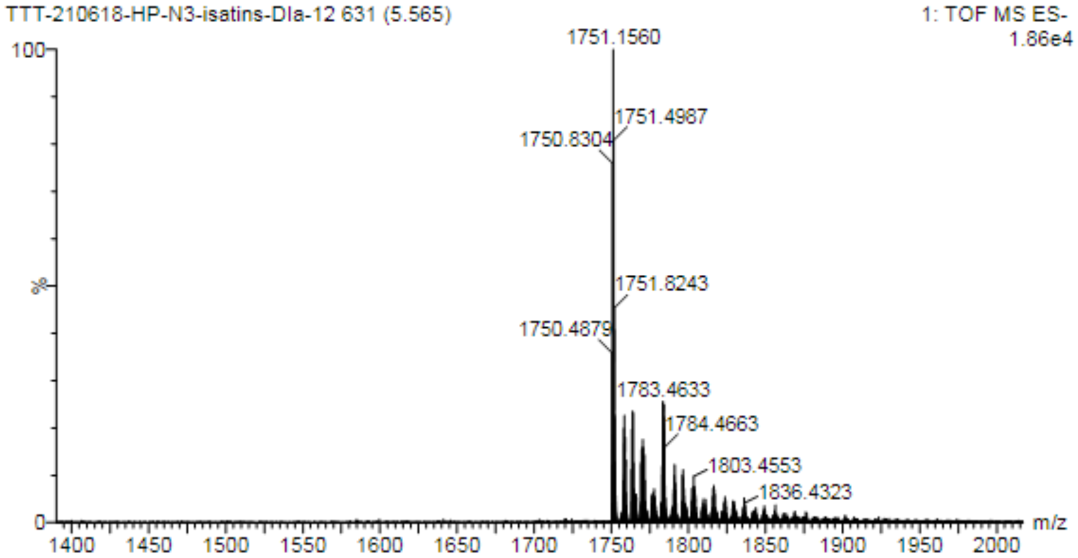

LC Trace and Mass of O2b

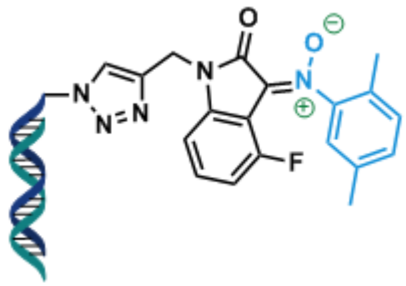

Following General Procedure vi

Yield: 29 + 53% = 82%

Exact mass: 5285.57

Triply charged mass [M]/3 - 1.00794, calculated 1760.8491; observed 1760.4547.

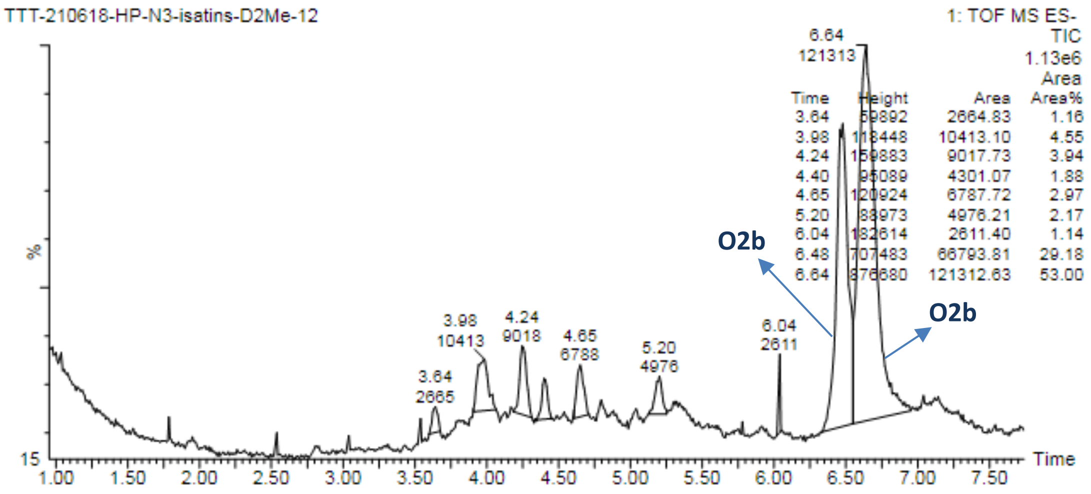

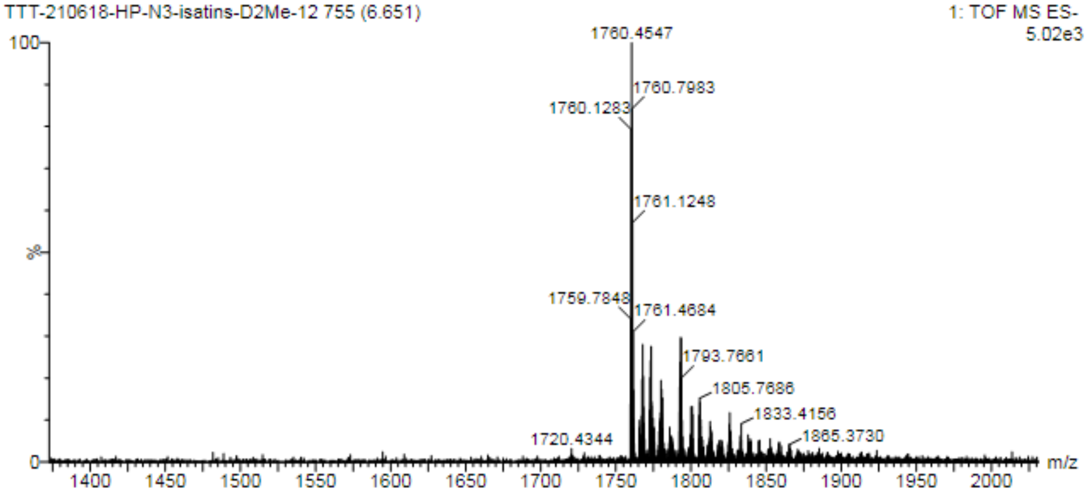

LC Trace and Mass of O2c

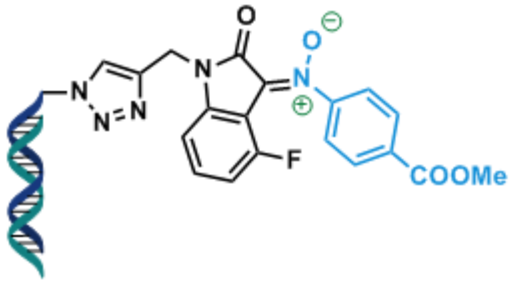

Following General Procedure vi

Yield: 74%

Exact mass: 5315.57

Triply charged mass [M]/3 - 1.00794, calculated 1770.8491; observed 1770.4498.

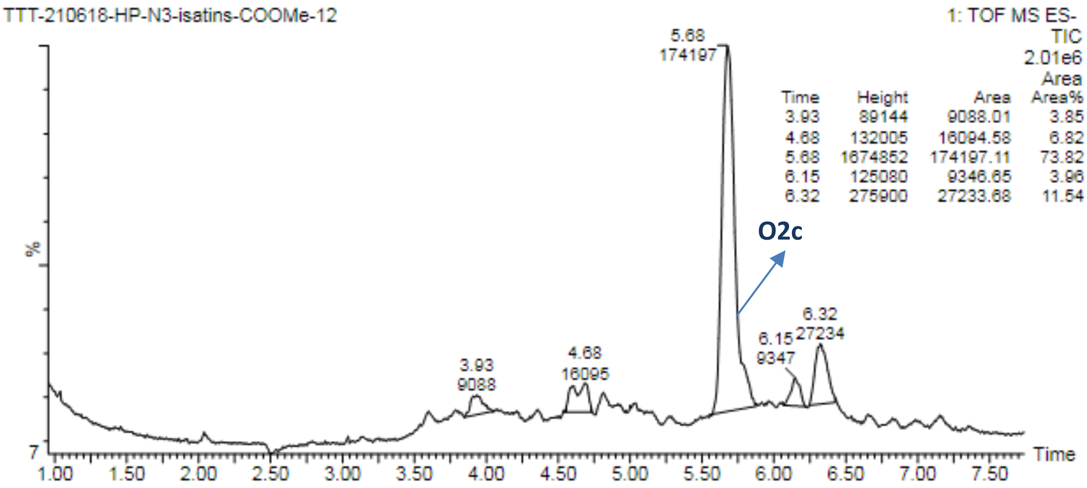

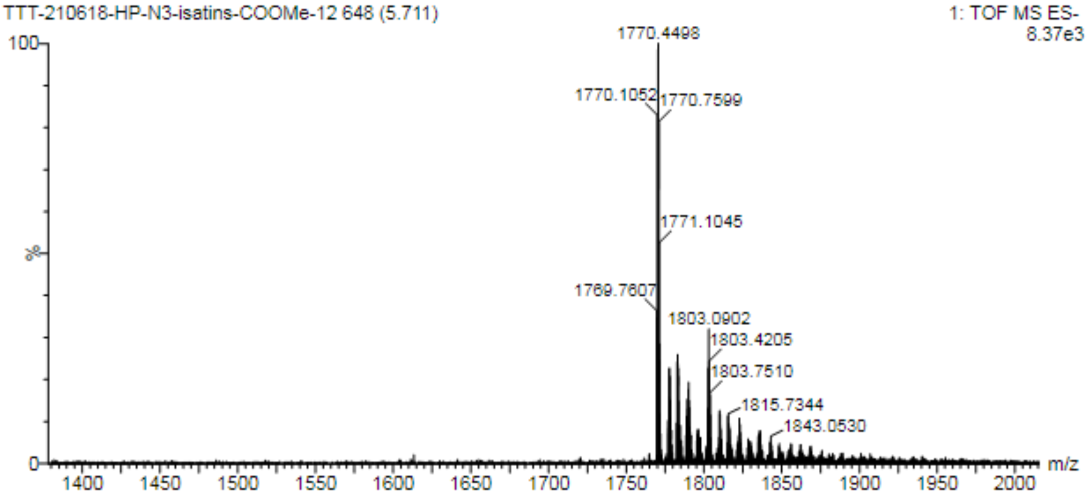

LC Trace and Mass of O3a

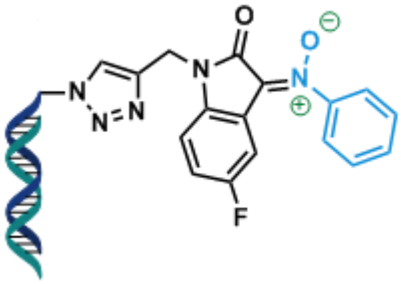

Following General Procedure vi

Yield: 74%

Exact mass: 5257.52

Triply charged mass [M]/3 - 1.00794, calculated 1751.4991; observed 1751.1560.

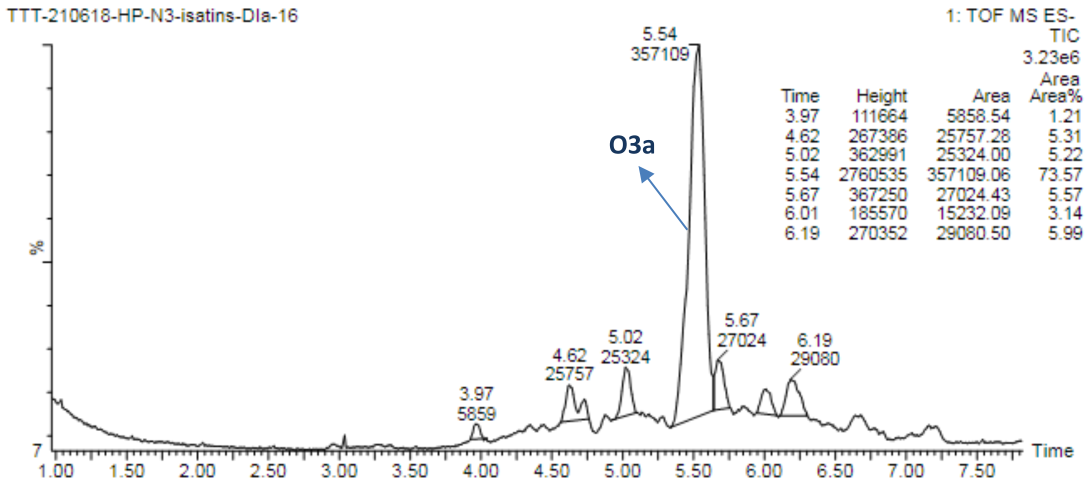

Time	Height	Area	Area%
3.97	111664	5858.54	1.21
4.62	267386	25757.28	5.31
5.02	362991	25324.00	5.22
5.54	2760535	357109.06	73.57
5.67	367250	27024.43	5.57
6.01	185570	15232.09	3.14
6.19	270352	29080.50	5.99

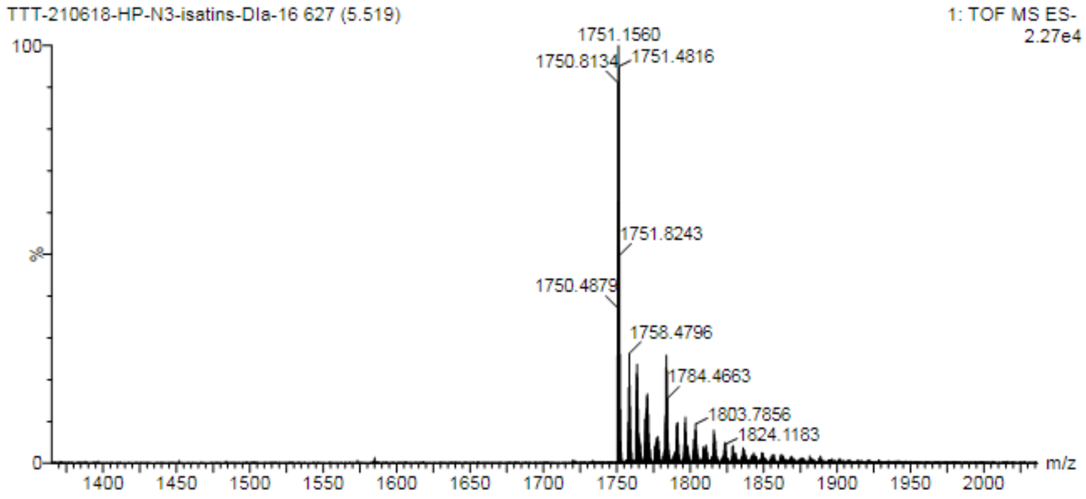

LC Trace and Mass of O3b

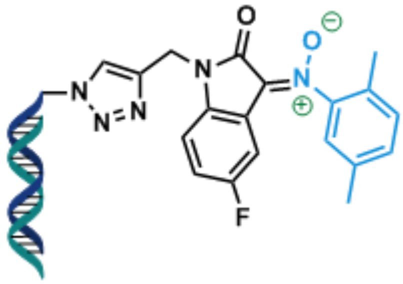

Following General Procedure vi

Yield: 75%

Exact mass: 5285.57

Triply charged mass [M]/3 - 1.00794, calculated 1760.8491; observed 1760.4547.

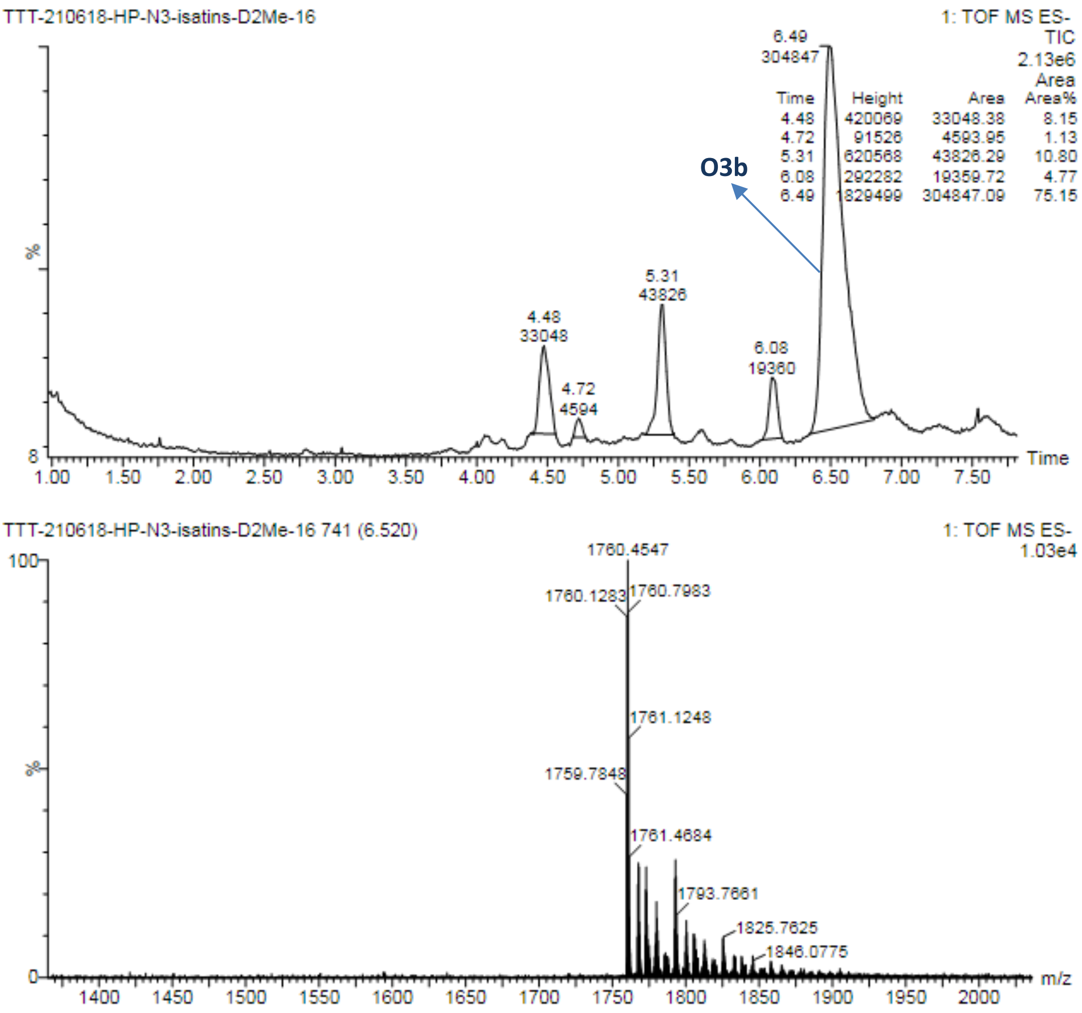

LC Trace and Mass of O3c

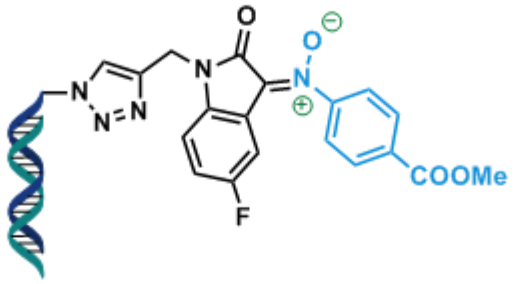

Following General Procedure vi

Yield: 75%

Exact mass: 5315.57

Triply charged mass [M]/3 - 1.00794, calculated 1770.8491; observed 1770.4326.

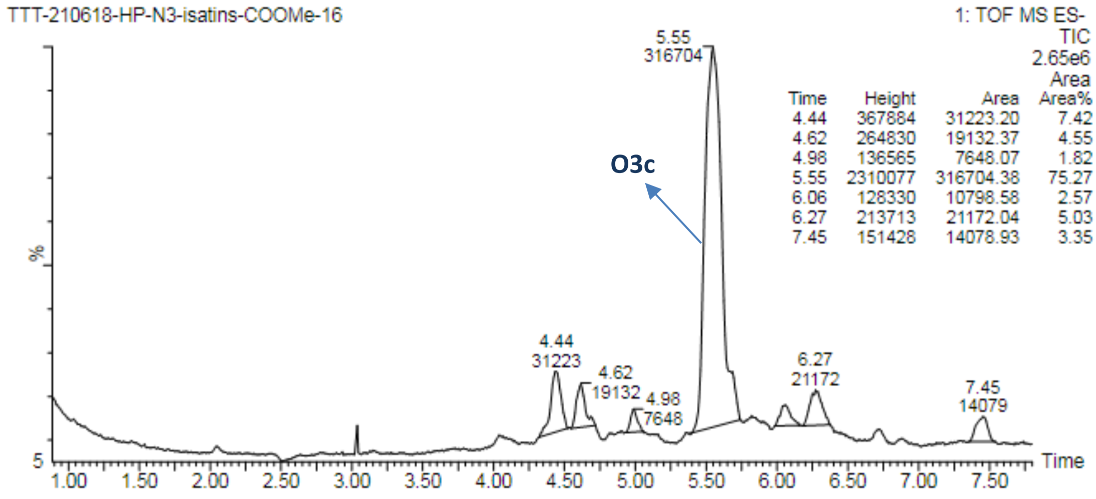

Time	Height	Area	Area%
4.44	367684	31223.20	7.42
4.62	264830	19132.37	4.55
4.96	136565	7648.07	1.82
5.55	2310077	316704.38	75.27
6.06	128330	10798.58	2.57
6.27	213713	21172.04	5.03
7.45	151428	14078.93	3.35

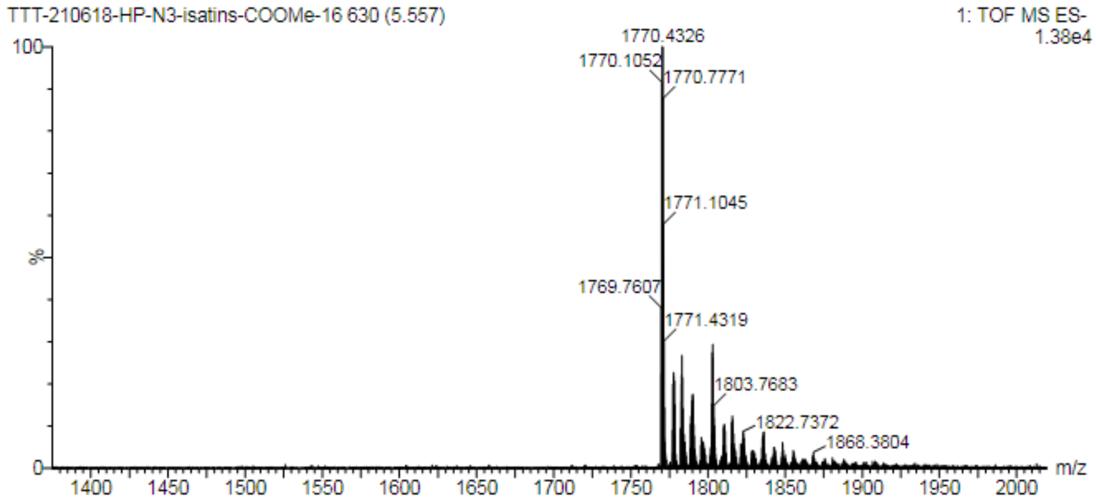

LC Trace and Mass of O4a

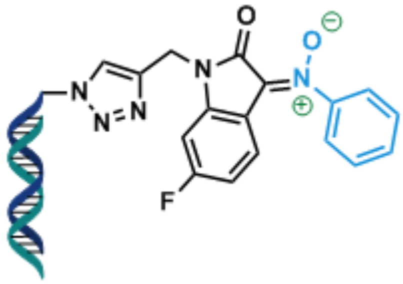

Following General Procedure vi

Yield: 72%

Exact mass: 5257.52

Triply charged mass [M]/3 - 1.00794, calculated 1751.4991; observed 1751.1389.

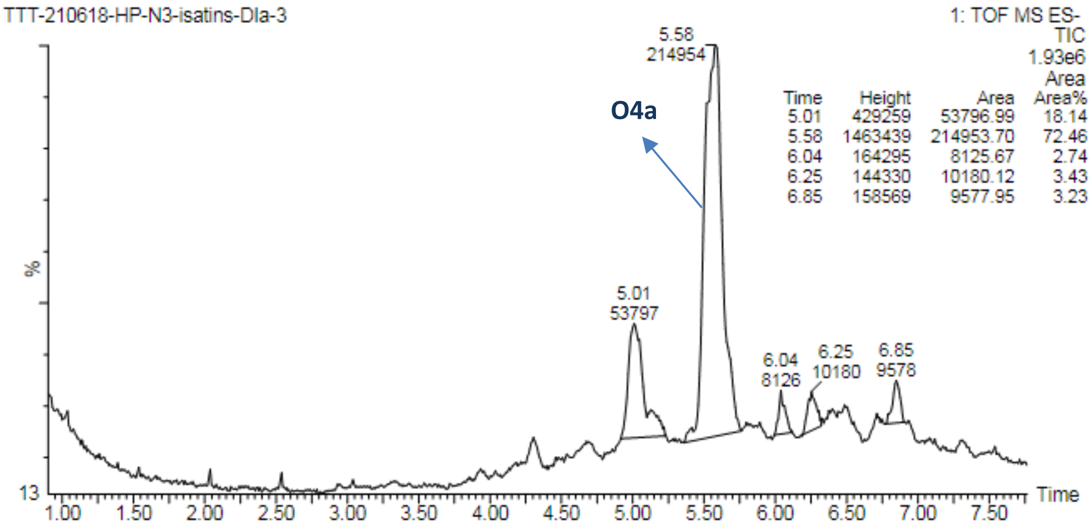

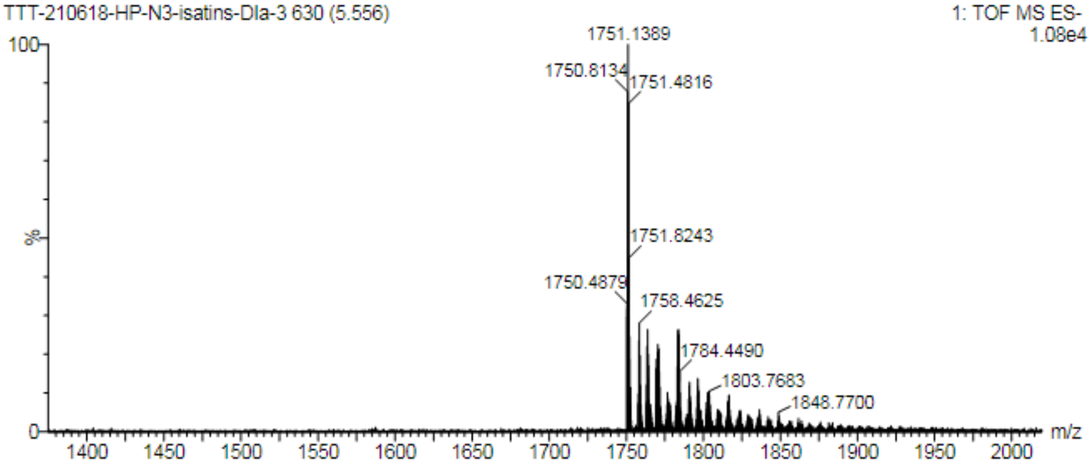

LC Trace and Mass of O4b

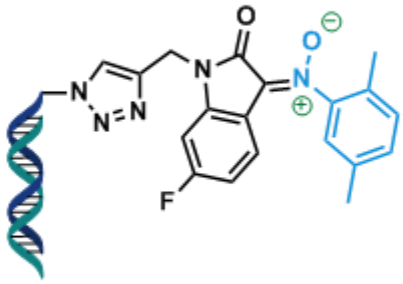

Following General Procedure vi
Yield: 83%
Exact mass: 5285.57
Triply charged mass [M]/3 - 1.00794, calculated 1760.8491; observed 1760.4547.

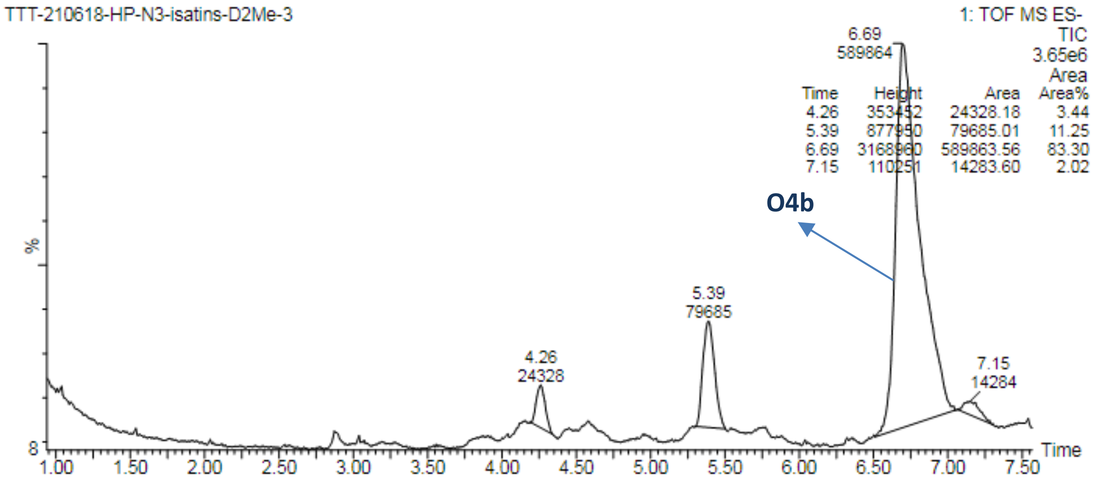

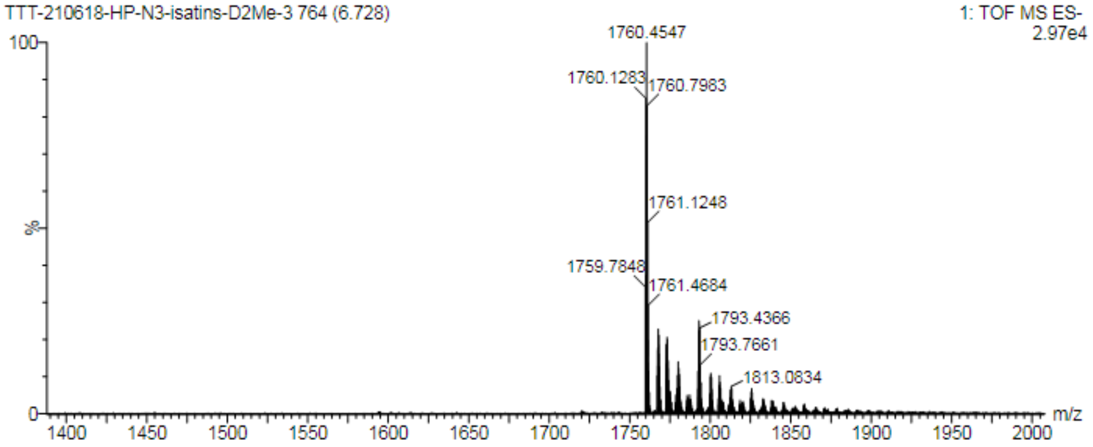

LC Trace and Mass of O4c

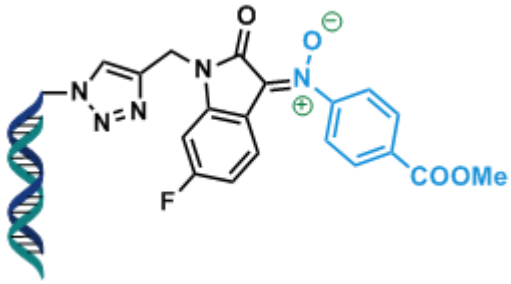

Following General Procedure vi

Yield: 83%

Exact mass: 5315.57

Triply charged mass [M]/3 - 1.00794, calculated 1770.8491; observed 1770.4498.

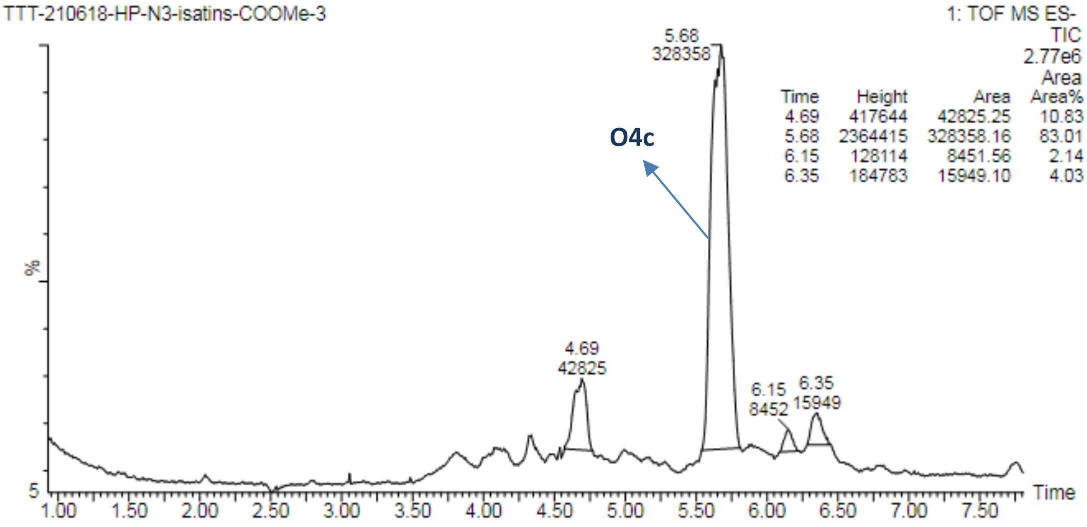

Time	Height	Area	Area%
4.69	417644	42825.25	10.83
5.68	2364415	328358.16	83.01
6.15	128114	8451.56	2.14
6.35	184783	15949.10	4.03

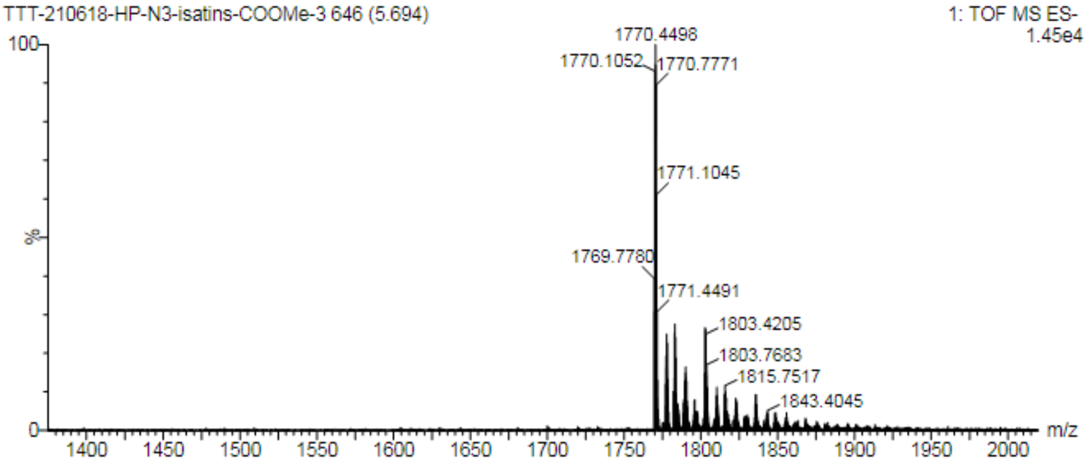

LC Trace and Mass of O5a

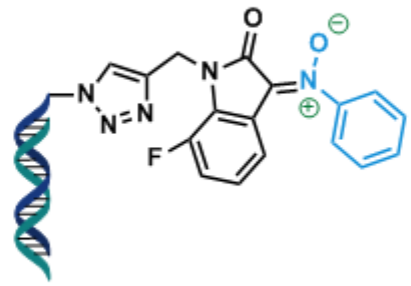

Following General Procedure vi

Yield: 71%

Exact mass: 5257.52

Triply charged mass [M]/3 - 1.00794, calculated 1751.4991; observed 1751.1560.

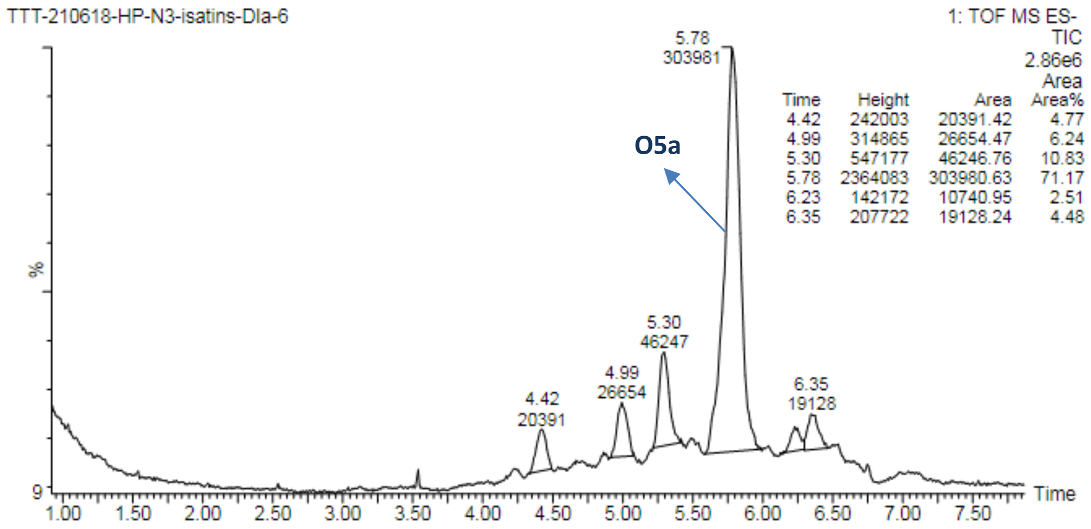

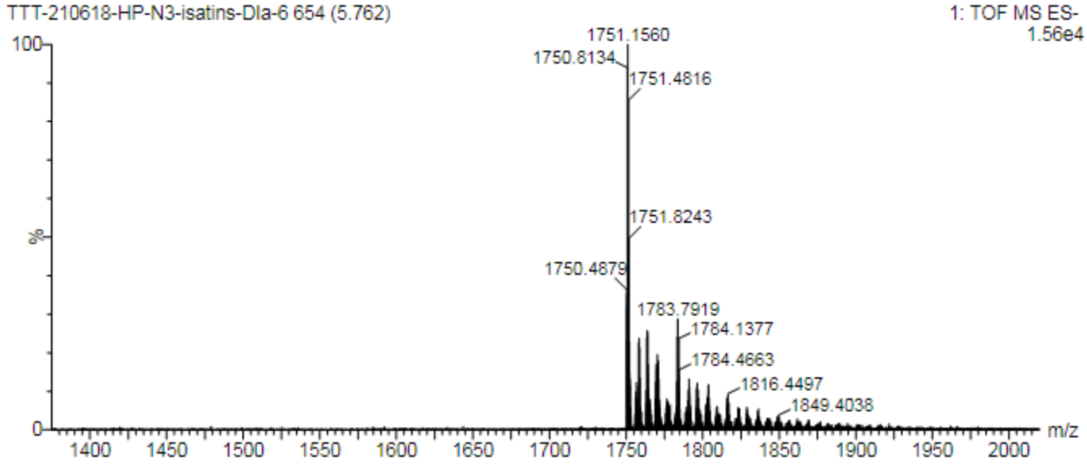

LC Trace and Mass of O5b

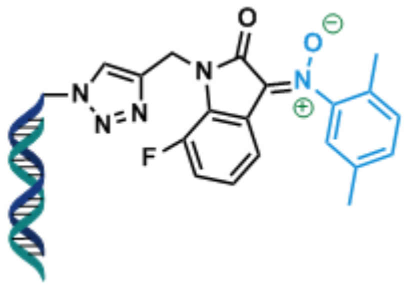

Following General Procedure vi

Yield: 90%

Exact mass: 5285.57

Triply charged mass [M]/3 - 1.00794, calculated 1760.8491; observed 1760.4547.

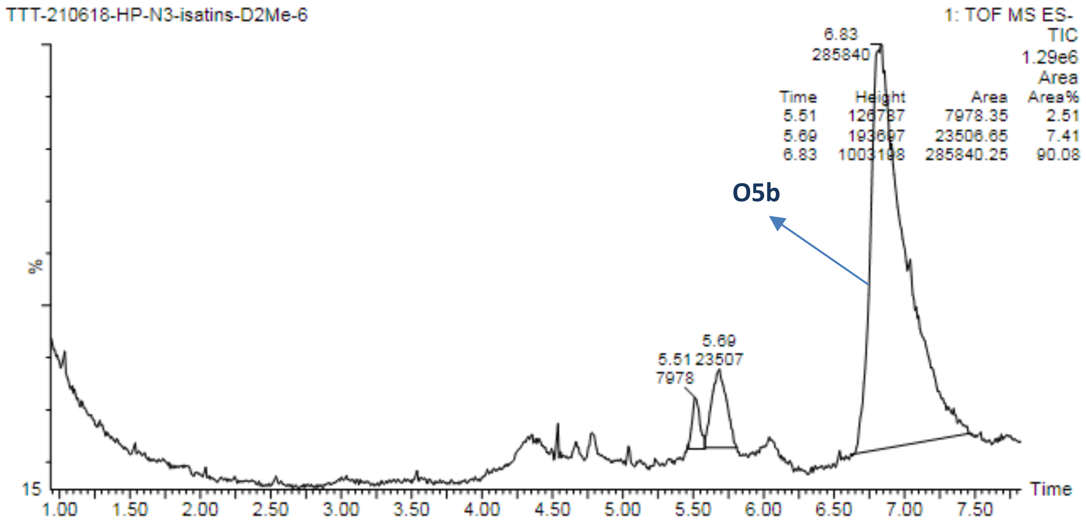

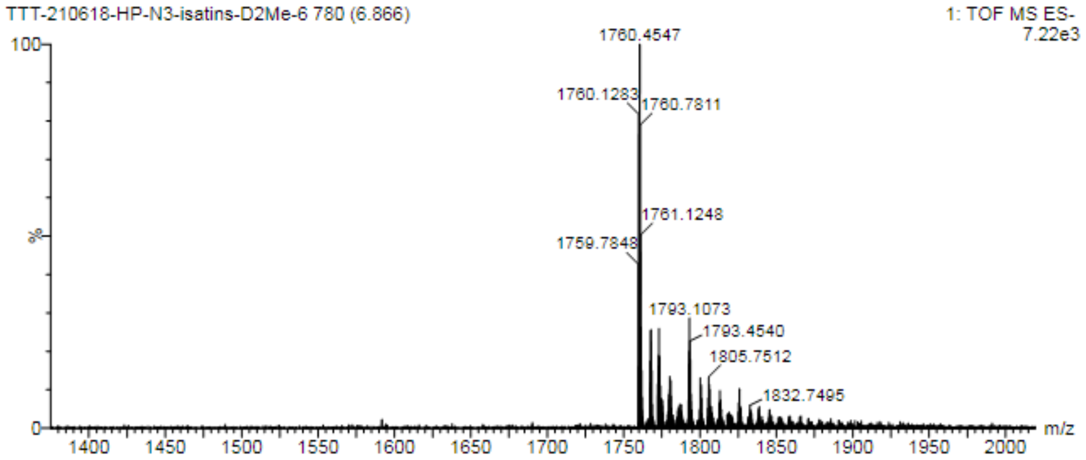

LC Trace and Mass of O5c

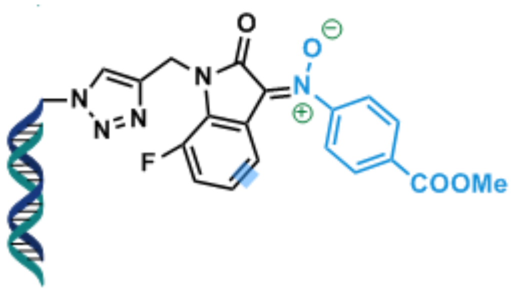

Following General Procedure vi
Yield: 71%
Exact mass: 5315.57
Triply charged mass [M]/3 - 1.00794, calculated 1770.8491; observed 1770.4498.

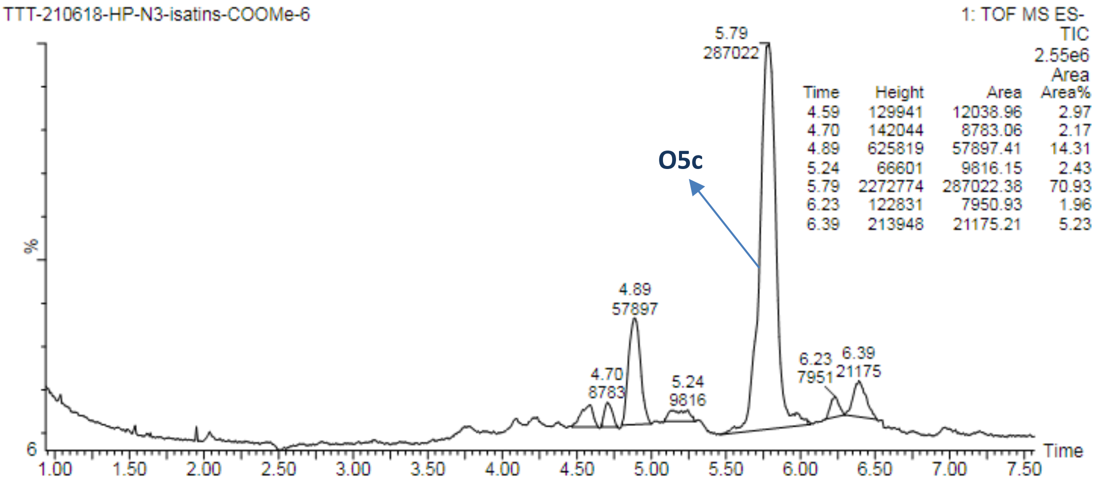

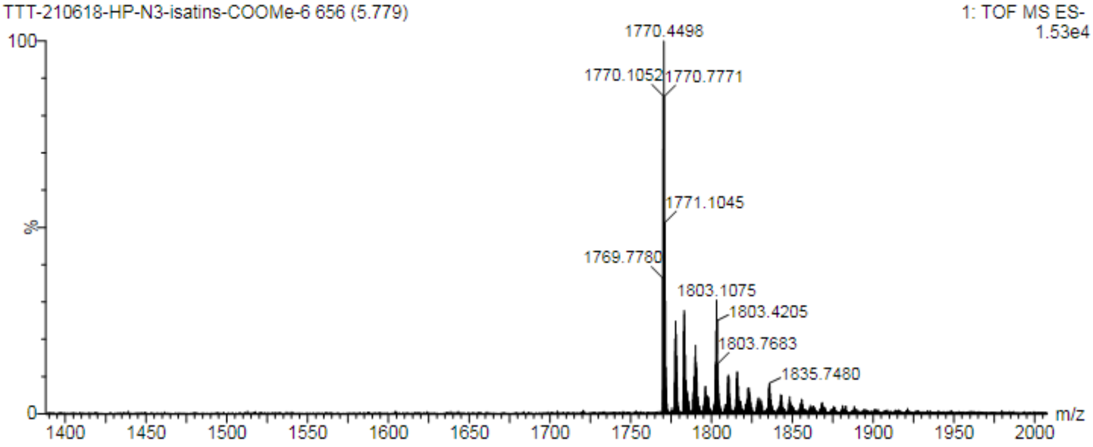

LC Trace and Mass of O6a

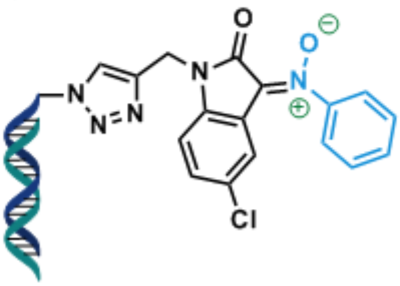

Following General Procedure vi

Yield: 85%

Exact mass: 5273.98

Triply charged mass [M]/3 - 1.00794, calculated 1756.9857; observed 1756.8147.

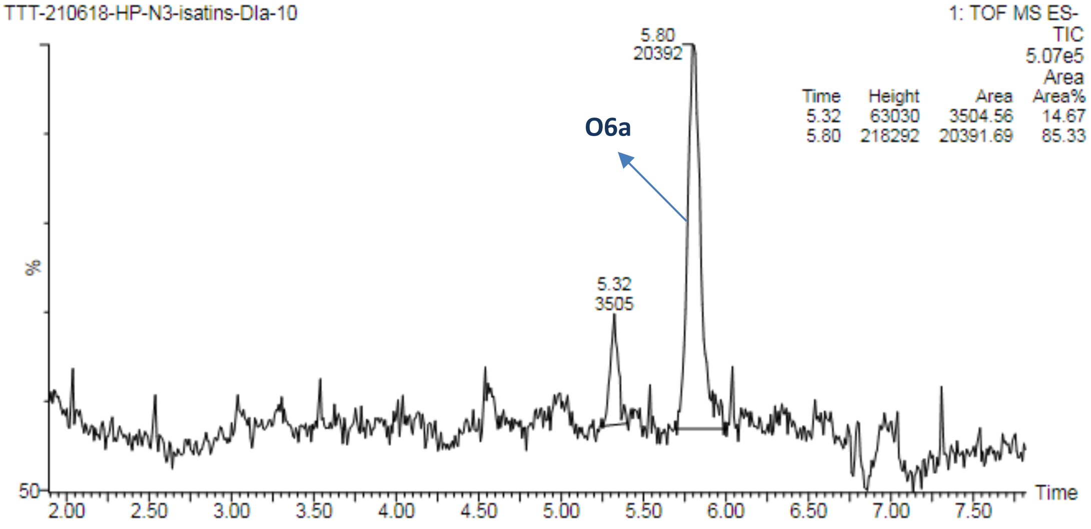

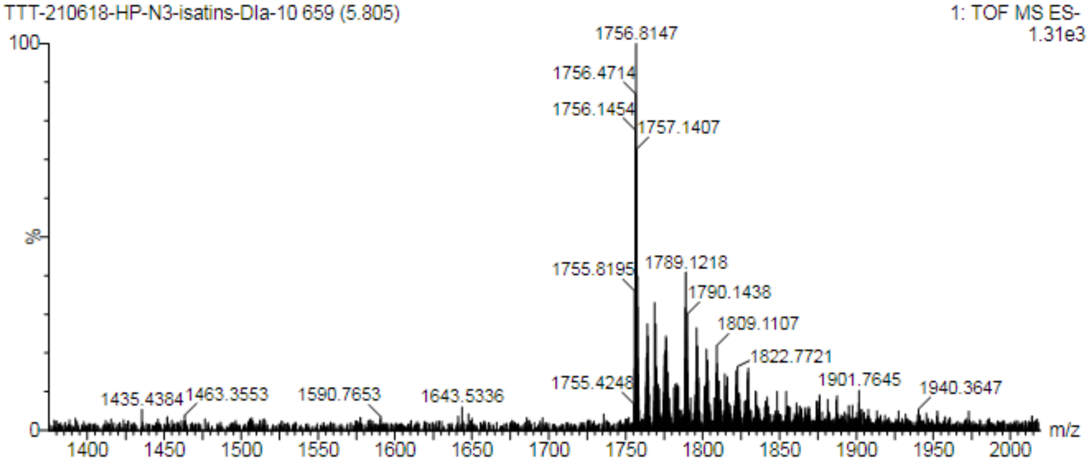

LC Trace and Mass of O6b

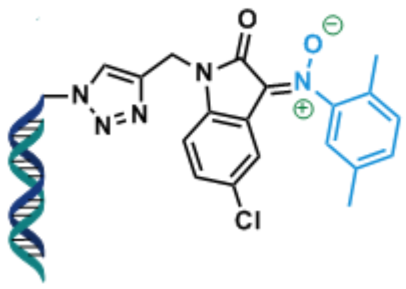

Following General Procedure vi

Yield: 81%

Exact mass: 5302.03

Triply charged mass [M]/3 - 1.00794, calculated 1766.3357; observed 1765.9906.

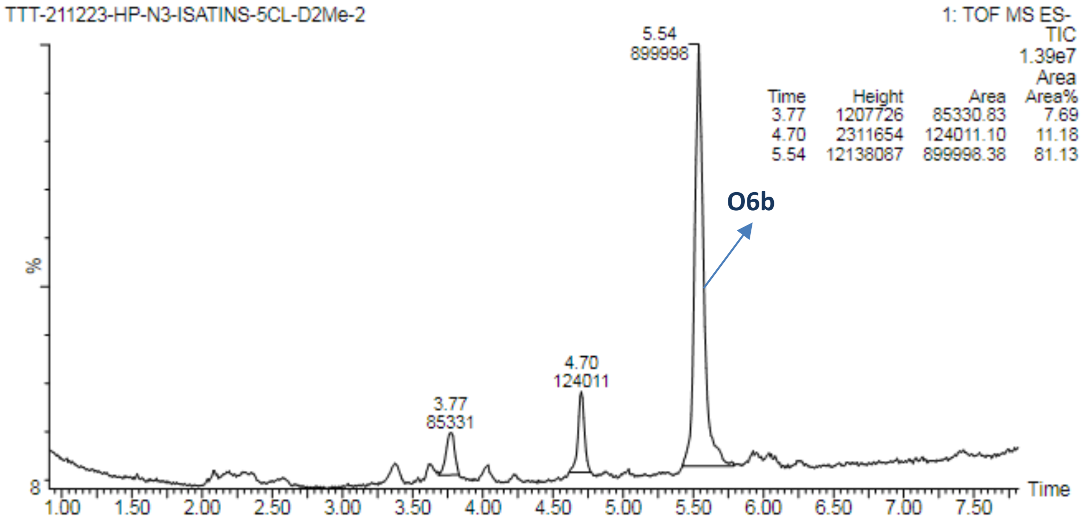

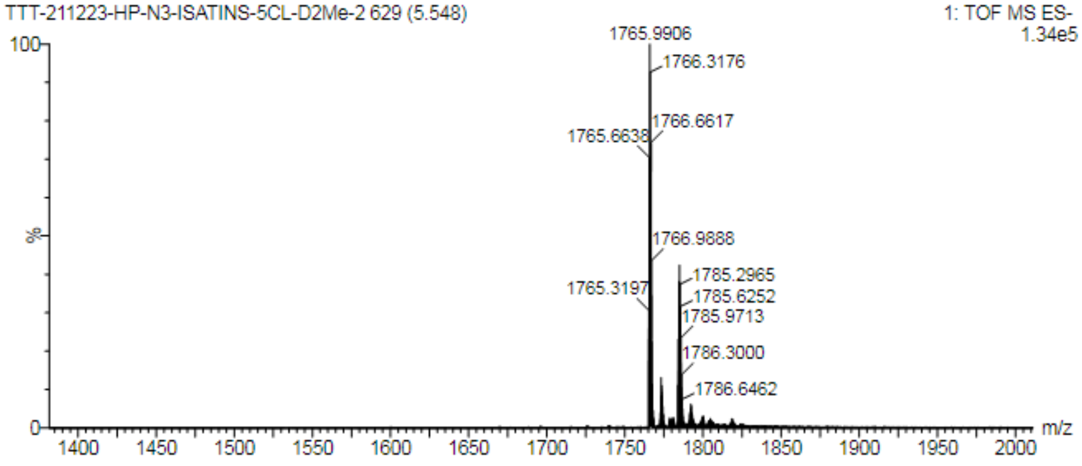

LC Trace and Mass of O6c

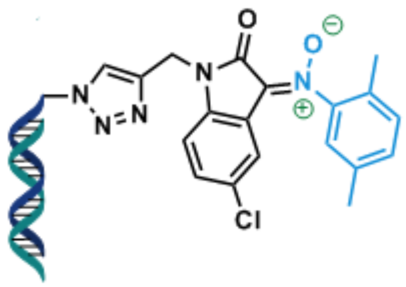

Following General Procedure vi

Yield: 93%

Exact mass: 5332.03

Triply charged mass [M]/3 - 1.00794, calculated 1776.3357; observed 1776.0878.

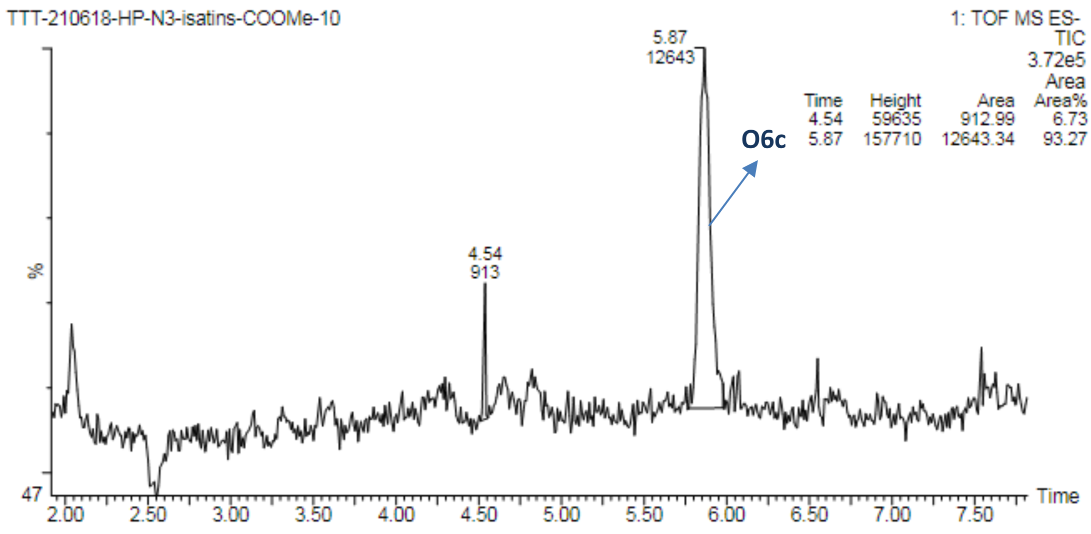

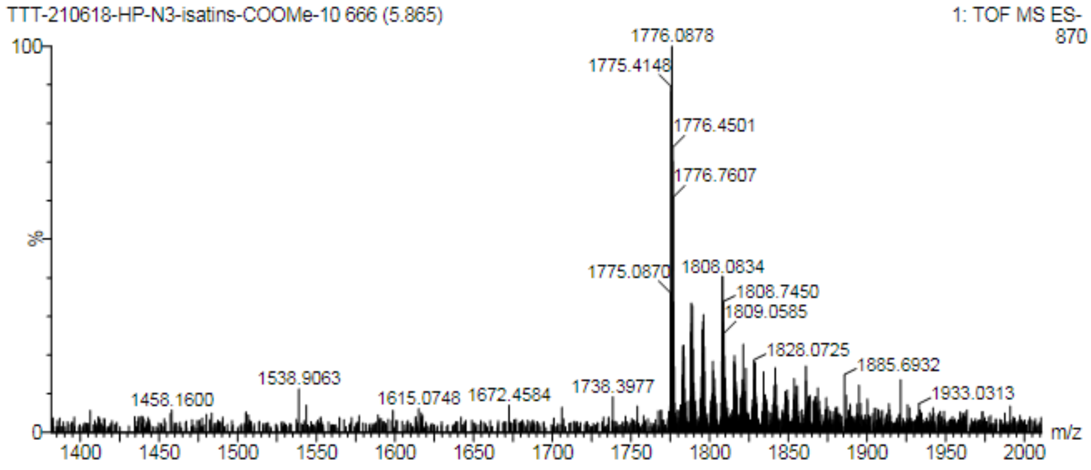

LC Trace and Mass of O7a

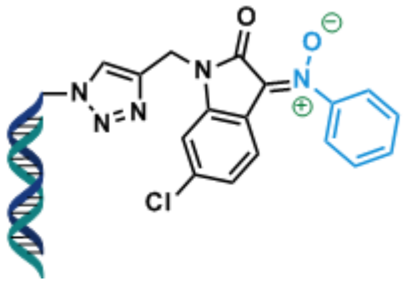

Following General Procedure vi
Yield: 81%
Exact mass: 5273.98
Triply charged mass [M]/3 - 1.00794, calculated 1756.9857; observed 1756.8147.

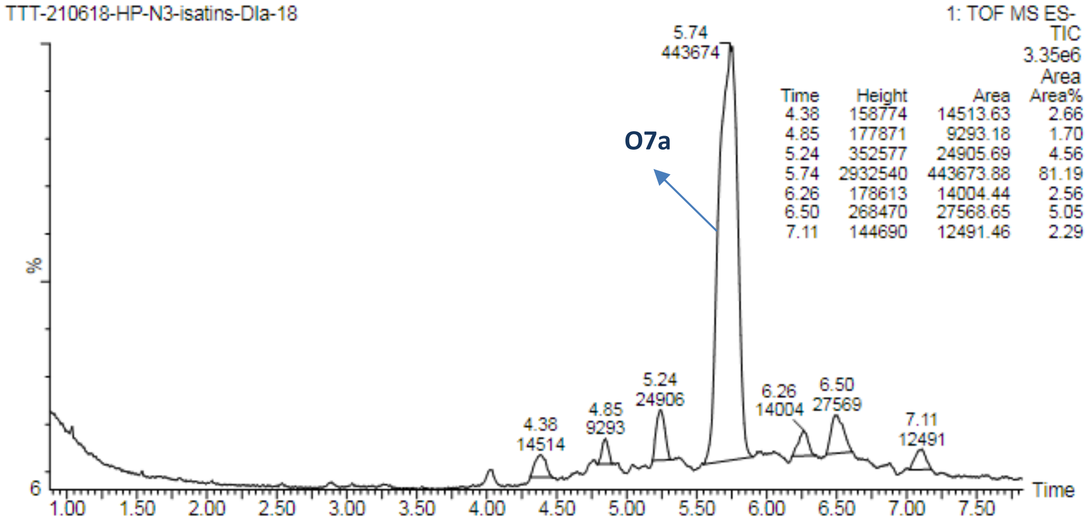

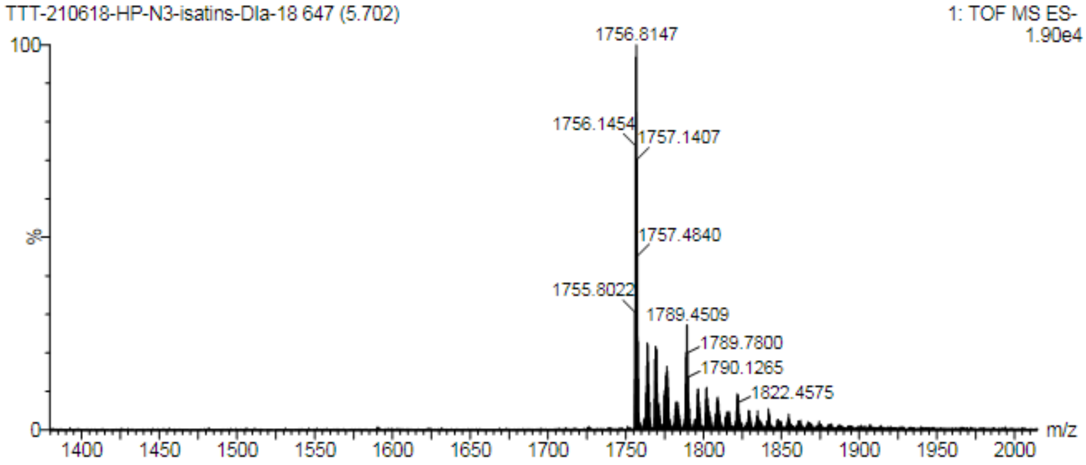

LC Trace and Mass of O7b

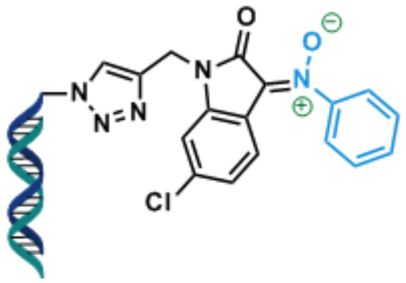

Following General Procedure vi

Yield: 76%

Exact mass: 5302.03

Triply charged mass [M]/3 - 1.00794, calculated 1766.3357; observed 1765.7842.

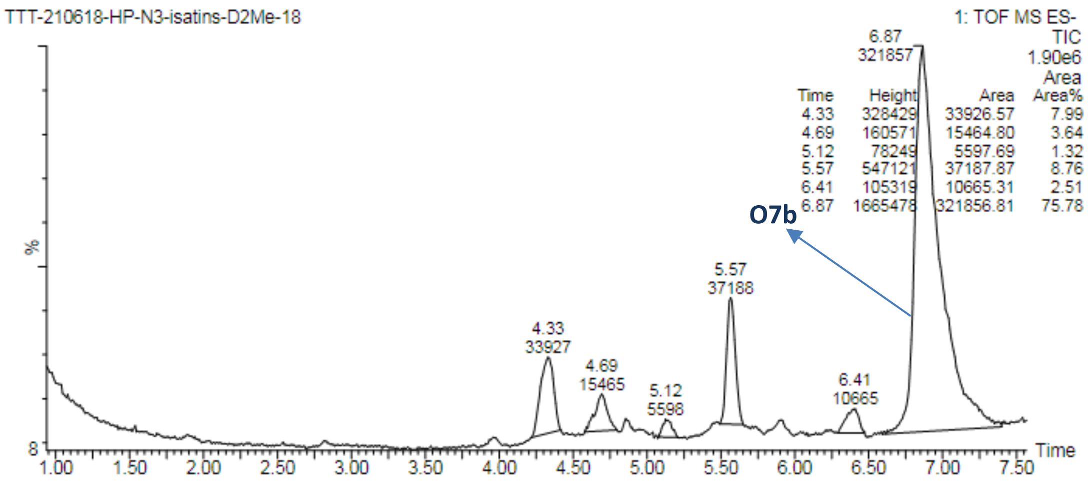

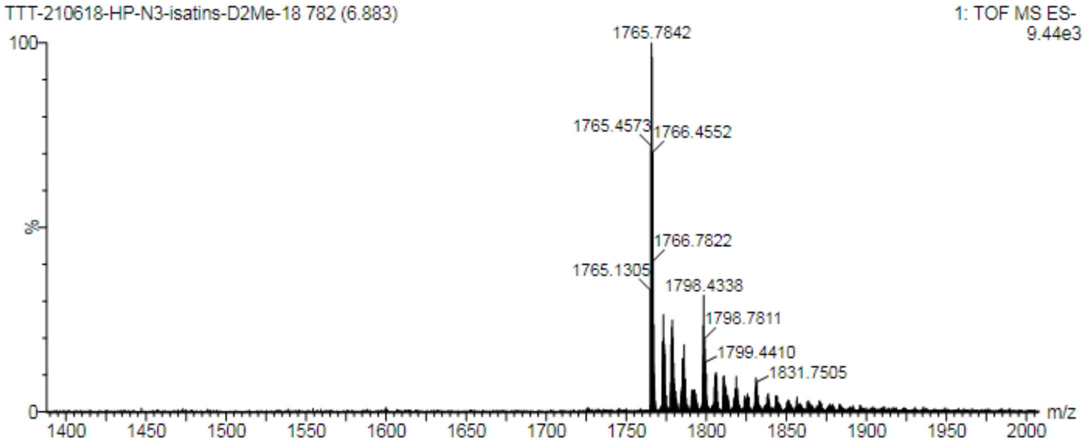

LC Trace and Mass of O7c

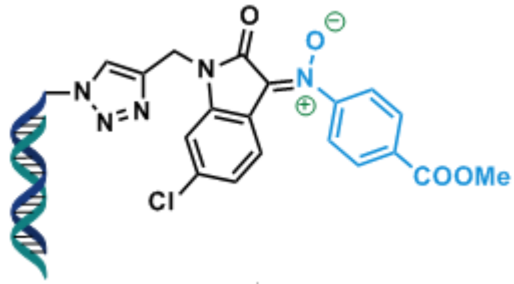

Following General Procedure vi

Yield: 77%

Exact mass: 5332.03

Triply charged mass [M]/3 - 1.00794, calculated 1776.3357; observed 1775.7599.

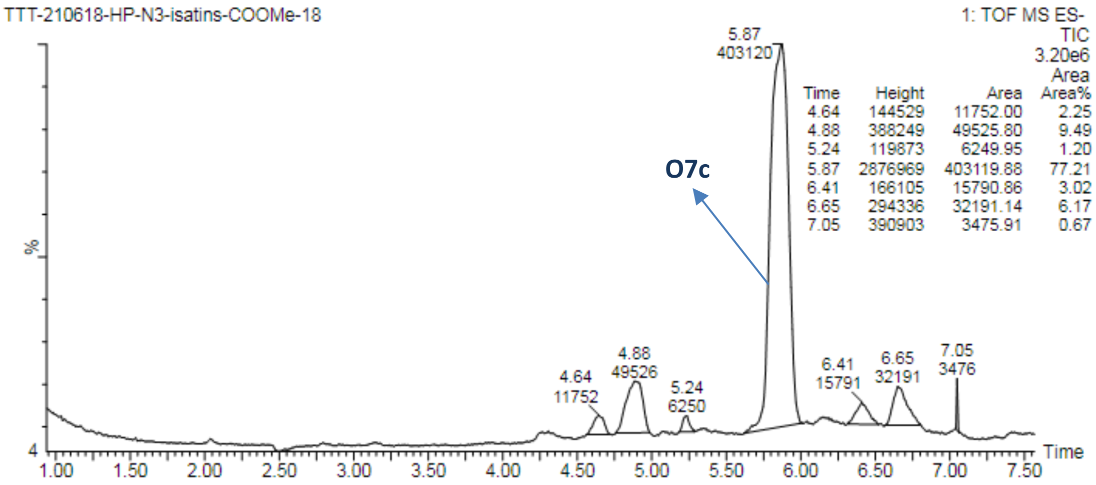

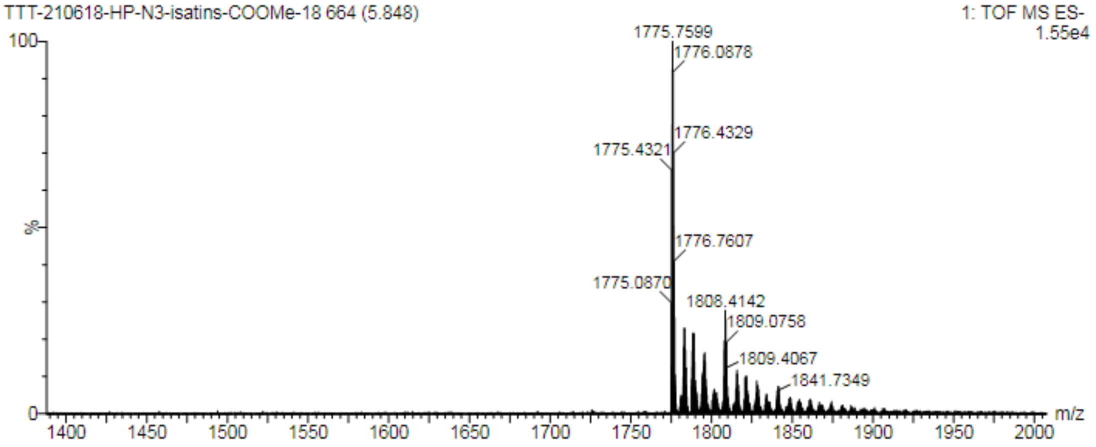

LC Trace and Mass of O8a

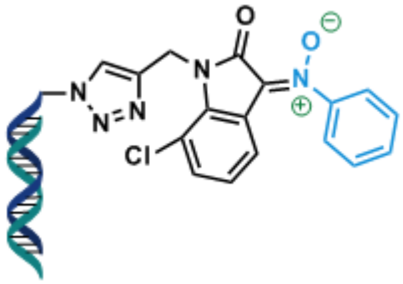

Following General Procedure vi

Yield: 89%

Exact mass: 5273.98

Triply charged mass [M]/3 - 1.00794, calculated 1756.9857; observed 1756.4714.

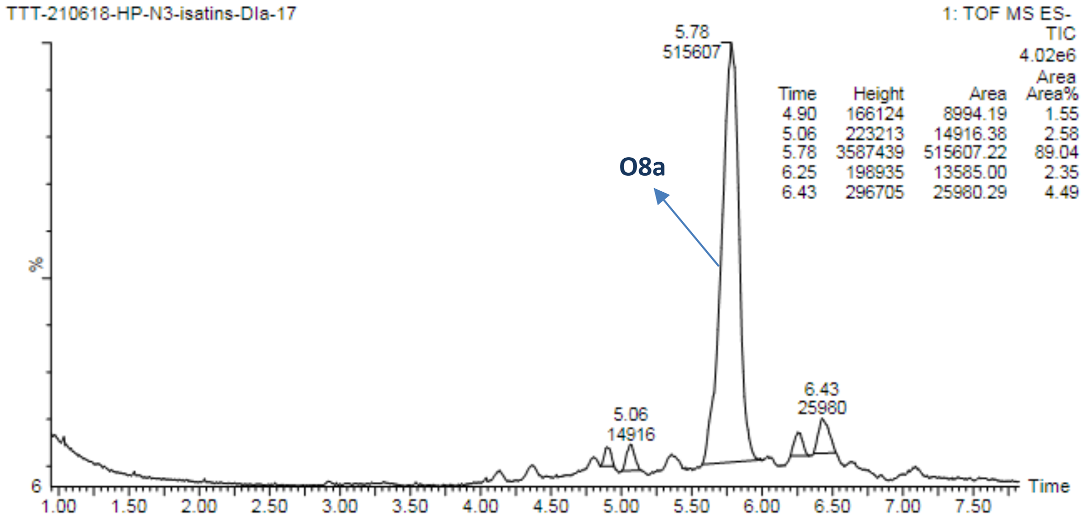

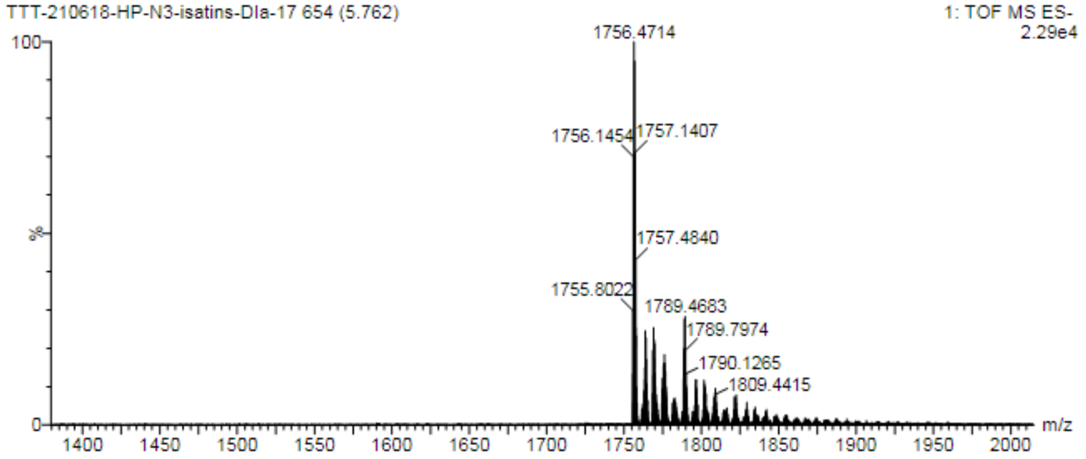

LC Trace and Mass of O8b

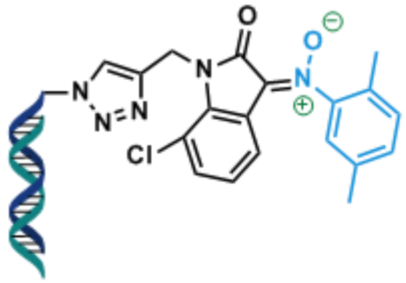

Following General Procedure vi

Yield: 80%

Exact mass: 5302.03

Triply charged mass [M]/3 - 1.00794, calculated 1766.3357; observed 1765.7842.

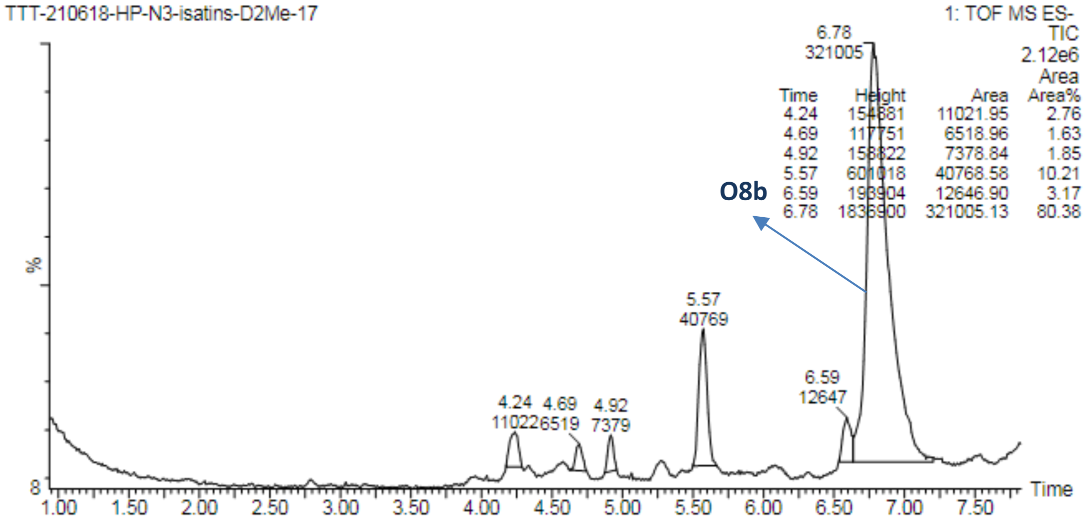

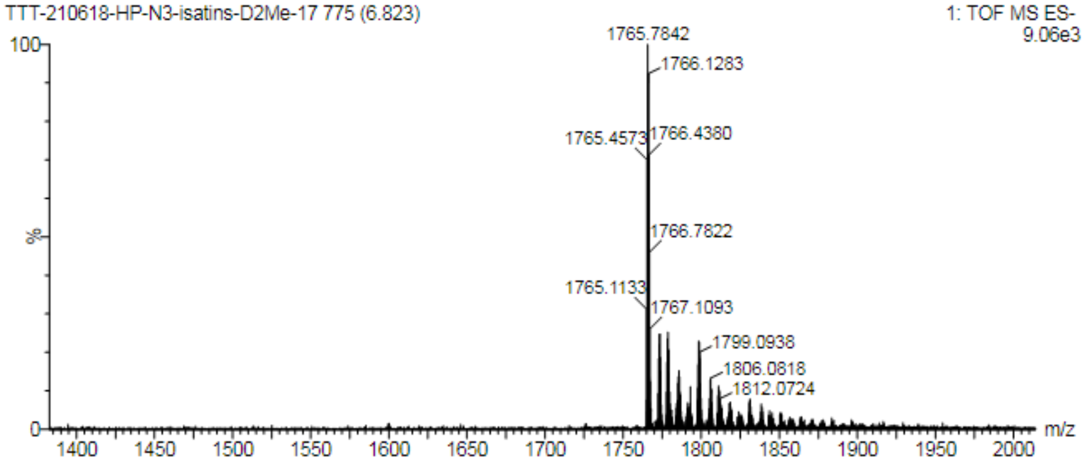

LC Trace and Mass of O8c

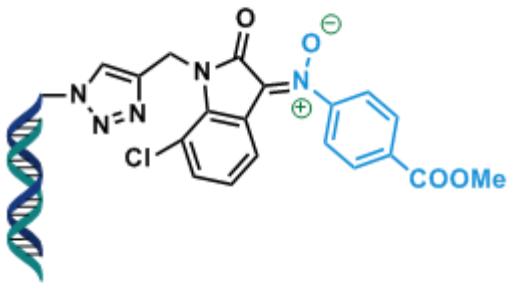

Following General Procedure vi

Yield: 82%

Exact mass: 5332.03

Triply charged mass [M]/3 - 1.00794, calculated 1776.3357; observed 1775.7599.

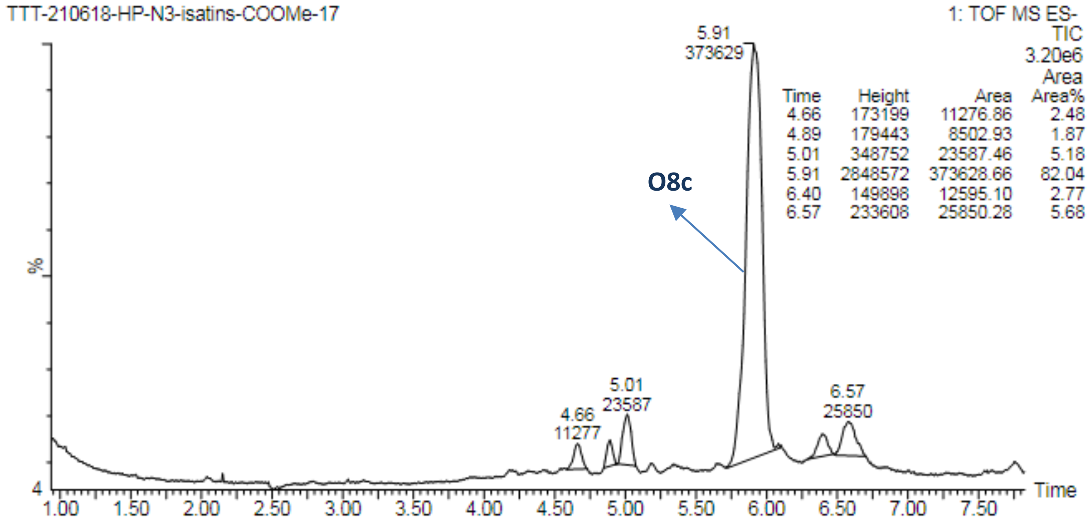

Time	Height	Area	Area%
4.66	173199	11276.86	2.48
4.89	179443	8502.93	1.87
5.01	348752	23587.46	5.18
5.91	2848572	373628.66	82.04
6.40	149898	12595.10	2.77
6.57	233608	25850.28	5.68

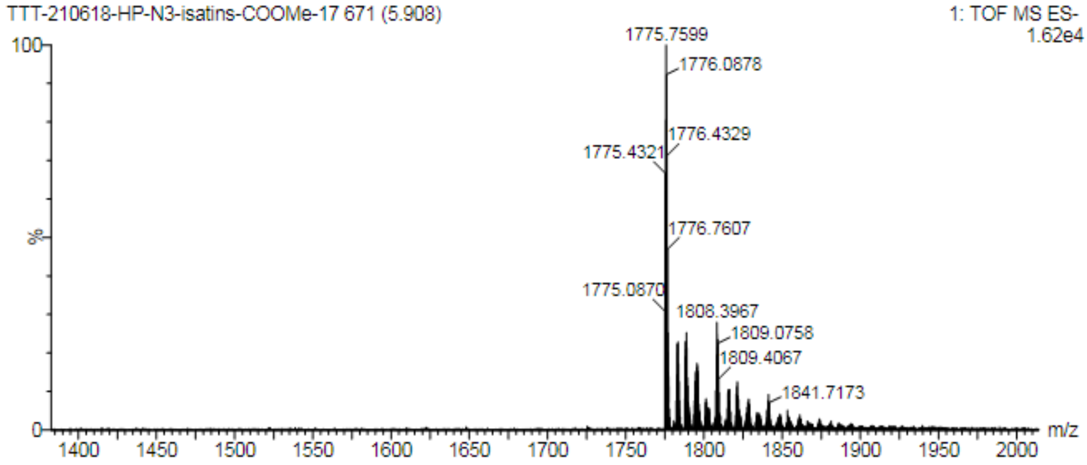

LC Trace and Mass of O9a

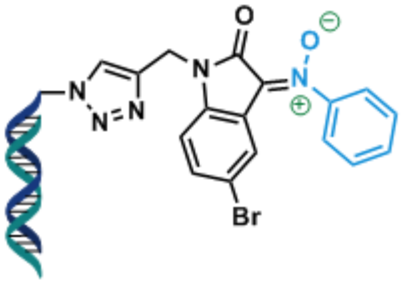

Following General Procedure vi
Yield: 76%
Exact mass: 5318.431
Triply charged mass [M]/3 - 1.00794, calculated 1771.8024; observed 1771.4491.

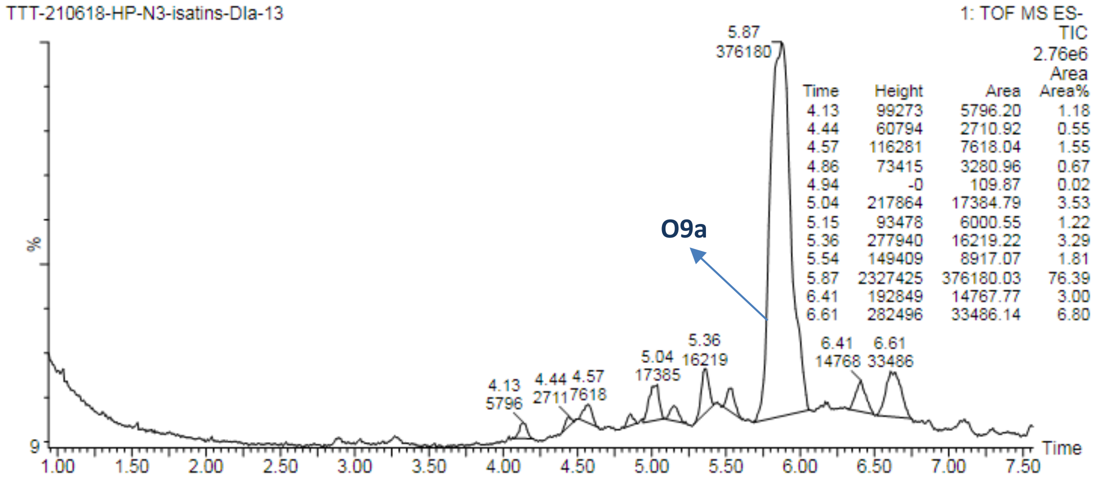

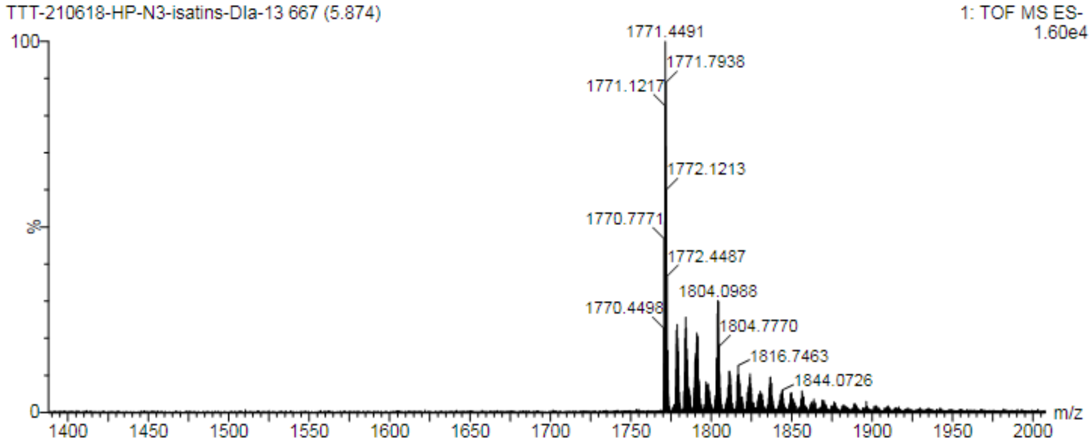

LC Trace and Mass of O9b

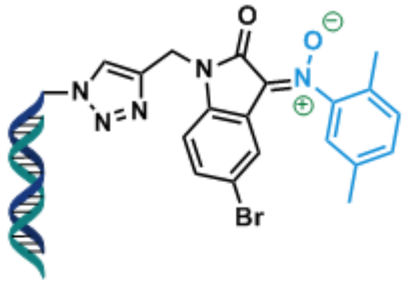

Following General Procedure vi
Yield: 75%
Exact mass: 5346.481
Triply charged mass [M]/3 - 1.00794, calculated 1781.1524; observed 1780.7496.

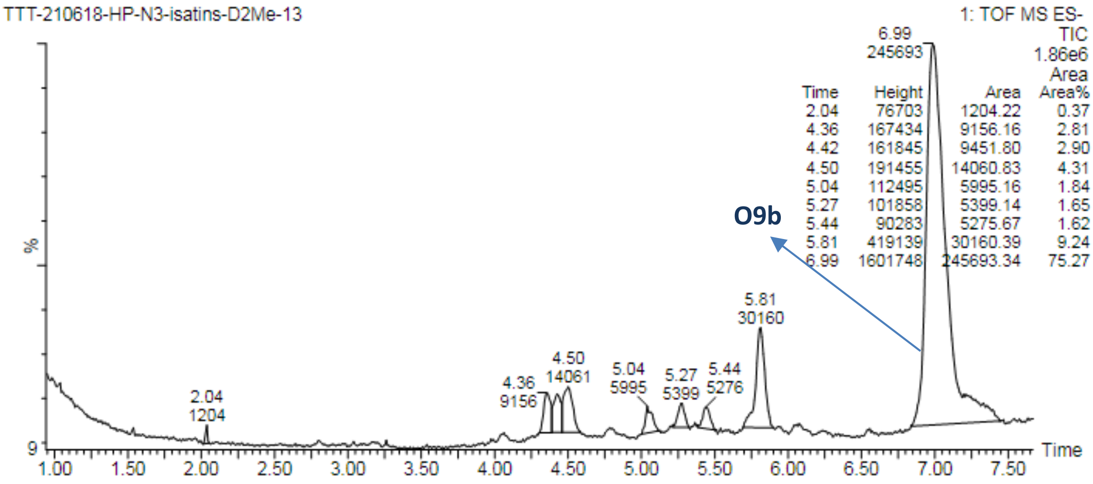

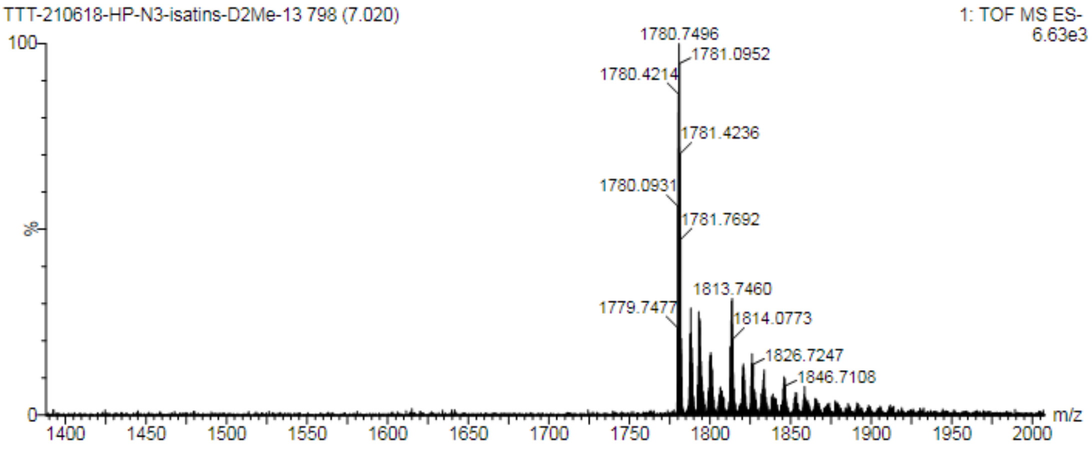

LC Trace and Mass of O9c

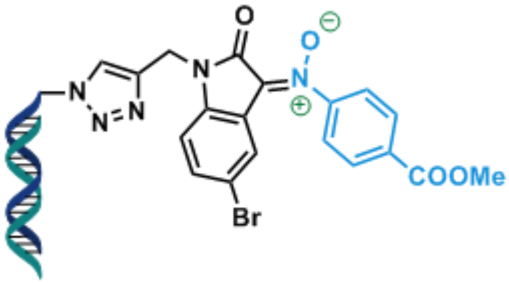

Following General Procedure vi

Yield: 80%

Exact mass: 5376.48

Triply charged mass [M]/3 - 1.00794, calculated 1791.1524; observed 1790.9927.

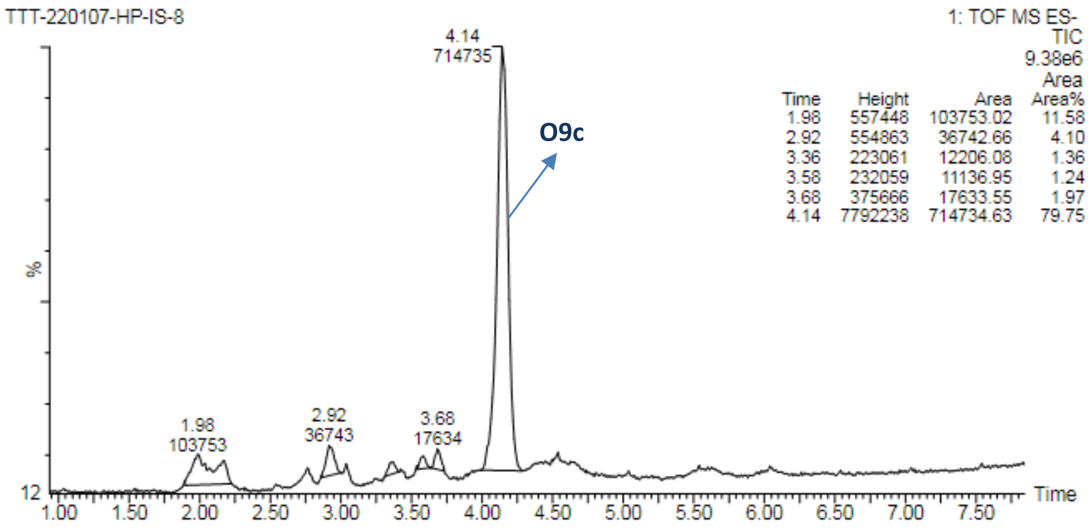

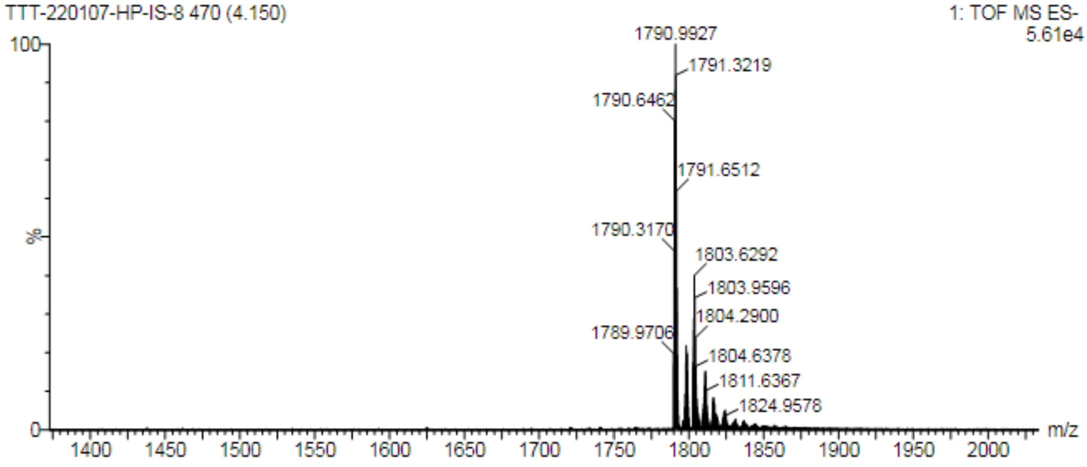

LC Trace and Mass of O10a

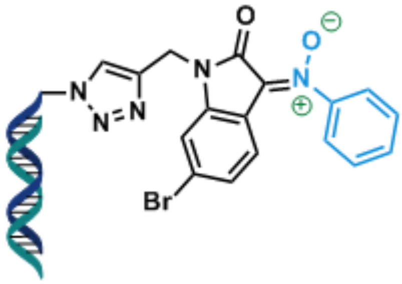

Following General Procedure vi

Yield: 65%

Exact mass: 5318.431

Triply charged mass [M]/3 - 1.00794, calculated 1771.8024; observed 1771.4491.

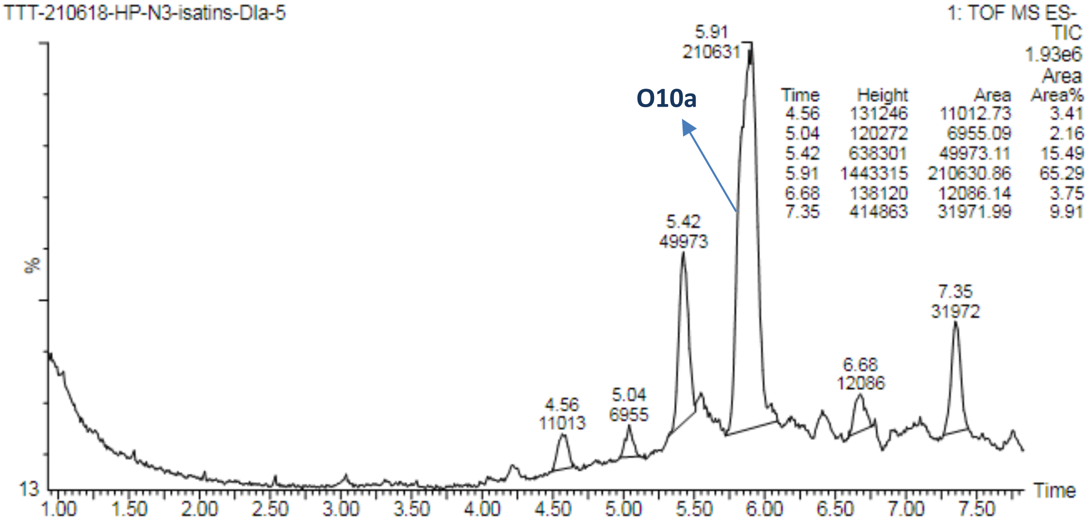

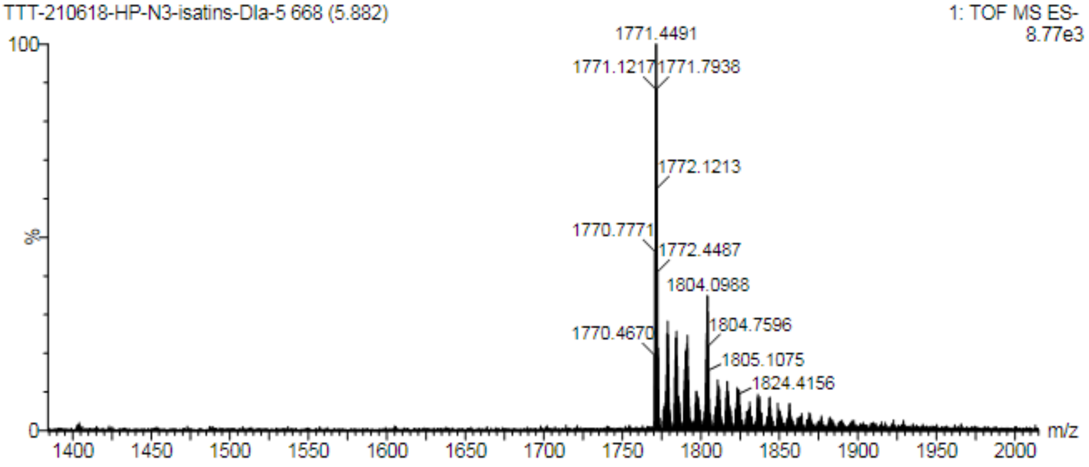

LC Trace and Mass of O10b

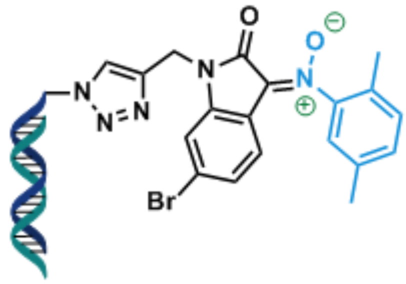

Following General Procedure vi
Yield: 82%
Exact mass: 5346.481
Triply charged mass [M]/3 - 1.00794, calculated 1781.1524; observed 1780.7670.

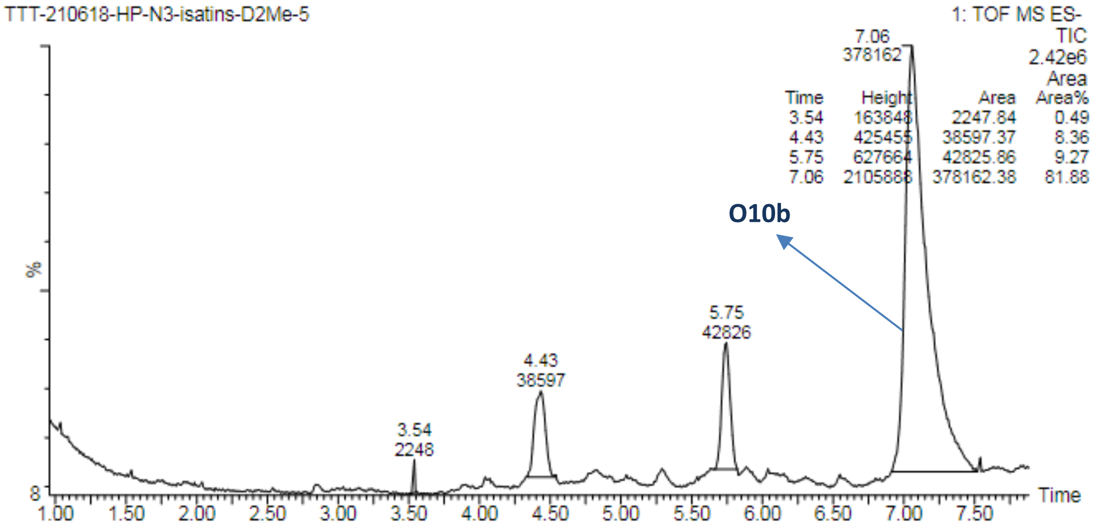

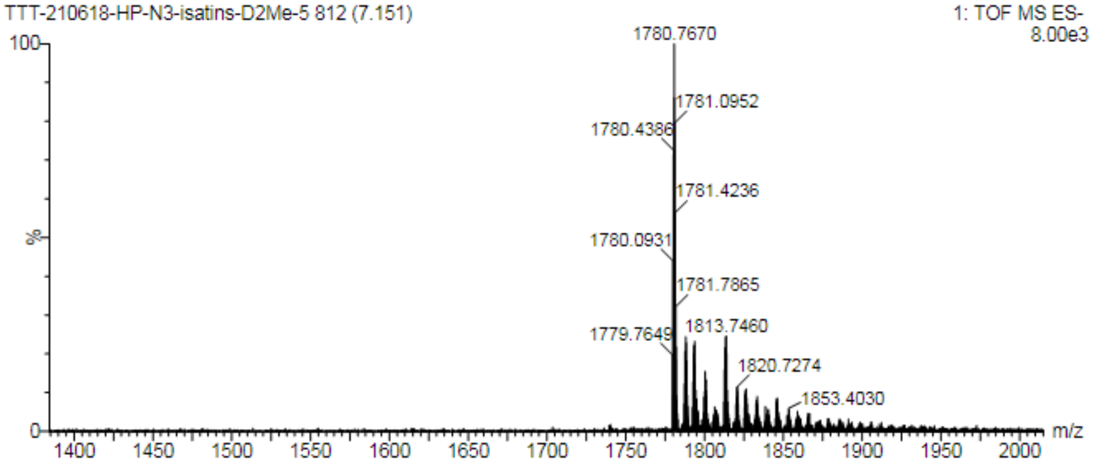

LC Trace and Mass of O10c

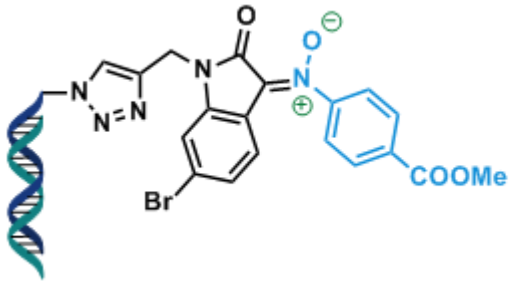

Following General Procedure vi

Yield: 65%

Exact mass: 5376.48

Triply charged mass [M]/3 - 1.00794, calculated 1791.1524; observed 1790.7501.

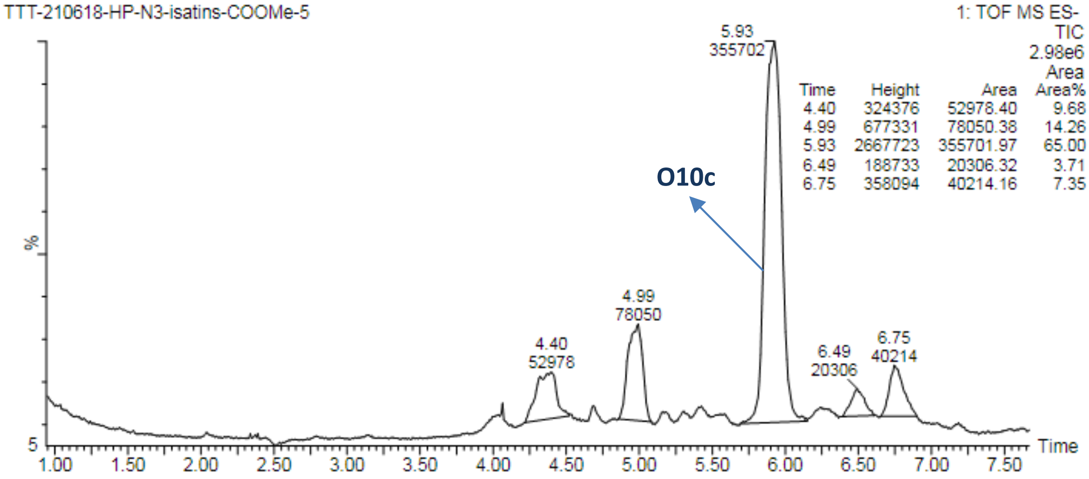

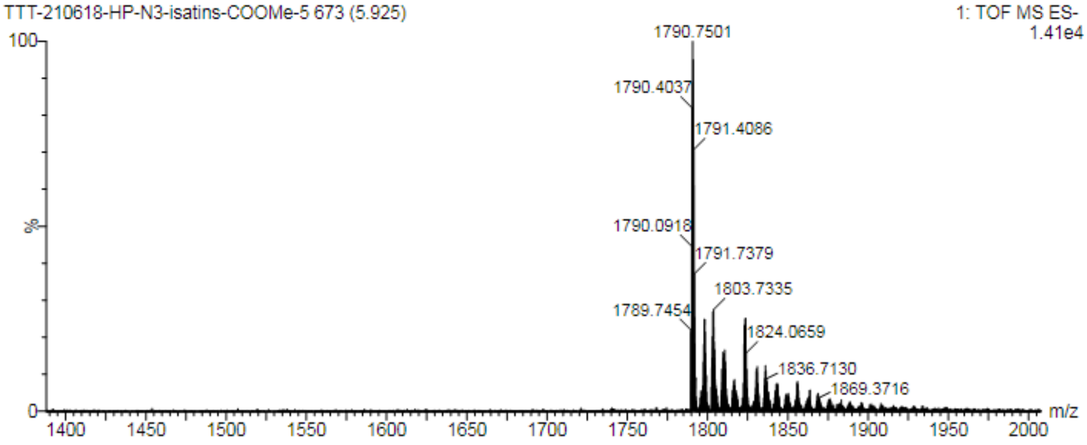

LC Trace and Mass of O11a

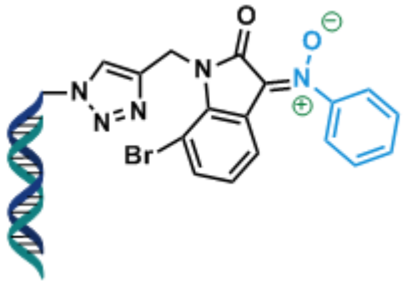

Following General Procedure vi

Yield: 73%

Exact mass: 5318.431

Triply charged mass [M]/3 - 1.00794, calculated 1771.8024; observed 1771.4491.

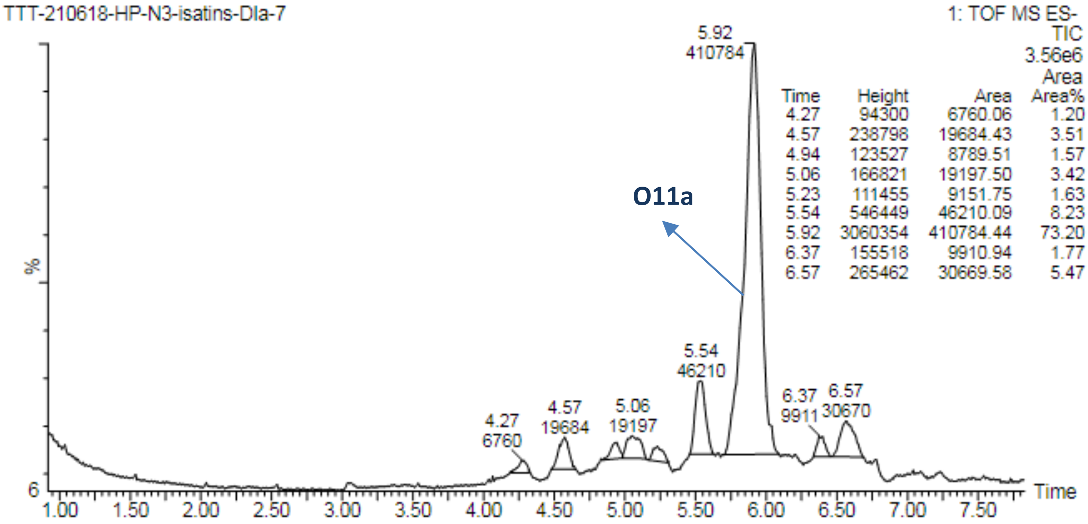

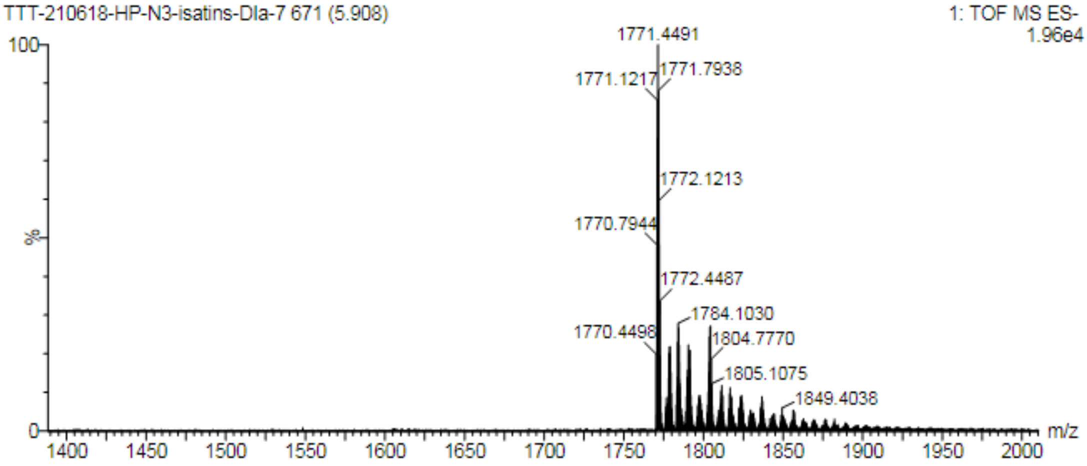

LC Trace and Mass of O11b

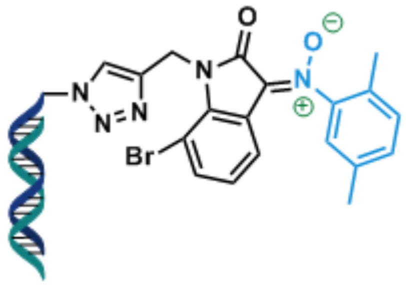

Following General Procedure vi

Yield: 82%

Exact mass: 5346.481

Triply charged mass [M]/3 - 1.00794, calculated 1781.1524; observed 1780.7670.

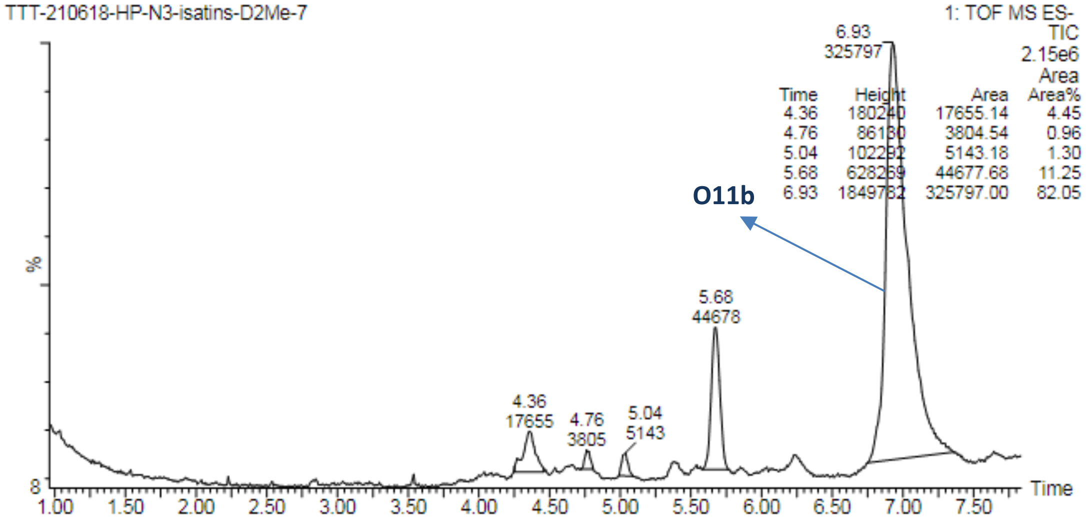

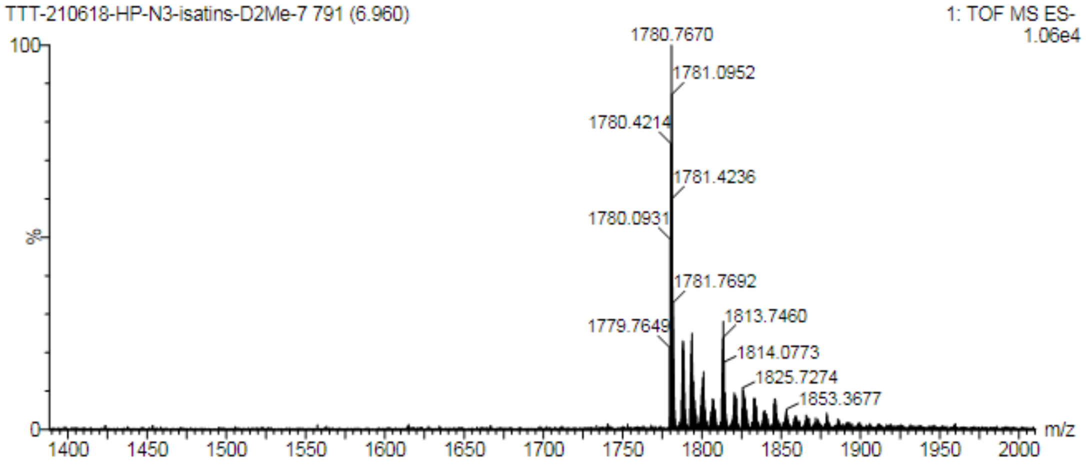

LC Trace and Mass of O11c

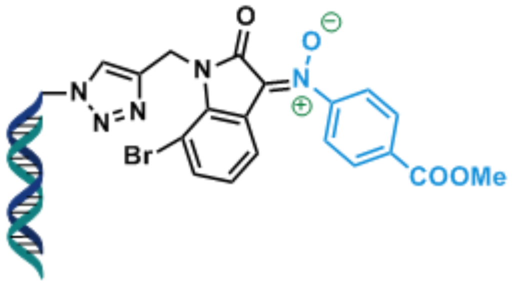

Following General Procedure vi
Yield: 83%
Exact mass: 5376.48
Triply charged mass [M]/3 - 1.00794, calculated 1791.1524; observed 1790.9927.

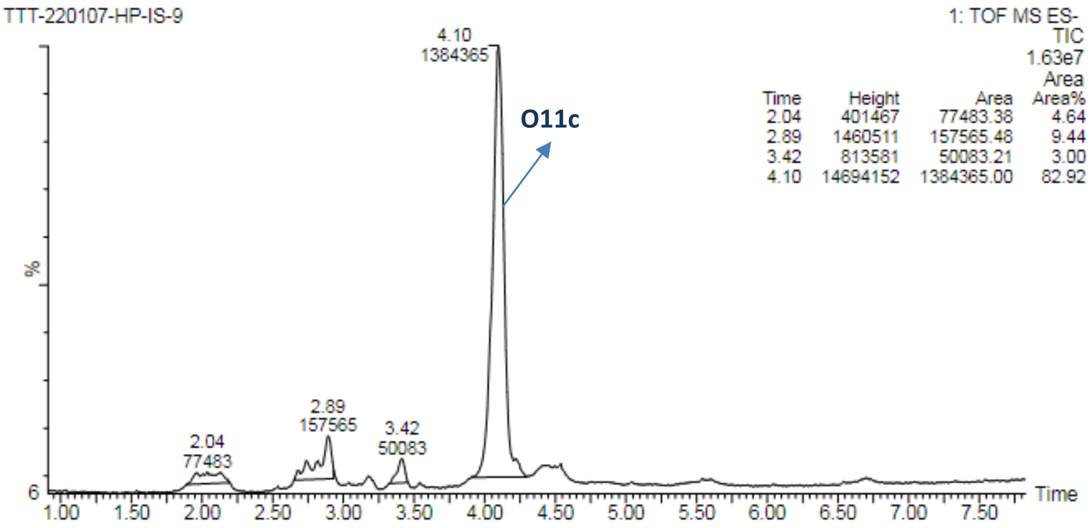

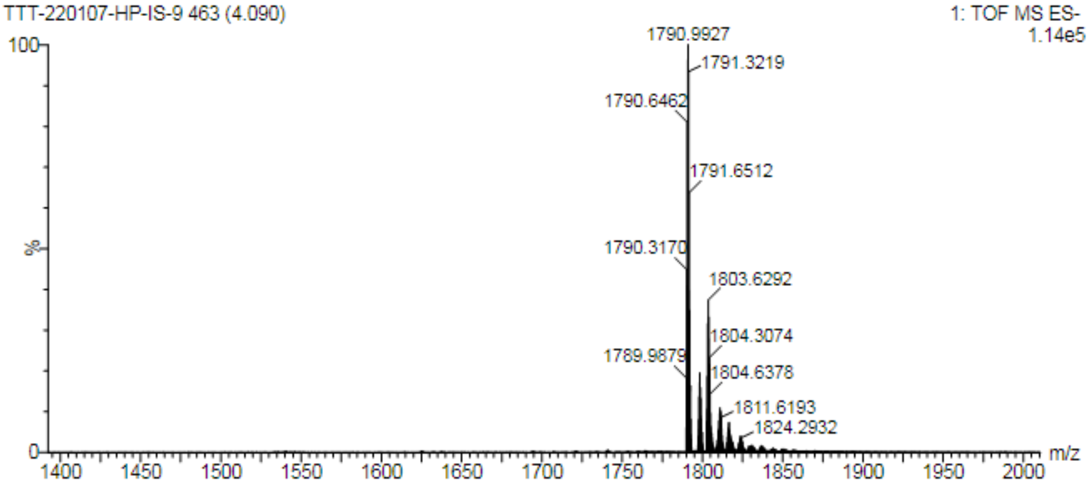

LC Trace and Mass of O12a

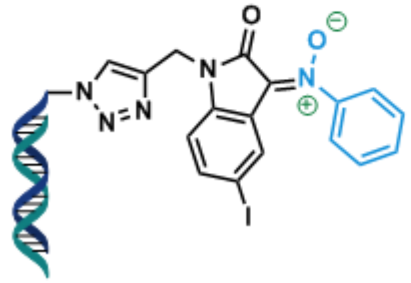

Following General Procedure vi
Yield: 66%
Exact mass: 5365.43
Triply charged mass [M]/3 - 1.00794, calculated 1787.4691; observed 1787.2866.

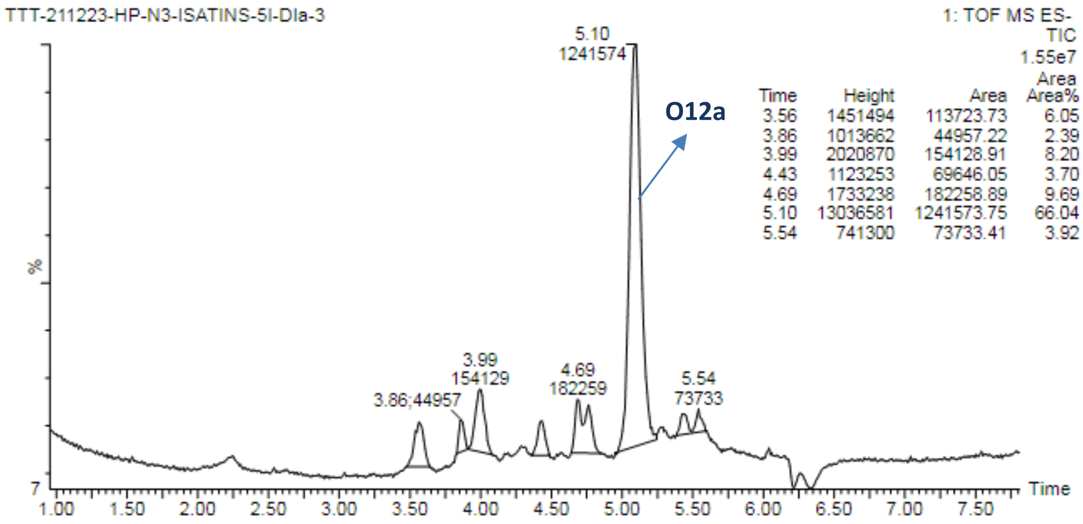

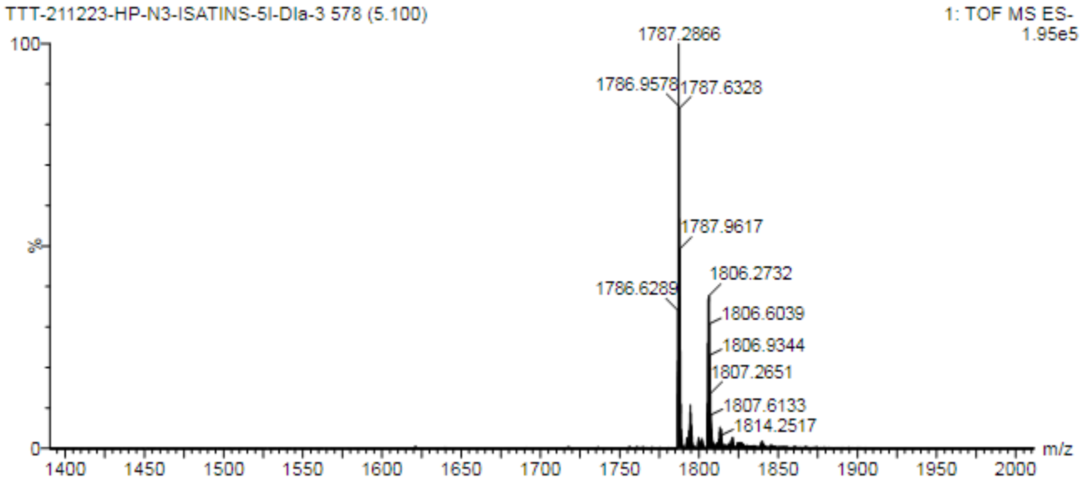

LC Trace and Mass of O12b

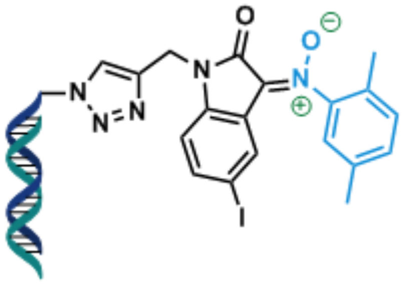

Following General Procedure vi

Yield: 70%

Exact mass: 5393.48

Triply charged mass [M]/3 - 1.00794, calculated 1796.8191; observed 1796.4203.

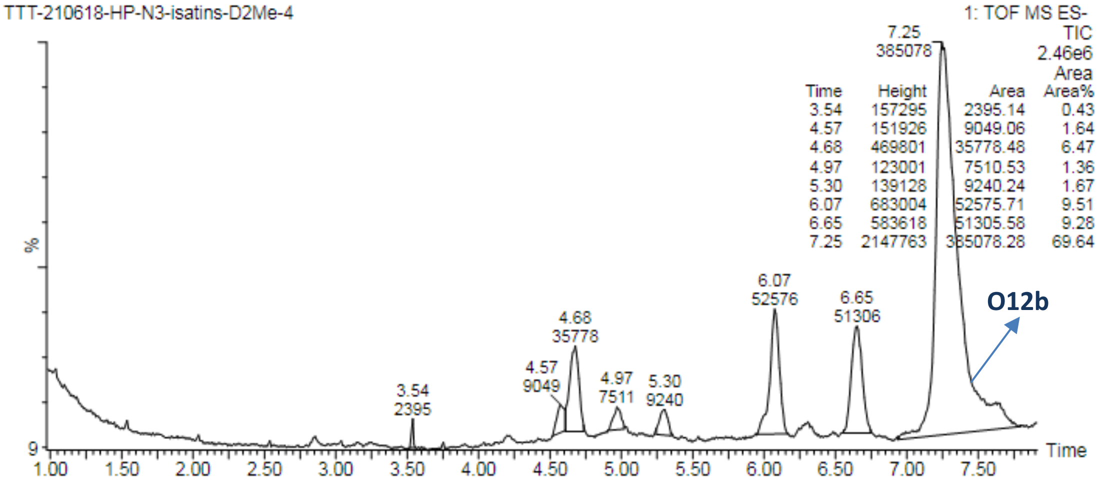

Time	Height	Area	Area%
3.54	157295	2395.14	0.43
4.57	151926	9049.06	1.64
4.68	469801	35778.48	6.47
4.97	123001	7510.53	1.36
5.30	139128	9240.24	1.67
6.07	683004	52575.71	9.51
6.65	583618	51305.58	9.28
7.25	2147763	385078.28	69.64

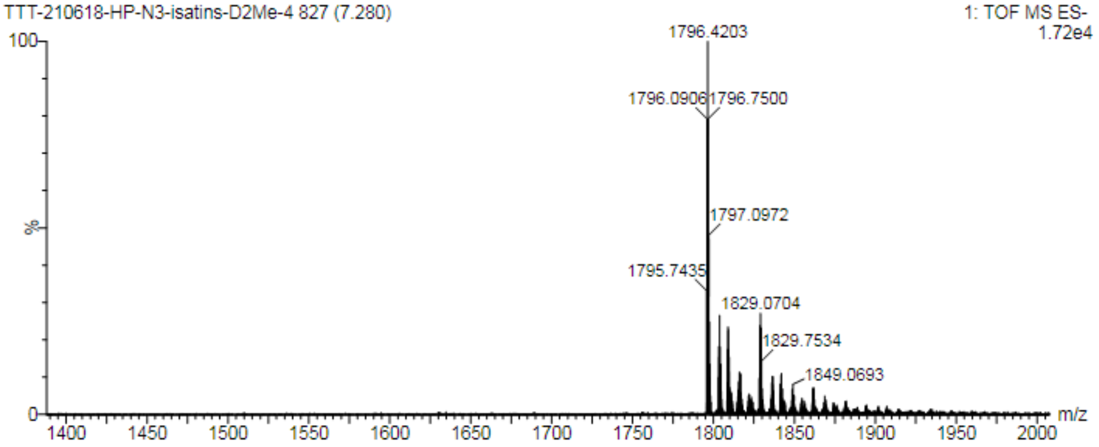

LC Trace and Mass of O12c

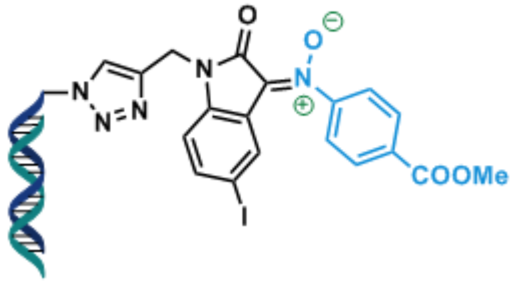

Following General Procedure vi

Yield: 73%

Exact mass: 5423.48

Triply charged mass [M]/3 - 1.00794, calculated 1806.8191; observed 1806.6212.

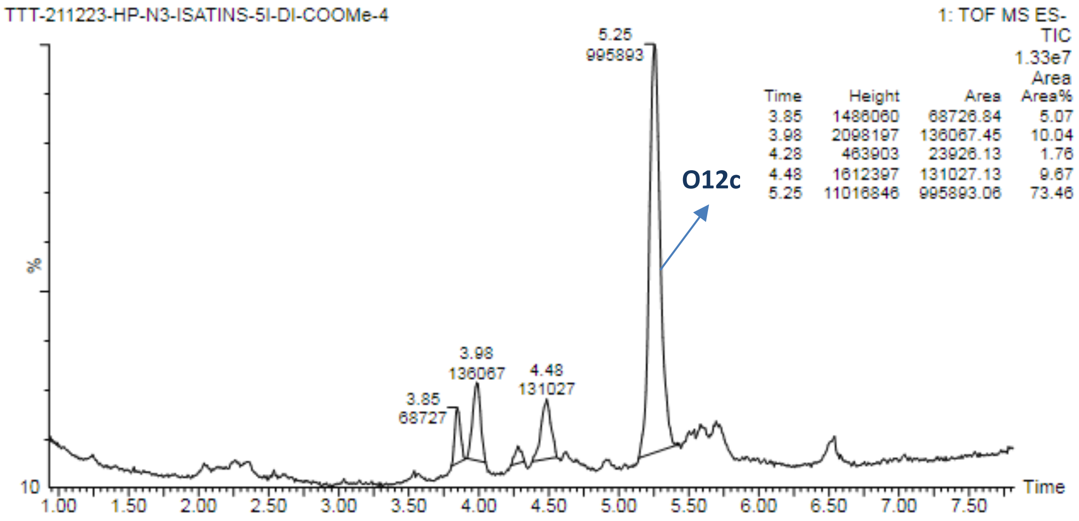

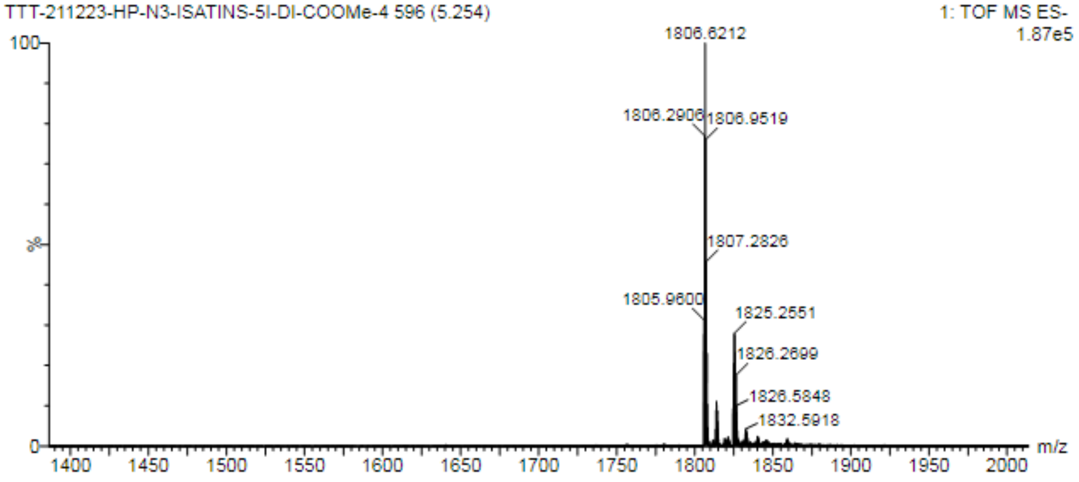

LC Trace and Mass of O13a

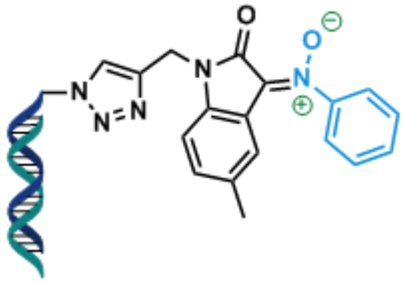

Following General Procedure vi

Yield: 87%

Exact mass: 5253.56

Triply charged mass [M]/3 - 1.00794, calculated 1750.1791; observed 1749.4945.

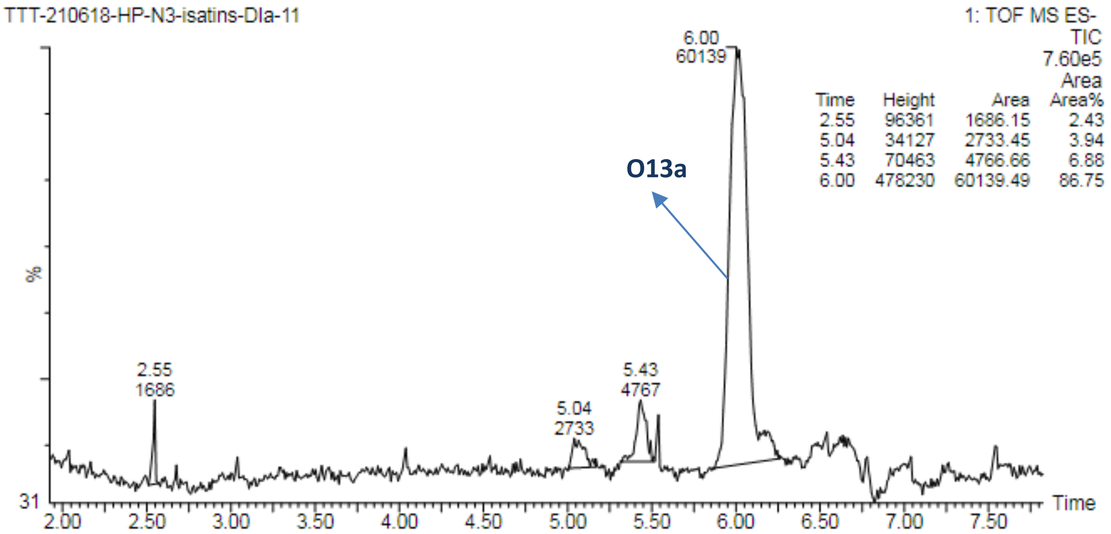

Time	Height	Area	Area%
2.55	96361	1686.15	2.43
5.04	34127	2733.45	3.94
5.43	70463	4766.66	6.88
6.00	478230	60139.49	86.75

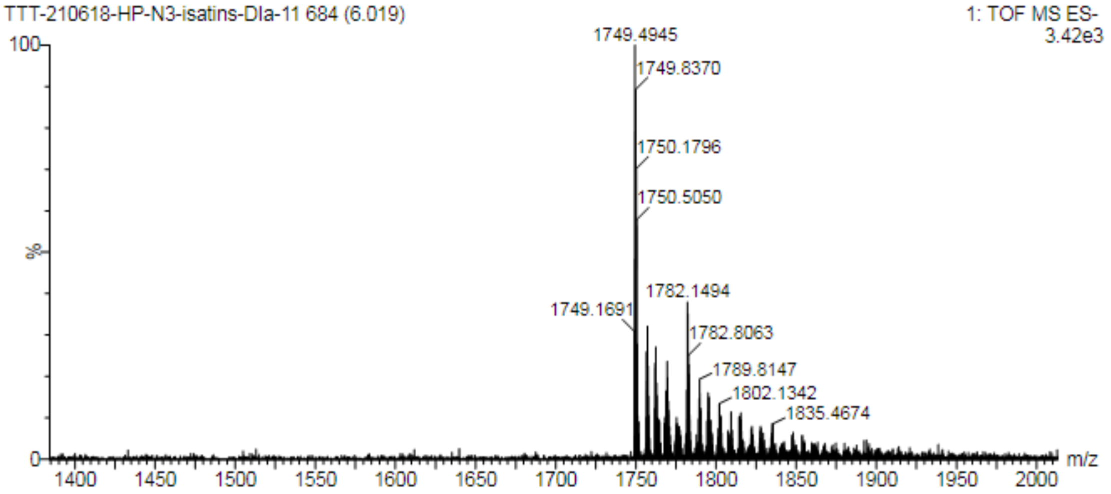

LC Trace and Mass of O13b

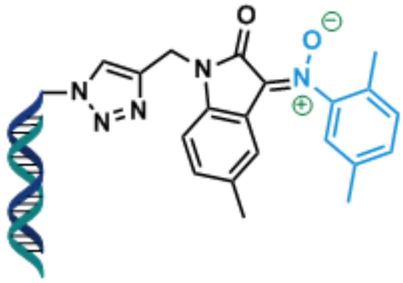

Following General Procedure vi

Yield: 83%

Exact mass: 5281.611

Triply charged mass [M]/3 - 1.00794, calculated 1759.5291; observed 1759.3555.

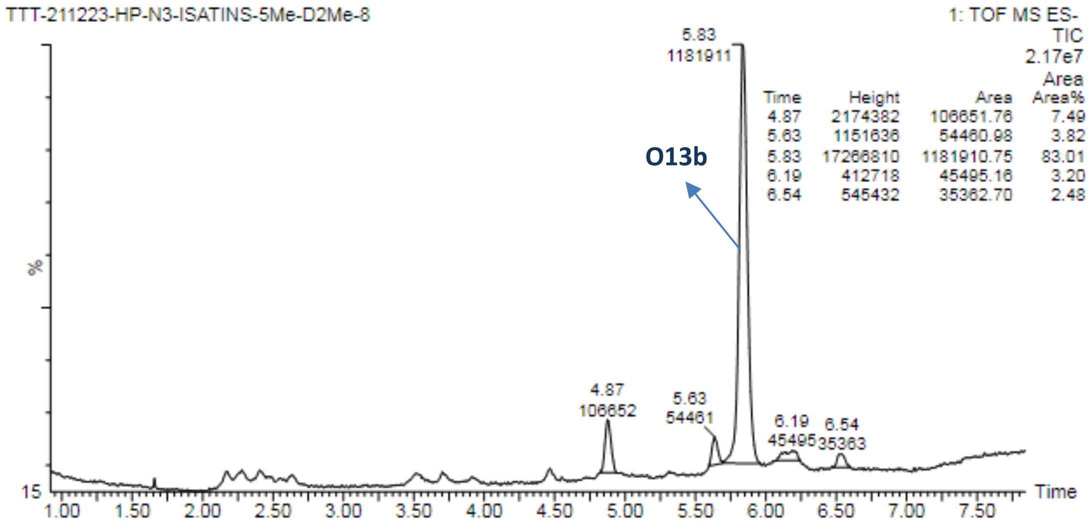

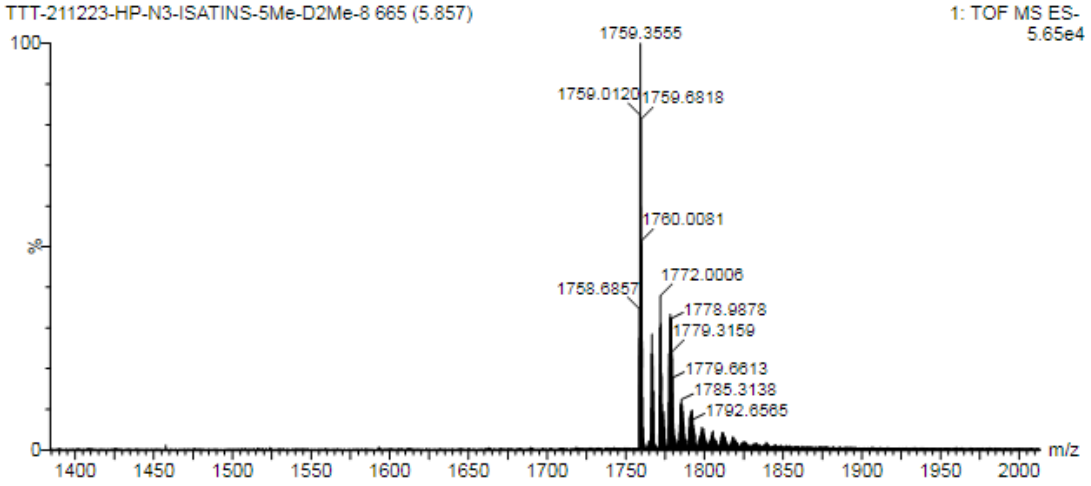

LC Trace and Mass of O13c

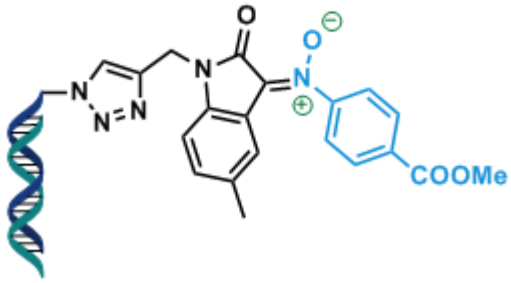

Following General Procedure vi

Yield: 89%

Exact mass: 5311.61

Triply charged mass [M]/3 - 1.00794, calculated 1769.5291; observed 1769.1063.

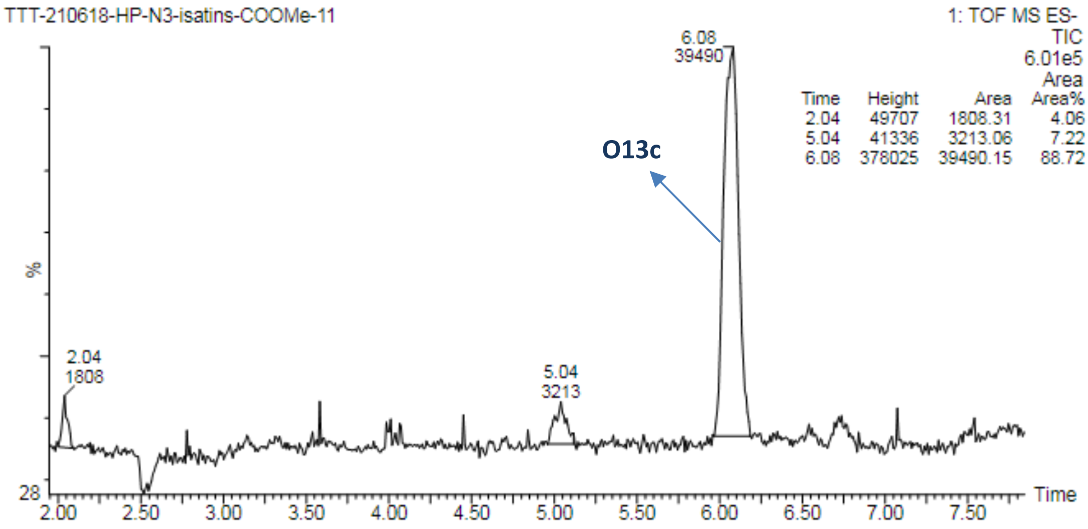

Time	Height	Area	Area%
2.04	49707	1808.31	4.06
5.04	41336	3213.06	7.22
6.08	378025	39490.15	88.72

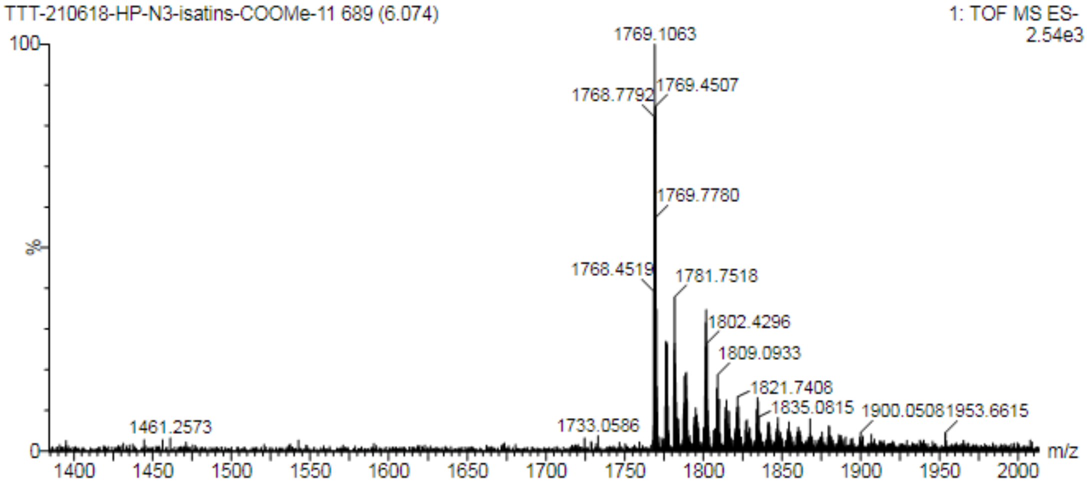

LC Trace and Mass of O14a

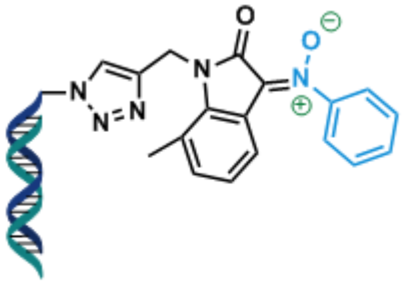

Following General Procedure vi

Yield: 79%

Exact mass: 5253.56

Triply charged mass [M]/3 - 1.00794, calculated 1750.1791; observed 1749.8198.

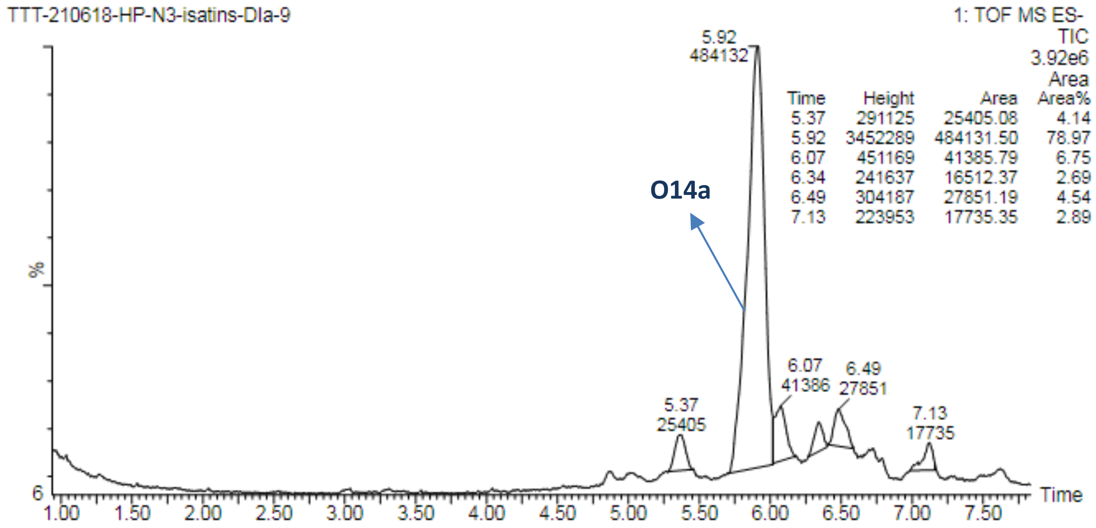

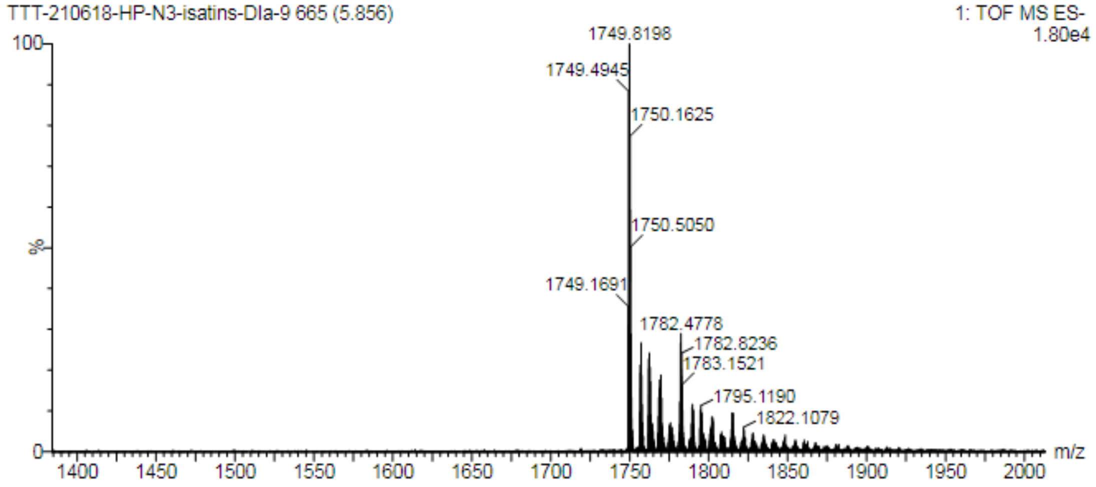

LC Trace and Mass of O14b

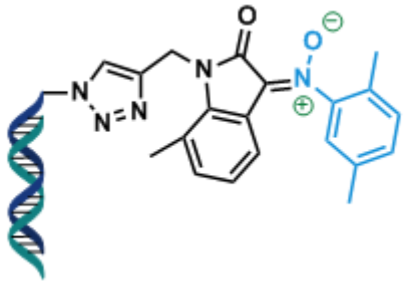

Following General Procedure vi

Yield: 75%

Exact mass: 5281.611

Triply charged mass [M]/3 - 1.00794, calculated 1759.5291; observed 1759.1322.

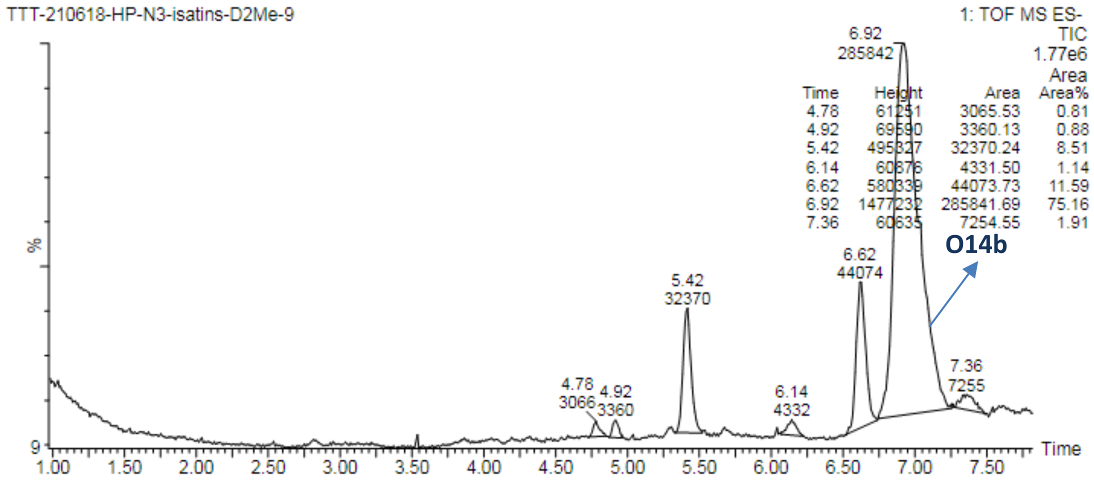

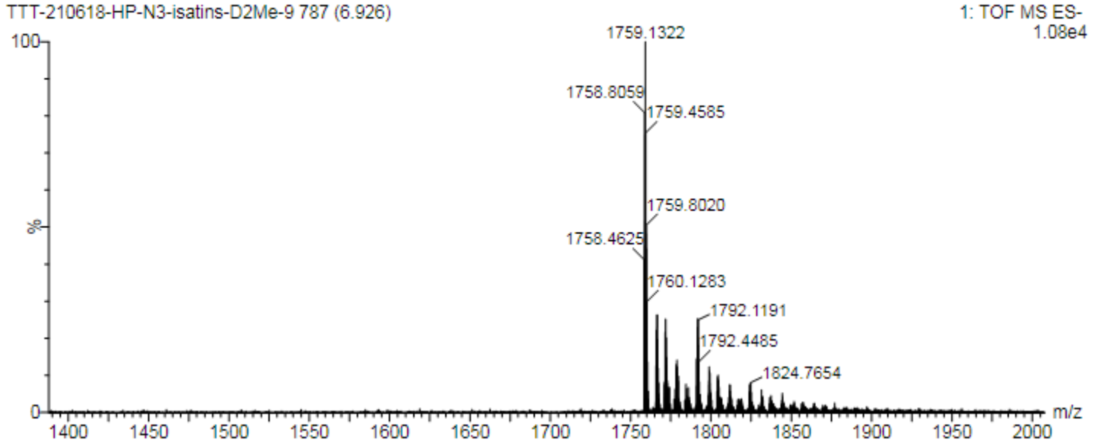

LC Trace and Mass of O14c

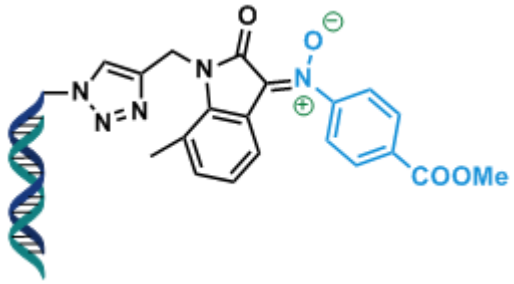

Following General Procedur vi

Yield: 82%

Exact mass: 5311.61

Triply charged mass [M]/3 - 1.00794, calculated 1769.5291; observed 1769.3646.

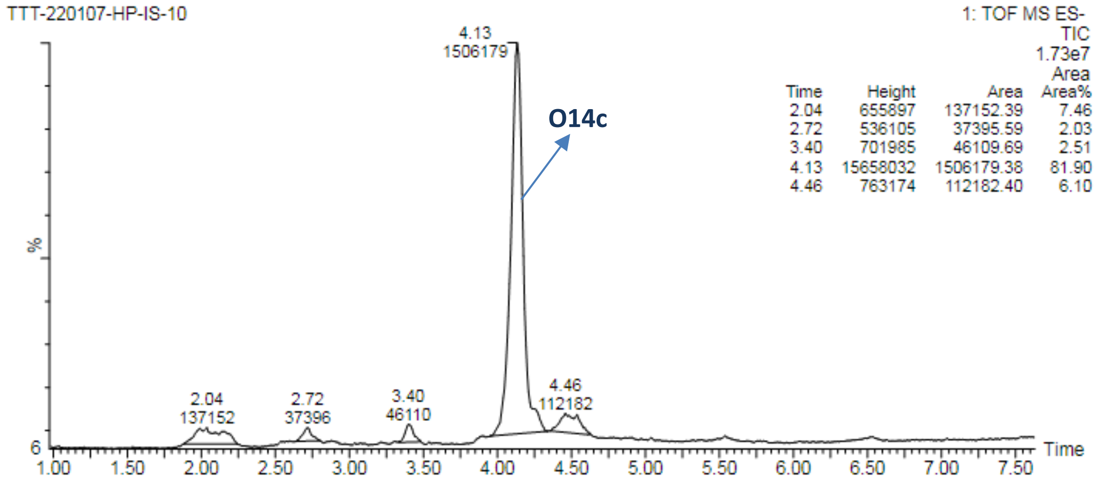

Time	Height	Area	Area%
2.04	655897	137152.39	7.46
2.72	536105	37395.59	2.03
3.40	701985	46109.69	2.51
4.13	15658032	1506179.38	81.90
4.46	763174	112182.40	6.10

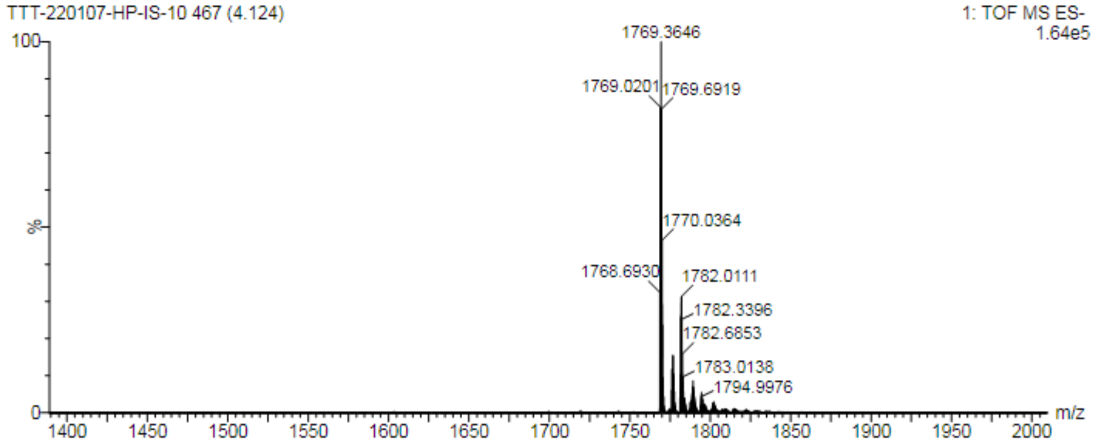

LC Trace and Mass of O15a

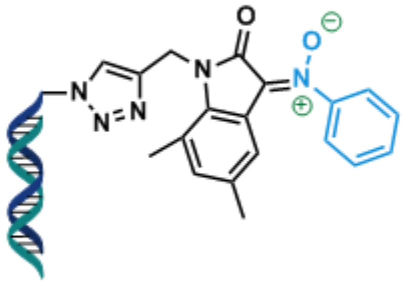

Following General Procedure vi

Yield: 82%

Exact mass: 5267.59

Triply charged mass [M]/3 - 1.00794, calculated 1754.8557; observed 1754.4987.

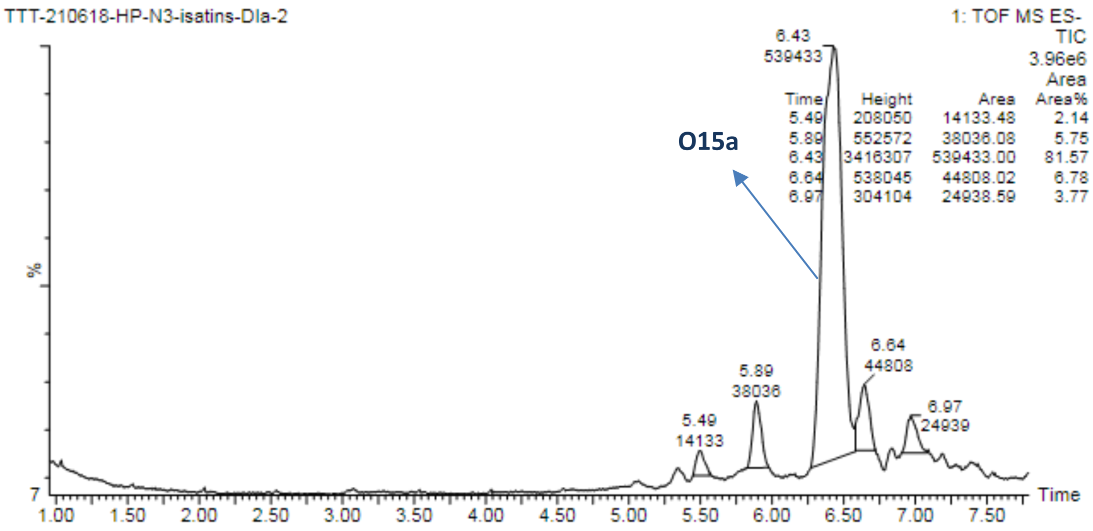

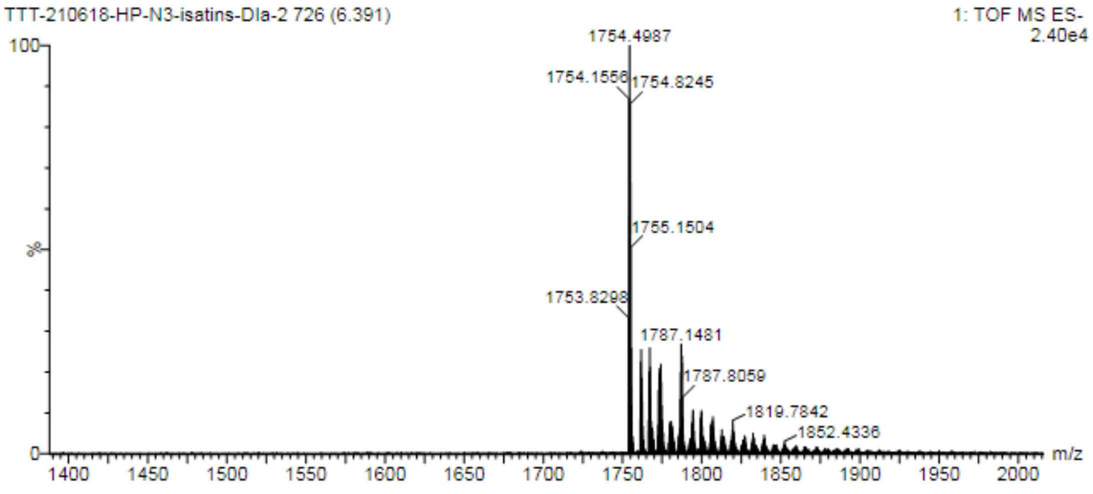

LC Trace and Mass of O15b

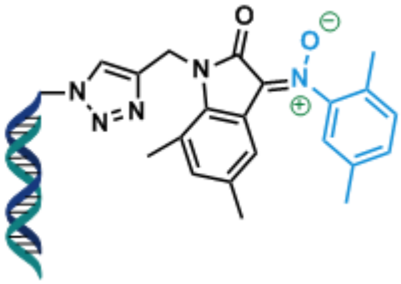

Following General Procedure vi

Yield: 87%

Exact mass: 5295.64

Triply charged mass [M]/3 - 1.00794, calculated 1764.2057; observed 1763.8062.

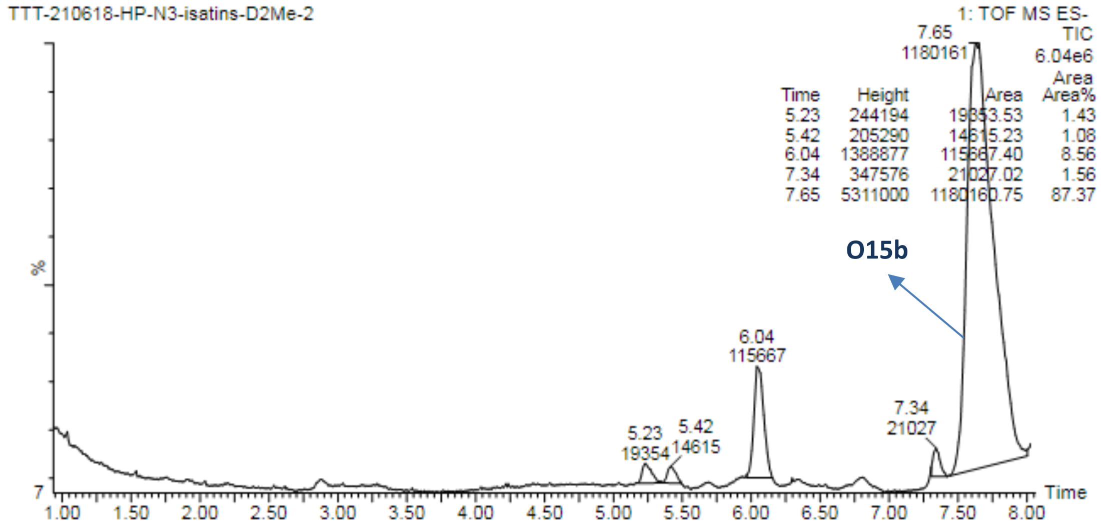

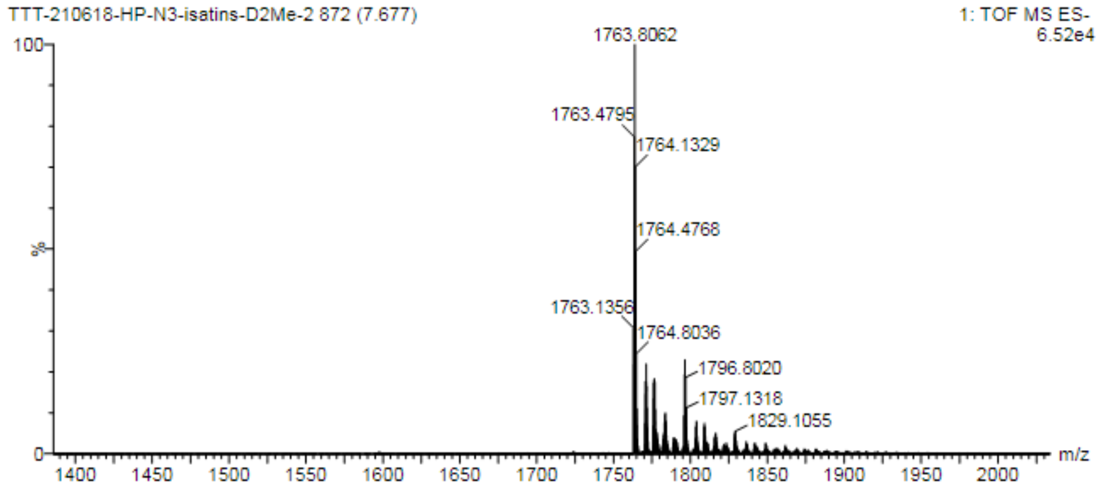

LC Trace and Mass of O15c

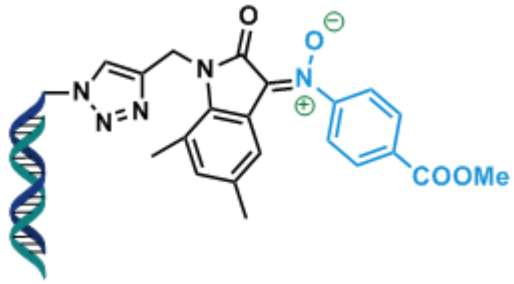

Following General Procedure vi

Yield: 89%

Exact mass: 5325.64

Triply charged mass [M]/3 - 1.00794, calculated 1774.2057; observed 1774.0349.

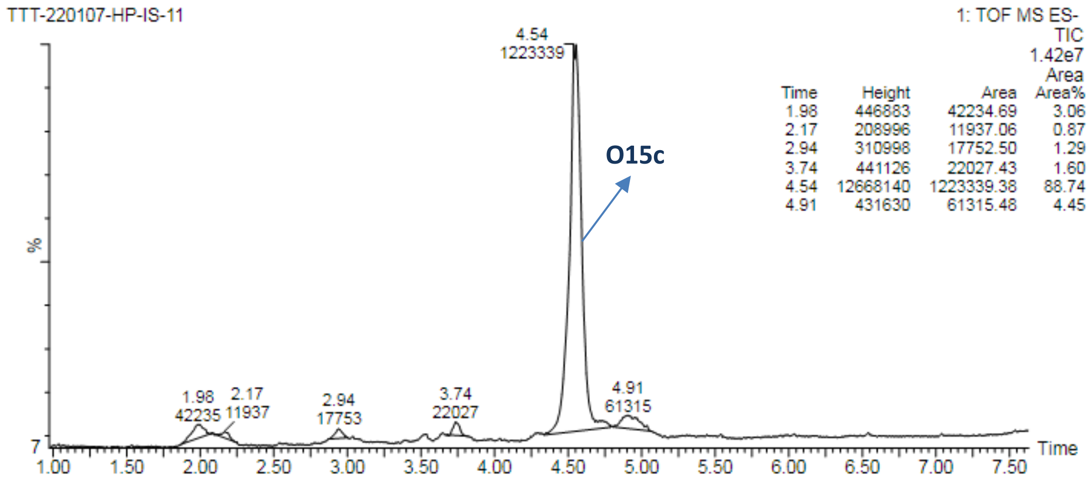

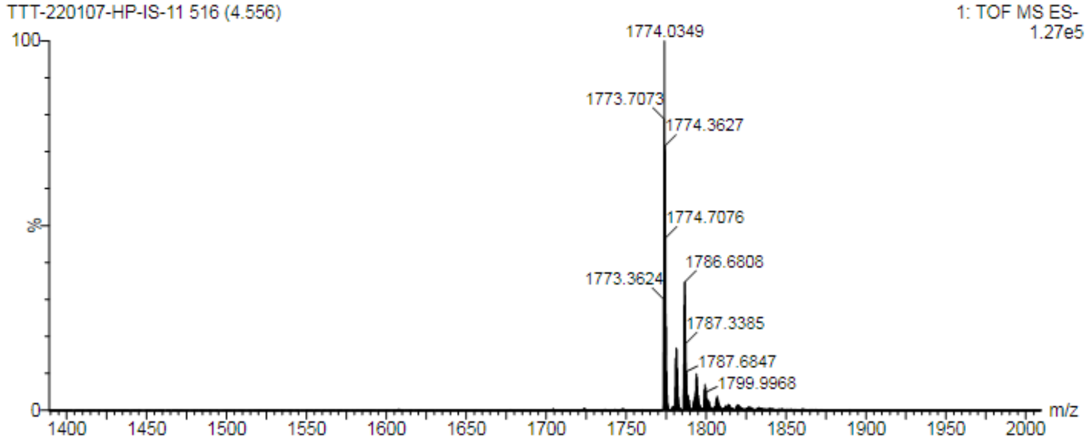

LC Trace and Mass of O16a

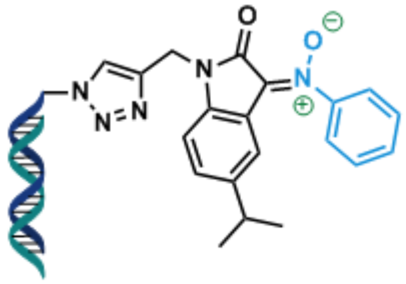

Following General Procedure vi
Yield: 86%
Exact mass: 5281.61
Triply charged mass [M]/3 - 1.00794, calculated 1759.5291; observed 1759.3555.

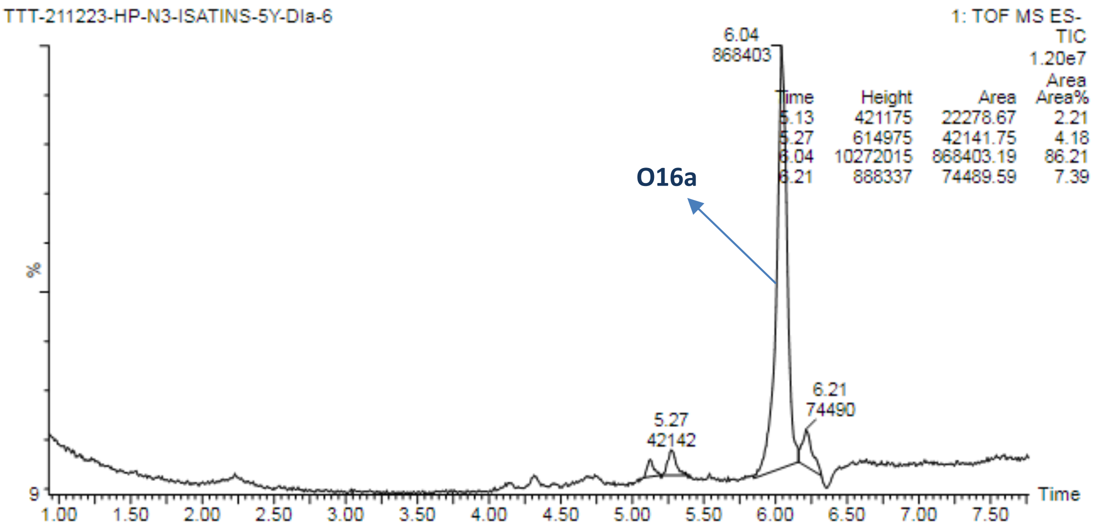

Time	Height	Area	Area%
5.13	421175	22278.67	2.21
5.27	614975	42141.75	4.18
6.04	10272015	868403.19	86.21
6.21	888337	74489.59	7.39

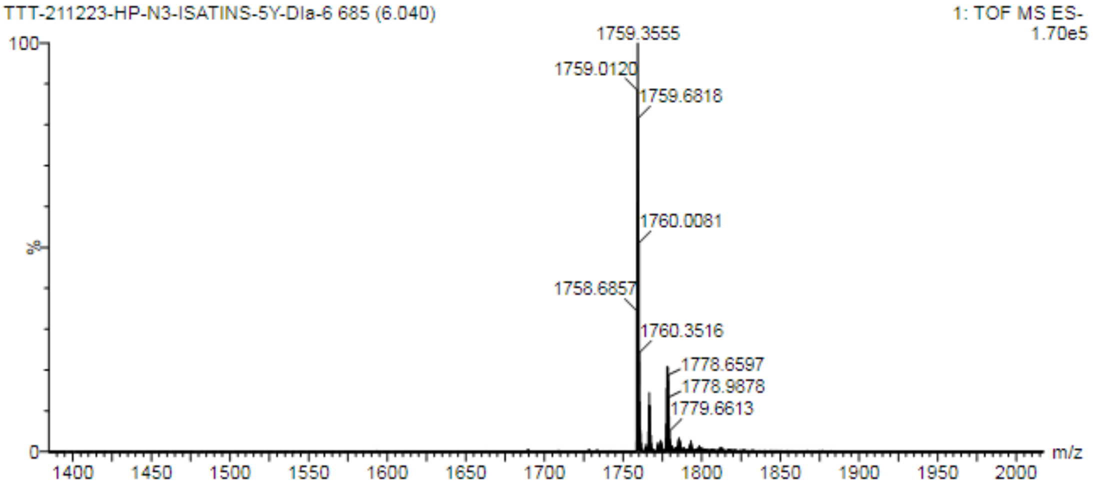

LC Trace and Mass of O16b

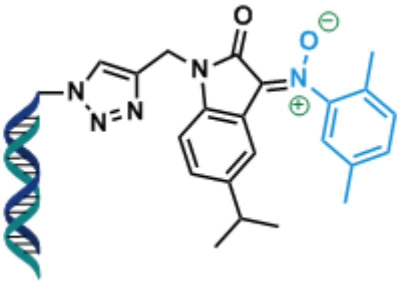

Following General Procedure vi
Yield: 83%
Exact mass: 5309.66
Triply charged mass [M]/3 - 1.00794, calculated 1768.8791; observed 1768.6930.

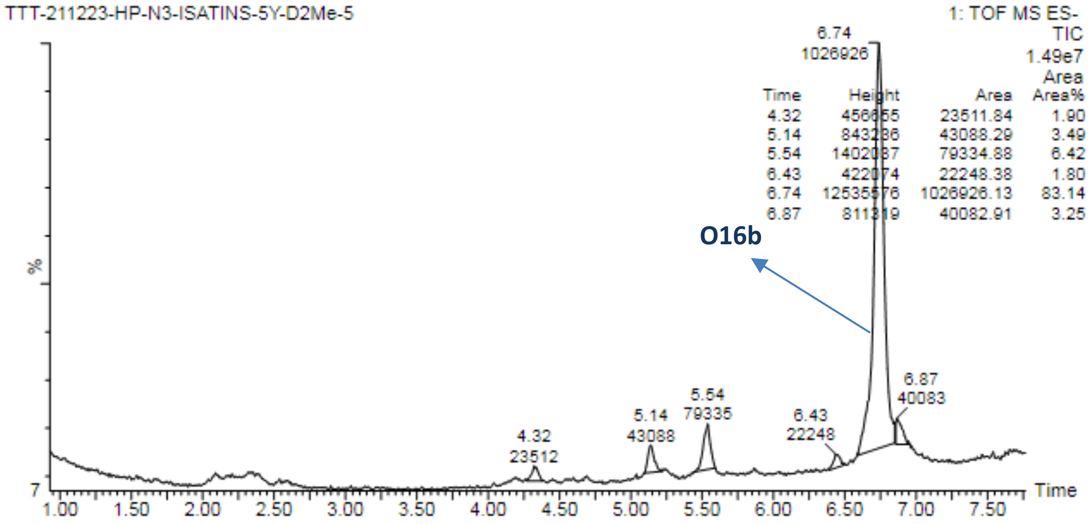

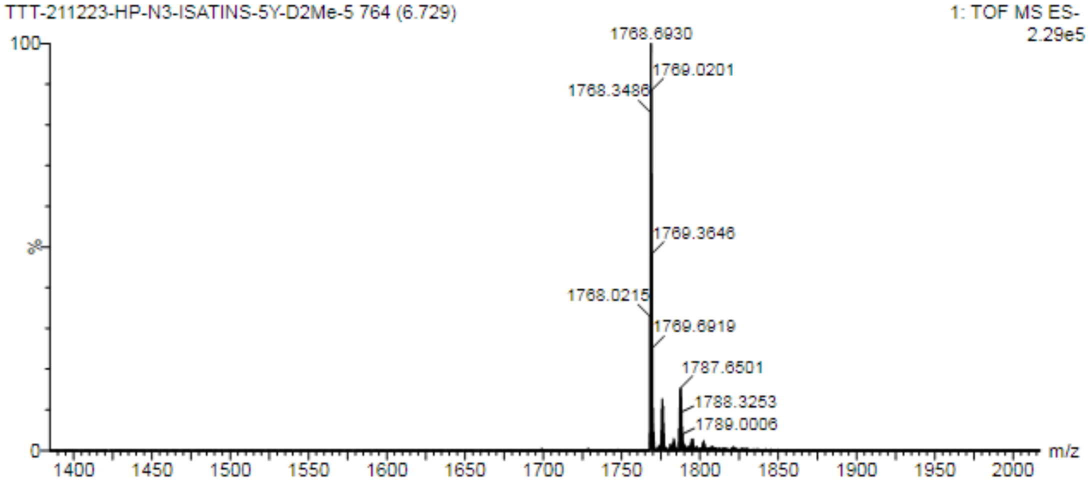

LC Trace and Mass of O16c

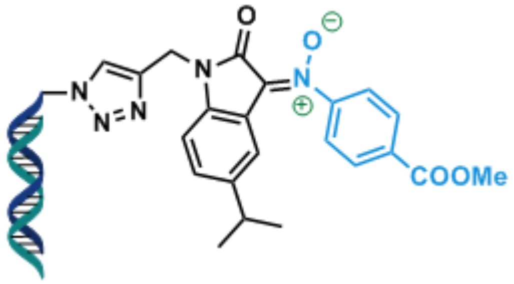

Following General Procedure vi
Yield: 80%
Exact mass: 5339.66
Triply charged mass [M]/3 - 1.00794, calculated 1778.8791; observed 1778.6769.

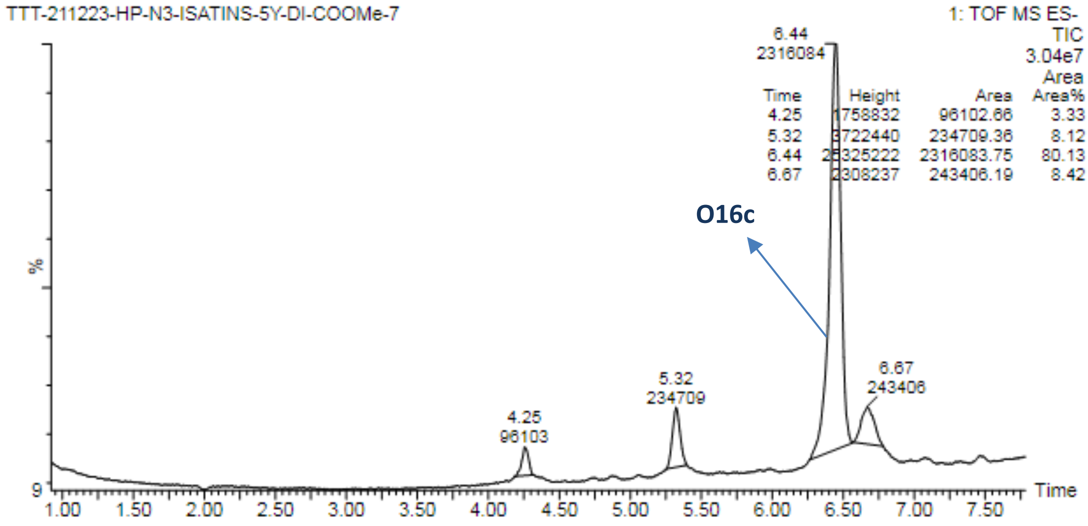

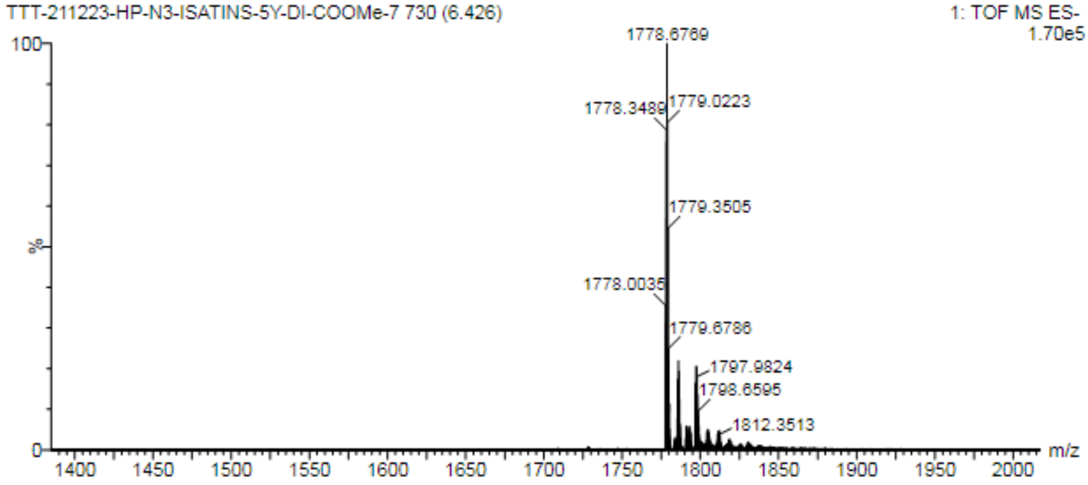

LC Trace and Mass of O17a

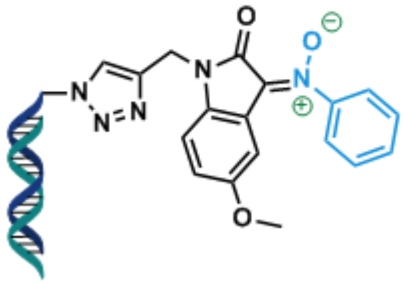

Following General Procedure vi
Yield: 87%
Exact mass: 5269.56
Triply charged mass [M]/3 - 1.00794, calculated 1755.5124; observed 1755.3391.

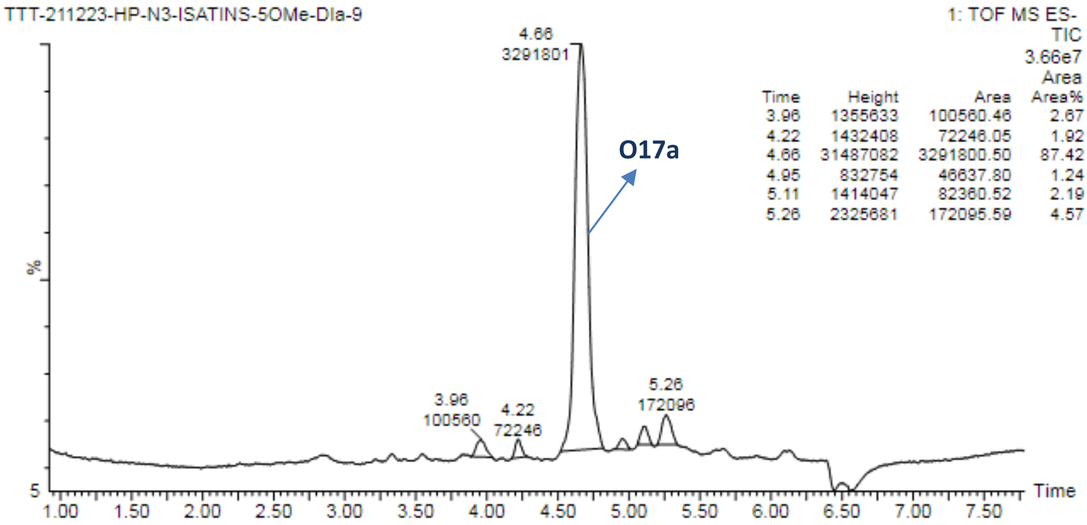

Time	Height	Area	Area%
3.96	1355633	100560.46	2.67
4.22	1432408	72246.05	1.92
4.66	31487082	3291800.50	87.42
4.95	832754	46637.80	1.24
5.11	1414047	82360.52	2.19
5.26	2325681	172095.59	4.57

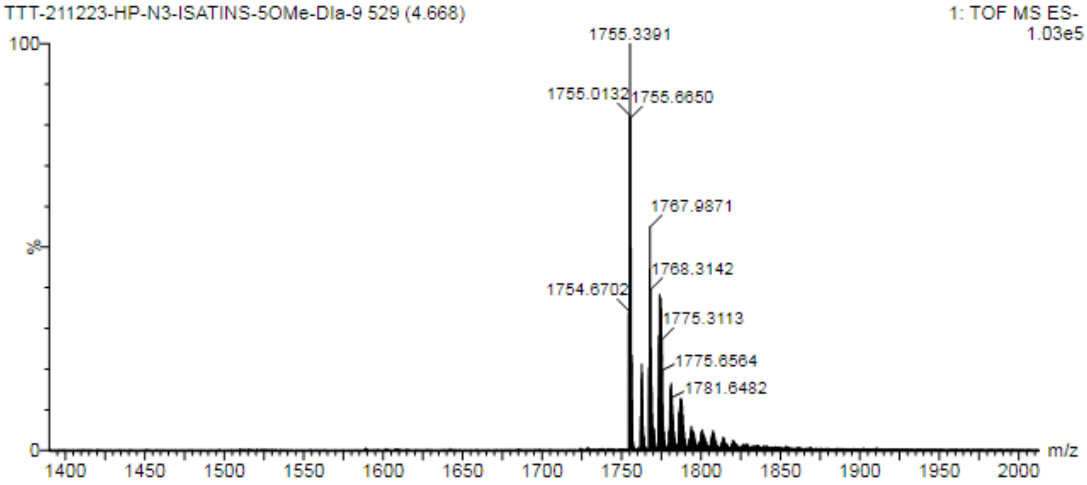

LC Trace and Mass of O17b

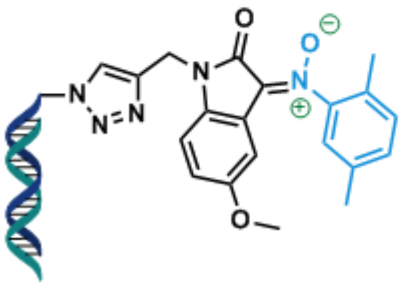

Following General Procedure vi
Yield: 90%
Exact mass: 5297.611
Triply charged mass [M]/3 - 1.00794, calculated 1764.8624; observed 1764.4597.

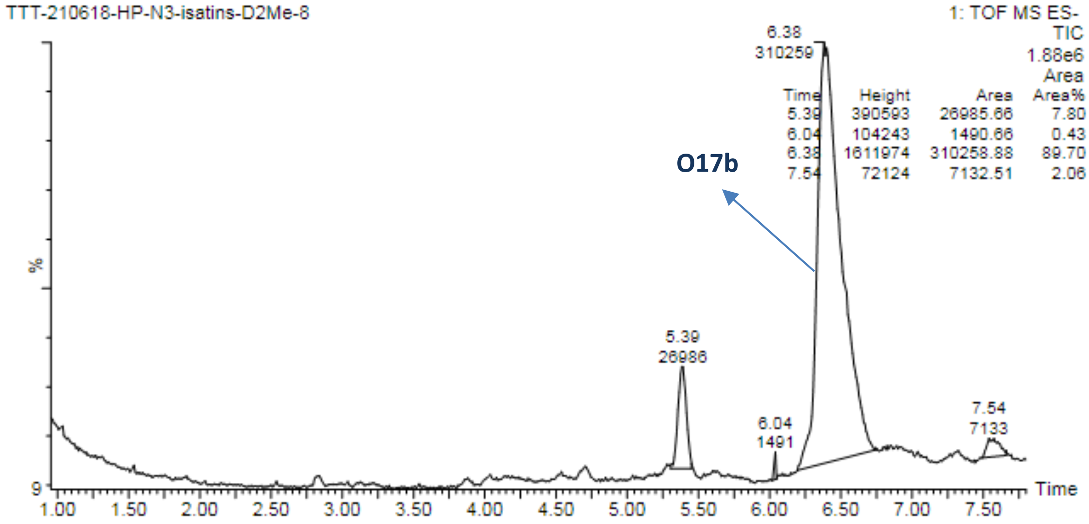

Time	Height	Area	Area%
5.39	390593	26985.66	7.80
6.04	104243	1490.66	0.43
6.38	1611974	310258.88	89.70
7.54	72124	7132.51	2.06

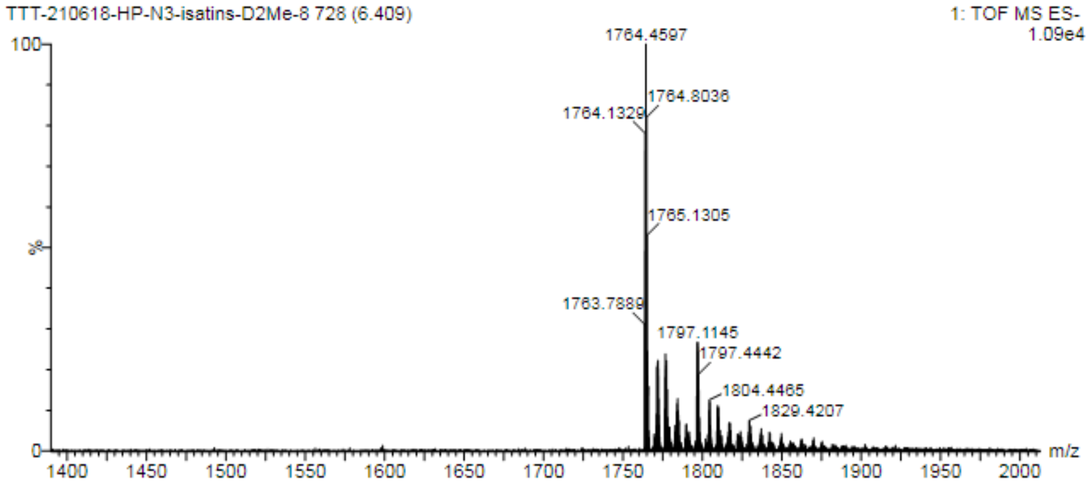

LC Trace and Mass of O17c

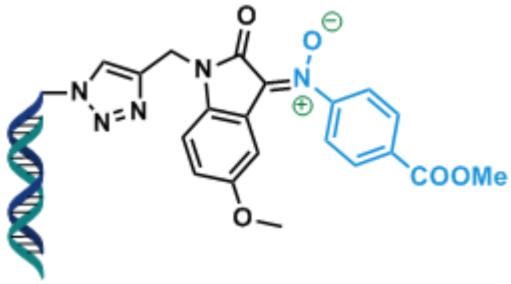

Following General Procedure vi

Yield: 76%

Exact mass: 5327.61

Triply charged mass [M]/3 - 1.00794, calculated 1774.8624; observed 1774.4489.

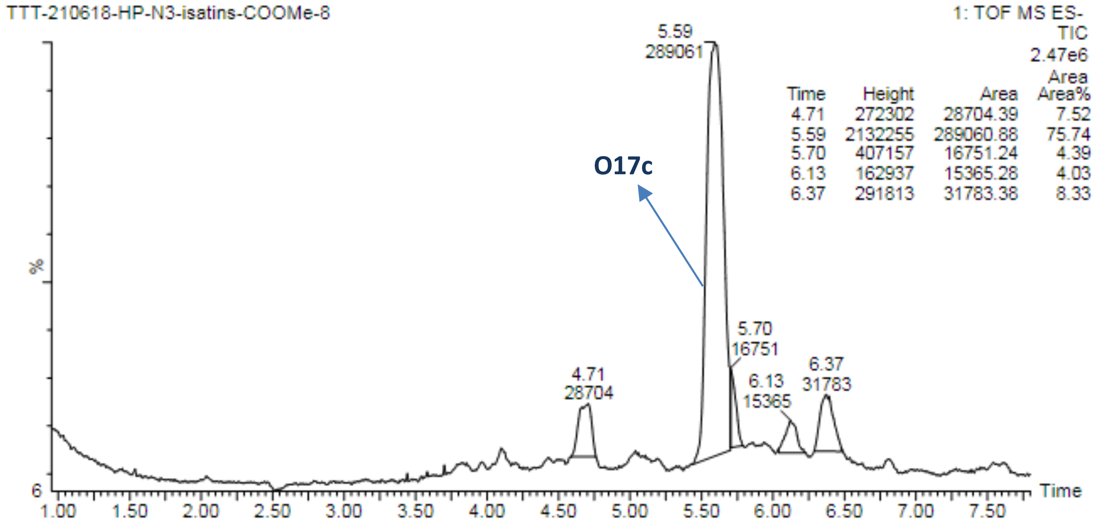

Time	Height	Area	Area%
4.71	272302	28704.39	7.52
5.59	2132255	289060.86	75.74
5.70	407157	16751.24	4.39
6.13	162937	15365.28	4.03
6.37	291813	31783.38	8.33

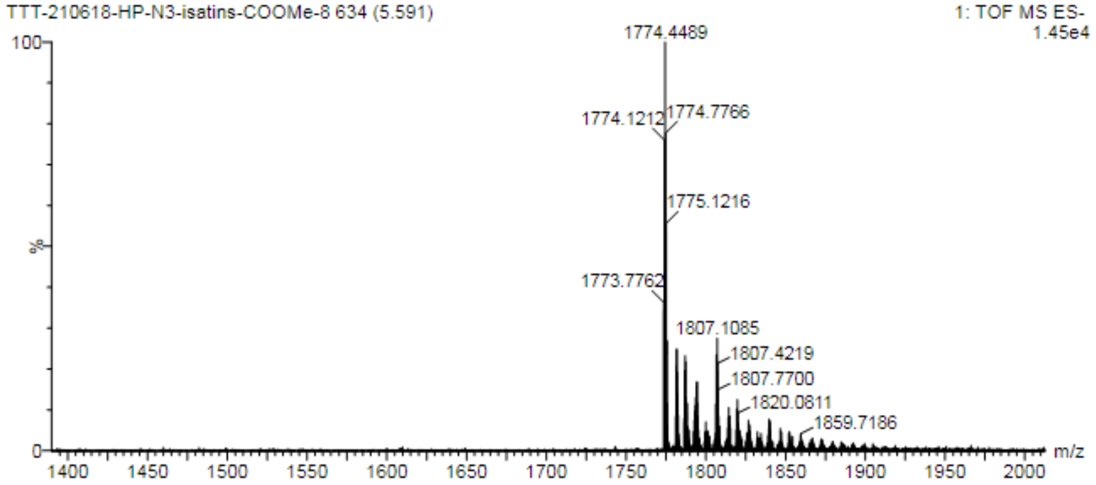

LC Trace and Mass of O18a

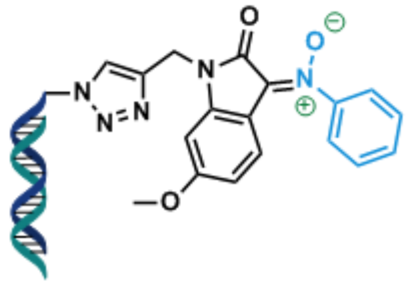

Following General Procedure vi
Yield: 80%
Exact mass: 5269.56
Triply charged mass [M]/3 - 1.00794, calculated 1755.5124; observed 1755.1504.

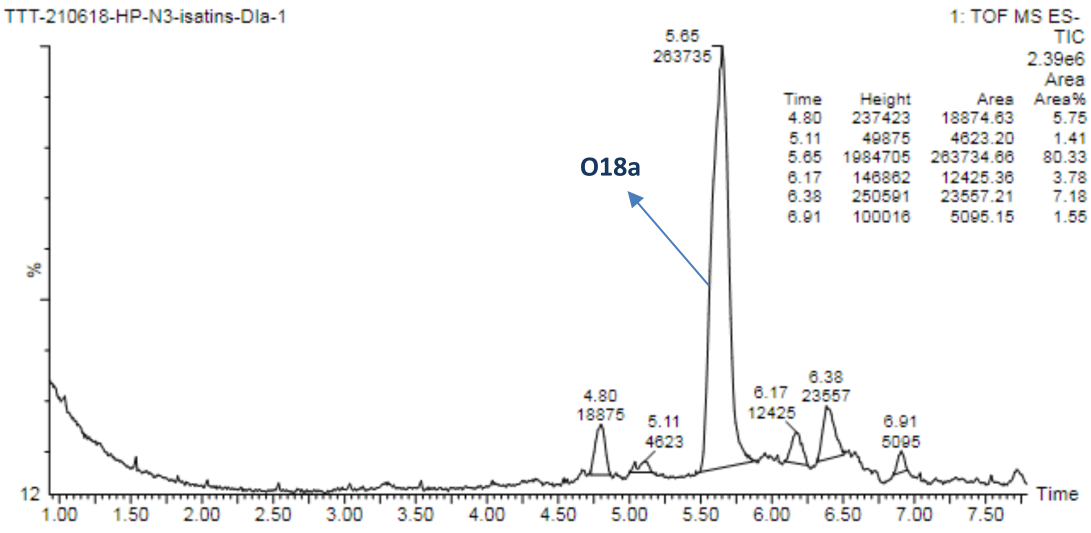

Time	Height	Area	Area%
4.80	237423	18874.63	5.75
5.11	49875	4623.20	1.41
5.65	1984705	263734.66	80.33
6.17	146862	12425.36	3.78
6.38	250591	23557.21	7.18
6.91	100016	5095.15	1.55

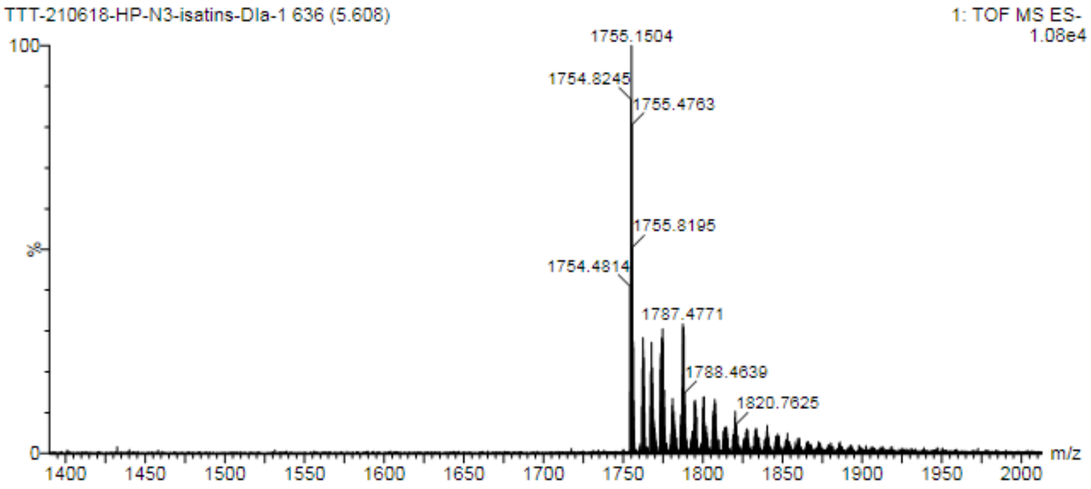

LC Trace and Mass of O18b

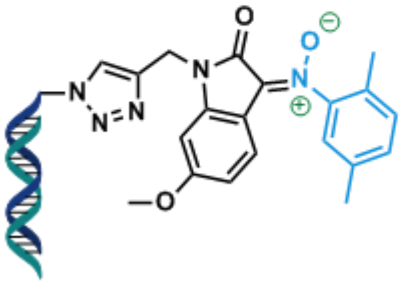

Following General Procedure vi

Yield: 80%

Exact mass: 5297.61

Triply charged mass [M]/3 - 1.00794, calculated 1764.8624; observed 1764.4597.

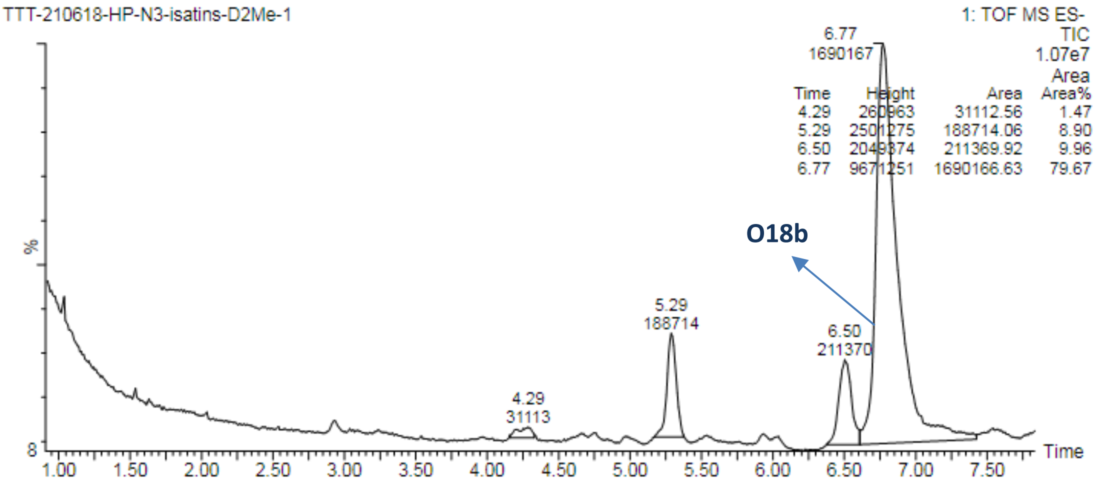

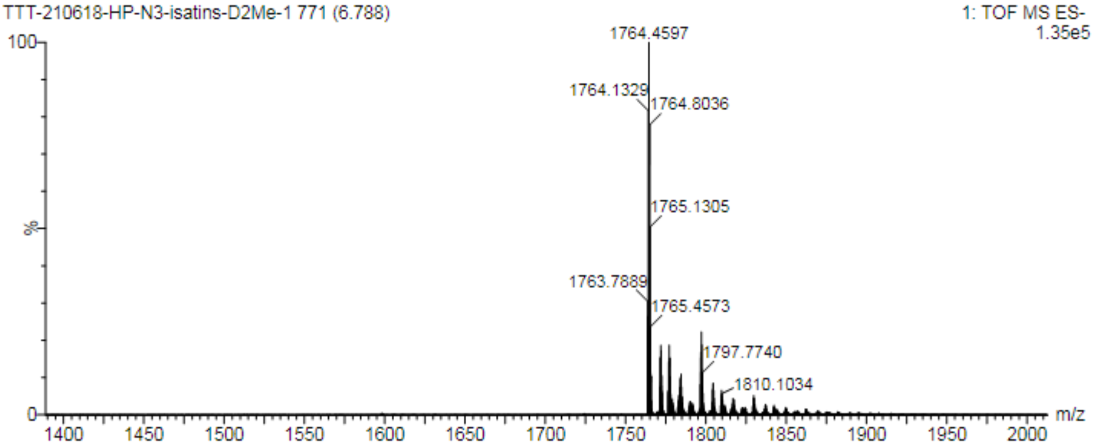

LC Trace and Mass of O18c

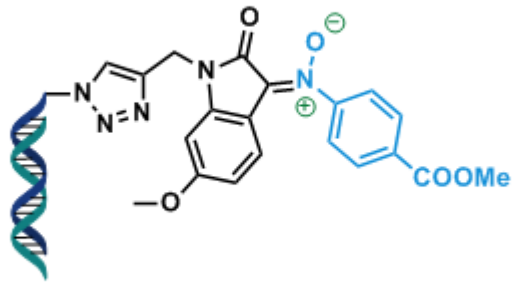

Following General Procedure vi
Yield: 78%
Exact mass: 5327.61
Triply charged mass [M]/3 - 1.00794, calculated 1774.8624; observed 1774.6731.

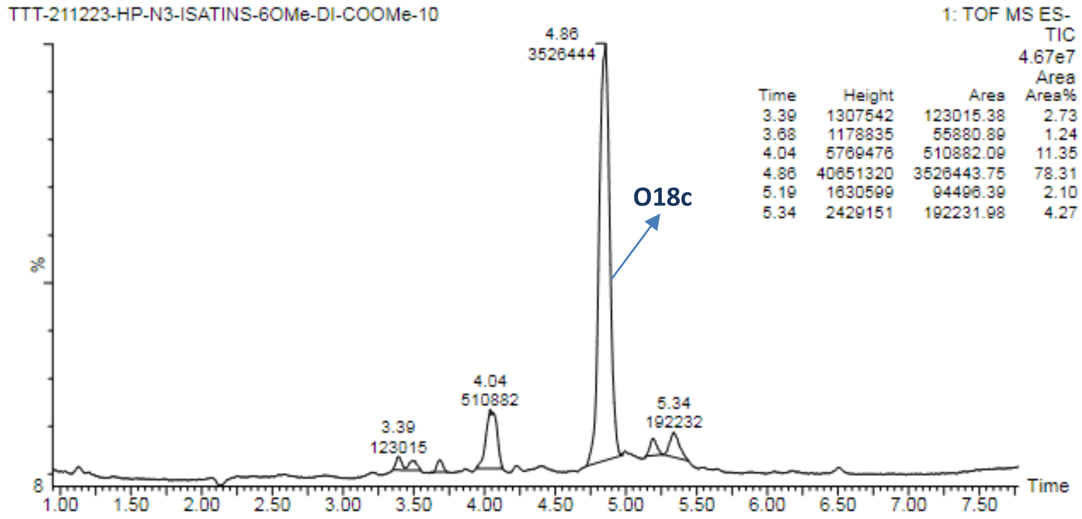

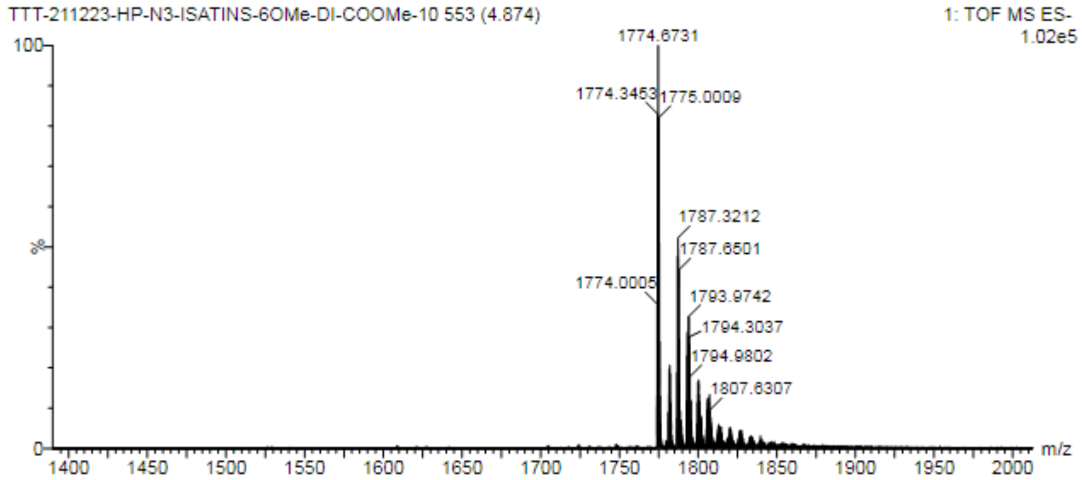

LC Trace and Mass of O19a

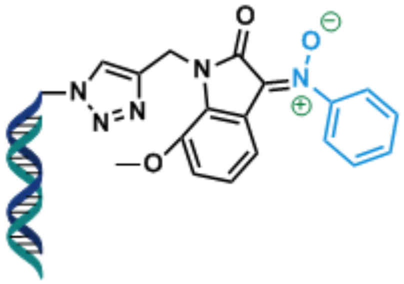

Following General Procedure vi

Yield: 85%

Exact mass: 5269.56

Triply charged mass [M]/3 - 1.00794, calculated 1755.5124; observed 1755.1504.

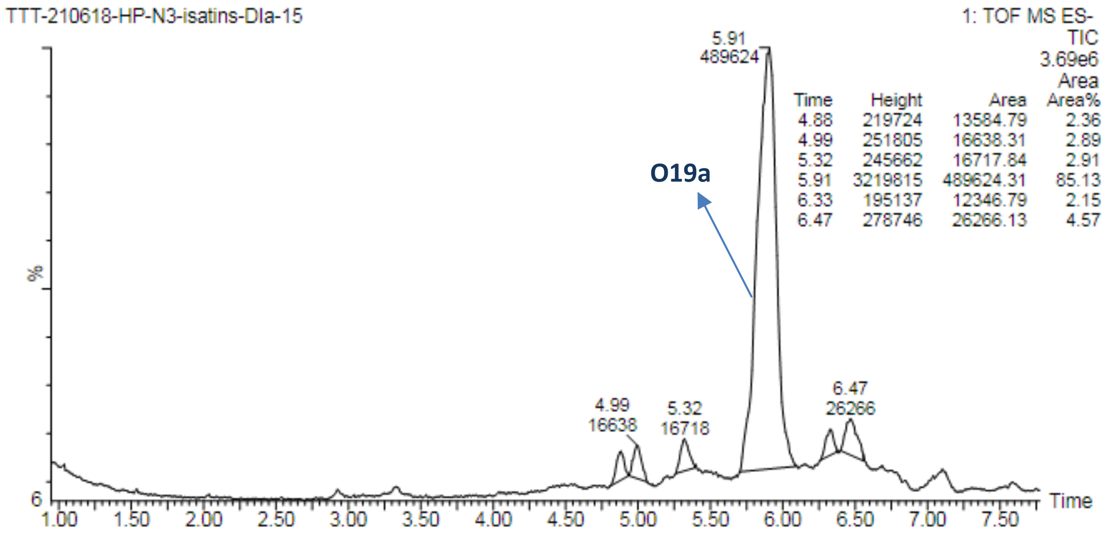

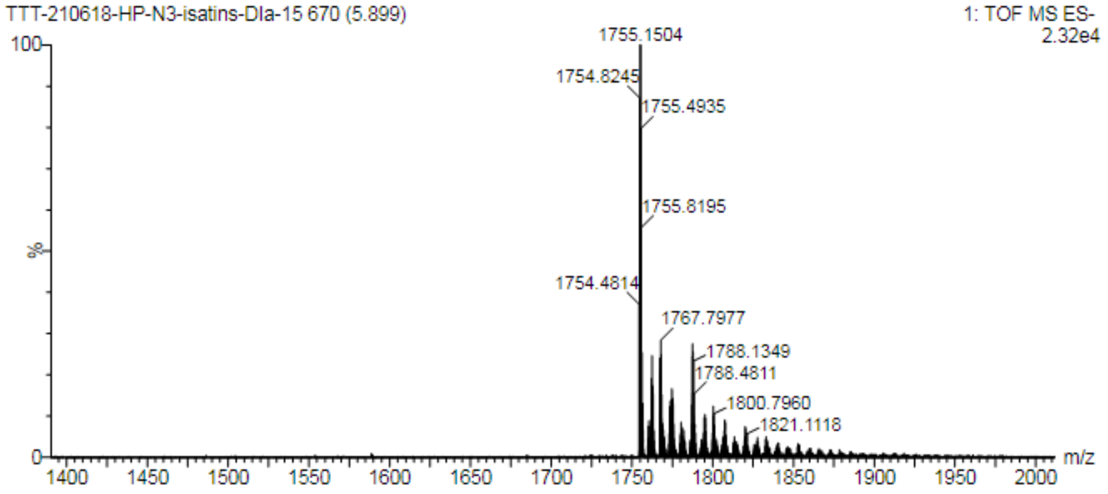

LC Trace and Mass of O19b

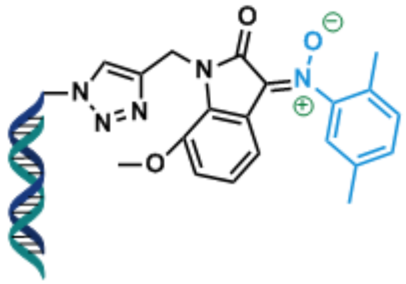

Following General Procedure vi

Yield: 80%

Exact mass: 5297.61

Triply charged mass [M]/3 - 1.00794, calculated 1764.8624; observed 1764.4597.

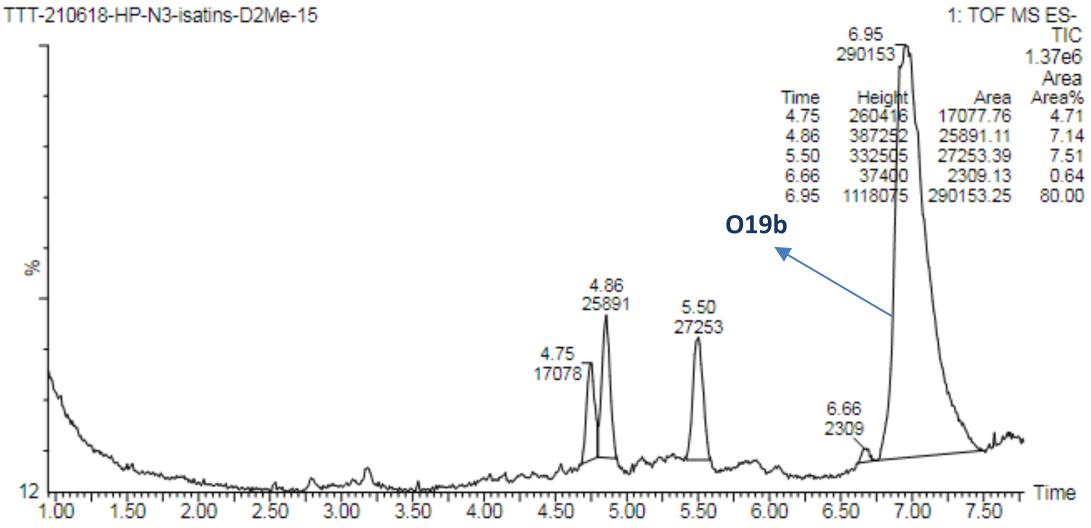

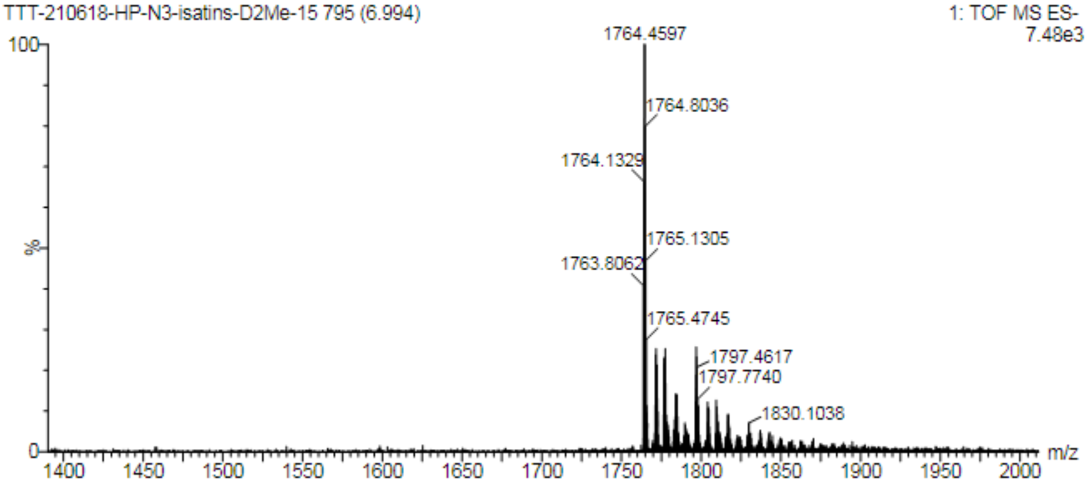

LC Trace and Mass of O19c

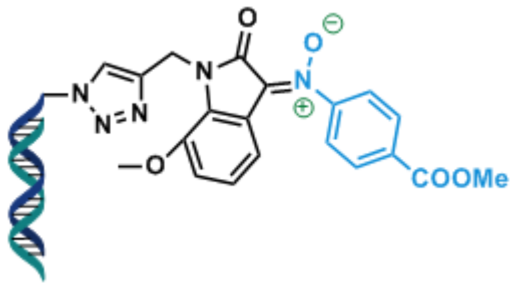

Following General Procedure vi
Yield: 77%
Exact mass: 5327.61
Triply charged mass [M]/3 - 1.00794, calculated 1774.8624; observed 1774.4316.

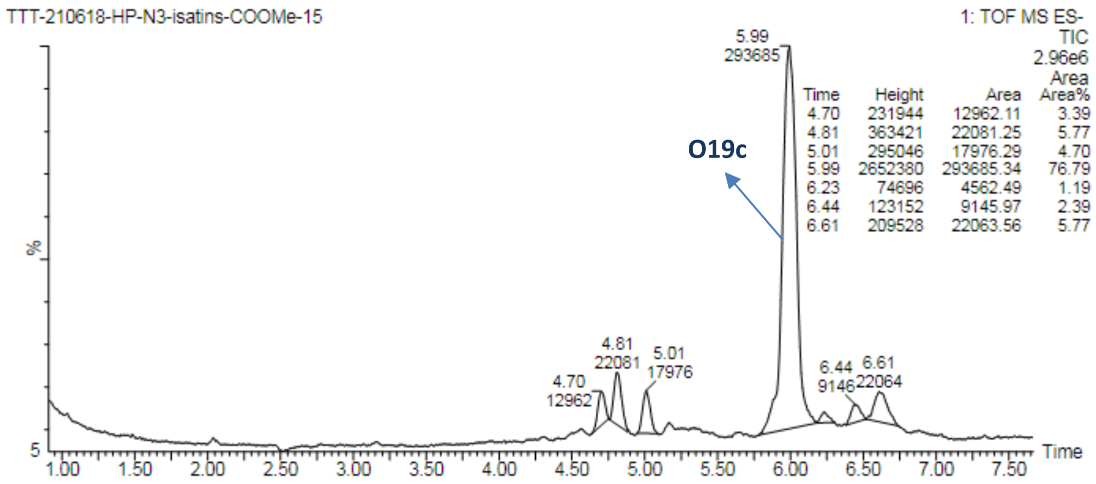

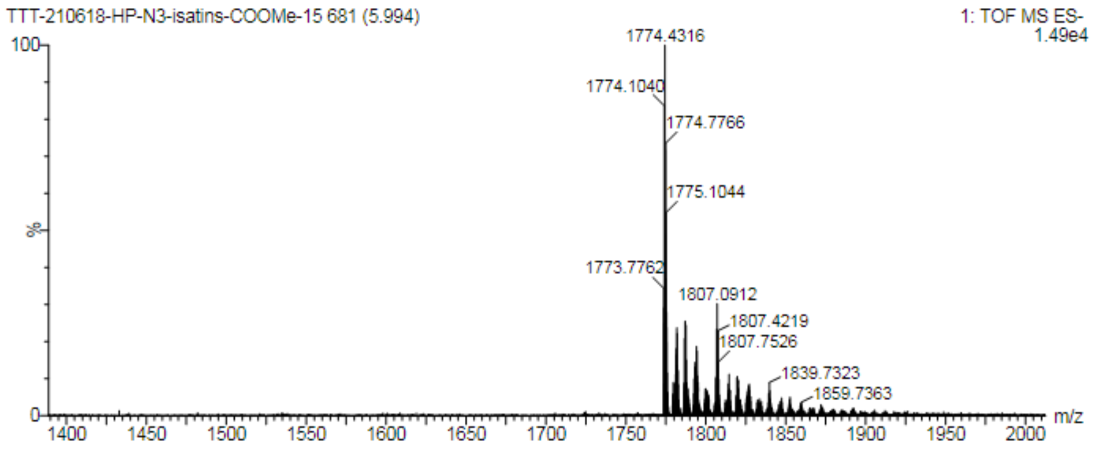

LC Trace and Mass of O20a

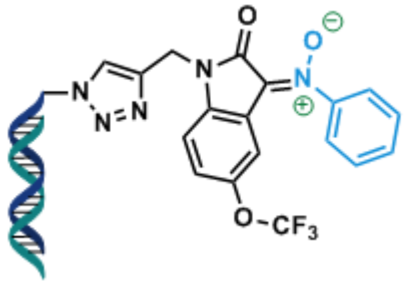

Following General Procedure vi

Yield: 70%

Exact mass: 5323.52

Triply charged mass [M]/3 - 1.00794, calculated 1773.4991; observed 1773.3452.

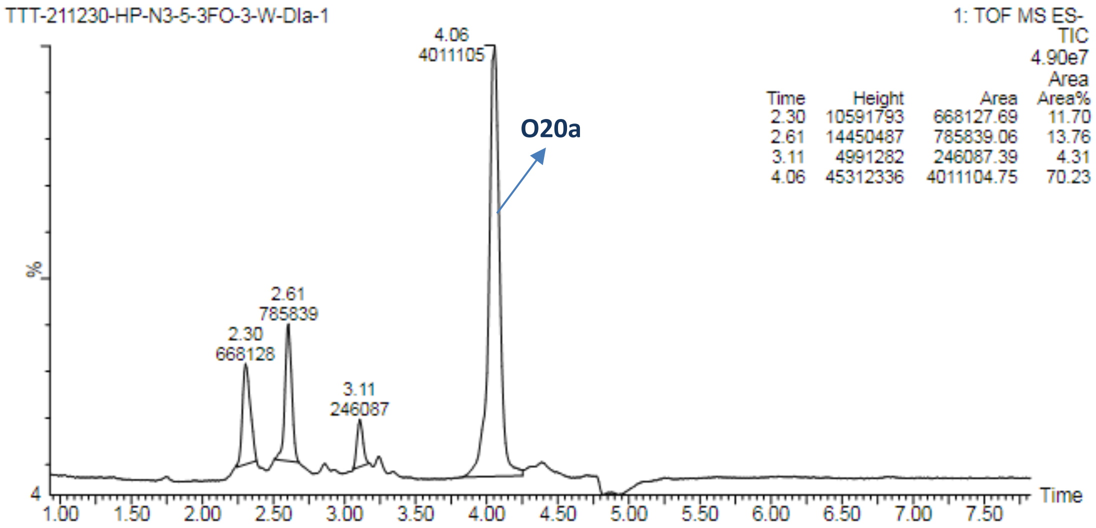

Time	Height	Area	Area%
2.30	10591793	668127.69	11.70
2.61	14450487	785839.06	13.76
3.11	4991282	246087.39	4.31
4.06	45312336	4011104.75	70.23

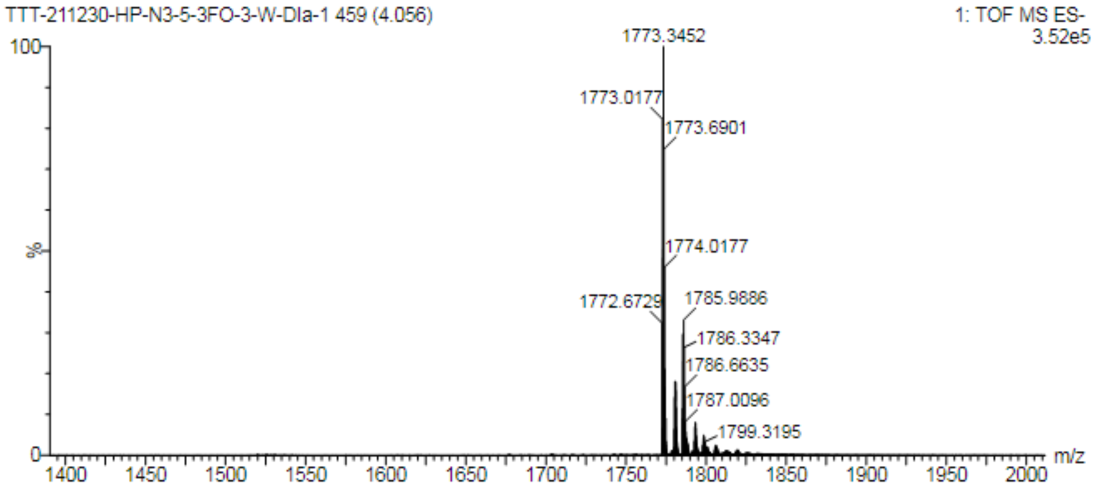

LC Trace and Mass of O20b

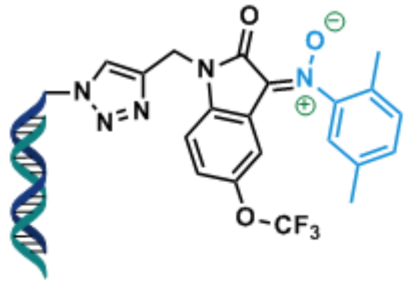

Following General Procedure vi
Yield: 70%
Exact mass: 5351.57
Triply charged mass [M]/3 - 1.00794, calculated 1782.8491; observed 1782.7025.

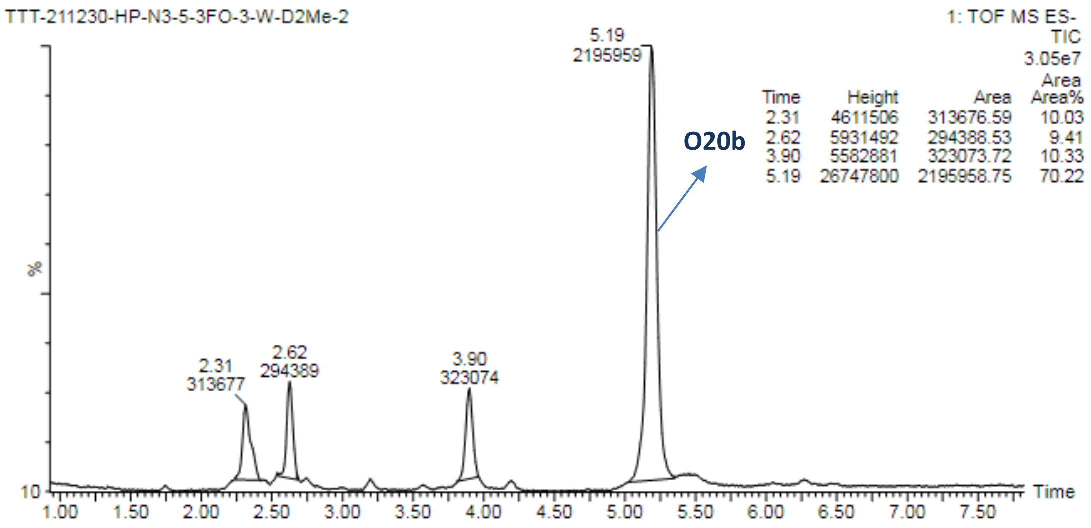

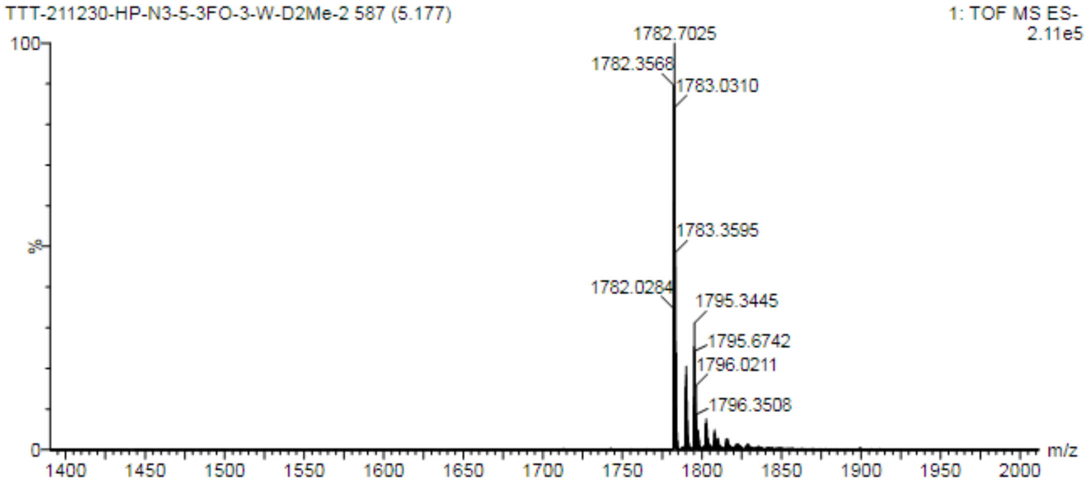

LC Trace and Mass of O20c

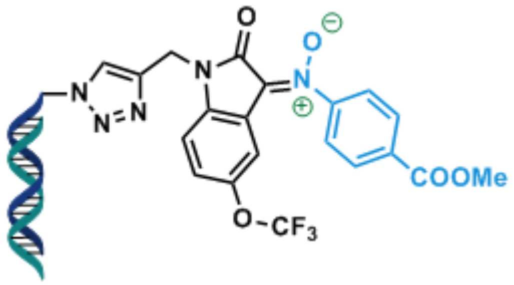

Following General Procedure vi
Yield: 92%
Exact mass: 5381.57
Triply charged mass [M]/3 - 1.00794, calculated 1792.8491; observed 1792.6912.

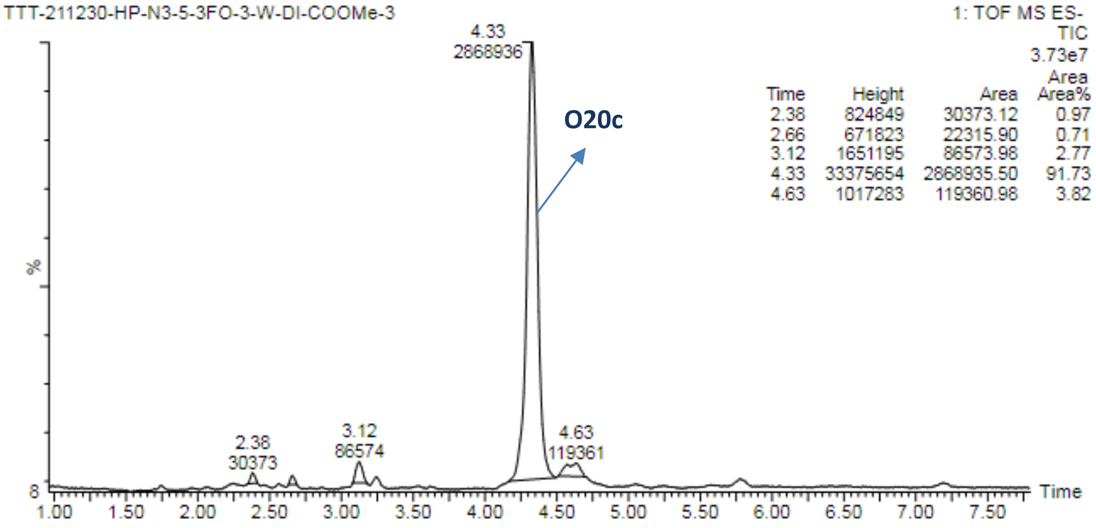

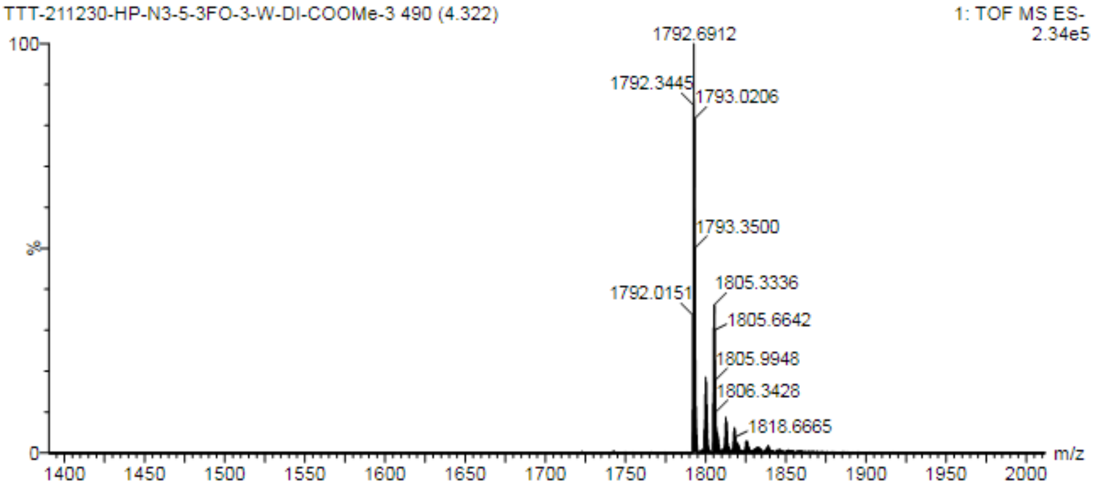

Supplement: Supplementary file 1 — Supporting Information [file ADVS-9-2202790-s001.pdf]
